# Identification and application of keto acids transporters in *Yarrowia lipolytica*

**DOI:** 10.1038/srep08138

**Published:** 2015-01-30

**Authors:** Hongwei Guo, Peiran Liu, Catherine Madzak, Guocheng Du, Jingwen Zhou, Jian Chen

**Affiliations:** 1 School of Biotechnology and Key Laboratory of Industrial Biotechnology, Ministry of Education, Jiangnan University, 1800 Lihu Road, Wuxi, Jiangsu 214122, China; 2 UMR1238 Microbiologie et Génétique Moléculaire, INRA/CNRS/AgroPan's Tech, CBAI, BP 01, 78850 Thiverval-Grignon, France; 3 Synergetic Innovation Center of Food Safety and Nutrition, 1800 Lihu Road, Wuxi, Jiangsu 214122, China

## Abstract

Production of organic acids by microorganisms is of great importance for obtaining building-block chemicals from sustainable biomass. Extracellular accumulation of organic acids involved a series of transporters, which play important roles in the accumulation of specific organic acid while lack of systematic demonstration in eukaryotic microorganisms. To circumvent accumulation of by-product, efforts have being orchestrated to carboxylate transport mechanism for potential clue in *Yarrowia lipolytica* WSH-Z06. Six endogenous putative transporter genes, YALI0B19470g, YALI0C15488g, YALI0C21406g, YALI0D24607g, YALI0D20108g and YALI0E32901g, were identified. Transport characteristics and substrate specificities were further investigated using a carboxylate-transport-deficient *Saccharomyces cerevisiae* strain. These transporters were expressed in *Y*. *lipolytica* WSH-Z06 to assess their roles in regulating extracellular keto acids accumulation. In a *Y*. *lipolytica* T1 line over expressing YALI0B19470g, α-ketoglutarate accumulated to 46.7 g·L^−1^, whereas the concentration of pyruvate decreased to 12.3 g·L^−1^. Systematic identification of these keto acids transporters would provide clues to further improve the accumulation of specific organic acids with higher efficiency in eukaryotic microorganisms.

Light have been shed on economic feasibility of production of organic acids by microorganisms, expecially considering the finiteness of fossil raw materials^1^. Although organic acids occupy a key position among the building-block chemicals, economic production by microorganisms is still a big chagllenge to replace petroleum-derived commodity chemicals^2^. In particular, bio-based production of organic acids is unharnessed by low titres, low yield and accumution of by-products^3^,^4^,^5^.

The metabolism and accumulation of α-ketoglutaric acid (α-KG) are subjected to a higher level of regulation than other organic acids involved in central carbon metabolism^6^, as α-KG occupies a key position in both carbon central metabolism^7^ and the regulation of the carbon–nitrogen balance^8^. Metabolic strategies concerning the following have been investigated for α-KG production: regulation of key enzymes, including pyruvate dehydrogenase complex^9^, pyruvate carboxylase^10^, fumarase^3^, aconitase^11^, isocitrate lyase^12^, isocitrate dehydrogenase^13^ and components of the α-ketoglutarate dehydrogenase complex^14^; and co-factor engineering of acetyl-CoA biosynthesis and regeneration^15^.

Extracellular accumulation of non-target carboxylates is a common problem for the production of carboxylates^16^,^17^. Manipulation of transporters has been an efficient tool to improve the productivity for target carboxylates^5^. A successful metabolic engineering approach for the over-synthesis of organic acids also requires incorporation of an appropriate exporter to increase productivity^18^. A comparative study revealed that the highest malate yield was obtained once the malate transporter was recruited in the mutants^19^. Lactate production in *S*. *cerevisiae* could be significantly improved with the combined expression of lactate dehydrogenase and the lactate transporter^20^. Although many studies have described the mechanisms of action and regulation for carboxylate transport in yeast^20^,^21^, *Y*. *lipolytica* orthologous gene of the carboxylate transporter have not yet been identified.

In this study, a number of carboxylate transporters were identified from *Y*. *lipolytica* WSH-Z06, which exhibits flexibility in substrate specificity. The duplication of endogenous transporters might provide a powerful tool to ensure efficient carboxylate synthesis and maintain homeostasis of the intracellular environment. In this study, we observed that a competitive dual effect — a significant increase in α-KG production with a sharp decrease in pyruvate (PA) accumulation resulted from overexpression of YALI0B19470g. This result suggests a new and promising strategy for gene manipulation that can efficiently address α-KG accumulation. The identification of these transporters also have uncovered the mechanisms of extracellular accumulation of diverse organic acids in genome level, and provides new clues to orchestrate competition of extracellular accumulation between target and non-target carboxylates in yeast and other eukaryotic microorganisms.

## Results

### Bioinformatics analysis of potential carboxylate transporters in *Y. lipolytica*

**Figure 1:**
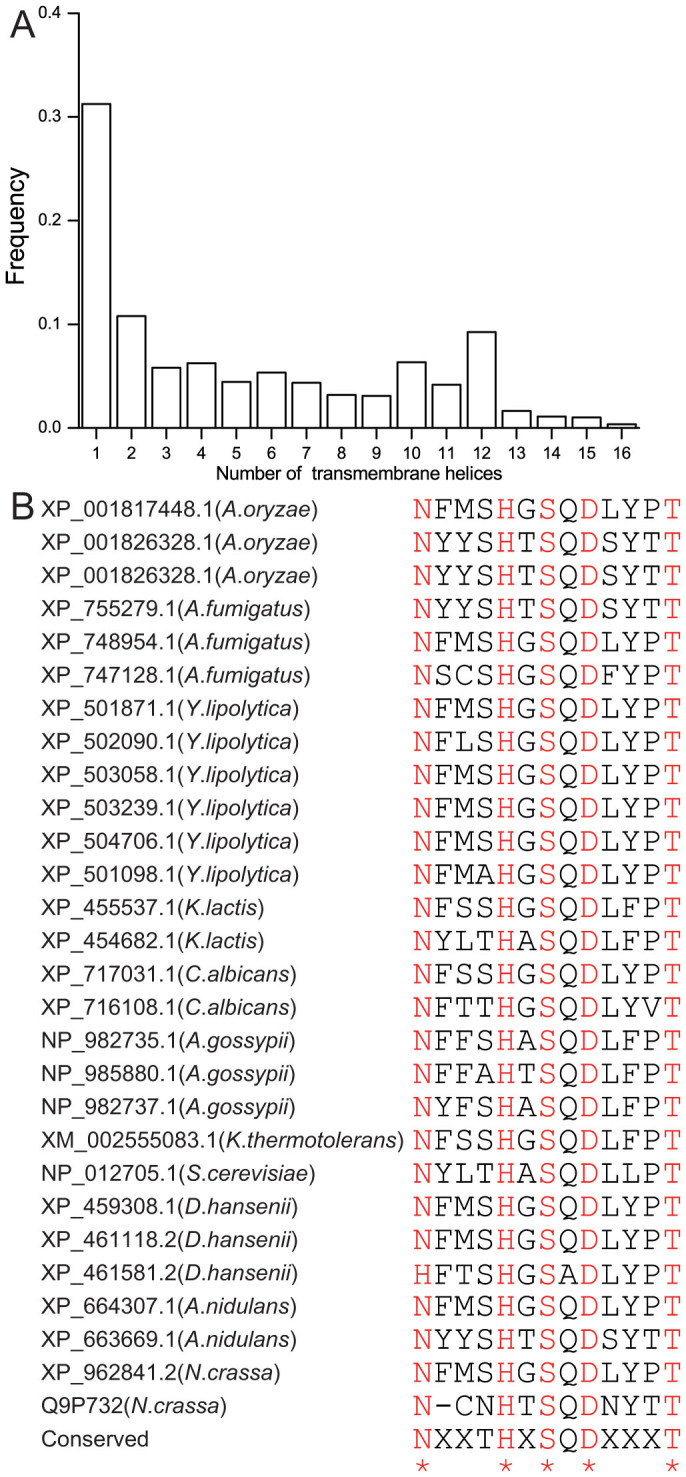
Genome-wide prediction of transmembrane proteins in *Y. lipolytica*. (A) Number of predicted transmembrane helices in identified putative membrane proteins. (B) Multiple sequence alignment of amino acids sequences from available yeast carboxylate transporter sequences. The results showed that 28 orthologs of the *JEN* family from *Aspergillus oryzae*, *Aspergillus fumigatus*, *Aspergillus nidulans*, *Y*. *lipolytica*, *Kluyveromyces lactis, Candida albicans*, *Kluyveromyces thermotolerans*, *S*. *cerevisiae*, *Debaryomyces hansenii* and *Neurospora crassa* had highly conserved sequences. The name of these orthologs was based on protein ID in NCBI database, as one potential *JEN1* from *N*. *crassa* could not find in the database, it was named as Q9P732 based on protein ID from Uniprot.

To screen putative carboxylate transporters, 6611 proteins encoded by *Y*. *lipolytica* CLIB122 genome were obtained from the UniProt database. Of these, 1104 proteins (Supplementary S1) were predicted as transmembrane proteins by TMHMM v.2.0 (Fig. 1A). Subsequently, 117 proteins were excluded from this set, due to the presence of a possible signal peptide at the N terminus, as predicted by SignalP 4.1. A sequence search using a conserved carboxylate transporter signature of *JEN* family, NXXS/THXS/TQDXXXT, identified six putative proteins encoded by the YALI0B19470g, YALI0C15488g, YALI0C21406g, YALI0D24607g, YALI0D20108g, and YALI0E32901g genes, which exhibited high similarity to the signature sequence. A multiple sequence alignment of these sequences using homologous carboxylate transporter sequences from other fungi confirmed the presence of the conserved sequence (Fig. 1B).

**Figure 2:**
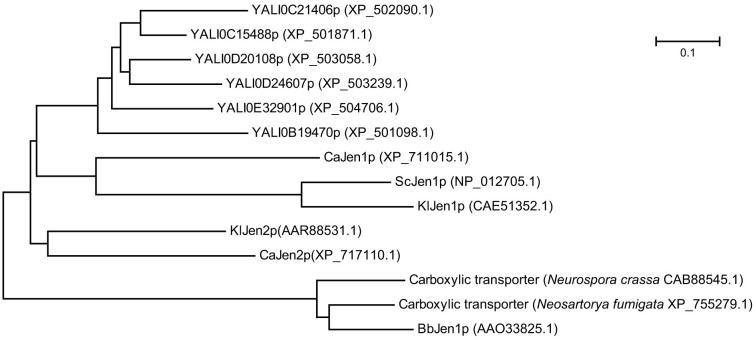
Phylogenetic analysis of putative transporters. The phylogenetic tree of carboxylate proteins was constructed using the neighbor-joining method. Bootstrap values >50% are shown at the branch points. One thousand bootstrap replications were performed using the MEGA 5.0 software^48^.

**Figure 3:**
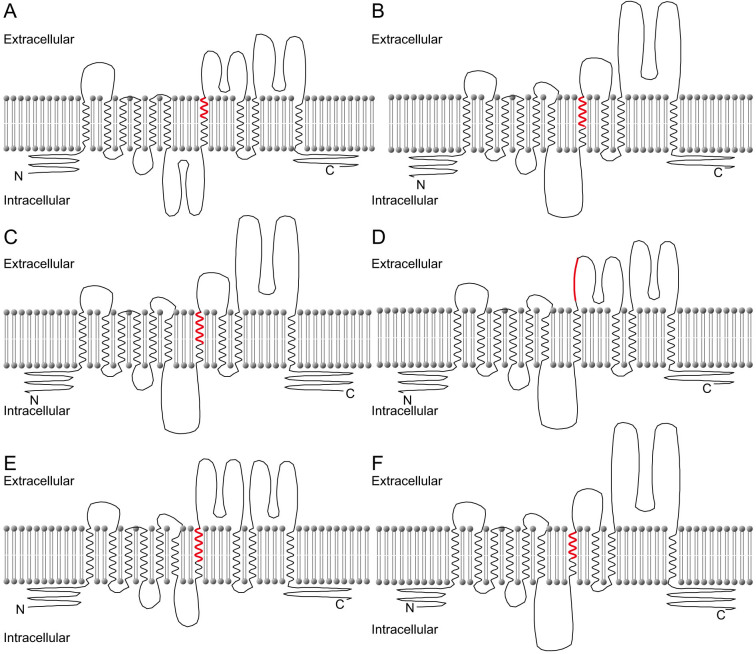
Sequence-structure mapping of putative transporters. (A) YALI0B19470p, (B) YALI0C15488p, (C) YALI0C21406p, (D) YALI0D24607p, (E) YALI0D20108p, (F) YALI0E32901p. The conserved signature sequence was illustrated in red in every putative carboxylate transporter.

In addition, a high level of sequence similarity among these putative transporters and the characterized carboxylate transporters was illustrated by two BLAST (http://blast.ncbi.nlm.nih.gov/) protocols, in which ScJen1p and KlJen2p were used as query sequences^22^,^23^,^24^. In these cases, the identity to ScJen1p and to KlJen2p were prospected, respectively (Fig. 2). *In silico* analysis of these protein sequences using the TMRPres2D tool revealed that the conserved sequence was located in the predicted transmembrane helices (Fig. 3). Hence, these transmembrane proteins were suspected to be potential carboxylate transporters.

### Exogenous keto acid treatment

**Figure 4:**
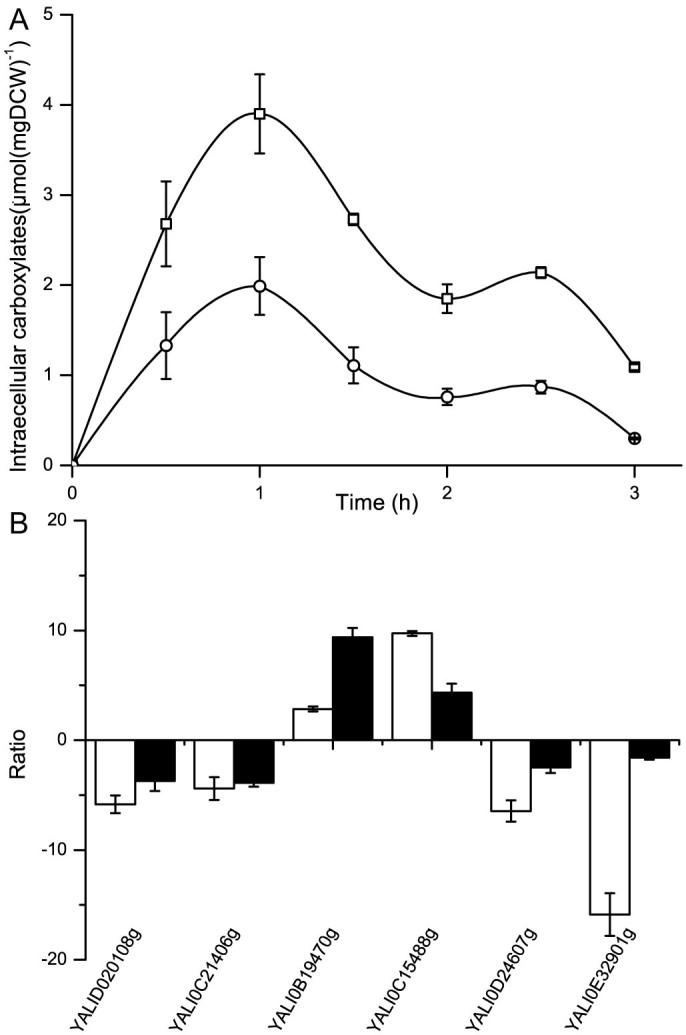
Time courses of intracellular carboxylate content in *Y*. *lipolytica* WSH-Z06. (A) Open square: α-KG, open circle: PA. The change of concentration of α-KG and PA exhibited the same trend during throughout the treatment. (B) Expression profile of putative transporter genes at 1 h, at which time the intracellular contents of α-KG and PA were greatest. White: PA treatment; black: α-KG treatment.

To uncover the response of carboxylate transporter candidates for uptake of exogenous keto acids, glucose-grown (repression condition) *Y*. *lipolytica* WSH-Z06 cells were transferred at the exponential phase to YPK or YPP medium (A large amount exogenous organic acids were accumulated in yeast cells, Supplementary S2). Initially, neither α-KG nor PA was detected in these cultures. During the first hour, the intracellular concentration of both carboxylates increased quickly to maximums of 3.90 μmol·(mg·DCW)^−1^ for α-KG and 1.67 μmol·(mg·DCW)^−1^ for PA in α-KG- and PA-treated cells, respectively. After incubation in YPK or YPP medium for 1 h, the concentration of α-KG gradually decreased 1 h to 3 h, until the minimum of 0.85 μmol·(mg·DCW)^−1^ was reached. During this period, the content of PA also decreased to 0.23 μmol·(mg·DCW)^−1^ (Fig. 4A).

To assay the effects of exogenous carboxylates on the expression profiles of these potential transporters, the expression of these genes were examined by qRT-PCR, using RNA extracted at 0 h as the control. When intracellular α-KG levels increased, the expression of YALI0D20108g, YALI0C21406g, YALI0D24607g, and YALI0E32901g decreased by 3.72 ± 0.91-fold, 3.88 ± 0.34-fold, 2.47 ± 0.52-fold, and 1.60 ± 0.18-fold, respectively, whereas the expression of YALI0B19470g and YALI0C15488g increased by 9.39 ± 0.82-fold and 4.32 ± 0.83-fold, respectively. Exogenous PA had a similar effect on expression, albeit to different levels. When incubated with PA, expression of YALI0D20108g, YALI0C21406g, YALI0D24607g, and YALI0E32901g decreased by 5.83 ± 0.81-fold, 4.04 ± 1.04-fold, 6.44 ± 0.98-fold, and 15.88 ± 1.94-fold, respectively, whereas expression of YALI0B19470g and YALI0C15488g increased by 2.83 ± 0.21-fold and 9.72 ± 0.22-fold, respectively (Fig. 4B). Hence, we concluded that transport of both keto acids should be predominantly mediated by YALI0B19470p and YALI0C15488p.

### Assessment of putative genes using heterologous expression in *S*. *cerevisiae*

**Figure 5:**
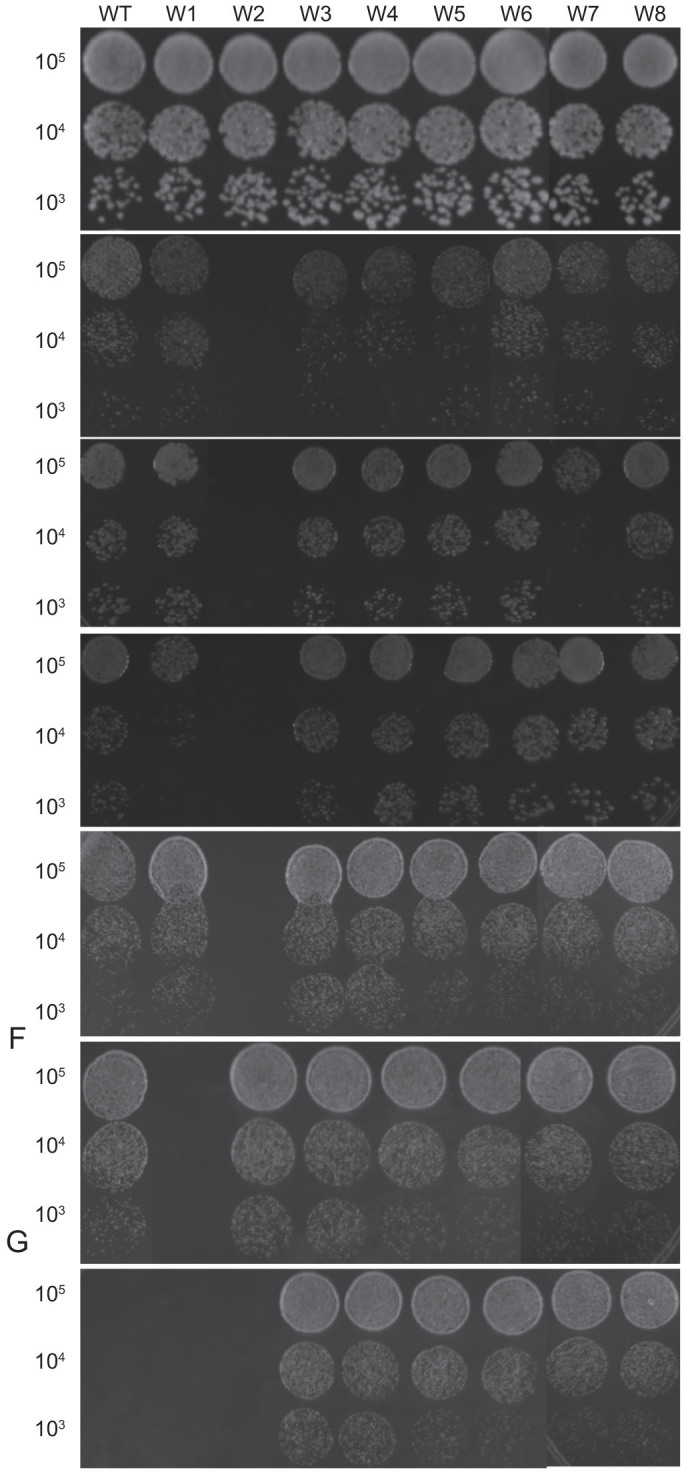
Growth test of *S*. *cerevisiae* strains under different carboxylates. WT: *S*. *cerevisiae* CEN.PK2-1D; W1: *S*. *cerevisiae* CEN.PK2-1D Δ*jen1*; W2: *S*. *cerevisiae* CEN.PK2-1D Δ*jen1*Δ*ady2*; W3: *S*. *cerevisiae* CEN.PK2-1D Δ*jen1*Δ*ady2* (pY13-TEF1-470); W4: *S*. *cerevisiae* CEN.PK2-1D Δ*jen1*Δ*ady2* (pY13-TEF1-488); W5: *S*. *cerevisiae* CEN.PK2-1D Δ*jen1*Δ*ady2* (pY13-TEF1-406); W6: *S*. *cerevisiae* CEN.PK2-1D Δ*jen1*Δ*ady2* (pY13-TEF1-607); W7: *S*. *cerevisiae* CEN.PK2-1D Δ*jen1*Δ*ady2* (pY13-TEF1-108); W8: *S*. *cerevisiae* CEN.PK2-1D Δ*jen1*Δ*ady2* (pY13-TEF1-901). (A) YPD, (B) YPA, (C) YPL, (D) YPP, (E) YPM, (F) YPK, (G) YPC.

To assess their possible roles in carboxylate transport, null mutants of endogenous transporters were constructed by gene disruption in *S*. *cerevisiae* CEN.PK2-1D. The *ScJEN1* deletion mutant displayed reduced growth on lactate, acetate, PA, malate, and α-KG compared to the parental strain. To completely abolish the uptake by endogenous transporters, the Ady2p was also disrupted in W1 strain. In contrast, the W2 strain, in which both *ScJEN1* and *ScADY2* were disrupted, cannot grow on lactate, acetate, PA, malate, and α-KG containing media. Moreover, the parental strain as well as the W1 and W2 strains did not grow on citrate (Fig. 5). All putative transporter genes were introduced into the W2 strain by genetic transformation based on Δ*his3* complementation. The resulting strains grew well on all sources including citrate, supporting the conclusion that these genes encode carboxylate transporters (Fig. 5).

### Substrate specificity assay

To investigate the substrate specificity of these proteins, glucose-grown *S*. *cerevisiae* CEN.PK2-1D and mutants were incubated in YPA, YPL, YPP, YPM, YPK, and YPC medium for 2 h after exhaustion of the endogenous carbon source in saline water. The intracellular carboxylate content in *S*. *cerevisiae* CEN.PK2-1D cells was measured (Table 1), revealing that 2.22 ± 0.21 μmol·(mg·DCW)^−1^ lactate, 0.94 ± 0.14 μmol·(mg·DCW)^−1^ PA, 3.14 ± 0.24 μmol·(mg·DCW)^−1^ malate, and 1.29 ± 0.12 μmol·(mg·DCW)^−1^ α-KG were present. In addition, acetate and citrate were not detected in cells incubated in the corresponding carboxylate-containing medium. As no carboxylates were detected in the double deletion cells, we concluded that ScJen1p and ScAdy2p deletions influenced the transport of PA, malate, and α-KG.

The transport of α-KG was restored in all heterogeneous transporter-containing strains, with the highest accumulation (1.41 μmol·(mg·DCW)^−1^) occurring in the W4 strain. Among the set, only the W4 could accumulate PA intracellularly (0.17 μmol·(mg·DCW)^−1^). Based on these observations, we conclude that YALI0B19470g and YALI0D24607g encode proteins that transport dicarboxylates and tricarboxylates. In addition to PA, W4 cells (containing YALI0C15488p) also accumulated acetate, malate, α-KG, and citrate, indicating the corresponding protein also transported these carboxylates. In a similar manner, we conclude the proteins encoded by the corresponding genes transport the following carboxylates: YALI0C21406p for lactate, malate, α-KG, and citrate; YALI0D20108p for lactate, α-KG and citrate, and YALI0E32901p for acetate, malate, and citrate.

### Copy number analysis of putative transporter genes in recombinant strains

**Figure 6:**
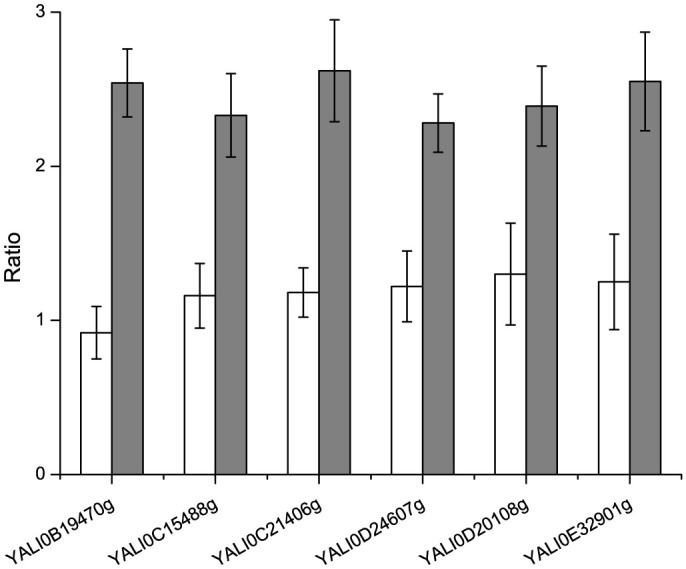
Copy number analysis of putative transporter genes in recombinant strains. Copy number of YALI0B19470g in T1, YALI0C15488g in T2, YALI0C21406g in T3, YALI0D24607g in T4, YALI0D20108g in T5 and YALI0E32901g in T6 were determined through qPCR analysis. As *ACT1* existed in one copy per *Y*. *lipolytica* genome, copy number of putative transporter genes in wild-type strain (white) and recombinants (grey) was expressed as relative change. With comparison to *ACT1*, one more copy of each transporter gene was determined in corresponding recombinants.

In order to estimate copy number of each expression cassette that integrated in genome of recombinant strains, quantitative-PCR (qPCR) analysis was carried out. *ACT1* was utilized as an endogenous control^26^. With comparison to *ACT1*, no obvious relative changes of these transporters were observed (Fig. 6). This indicated that these transporter genes were presented in a single copy in parental strain, respectively. After individual transportation of each endogenous transporters gene into cells of parental strain, relative changes of 2.54 ± 0.22-fold for YALI0B19470g, 2.33 ± 0.27-fold for YALI0C15488g, 2.62 ± 0.33-fold for YALI0C21406g, 2.28 ± 0.19-fold for YALI0D24607g, 2.39 ± 0.26-fold for YALI0D20108g and 2.55 ± 0.32-fold for YALI0E32901g were observed, which indicated one more copy of each transporter gene existed per genome in corresponding recombinant strain (Fig. 6).

### The effects of transporter genes on carboxylate accumulation in *Y*. *lipolytica*

**Figure 7:**
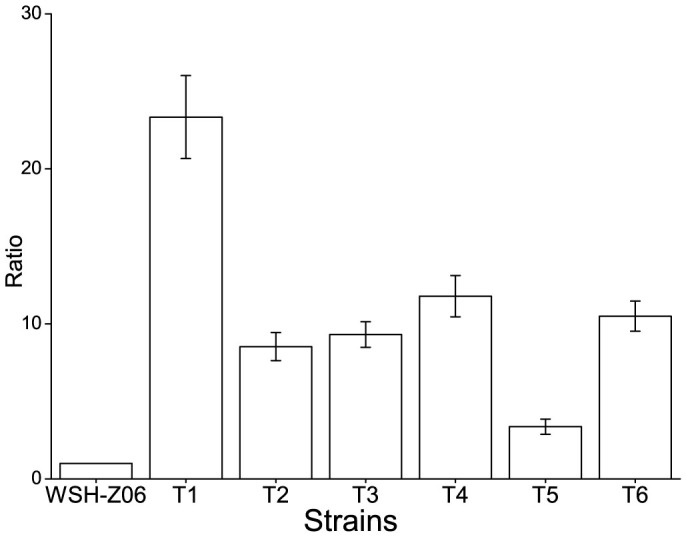
Verification of transcription of putative carboxylate transporters in *Y*. *lipolytica* WSH-Z06. RNA extracted from glucose-grown cells of the T1, T2, T3, T4, T5, and T6 strains was subjected to qRT-PCR. The strains T1-T6 were mutant variants in which plasmid p0(hph)-470, p0(hph)-488, p0(hph)-406 p0(hph)-607, p0(hph)-108 and p0(hph)-901 were individually expressed in the wild-type strain. The transcription of YALI0B19470g, YALI0C15488g, YALI0C21406g, YALI0D24607g, YALI0D20108g, and YALI0E32901g increased by 23.34 ± 2.67-fold, 8.53 ± 0.90-fold, 9.32 ± 0.82-fold, 11.79 ± 1.32-fold, 3.37 ± 0.49-fold and 10.50 ± 0.97-fold, respectively.

**Figure 8:**
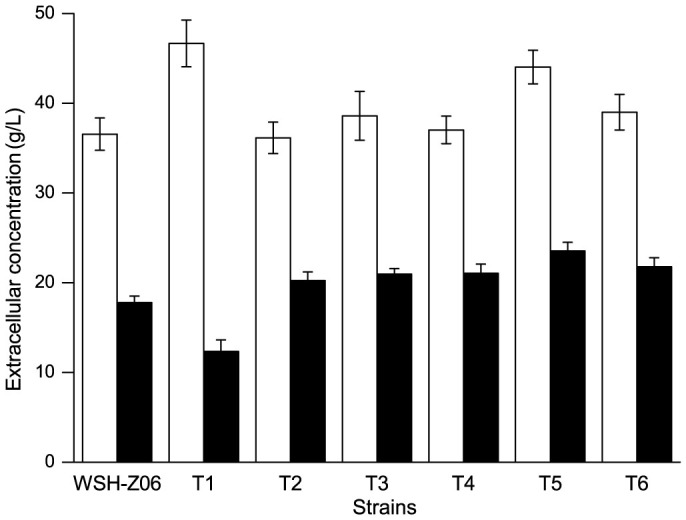
Effects of potential transporters on keto acids accumulation. White: α-KG; black: PA. The strains T1–T6 were mutant variants in which plasmid p0(hph)-470, p0(hph)-488, p0(hph)-406 p0(hph)-607, p0(hph)-108 and p0(hph)-901 were individually expressed in the wild-type strain. The transport of PA was enhanced in all mutants except the T1 strain. Extracellular PA concentration increased from 17.77 ± 0.72 g·L^−1^ to 20.22 ± 0.98, 20.95 ± 0.62, 21.06 ± 1.03, 23.53 ± 0.98, and 21.79 ± 1.0 0 g·L^−1^ in cells of the T2, T3, T4, T5, and T6 strains, respectively, and decreased by 30.6% to the concentration of 12.32 ± 1.31 g·L^−1^ for T1 strain. The content of α-KG varied slightly in strain T2, T3, T4 and T6, while the content of the dicarboxylate increased to 46.66 ± 2.59- and 44.02 ± 1.87 g·L^−1^ in strain T1 and T5.

As previously reported carboxylate transporter possess bi-functions for carboxylate influx and efflux^25^. To clarify the roles of these transporters on carboxylate extracellular accumulation, they were overexpressed in *Y*. *lipolytica* WSH-Z06. The *hp4d* promoter was used to increase the transcription of these genes, and YALI0B19470g, YALI0C15488g, YALI0C21406g, YALI0D24607g, YALI0D20108g and YALI0E32901g expression was improved by 23.34 ± 2.67-fold, 8.53 ± 0.90-fold, 9.32 ± 0.82-fold, 11.79 ± 1.32-fold, 3.37 ± 0.49-fold and 10.50 ± 0.97-fold, respectively (Fig. 7). Due to the flexible substrate specificity of these transporters, the accumulation of PA and α-KG increased in T5 cells, and the ratio of extracellular α-KG/PA decreased from 2.06 to 1.87 compared to the wild-type strain. This ratio also decreased in YALI0C15488g, YALI0C21406g, YALI0D24607g, and YALI0E32901g overexpressing strains, as only extracellular PA increased for T2, T3, T4, and T6. A competitive dual effect was observed for strain T1: the transport of α-KG increased dramatically, whereas the concentration of PA dropped by 30.6%, resulting in an increase in the ratio of extracellular α-KG/PA from 2.06 to 3.79 (Fig. 8).

The intracellular carboxylate content, C_in_ (μmol·(mg·DCW)^−1^), was also determined (Table 2). The intracellular accumulation of α-KG decreased from 0.026 ± 0.005 μmol·(mg·DCW)^−1^ to 0.014 ± 0.002, 0.023 ± 0.003, 0.014 ± 0.002 and 0.020 ± 0.002 μmol·(mg·DCW)^−1^ for strains T1, T4, T5 and T6. The intracellular accumulation of PA decreased from 0.034 ± 0.006 μmol·(mg·DCW)^−1^ to 0.009 ± 0.002, 0.029 ± 0.003, 0.017 ± 0.002 and 0.025 ± 0.002 μmol·(mg·DCW)^−1^ in strains T1, T4, T5 and T6 respectively. Compared to the wild-type strain, the growth yield decreased from 11.17 ± 1.08 g·L^−1^ to 8.45 ± 0.72, 8.27 ± 0.89, 8.34 ± 0.76, 8.47 ± 0.61, 8.36 ± 0.88 and 7.40 ± 0.71 g·L^−1^ for strains T1, T2, T3, T4, T5 and T6, respectively, suggesting that enhanced carboxylate transportation caused the shift in carbon flux from cellular growth to carboxylate accumulation. The combination of increased carboxylate accumulation with decreased cell growth lead to a sharp increase in the yield of α-KG and PA, Y_α-KG/DCW_ and Y_PA/DCW_. Finally, the ratio of extracellular carboxylate to intracellular carboxylate increased, and maximum values of 2399.71 ± 241.63 for α-KG and 1685.20 ± 208.56 for PA were observed for the T1 strain. Based on these observations, overexpression of YALI0A9470g was considered the best strategy to enhance α-KG transport and reduce PA accumulation.

## Discussion

The object of the current work was to screen and identify carboxylate transporters, then determine whether the identified proteins regulate accumulation of non-target carboxylates. The duplication of transporters and the flexible substrate specificity demonstrated by the identified carboxylate transporters facilitated extracellular accumulation of carboxylates. Moreover, knowledge of the examined carboxylate transport mechanism is a prerequisite for improving carboxylate synthesis through metabolic engineering. The results provide new insights for regulating extracellular carboxylate accumulation in similar eukaryotic microorganisms.

To circumvent the issue of PA accumulation, previous studies have focused mainly on the regulation of intrinsic forces that redistribute carbon flux from other intermediates to α-KG production. Expression of PA carboxylase^10^, malate dehydrogenase, and fumarase^3^ dramatically decreased the accumulation of PA. A strategy to regulate co-factor regeneration resulted in remarkable reduction of extracellular PA^15^. However, as PA has a pivotal role in the regulation of carbon metabolism^20^, these modifications could not entirely overcome PA accumulation. As accumulation of carboxylate is believed to be a yeast defense response to severe environmental conditions^27^, regulation of carboxylate transportation process might be another potent route for enhancement of α-KG production.

The carboxylate transport process of yeast is an intensively investigated field^20^. The *S*. *cerevisiae ScJEN1* and *ScADY2* genes were identified as key carboxylate transporters^28^,^29^. The duplication of transporters has been strongly implicated in the utilization of organic acids as a carbon source^24^. In *Kluyveromyces* *lactis*, the presence of two carboxylate transporters, Jen1p and Jen2p, guaranteed efficient uptake of lactic acid as a substrate from a lactic-acid-producing habitat^30^. As the cells of *Y*. *lipolytica* harbored a powerful potential to use a wide range of substrates as a sources of carbon and energy^31^, these results indicated that powerful carboxylate transporters that maintain intracellular environment homeostasis. A previous study reported that reduction of by-product resulted in enhanced synthesis of target carboxylate by de-repression of the feedback inhibition^3^. It was speculated that the enhanced synthesis of α-KG could be achieved through de-repression of the feedback inhibition by efflux of intermediates.

In *Y*. *lipolytica*, efficient carboxylate transport was achieved by the duplication of *iso*-functional transporters. Evolution analysis and motif identification confirmed that a precursor form of Jen1p, preJen1p, arose from the duplication of an ancestral Jen2p^32^. In *S*. *cerevisiae*, the transport capacity and substrate affinity of Jen1p were determined by the conserved NXXS/THXS/TQDXXXT sequence^33^. Presence of this signature sequence also determined the flexibility of substrate specificity for these transporters. Previously, Jen1p was induced by lactate, PA, and propionate, whereas Ady2p and Jen2p were induced by acetate^29^,^30^. Expression of transporters from *Y*. *lipolytica* displayed different responses to exogenous carbon source, and single carboxylate induced multiple transporters.

The roles of these carboxylate transporters were assayed via double their copy numbers in the genomic DNA of α-KG producer. One more copy of each endogenous carboxylate transporter was observed (Fig. 6). Our observation that overexpression of carboxylate transporters resulted in enhanced accumulation of extracellular carboxylates (Fig. 7). Previously, uncovered the mechanism for malate efflux was mediated by monoanionic malate concentration gradient, in which the proton symport was major force^25^. Similar to the observations that production of carboxylate was benefited from overexpression of carboxylate transporter^13^,^34^, the efflux of carboxylate accompanied by symport of proton was speculated^25^. Based on measurement of total content of mixture of monoanionic, dianionic and undissociated form of the carboxylates, the intracellular content of carboxylates was not correlated with extracellular carboxylate content. While, the reported carboxylate transporter was specific for monoanionic form of carboxylate for efflux, the efflux specificity of this series of transporter would be the key to the contradictory observation in future studies.

## Methods

### Strains and plasmids

All strains used in this study were summarized in Table 3. *Y*. *lipolytica* WSH-Z06, an α-KG producing wild-type strain, was previously screened by our lab^35^. The plasmid p0(hph) was previously constructed based on plasmid p0. The p0 could integrate a single copy of exogenous sequence via recombinant at the locus of *XRP2* in the genome of *Y. lipolytica*^10^. The hygromycin phosphotransferase encoded by *hph* was amplified from plasmid pUB4-CRE and was used to replace the *URA3* sequence in plasmid p0^15^,^36^. Plasmids were propagated in *Escherichia*. *coli* JM109. *S*. *cerevisiae* CEN.PK2-1D (MATα, Δ*ura3-52*; Δ*trp1-289*; Δ*leu2-3*,*112*; Δ*his3*-1; *MAL2*-8^C^; *SUC2*) from EUROSARF (Frankfurt, Germany) and the pY13-TEF1 plasmid was used for heterologous expression of potential transporters.

### Media and culture conditions

*E*. *coli* was cultured in Luria broth (LB) medium (5 g·L^−1^ yeast extract, 10 g·L^−1^ peptone, 10 g·L^−1^ NaCl) supplemented 100 mg·L^−1^ ampicillin when necessary. Yeast strains were cultured in YPD medium (10 g·L^−1^yeast extract, 20 g·L^−1^ peptone, and 20 g·L^−1^ dextrose) or YNB medium (20 g·L^−1^ glucose, 1.7 g·L^−1^ yeast nitrogen base without amino acids, 5 g·L^−1^ (NH_4_)_2_SO_4_) supplemented with 50 μg·mL^−1^ uracil, leucine, tryptophan and histidine when necessary. The following media were used for carboxylate treatment: YPA (1.7 g·L^−1^ yeast nitrogen base without amino acids, 5 g·L^−1^ (NH_4_)_2_SO_4_, 50 g·L^−1^ acetate, pH 7.0), YPL (1.7 g·L^−1^ yeast nitrogen base without amino acids, 5 g·L^−1^ (NH_4_)_2_SO_4_, 50 g·L^−1^ lactate, pH 7.0), YPP (1.7 g·L^−1^ yeast nitrogen base without amino acids, 5 g·L^−1^ (NH_4_)_2_SO_4_, and 50 g·L^−1^ PA, pH 7.0), YPM (1.7 g·L^−1^ yeast nitrogen base without amino acids, 5 g·L^−1^ (NH_4_)_2_SO_4_, 50 g·L^−1^ malate, pH 7.0), YPK (10 g·L^−1^ yeast extract without amino acids, 5 g·L^−1^ (NH_4_)_2_SO_4_, and 100 g·L^−1^ α-KG, pH 7.0), YPC (1.7 g·L^−1^ yeast nitrogen base without amino acids, 5 g·L^−1^ (NH_4_)_2_SO_4_, 50 g·L^−1^ citrate, pH 7.0). The seed-culture media (dextrose, 20 g·L^−1^; peptone, 10 g·L^−1^; KH_2_PO_4_, 1 g·L^−1^; and MgSO_4_·7H_2_O, 0.5 g·L^−1^, pH 5.5) and fermentation media (glycerol, 100 g·L^−1^; (NH_4_)_2_SO_4_, 3 g·L^−1^; KH_2_PO_4_, 3 g·L^−1^; MgSO_4_·7H_2_O, 1.2 g·L^−1^; NaCl, 0.5 g·L^−1^; K_2_HPO_4_, 0.1 g·L^−1^; and thiamine-HCl 4 × 10^−7^ g·L^−1^, pH 4.5) were described previously^37^.

### Bioinformatic analysis and genome-wide prediction of transmembrane proteins

The 6611 putative proteins encoded by *Y*. *lipolytica* CLIB122 genomic DNA were obtained from UniProt (http://www.uniprot.org/). A genome-wide analysis using a transmembrane-helix sequence was performed following a method described previously^38^,^39^. For each protein, the transmembrane protein topology was predicted using TMHMM (http://www.cbs.dtu.dk/services/TMHMM/). The predicted result was visualized using the TMRPres2D tool^40^. The following values were used to discriminate helical proteins from other proteins^41^: (i) the number of predicted transmembrane helices; (ii) the expected number of residues in the transmembrane helices; and (iii) the expected number of transmembrane helices. To avoid false prediction, we also analyzed all proteins with putative transmembrane helices at the N terminus with SignalP (http://www.cbs.dtu.dk/services/SignalP/) to predict whether the sequence encoded a signal peptide^42^. All screened transmembrane proteins were used for sequence-similarity searches using BLAST (http://blast.ncbi.nlm.nih.gov/) to identify orthologs that have been characterized as carboxylate transporters.

### Keto acid treatment

The cells of *Y*. *lipolytica* WSH-Z06 were streaked onto YPD slants from glycerol stocks. Cells grown in glucose were harvested at the exponential phase. After a 2 h treatment in saline water, cells were transferred to 100 mL YPK or 100 mL YPP and incubated at 28°C. PA- or α-KG-treated cells were collected at regular time intervals for quantitative real-time PCR (qRT-PCR) analysis and determination of intracellular carboxylate content.

YPA, YPL, YPP, YPM, YPK, and YPC media, supplemented as necessary with 50 μg·mL^−1^ uracil, leucine, tryptophan, and histidine, were used to assay carboxylate transportation of *S*. *cerevisiae* CEN.PK2-1D cells and derivatives.

Yeast cells were harvested by centrifugation at 10,000 × *g* for 10 min, and washed twice with cold distilled water. The dry cell weight was determined according to protocol described previously^37^. The intracellular carboxylate content was determined according to the method previously, with little modification^43^. Harvested cells were stored in liquid nitrogen until extraction of intracellular organic acid which was followed by cell disruption of according to a protocol described previously^44^. Supernatants were used for determination of intracellular carboxylate concentrations by high-performance liquid chromatography (HPLC). The intracellular concentration of carboxylate was expressed in μmol·(mg·DCW)^−1^.

### Quantitative real-time PCR analysis

The cells of *Y*. *lipolytica* WSH-Z06 were harvested, centrifuged at 10,000 × *g* for 10 min, and immediately frozen in liquid nitrogen until RNA extraction. Total RNA was extracted using Trizol reagent (Life Technologies, Carlsbad, CA), according to the manufacturer's instructions. cDNA was synthesized from 5 μg total RNA using the PrimeScript RT Reagent Kit Perfect Real Time (Takara, Dalian, China). qRT-PCR was performed with the synthesized cDNA and primers listed in Table 4 using the SYBR Premix Ex *Taq*^TM^ Kit (Taraka, Dalian, China) and a LightCycler 480 II Real-time PCR instrument (Roche Applied Science, Mannheim, Germany). All experiments were performed in triplicate and mean values were used for further calculations. Fold changes were determined by the 2^-ΔΔC^_T_ method and normalized to the *ACT1* gene^45^.

### Disruption of the *ScJEN1* and *ScADY2* genes in *S*. *cerevisiae*

To disrupt the *S*. *cerevisiae* CEN.PK2-1D *JEN1* and *ADY2* genes, two disruption cassettes were constructed using a protocol described previously^46^. The *loxP*-*URA3*-*loxP* and *loxP*-*LEU2*-*loxP* modules were amplified from plasmids pUG72 and pUG73, respectively, with oligonucleotides JEN-MF/JEN-MR, ADY-MF/ADY-MR, respectively (Table 5). Two fragments of *ScJEN1* were amplified from *S*. *cerevisiae* genomic DNA and were subsequently flanked by the *loxP*-*URA3*-*loxP* module to generate the *JEN1* disruption cassette. *ScADY2* fragments, PCR-amplified from *S*. *cerevisiae* genomic DNA, were flanked with the *loxP*-*LEU2*-*loxP* module to generate the *ADY2* disruption cassette.

The disruption cassettes were introduced into *S*. *cerevisiae* CEN.PK2-1D with a previously described protocol^46^, and the resulting line, *S*. *cerevisiae* CEN.PK2-1D Δ*jen1*, is referred to as W1. The *ADY2* disruption cassette was introduced into this W1 strain, and the resulting double deletion strain is referred to as W2.

### Heterologous expression of putative transporter genes in *S*. *cerevisiae* W2

Six putative transporter genes, YALI0B19470g, YALI0C15488g, YALI0C21406g, YALI0D24607g, YALI0D20108g, and YALI0E32901g, were amplified from *Y*. *lipolytica* WSH-Z06 genomic DNA with the primers listed in Table 6. The amplified fragments were introduced into *Bam*HI–*Eco*RI sites of the pY13-TEF1 expression vector, resulting in the pY13-TEF1-470, pY13-TEF1-488, pY13-TEF1-406, pY13-TEF1-607, pY13-TEF1-108, and pY13-TEF1-901 plasmids, respectively. These vectors were introduced into *S*. *cerevisiae* W2 using a previously described protocol, and the resulting lines are referred to as W3, W4, W5, W6, W7, and W8, respectively.

### Overexpression of putative transporter genes in *Y*. *lipolytica*

The YALI0B19470g, YALI0C15488g, YALI0C21406g, YALI0D24607g, YALI0D20108g, and YALI0E32901g open reading frames were PCR-amplified from *Y*. *lipolytica* WSH-Z06 genomic DNA with the oligonucleotides listed in Table 7. The amplified fragments were digested with *Eco* RI and *Bam* HI or *Not* I and were subsequently inserted into the integrative expression vector p0(hph), resulting in p0(hph)-470, p0(hph)-488, p0(hph)-406, p0(hph)-607, p0(hph)-108, and p0(hph)-901, respectively. These vectors were digested with *Avr*II, purified, and transformed into *Y. lipolytica* WSH-Z06 using a previously described protocol^47^. The resulting transformants, *Y*. *lipolytica* T1, T2, T3, T4, T5, and T6, respectively, were screened on YPD plates containing 400 mg·L^−1^ hygromycin B and verified with the oligonucleotides listed in Table 7.

### Copy number analysis

In order to determine the copy number of the integrative expression cassettes in the recombinant strains, a qPCR analysis was performed on the genomic DNA template using *ACT1* as the internal control^26^. Genomic DNA from parental strain (WSH-Z06) and six recombinant strains (T1, T2, T3, T4, T5 and T6) were isolated after disruption of yeast cells with glass beads (Sigma-Aldrich, St.Louis, MI) by FastPrep 24 (MP Biomedicals, Santa Ana, CA). qPCR was performed with the 5 ng genomic DNA and primers listed in Table 4 using the SYBR Premix Ex *Taq*^TM^ Kit (Taraka, Dalian, China) and a LightCycler 480 II Real-time PCR instrument (Roche Applied Science, Mannheim, Germany). All experiments were performed in triplicate and mean values were used for further calculations. Fold changes were determined by the 2^−ΔΔC^_T_ method and normalized to the *ACT1* gene^45^.

### Shake flask culture

Shake flask culture was performed in 500 mL flasks containing 50 mL fermentation medium following the protocol stated previously^35^. A yeast seed culture was inoculated from an agar slant and incubated in a 500 mL flask containing 50 mL medium for 18 h on a rotary shaker at 28°C. The culture was used to inoculate 500 mL flasks containing 50 mL fermentation medium. An inoculum volume of 10% (v/v) was used for α-KG accumulation assay. Flask cultures were incubated in a shaker at 200 r·min^−1^ for 144 h at 28°C.

### HPLC analysis

Samples taken from shake flask culture were centrifuged at 10,000 × *g* for 10 min. The supernatant was diluted 50 times and filtered through a membrane (pore size = 0.22 μm). α-KG, pyruvic acid, acetate, lactate, malate, and citrate present in the supernatant were simultaneously determined by HPLC (Agilent 1200 series, Santa Clara, CA, USA) with an Aminex HPX-87H column (300 mm × 7.8 mm; Bio-Rad Laboratories Inc., Hercules, CA, USA). The mobile phase was 5 mmol L^−1^ sulfuric acid in distilled, de-ionized water filtered to 0.22 μm. The mobile phase flow rate was 0.6 mL min^−1^. The column temperature was maintained at 35°C, and the injection volume was 10 μL. α-KG, pyruvic acid, acetate, lactate, malate, and citrate were detected with a UV detector (wavelength at 210 nm)^37^. To determine the intracellular carboxylates, cells taken from shake flask culture were disrupted and lysates were centrifuged at 10,000 × *g* for 10 min. The carboxylate content in supernatant was determined by HPLC followed the protocol above.

## Author Contributions

G.H.W. and Z.J.W. designed the experiments and wrote the paper. G.H.W. and L.P.R. performed the experiments. G.C.D. and J.C. conceived the project, analyzed the data and wrote the paper. C.M. analyzed the data.

## Supplementary Material

Supplementary Information

## Figures and Tables

**Table 1:** Intracellular carboxylate accumulation in *S*. *cerevisiae* CEN.PK2-1D and derivatives

Strain	Genotype	Acetate	Lactate	PA	Malate	α-KG	Citrate
WT	*S*. *cerevisiae*, CEN.PK2-1D	/*	2.22 ± 0.21	0.94 ± 0.12	3.14 ± 0.24	1.29 ± 0.12	/
W1	*S*. *cerevisiae*, CEN.PK2-1D Δ*jen1*	/	/	/	0.05 ± 0.01	0.86 ± 0.08	/
W2	*S*. *cerevisiae*, CEN.PK2-1D Δ*jen1*Δ*ady2*	/	/	/	/	/	/
W3	*S*. *cerevisiae* CEN.PK2-1D, Δ*jen1*Δ*ady2*(pY13-TEF1-470)	/	/	/	0.15 ± 0.01	0.09 ± 0.01	0.04 ± 0.01
W4	*S*. *cerevisiae* CEN.PK2-1D, Δ*jen1*Δ*ady2*(pY13-TEF1-488)	0.81 ± 0.07	/	0.17 ± 0.03	0.11 ± 0.01	1.41 ± 0.19	0.08 ± 0.01
W5	*S*. *cerevisiae* CEN.PK2-1D, Δ*jen1*Δ*ady2*(pY13-TEF1-406)	/	0.53 ± 0.04	/	0.09 ± 0.01	0.77 ± 0.06	0.04 ± 0.01
W6	*S*. *cerevisiae* CEN.PK2-1D, Δ*jen1*Δ*ady2*(pY13-TEF1-607)	/	/	/	0.09 ± 0.01	0.56 ± 0.06	0.05 ± 0.01
W7	*S*. *cerevisiae* CEN.PK2-1D, Δ*jen1*Δ*ady2*(pY13-TEF1-108)	/	0.67 ± 0.09	/	/	0.38 ± 0.04	0.09 ± 0.01
W8	*S*. *cerevisiae* CEN.PK2-1D, Δ*jen1*Δ*ady2*(pY13-TEF1-901)	0.66 ± 0.04	/	/	0.11 ± 0.01	0.39 ± 0.03	0.03 ± 0.01

*: Carboxylate content less than 0.005 μmol·(mg·DCW)^−1^ or undetected. The intracellular carboxylate content was measured during substrate specificity tests and presented as μmol·(mg·DCW)^−1^.

**Table 2:** The effects of transporter overexpression on carboxylate production in *Y*. *lipolytica* and derivatives

		C_in_(μmol·(mg·DCW)^−1^)				Ratio_ex/in_	
Strain	DCW(g·L^−1^)	α-KG	PA	Y_α-KG/DCW_(g·g^−1^)	Y_PA/DCW_(g·g^−1^)	α-KG	PA
WSH-Z06	11.17 ± 1.08	0.026 ± 0.005	0.034 ± 0.006	3.28 ± 0.32	0.23 ± 0.03	860.35 ± 78.45	531.45 ± 48.97
T1	8.45 ± 0.72	0.014 ± 0.002	0.009 ± 0.002	4.94 ± 0.52	0.30 ± 0.04	2399.71 ± 241.63	1685.20 ± 208.56
T2	8.27 ± 0.89	0.027 ± 0.003	0.032 ± 0.004	3.90 ± 0.42	0.25 ± 0.03	975.27 ± 109.21	769.61 ± 84.21
T3	8.34 ± 0.76	0.030 ± 0.005	0.033 ± 0.005	4.13 ± 0.44	0.24 ± 0.02	938.78 ± 96.32	779.42 ± 94.38
T4	8.47 ± 0.61	0.023 ± 0.003	0.029 ± 0.003	3.91 ± 0.38	0.31 ± 0.04	1161.29 ± 125.76	865.52 ± 90.76
T5	8.36 ± 0.88	0.013 ± 0.002	0.017 ± 0.002	4.71 ± 0.46	0.59 ± 0.06	2358.20 ± 359.79	1641.53 ± 178/94
T6	7.40 ± 0.71	0.020 ± 0.002	0.025 ± 0.002	5.27 ± 0.61	0.36 ± 0.04	1756.27 ± 193.1	1343.41 ± 147.89

The strains T1–T6 were mutant variants in which plasmid p0(hph)-470, p0(hph)-488, p0(hph)-406 p0(hph)-607, p0(hph)-108 and p0(hph)-901 were individually expressed in the wild-type strain. Ratio_ex/in_ is the mass ratio of extracellular carboxylate to intracellular carboxylate. The yield of Y_α-KG/DCW_ and Y_PA/DCW_ is the mass ratio of α-KG to DCW and PA to DCW, respectively.

**Table 3:** Strains used in this study

Strain	Genotype and remarks
*S*. *cerevisiae* CEN.PK2-1D	MATα Δ*ura3-52*; Δ*trp1-289*; Δ*leu2-3*,*112*; Δ*his3*-1; *MAL2*-8^C^; *SUC2*
W1	MATα Δ*ura3-52*; Δ*trp1-289*; Δ*leu2-3*,*112*; Δ*his3*-1; *MAL2*-8^C^; *SUC2*; Δ*jen1*::*URA3*
W2	MATα Δ*ura3-52*; Δ*trp1-289*; Δ*leu2-3*,*112*; Δ*his3*-1; *MAL2*-8^C^; *SUC2*; Δ*jen1*::*URA3*; Δ*ady2*::*LEU2*
W3	MATα Δ*ura3-52*; Δ*trp1-289*; Δ*leu2-3*,*112*; Δ*his3*-1; *MAL2*-8^C^; *SUC2*; Δ*jen1*::*URA3*; Δ*ady2*::*LEU2*(pY13-TEF1-470)
W4	MATα Δ*ura3-52*; Δ*trp1-289*; Δ*leu2-3*,*112*; Δ*his3*-1; *MAL2*-8^C^; *SUC2*; Δ*jen1*::*URA3*; Δ*ady2*::*LEU2*(pY13-TEF1-488)
W5	MATα Δ*ura3-52*; Δ*trp1-289*; Δ*leu2-3*,*112*; Δ*his3*-1; *MAL2*-8^C^; *SUC2*; Δ*jen1*::*URA3*; Δ*ady2*::*LEU2*(pY13-TEF1-406)
W6	MATα Δ*ura3-52*; Δ*trp1-289*; Δ*leu2-3*,*112*; Δ*his3*-1; *MAL2*-8^C^; *SUC2*; Δ*jen1*::*URA3*; Δ*ady2*::*LEU2*(pY13-TEF1-607)
W7	MATα Δ*ura3-52*; Δ*trp1-289*; Δ*leu2-3*,*112*; Δ*his3*-1; *MAL2*-8^C^; *SUC2*; Δ*jen1*::*URA3*; Δ*ady2*::*LEU2*(pY13-TEF1-108)
W8	MATα Δ*ura3-52*; Δ*trp1-289*; Δ*leu2-3*,*112*; Δ*his3*-1; *MAL2*-8^C^; *SUC2*; Δ*jen1*::*URA3*; Δ*ady2*::*LEU2*(pY13-TEF1-901)
*Y*. *lipolytica* WSH-Z06	Wild-type
T1	Wild-type(p0(hph)-470)
T2	Wild-type(p0(hph)-488)
T3	Wild-type(p0(hph)-406)
T4	Wild-type(p0(hph)-607)
T5	wild-type(p0(hph)-108)
T6	Wild-type(p0(hph)-901)

**Table 4:** Oligonucleotide primers used for qRT-PCR

Gene	Primers	Sequence (5'-3')	Product size (bp)
YALI0B19470g	YALIOB19470-F	CAACAAGGAAGACAACAG	153
	YALIOB19470-R	AGGTAGGTGAACATAAGC	
YALI0C15488g	YALIOC15488-F	GCAACCATCTCAGCCATTC	199
	YALIOC15488-R	GTAACCTCGCATCTTCAGC	
YALI0C21406g	YALIOC21406-F	GCAGACCTACCAGCAGTTC	171
	YALIOC21406-R	ACGACACAGAGCAAGTATCC	
YALI0D20108g	YALIOD20108-F	TGCTACAGGAAGGCTATGC	135
	YALIOD20108-R	GGAAGATGATGATGAGAACAGG	
YALI0D24607g	YALIOD24607-F	CTGCTTGTAGGTGGTGAC	104
	YALIOD24607-R	GAGTGCTGAGTGATAAATACG	
YALI0E32901g	YALIOE32901-F	TCTATGATTACGGTAAGGTTATG	188
	YALIOE32901-R	GACTCGCTCAAGGTTCTC	
*ACT1*	ACT1-F	AAGTCCAACCGAGAGAAGATG	132
	ACT1-R	ACCAGAGTCAAGAACGATACC	

**Table 5:** Oligonucleotides used for gene disruption in *S*. *cerevisiae*

Primers	Sequence(5'-3')	Description
JEN-L-F	GGATCCATGTCGTCGTCAATTACAGATG	*Bam*HI
JEN-L-R	ATGTGCAGTAAGGACGTAAATC	
JEN-R-F	AAAGGCTATATTAGGTGCCG	
JEN-R-R	GAATTCTTGTTCAACAATGTCACTAATCG	*Eco*RI
JEN-M-F	GATTTACGTCCTTACTGCACATGTGAAAACCTCTGACACATGC	
JEN-M-R	CGGCACCTAATATAGCCTTTGCCTTTGAGTGAGCTGATACC	
ADY-L-F	GGATCCATGTCTGACAAGGAACAAACGAG	*Bam*HI
ADY-L-R	CCACCATAAAACATAGCACAACC	
ADY-R-F	GATTGCTGGTATTTGGGAGATAG	
ADY-R-R	CCTAGGCCCTTTCAGTAGATGGTAATGGG	*Avr*II
ADY-M-F	GGTTGTGCTATGTTTTATGGTGGGCATAGGCCACTAGTGGATCTG	
ADY-M-R	CTATCTCCCAAATACCAGCAATCCAGCTGAAGCTTCGTACGC	

**Table 6:** Oligonucleotides used for gene expression in *S. cerevisiae*

Primers	Sequence(5'-3')	Description
YALI0D20108g-F2	CGGGATCCATGAATTTTGACAACTTCCCAGC	*Bam*HI
YALI0D20108g-R2	GGAATTCTTATCGAGTATCGCTCGAAGAAC	*Eco*RI
YALI0C21406g-F2	GGACTAGTATGGATCTCGACAACTACCCTCC	*Bam*HI
YALI0C21406g-R2	GGAATTCTCACTTTTGGGATCCGGGG	*Eco*RI
YALI0B19470g-F2	CGGGATCCATGCCCATCACAGTTTCACAAG	*Bam*HI
YALI0B19470g-R2	GGAATTCTTAACGAGTGAGATTGGTGTCG	*Eco*RI
YALI0C15488g-F2	CGGGATCCATGGATTTGGACAACCTCCC	*Bam*HI
YALI0C15488g-R2	GGAATTCCTACTTAGTAGCATTGGTGTCAACTC	*Eco*RI
YALI0D24607g-F2	CGGGATCCATGACCCAGTCGTACGAAGTC	*Bam*HI
YALI0D24607g-R2	GGAATTCCTAATGAACACTTCCAACAGTGG	*Eco*RI
YALI0E32901g-F2	CGGGATCCATGGAAGCTCCTAATCTCTCG	*Bam*HI
YALI0E32901g-R2	GGAATTCTACTTGGACTCGTAGGGGGA	*Eco*RI

**Table 7:** Oligonucleotides used for gene expression in *Y*. *lipolytica*

Primers	Sequence (5'-3')	Description
YALI0D20108g-F1	CGGGATCCATGAATTTTGACAACTTCCCAG	*Bam*HI
YALI0D20108g-R1	GGAATTCTTATCGAGTATCGCTCGAAGAAC	*Eco*RI
YALI0C21406g-F1	GGAATTCATGGATCTCGACAACTACCCTC	*Eco*RI
YALI0C21406g-R1	TTGCGGCCGCTCACTTTTGGGATCCGGG	*Not*I
YALI0B19470g-F1	CGGGATCCATGCCCATCACAGTTTCACAAG	*Bam*HI
YALI0B19470g-R1	GGAATTCTTAACGAGTGAGATTGGTGTCG	*Eco*RI
YALI0C15488g-F1	CGGGATCCATGGATTTGGACAACCTCCC	*Bam*HI
YALI0C15488g-R1	GGAATTCCTACTTAGTAGCATTGGTGTCAACTC	*Eco*RI
YALI0D24607g-F1	CGGGATCCATGACCCAGTCGTACGAAGTC	*Bam*HI
YALI0D24607g-R1	GGAATTCCTAATGAACACTTCCAACAGTGG	*Eoc*RI
YALI0E32901g-F1	CGGGATCCATGGAAGCTCCTAATCTCTCGC	*Bam*HI
YALI0E32901g-R1	GGAATTCTTACTTGGACTCGTAGGGGGA	*Eco*RI
VB-F	CGTTTGCCAGCCACAGATT	
V-YALI0D20108g-R	GCGTTTGCCAGCCACAGAT	
V-YALI0C21406g-R	GTAGATGCAGGCAGCACCG	
V-YALI0B19470g-R	AAGACAGAGGCGTTGATACCG	
V-YALI0C15488g-R	TGCGAGGTTACCAAGCTGAT	
V-YALI0D24607g-R	GACAAACGCCCAGGGATAG	
V-YALI0E32901g-R	TGTCCATCTGCTTGCCCTC	

## Notes

Sequence of six transporter genes, YALI0B19470g, YALI0C15488g, YALI0C21406g, YALI0D24607g, YALI0D20108g and YALI0E32901g, can be acquired in NCBI through GenBank (XP_503058.1, XP_503239.1, XP_504706.1, XP_501098.1, XP_501871.1 and XP_502090.1).
