# Supplementary material for: Identification and application of keto acids transporters in Yarrowia lipolytica
Source: Sci Rep. 2015 Jan 30;5:8138. doi: 10.1038/srep08138 (PMC4311248; doi:10.1038/srep08138)
Supplement: Supplementary Information [file srep08138-s1.doc]

**Identification and application of keto acids transporters in *Yarrowia lipolytica***

Hongwei Guo^1,3^, Peiran Liu^1^, Catherine Madzak^2^, Guocheng Du^1,3^, Jingwen Zhou^1,3*^, Jian Chen^1,3*^

^1^ School of Biotechnology and Key Laboratory of Industrial Biotechnology, Ministry of Education, Jiangnan University, 1800 Lihu Road, Wuxi, Jiangsu 214122, China;

^2^ UMR1238 Microbiologie et Génétique Moléculaire, INRA/CNRS/AgroPan’s Tech, CBAI, BP 01, 78850 Thiverval-Grignon, France;

^3^ Synergetic Innovation Center of Food Safety and Nutrition, 1800 Lihu Road, Wuxi, Jiangsu 214122, China.

^*^ Corresponding authors.

Jingwen Zhou, Jian Chen

Mailing address: School of Biotechnology, Jiangnan University, 1800 Lihu Road, Wuxi, Jiangsu 214122, China.

Phone: +86-0510-85918312, Fax: +86-0510-85918309

E-mail: zhoujw1982@jiangnan.edu.cn, jchen@jiangnan.edu.cn

>sp|P25613|ADY2_YEAST Accumulation of dyads protein 2 OS=Saccharomyces cerevisiae (strain ATCC 204508 / S288c) GN=ADY2 PE=1 SV=1

MSDKEQTSGNTDLENAPAGYYSSHDNDVNGVAEDERPSHDSLGKIYTGGDNNEYIYIGRQKFLKSDLYQAFGGTLNPGLAPAPVHKFANPAPLGLSAFALTTFVLSMFNARAQGITVPNVVVGCAMFYGGLVQLIAGIWEIALENTFGGTALCSYGGFWLSFAAIYIPWFGILEAYEDNESDLNNALGFYLLGWAIFTFGLTVCTMKSTVMFFLLFFLLALTFLLLSIGHFANRLGVTRAGGVLGVVVAFIAWYNAYAGVATKQNSYVLARPFPLPSTERVIF

>sp|Q6C0R5|AIM11_YARLI Altered inheritance of mitochondria protein 11 OS=Yarrowia lipolytica (strain CLIB 122 / E 150) GN=AIM11 PE=3 SV=1

MKFRFGEGAEGTLNTSVTTAMIDQEKRAADLKRRKNQMLLFGGATLATLASCRLTARGISSRRYIPKMFQANHMPPQSDMVKEAAMAVVFATTMALSSFSMVVFGVAWSQDVTSLKQFALKMKTKLGAQQIEDEIRNAPMTPETQDLQDQLAGALKKD

>sp|Q6C188|AIM14_YARLI Probable metalloreductase AIM14 OS=Yarrowia lipolytica (strain CLIB 122 / E 150) GN=AIM14 PE=3 SV=1

MLEKRHGDHHNANIKYGTFVLVISLLVVCYIAVRNLTQPEVRARTKRDLRLPLWLSVLLWTALVIGMGVIHVEELNEAAKRFGRLCYALLPLIVFLAIRPSPLPRTFYLKLLPLHKWLGRLATLVGVVHGVLYTVHFVKKNEFYKVFKFDNFLGVIILAVFLVMVVTSLPFFRKRMYSLFYTIHYLSAWFVAIATIFHARPGVGWLFFWVALFMGSSLLYRVLASSTVQIESTEAMGPDLHRVTFPRSILPEFFVPASHIRVSRTLRNPLSWISSTHPYTISSLPSDDHVELIVRPTKFSLAHAASGTQFAVYGPFESLPDDFFSTANRVLVFAGGAGISFALPVVQTLAKAGISHKLVWVLRNKAGVSEVESRLGETPTDIYITGQDPFLGEGVVYAKDAEGTAGLLSEDMEMEEIGQEDEDRERDELDDLLSEDEGSSSQDSTKGAHKNKGKDQDNGKREASQSITYHDGRPQPADTASAYFLDRSAPEGKWILACGPNGLVVTAEQAAKKTGVRFCNETYSM

>sp|Q6CFD4|AIM34_YARLI Altered inheritance of mitochondria protein 34, mitochondrial OS=Yarrowia lipolytica (strain CLIB 122 / E 150) GN=AIM34 PE=3 SV=1

MLRIARTLNRSPLASPLARGIHQSTPKTNPSPLLSSTPTQYSSMKVASLKDECRRRGLRLGGRKADLIERLASHDFSTVSKRAVVSTPNPVAAPAQTATAAGATATLTRVRLITSSAPTLAQGDTSTIDFCKLPHTGMPADAPRIKIPTSPDAYGEVARYGSHAITNDRKVAESVAEDELHSKQPQEIHYASGHVVRSFAGDHSSETGDHDFSTNDKLVLGGIVGAVGFWWFLGLEGKVE

>sp|Q6C520|AKR1_YARLI Palmitoyltransferase AKR1 OS=Yarrowia lipolytica (strain CLIB 122 / E 150) GN=AKR1 PE=3 SV=1

MSTDAELQTISGLSVASKSAPSTQTEGVTASGKVESTTNAEEATSDVEEEENPLVVAARDGNTAEVKRLCESGSYSVLDTAEDGVTALHWAAVNNRISTCQYLVEQGAVVDAKGGQLNGTPLHWACRRGLVYIVHYLIQNGADPLRSDVQGYNALHLATHSSNVMLLVYLLHQGLPVDCQDPNGRTALHWAAYQGDALSVDVLLRWGSDVKITDTQGFLPLHWGIVNGSRNSLARLIEEGSDMYAKSSDGKTPHVMAAEMNTTAQLEGALDDCGRFPDGSQKTKYFDARTTNLLCFFTPFILILLGLVLCTFCGPIFGIILTVATLFGSIKLLKTLVLPSLYNGHAALLKSPFQAGIFTGSAFWVTVKYLTSVLPATFASHPILNFFFASIFGLAMYCFFRCMSMDPGYIPKLSGITEQKEVIETLIERGEFDTRHFCFVTYVRKPLRSKFCRQSKRVVARFDHFCPWVWNAIGVRNHRMFVLYVLFLQIGIPLWLALNSAYFGELLEIKRWDPLEFYLVIWISLQLIWITFLSFVQIFQICRSLTTSEAVNLQKYGYMGADDYSSVPLDHSAATASAKSVMNAHGHAAKSPCFSSVLKLLGVDQFVATAGEAIKHRDNRSWKEKNPTDSGAGTNCFDFWFPNGKFDLLAVFEAGKGGGAIGGHAVDYYKLWDFPDVSPNQQQTNNRSTREDGEALLAESQV

>sp|Q6CEA5|ALG10_YARLI Dol-P-Glc:Glc(2)Man(9)GlcNAc(2)-PP-Dol alpha-1,2-glucosyltransferase OS=Yarrowia lipolytica (strain CLIB 122 / E 150) GN=ALG10 PE=3 SV=1

MLTVPPLIPRLLLALTPLATIAAAFFYFSQTNVLQPTPYIDEIFHIPQTQQYCKGHWNAWDSKITTPPGLYIIGYAWARMLTLTGLSESEACSTLSLRAVNLMAVVIYIPATLYIIQRRVWGSQAHFSAFSLVSFPLIWFYAALYYTDVWSTATVLMALAFALSPRVPFYMVQLSALMCAVSLFFRQTNILWAAVVAVIAIENSHYSNGAPPKNGALAQIFSTISYTFQIELPIFNILISYASVAVGFSFFLYINGGIALGDKDNHVAGNHIPQVFYCALFITTLGFPVWFTWAHLKAYISSSFSVLGLTVRPLFIFVLIPRLLKSYAIEHPFLLADNRHYVFYLWRRLLKPAIYSISVEDVMQSQDAIDPKTLDVISIGLKYALSAAIYFSLWNIWTTLTNSIPAAILVRGRGFNRRNGKSISPAIDLQCLTWPILLAMVFATLASLIPSPLIEPRYYILPYLFWRIYMTPTTAGKRVNTDARFLREWIWYMLINAATVYMFLYKPFEWAHEPGVLQRFMW

>sp|Q6C9T3|ALG11_YARLI GDP-Man:Man(3)GlcNAc(2)-PP-Dol alpha-1,2-mannosyltransferase OS=Yarrowia lipolytica (strain CLIB 122 / E 150) GN=ALG11 PE=3 SV=1

MALQLDLPTLHDLRVVLNADFLAALAALLLLAVILVPLCSYISLYAWSAILAFKLRSPPANWEKSIVKGVQANGTSTLFGFGFWQAAAVRRQLILQSNDPSYYSVTHVSRRSEIAISPEDRNTEFRNRAQDSGAPRRVIYGFFHPYANAGGGGERVLWAAVKDTLMYDDNIICAIYCGEQDLPTRTSPSTVLDAAVSNFHVTELADKELRKRIVFIGMRGRRLVDPKTWPRFTLMMQAAGSVWMAWHGISTLVPDVFVDTMGYPFAYPLVSWVTHVPVAAYVHYPVISKDMLATVSLKQSPVRAALAVAKLVYWRVFALTYTFAGSYCSVVMTNSSWTNNHMQHMWWYNHKAEHIKIVYPPCGTQALSEIAMSEETSARSPNIVYIAQFRPEKRHDIVLREFNKFYKEYTEKYPNQPAPHLTFVGTVRNDDDKSRVYLLRLQARDLVNPDSVSFVLDAPFDKVRDILRTASMGVNAMWNEHFGIVVVEYMSAGLIPVVHNSGGPKCDIVVPYEGQSTGNSGTLSAMPSSTSIRSHYEAVPPGPTGFHFNCPGSDPTTDSGPSYDGEPIGTLAETLMRAFELSESDTHNMRARARESVKKRFSNEQFGSHWQVRMRILEKLEQIRRGHRLTRGDFD

>sp|Q6C3P1|ALG13_YARLI UDP-N-acetylglucosamine transferase subunit ALG13 OS=Yarrowia lipolytica (strain CLIB 122 / E 150) GN=ALG13 PE=3 SV=1

MHESSALSKLYNWDCSVVFIILNIYTTMLVLVTTGGTVPFEALIELVLSHESITTLSQLGFSKMRVQYGRGNRHIFTKHHKEGVMSITGFEYTDDLAGEMSRAHLVISHAGTGSVLDALRIGKHPVVVVNSKLMDNHQIEIAEELFRKRHLLVSGDTDSVGFIKALKMHREYLFETLPDPEEGILQRIIEETVSFM

>sp|Q6CF02|ALG14_YARLI UDP-N-acetylglucosamine transferase subunit ALG14 OS=Yarrowia lipolytica (strain CLIB 122 / E 150) GN=ALG14 PE=3 SV=1

MVTTILIAASAILVLLLLRLLFVLPASNRFGFLYRPKHSNPKLMVMMGSGGHTGEMLRMLKTLKLQSYAKRVYVSSSGDVDSLEKVKVLESTTKTDIKTMVLENIPRARKVGQSYPSSVITSAVSFAVAVKLVHKHKPHVIVCNGPATCVMLCYAAFLLRFMALIDTRIIYVESLARVNRLSLSGLILLPFCDRFLVQWPQLAEKYPRAEYHGILV

>sp|Q6C3K2|ALG1_YARLI Chitobiosyldiphosphodolichol beta-mannosyltransferase OS=Yarrowia lipolytica (strain CLIB 122 / E 150) GN=ALG1 PE=3 SV=1

MKAWHWSVTLVVIYLAIPVILYLLTRKDDRKPLSDIRKRKRTIVLVLGDLGRSPRMLYHARSLARSGHKVDLCGYDGAKPFDEILNNDLIKIHHIPLILNTRKLPFVVFGILKVIRQHWLLISLLYKLRGADYLLVQNPPSIPTLGVVRFYNLFLSTRTKVVLDWHNFGYTILALKLPETHPMVKFAKFYEGFFGGRAFVHLCVTVLMGQAMRKTFGMSGRRIVPLHDRPAFHFKPLSESEKLDVLRDFKETLYDDMTADHKIIVSSTSYTPDENFNILLDALALYDESKLDLPPLRVIITGKGPMMPEFLAKVEKLQLKRVSIRTAWLEFADYPRILGAAHLGVSLHESSSGYDLPMKVVDMFGCGIPVVSVDYAALSELVKTNTNGVAVKGHVEMGNTFMSLFSNRGKLDNIKRGAMIESRNTWDQTWVKTVGPLFDIGEYVQQRPDEDYDFSSSSSDDDH

>sp|Q6C3V7|ALG2_YARLI Alpha-1,3/1,6-mannosyltransferase ALG2 OS=Yarrowia lipolytica (strain CLIB 122 / E 150) GN=ALG2 PE=3 SV=1

MRVAFIHPDLGIGGAERWVVDAAVGLQNLGHEVDIYTSYCNKSHCFDEVRDGLLKVTVLGDTICPHTIKGKFAIFCATFRQLHLAYELKKGPGSKVDVFVVDQLSACVPLLKLWFPKARVLFYGHFPDQLLVQNRNQMSLVKKAYRYPFDKFEEITTASADRLVVNSHFTKDMFEKTFPATKNPLVIYPCVDTDIKEQQQGLDRDMITAASQYTFLLSINRFERKKNILLAIEAFGEAQKKSSNLKLAVAGGYDFRVNENVEYLQELILACEKLKLSHISITADKYAKLLEKDTPAAVWTSIFKNDVIFFPSASNSFKNTLLHISKLLLYTPQNEHFGIVPLEGMLWKTPVLATNSGGPLETVKDNVGWTVEGKSELWAPVIDKVVHMNASDYAVLQTECVNWVNRFSQDTMASELEEAMEEVRKKAPTENVGWDYIRLGMWYSVLMTLTLSIVLLAIWP

>sp|Q8J0I8|AOX_YARLI Alternative oxidase, mitochondrial OS=Yarrowia lipolytica (strain CLIB 122 / E 150) GN=AOX PE=3 SV=1

MTVRDWANRGQTYVNARAPNLLGRFRSTDDEDENNPSTELATDTTSAYGSTAASVVTMANSKPDDVSLYATSSHHHEYFTGSAWIHPVYTKEQMDALEVNHRKTETFSDRVALRAILLMRIIFDLCTGYKHPKEGEAHLPKFRMTTRQWLDRFLFLESIAGVPGMVAGMIRHLHSLRALRRDRAWIESLVEEAYNERMHLLTFLKLQKPSVQMRTGLLIGQIIFYNLFFISYLISPATCHRFVGYLEEEAVITYTRCLEDIDAGRLPELASMEVPDIARTYWHMEDDCTMRDLIQYVRADEAKHCEVNHTFGNLHQTSDRNPFALVIDNGRPQPSKDLTTFRSVGWRRDEIAN

>sp|Q6C1X5|ARO1_YARLI Pentafunctional AROM polypeptide OS=Yarrowia lipolytica (strain CLIB 122 / E 150) GN=ARO1 PE=3 SV=1

MFAEGQIQKVPILGKESIHIGYKMQDHIVSEIVANIKSSTYILVTDTNIEDLGYVESLKTKFEAAFAKDGIKSRLLTYTVAPGETSKSRATKAAIEDWMLSKGCTRDTVILAVGGGVIGDMIGYVAATFMRGVRFVQIPTTLLAMVDSSIGGKTAIDTPLGKNLVGAFWQPVNIFIDTSFLETLPVREFINGMAEVIKTAAFYDAEEFTRLESASEIFLSTIKKRDAKDPRRVDLSPITDTIGRIVLGSARIKAAVVSADEREGGLRNLLNFGHSIGHAYEAILTPYILHGECVAIGMVKEAELSRYLGILSPVAVARLAKCIKAYELPVSLDDATVKARSHGKKCPVDDLLRIMGVDKKNDGSTKKIVILSAIGKTHEQKASSVADKDIRFVLSEEVIVGEAPVGDKKSYTVTPPGSKSISNRAFVLTALGKGPCKLRNLLHSDDTQHMLEAIELLGGASFEWEADGETLLVTGNGGKLTAPAQELYLGNAGTASRFLTTAATLVQKGDKDHVILTGNKRMQERPIGPLVDALRSNGADIAFQNAEGSLPLKIEAGVGLKGGLIEVAATVSSQYVSSLLMCAPYAQTPVTLSLVGGKPISQFYIDMTIAMMADFGVVVTKDETKEHTYHIPQGVYTNPEEYVVESDASSATYPLAYAAMTGHTVTVPNIGSKSLQGDARFAIDVLKAMGCTVEQTATSTTVTGVPNLKAIAVDMEPMTDAFLTACVVAAVSEGTTVITGIANQRVKECNRIEAMRVQLAKYGVVCRELEDGIEVDGISRSDLKTPVSVHSYDDHRVAMSFSLLSSIMAAPVAIEERRCVEKTWPGWWDVLSGVFNVPLE

GVTLAKTVSKAESGLSKPSIFIVGMRGAGKTHLGAQAANHLGYEFIDLDQLLEKDLDTTIPQLIADKGWDHFRAEELRLLKQCLNDKSEGYVISCGGGVVETPAARDALQTFKGVGGIVLHVHRPVSRILEYLNKDQSRPAFVDDLEAVWQRRKELYRSVSSNVFFAPHCDSAEATAKVQQMLGAFLDRVTGKSEFVIPHKDQFTSFLSLTFPDVSIAATMLPSLSEGCSALELRVDLLNENDEAIPSEEYVLSQLAILRQNVDLPILYTVRTKAQGGRFPDDKPVELANLVNLGLKTAVELLDVELTYPAELVSSVGASRGYTKLLGSHHDFPGALNWSSLEWENMYARAEAVPVDVVKLVGMAKSFSDNFALENFREAHTSSPLLAINMGSHGQLSRVTNTLLTPVTHADLPVAAAPGQLSVEEINQTRSTIGMFNKNLSFFIVGTPIGHSKSPILHNTMFKKLGLPYEYSRFKTDDAAAVNAKARALLAQGNLGGISVTIPLKQDIIPFLDEVSPLAQQIGAVNTIIPGPNGTLKGDNTDILGLVNALTRFGANSLDKKTALIVGAGGTSLAAVHGLRSLGFAKILIANRTLSKAEAIADKFDNVEAVTLDSFVANKYTPSVIVSCVPATTFSMLDESNKLVSAALAASPKGLVLEAAYSAEATPLLKQVMDVEGWEFISGLYMLTEQGFEQFRLWTGIPAPKEVGEKAVLGN

>sp|O43108|ATC1_YARLI Calcium-transporting ATPase 1 OS=Yarrowia lipolytica (strain CLIB 122 / E 150) GN=PMR1 PE=3 SV=1

MDSHTSTEGVPLSETNNRSHATPSAQYCQMTVEETCSKLQTNPETGLTSSQEAMHRRDIHGSNEFAQEEEDSLIKKFFEQFSENPLLLLLIGAAAVSFFMGNHDDAISITLAILIVTTVGFVQEYRSEKSLEALNKLVPPEAHLIRAGNSQTVLASTLVPGDLVEFSVGDRIPADCRIVKAVHLSIDESNLTGETTPVTKDTNPVTGTPPIGLADRTNTAYMGTLVRDGNGTGIVVGTGSHTAFGAVYDMVSEISTPKTPLQASMDNLGKDLSLVSFGVIGVICLIGMFQGRDWLEMFTIGVSLAVAAIPEGLPIIVTVTLALGVLRMSRQKAIVRKLPSVETLGSVNVICSDKTGTLTRNHMSCTTCWTVDMGDLANAVTLKPGQSHTEADPKAVAALKNSVSLANMLKVGNLCNNSKFNREAGHLVGNATDIALIEVLDYFGLEDTRETRKRVAEVPFSSSRKWMLTSTTTGDSSTPMISVKGAGEVIAPFCEYYCKKDGKTAPFNDDMRKKVTEIASEMSNDGLRIIAFAYKQGKYEEGSEEAPEGLVFAGLMGLYDPPRPDVPRAIRRLTTGGVRVVMITGDSAATALSIGRRIGMPLMPGTQSVVEGSKLATMSDQALDECLQTASIFARTSPEDKMKIVKGFQRRGDVVAMTGDGVNDAPALKLADIGIAMGQGGTDVAKEAADMILTDDDFATILSAIEEGKGIFNNIRNFITFQLSTSMAALSIVAVATIMGLENPLNPMQILWINILMDGPPAQSLGVEPVDPDVMNKPPRPRNEKVMTPDLVKKCVEAAVIILVGTMLVYVTQMQDGVIDKRDTTMTFTCFVFYDMFNAL

ACRSATKSVFEIGFFSNKMFLYACGASIIGQLAVVYVPFLQSVFQTEALSVKDLLSLVLISSSVWILDEAKKYFLKSRSTNNYTNSVV

>sp|Q6C2N7|ATG15_YARLI Putative lipase ATG15 OS=Yarrowia lipolytica (strain CLIB 122 / E 150) GN=ATG15 PE=3 SV=2

MKQDLYKESSPPPSTTKSKGLYVIVAALVTTAIYLLYSQGYSNTHGEKDMPSVVPNLVLPANPSSDHSFAVKHIYHHNTEADANHGRMDVSGEWVRAAQKAQHLNLGQDTHRYMASNARDPYTNLPLKSRTQKVKRWRQRDPDHVESYLEAARLNPQLYGAMDFDWVEEDILVPDVTDRDTVVSLAVMASNAYVDVPFTGDWTNVSWKETGGIGWQSDGVRGHIFVDQTPGSPLVVIALKGTSAAIFDSGGDTVINDKTNDNLLFSCCCARVSYLWNTVCDCYTGESYTCDQECLEKELYAEDRYYRAVLDIYRNVTHLYPQKQIWVTGHSLGGALSAMLGRTYGIPAVGYEAPGELLPTKRLHLPSPPGIPWSQEHIWHFGHTADPIFMGVCNGASSSCSIGGYAMETSCHSGLQCMYDVVTDKGWHLSMVNHRIHTVIDEVLLAYNETAACVPPPPCQDCFNWNFVMGNDKDDDDKDKKKKKKTSTSSSVVSKTKTSTSSTVATNTMPSLPDPTCVERNWYGKCIRYDPEIKQQYGDSHTVTHVTMA

>sp|Q6CD56|ATG22_YARLI Autophagy-related protein 22 OS=Yarrowia lipolytica (strain CLIB 122 / E 150) GN=ATG22 PE=3 SV=1

MDIVATTQKELYGWYAYAWAAEPFMVVAVATYIPQLLQSYARQNAVLADDHSQPCDSPPVPFPGDPGVPTDPGIPPNNSLSSSVPWFLRANEIQLLESPDTVHTMKDHKAKPTQPTCVIKFFGIYIDTASFPLYTFSLSVLLQVVVVISMSGAADRGRFRKQLLLFFGIAGALTTGLFVFITPKRYYLGSFLAIVSNAAFGAATVCGNAYLPVLAAGMKDGTTSEEPSEPSTPSDTSKPASRSENTPLLSASGVDYETGESSNTAEIVKIDHRANVSARISGTGVALGYLAGFIVQIISIYLVITTGSTTWSLRLALLIVGVWWLIFQIPVLMWLKPRPGPPLPIKTDPQNHPWTATLDRVTNGGWSYVTYGWKTLLVTFKEARQMKDVALFLVGWFLVSDGITTINSTAVLFAQGELRMSPANLAVMGMLVVISGISGAKLTPLIGGTRASPIKSIVVVVSLAAAVPAYGILGFFFTNIGLKNPWELYVLAVWYGFALGGLNTVCRSTFSMLIPRGKEAVFFSLFSVTDKGSSVLGPLLVGLIVDKTHNLRHAFYLLLVLLITPIGLFLMIDMERGRKEAEYLETVEE

>sp|Q6CBS9|ATG27_YARLI Autophagy-related protein 27 OS=Yarrowia lipolytica (strain CLIB 122 / E 150) GN=ATG27 PE=3 SV=1

MKSAIIGYMAVAVAAASCQFTVDSKNYDLSAISGPKSVEYTIETPPSKRKMEFVLDPCASLKQDKKKPADEQCPDNTIVCGLGYILLPKEKDFVLSEVMPFGNGPAPQYQPLKTGPEGTEGLSTSYGNPWGSEKLDIDVNYICSDKEEGPKLENVGLGLNNYYEINWKTPAACINDGSKPKQPVKEPGKTPNDGDDASNGNPSWGWFTWLFIIIVLGVAVYIIGNAWINYDRYGNAGVDLLPHADSLRDVPYLIRDLIAKVVGTFTGSSRTGYSAV

>sp|Q6C2F5|ATG9_YARLI Autophagy-related protein 9 OS=Yarrowia lipolytica (strain CLIB 122 / E 150) GN=ATG9 PE=3 SV=1

MTDKSTFLSVLFGGGSVYQDLDGDGEVEDAEILRRVEEEHAQTSDNSNSDNDSGNDSDVPTSLMVEGVQDPKPGSKRRQPHRMATLSNLQSSAGPGARSVSFAQGTKTQTPIRLTKPTGVANGGLPRHKQDLGASIRTMVDPKELALWKWANVQNLDNFFAEAYMYYTGKGLVSIILSRVLNMSTIMFVVVFSTYLGSCIDYSKIKGSRTLDEVHVKQCYAKLGSFHVFVLWTFFVLWFMKLFQYVKDIRRLVDMKSFYQELLEIDENELQTISWPQVAKRMATLSEANAATQVGNSTKQRIEPHDIANRVMRKENYLVAMFHKRVLNMTVPLPQPLQRIFGRPQLLSRALEWNLSLCILDYVFNPAGQVRPMFLKSTHKQILSTGLRRRFVFAAIMNVVFAPFIILYLALLYFFRYFNEYHKNPASIGTRRYNPLAEWKLREYNELPHQFERRLTLSYIPASKYLDQFPKEKTALVSKFVSFIAGSFAAVLGIASLIDPELFLMFEISANRTVLFYIGVFGSILAVSRSLIPEETLVFDPEISLRYVAEFTHYLPPEWEGKLHTEQVKNEFSLMYEMRLIILLKELASIFLAPFILYYSLTQSCDDIVDFIRDHSVHVDGLGYVCTFAMFDFKQKSPDHQPDEDQKMLKSYLYFMDHYGDKPTNVSQTQQNVPMYSSVRLKNDGLDLNNSIMQKFQKHNGHHALSGLTQRDVGLSPAAPTATTATSGTATGAAPRRDLINFDVDESFIDQTTSNRDMDNDEQPKDKERVVDMLNQFYKKTDNMNLGA

>sp|Q6C6N0|ATM1_YARLI Iron-sulfur clusters transporter ATM1, mitochondrial OS=Yarrowia lipolytica (strain CLIB 122 / E 150) GN=ATM1 PE=3 SV=1

MWLSLPRSGYGSVATLTSKRVLACLTPLRQFSTSPAVSNANHKNVDNINKSPANDAANNAVEKGDKPTTSPEKLATKAEKSSANSVKAAANALGESNLSNSEQRRLDWIIMKDMLKYIWPKGKTSVKFRVLVAVALLVGAKLLNVQVPFFFKEIIDDMNIEWNSATALGVGITALIFSYGAARFGAVLFGELRNAIFASVAQKAIKEVATNVFRHLLKLDMAFHLSRQTGGITRAIDRGTKGISFVLSSMVFHIIPIALEISLVCGILSYNFGWKYALVTGATMVSYAIFTITTTSWRTKFRRNANRADNEASNVCLDSLINIEAVKSFGNEGYMVDKYQSALTKYEKASIKIATSLAFLNSGQNLIFSSALTAMMYMTCCGVADGSLTVGDLVLVNQLVFQLSVPLNFLGSVYRDLRQSLLDMGSLFSLQKVAGQIQESPNAKPLQLTNGEIRFENVTYGYHPDRPILKNASFVIPGGLKTAIVGPSGSGKSTILKLAFRFYDTQEGRILIDGQDVREVTLASLRSAIGVVPQDTPLFNDSIMNNIRFGRLEADDKEVENAACAAKLDALVRQLPDGWNTNVGERGMMISGGEKQRLAVARVLLKNSPVVFLDEATSALDTNTERQLLANMDQVLGDKTCVAIAHRLRTVADSDKIICLNQGGVEEEGTQAELLLKDGLYKSMWDAQEQVELGEEGIKEAEEKAAKKDV

>sp|P32907|ATO2_YEAST Ammonia transport outward protein 2 OS=Saccharomyces cerevisiae (strain ATCC 204508 / S288c) GN=ATO2 PE=1 SV=1

MSDREQSSGNTAFENPKALDSSEGEFISENNDQSRHSQESICKIYTAGKNNEYIYIGRQKFLRDDLFEAFGGTLNPGLAPAPVHKFANPAPLGLSGFALTTFVLSMFNARAQGITIPNVVVGCAMFYGGLVQLIAGIWEIALENTFGGTALCSFGGFWLSFGAIYIPWFGILDAYKDKESDLGNALGFYLLGWALFTFGLSVCTMKSTIMFFALFFLLAVTFLLLSIANFTGEVGVTRAGGVLGVIVAFIAWYNAYAGIATRQNSYIMVHPFALPSNDKVFF

>sp|Q36258|ATP6_YARLI ATP synthase subunit a OS=Yarrowia lipolytica (strain CLIB 122 / E 150) GN=ATP6 PE=3 SV=2

MNFIINSPLEQFTTRVYFGLSSGLINLDTITLTSFSIYSIAVVALILGFSILNDNNTNILPTRWSLAFESLYFTVEKMVSEQIGGLEGRLLFPFMFSLFMYILIANVVSLVPYSYAINAQLIWTIGLSVAIWIGCTLTGLANHGAKFFGLFLPSGTNLPLVPVLVIIELLSYIARALSLGLRLGSNILAGHLLLVILAGLILNFISISIFTFALGILPLSILLGIVALESAIAFIQAIVFTILTCSYIKDAIHLH

>sp|Q36257|ATP8_YARLI ATP synthase protein 8 OS=Yarrowia lipolytica (strain CLIB 122 / E 150) GN=ATP8 PE=3 SV=2

MPQLVPFYFTNQIFYGFASLSVIVYLFSIYILPHYLEIYVTRIFITKT

>sp|Q37695|ATP9_YARLI ATP synthase subunit 9, mitochondrial OS=Yarrowia lipolytica (strain CLIB 122 / E 150) GN=ATP9 PE=3 SV=1

MQLVLAGKYIGAGLASIGLVGAGIGIAIVFAALINGVSRNPALKGQLFTYSILGFALSEATGLFALMIAFLLLYAV

>sp|Q6CB37|BIG1_YARLI Protein BIG1 OS=Yarrowia lipolytica (strain CLIB 122 / E 150) GN=BIG1 PE=3 SV=1

MKLALLSLIGTALATPAVMLSHKELTAFEKYTNKQTSNSISAVELDRVARKLMHDCSANTYVVVNQPGLKAKDFQTESAFPFLKKLLERTSTMYTVPYADGGIELGKLANSVAKQCGAVKIDVNLDNNITPLEEYMDTTKRVIEINFEPLPNTYVSRLAALAANDEMLEKIVRATPSPFIALILTSQKGEEGDFETRNPDFKIFPGVARAKTVPGAKDKYKYHMELQNVPVDEKKLTAEALLKVKTPPTHAPEVRQTGDGELVDDKLLLMIVGSVVAILLSLLVFNGLMAAKPDVVKVDKTDAKVEKAVKKEVQKKERLVNGRVEQLKRETR

>sp|Q6BZQ6|BOS1_YARLI Protein transport protein BOS1 OS=Yarrowia lipolytica (strain CLIB 122 / E 150) GN=BOS1 PE=3 SV=1

MNAIHNHLVKQNASLKKDLSEFSANPAGAPMSLQGQISATMTSFSRTLDDYSEIINKEHNKEKKEKAEARLARFREELADARSEFKNLRSAREEKTLEENKTALFGDNPYGESRNRNVNRDVPIQPTYTDLTREEGMQREQSSLNQVGQQLDSFIEQGMAALGDLQEQSDILRSTGKKMRSVAETLGLSRETIKMVEKRARQDKRFFYGGIVFMLVCFYYILKWFS

>sp|Q6C2Z2|BST1A_YARLI GPI inositol-deacylase A OS=Yarrowia lipolytica (strain CLIB 122 / E 150) GN=BST1A PE=3 SV=1

MHIATFPALAITALALVLWATVATHSSNTNSCHMSYMKPDMIAMTGFNTTQTPLAHKYSLHLYRELDVDLSREVGGRPVLFVPGNGGSMRQIRSIAGEAAVQYWHDPRRAGADADTWANGSTARPKSALQKLNTFAFGDTESDTEGVRGLGDAVTARDMSGDDRRDAVERPLSNGKLPSQWPDDLPLDFFTVNFQEDLTAFDGTTVIDQAEYLNQAIAYILSLYSSHPNPPTSVIVIGHSMGGIVARTMVTLDSYIHGSINTILTLATPHVLPPVSFDKGIVGLYHNVNEFWKTETVPGGKLEDTLLVSVTGGIRDQMIPAEYSSVDTFLPPTNGFAVATTSIPDVWMSIDHQAMVWCHQLRRVVAETLLVVAGETTEKVSTRLDTFQEYFLSGMERVEKKAQNASESSKLTLDTLVSINTPLTTTSNIIINDHSPNQLVKTKSYFKFPVLKASEINEMSRSFAMPVDKGKSLLVKTNMPLEDLHILVCRTNTGDVDTNGFSFLRYGSKSKGVRVGTDTLVCANVAGEAVAMPSSIKYATGAKIPEEEETGEDNDIPVDSAISSTSLSEYIQLDASSLSGFQYVVVIDNTMSETISSDSYLLAEMALPSDMAVVAAPTWWEVLKVGRFTIELPEKRALLTKISLPHFWSSLVAFSIRLSTSDSFSMEYQCEAKKSMGSEHDVLFAPLLMQYSAQLHEAKFSTNLCGGYEQGTRVAVHGGAPYMPLAEGRDVSGTELYLWTDSSSRSSESLSLTLEIDLWGSLGRFLGFYRVMFAIFPMFVFLCLLMIQLRVWTTTELFVSLSDALDVFVDFQLPWILAGSCAIPFVPHVLVSLLYPQMSQ

PGQFFLGLNGTHLWFLGPVSLVIATGIVVVLHWLLQILTLWVCQCYYMLGLAPIAPESPFSVRRIVTISFLLLLVFKIVPHQFAFMVAVLVMAMGAGKARVGRLMQSEDKDNKSEPCVVIDRNLINYTHSLLLLLVLLLPINAPTLVVWLHNMANKWHTPLESHHEVSAILPILLLVLTASRGIMIRLPQSKRAIYATFAFLAYFALFVLFHGVVHSYRLHLLTNGLCLCLLYLSL

>sp|Q6BZU7|BST1B_YARLI GPI inositol-deacylase B OS=Yarrowia lipolytica (strain CLIB 122 / E 150) GN=BST1B PE=3 SV=1

MRRININASVALWTVFTILTIWISFALHQPDVQTCDIARTWISTAHVEGFDSKHSRFGEKYSLHLIRASQHAIPQPIRPSGVPVIFVHGNAGGFRQIGPFAGIAQELNDELRLLTKGDAGTEFDFFSIDFNEAYSALHGRTLLDQAEYLNDAIAYILDMYKRNQQEGLQVPESVIVLGHSMGGIVSRVAVTLENYRPQSVNTIITLASPHLIPAATFDADITKVYHLVNDYWRAAFAEGDTNDNPLRDITILSIAGGKSDTMVPSDYVSLDSLVPATNGLSTFTNSIARVWTGIDHDAVMWCHQLRRQIASALFHIVDPNVPSQTKPREVRMSTFHRSFSGSQSLSSAMQDFINIEATPLQDGVEQRLAPGFYWGRNLQMLTNHVINYDSNIDLYERKSGSLLKVWQCRSRQGSSFRQCKRIYPLFVPGINDSVLSHVSVNGILLLDVSKEASESGDWINIDETSMSAAPFNIYGNIVFSTSNMVSQDIAFPALTSGLISYKVLTSGGVGLIRQYMGRNHPSRTYDSKYLIPHYARVDISFHGDGAPFVPFKLKTPTKTDLSTKSYKAPLHLQVFGQGKVTISVDYVGSLGNLFMRYRTLLFSLPTAVLYAVLLLQFWRYYQSGSDAKFLSLRDATGLFIKQYLSWACLVVAGLSFVIKFEFIRDFLHFIQIPATGSSKSYEIETFYGSLYTHIDLFLGISGPIGVVLAPAFLALATGIVVVVTEIVIAVTTLASLAISRGHRKMSLLQTPKNVTIQSSDDPIGDLLHKRTVIIALMALLVLLFVPYQLAFALATASLLALTAYFDAEESASASHDLSTSLYANRQAQEKVGSFINYATT

MSVVMVWTTLVNIPVLAVWVQGMVFGRSTIFSSHHNLLSVLPTLLFIENLSFRRMPERGLPFVTYIILGYACFNCTAYGMMHAFMIHHWFNLLAGWLLITSYKRNKTVIKESRIE

>sp|Q6CF60|BST1C_YARLI Putative GPI inositol-deacylase C OS=Yarrowia lipolytica (strain CLIB 122 / E 150) GN=BST1C PE=3 SV=1

MWVAYSPIEGLTTEHSRLAEKYSLYLVKSTPYDIPLPVRPSGVPVLFVPGNAGSYRQIRSISDTCRELNEQYGGSEIDFFALDFNEAYSALHGRTLLDQAEYLNDAINYILQMYRDNGKDVSSVMLLGHSMGGVVSRLAISLDNYKPGTVTTIFTLASPHLVPPATFDGDIQKVYNRMNDFWRSNYADSDNNSLSDMTVLSIAGGKRDTMVPSDYISLDSVVPSSHGLSTFSNSINRVWTGIDHDAMMWCHQLRRQIAIALMNVIDRDVNGRMEVFRKVFSGTQTLSDAEEDFEDVEVTPVKHGLHQKLESGWYYGENVQVMTTHTVNQESAFESYEMSSGSLLRAFECRSKSGSSFKGCRKIFPLLVPGSNDAVIAFAETEHYLLLDVSSSSDWISIDQVSQKSASFDFVTGATISTSNTISTDISFPKLTSGLVSYKVSVSKGVQLVRQYVQQNSSRVYDSKYLVPHNGVVDVSFHGDVPFVPYFQSSPLHLQVFGGGHVTIRVDWIGSLGNLFMRYRILFISLPSAILYAIFLVQFHAGTARFLSLRQSTSIFINRYLLTSCLAGSGIAYLTGLSQVRDFLHLIQIPITKTFAVDPSYTKNDLFLGLSGVSGTVLAPIFTVFSTGMVVLITELVMGLTSLLSFCFKSTTTAQSESESGDPISDLLHKRTVFVAVISVLVLLFFPYQLAFTLATVALLVMTAYFKSNKAPQEQSFNNYISTICVLMTWTCIINAPVLAVWIQGIVVQRSMTFSSHHNLVSILPTLLFVENLSFRRIPSGSSITSLLLAYTSLHCLFYGMMQAFMIHHGFNLLATWLLCMSYKKVFFKSKHE

>sp|Q6BZW3|BTN1_YARLI Protein BTN1 OS=Yarrowia lipolytica (strain CLIB 122 / E 150) GN=BTN1 PE=3 SV=1

MQLEPAHLVYAAFWTFGLVNNVLYVVILTAAVDLVGPQTPKAVVLLADVLPSFLIKLAAPFFIHKIPYNRRITLLVGLSTVGMMSVSLASPLFLKLVGVVLASFSSGLGEVTFLQLTHFYDTRALNGWSSGTGGAGLVGSGLFMLLTTVLGVSVKTSLLVFAFFPLAFLGVYFYLLPPRDYNRVPGAFPVHSNDPETGIETQPITWEFVASGYETTMVRLKPLVMPYMLPLFLVYFSEYTINQGVAPTLLFPMEELPFSKFRDVYVTYGTLYQLGVFISRSSAPFVRIRRIMIPSVLQFANLVFCIAQSMSPILPNVWLVFILIFYEGLLGGAAYVNTFLLITEQADLAEREFALGSVGMSDSAGIVLAGLVSLWLEPGLCNYQVNDGRGWCTLE

>sp|Q6C5S0|CBP4_YARLI Assembly factor CBP4 OS=Yarrowia lipolytica (strain CLIB 122 / E 150) GN=CBP4 PE=3 SV=1

MRLENWPIVEMFRSRPGVPNWPKFGLFAVGVIGSAYLGYRYATPSEEDIVRRMNPELRERYMLERDARQEYFNEFVKEAIAQSKTNEPIWKVGPMASKPIDFNVAVREKMKEIEARNDQDRNERIKNELAAIAKKEEEEKNKKGWW

>sp|Q6C0Z6|CCPR_YARLI Cytochrome c peroxidase, mitochondrial OS=Yarrowia lipolytica (strain CLIB 122 / E 150) GN=CCP1 PE=3 SV=1

MRSFRAVRNFSTTAKRLSQAPKASTPNASSGNGFVLAFVAAAAGAGAYYYYANSPAAKVETFNATKADYQKVYDAIADKLIEDDDYDDGSYGPVLLRLAWHSSGTYNKSDNKFGSSGGTMRFKPEASHAANNGLVNARNFLKPIHEKFPWISTGDLYTLGGVTAVQELGGPIIPWKRGRVDEPESASPPDGSLPDASQGATHVRNVFNRQGFNDQEMVALIGAHALGRCHKQNSGFEGPWTFSPTMFTNDFYKLLLDDKWQWKKWDGNPQYEDVKTKSLMMLPTDMALATDKNFKKWATAYAKDQDLFFKDFSAAFSKMLNNGVDFPQGTEIWEFKPKNA

>sp|Q6C6U9|CHO2_YARLI Phosphatidylethanolamine N-methyltransferase OS=Yarrowia lipolytica (strain CLIB 122 / E 150) GN=CHO2 PE=3 SV=1

MAVPSAAIKASGVDMGDLKKRHDTKLDVEESVIDEKINENESDEKLTEKLTEKLTEKLTEKTSTEPIAYVGKCPDGTTFIVPETEDMLTNLFDPRITKSLTDVIIVSILVTFMGLFFVVPKSWRVPLYLFLFAAWRLAYNGGIGWLLHNQSNYHKLTKWALKYKVFDKDNKTWWHQLIKREFETRFQNQPNYSFYEVPVEFNTWILFRHVVDLILMSDFTCYFMLAWSCALSVRTNQPWWLVIGRWLAGALMLAFNLWAKTDAHRVVKDYAWYWGDFFFLKDMELTFDGVFEMAPHPMYSIGYAGFYAASLMACSYTLFLASLAGHAAQFVFLSIVENPHIEKTYNPHQPKQRRKASHVRDRSSIQLGLDQKQKDELSAIVEGDESVSIASTPISTTFDEPETVQKHSLPPLVVFSNFQITRVTDIMTVGAAVYTMLLYFLPANRLWYTVTFFMALSARLFHTLGLGVILRRQSERRSFTKTFLKFGIYPFEAYEQWQVWYNVSTVLSYTTFGLFCLRQWRAPSTDPLWPLKYILGALLIALHGWTSKSIHDSLGHFGWFYGDFFLDRKQNLTYSGIYRYLNNPERFFGIAGIWGLALMTNSPGVGILAFLWTLEGMAFIKFVEQPHMQRIYGTSIRHDAGVTKTIKNSLKLPSPFEKRVRQFQGSVDKVINDTLTVVEEFIGLAKPTLNEVVNDSKILLRQYPAKLTLTRMLDVPENIDTADYSVKLVGGEKCADSNRISYAFGTPIHVKWQASPNRSAKDWIGLYRVHDNTSPEVTSLPSKGRWSGIDETGHESHLDGIVSSSKTNGEVVFRGDTLFWECGVYEFRYHHAGKHTVLST

SEPFEIVAPKIDLSGEVDAKTLALEILPIVQRCFSLSMEFPPEEVDDYWALEEPKVINRVQAAVQAYFNVELAQEVLQSDESVENLAERLLRIRDALKGLTV

>sp|Q6C8U1|CHS7_YARLI Chitin synthase export chaperone OS=Yarrowia lipolytica (strain CLIB 122 / E 150) GN=CHS7 PE=3 SV=1

MGFGDFDFLCNKSPLPLCMLVGPYDKPTTDQTPLLNGIGLMSECYPRSIELANTIIFQVGNTFIHIGALPVILIMMYTVKGKYTAIGRKELFHFLSCFLFLTCMSLVVDAGVAPPGSAAYPYLVAIQNGAISGTMWSLVNFGFLGFQFYEDGTRRAMLFLRGTTLCAFLLTFIISLFTFIPSWGSDAIGPHNTVGLFVVLYLFNLIFVVVYILSQFALAIFILQDIWMIGAVALGTFFFVASQILLYPISSIICKQVKHYIDGTFFATVTNLFAVMMVYKFWDMSTKEDLEFSVGQKDNMWETKELLGEDNGMSRYEVNGSEYAGSTFALNQHQF

>sp|Q6CHT7|COA3_YARLI Cytochrome c oxidase assembly protein 3, mitochondrial OS=Yarrowia lipolytica (strain CLIB 122 / E 150) GN=COA3 PE=3 SV=1

MSQDPHNGKGTFEVHKQPQPGLKVFRKGKYIDPKTFQMSPALIRARRPFFVRNMLALAGLTGFVAGIYGYTMYSLRTDDFGDVPIPPLDPEEIKKLQSKYSDDSKA

>sp|Q6C0Y0|COFI_YARLI Cofilin OS=Yarrowia lipolytica (strain CLIB 122 / E 150) GN=COF1 PE=3 SV=1

MFQPCCSKSTMSRSGVAVNDSALQAFNELKLGKKVTFIIYKINDAKTEIVVEEEGTTDSYDTFLGKLPENDCRYAVYDFEYEISSGEGKRSKLVFFTWSPDTAPVRSKMIYASSKDSLRRALTGISTEIQGTDFSEVAYESVLERVSRGAGSH

>sp|Q6C0L2|COX10_YARLI Protoheme IX farnesyltransferase, mitochondrial OS=Yarrowia lipolytica (strain CLIB 122 / E 150) GN=COX10 PE=3 SV=1

MLLSNVAVNRTVVHTQLVSGSRSALHALSRTSHSVPVTHTHQRRHIFSHKRRLSSSTLAIPFALSNTNSATTAPLQFLSNVSLCRVTPGTITTSAKATAPNLDKMSAEAATTAAQASASPVEDSFLKKAVSCQSETEREAVMAARAAKKALRETKETWWAPYVALTKPRLTVLVVLSAMSSYALTPEAVSLTNLLFLTVGTALCSGSANAINMGREPAYDSMMTRTRGRPVVRGAVTPNQAFTFAGITGTVGTAALYFGVNPTVAILGASNIALYGGLYTTLKRKHIINTWVGAVVGAIPPLMGWAASGGSLLHPGAWCLAGLLYAWQFPHFNALSYSIRDEYKKAGYVMTAWKNPGLNARVGLRYALLMFPLCVGLSYYNVTDWWFVLDSSVLNAWMAWWAFKFWQQENVNIALAAAGKQYSQNPFARKLFWGSVVHLPGVLLLAMIHKKGQWDWLFGPSEDEKKKTLSS

>sp|Q6CCF6|COX16_YARLI Cytochrome c oxidase assembly protein COX16, mitochondrial OS=Yarrowia lipolytica (strain CLIB 122 / E 150) GN=COX16 PE=3 SV=1

MLEQNKFKTKKQQQAWNKTLAGRYYNAMVKRPFLFFGLPFLTTIYAASVYFAEFTAYRYEIQDGKVKALSEEEALKLDKGRRKVDMKEEFYRLQQLGKQDDWEQVRVPRMKGESDNVF

>sp|Q9B6E7|COX1_YARLI Cytochrome c oxidase subunit 1 OS=Yarrowia lipolytica (strain CLIB 122 / E 150) GN=COX1 PE=3 SV=1

MSLKLNIQRWLFSTNAKDIAVLYFIFALFSAMIGTGLSAIIRLELANTGSPFLHGNTQAFNVVITAHAILMIFFFVMPALVGGFGNYLMPLMLGASDMAFARLNNISFWLLVPSLILILTSALVEAGAGTGWTVYFPLAGIQSHSGPAVDLAIFSLHLSGFSSLLGAINFITTFINMRTIGMKYENVPLFAWAVLFTAILLLLSLPVLAAGLTMGIFDRNFNTSFFEYAGGGDAVLYQHLFYWWNHPEVYILIIPGFGIISHAVSAIASKPVFGVQGMIYAMWSIGLLGFCVWSHHMFAVGLDSDTRAYFTSATMVIAVPTSIKIFSWLATLYGGTIRLNVTALFALGFIFLFTIGGLTGVVLANSALDIPFHDSYYVVAHFHYVLSMGAVFSIFCGWYLWSPKILGLHYNERLSHIHFWLMFIGVNVTFFPMHFLGLQGMPRRINDYPDAFIGWNQVASLGSIISIVASIVFIYVVYDQLTNGLHQGNKALDSQFKPSFMGTNLNVEGYTGPTLEWTVSTPPSLHAFNTPAVLY

>sp|Q9B6D5|COX2_YARLI Cytochrome c oxidase subunit 2 OS=Yarrowia lipolytica (strain CLIB 122 / E 150) GN=COX2 PE=3 SV=1

MFKIFNDVPVPYGLYFQDSATPTFDGIIELHDIVMFYIVVTIVLVSYLLFVIIKNFSNDHISYKYLTHGTTLEIVWTIFPVVILLFIAFPSFILLYLCDEVIDPAMTIKAIASQWYWTYEYSDFIGETGEIVQFDSYIVPTDMLENGQLRMLDVDARIVVPTNTHLRFIVTSRDVIHDFALPSLGIKCDATPGRLNQVSALLQRESVYYGQCSELCGVLHSSMPIALEAVSIDKFLSWLDEQ

>sp|Q9B6D8|COX3_YARLI Cytochrome c oxidase subunit 3 OS=Yarrowia lipolytica (strain CLIB 122 / E 150) GN=COX3 PE=3 SV=1

MNLTLKKFQVHPFHLVAPSPWPILVSFSVMSIMLTLVFNMHGFMHNNYWVVFSAIVAIMTMALWFRDIISEATYLGDHTLAVRKGLNIGFILFVVSELFFFIAIFWAFFHSAMAPTIELGGVWPPVGIEAIGPSELPLLNTILLLCSGATLTWSHHALLGGNRFNTLLGLILTIALAVTFMICQYMEYSNAPFTISDGIFGSVFYFGTGFHGLHIIIGIIMLGVSLWRIYTYQLTNNHHVGYETSILYYHFVDVVWLFLYIVFYWWGT

>sp|Q6CF50|CTU2_YARLI Cytoplasmic tRNA 2-thiolation protein 2 OS=Yarrowia lipolytica (strain CLIB 122 / E 150) GN=NCS2 PE=3 SV=1

MSAESTCRRCDGPTAIKTRQANFCQPCFITFIQQKQRKAMEGCKVLFARPGCVLPPAINILVPISFGQSSLALLDMAHAQLEEQAKTYENAAGFTLNAVFIDCSEADPLEKEPNQIISELEKRFAHAKFTCIPLSKAFEGASSVTLKHNRDYTSFVSGISEEPTSVQQLLSCIGTKSAREDIISVLQRHLIIEEAKKQNESLPTTVAWGHNATRLAELTLSLTIKGRGNRIHAQVLEHKNPKDSISGLPEIHPLNDVLSYEIPFYNSFRNVSDLAVDTVSKPSQVTKNLSIDQLMHQYFENIQTNFPSIASTVVRTAAKLDDPNAAKQGLTPCLICASPVDPNQSLAWLTNITVNEPAAPETEEEEELSKKAHMEKSQEKTGDADRHLPVPNLCYGCIITIRDTDSFTFPKRASKQDILDEFTL

>sp|Q9B6D0|CYB_YARLI Cytochrome b OS=Yarrowia lipolytica (strain CLIB 122 / E 150) GN=COB PE=3 SV=1

MALRKKNSLLNMANSYVLDSPQPSNLNYFWNFGSLLALCLVIQLATGITLAMHYTSHASLAFDSVEHIMRDVNFGWFIRYAHANTASFFFICIYAHMGRNIYYGSYKTPRVLPWSIGVIIFLLLIITAFMGYVLVFGQMSLWGATVICNLVSAIPWLGEDIVHFLWGGFSVGNPTLQRFFALHYLMPFVLAVFALLHLIALHTAGSSNPLGITSNVDKLSMHPYYSFKDLITVFAFLLMFTLFVFFSPDKLGHPDNYIPANPMVTPASIVPEWYLLPFYAILRAIPDKLGGVIAMVAAILILLILPIVDRSIIRGNAFKPISKLLFGFFICNFLLLGVLGQVHIEPPFIVLGQICTIFYFSYFLILLPMVSTIENIFFYIGSLRK

>sp|Q6C890|ERFB_YARLI Palmitoyltransferase ERF2 OS=Yarrowia lipolytica (strain CLIB 122 / E 150) GN=ERF2 PE=3 SV=2

MDFYTPPTTAGPPTRGSPESVPTTAASTFPLRKRTRAHLRARDKSDGPAPSSRGTSDRLW

SWVFLKQPLSLPEHNYQAHLGNNVFLIGGRFLSARQKPLNIAVLCVILILGGLYYGFVAP

WTWNHISPAIPAVFTYIFLLCVASFLRASFSDPGILPRNIHLTDRIADGSIPNEYSVEPG

IDAFDPRKNTTSLSCFKQPESSENLVYLKYCSTCKIWRPPRASHCSDCDNCVDFHDHHCI

WLNNCVGRKNYRYFVAFVMTGGLCGLYIVGNSIAHVICYKRHMHMTIAESLRHRPMPLVM

IFLGFLGAGYPLALVGFHLWIASRGESTHEFVSMNPVTKHVVDGHVGVTLSKCKVMGSHD

GFKRFSDAVLAVVCARLCAHVSPHSNPVTKQTTPQGVQKSTFRHWKRF

>sp|Q6C060|FCJ1_YARLI Formation of crista junctions protein 1 OS=Yarrowia lipolytica (strain CLIB 122 / E 150) GN=FCJ1 PE=3 SV=1

MIRATAMHRSIVQRRMLSTASRLQQAAKPATGSTVGTANGASAASGKPPGATFEGASKQK

KKTHKFRNFVLLSTFVGAGAFAGGVYYSLQNDQFQSLFVEYVPAAEHAINYIEEQQLRSG

RLKIATPTSKHDVDQSTVRVPKSGATWRAVDSTDAATSAKSASSPAANVAKDVASPAPAA

TASPVSSGSPKTDNTTVPAVRLANDSDPAVKAAVQTFNDLIAVAPSGAAKQLSAKVSTVV

DQLQHNVAQIKSEAAEEAKNSINKLNSELAKLKASTGEEISSKVSAAEQQLRNEFAALRA

HSEKVYHDRLRVEIEATKSLVSSHANNLIQAVEAERQKQYAQEIAERVETEREGRLSKLK

DLQTSLTQLQDLALKTEQAVDASGRTAALHLAIAKLTGALKGSEPVALGPYVESIRRAAG

DDPLLQAALDSIPEVAQTEGVLTPAQLTIRFKLLEPELRKSSLVPVNAGVAGHLGSLIFS

SLLFKKSGVPKGDDVESVLARANIALEQGKLYDAVAEVNTLKGWPRKLASDWLDEGRRRT

EIEFLADVIAEEGKLYGALSSKK

>sp|Q9Y753|FDFT_YARLI Squalene synthase OS=Yarrowia lipolytica (strain CLIB 122 / E 150) GN=SQS1 PE=3 SV=1

MGKLIELLLHPSELSAAIHYKLWRQPLHPRDLSKESTELRRCYELLDVCSRSFAAVIREL

HPEVRDAVMLFYLILRALDTIEDDMTLSRDIKIPILRDFTKCMKTPGWKFTDSDPNERDR

VVLQEFPVVMTEFNKLKPKYQEVIYDITDRMGNGMADYVIDDDFNNNGVDTIAAYDLYCH

HVAGIVGEGLTRITILAGFGTDVLHENPRLQESMGLFLQKVNIIRDYREDIDVNRAFWPR

EIWHKYAEEMRDFKDPKYSKKALHCTSDLVANALGHATDCLDYLDNVTDPSTFTFCAIPQ

VMAIATLDLVYRNPDVFQKNVKLRKGTTVSLILEASNVSGVCDIFTRYARKVYKKSDPND

PNYFRVSVLCGKIEQHAALIKRQRGPPAKTIAQLEGERKEMALSLIVCLAVIFSMSGLMA

YIAYVSGFRWSPREIFDSKMFPLRD

>sp|Q6CFJ0|FIS1_YARLI Mitochondria fission 1 protein OS=Yarrowia lipolytica (strain CLIB 122 / E 150) GN=FIS1 PE=3 SV=2

MKKEDYLPNLVDIESPLSDEELYVLSQQYNNEGDFVSVQTRFNYAWGLIKSRKVEDQQLG

VQILAQVYKDTPSRRRECLYYLAIGSYKLGEYTDARKYCDLLLQIEPDDPQSAKLRQIIE

DKLAKEGMIGIAIVGGVIAVGAAVLGAVLSQKKR

>sp|Q6CGG3|FKBP2_YARLI FK506-binding protein 2 OS=Yarrowia lipolytica (strain CLIB 122 / E 150) GN=FPR2 PE=3 SV=1

MARIIVLIVAFMALIAGVFATEEKLAKLQIGILKKISPEECTQKARKGDTVSVHYTGKLE

DGTVFDSSVERGQPIQFPLGTGRVIPGWDQGILGMCVGEKRKLTIPPHLAYGKQGAGRVI

PPDSTLIFTTELVSIDNDGDRDEL

>sp|Q6C2J1|GEM1_YARLI Mitochondrial Rho GTPase 1 OS=Yarrowia lipolytica (strain CLIB 122 / E 150) GN=GEM1 PE=3 SV=1

MTNDVIRIVVCGDEGVGKSSLITSLIKDTYVPNIQKLLPPITIPKGFSSSPDAPLSTVIV

DTQFSNSPAEAEHLHREIRQANVIWLVYSDHYSCERVSIFWLPYFRNLGVNLPIVLCANV

FDDVDSWNSRDSERIISDEMIPILREFKEIESCIRVSAKLNHNINQAFYLCQKAVMHPIA

PLFDAKEGKLKPNAVAALQRVFFLSDRDQDGYLSDQEMLELQVKCFGRSFDATDLIQIRA

QLAKINPALATERGVSEEGFITLNRLYADKGRHETTWGILRTFHYTDYLSLSDQFLYPKL

DVPENSSVELSPEGYRFLVDLFLLFDKDNDGGLNDSELKTLFKPTPGIPQKWLDFNFPYT

TVHDEQGSITLQGWLALWSMTTFLDYKTTMAYLAYLGFEGDNSKKRFSGSSVTVAMTTAA

AAAARLTAFKVTKPKKRRSRPRPYYRATPNDRSVFNCFVLGSHMSGKTSLLEAFLNRPLM

TDIYKPTIRPVSVVNSVEMTGGKQCYMVMEELGQQEAAVLSNAARLEECDVICYTYDSSD

PNSFSYIDGLRRKYPVLDTLPCVFVALKADNDRQQQRFDLQPDEYTKQIRIAAPLHVSSK

WPSSVTELFIQLAEAAQQPGRGLPNQDPEEETNTIMPFALAGGATVLLAAAVAWIFKNVR

VAGRE

>sp|Q6C555|GET1_YARLI Golgi to ER traffic protein 1 OS=Yarrowia lipolytica (strain CLIB 122 / E 150) GN=GET1 PE=3 SV=1

MDEAIIVDAEFVAPVGTTAGEFVPIDRAPAAGLLLLVAFVVLYAKVISKLGKPAIQEFLW

EIITRIVPSKQLRRRKEAQLRAIEVHTQRSNTSSQDQFAKWAKLDREYGKLKVEIEDINN

LLTASKARFFTIISSAIFLSTTGMKMFLRIKHRKAAIFWLPKNAFPYPIEYILSFSSAPL

GSVSVSAWLMICDAAMDLIVTIFVALVVGVIGMLRSNKVKPKTA

>sp|Q6C0U0|GMT_YARLI GDP-mannose transporter OS=Yarrowia lipolytica (strain CLIB 122 / E 150) GN=VRG4 PE=3 SV=1

MEKSITNSPVLSILSYCAASILMTVTNKYVLSGTSFNLNLALLAVQSIVCLTAISIGKSF

GLCKFRSFNADEAKKWFPIALLLVVMIYTSSKALQFLSIPVYTIFKNLTIILIAYGEVLW

FGGSVTSMALASFVLMVLSSVIAAWSDISGAIAVSGSATTTVTALNIGYFWMMSNCFASA

AFVLYMRKRIKLTNFGDFDTTFYNNLLSIPVLLIASLLFEDWSPANLAVNFPPESRNLIF

FSMVVSGLMSIGISYCSAWCVRVTSSTTYSMVGALNKLPLALSGIVFFGTPATFSSVSAI

FVGFVAGIVYAVAQIQKKKAEAALPK

>sp|Q6CAB8|GPI10_YARLI GPI mannosyltransferase 3 OS=Yarrowia lipolytica (strain CLIB 122 / E 150) GN=GPI10 PE=3 SV=1

MMQHKEIKHPYQGIKYTVLVVIAAFRVANALTTKTFFQPDEYWQSLEPAHRLAYGYGYLT

WEWHEGLRSSLPPLVGAGIYKALQLLGLDDPRIVRIAPKIVMALFASAGDVYTWKLSARL

QGPAEAPWALFVSLLSAFNWFFLTRTFSNSAEMVLTAVALNYWSFNGKEVNFKRLSVALF

IGAISCVLRPTNAILWAVLGLHLVLTTTAKMRVLWLAVRNVALVFAATYYIDYLYYGEPV

FPLLNFLKFNLLQSLAHFYGTSPTLYYFYEALPLLTVGWLPLTLWGLWINRSQVLVKAAL

AVVVAFSLIRHKEVRFIYPLVPILHMAAAEAITQTPKKLRKWLVWSLALVNILVAGYFSQ

VHQRGVVDVVEYLSTEPQVTSVGFLMPCHSTPYQSHLHRDIPVWFLTCEPPIGFTAEEQM

TYRDQADQFYDDPLQFVQTQFPESVAVSAREARTPLFHPSHLAIFESLEKKSPEVIEHLV

ELGYKRGKTFFNSHFHDDWRREGDVVVYNL

>sp|Q6C741|GPI11_YARLI Glycosylphosphatidylinositol anchor biosynthesis protein 11 OS=Yarrowia lipolytica (strain CLIB 122 / E 150) GN=GPI11 PE=3 SV=1

MATRKAAKTKANGPAHVPSGPSQVLAVVYGTLLLVYLRFIFSAGITHDPAKVMTQALPGL

LLLHMGYCVVVLGNKPGRKIGTDVSTAMIAAALSVFFSVIIFGLLVLFGAPAISLVHNTF

VCAMHMSILAVLPLFFTYHLDSKVWADIIAMRRPLDHVYAASVCTLIGAWLGAIPIPYDW

DRPWQQWPITILAGAYLGYFVGTLGGIALELTKSLCSKTKKTE

>sp|Q6C216|GPI18_YARLI GPI mannosyltransferase 2 OS=Yarrowia lipolytica (strain CLIB 122 / E 150) GN=GPI18 PE=3 SV=1

MKHFTTLIVVFVAIKAYLVALALVVPRQYDTSSTLLFPNNRFLSRLVIWDSVFFVSSAER

SHLYEHEWAFSWMWSRALGLAGSRDAIAYTAIAVSSLSHLLAALMLRKLTESVFHNKRFA

ETTALMYILSPAGIFLVAGYTESLFALLSFTGLYLRQRGQYPLAGAVLGASCLLRGNGLL

WGIPFLFDLASAIKHNQFNRGVSVVIGGSLVGAVFLYTQYLPWSIFCPERDEWCNYYIPS

IYGYVQQRYWNVGFLRYWTANNIPNFLFAAPVLYLMYQSMSTNPSLVPFYTVHAIMGLAC

VFMWHVQIITRISTCLPTLYWYMAKLAQGYNGHYVVRYIFVWITFQVVMWGAYLPPA

>sp|Q6C7Q6|GPI7_YARLI GPI ethanolamine phosphate transferase 2 OS=Yarrowia lipolytica (strain CLIB 122 / E 150) GN=LAS21 PE=3 SV=1

MLWKRWTLAVTIVVLQLAAVLLFARGFLPSRVLLPGYTESRVSTEAPFQKAIIMVVDAFR

SDFAFSDQSNCPQLHKRINSGGAIPFTAHSTPPTVTLPRIKGLTTGSTPNFLDAVLNIAE

SDNSSTLANQDSWLAQASRDGRKIHMFGDDTWIKLFPGMFDDCEGTASFFVSDYTEVDNN

VTRHIDTQLDQKTEWDVLILHYLGLDHIGHKTGPESPFMPAKQKEMDDIFDKLYNSCDDD

TVLILLGDHGMNEVGNHGGSSAGETSAAMVFASPKFETAQLTETAETSPLPWTDTYKYHS

RMDQTDLVPTLTALLGLNTPKNNLGVLVSQMLGLWSPEDQLNVLKNNADQMVQILQGQAS

RESDAKEVYELYDTLNSNPSVKDYYNFLYEAQSYLTHASSNYNTNDMLGGIGLGLLSTIL

ALTVFSALTLAVQGLKRLYLIILLVYFISVFGSSTVEEEHQIWYWITSGWMAFLYISGSR

NKFGDGFNWMFVQVFVRMMISWNQTGQKFTKKDDIVTWLSKDGNHPVLWILILVTYGVAF

NKVWRYGFSKVESKLAFLLTLITTFASVGFKITQAWEAGEVVPAPLLYLMGLPGTLNEVN

ARMAGLARFAFSTIAAGSLYRVLSLAGTDKVNLIRDLHAFLTLFLITQSRIQNIPLFMVY

YFLEIFLRKATNRSFIFSSRDIYQTEALFQKLVLVLSVSTLLLEQVSFFSMGNSNSMASI

DLSNAYNGVTLYQIEFVGVLTFVSNWIGPLYWSTAGLSFLLEDHVRNAIFAKIAEKNNDV

KLTTKLVQQALTLRVYVVLAFSSVAISAVMITCFFLREHLFIWTVFSPKLLYQFVWTVLQ

FALVDVILSSIFVVLVYRSV

>sp|P41943|GPR1_YARLI Glyoxylate pathway regulator OS=Yarrowia lipolytica (strain CLIB 122 / E 150) GN=GPR1 PE=1 SV=3

MNTEIPDLEKQQIDHNSGSDDPQPIHDDMAPVSRIRSSGPNHEYIHIADQKFHRDDFYRA

FGGTLNPGGAPQPSRKFGNPAPLGLSAFALTTLVFSLCTVQARGVPNPSIAVGLALFYGG

VCQFAAGMWEFVQENTFGAAALTSYGGFWMSWAAIEMNAFGIKDSYNDPIEVQNAVGIYL

FGWFIFTLMLTLCTLKSTVAFFGLFFMLMMTFLVLACANVTQHHGTAIGGGWLGIITAFF

GFYNAYAGLANPGNSYIVPVPLDMPFVKKD

>sp|Q99170|GRP78_YARLI 78 kDa glucose-regulated protein homolog OS=Yarrowia lipolytica (strain CLIB 122 / E 150) GN=KAR2 PE=3 SV=1

MKFSMPSWGVVFYALLVCLLPFLSKAGVQADDVDSYGTVIGIDLGTTYSCVGVMKGGRVE

ILANDQGSRITPSYVAFTEDERLVGDAAKNQAANNPFNTIFDIKRLIGLKYKDESVQRDI

KHFPYKVKNKDGKPVVVVETKGEKKTYTPEEISAMILTKMKDIAQDYLGKKVTHAVVTVP

AYFNDAQRQATKDAGIIAGLNVLRIVNEPTAAAIAYGLDHTDDEKQIVVYDLGGGTFDVS

LLSIESGVFEVLATAGDTHLGGEDFDYRVIKHFVKQYNKKHDVDITKNAKTIGKLKREVE

KAKRTLSSQMSTRIEIESFFDGEDFSETLTRAKFEELNIDLFKRTLKPVEQVLKDSGVKK

EDVHDIVLVGGSTRIPKVQELLEKFFDGKKASKGINPDEAVAYGAAVQAGVLSGEDGVED

IVLLDVNPLTLGIETTGGVMTKLINRNTNIPTKKSQIFSTAVDNQSTVLIQVFEGERTMS

KDNNLLGKFELKGIPPAPRGVPQIEVTFELDANGILRVTAHDKGTGKSETITITNDKGRL

SKDEIERMVEEAERFAEEDALIRETIEAKNSLENYAHSLRNQVADKSGLGGKISADDKEA

LNDAVTETLEWLEANSVSATKEDFEEKKEALSAIAYPITSKIYEGGEGGDESNDGGFYAD

DDEAPFHDEL

>sp|Q6CAW6|GWT1_YARLI GPI-anchored wall transfer protein 1 OS=Yarrowia lipolytica (strain CLIB 122 / E 150) GN=GWT1 PE=3 SV=1

MSSQKLLKEEHVSGLTGGSIGEIYVVTCVNLTAYVAWALLRKRYGDHSPWDVDFVIFDFL

LNWLGLLLSVTIYSNQPLLLNALIIVPAGVWYIWGRRDRVKKRKELRPDFQQEKDKEVKR

ADKEMPFLSVYRGSMMVITCIAILAVDFNIFPRRFAKVETWGTSMMDLGVGSFVFSMGVV

SKPRTDEPFGPQMKKSLKHAFPVLVLGFIRLISVKSLDYQEHVSEYGVHWNFFFTLGFLP

PFVTLVGGLFKKTKIPLMGQSVIIALAYDVLLSVTSLKEYILTAPRVDIFSQNKEGIFSF

IGYLAIFLAGQAVGTVILRTKLPEPTPANSKRTPHNLRYRQIIKYLTISSILFHVARLYY

DGTIEINVSRRLVNMPYYLWVCAYNTFFLGCYAAIEVILVPIRASQPATPRVPLTLDAVN

YNGLVIFLLANIGTGLINMSVNTLEASPAKTMVILVAYCAALSGISLVLYKKEIRLKL

>sp|Q6C4X5|HUT1_YARLI UDP-galactose transporter homolog 1 OS=Yarrowia lipolytica (strain CLIB 122 / E 150) GN=HUT1 PE=3 SV=1

MSELRKRNQAAIADLSENIEVTETVELTEKKPETTKSTKGHIIDLIICVSGIYASFLTWA

VLQERIATTPYGPDNKIFRASLVINTVQSFLAAAVGYAYLQYKQSRRAAKGLKKNTTVFD

SMYTLKQLSLVALSQSLASPLSYTALKYVDYLTSILAKSCKLIPLMALQVTLYRRKFPAY

KYAVVVLVTIGVSMFTIFHAAPKKASGAGSEHQLYGLGLLGISMLLDGLTNSTQDQIFRK

NADITGPHVMCGLNLLTGVFTTVSLLTFSRPQLDTAIAFIRLHPEIMRDIVLFGLCGAVG

QVFIFQTLEKFGSVVLVTVNVTRKMFSMLLSVVWFNHRLTLGQWAGVAAVFGGIGFEAWM

KMKKN

>sp|Q6CAP3|IML1_YARLI Vacuolar membrane-associated protein IML1 OS=Yarrowia lipolytica (strain CLIB 122 / E 150) GN=IML1 PE=3 SV=1

MASCATVCLPTCGTRLSGHSRSRSVWKLVSYITLLHFILKVFLVFLTSFHLLTQLSIKSG

NLLSSLGVAARSQVQIKLRKKEQVEADLVEICFKETYLTRADMWQITTMLRESCVYHQKR

IVFCDVIRCWVDSIFKNGRKVFSAYIGPNTNIVFRSESSRMIIIIQLSQETWHFEETGEI

IFHRMVNSLLPDIFKQWEENEHHHTVTIVLFTSVSMDSHAVKLKRGEKAKDVQDFYRVVC

DQVQLSQWEQIMVRLRFEFQNFAKEVLTESHAKSHTEIRGRILPSVKSNILGAITLAASL

VHSPAIDRDLRRTNVEVIVVSPGSGVYEVEYDALYRASEKISSTEVGVDIICMSKAPLHV

TPLFLYKPKKCSNQLEYCVPSWIDISYYGDSDFFTNQWVPRCKIYEIQMMGVMENELSAI

TIDYLNKPSGKKPLSEYCREYDASLFGPIAVELSVEDAASVVSKMRSDTFPKLPVKQLLS

SKGSISSLHAPTAVTSKTPPPKASVKVADVRPSPAASKPRVSALTSLLAFGVRAEKSAPA

SPALSAVNTLSSIQSMDLRRDNDSVKRDTESIVTDDTVHNTKAEPRITEIAPIAPSLPFA

PNLKPKKSTSTLSQSPTYNRIAATPDQEVERRSSLSQKVQMGLSGSPLDHRGKVAKAASA

NAKSINTETTPEKRNMMWRNIRTPSNISQDEMLDILTYSRWRTVYPQKTKRRSAKWKLLA

SPAALPLRTNIFPTVAELQTEYRFQVYDVSLDTDMENVLGIHGLFREMVSVRLCKGFQLA

IGSRVRHVESQRSDGRPSAITTDIGKDALGAVVYLTTGDQVHRISCDYSGMMNVQIYHAI

DKVDIEEPVFDIYARRTYSDKFKHFIFPPFDTSSKRLNWNLYDHSLAGYDTAGSAATQTR

LNQLRVVLIPSFLSRSKNLGHDDSSEVYNAEEVRLDGLRRILGGIYRHSISRDEDKERAK

IAPNLKFYTGELEDFLFQIVESNPAELAGGRDSLFMKRNQRFDKNIKLAQLATELQAPKG

GIRFFDRRWHWKSYPHCFIGQDFVEWLIDNYSDIDTPDEAVAYGNELMKKDFFVHVEDRH

AFLDGHYFYQLKSEYATEATDSKPAEEKSGWFNSKRSMASVSSEKSLGRFKGSRNQSVQS

FGDFLSLSRVTSRSSNDQKEEEINPITVEISKSVRVDLDPSKKSYRPETVLIHHDRIHNP

RKCFHLRFEWANTTPKFIEDYISGLTRTCERYGLKLVELPILEVSALLEHNPFASEITLD

MPTLDQLSGLPPHVLEYYASDPHAIAREILHHFGFVIDTLSMSDWKLAEVQIRNSWGVPT

YKYSQWVEKNGLLIAQVVGDQIIMIPNNLQLNRQSGAQSNAIQLQSEAQGIILEMQYLMC

RSEKVRDMLGKVNLSPEASPGTMTPGEEGSKNGTPKLAIEAKMEGRVESPQVEWREDVEI

APEVEHKGGADGDENDEEGDEKSEEGDKGESENENAGMGMEQDGENENENENENENSKDN

KANETTSEVKEVPAGVEIEHKEHVEEDKSDGPEPKKEDKGKEVEDDK

>sp|Q6C994|KAR5_YARLI Nuclear fusion protein KAR5 OS=Yarrowia lipolytica (strain CLIB 122 / E 150) GN=KAR5 PE=3 SV=1

MSLSYSFNTTIFTENRDLVAALQPQTNCARTALTLIVSDCGKLSSFNEDQQLRVSLAVGL

AVCEFKAAQVTYPDACNNIEDWMSTSACTQQLVSSPQWWTTYHGCYNSVKQICHMHEASR

ECDRALKTHSQIVDMQEKLHTKMDQYWELVETMSDHRDAVLDYWNDTFDFMSETLAHMKE

TSVSLNAVYRDNFAQAQEHFQMLSENLQEARVQMENLGWAAQDAVVSLSKSTLAEQSLVS

ERLKNDASSLHKLLVLAHQDTTESFETQLQQSLTILVESSDNVLLNHVQQVSSRLSALMS

DLEESQKKNMDMQHQLQQKVRTINDDIEGFTDTVKQGLEASHSLLNLVKSKIQLVNGVVS

IFSRPVRSAFQLASFIIMIRAAFIGGIYTSIGLVMGSMLGVLVMQQV

>sp|Q6CFP3|KEX1_YARLI Pheromone-processing carboxypeptidase KEX1 OS=Yarrowia lipolytica (strain CLIB 122 / E 150) GN=KEX1 PE=3 SV=1

MKLSWSLFCGLASLALSQFDEAPPSQSDYFVRHIPGLDSVDNYTMHSGNILTDAAHNGNL

FFWLVEAQYKITERPKTIVWFNGGPGCSSMDGALLEVGPFRIVDDKLRVDPNKGSWHKYA

NVLFVDQPYGTGYSYSDTDSYLTGLGQVGDEMDSFMTQFLKLFPERAHDDFYLAGESYAG

QYIPYIATKLQQTRTVDLKGLLIGNGWMDPANQYYQYVPYALDYGVIEKTEEHVKDLKEL

TDTCERAINIAKDKNNGRLPVHIRACEDIMNGIVELSRNERSAPESEGICVNYYDVSKED

KWPSCGMNWPEILPYVTDWLRQDATVQALNVNNDKQESWQECNGAVGSRMRQGNDDAAVY

LLPDLLESMEILFFNGDRDLICNHYGNERMIEQLEWNGKKGWTEGLELDDWVVDGVSKGK

KQSDRNLTYVRIYNASHMVPYDEPEACLTMLNDFIGVSKALSDLSGNKPGRGSENPSDLD

DQKSGDQKSDDDSSSDDDDDAEHDKKIASDAMWKAYYQAGFTALIVVLIILGLAGFLFWR

KNRGHIYQEETSLLGSCFGGISRWRNSSGGPLSNQQGTFDSRQRLMEPGEYYDLGEIAEE

DEDAEELVIRRPEV

>sp|Q6C9M8|KMO_YARLI Kynurenine 3-monooxygenase OS=Yarrowia lipolytica (strain CLIB 122 / E 150) GN=BNA4 PE=3 SV=1

MHEVTVVGAGLVGCLAALAFADRGHKVALYDARPDLRSEAELKNASLRSINLAVSARGIE

ALRSVDTKMAERVLADIIPMYGRMIHDLQGGQHAQAYGLWGECINSIDRAQLNRTMLDVI

EDNANITFFPEHKLTNISLSRKDKKYQRPTSTFETKEGEERVVESDYIIGADGAFSKTRD

RLQRYVRMNYAQQYIDCVYLELKIPKADGPDPFSISPNHLHIWPRHKYMLIALANGDGSF

TSTLFAPPALMEQVCESQNTFISFFKEQFPDAYELMGESQILESYENNPRSPLVSLKCSP

YNHKGECLLVGDAAHCMVPFYGQGMNAGFEDIRVLMEILDEKKWNVEEAFNTYTERRHKD

LVAIVDLAMRNYVEMSHSVVSLPYLIRKKVDGVLGRVFSSAWVPLYSMVSFRADIPYSKA

LSRSARQDRIIGNIVNWTSFAGLVGMGALFYYKGRHLFGRLFE

>sp|Q6C427|LCL3_YARLI Probable endonuclease LCL3 OS=Yarrowia lipolytica (strain CLIB 122 / E 150) GN=LCL3 PE=3 SV=1

MPEDSNKASNTARVVFYTSILTGGILSSFYVYSRYFRRFTCTAEVPKKIYRGRTLFGRVT

SVGDGDNFHFYHTPGGRLAGWGWLRPYPETNKRGLGKETLHIRLYGVDAPERPHFGRQGQ

PYGDEALEWLRSYILGRNVRVKLFSPDQYGRIVGGAKVWKLTGRKDVSTEMLKNGWGVKY

EGKMGAEFNGKGKLFQKLEDHARKKKIGMFQQKGKIVTPGQYKKDE

>sp|Q6C8H7|LPE10_YARLI Mitochondrial inner membrane magnesium transporter LPE10 OS=Yarrowia lipolytica (strain CLIB 122 / E 150) GN=LPE10 PE=3 SV=1

MHIKISTDFAIQNLHCTTMIRPLLRLCGQRTAATPFVSFFRPPKKPLSGISFARHYSPTK

KPPSEVPTPTPGNYLVPITGPPGDTPRDTDLLIKSLTHKSLLPENNLVRCTVFDSDGNVT

VASGEFKRTELLNKHGLLPRDLRKLDTGVNSIVPTILVRDNSILINLLHIRALIKADKVL

LFDVFGSTDSKTQSLFMYDLGHKLKKSNKTMGSLPYEMRALEAIFISVIAALDAEMKVHT

TVINGILSELEQDIDREKLRHLLIQSKKLSAFLQKATLIRDVIDELLDTDEDLAGLYLTE

KKAGHPRAIDDHSEVEMLLETYYKHCDEIVQTVGNLVSNIRNTEEIVNIILDANRNALMH

LDLKFQIGALGLAGGTFIASLYGMNLKNFIEESYWGFLGVTGVASLLTVWIIAHFLKSLR

QVQRVTMTSDKKKAMKKKDTVAEKRRNHLRNWLTK

>sp|Q6CDE6|M28P1_YARLI Probable zinc metalloprotease YALI0C01133g OS=Yarrowia lipolytica (strain CLIB 122 / E 150) GN=YALI0C01133g PE=3 SV=1

MDRQSLRTTLRAMDASNENGSAKGAKKTTIGSFVRWTFGFNSVPLTTLVTITTVLLGLLV

YVSTSVNPPDVTEAQKPLLNYAWAQLGEISRYPHPYFSHDNDRVRQHILKEVYTLAGREH

FEGAQIEVDDSQTDIFIQKEDVFDKSAPPGKLTYFEGNNVVVRLSSKNSDKSLGAILLSA

HFDSVPSSFGVTDDGAGIATMLAVLKHALAQNEGPKRDIIFNFNNNEEFGLLGAEAFMHH

PWAQNVSAFINLEGTGAGGKAILFRASDYGVASHYSAAEMPFASSVYQEGFSNGFIHSQT

DYKVYTEGGLRGLDIAFYKPRALYHTRRDNIAETTKNALNHMLVNTIDVTQSMTEADSFD

HADQPAVFSDIGGYLFIILPLQYIFVISCLTLAVGPIFVGFLFLLVLRKQINAGTSETIL

GGWLRSIVSVLVSVVATYFVVETLHLGNELYVVRSFYTPLFAGLGTFIFVNYVLLGFFHF

VRPVCDQKLIILLELSVVLWVLLLLSVIHEATHKATGEYHFLILYIVVATASILGLFGHL

VTSTETSTFVEGPEDEEDTVDASEATETSPLLPEASPDNAAPSIHGAVDPENQQEDKTLQ

KIAVSMGYDWSIQFLLVVPITFFVTFGLAASLLDGLHQTPLESEKSADFVYTTITAMSVL

VGITFLPFVHKLQVFVPIVVVGVAVTASFVHILSPPFSSNAPAKMRFVQNINLDEGTSYA

NVLGRQDVLQEVLEYIPSTDTFKPNCSSRGDGVEICNYIAPRPWLIDGEKVSSDGDFAGL

PTNLLDVKVVPVNETSSGPFDRFDGRFKISALNNRGCVLRFNTTRFKSGDVETGVSPVKM

VTLRHNRVGKTGITTGSGSRFSMFGWTRDPKTGKDEFRSFMHGVDDVTLHKLDWEDPEYD

IQLSWIPRWYEVGDPEDEGNEALKNRLGVTVTCSWAEYLDPSSIVDEQGRRRTMDKIPAF

TELNNFSPAWSIWSNQGRGLVEVSKYVEL

>sp|Q6C0Z3|MCD4_YARLI GPI ethanolamine phosphate transferase 1 OS=Yarrowia lipolytica (strain CLIB 122 / E 150) GN=MCD4 PE=3 SV=1

MISLNKKLVLLVGVIFHVAFMWSIFDIYFVSPLIHGMKHHQSTATPPAKRLFLIVGDGLR

ADKAFEKVRHPTTGESEYLAPFLRSKVMSDATFGISHTRMPTESRPGHVALIAGFYEDVS

AVTKGWKENPVDFDSVFNQSRHTYSLGSPDILPMFKHGAEDQSRIDAIMYGHDFEDFTKG

SIELDAFVFDHLDEIFDKSKTNKTLDDQLRSDKTVFFLHLLGIDTAGHSYRPYSAEYYDN

IKYIDENIEKLVDKVNKFYNDDEQTAWVFTADHGMSDWGSHGDGHPDNTRTPLIAWGAGV

NKPIPAFEDKGNHDDYSEVWDLPVKRNDVNQADIASLMSYLVGLNYPSNSVGELPLAFVN

ATSETKALAIRNNALALVEQYLVKEEQQKGSQIIFKPYPPLSDAGKTIDERLAHIDELIA

QGLDQESIVASEELMTYAITGLKYLQTYNWLFLRTLVTIGFFGWIAVAFCSYLLAFVVQS

DKPFTTSLPLKGVAYVALAILSGFFVFQKSPLHYHLYAVFPVVFWEAVLQRRTAVAEGIS

ILARRSTSKAPALAAILDIGLSLVLLEAIVYGYFHREIFSVCFGLATLWPFVHNFTVAKR

EWPTTLAWVVMCAIMSSFTLLEVVKVESIEQILLSGALMLVIGLVFTIHLQRKLALAAST

VCVLFAQILLVVATMYFTRESVESLTARNGLPLFSQVGGWISLLLSLAVPFLHFLGSDAK

DYRLRLLIIFLAFGPTFVILTISWEGFFYVCFFAILVIWIELETQMRDARVTPQTRADLT

PGDFRMALFTFFMSQIGFFGIGNIASISSFSLDSVYRLIPVFDPFSMGALLMFKILVPFA

VLSACLGILNLKLGVPPSALFSMVLCVSDILTLNFFYLVVDEGSWLDIGTGISHYCIASG

LSLFMMVLEYLSGVLVAGVTIAPHVSKIKKDM

>sp|Q6CGU8|MCH1_YARLI Probable transporter MCH1 OS=Yarrowia lipolytica (strain CLIB 122 / E 150) GN=MCH1 PE=3 SV=1

MVDERTPLKAVTTREDAPTAVMRPKPVGKSIFALSILGTLSAASISLFSMYGQTLQHKLG

FTQVQVNSVSISSLLGMYLLMPVIGYLGDTYGSNYLALFSWITFPASYSIASGIFSTAAD

WHRETGEALPRAAPEMALCFFFIGASTSCMYYASLKASAHSMSVYFAEDGKLQTYTPGYA

IWGPVAAFGLSSLWQSQLLRAVFITGKNVNVAGIFLFFAGLYAVICGIIFFSCRTAESMA

SELRKKAEASTDCNCDGPGHEGATLKEFFTDKTAWLFLLCFVFIGGPFEMFQNNMGAILD

TVTVENADSPSFSTHVSLFATFSTVSRLVVGFSSEAMESHVSRPVLLSVIALVAACIHLM

VPSGIFTVFDNAKYFSVVTIVNGFSYGSSFTLVPTIVTKVWGIANLGTIWGSFILALAVG

SLGYGLLFAKVYDAASEVGVGSMSQVCSGVHCYGLTFVITGTGLAFAAAAVFFIWVFMWK

KRGIHM

>sp|Q6CHP1|MKAR_YARLI Very-long-chain 3-oxoacyl-CoA reductase OS=Yarrowia lipolytica (strain CLIB 122 / E 150) GN=YALI0A06787g PE=3 SV=1

MVYVNAKNYFCDSIINNTDRVLSALIKYHGLSIIAVFLLAIGLFHVALKVVSYVAVLLDV

FVLPPTNYLPYGSQRGAWAVVTGASDGIGKEYARQLGLRGFNVFLISRTESKLRELAQEI

AEKSKVETKFLAIDVSTDSPQNYKDIETVLETIPSVSILINNVGLSHSIPTPFLETPPAE

LHNIIAINNLATLKITQLIAPKIVESVKEARATKKFQKGLILTMGSFGGLLPTPLLATYS

GSKAFLQHWSNALAVELAPEHVDVELVVSYLVTSAMSKVRKTSALIPNPKQFVTATLSSV

GRAGGAQEKFATSTPYWSHALLHWWIAQTVGVFSKLVAGFNYKMHVDIRKRALKKQARQA

AGGVADPKNTTAAREGYATESLKNETLKH

>sp|Q6CAP9|MMM11_YARLI Maintenance of mitochondrial morphology protein 1-1 OS=Yarrowia lipolytica (strain CLIB 122 / E 150) GN=MMM1-1 PE=3 SV=1

MSQFVLPAVASEGIINWPFLTGFMLGQFSVGLVLLIFVRFFIFSDQTEPDINTQRRTAKV

LPTGNPSTDAILEKTYYNTKTHQPESLDWFSVLVAQALYQLRDEVRGNDEVLERLNEILK

SDKLPGFLDTINVVDLDIGDAFPQFGACKVNKDESGDLEAEIKVSLEDTIKLGVETKMLL

NFPIPKFASVPVSLSVSLVKFSGTLTVAIRSAFNSGEANRVLVSFAPDYELQWKIESSVG

STQKLQNAEKISRLIESRIRRWFKDRCVYPEYQQFELPKLWRKTSAPPPSAGAGTGTSTG

VPPSPFPQPANPSSVPKPLELPGGFPHRNMSMSSQRPNINNPKFIRSMSVNTRPTYSSSF

YEMQGTAGSSSGSAVADEAYRSLQTSPRR

>sp|Q6CA06|MMM12_YARLI Maintenance of mitochondrial morphology protein 1-2 OS=Yarrowia lipolytica (strain CLIB 122 / E 150) GN=MMM1-2 PE=3 SV=1

MVIPAELIQKALKQQSGWGFTEGLVLGQLSVIITVIIILKFVIFAENKSPKKGNDMTAVS

AKDKEAEHVAMGGQTANGVKTSGVRRNKSSTNLRNRLATGAAGASISRPGSSRVSMVRST

SGAVPVLGQNGGATPRGMAGSSVAGSTSNLAVPVPTTPTIAEGIEPENDSTLQDDSAIID

LDLDLDLSPVDEILHKTCYQLSSHAPESLDWFNVLVAQIITQLRFDAKANNNLNLLNSLD

AAFNSKRPDFIDRINVTEINLGDDYPILSNCKITHKGTGPAGSGAASGNNMPNNAEYDDS

RLEAQMDIDLSDTITLGIETRLNLNQPKILSPFLSLSTSLPVSLSVTIVRFTARLNISLY

QQIEQTEEDEKDKRDTILSINFEPDYKLQLSVKSLVGSRSRLQNVPTLESLVDSKIQKWF

RDHYVEPHQMIFILPSLWPRKKRSATAGAAGTATGADTASGSS

>sp|Q6C2P2|MRS2_YARLI Mitochondrial inner membrane magnesium transporter MRS2 OS=Yarrowia lipolytica (strain CLIB 122 / E 150) GN=MRS2 PE=3 SV=1

MDEEEHKDHQEEEDPHHFAFSDNYSEAVLGGEISATVFDVTGKVVHVAHKMTKYEFLLEH

GLYPRDLRNIDPSPVSIIPSILARGRKGAGRCILVNLLHIKALILHDKVLIFDTHSKNKS

DTHRLGMFLYELENKLKPTINPEKMHTDMTVLPFELRVLEAILVNVMTTLDGELQVHLKT

LNEILVGLEDHVDREQLKELLIGNKNVSRFYQKAVLIRDVLEELLESDDDLQQLYLGTHP

KEGLAEVELLIESYCKQADEIVQQASNVRSHIRSTEEIVNIIVDANRNALMLLELKVTIV

TVGFAVGAFVAALYGMNLENFIEETNEGMVLVVGVACLGGLLVTWFNLTKLHKTQKIAMF

SNAATHTASKPYMLQWIIGLLRRKRTKSRFDNIDMSRPKMKGKKSNILHQLTKMTGKRR

>sp|Q6CEA4|MTC6_YARLI Maintenance of telomere capping protein 6 OS=Yarrowia lipolytica (strain CLIB 122 / E 150) GN=MTC6 PE=3 SV=1

MVWLWTLPWTFILVALIPLATAKSPDVHHSLQQLKKRDDDSLRDENCQAWIAPVWPGMSH

MMVAATRAERDVSYNVSIDQLTYKGAELTPLVFNKYGYTQDGIFKSMDLLKSGMTSLGLD

VYWNNKEQYWQLCPVIFPSSAEPGTPVLLQRRLTTDIVTCDQNATLSYFLAALSNHVNVT

DNNLDVDLISLKLNLRTVETPIQSNSSSQRQKRQFRETSTSVTQLASSVLSSLLNASKTT

RMESTGSFSNSDSNSSSNSSRMSNSTESTRTTSSTESASSTSTTPSTYTTGPQMLGNIVG

SSYIGPRLYTPKDLHRDREAEPPRTYDYQGLSNRGFPLAHYILLEMKARVMVSISQNDQP

DGYGWGSADQDVIFTKQALLGSSDPENTNENIAYENLQECIRLDPQFLQSINLTGAASWR

TYSSTQQDPISNVSFQEFINCGLSPSINNTLAPGETLQGLMTESLWSWAEDEPSKCHNES

VTRTQDRNTGLVAWNCAVLMPDGWHVANCYDTHYPLCRQGELAYNWAVGTQKVNYFDTAQ

EKNICPPGYTFSLPRTALQNIAAKITVRGVSGKTRVYKGGDTTDPDSYDTIDYGNTAFPV

WIDLNSISQEDCWVSGGPTARCPYRTVQSHSVSVALLTVASAVCAGVAAAIILLQMDTQP

IKKNSGRWRRLMSNFKKSEYESVPA

>sp|Q6CA86|NCB5R_YARLI NADH-cytochrome b5 reductase 1 OS=Yarrowia lipolytica (strain CLIB 122 / E 150) GN=CBR1 PE=3 SV=1

MRSSSSRQPQMQSYYAIATVWALIIGAATYYFFSNSKPKAVLQRGDTAFKEFPLIQKTVL

SHNSAIYRFGLPRPSHVLGLPIGQHVSLSANIGGKEVLRSYTPTSSDLYDKGYFDILIKT

YPQGNISKYVSELAIGDTMKVRGPKGNFVYNHGLVESFGMVCGGTGITPMYQILRHIAAD

PADNTKVNLVYANVNHDDILLKKELDAIAAENDNIKIHYVLNNAPEDWTGSVGFVTKEIL

EKHCPPPGPNTKLLLCGPPPMISALKKASVELGYEKARPVSKLEDQVFAF

>sp|F2Z699|NDH2_YARLI External alternative NADH-ubiquinone oxidoreductase, mitochondrial OS=Yarrowia lipolytica (strain CLIB 122 / E 150) GN=NDH2 PE=1 SV=1

MLRLRPAVRAVSVARSVALTRSLHVSVAKFNKIEGTAPAGLPKEVKQTAGHQGHHQEIPK

PDENHPRRKKFHFWRSLWRLTYLSAIASLGYIGYRIYVIRNPSDQLPADPSKKTLVVLGS

GWGSVSFLKKLDTSNYNVIVVSPRNYFLFTPLLPSCPTGTIEHRSIMEPIRGIIRHKQAE

CQYLEADATKIDHEKRIVTIRSAVSENSKEEVIKEIPFDYLVVGVGAMSSTFGIPGVQEN

ACFLKEIPDAQQIRRTLMDCIEKAQFEKDPEVRKRLLHTVVVGGGPTGVEFAAELQDFFE

DDLRKWIPDIRDDFKVTLVEALPNVLPSFSKKLIDYTEKTFSDEKISILTKTMVKSVDEN

VIRAEQTKGDGTKETLEMPYGTLVWATGNTVRPVVRELMSKIPAQKGSRRGLLVNEYLVV

EGTEGIWALGDCSATKYAPTAQVASQEGSYLANLLNGIAKTEDLNNEITNLEKQSEHTFD

EQERKNIFAQLESKSRKLRRSRAMLPFEYSHQGSLAYIGSDRAVADLSFNFWGIMNWSSG

GTMTYYFWRSAYVSMCFSMRNKILVCIDWMKVRVFGRDISRE

>sp|Q6CF18|NTE1_YARLI Lysophospholipase NTE1 OS=Yarrowia lipolytica (strain CLIB 122 / E 150) GN=NTE1 PE=3 SV=1

MDSLHVSSTSVLVDVVEAVETATSLVVDTAEAVATEQATPTAVISNALARSAYAAHTSLS

YLAWAFGLWFLRLIGWVCYGIPTYVLGLLGRTINISLQFSSLLLILIALVTVVVAVVRYK

YLTVYSRLPQEQPRQEPEIDMYDESDNEDEKTGFANYFDEFLSAVKIFGYLERPVFHELT

RHMQTWKLSADEMVPLDDEQGFSVVVEGTVQVFAKQSSFVQNPTSVPDSRKEDSIMFNGE

RYTLLSEIKNGAPLTSLFNILSLFTDDLQLHKNFDSAVNSPMTSASDVPNMSLSSDGSDD

LQKGEPQFGEPRLSEKPAAKLSVTVRAATDSTIAIIPAAAFRRITKKFPQATAHIVQVIL

TRFQRVTFQTGHHYFGLTPEIFQTEVNLNSHARNELPGYLREGAVKKLNQVYDASQMGGR

PKKYTVTLNKKNKGKGSRRQRFSVQLNNQGHLNSQSRMVSLDSLEAVGDHMNPGDLLTNV

PLSRQGRPVFELSSVKHKASIQNLSFSGNDDENEDTALRTALVEAIFKVLGIDRDSIQSS

IMAVKTMSNTASPMFTGATTGGSSGSLGEELRSRRTGQDSLGASHFGVGLPSERSQNSFY

ARSETSTSSVDEDSLMAAPFDTIRNDVAQYMDVVLFKKDSLLIKQDDPTPGLYYLIDGVL

EVGYTDHHKIYHDLYTVQPGGVGGYIGSILGHRSFADLRARTDVYAGFLPRAAIERMSDK

YPMVHLTMAKSLTKVLSRLLLHLDFAMEWVQVRAGQKIYKEGQEADAIYIVLNGRVRSVA

ETKGDSGIVGGESGDAKDGKSHRKNLTSIGEYGKGESVGELEVLTLTRRPSTLVAIRDAE

LAKIPRALFESLALHYPSITFEISRIVASRVRTLMEDSAPIPRRMHTFDMAAHHDSYLTI

AVVPISQDVDVSEFGRRLYNGMQAVGREACHLNHASVLNHMGRHAFNPLGKLKLSGFLDD

IEDRYQTVLYVADTPPGSSWTHTCISQADCVLLVADARSEPDIGEYERVLVKMRTTARTE

MVLIHPERYVPPGLTSAWLKPRVWVHTHHHVQMDLPRHEADVLASIRKMKRTGTLANLKN

KVQTIQEEFRSMYRPKANIYSTSSANKDDFNRLARILSGQAIGLVLGGGGARGISHIGII

KALEDSGIPIDFVGGTSIGSFIGGLYAKEYDLVPIYGRAKKFSGRVSSLWRMALDLTYPA

TSYTTGHEFNRGIWKAFGDSRIEDFWLRYFTNTTNITHSRMEIHTSGYAWRYIRASMSLA

GLLPPLTDNGSMLLDGGYVDNLPVSEMKAQGASVVFAVDVGSIDDTTPMNYGDSLSGAWV

MWNRWNPFGRHPNVPNLAEIQARLAYVSSVGALEKAKHTPGVIYMRPPIDDFATLDFAKF

LDIYRVGNKYGHKFLTELREDGKFPAIPGMENVKTKHKRTIARRNSI

>sp|Q9B6E8|NU1M_YARLI NADH-ubiquinone oxidoreductase chain 1 OS=Yarrowia lipolytica (strain CLIB 122 / E 150) GN=ND1 PE=1 SV=1

MIINIVEILIFLVCVLFSVAYLTVAERKTLAYMQRRLGPNFVGYYGLLQAFADAVKLLLK

EIVLPKESNYIILVISPLITLITALIGWVVIPLGPGITLGELNLGILFSLAIGSLGVFGS

LLSGWSSNSKYSLLGSIRSTAQLISYELILTSIFIIIIMFVSSLNITTIIETQRVVWYCI

PLLPLLLIFFIASVAETARPPFDLTESESELVAGYFTEYSGSPFVFFFLAEYSNIILISA

FNGYLLLGGYLSFNYSYLFNILFNDYSYVSFLFEGLINSSAYAIKLVFLMFSFIWVRAAF

PRFTYDNLINFCWIILLPLLFGIFLIIPSTLYIFDSFPTLI

>sp|Q9B6C8|NU2M_YARLI NADH-ubiquinone oxidoreductase chain 2 OS=Yarrowia lipolytica (strain CLIB 122 / E 150) GN=ND2 PE=1 SV=1

MLILAIISLITFVSMSKLSDNRAIIRLINIYLILVLVLDSFLYLLFLNNQTYTVMGELLI

FNSFTFYIDMLIYFIMIVISSLYGYNLYNNNLYKTLFEPKKELIILFLINILGALLIVHS

NDFITLFVAIELQSYSIYLITAIYNSSYKASKASMLYFFMGGILSILIAYSINTYYSVLN

SYTLHSLDSLIINTLDLNLILIALSLGLLFKIGIAPLHKWLISIYENTPILITIYISLIP

KISILSYLVLSNISINSLVISILAILTLLVGSVGGLLQIKIKRLLAFSGLTNAGYMMLLL

LLNNNEFSYLYYITQYSISHLAIFMIIIFSIYYINYINNQYNPIIYVNQLKGLIHDNAYL

VLSMAIVVFSFIGIPPLLGFFGKLNILMSILNNGYYFISIVLIVASLISALYYLYLLNVS

IQDKNNILINSNETVSSVLSYILSSLIILITFGFIYNSLIIDIFNVYFN

>sp|Q9B6C7|NU3M_YARLI NADH-ubiquinone oxidoreductase chain 3 OS=Yarrowia lipolytica (strain CLIB 122 / E 150) GN=ND3 PE=1 SV=1

MNTFIIFIILIPIVGFALLAVNILLAVYKPYNEKLGAFECGLTSFNQTRLAFNAAFILVA

ILFLPFDLEISTLLPYVMSIYLVSNYGFTIVLLFLLILIIGFVYEINTNALKINKHNKPN

TDSLIYKL

>sp|Q9B6D4|NU4LM_YARLI NADH-ubiquinone oxidoreductase chain 4L OS=Yarrowia lipolytica (strain CLIB 122 / E 150) GN=ND4L PE=1 SV=1

MFIGTIILVLSFLGFVFNRRNIILAFICLETMLLGINLILLRNSVLFDDISGSLFAIVII

ILAGVESAIGLSLLVSYYRLRGVINSYGI

>sp|Q9B6D6|NU4M_YARLI NADH-ubiquinone oxidoreductase chain 4 OS=Yarrowia lipolytica (strain CLIB 122 / E 150) GN=ND4 PE=1 SV=1

MFLTSILLSSLYLFNRILAWQGNVKHFYLFASNLLLLFIVVLYINFNTFSNSFQFNFELF

NSLNPFGLSNSDISNGLLFGIDGLSLTFILLTVLLIPLTLLGNWYNINFNSNLYYTLVLA

IGLVILLNFWALDYISFYILFEATLPLLFILIHIYGSSDSERASFYVLMFTLSGSLFMLL

SIVVISIVLNTTNFINHNLFVLSLDLQTIIWLGLFIAIMVKTPLFPIHVWLPVVHSESPL

AGSMILAGLILKLALYAILRLLLPLLCEAQILYTPMIYIISLLTIILTSLATLRQIDLKV

IIAYSSISHMGIAILGVCSNTSLGIYGSIVLGVAHGFVSPALFLIVGGILYDRYHIRIVN

YYKGLTTYMPQLATYIIILSFANIGTPLTGNFTGEFLSLQGGFIRNPIIGGISCISVLLA

AIYQLKLTNKLTGGISSIYMHRTNDVTIREKFIMNILIISTLIIGICPQIMYNLLYWTVN

NYIYII

>sp|Q9B6D3|NU5M_YARLI NADH-ubiquinone oxidoreductase chain 5 OS=Yarrowia lipolytica (strain CLIB 122 / E 150) GN=ND5 PE=1 SV=1

MYNAISLIIILPCISWLFPLFFGRQLGYVFVTRMTSTLIIITTLITYYYFYQLLGNNNPI

NLELFNYLNIDYLDINYNFEIDALTITMLLAITTISSMVHIYSIGYMETDPHQVRFFSLL

SMFTFWMIILVTGSNYFVLFVGWEFIGVTSYLLISFWVTRLQAMKSALSAVLMNRFGDAF

FVLGLCVIAYVFGTLNYSTIFATAYLINTDLLVLIMLALFIAAMAKSAQFGLHNWLTLAM

EGPTPVSSLLHAATLVTAGIYLLLRSANILEYTPTVLFIILWIGALTTLSAGLIAICSND

LKRIIALSTMSQLGMMTIAIGLSAYNLALFHLLGHAFFKALLFMSAGSIIHSILNESQDI

RTYGGLLSYLPYTYICITIASLSLMAMPGLTGYYTKDIIIESTYGSYSISNYVVYWIAYL

SAVLTCVYSMKILYLTFYSNPNNNTITYYNAHESNIYITLPMFILAIFAMFAGWILKDIY

LGVGTDFVGTHILPNNFSYFDTEFSITQFYKLLPLISAILVSILIVVLNEFFAIVFNLNN

KYINTVYSIFNQKLVSDQILNHFIIFKGLVTSGNIAHHVDKGSLYRLGPVGINRLLNKAS

YNVINLSSNTRSSLSMNSMLILITIVSLLLLVLVMNVNFIIVIPVLISILYILFS

>sp|Q9B6E9|NU6M_YARLI NADH-ubiquinone oxidoreductase chain 6 OS=Yarrowia lipolytica (strain CLIB 122 / E 150) GN=ND6 PE=1 SV=1

MMYLTYYFIEITIFLAILCTIFIISAKNPMVSILYMIALFVIAAMYLYLIGLGIFSLLYI

MIYIGAIAVLFLFIITLLDINSTELSVKSNIRDLPLVLISLIVLTISGLMIYSNDSILIN

KLLEAFGNDYNTIITQDWFNIENTTLLTTIGNVLLTNNAFILLVLAIVLLLGIIGPISIT

MKHKE

>sp|Q9UVF6|PALH_YARLI pH-response regulator protein palH/RIM21 OS=Yarrowia lipolytica (strain CLIB 122 / E 150) GN=RIM21 PE=3 SV=2

MHSDAPTPVANELSEVSHLAEGDNASFSAGNFTVLQLPSDPSHCIDYAIPAGTLVIVDPN

NPDNNRTVKLQAPAVFRPQCALGGTRAPAPRPEESFPDWSEYQKYHHNDRRDPFYGSVTP

IAYTIAASTVTAWMLLIILFLSRKPSPLFQKIAVLITAVSLTVFLAQATDTLESQYNEGY

QNAYELRHKIMGGWAFRILQVITCVITWLARLQVVIRLFDVPKINTRLAVVGSTLIFTNA

TIWACLNLIPPWSQYVRNAKSVLPVFGALCSLLLEVFYLVVVVIYSISKRKYAYSRTSIV

MAAISWLAMILPMVFIVFDIAHYWIAGWSDFIRWTADAAASVVVWEWTNVIVYQERREQR

QSVLGRQVYRDEILDFKGDNGGGTVGGGRTKYPSRMEEDDVPFRSSPNDHHFTSNIPTSA

GEGQSFQFFKRARLPMYSRKIWKIARGESAASNNTHYEHAIIEEEEEESIERNRRTPTVQ

ENGEEDDEETYDEENDQYSQDNHSSVHSFESSRPSQHPVPSSGGTRAVGHTHFPLPGQSE

GAHTTSPAAAAAAAEPEPEPVAGPSGGAAAHGDSDDDSDNSDDSSLASFTVIQQTGFSVD

NQGVPEYDADSAPPTFEPIPGFHRQDYSDAKG

>sp|Q7Z8R5|PALI_YARLI pH-response regulator protein palI/RIM9 OS=Yarrowia lipolytica (strain CLIB 122 / E 150) GN=RIM9 PE=3 SV=3

MGLRMKSATGLLVILLIAFALQLVAVLSVPVTKTISLGSYQDHKFGVFGYCNVKDGTCSP

AGVGYNLVDSDNAGFSLPSNARHTLSNLLIVHPIATGFTLILTVLAMLAHIQGPASSSRY

LLFCLVFSLPTFLLVLLSFLVDILLFVPHLDWGGWIVLAATILVAISGVVLCVMRRTLSS

KKAMKKHQLDTTNELSAFSSHKHLNSFTYSSKENVPQFSELRYETSHDTSKDEEVLPLTS

HVYEEPGHHVGDTSYASQGTNNSRVNLLTSEEPQAPKRESPFKDQRQDRYTPDRYGPSPD

RYDQGGPGRPPNGSRGVPPRRPSNGPTPPGQGPSPTGAYGRNNNPNYNGGYNNRLPRPRG

PPGSNNSSPFLGARNGPGLTPPHNLQTATTGPMQLPAGTYLPGEEPANSPDTYGPGVIPI

PEIRRESKVPGASPTPPPVSGNSPPTETSESGVSRPYRGNYSRRGSEASAQTPPNAQPNP

PPGGAQNYEYVPARQQWNLTTEESNATAPAPGPQPLQRNHSYDTYNPYRSETPSAPGSRA

QSQGTDDNLGTLHQPVPTTVTPSNSGALNNKDSPWYSPPVDEQFRNSFIPDAPVSPSESI

SSNFTSVSQRGINPRYFGADGPPGGLHQGAPPPPHMRGPPPHMGMGGPNMGGHNMGGHNM

GGPPPNMGPRPPYGGGGPGHGNKHDLLLSGNPDSQFQPQTRRKPGRGGRPGMSPASLMGR

DTGPYSMR

>sp|Q6CFW6|PAM17_YARLI Presequence translocated-associated motor subunit PAM17, mitochondrial OS=Yarrowia lipolytica (strain CLIB 122 / E 150) GN=PAM17 PE=3 SV=1

MSLQMLRSRVALARPTLVRPSVGQMTKRFAGTAPVPLTWERFLLLRTRRRQINFVASIFT

GVATSVLAWGFISEAELDLEQQDVFFGLDAFTAAGLGVVAAGFMGSLLGPTIGQLIFKAT

NSKQWPAFLMKETDFLSHIQKNRVNPRYQSVSNPVPDYYGEKIGSLKDYRRWLRDCAKYN

RAREV

>sp|Q6C5K1|PBN1_YARLI Protein PBN1 OS=Yarrowia lipolytica (strain CLIB 122 / E 150) GN=PBN1 PE=3 SV=1

MRRRATILADLQQHGDKISFKPGDNNKSKLTVSVNAPRQDRFSAPVSNADLDVLNHVADV

SVAWGRPSAAQQPVLFAAAYEPGLHVTVVKKQASEPRKWGEEKPSEAAPELVHVAKFLEE

QLGLPVDPKTFIESRSDYTYYDPLVGPEAFGHFVDKHSACSDESEEFVNDLDTFNYAQSA

SFHIDHDKKELVTEVTIPDLEHYFNQKKQHISRACKNDRLEVGIFDLNNLATSYAFRGVA

ELKGLLHDSTKEKPQETFLVIEPRHNIGAGSVSVDFERPVGLHPKSELKLRHIAIPTDHN

DENDGVKHCRLFAKYSAPSSIFFDKYQLADLERTTSEHPNGQGRLLALWGEADLEEPRYH

TEGWGSEALVEIFPITDAPEGTPFNLNFTLPLHLRYEEPKEGETYEPNEAPWPLVFWVCA

ETDEQLANFKQSPFDVTHNSYMRLFPEGVSYHHIGPREANGRGFPLLSTSWGTPVADIDR

FAAVRDYTSIMMVAGFLMVTIAILRKVFRGKAMSEELKEKKEQ

>sp|Q874C0|PEX3_YARLI Peroxisomal biogenesis factor 3 OS=Yarrowia lipolytica (strain CLIB 122 / E 150) GN=PEX3 PE=3 SV=1

MDFFRRHQKKVLALVGVALSSYLFIDYVKKKFFEIQGRLSSERTAKQNLRRRFEQNQQDA

DFTIMALLSSLTTPVMERYPVDQIKAELQSKRRPTDRVLALESSTSSSATAQTVPTMTSG

ATEEGEKSKTQLWQDLKRTTISRAFSLVYADALLIFFTRLQLNILGRRNYVNSVVALAQQ

GREGNAEGRVAPSFGDLADMGYFGDLSGSSSFGETIVDPDLDEQYLTFSWWLLNEGWVSL

SERVEEAVRRVWDPVSPKAELGFDELSELIGRTQMLIDRPLNPSSPLNFLSQLLPPREQE

EYVLAQNPSDTAAPIVGPTLRRLLDETADFIESPNAAEVIERLVHSGLSVFMDKLAVTFG

ATPADSGSPYPVVLPTAKVKLPSILANMARQAGGMAQGSPGVENEYIDVMNQVQELTSFS

AVVYSSFDWAL

>sp|P45817|PEX9_YARLI Peroxisomal biogenesis factor 9 OS=Yarrowia lipolytica (strain CLIB 122 / E 150) GN=PEX9 PE=2 SV=2

MTMSARVRKMALPSNCDLSDVEMICDNVLGCLEDLDSSRVVSRVTSREDVDESTIGDLIS

LDCVSLETPLFSFLFLGVTLFLFLSLLQQSLVFLLGVFLRLVQLVGHFLLLLMGEQIGVI

DKAMSHIPLPEVGQHSQQVQIQHLVERRFGLDVVTAIVVIGDNELFQVVGHQFRVGIMSD

GQRSQQSQNSGMNVASSSRGRHQLVPDRPGSQLSSQKLSSLVPLARIAAAEIPCAVQQSL

SRLFARSVQNRQVQRPHLDPQRQRNIVGVFGVQHGRAVLLCALGGDLIEKRPNQLVRVVK

VLVDKFPRRLPKRLVHLVHLGRGSLVHCRCDRVCQQRGCCHLWHCGGHFFVGLVSWIVDE

TAACVVFCLFKVVSETQRISSLPRYMHRELNTAGVVWGCGGKHM

>sp|Q6C4W5|PFA3_YARLI Palmitoyltransferase PFA3 OS=Yarrowia lipolytica (strain CLIB 122 / E 150) GN=PFA3 PE=3 SV=1

MQCRKCCFACEKWCFIGAKAFLPLVVNFLIIWACWVHAWLVCWEPQLFESDTTFWRVYGV

AGVAIGIMCNVLYLKVCKVGPGSPTDIDNFSVPLVEYQNACSAEGQHLTPPREMANSVCA

KENGGLRFCTKCIGWKPDRSHHCSNYKRCVLKFDHYCPWFATAIGFHNHKYFVLFLWYVT

ILCFFCLGSTGFVFYNHILEIGAMRGPDGNTDYVGAISVNVMILMVLALVFAIAVGTFAT

FSLYLVFNNQSTVEFLESTQYRSAVPTAAYRYTFAPTSKTVGNVFDVGWKRNFQLVMGDK

WWMWLLPIQPSEAARGNGTQFPLNKQVLQKIREAAAKEVQIRDQNQAYIKQQRQQQQKRT

QYDLPQHLQPPPQEHYEYDDEAQDSGDDIPLINMVNKNNTK

>sp|Q6C7Q0|PFA4_YARLI Palmitoyltransferase PFA4 OS=Yarrowia lipolytica (strain CLIB 122 / E 150) GN=PFA4 PE=3 SV=1

MITFSNPWIGVIIPCIIIFTLSTFSAIYILPHHVSNNELTLFICASAMVWISYIIAIIVP

PGSPPKNYTPPENGMKMYCLKCKAYKPERTHHSKALGVCVLKMDHHCPWTNNTVGHRNMP

HFMRFLVWVDMTVGYLFIRLCIRIMKLWRDKHLPSYLFDKTEVILSIVFLPASFFVLFTV

GILTIRVFVNMCNGITQIESWECDRIESLVRRKIVTEERAEFPYDIELFTNIFNAVGSPL

TFWLPWGQPRGDGITFEKNESGYTEEGEPLCWPPDHVDYDPENVPLQNLKDGLRRRGEEP

DCLLGGIGAGKMQAENDFYKRDHWRNVEGEKLADFGVEHTDI

>sp|Q6C7D1|PFA5_YARLI Palmitoyltransferase PFA5 OS=Yarrowia lipolytica (strain CLIB 122 / E 150) GN=PFA5 PE=3 SV=1

MNRAAFRQALRIVFPGVILGLLGYGTYAYCYILCWNLYHHMGYRAGLALLIVYCILKTLV

FIYWAAVVIVGPGKVTGVSPLQIFIPGSEVVDEKAQAIVSRQINPKLPDTYICDGWGMPK

WCSECQTHKPDRTHHSAIVGHCVPKMDHMCFWVGTVIGQHNYKIFLQYTTLFSTYLIYTL

VTTAVFTPRMGQYRRDRGAPDSIPNGNIIALLILTGAWAAFCTSVCLQSFWGVCRNLTAM

EGLGRKQGDMVLVNFRYEGKRIIQPLREEDPLPFDQGFAKNWRQVFGTNPLIWFLPFPVV

PAAEDYFSNAYGQKFLERIPARWLEGDGREMAAV

>sp|Q6C8W4|PLPL_YARLI Patatin-like phospholipase domain-containing protein YALI0D16379g OS=Yarrowia lipolytica (strain CLIB 122 / E 150) GN=YALI0D16379g PE=3 SV=1

MLKLKFRWNKPDDGPRVKRQKSQQLTLDSPEGSETSSRPGGGYDNDRDVNGTFKRPWDIT

KIPGYNQTYVNEASLDKFAAYLKEEEVPVESSTPVSSEEMLPLEPVQKEYITSKHDWTPV

FSVLKNNNSVNKRKMSHTALKSDFKHYLDMYDKEEKRRRGKSKKDKEGSEGDKTKTKEPK

ELVRKSVFSSILGVFSIFSKTKDGRKLKRDEIGEGIGFWLMRWPALFFIGMWLLFLTTIY

ASVRVLVAGYENILTWRGKRAKLRKVLRGARTYEQWVQAAQDLDVELGNAEWRENPKFGY

YDHVTISKLTKMVRKLRLQDQAEDLSNILQGCIKNNFAGTESSTLYSQTYYGTKKVVEQW

NEELGKAVTYVLESPKIDDEEKRDLFRLYSKNFGKSALCLSGGGCFAYLHFGIVKAMLDQ

DLLPQIISGTSGGALIAALACTRTDEELRQILVPELAYKITACWEPFPKWVFRWWRTGAR

FDSVDWARRSCWFTLGDMTFKEAYQRTGRILNVSTVPADPHSPVILCNYITSPDCLIWSA

LLASAAVPGILNPVMLMNKTKSGDIVPFSFGSKWKDGSLRTDIPVDALNTYFNVNCSIVS

QVNPHIALFFYAPRGTVGRPVSHRKGKGWRGGFLGAALESMIKLEIRKWLKFIKAVELLP

RFVDQDWTNVWLQRFSGSVTLWPKIHLADFWHILGDPWPEKMEDLLYRGQQCAFPKLLFL

KHRMNIEKRIRNGRQATRHRKRKLEETDVCDLVRKTFSESDPDSDVKHSFVFPALMAPRS

AGTDISSSNSDYDHEPQWEMDEGDSFLTADEEYPTTIL

>sp|Q6C938|PNS1_YARLI Protein PNS1 OS=Yarrowia lipolytica (strain CLIB 122 / E 150) GN=PNS1 PE=3 SV=1

MNDEEKHLAAPANAYQPNMHYQQQQEKQTGYNAEYDTHQGGYNQNQNQDYYNHSQGGYQM

DNMGQHGGYQGNPNDNYNNQQPPPYTPDFPPDYNYKPNPNAATFDEAFAVPKPKWNDKIG

LVILALIFSGYLALSIIVIRAYAQTHSFQGWGIYSGENDYSLNTHTLILYAFVLATAMVL

SLLYFIAARVWTKQFIWITYILHLLFSWGTAIYYLVVGYYSAGIVFIVFAALTTWWFWCS

RKRIPFATIVLQTLIDVTRANPSVLVISAVGTVVGACFGTWFSFTIVSIYVKYDPDNRNP

GCMTTGGSCSNGKLIGLILFAIFCGYYLTEVIKNVIHVTISGVYGSWYYCSKSDQGMPKH

AAMSSFRRAVTYSLGSISLGSLIVSIINFIRQILSVLQQDARQSGDTLATVLLCFVQCCF

GVLDWLVTYFNHYAYSYIALYGKAYVPSAKATWKLMQTRGIDAMVNDSLIGSVLSFGASF

VAYAAALVAYCFLKYTDPSYNSGGGFYAPVVGLAFVIALQVSNITNVSLKSGCSTFFLAL

ARDPEVLRVSYPQIYEEICRTYPPARDKLDI

>sp|Q6C4Q0|PRM10_YARLI Pheromone-regulated membrane protein 10 OS=Yarrowia lipolytica (strain CLIB 122 / E 150) GN=YALI0E24651g PE=3 SV=1

MMRTQSDEHVATRSSSANKQRQRVRFPKDNDPTTKRILYPADENDDGHDDSDEKETSVVI

PTPSVVIVGPDGEEPASAPISTGSPGADTDDYFSTHPNKKKSTLKSPGTPTTYSPSVPAG

RARSATGVTTHTPPKGSSLSSTTLMNTLLNSSGLGQYDTESEEDDDEDEEVTFTARPRQP

AQPSNSEEAGPSEQPQFRIDKVKSISLMRARQSRAREAQEEETVASTDDDGVGNNDMFKI

DDDARSVSTDESSDDESDADRAARADAPLIERERASAAHVDAFAQNSERGSNMAVGAAIM

GGLASFGSMSGGGMAPGAVDRSSESSQTLDDQRDQRDSHLALRRENALGMHSHSAPGSED

HTPGDASGNASAGNHDDGEDSLAGHDINDIPLVALDRRLEDIKEKRAEDKEKRAEDNEKE

RQHHHHNHHHHHSSETGPNTGASSPFSEEEKDREAEEAEILRDQARDLVNQHKHAVHTPD

AEDEDYDPFTAPAVDYFSSIHIHDDDEDEDNHGNFLTYDAADGSVTPTHEDYVAPPKRFK

QGVLGALLKLYNEEEGNKSTSTLATTVGDGTSTGDVSPMLYGSEPTTPGGTPIDHPTKSK

TKTTQKLGLKKKKKELLKIIEDQRKEKEENKKRPKWYDKSRSTSPSPGGTPAPHHHHHIP

GLHLHHHTKGHQRSHSEGYLDEMETAAGGGGDGGDKPPDRPRSLRSEALRPVKDIKKLNK

MAKNTGKNFKKRERELKRRRRQEVKITVHIAELLQRQRFILRMCRALMLYGAPTHRLEEY

MKMTSRILEIDGQFLYIPGCMIVSFGDATTHTSEMQLVRCVQGVNLSKLQDTHEIYKEVV

HDMIGVEEASNRLDEILRSKNLYPPWLCVIFFACGTGVVSPYAFRGRWADIPMCIILGSV

VGFFQIIVAPRSDLYNNVFEVSMSILISFLARAIGTISSGGSHPFCFAAIAQGSLAIILP

GYIVLCGSLELQSKNIVAGSIRMFYAVIYSMFLGFGITLGAVVYGWCDHNATRQDKCPQQ

LDPLWRILFVPLYSFFIALVNQARITQLPSMLLISGAGYTVTYFVGANIPDPDNSSYLTS

AIGAFTIGILGNLYSRLGHGLAFAAMLPGIFVQVPSGVASQGSLVAAIANSNRLIGNKNV

TTTIRETIVNSATSTVVTRVQTSLLTETSTAAIEQATPGGAANLGFTMIQVAIGITVGLF

AATLCVYPFGKKRSGLFTF

>sp|Q6C6L1|PRM1_YARLI Plasma membrane fusion protein PRM1 OS=Yarrowia lipolytica (strain CLIB 122 / E 150) GN=PRM1 PE=3 SV=1

MAMSLASQIKLWWNTQNSPTEHSSIPLLKARLSQTWLNKWTIGLLLVSIKLWMFKMSLSG

QLDGAESNSERSCSALERSVSKMMSIPHYTATGMNHVIATAIQKFVSGLIKMVLLIITGV

QELLIFAVNMLISTYTCLITLVVQGAVSLAVDTSKHVISFVNDTISTVVPEIEKGLNGLA

DGLNTATNAFVDLGNLITGKQAEEYTGQIDFVKLQLDGLKNVSIPASVNEKLDSVKDKVP

DFETVKNEIESLIRKPFQTISKSMNETLATPLNVSDSLKVPALKSAQFCKDANIPEVYAK

LNSGLNTGLKVIISLLLILALIMIVPVAWSEWRLWRYHEELWVEQSTVDVKQEKRHAFHT

VLFQAQHKYVTMVKQKLAWGKKLYQRNLSQWYWAYILYPYMLTLLLVGLFGILAFLLQLG

LLSILKSGLQSLTVPTSEVGADVTETVVREIETWKNDTNLFLNSQETHINKNLLGWVVDS

TSTVNTTLSTFIDTMNNGIDSVFKDTPLHGAVQGVTKCLVTLKLQKVADGMGWVSDNAHV

TLPRIDKELMSHQDIEQEASEKADYVSDGIARIIKTVEKLLFTELYIALACLGIWLFMCL

IALVYVLIKSRDRTTNFEPSRELKFDMVDTSRDINVPRVLPPQITIPKPALFMRNHIPSP

ATDPFEEYPIRRNKSSSWLFNLKSPVLRKS

>sp|Q6CDV6|RBD2_YARLI Rhomboid protein 2 OS=Yarrowia lipolytica (strain CLIB 122 / E 150) GN=RBD2 PE=3 SV=1

MAQFPLFQKFQKAIQHPPALSLGLPIFLTVIFLLSQRYVWIEDDLELRSTALTNFELNRI

SFYPLVHATWFHLLLNLVALQPIVSQFERVNGTVRTGIVLNILAVVTAIPWCLLSIGFFP

DEAVLGSSAWIFSFMGYWAIRESSKQPTTQLAPNLVVPTWLLPIIYLVVIAIVIPSSSFI

GHLLGLIAGWMMALGYLDVLIEPSSKVVLWIENKISRVIDLIPSSIVTFYREEGALDTRA

AARADTNRSLSVSGGNFLGFQANSSQADLEAGTRSRGNSSVDPTTSFPGTGQTLGTQ

>sp|Q6CBQ8|RCF1_YARLI Respiratory supercomplex factor 1, mitochondrial OS=Yarrowia lipolytica (strain CLIB 122 / E 150) GN=RCF1 PE=3 SV=2

MSDLPSSFDNGNSIDENEKSPGYYKILERCKEQPLVPLGCLATCGALILSARALRVGNKR

QANRMFFARVAFQGLTVAALIGGAMYYGQDPKQKLEQKEREKMLARHREKLWIEELERRD

LEVQERRKRAAAFRQQEEEK

>sp|Q6C6S3|RFT1_YARLI Oligosaccharide translocation protein RFT1 OS=Yarrowia lipolytica (strain CLIB 122 / E 150) GN=RFT1 PE=3 SV=1

MDELPSHEPKNRYSRSSSIGSPADARYAPPSPLHEPSDASQIAIAMGGVRQRSASIRKHR

RQQPSQVFIPPMHENVTAPRDEHEPKMSSPVPETDEKPLLQTSAAGATLLIGIQILSKLA

SFGLNQMLLLVATPALFGANAQLEFVLNTVLFFSREAVRLALQRLTLAGKKPDVYVFGGG

VVQDTVSGTSQAVINMGYISVLLGVFFSSVAAASHSLFSVAYASWAVQLVCIAAMVDLAS

EPYYVLAMQQLRFRSRAAAEAVAILVRCVVTFSFTLLAKDTDGGLNGGVLAFAFGQLAYS

LISSAVYIYTVRQDNRDRQFSFRPQKIQPFESQMEMSDNNRDVITHNASPYYLDKPTVRL

AGSIWIQTVFKHCLTEGDRILVSYFLPLYDQGVYAIVLNYGSLVARIVFFPIEEGLRTFF

SNLLGEKPSETALKLSRQVLCSVVRIYTYVALFAAGFGPTTLPFIFGTLLGARGGQWSEG

APSRSAPAVMGAFALYIPFMALNGALESFVQSVATPADLRRQAVALGVFSVVFATVGGLL

MKTMDLGARGLVFANIVNMTLRIGWSVVFIYHYYVSHKAENVNPTHLLPGKLVIATGVTT

ILASLFGVGRVSSFRDVGINIGLALALAAAIAVEERQALVQLVQLVRKKEDKVKVDDKEE

KEKESDKSEGESE

>sp|P79088|SC61A_SCHPO Protein transport protein sec61 subunit alpha OS=Schizosaccharomyces pombe (strain 972 / ATCC 24843) GN=sec61 PE=2 SV=1

MSDLRFLDLVKPFAPFLPEIAAPERKVPFKQKMLWTGVTLLIFLVMSQVPLYGIVSSDSS

DPLLWLRMILAANRGTLMELGISPIVTSSMLVQLLVGSQLIEVNMELKSDREMYQLVQKF

LAIIIAFGQATAYVLTGMYGRPQDLGAGICLLLILQLAAASLIVLLLDELLQKGYGLGSG

ISLFIATINCENIFWKAFSPTTYHIANGVQFEGAVINFVYVMFTWDNKAAALYQAFFRSG

LTSSQIQLPNLWNFFATLLVFGVVIYLQDFRVEIPIRSQKFRGYRSTFPVKLLYTSNTPI

MLQSALTSNLFFASRLLFNRFSSNFLVRFLGVWEQTATSGLSYYLSPPASFQDALIDPIH

TLVYVFFTMFACALFSKLWIEVSGASPRDVAKQLKSQQLVMAGHREGSMYKELKRIIPTA

AWLSGAVVGALAVASDLLGALGSGTAVLLCTTTIYGYYEQLQKEIKGDQYGLPVTPMMQ

>sp|P78979|SC61A_YARLI Protein transport protein SEC61 subunit alpha OS=Yarrowia lipolytica (strain CLIB 122 / E 150) GN=SEC61 PE=3 SV=2

MAGVRFLDLVKPFTPFLPEVQAPERKVPFNQKIMWTAVTLMIFLVMSEIPLYGINSSDKS

DALYWLRMMLASNRGSLMELGITPIVSSGMVFQLLGGTQLIEVNMDLKSDRELYQTAQKL

FAIILSLGQATVYVLTGMYGPPKDLGVGVCLLLIFQLVLAALVVILLDELLQKGYGLGSG

ISLFIATNICEQIFWKAFAPTTVNKGRGYEFEGAIVAFVHLLFTRKDKKRAIIEAFTRQD

LPNMSQLVTTVAIFAAVIYLQGFRVDIPVKSSKQRGPYGVFPIKLFYTSNLPIMLQSALT

SNIFIISQMLFKKFPTNVLVRLLGVWDGREGMQQLFPVSGIAYYMQPPFNAKEALADPVK

TVIYIAFVLGVCAVFSATWIEISGSSPRDVAKQFKEQGLVIAGRRETSAYKELKRIIPTA

AAFGGATIGALSVASDLLGALSSGTGILMAVTTIYGYYEMAAKEGYVDAAI

>sp|Q9HFC7|SC61B_YARLI Protein transport protein Sec61 subunit beta OS=Yarrowia lipolytica (strain CLIB 122 / E 150) GN=SBH1 PE=3 SV=1

MSTSAQVPGGPAAQMKRRNNAQRQEAKASQRPTSTRSVGAGGSSSTMLKLYTDESQGLKV

DPVVVMVLSLGFIFSVVALHILAKVSTKLLG

>sp|Q6CAG9|SEC11_YARLI Signal peptidase complex catalytic subunit SEC11 OS=Yarrowia lipolytica (strain CLIB 122 / E 150) GN=SEC11 PE=3 SV=1

MVNFGAQSIRQTLVQLLGFAAIFTSSYMFYKGLSIVANSESPLVVVLSGSMEPAYQRGDV

LLLWNRQKHVDVGEVVVYNIDGRTTPIVHRVLRSHASDNKQLLLTKGDNNAVDDVSFYGG

RNQYLDREKEVVGVVKGYLPLVGYITILLAENQYFKYGLLGITGLLAFIQGE

>sp|Q6C880|SEC22_YARLI Protein transport protein SEC22 OS=Yarrowia lipolytica (strain CLIB 122 / E 150) GN=SEC22 PE=3 SV=1

MVLSTLIMRASDGLPLSASVDDGQENLSEQKKQCKLVTQKLSANSADRASIESGNYVIHY

LMKDGIVYYCISESSYPRKLAFSYLDELDREFQKSHGQEALKEGVRPYKFVEFDTFMQKT

KRVYQDTRATHNLDKLNTELQDVTRVMTKNIEDLLHRGHSLDHMSDLSSNLRTESKKYRR

AAQRINWEAMIRQYIPFIGVGLIGLFMLWWMLF

>sp|Q99161|SEC62_YARLI Translocation protein SEC62 OS=Yarrowia lipolytica (strain CLIB 122 / E 150) GN=SEC62 PE=3 SV=2

MTENVPQGQITMPLQQGGAREISPQALAVADYLRSHKLLKQRPGILNGKRSDFFRVKRAI

RALEDPKYKQLQSKPNSKLPPINSRNEAISIFRLMPINQMALRVDKLPTQTALMMKQKPE

QGVPVLQVNPQQEFGDDMYYTWFYNPVPLTTYLYGALGVAAIFAGVLFPLWPIFLRQGVW

YLSVGMLGLIGVFFGIALVRLVIFVLTWPTVKPGIWIFPNLFADVGFVDSFIPLWAWHGT

PERDLLPQKFKNKKKKKNAGTVIESKEPPRKLTKEEKQKQKEANAQMEQMQAAFQTQLSS

FATQMQQIKEMSDSGIDPQIIAAQLQAQYPPDKQAAIKLENEQAQAKLDERIRELAAQIQ

DKTNANKTGDKIEEVADKENKEAPKRIVTLEDANDE

>sp|Q6C3B0|SEY1_YARLI Protein SEY1 OS=Yarrowia lipolytica (strain CLIB 122 / E 150) GN=SEY1 PE=3 SV=1

MTSQSHGAPPVPSSRPPASRVPVSGYDSHDSHSVSSSHSSHSPVTTHHHPAPPPPASRPV

RESGVAPVTAPEPIAAPEPIAAPEPIPAPEPIAAPVPEGLKSEHKPVEREHKPVERKPVS

SPAEKSIPASSPVHKAAPTPAASHAVPTSQKSAKSTPGSYAGIPSLQLIDGNKEFNPDVS

SYFKKVHLDRAGLDYHVVAVFGSQSTGKSTLLNALFGTQFDVMNETARQQTTKGIWMARA

QLEAPHSANSAHSQDCSDSGVLVMDVEGTDGRERGEDQDFERKSALFALATSEVLIVNIW

EHQIGLYQGANMGLLKTVFEVNLNLFATSQNRSLIMFVIRDHIGATPLANLSTTLKTDMG

KLWDSINKPEGLEHAKLEDFFDLQFTALPHKLLQPNEFYADVEQLACRFTVPKDPNYVFK

PVYHRNVPLDGWSFYAEQVWDQIEQNKDLDLPTQQILVARFRCDEIAAGALDIFLSLLVK

IRDQLSGGAVASAVLGGLMGEARKQTVDEYDSQASRYTPSVYSATLEKLEDRVDNDLGKV

YQSYLAQLKRESLEQFNAALESSSALTFGENLSRASKAAHAHFIDNAKQVTAAIGQPNSS

HFSYDDTLAALEQELDTLRDHKSKVEIDRLISRSAKRFKSSFHEEFDENLNKPDETVWDR

ILESFETLLNASIKKIDPNYSPSAPSAFSFGFGSPKTSAEGLKQIQQEAWAVFGAELKEL

SKEEQVLSRLKNKFKESFRYDANGVPIVWRPGDDIDGAFAKSREQALEIMPLLSTAKLSS

GKSIEPTVALEDDEDDDDETAFAVILTPKRQASLIEKFKKQAEGLYLEAKRSTIQSTTQI

PLYMYGLLLLLGWNEIMAVLRSPVYFMFLLVAAGAAYVIHTLHLWGPLTHMTNTMIAEAT

DMAKAKLKQVLNEAPTGETREREAPVGSSRDDVELKDL

>sp|Q6C7F7|SHE9_YARLI Sensitive to high expression protein 9 homolog, mitochondrial OS=Yarrowia lipolytica (strain CLIB 122 / E 150) GN=SHE9 PE=3 SV=1

MLTRRMQCLRGAGVFRTVAALPLLAQRPNFYRPLTCSALRRNNDHSYRHESRKDKEMQDL

KESRKMREQEALDDAAFRNTFEFAVEKTRDSDGKSPEPEVEIDVDREAQTPWDPVGSQSR

HETRQEHRERFDDIKKTLPSSVHESQPQMYKWFGEKMDQIQAGALTAGQTLNEVTGYKAI

EKLKLSIEKLEDEVLEARAEVREAKRMYSDAISERSNSQREVNELLQRKHNWTPADLERF

TELYRNDHANEHSVNDAKKRLGECEHHVEDLTLQLSKQILTRYHEEQIWSDKIRRASTWG

TWILMGFNVVLFIVVQLGLEPWKRRRLVGSFEDKVKESLQEWEARNEARRLEEATMAATA

TQAITELQRRIESPHVSKADVVEAEAEINSIKTNLATWFKEREEEEKEEIAKEAHELAVI

AEDPDMTDVHHKPHPPQPVDTERVVRPHLVENFEHHVADPVHHPTLPITSTPVLEEDAPP

PRIHPLVQMSPDSPDDGTKTFTATRDGYDVHPLVVRPTTPPHFMLQVWGAAKLSVESATN

KTVAVVRSTFQTAEQRPFESTIVASLGGLCGGLVTFLYLR

>sp|Q6CAC5|SHO1_YARLI High osmolarity signaling protein SHO1 OS=Yarrowia lipolytica (strain CLIB 122 / E 150) GN=SHO1 PE=3 SV=1

MRQPFSYRPQFQLGLLMGDPFALSTISLGIIGWIIALGGSIGASDQFKNFGWWGLAFEFF

VILCVFLVVATDSVEPYRQMLLAFLALATMYVTNSTNNVVYNGTSASSASGAGHILLSII

NFLWMIYFGTTHEAGIHAWVDSFAADKGHSYNNDQFAMRRSNPFSLHDQQRQSLGVPGRP

VSAYSAGMGGVANYNGLQLGGFENSSNAEPHQASPAFSQQQQQQTATPQQATDSLFVATE

YPYRARAVYAYQANPEDANEISFDKGEILDVSDISGRWWQARRDNGEIGICPSNYVELLA

>sp|Q6CDD0|SMP3_YARLI GPI mannosyltransferase 4 OS=Yarrowia lipolytica (strain CLIB 122 / E 150) GN=SMP3 PE=3 SV=1

MIWNNRLILALSLLLRLHLAISPSYIHPDEHFQGPEYALGNLFDWAHETTWEFRGDSPIR

SFVPLWILYTAPLSVLNFLWKGQLSPREAYWFIRAGHALAYWILGDMALDRLSDSKKSKT

KTLYLVGCSYVTWSYQSHTFSNSTETLLVLWCLVIIKESQQRHSMHHQRVHKFMDAGLLG

LLIVIGTWNRVTFPLWLIVPGLTYLRKYLIHNISSLILLIASVALTAFFVIHVDSVHYDL

EWTITPLNSFLYNSQGHNLAEHGIHNRLTHLVSNLPVLLGPLLILLRTPSQYWKSLQFQS

AISGVFFLSLFPHQEARFLMPAVPLLISCYDINAVPRRFTSAIFLLSYVFNIIMGFLMGT

LHQGGVVPAQHYLSKHVDSGSHTVVYWRTYKPPSWLLGIPEGELEILDKDHPLGTNLFKR

VTDEIENIQIRHKKTMVTVLDLMGSSPEYVNDVIAALAPINPLLVAPVAGLKELDLPAYK

EVWKTRFHLGLDHIDGLESLEPGLVVLEVL

>sp|Q6C4R5|SPC3_YARLI Microsomal signal peptidase subunit 3 OS=Yarrowia lipolytica (strain CLIB 122 / E 150) GN=SPC3 PE=3 SV=1

MHTSIQRIQQTFSQASTVLSIIAAIVFVVSYIQLVVANVWSLPEANFNLRGSKAARRFSR

QYGANDPKKGKENVALKFDLDADLSPLFNWNTKLVFAYLTATYDGKRDDIVNEITIWDQI

ITDKDDSHIKLKGANSKYSLYDVEESFRNRNATVKLHWNIQPHVGAKIYGSLDATKGSIK

FPQLV

>sp|Q6CG20|SWF1_YARLI Palmitoyltransferase SWF1 OS=Yarrowia lipolytica (strain CLIB 122 / E 150) GN=SWF1 PE=3 SV=1

MWLKIYLLSILAISVFTFVFLFGALPQFEDTAVWKFRKWLSNRPAAIRSWDSKYCGGRLS

VVGDFCGSVVAPAAPWSVPILYCAFTTYMFSAYYEDLHPFIAENHWYYAWLAPVAYTILV

VSFVLATFSDPGKITKQNHALLLNQFRFDNLMFLEDTECSTCKFTKPARSKHDRFTNKCV

AKFDHYCLWINNTVGLYNYRWFLFFLLGNVWTLCWGALLAGLKMIVMVAAEYKDHPKPLP

SIFSQWWQVMITNENKRVGIIFLLSVSTGALACAFTAMHFYYIYLGATTNETDKWGDIHA

AISEGSVWMFQKPGFKLDRSILLQKDEEGRPNRSLTAEEREYVAQNGLALTLLTDHKPIV

NIYDKGFLNNLKAVMFPNSAY

>sp|Q6CAQ9|TIM21_YARLI Mitochondrial import inner membrane translocase subunit TIM21 OS=Yarrowia lipolytica (strain CLIB 122 / E 150) GN=TIM21 PE=3 SV=1

MLTRPLLALSRPMALGALRPAVLVRTLTTAGPTTTVRSTPIITPSHLSTLSRSTPFVSNC

TLSLLKRSFHVSSDARSAAKAEGKPKVSAYEKANMRLAKIGVFFQLSWYLGIILAALGLF

GLVWYYLIMELVMPSGDVRIFNRAFKEIEKNEDVMRVLGGQLSSMGEGGGGRWGRNQPPV

SKRGIDKYGREHIWMNFYVSGDINEGRAKLELVQNTDSKLSSERFVYRYFVVDIPGHKRI

YIAGNAAEKMEKKKSTGWLGVNWGKSDDE

>sp|Q6BZY4|TIM22_YARLI Mitochondrial import inner membrane translocase subunit TIM22 OS=Yarrowia lipolytica (strain CLIB 122 / E 150) GN=TIM22 PE=3 SV=2

MSAFGFPGGGAPTPPQGSFWDMTPEQQGMYSANLIMGTMQSCPGKSVMAGVTGFGLGGVF

GLFMASMAYDAPVGMGVQTMSDLPFKQQMKIQFTDMGKRAWSSAKNFGFIGGVFSGTECC

IESLRAKNDIWNGVAAGCLTGGGLAVKAGPQAALVGCAGFAAFSAAIDVYMRSDNKAPPS

TDEDL

>sp|Q6C503|TMEDA_YARLI Endoplasmic reticulum vesicle protein 25 OS=Yarrowia lipolytica (strain CLIB 122 / E 150) GN=ERV25 PE=3 SV=1

MKSIVSVLTLLLLINAVAALRFVLPAKDKNELPFCVRDFVKNGELVVVTVESPKYADGQQ

LSVVVRDAHGNEYTRIKNVLGREITTFSSHQDTALDVCFHNVANSHQDLGKTKEIDLSVA

IGANARDWEQIQASEKLKPAEVQLRKIEEIVDEVDKEMNYLKMREIRLRDTNESTNRRVK

FFSVGITLALIALGVWQIIYLRSYFRSKHII

>sp|Q6CE86|TSC10_YARLI 3-ketodihydrosphingosine reductase TSC10 OS=Yarrowia lipolytica (strain CLIB 122 / E 150) GN=TSC10 PE=3 SV=1

MIFPISEIPDKVTHSILEGVSALQNMSHTAFWSTVLGFLVVARIAVILATPKRRVLDIKG

KKVVISGGSQGAGAALAELCYTKGANVVIVSRTVSKLEAQVQKIVTKHEPVFEGQTIRYI

SADLTKEEEAIRVFSEETMPAPPDVIFSCAGAAETGFILDFKASQLARAFSTNYLSALFF

VHAGTTRMAKEPISPKNPRYVAIFSSVLAFYPLLGYGQYCASKAAVRSLIDSLRVEALPF

NIRVVGVFPGNFQSEGFEEENKSKPEITRQIEGPSQAISAEECAKIVFAQMEKGGQMITT

DLIGWILQSIALSSSPRSFSLLQIPLAIFMCIFSPVWNAFVNRDVRKYFHANTEYVTRHQ

RGGVGSENPTPQ

>sp|Q6CC06|TVP18_YARLI Golgi apparatus membrane protein TVP18 OS=Yarrowia lipolytica (strain CLIB 122 / E 150) GN=TVP18 PE=3 SV=1

MSLVEELKTRNFSIYGQWIGVLCIILCIALGIANIFHASLVIIFSIICIVQGLVVVFVEI

PFLLRICPVTERFSNFIRFFNQNWPRAAFYIGMATIQYCSLIFMTTSLLVPAVFLTITSM

CYALAALKHQEFTGSSTLGGAGIARQIL

>sp|Q6C7V5|TVP23_YARLI Golgi apparatus membrane protein TVP23 OS=Yarrowia lipolytica (strain CLIB 122 / E 150) GN=TVP23 PE=3 SV=1

MTLWQRLSESSHPVALVFFLAFRLGALFTYMFGLLFTDKFVLMFVLVVLLLAADFWNVKN

IAGRLMVGLRWWNEASETGESVWVFETADPQRYINPIDSKVFWMMLYGAPVLWVCLAVLA

LLKFQFLSLILVFIAVSLTVTNAMAYSRCDKFGKANNIVGQVSGGLLSRAARGTFLGRFM

>sp|Q6CDK3|VMA21_YARLI Vacuolar ATPase assembly integral membrane protein VMA21 OS=Yarrowia lipolytica (strain CLIB 122 / E 150) GN=VMA21 PE=3 SV=1

MADIPRSVLNKLVFFTACMILLPLTCFFTAQMFTDNTLISGGLAAFVANVVLIGYVIVAF

LEDVPVDEKKEQ

>sp|P42781|XPR6_YARLI Dibasic-processing endoprotease OS=Yarrowia lipolytica (strain CLIB 122 / E 150) GN=XPR6 PE=3 SV=1

MLRKFILGLLLASQAVAQLPHKERDYDSRVYVALSLRDGLDPREFEASVSGLDHGQWTFE

HPVGTIPNTYVFSAPKEYAPIENIRDQDRLEVAGGVLAKRELRKREKLQKKYGMSEEDVE

KRLVALERLDYDWSERGLGSLEVLSERRIHKRAPVNWTEEEMEYLKEIKRRAEEAQKAQD

DKGDKKEDQKDDKKEGQEAQKEGDKEDNKGDDKEDGEEDDDDDEDEDDDDASPAMPVQWK

PVDESMYGGMPDDSLYDVYRKYYPDEVGIKDPSLWKQWYLHNVHKAGHDLNVTGLWLRNV

TGWGVVTAVVDDGLDMNAEDIKANYFAEGSWDFNFNKSDPKPSSHDDYHGTRCAGEIAAV

RNNVCGVGVAYDSKVAGIRILSKEIAEDIEALAINYEMDKNDIYSCSWGPPDNGQTMARP

GKVVKDAMVNAITNGRQGKGNVFVFASGNGGSRGDNCNFDGYTNSIYSITVGALDFNDGH

PYYSEACSANMVVTYSSGSEHYIVGTDINAIDDKSAAPRCQNQHGGTSAAAPLAAGVFAL

ALSVRPDLTWRDMQYLALYSAVEINSNDDGWQDTASGQRFHHQFGYGKLDASKIVELAEG

WNLVNNQTSFHSEVKTVSQKVKYNEPLKSVITVTRDDLDKVNFKRAEHITAVLNLEASYR

GHVRVLLKGPRGVVSELAALRRDDRSKDGYDNWAFMSVAHWADEGEGDWELTVENTGEQD

QVELVNWQLNVFGEQKDKREENKEGESKPEDENKEGEKEGEKKPEDENKEEGNKEDDKGD

QKEDKPEDKPEDKPEDTPEDKPEDKPEDAPEDKPSDEKKPEEKPEEKPVDNSDSSSDSSD

SHTSWWPDLSSKKSAWLYGAVLLVGGFIAVIGIYACVTRRNRVRRNRSKDAPSASSFEFD

LIPHDDSDDDFVYPEDTHRRSGDNDRLYDPFAEVEDDDDMFRISDEGEDAHDVEPELNRV

SMEADKRDNDRQNLLG

>sp|Q6CE07|YOP1_YARLI Protein YOP1 OS=Yarrowia lipolytica (strain CLIB 122 / E 150) GN=YOP1 PE=3 SV=2

MSQIIDQVQAALQNIDKELEKYPALKELEKQIPVPKSYILLGFVGFYFILIFLNIGGIGQ

LLSNIAGLVIPGYYSLLALETPGKADDTQYLTYWVVFATLNVFEFWSKAILYWVPFYYLF

KTAFLLYIGLPQYGGAELVYKAIVKPLAQKLVNIQPHGGPSDSLKAQAQSAVDAAESHVP

QGHSTGVSH

>sp|Q6C2Z7|YSH1_YARLI Endoribonuclease YSH1 OS=Yarrowia lipolytica (strain CLIB 122 / E 150) GN=YSH1 PE=3 SV=2

MARWSGQIMCFRVGHFLLVGHFLLVGNFLLTDDSDTFSFVALGGGREVGRSCHVISFKGK

TIMLDAGVHPAHSGLASLPFYDEFDLSTIDILLISHFHLDHAASLPYVMQKTNFKGRVFM

THPTKGIYRWLLSDFVRVTSGAESDPDLYSEADLTASFNKIETIDYHSTMEVNGVKFTAY

HAGHVLGAAMYTIEVGGVKVLFTGDYSREEDRHLNQAEVPPMKPDILICESTYGTGTHLP

RLEREQRLTGLIHSTLDKGGKCLLPVFALGRAQEILLILDEYWEAHPDLQEFSIYYASAL

AKKCIAVYQTYINMMNDNIRRRFRDQKTNPFRFKYIKNIKNLDRFDDMGPCVMVASPGML

QSGVSRSLLERWAPDPKNTLILTGYSVEGTMAKQIINEPNEIPSAQNPDLKVPRRLAVEE

LSFAAHVDFQQNSEFIDLVDSKNIILVHGELNNMQRLKAALLAKYRGLKNSPREKTIYNP

RNCEEVELAFKGVKVAKTVGKMAEEKPHVGQIISGVVVQKDFNYGLMGVADLREHVGLST

SSVLERQTVTVNAGVDLVKYHLEQMFGYVEMRETENVKIEEMEDDVAEEEEDKEVKQEVE

DVTMEGEVKDETAEEVKKEEEVAEEFKQEVEGDSDTSAGTTFVVMNSVTVKHTPTSCTIE

WVGSCLNDSIADAVLAILLTVDNSRASVKMSSKQCAHSHGHEDGHSNSSLDERVLQLSSI

LKAQFGDSYIVSEDGKSANIKIDAMEATISFSDLSVTGSPPPLVQRVQVAVDRAISLVAP

LAQKLSAVDLVEGFKAIENVKDREENGEVKAEDEEKVKAEEKVKEEE

>tr|Q9P8M1|Q9P8M1_YARLL ADP/ATP carrier protein OS=Yarrowia lipolytica GN=AAC1 PE=2 SV=1

MSDKSNFLVDFLMGGVSAAVSKTAAAPIERVKLLIQNQEEMIKQGRLSRPYKGIIDCFKR

TAADEGIASFWRGNTANVIRYFPTQALNFAFKDKFKKMFGFKKSEGYWWWMAGNLASGGL

AGATSLAFVYSLDYARTRLANDAKSVAKDGKAAGERQYNGLIDVYRKTIASDGIAGLYRG

FGVSVVGIIVYRGLYFGLYDSLKPVVLVGPLEGNFLASFLLGWTVTTGASTASYPLDTVR

RRMMMTSGTGVKYSSSMACMASIIKNEGVGALFKGCGANILRGVAGALVISMYDQMQMIM

FGKKF

>tr|Q9UVF7|Q9UVF7_YARLL Peroxin 10 OS=Yarrowia lipolytica GN=pex10 PE=4 SV=1

MSDNTTIKKPIRPKPIRTERLPYAGAAEIIRANQKDHYFESVLEQHLVTFLQKWKGVRFI

HQYKEELETASKFAYLGLCTLVGSKTLGEEYTNLMYTIRDRTALPGVVRRFGYVLSNTLF

PYLFVRYMGKLRAKLMREYPHLVEYDEDEPVPSPETWKERVIKTFVNKFDKFTALEGFTA

IHLAIFYVYGSYYQLSKRIWGMRYVFGHRLDKNEPRIGYEMLGLLIFARFATSFVQTGRE

YLGALLEKSVEKEAGEKEDEKEAVVPKKKSSIPFIEDTEGETEDKIDLEDPRQLKFIPEA

SRACTLCLSYISAPACTPCGHFFCWDCISEWVREKPECPLCRQGVREQNLLPIR

>tr|B5MFD1|B5MFD1_YARLL Putative uncharacterized protein SCS2 OS=Yarrowia lipolytica GN=SCS2 PE=2 SV=1

MEITPEKLEFHAPFTKHTSNNLELRNPTNEYFAFKVKTTAPKLFCVRPNASIVAPNESLT

VSITHQALPQEPGPDYTSKDKFLILSAPLNEAAVQAGENLANFVEKTKENIAEFWTQAEH

TKSVPITSKKMKVQVRPFDANGQHSHELGAGGAALGAGAVAGGALGHSASRGDQTLGDES

FANYEAAAQSPAPHSPANQQFNNFQQQQQQHYQQQVPSSASGIASHPTEAVNRGAEAVGA

PSASSQVHSQDSAALGQAQDNISSLKKDIGSTSEKHTVPSTQTSQAVGSQGVPVGVVAIV

ALLALLVGWFFL

>tr|Q6H9R7|Q6H9R7_YARLL Putative scj1 protein OS=Yarrowia lipolytica GN=scj1 PE=4 SV=1

MRLAIQAVFLLATLMWLVAAQADFYAVLGLKKGASDKDIKKAYRTLSKKYHPDKNPRNEE

AHQTFIEIGEAYEVLSDEEKRGKYDKFGHEGLKNGGGGGTNPFDLFAQFFGGGGGGQRRG

VPKGPNTETHIDVSMKRMFKGFDMDLQVNLQGICSSCKGSGSADGVNHKCDGCDGSGVVI

QVAQMGMMIQKFQQQCPKCQGKGHLISNPCNKCGGQKVQREDRKYNVYIEPGTPRVHSYS

FEEEADKSPDHVAGDLIVQVREAPTENMGYRRRRNDLFRTEALSLKEALHGGWTRKIPFL

DEESEVVLSRKKGEPTQHGHIEVIKNHGMPQMGVDDSHGDLYIKYVIVMPMGASSKVRDE

L

>tr|Q6H9N7|Q6H9N7_YARLL Putative uncharacterized protein OS=Yarrowia lipolytica PE=4 SV=1

MQFSIVLLAAAAGLVSAGNATAVVTQIGDGQIQAPPSAAPVQANGAAALGVSAAAAGVVA

AAAMLI

>tr|Q7ZA17|Q7ZA17_YARLL Hrd1 protein OS=Yarrowia lipolytica GN=hrd1 PE=4 SV=1

MSSWRLILALVISTLVTCLMFAEALVVRPNFYSACISLTQSNSKKSVLLATILTHIFCAR

KLLMRIFYGELRAVEEQHTGEKLWYSGTEILFAIAVFHKELSQVHYASIFTFVWLVKSLH

WVAEDRVDLLFTTGESDWAHVRYLSTLTVLCATDVYLLNSFYPHIDFDFSKFSTGVEQGI

WVILALEIGLVLNSIALCHNKYLISMRERYFLRQNPNDETWTHKKDWLFAAEAASDLIQI

AMFCIFFVLISSSHGMPIFKFRDAVVSVLNLVSRVKGYYNYRVLTRQVDSFTTTPSEDDL

ARNQTCIICFEDMELVEEPKQLVPNKLSCGHVLHNGCLKHWLERSKLCPTCRRNVFTAPE

VVATLPWPRLHKSQFPWLRSLQITPRGWSRLCSHKAALPLSRHQTTLMPTTSSSSDKVPL

RRVCMTRGKSFRPGETTKTRWSCVWEREDGPFLKLSISI

>tr|Q8J0M1|Q8J0M1_YARLL Mitochondrial ADP/ATP carrier protein OS=Yarrowia lipolytica GN=AAC3 PE=3 SV=2

MGGSSDTQHFWIDFLMGGVSAAVSKTAAAPIERVKLLLQNQEEMIKQGRLSRPYKGIIDC

FRRTWGEEGLASFWRGNTANVIRYFPTQALNFAFRDKFKAMFGYNRKKDGFWLTLYGNIA

SGGMAGATSLFFVYSLDFARTRLANDAKSVTKGPDGKPVEGQRQFKGLIDVYVQTIKSDG

VQGLYRGFVPSVIGIVVYRGLYFGLYDTLKPIVLVGPLEGNFLASFLLGWVVTTGASTAS

YPLDTVRRRMMMTSGQKVKYKSSIDAFSKIVAAEGVGALFKGCGANILRGVAGAGVLSIY

DQLQMIMWGKKF

>tr|Q8J0M2|Q8J0M2_YARLL ADP/ATP carrier protein OS=Yarrowia lipolytica GN=AAC2 PE=3 SV=1

MSDFLVDFMMGGISAAVSKTAAAPIERVKLLIQNQEEMIKQGRLSRPYAGIIDCFKRTAA

EEGVVSFWRGNTANVIRYFPTQALNFAFKDKFKKMFGFKKSEGYWMWMMGNLASGGLAGA

TSLAFVYSLDFARTRLANDAKSVAKDGKAAGERQFNGLIDVYRKTIASDGIAGLYRGFGV

SVVGIIVYRGLYFGLYDSLKPVVLVGPLEGNFLAAFLLGWTVTTGASTASYPLDTIRRRM

MMTSGTGVKYSSAFDCGVQIVKAEGVASLFRGCGANILRGVAGAGVISMYDQMQMILFGK

KF

>tr|Q70MH4|Q70MH4_YARLL Alpha 1,6 mannosyltransferase OS=Yarrowia lipolytica GN=och1 PE=4 SV=1

MALITARRVILLGLAVATVCFLFFGSSEKIAPTVLLPGDMSAFENKIYKMRAETEKALKE

QEAAAAKRFDSFNKKLSELETENRVLEKEIQRLRTPPQGASLREKLAYTFPYETYKKFPA

FIWQTWKDEITDDTPDTIRQPIRTWTEKNPSFVHEVLTDDAAAMFVQHLYAQIPEVVEAY

KAMPKNILRADFFRYLVLLARGGVYSDVDTEDLKPIPNWIPDEVSPSTVGLIVGIEADPD

RPDWKEWYARRIQLCQWTIQAKPGHPVLRDIVARIVEKTLAKKRSGTLEMAGDKTDGSEI

MDWTGPGVWTDSVFDYFNDRKKSGLKSQVGIKDFFNLKTPKHVSDVLVLPVTSFSPGVDQ

FGAKDTDDPLAFVKHLFSGSWKPEDERMNQNQPQVNQ

>tr|O74132|O74132_YARLL ALK6 OS=Yarrowia lipolytica GN=ALK6 PE=3 SV=1

MIQSVFLALAILIAYLGFAEWFSRFQHRRISKKKGCGMPPMANGGFLGWYGLYKTYQITS

ERTYPHSMRMGLEAFGHTFVYPVPGTDMLQTIHPDNIKAILATQFKDFSLGTRHKIMLPT

LGDGIFTLDGEGWTHSRALLRPQFARDQVSHVASLERHIQVLFKTIKKENKECDPAKGFD

IQELFFMLTLDTATEFLCGDSVDSLTDYLADPTAPQLDHSGIDENVRRAFPEAFNTAQWF

CSIRAKLMKLYFFAGTVFYRKKYADANKIVHDFTDFYVSKALAARKEKFQELDQEGKYIF

LYELAKETRNPKVLRDQMLNILLAGRDTTASLLSWVMFRMARQPETWKKLRQAVINDFGD

TPDELSFESLKRCEYLRYVLNEGLRLYPSVPMNFRVATRDTTLPKGGGPDLDQPIFIPKG

GIVVYSVYHTHRAEEYWGKDTEEFIPERWDPAEGYQIARGWEYLPFNGGPRICLGQQFAL

TEAGYVLARLAQEFETVTSCDDKPLPPKYNTHLTMSHDDGVWLKME

>tr|Q6H9P4|Q6H9P4_YARLL Putative sugar transporter OS=Yarrowia lipolytica GN=stl1 PE=3 SV=1

MKLFKREAPQGTVRTSYFGSRGTRLHRIIAAVAGIGFLLFGYDTGVMGSLLTLPTFIHQF

PTMDSTSPHLDAKTKSFHSTVQGTAVAIYEIGCMLGALFTMWGGDKLGRRKIIFIGSIIV

TIGAILQCASYSLGQFVTGRVIAGIGVGFTTATVPMFQAECARPERRGALVMLGGALTTG

GIALSYWIDFGFYFVKRNDSDWRFPVAFQILFSLILSCTVLYLPESPRWLIKKGRYEEAA

GVFAALEDVPIDDIYVSQQLMQVKESLMVGQLAQEGIEGDEARRRIASGDVELGEEPPFR

KQLVLLFTFGKKKHLHRAMLAYSQQIMHQMCGINLISYYAAYIFQTSIGMSPLNSRILAA

CDGTEYFLASWIAFYTIERFGRRKLMLFGTIGQACTMAILAGTVYAASSVKDGGLDKPQA

GIAAAVFLFVFNTFFSIGWLGMAWLYPAEIAPIEIRAISQGLSTSGNWVFNFLVVMITPV

AFNSIKWRTYIIFACINVAMVPSIYFFFPETMGRSLEEIDLIFEDSNPRTPWDVVGIAKR

LPRGTLDGVVPSDMELEKEIEHVSVKSLGGATIRG

>tr|Q2UZR5|Q2UZR5_YARLL Alternative oxidase OS=Yarrowia lipolytica PE=3 SV=1

MSMLEPQTFWDGSDRRTMKTRIILQLSSLLIPLQPMDPLLLPLSQWLTPNPMTCLSTLPH

RTTMSTSQAPLGSIQSTPRSRWTLSRSIIRKTETFSDRVALRAILLMRIIFDLCTGYKHP

KEGEAHLPKFRMTTRQWLDRFLFLESIAGVPGMVAGMIRHLHSLRALRRDRAWIESLVEE

AYNERMHLLTFLKLQKPSVQMRTGLLIGQIIFYNLFFISYLISPATCHRFVGYLEEEAVI

TYTRCLEDIDAGRLPELASMEVPDIARTYWHMEDDCTMRDLIQYVRADEAKHCEVNHTFG

NLHQTSDRNPFALVIDNGRPQPSKDLTTFRSVGWRRDEIAN

>tr|Q6ZY24|Q6ZY24_YARLL NUVM protein OS=Yarrowia lipolytica GN=nuvm PE=4 SV=1

MVELKPSSAIQRGPLNKGGWDAPHALHNDGAIDRYAHWRTFYQERFKYTRATGKSTLIFL

VAFPALIGYVAYQSEGLFEFAGKRRGESVTTRG

>tr|Q70ML1|Q70ML1_YARLL Alpha 1-6 mannosyltransferase OS=Yarrowia lipolytica GN=anl1 PE=4 SV=1

MISRDKKPNELPKYEKTGGVDYRTDFKPRKPSPLSRPNLQVPQITKLRLAAVFVAVTLIV

FLFGSHHEVSYSDVLQSVQGYKQYVSDSIKQGSISSQSPAEAHRKPLGEVSFVDLRDAAG

DWSKNSPLNQRVLVCMPLRNVEKVVPIMASHLRNLTYDHNLIDLAFLISDTDDNTVNVLE

DEINSIQSDADPKMPFNKITLLFRDFGSAVGTDFSDRHGVAVQGVRRKLMGRARNWLLSS

LLEPTHSWVYWRDADIETSPPTIIEDLMKHNVDVIVPNVWRPLPDWLGSEQPYDLNSWQE

SQPALDLAATLDEDEVIVEGYAEYPTYRPHLAYVRNADGNPDEQVDLDGIGGVSILARSR

VFLSGAHFTGFTFENHAETEAFGKMCKKMGFTVRGLPHYTVWHMYEPSEDDLKEMMRREK

EEKEKEKQEKGTTENKDLEVNEAPEVKELEA

>tr|F2Z676|F2Z676_YARLL Alpha-1,6 mannosyltransferase OS=Yarrowia lipolytica GN=ANL2 PE=4 SV=1

MFDRNQGGLAPSTRKTLGLVSVFGTALYLCLSILSWVFSGELALVPFTSSSLPPMADLGI

DSVHHYDLADYTGSARGQDRNERVLFLVPLRDAAAHLPMLFAHMDKMTYPHVLIDLAFLV

SDSKDDTMGVLLKELNTLQSNRDVSKRFGSIEIFEKDFGQAVGQGFSDRHGFEAQGPRRK

LMARARNWLLSVALRPDHSWVYWRDADVEKVPTTIMEDLMSHDKDVIVPNVWRPLPDWLG

NEQPYDLNSWKESDAGLRLADTLDEDAVIVEGYAEYATWRPHLAYLRDPFGDIHVEMELD

GIGGVSILAKAAVFRHGAHFPAFSFQKHAETEGFGKMCKRMGMSVVGLPHYTIWHIYEPS

DDDLRHMEWMAQQDELREKERVLKEAHDKVWQSGFDNVKQEWERERELILKNVELDFDVH

QKVRAKKAEEQSKAEAKVHGPEEEKVVPGVGKAALQPDAVKKVVQG

>tr|Q8X1X9|Q8X1X9_YARLL Mnn9p OS=Yarrowia lipolytica GN=mnn9 PE=4 SV=1

MRSARRYLLVLLPVVALLYVLSTYFRGTPVEKPVARNMAGNHIVKYNMNKLQASSQAASH

SEKVLILTPLARFYDEYWENLVALSYPHDLISLGFIVPRSKRPTGAEKLDRAVAKTQKGK

DKFSKVTILRQEAEDALAFTFGKGPTRDVCAEGPAKCHVSGSKLTVPVHQWPRHMQGILW

LNSNVVETPHTLIQDMTKHDKDVLVANCYQRYTNDQGKPDVRPYDYNSWVESQQGIKMAE

SMSPDDIILEGYGEMATYRLLMAKIYNADGDIHEEIALDGVGGTALLVKSEVHRDGAMFP

PFPFYHLIETEGFAKMAKRLGYKPYGLPNYLVYHYNE

>tr|P79067|P79067_YARLL Delta 9-fatty acid desaturase (Fragment) OS=Yarrowia lipolytica PE=3 SV=1

VSITAGYHRMWSHRAYKAALPVRIILALFGGGAVEGSIRWWASSHRVHHRWTDSNRTLTT

PERDSGSPTLAGCCLCPTPRTRSELTFLTSTTTGLSDSSTSTTVYVLVFMAIVLPTLVCG

FGWGDWKGGLVYAGIMRYTFVQQVTFCVNSLAHWIGEQPFDDRRTPRDHALTALVTFGEG

YH

>tr|Q6H9P3|Q6H9P3_YARLL Putative Sip3 protein OS=Yarrowia lipolytica GN=sip3 PE=4 SV=1

MNDQGVPTNQLKPNAAANMPITLIPVIFKEASLDSPSFRATVNHVISQLAVTSQWLEGFV

QSVHDVTAEMHTLQAVINNLVGKFMPSFVTEGVVDHDYTMLVLRRFAEGSKLFWTNAMKN

INGQNTHLGEPLVKLLKQRIAPFSETIKAFRAAQTKYDAAQAKYLALNKTREPSHLRDEA

MAVYEARATYIKASFEFCAGYLRLHHAIDRALILTITDTWRSNARNHDDSAHQHAGSAYF

RLGIEMHRIRNWSNAIHQANKVIVREIGKARKEIEDSTLAAAKPSKDIAEYQPLSPSTKL

SVDSKVTNHPIEKHGWLLMKTNSPAEKNPIWVRRWAFVKAGLFGWLNVSPPPECDFVEES

DKIGVLLCSIKQLPTEERRFCFEIKTQEVTLIAQAESLTEFKSWLVAFELAKQQSLDKES

PYRSSLAFKKWTVPLPEFASSGGTSIDVAMAHTSSKTTDTTSPSYLGGPHAYASEHHVHQ

TPLHKLMASGKAFIDGNETNEHGEKSAFNFSGFGPFNTSLAPSPPIITPIYTRMTKEAIV

STAFVKPSYMPNAVTANVWGSVNWALYAKNSTVAIASHENMVEFQRKMDENPTTETDPFP

ENYPIELKSQDLQMRAIFQTAVDHDPNDRVVTVFRCLWSPNPRQELPGRVFVTLKNLYVY

SYSLGFVCAVKKPLTEIVSVQGSPDNAWENLYVLGTDGAVLRCRVFLDSGRLVQRRLQII

LSNVLSDSPRNLEEMLPILQDGDSDHKILTENDGWYERESDLGYIDNVQGEHDEKFSQEE

LSEHLVKRYRDLRNKRKEVKATKRPTSSSARSVASKTVDTPDYSLHSSTAQKVVDREFLC

PPKALFHIMFGQKSRMFKDIYSRFHKVISSRRTRWMLNPEDKTLSRLITYDLKLRSLMET

GTLVSRNLQVIERMDENVCYVVHNSRNPWEISHVSSFYQSMRFVITANDTGGSRLCIWDD

LVYTEKKRSLFKSVVETVAMRYMEHEAESHVRLIQQCLRRVGTFDSTNRSIIMYGKLGQI

DTDPTPEQLAELERKDSQDVLKVSRRRLVTWVSESIFTFLLTALTAFMIIIVKLAKGFFK

SLSPHIVLIVLLAVSGVSNLWLSGRLTYFHIKQHQEARRAAPSDTLYRAVFINDLDPMML

PSDTNNTDSIALQKYLSLEKSRVFGMDPDKILKGDDGIGLEDKFFENKYFSDVASRSASR

KIQKARYMLGIKRRELLVNLRVLNRSDRDLAIEEWQNWLRSEVRNCERFTARVAADPLYG

KDKLDEGKFEQIENYCNSCRAEIKVPALL

>tr|Q2UZR4|Q2UZR4_YARLL Alternative oxidase OS=Yarrowia lipolytica PE=3 SV=1

MTLLTTTSLLRTSRALAKVSVSVSRPTQFQHRFMATATSKEDYVKDPGDTKTLANTKMND

SSIYGADDSQKYETTPMWVHPIYTKEQMENLVVRHREAKTFSDKCALAAIRTMRWSFDLF

TGYRHPKPGQENLKRFEMTPDKWYQRFLFLESIAGVPGMVGGMCRHLQSLRALKRDRAWI

ETLLEEAYNERMHLLTFLKMHKPGYFMRTMILLGQGVFFNLFFMAYLMSPRICHRFVGYL

EEEAVITYTRCITDIDAGRLPEWEGHKVKIPEIAIDYWHMGPNPTMRDLIEYIRADEAKH

CEVNHTLGNLDQDHDRNPFALKIDNGHPQPSKDLSTYKGQGWSRDENAN

>tr|Q874C3|Q874C3_YARLL Longevity-assurance protein OS=Yarrowia lipolytica GN=LAG1 PE=4 SV=1

MASPKGANPVEASKPPVAVAHKQKTTAWRGLMDYLSARQITLPLKVLTYIVVMHRFAALR

PYTRKFFHLQYPKHEVDGVAAYYSRGRDDIYFVLTGILALTFLRAACMDFVLVPMARALK

ITKRKPQLRFAEQGWALIYYTSSTWIGFYLYYHSPYWLNVEELWRGYPHFELDPFFKAYY

LIQFSFWVQQIFVLNMEEKRKDHYQMFTHHIVTCALMCGSYYYYYTRVGHLILVLMDGVD

TLLASAKMLKYLRYDTMCDAMFGLFVIAWVVLRHGLYNYVTWSAYFQAPVLVAENCLRDE

DGQETCFNPALHRVFVVLLIALQIITLIWLYMIVRVIVKILKGGGAEDSRSDDEDSDEEE

GEAEGEEEEEEEEEVMEEKVSDDN

>tr|Q9HFC6|Q9HFC6_YARLL Calnexin OS=Yarrowia lipolytica GN=cnx PE=3 SV=1

MRLSKLAVSSVLAAVACAQDADAAADAAPSSQVEHPEFTPYTGAVTGFFEQFLDGHKWQK

SSAMKDDEFSYVGEWAVEEPYVFPGFKGDKGLVVKSPAAHHAITTAFDTPINNKGKTLVV

QYEVKLQKGLECGGAYVKLLSAEVNADDKGVEEFSSETPYQIMFGPDKCGSTNKVHFIVK

RPLPDGTYEEKHLVSPAHARLNKLTNLYTLVIRPKNEFEIRINGNVVKTGNLLEEGLFKP

SFNPPAEIDDPEDTKPADWVEEPYMPDPEQAEKPADWDEKAPFYIADPEAVMPADWQEDT

PDYIVDPEAFKPEDWDDEEDGEWVAPEIPNPVCEEIGCGPWVAPKIQNPDYKGVWSQPMI

ENPDYKGTWAPKKIPNPNFKADEHASDLEPIGGLGFELWTMQEDILFDNIYVGHSVDEAE

AIGNATFVPKLALEAEEEKLSGPQKETAPWDTDEGVLSSVDMFLADPVSFVLERVLGFFE

VFSQDPVSAIREDPVGAAASFGLLLITSATAFGLLNVIIFLLFGKKKQSAAPAKKTKKTG

DGPSKLTKADVVEAEAEAEQAAETVVASGVDDGLPSSRSERA

MTKIENSHVEYTNDDTERRLSTSSSVNSKKSEVIVSTITATLPPTLASLSQDDIDVLEKK

FVRRIDIRMLPMLMLLYILNYLDRNNIATARLGGLEKELNLHSVQYQTAVSILFVGYILM

QIPSNMIVSRLGKPGLYLTTCMFIWGTISALTSTVHSFGGLVACRFFLGFIEAVYFPGCL

FLLSSWYTRKELALRTSILYCGSLISGAFSGLLGAAIMENMDGMRGISGWRWLFIIEGSF

TVLVVPFAYIVLPDFPQTTSWLSQQEKELGIWRLQRETGTTDIDSEESAWQFVKKNMLLV

LKDPKVWLVAGINFFNVAAAGVTNFFPSVVETLNFSKTITLVLTAPPYLIATVLVPLNSW

HADKTGERCWHIVLPFCVTIASFIIAAATLNTGARYFAMCIMIPSIYMGFVVTLTWMSNT

IPRPAAKRGVALALMNCLSNTTSIWNSYLYPKSAAPRYAPAMAANSVFLLVAIGCAIALS

MLLKRINKKLDRGEYDIVAEFGENDDDPVVVHQTQSFRYVY

>tr|O74129|O74129_YARLL ALK3 OS=Yarrowia lipolytica GN=ALK3 PE=3 SV=1

MIIIETLIGAVVFVAVYVAFVKLDYYRRKAKFETSDMPVAYNGLLGWKGLRHMLTVFNND

IGPVGWREVFATYGKTLKYYAFPSNTILTYDPDNIKAMLATQFKDFSLGLRKEALAPSLG

YGIFTLDGSSWSHSRALLRPQFSREQISRLESVETHVQEMMSCIDRNQGAYFDIQRLFFS

LAMDTATDFLLGEAVGNLQEILHPEMPRTGTTFQVAFDRAQRLGSLRIICQEAFWVVGSL

FWRRDFNNTNQHIHDYVDRYVDKALLARKEKSEIYTNPDKYIFLYELARETTNKITLRDQ

VLNILIAGRDTTASTLSWIFMELAKKPDIFHKLREAILNDFGTSCESISFESLKKCDYLR

QVLNEGLRLHPVVPVNLRVAVRDTTLPRGGGPQGDKPIFVAKGQKINYAIFWTHRDKEYW

GEDAEEFRPERWETTSGGALGKGWEFLPFNGGPRICLGQQFALTEMGYVITRLLQEYSDI

SIQPSDAAVKVRHSLTMCSAQGINISLTRAKEE

>tr|Q8NJX0|Q8NJX0_YARLL Integral peroxisomal membrane peroxin OS=Yarrowia lipolytica GN=PEX24 PE=4 SV=1

MFRRLTGSQSESSIYGLVNDSQTSMGTMDYANELASQLDMLLKKDKPLSTDDSASVASSS

AASRTSWLGPSIPRHSRHFVDALYDKVILPKLKKSIIGDVDSDLGSAVATPTGSGSRRGS

FSTIRALDISEEPFSESDISVFDDSVSVASTASSEPFRISQHNPPKPVFDPEAHEAGPEL

SIPLLLRNIAELSKRAGGVMKTQTVVLKAIQWQNPCLSISCIFIYTAMCLYPGIVFVLPG

LLFMYGIMAPAYAEKHPLPKEYRPSPWTVKNEFDMAADDDTSIRAAQIKRIKAAEKKPVD

VKKSMKNFQNATTNLIKALDKLESFLSGPAGFANESLSALIFLLIGFAMIGTFFLSAFIP

WKALQIGAGWGVIIAGHPTLLKRIVAILDELEKSDDEEKPKESPVIAILQKVQKKEFIND

EPPQGETVEIFELQRQGLTPRQWDSWVFTSLVYDLGSSWRQAKKRPPGAPFLGDVEAPEG

WLFSKMHDWEVDMNPNEWVGERGLGRCTEMDNDEWVYDVEKGVRGEWRRRRFIRTAFKES

TVPKELLGLV

>tr|O74127|O74127_YARLL ALK1 OS=Yarrowia lipolytica GN=ALK1 PE=3 SV=1

MSNALNLSLALGVFLLAYYGFSVIQYRIKTRKLEKKWKCGKPKDISRFPFSASFFIPFLV

ESKKNRLLEFVQWMFESQVYPGYTCKTTVFGVDMYHTVDPENLKAVLATQFKDFCLGERH

AQFLPVLGNGIFTLDGQGWQHSRAMLRPQFARDQVSDVEMIEEHIQYMTSRIPKDGSAFD

AQELFFNLTLDTATEFLFGQSVGSQTVETNPTAVPTDMPVHLRKSFQEDFNTAQEHLGQR

ARLQMFYWAWRPRELYSSGERVHAFVDHYVKKALEESEKHVDDGKYVFLRELAKETKDPI

VLRDQALNILLAGRDTTASLLSWCLYLMARRPEVYAKLREEVIENLGDGEDLSTITFESL

KRCDYLRYVLNEVLRLYPSVPANMRYATRDTTLPRGGGPDGMQPIVVRKGNLVSYHVFTT

HRLKEFWGEDAEEFRPERWYEDGASQAKGWEYLPFNGGPRICLGQQYALTEAGYALARIA

QLYDTIENADDKPEPPVKFHALTMCHHTGVLVKLYNSKTTKAQ

>tr|Q9UV60|Q9UV60_YARLL Integral peroxisomal membrane protein OS=Yarrowia lipolytica GN=PEX23 PE=4 SV=1

MSDKEKKKSSATHAAFPPSTASQPQSMSPLLSSTPPTVTKALAQAYPYILASDKVLGLLT

WTEDDQWQSFLLVAVYVTVVMYYEYLVIYCGHILAVGFIWAFVYIRQSVERRQTSEPSLD

AIVHTLTNVTTKANLLLLPITSLSLTPRDVTRLAFTTLFLSPLYMFGAYFFLGPRKFLLT

TGVFFLTYHSMAARVTRAVIWKSKAIRLVTFYLTGLDFSNTKRNLGAFGFTQSPLSVQSK

DGKPVRFTYVLYENQRRWLGIGWTANLLAYERTPWTDEFLNEVTPPSEFKLPDTEGTGMK

WQWVDPTWRLDCTNDGALVIIGNKALSTPDPSPSEGWIYYDNTWKRPTADDSFSKYTRRR

RWVRTAELITVTKPTDVVVTVEEDGVTDAAGDVEIITTETEEKVRRRKGIRFEEDSKR

>tr|B0LCC1|B0LCC1_YARLL Cytochrome c oxidase subunit 2 (Fragment) OS=Yarrowia lipolytica PE=3 SV=1

DIVMFYMVVTIVLVSYLLFVMIKNFSNDHISYKYLTHGTTLEMVWTMFPVVILLFMAFPS

FILLYLCDEVIDPAMTIKAMASQWYWTYEYSDFIGETGEMVQFDSYIVPTDMLENGQLRM

LDVDARMVVPTNTHLRFIVTSRDVMHDFALPSLGIKCDATPGRLNQVSALLQRESVYYGQ

CSELCGVLHSSMPIA

>tr|Q8WZL3|Q8WZL3_YARLL Opt1 protein OS=Yarrowia lipolytica GN=opt1 PE=4 SV=1

MSQYSRHAFDTDSDSDTELTPIYIDDRYSSMVSNDSRAGLLRRGGDLERDAESEKTREEK

EEELRNHSFSKSRDKTRETVADATLESSSCVDSQYDTVETTSIFSVGRKKAINSPYPEVR

AAVPATDDPLMPQNTPRMWVIGIFLVTICSGLNILFTLHWPAFVISSFFAAMVAWPLGRI

WDQFVPNWRIPLTNIYLNNSPFNIKEHCLIIVMANVSYGTGVSYVPVIVLTMKHRYNNTY

FGWGFSIVSSICVQCLGYGMAGICRRILVYPASLIWPSNLVTTTFLTNIHMNVNHTADGW

KISRLRFFLIVFSAYFVWNWLPGYLAPFLSNFAFVTYAAPDNVIVNQIFGSSSGLGLIPI

NFDWNVIAGYIGSPLVPPFFSIGNVAAGVVVIFWIITPILHYSNAWYGRYLPMSDSNSYD

RFQRTYDAERVLKPLVKGGKKKFELDVEKYKEYSPLYLSTTFALSYGVSFAAITSTVVHT

ILFHGKEIMYYWKHSRKEPEDVHMELMRKYPEVPEWWYALVLAICFALACVTVSVWETDL

PIWALIIALLIAGGLLIPAGMIAALTNVTMGLNVLTEFVVGYILPGRPVGMMFFKTFGYI

SNAQALNFLVDLKLGHYLKLAPRVMFCAQILATTWGSICQLAVVEWAQMAIPDLCEESQK

TNFSCPQVQVFYSASVIWGLVGPGRIFGEGQLYHGLLWMFLVGAALPFFSWLWLKRHPNS

RLKFIHWPVFFNGPSQIPPATPYNYASWCVVGYIFNWLIKRRWFNWWAKYNYTLSAGLDV

GMAVSAAVIFVSLQLPNINPPSWWGTNIFTPTMDIQGNAFQEVLKEGESFGMKTWK

>tr|I0CKU9|I0CKU9_YARLL Lipase 11 OS=Yarrowia lipolytica GN=Lip11 PE=2 SV=1

MCSNQINQLAGFILDLHLSIFVLLMLQYSSETFLNRVVEGWSGAKGLENPTCKVIKRSTR

SRRNSPESWTVSFSFFFASSFCSFSCFAMLLSSLVLSALSLVSVTAQNVVQATQDTWDLI

NYAEHLSSLAICGEPYGVIYKPFQCAGRCSDFPDMELITQFTPQDPLDFSVSGFLAVDHK

RKVFWHVFRGTATLNNGLTDLRIKRQPLTSWNTAKMDCPDCQVHVGFLQAYNLAYSEAKG

AMDDTFAKYPDYQVIVTGHSLGGAATFLHGINLKTSGYDPLVITSGQPLTGNKALADYND

KLFFGDNPDFTHQGPDRRFYRVTHKEDIVPRIPFWTPYHQSGGEVYIDFPGINPPVNTLK

VCDGQQNPLCSFSTSLASTATQGIVEAAHLIYFTFFFLCSTLLYPPLNSDLPVGVWGKPL

NGTI

>tr|Q6H9Q1|Q6H9Q1_YARLL Putative uncharacterized protein OS=Yarrowia lipolytica PE=4 SV=1

MNVVTLLLYVSTVSAVVVTVTLIETAQAVDSSRAATTKSMTSTSSSSRAQILATGTPPMF

ASAKNAIISNVRSLKVPSASGYLAKDVEPTQSVTPFTLSSSVSPATTASANPTSAVSVAQ

TNSVSRTGVSVMFLSLSFVLVTCSIA

>tr|O74134|O74134_YARLL ALK8 OS=Yarrowia lipolytica GN=ALK8 PE=3 SV=1

MIPFTKINLELLLLPLLIIPITLFLLVLNVQVNTVHRRYKEYRLKLARCPQQSTGLFGWW

LIYAVVRCNRDRFYSAKVVDYFTKLRTFIVSLGGELVIYTSESENIKALLATQFSDYDLG

KTRHALLFTLMGDGIFTLDGQGWAHSRALLRPQFSKETVSPLSSLETSLQQLMAIVKRRV

ALKGEVDIQELFFMLTMDTATNLLYGESVDSLGDCLKETKKETHVMTDGKRNLLQSRDHH

SNVPNEWEPIYGTYEKKGLGIGSENSLQSGVSPRDSPVFEVSVSEDIRKAYPAALTTALE

FSAMRSKLQRFYWIWGDVLYRQKFHSAVNTVHAFSNHFVNQALKLTPQELQMKSAEKYTF

LYELAQRTRDPVAIRDQLINILIAGRDTTAALLSFVFLCLVANPEKLAKLREGIMTDFGT

STDSITFESLKRCVYLRYVINEALRLCPPVPINMRQANKDTTLPTGGGKNHDEPIFVAKN

QIVTYSVLFMHHNQNIWGPDASEFRPERWGEPACPKGWEYLPFNGGPRICLGQQYALTEA

AYVIVRLVQEFTEIEWRDRKGLPVLFKTHITMSLGEGLRVHMK

>tr|O74130|O74130_YARLL ALK4 OS=Yarrowia lipolytica GN=ALK4 PE=3 SV=1

MLTNLTIVLITLLVTYTVLTRTALRIQRARKAKQMGATLPPRVNNGILGWYGLWLVIQNA

RSMKLPHTLGKRFANGPTWLTPVAGNEPINTIDPENVKAILATQFKDFCLGIRHRALSPS

IGDGIFTLDGEGWTHSRALLRPQFSRQQISRVHSLERLMQILFKLIRKENGEYFDLQNLF

FMFTLDSATEFLYGASVDTLADLLGEPVEGDHGGVGEEVRKAYQQSINNAQDISAIRTRL

QGLYWIAGNIYQRNLYQKSNKGVKDFSQFFVDKALNTSKEKLKEMEDSDNYVFLYELVKS

TRNPVVIRDQLINILVAGRDTTASLLSFTFYTLGRRPDVLKKLRAAILEDFGTSPDEITF

ESLKRCDYLRYVLNEVLRLYPSVPINARSATRDTTLPRGGGPDGKQPVFVYKGQMVAYCV

YWMHRDKKYWGEDALEFNPDRWDPKVQPQNKGWEYLPFNGGPRICLGQQFALTEAGYVVT

RMLQEFDTVHCKNQKEEEHPPYALDLTMRHGEGVWVSMK

>tr|Q9UVK9|Q9UVK9_YARLL Fks1p OS=Yarrowia lipolytica GN=FKS1 PE=4 SV=1

MAVKAATAPVAPATPPSLQKPCRQLQLSYDYNYDYQQPGQQGGDGQHNGQNQQHPGQQEY

YDDYYDDGYGNQYGAQEGYQEGVDQHGNPYQHDPHAGVAAATSGGYRNVKDDGSETFSDF

TEQNGQGYNQYNQGYNQGQYNQGYQGQYNQGYGGYDQYGQYNDASQISFRSSGASTPIYG

LDYAASQQGIGAGSGHSREPYPAWSADNQVPVTKEEIEDVFIDLTNKFGFQRDSMRNMYD

FLMVLLDSRSSRMTPNQALLSLHADYIGGDNANYRKWYFAAQLDLDDDIGFRNMKLGKTN

NKRTRKARKKFRKEMQDPDADPAKTLEEIEGDNSLEAAEYRWKTKMNALSPLERVRHIAL

WLLCWGEANQVRFTPECLCFIFKCADDYYTSAECQQRVEPVEEGDYLNRIITPLYRFIRG

QGYEIFDGKFVKRERDHNKVIGYDDVNQLFWYPEGIERITFEDESRLVDVPQSERYMKLG

DVIWDKVFFKTYKETRSWFHVFVNFNRIWIIHVTFYWYYASFNSPTLYMKNYVPTLDNHP

PPACKWGAGAIGGVIATGLQIIATLSEWAFVPRKWAGAQHLTRRLMFLIGILIVNLVAPV

YVLGVVGTTHESTSALAVGIVGFIISIFTFIFFSIMPLGGLFTSYMKKSTRRYVASQTFT

NSYPRLEFHDKIMSYLLWVCVFGAKLSESYYFLILSLRDPIRDLSQMKMRCFGQKWFGVE

YHDALCKVQPQITLGLMYATDLILFFLDTYLWYIICNTIFSVARSFYLGISIWTPWRNIF

SRLPKRIYSKILATTDMEIKYKPKVLISQIWNAIVISMYREHLLAIEHVQKLLYHQVPSE

VEGKRTLRAPTFFITQDDHAFETEFFPRNSEAERRISFFAQSLSTPIPEPLPVDNMPTFS

VLVPHYSEKILLSLREIIREDDQFSRVTLLEYLKQLHPVEWDCFVKDTKILAEETAGFGE

GSNDDLAEKDSDEVKAKIDDLPFYCIGFKSAAPEYTLRTRIWASLRSQTLYRTVSGFMNY

SRAIKLLYRVENPEVVQMFGGNTEKLERELERMARRKFKFIVSMQRLTKFKPDEMENTEF

LLRAYPDLQIAYLDEEPPLNEGEEPRLFSALIDGHCEILENGRRRPKFRIQLSGNPILGD

GKSDNQNHALIFHRGEYIQLIDANQDNYLEECLKIRSVLAEFEELNVENVNMSPYTPGVN

NKTPCPVAILGAREYIFSENIGILGDIAAGKEQTFGTLFARTLAQIGGKLHYGHPDFLNS

IFMCTRGGVSKAQKGLHLNEDIYAGMNALLRGGRIKHCEYYQCGKGRDLGFGSILNFTTK

IGTGMGEQMLSREYYYLGTQLPLDRFLSFFYAHPGFHINNLLIITSVQMFMIVMMSIGPL

AHETKETICWYDKDKPITDPQTPVGCYNLKPVLDWIRRCVLSIFIVFFISFVPLVVQELT

ERGVFRAAFRFARHFMSLSPLFEVFVCHVYANSFINDLAFGGARYIATGRGFATARLPFS

VLYSRFAGDSIYLGARSTLMLLFGTIAMWQAALLWFWVTLIAMCISPFVFNPHQFAWTDF

FIDYRDFIRWLSRGNAKWHKNSWIGYVRLTRTRITGYKRKVLGDESEKGAGDLSRAGISN

VFITEIFLPLIVAAFCFCGFTFINAQTGVAEPVIVNSILRIVICAIGPIVINAGLLLVLV

CISCCAGPMLGLCCKKTGPVMAGIAHGIAVIIHLIFFELMWFFEGWSFVKGLCGTVTAIA

IMRLIFNILTILCLTREFKHDHANQAWWTGKWYGAGLGWASISQPFREFVCKLIEMSLFA

GDFYLGHWLLFFQLPVLCVPYIDKWHSMMLFWLRPSRQIRPPIFSLKQNQLRKRIVKKYA

TLYFIILIIFLVVLIAPAVAGPTMSMDSLESLAGGKADNDSIQDTNILKGLIQVRSQNQN

DTGRCSTGNGTECLLPTTGSWSTHVWWTFTPSHTTSYSTKP

>tr|Q6H9P1|Q6H9P1_YARLL Putative uncharacterized protein OS=Yarrowia lipolytica PE=4 SV=1

MGALLSIPLAVIPSVSAIGTWAATCCGAAIGSAMCSACNKCSSSIATRVGYAVLFLVNSI

LSWIMLTDWAVKKLERFTLDYMKFKCLGEECTGFVAVQRMNFALGVFHLIMALCWVGVHS

TKNPRSKIQNGYWGFKIALGLALIVLCFLIPEKFFWCWGNYFAMIGSAIFILIGLVLLVD

FAHSWAEQCLERIEETDSGTWKFILVGSTMSMYIASIVLTILMYVFFCTSGCSMNQAAVT

INLVMLMLVTLVSVNQNVQEYNPRAGLAQAAMVAFYCTYLTMSAVSTEPDDKNCNPLVRS

KGTRTASIFIGALFTFVAIAYTTTRAATRSSVIEPEPESLVDDTVYTEPSAVTMRQQAIR

AAVEEGSLPESALHEQEWETFEADDEKSTTKYNYVLFHIVFLLATQWTATLLTMNVEKDD

VGDFVPVGRTYFSSWVKIVSAWICYFLYTWTLIAPVWFPDRFS

>tr|Q6CAY9|Q6CAY9_YARLI YALI0C23254p OS=Yarrowia lipolytica (strain CLIB 122 / E 150) GN=YALI0C23254g PE=4 SV=1

MVSINIFIISTLAAFVAAQAGFFVTQVSQGIVSGFVQGRSSGVDEQVASSEAAELVSLIL

NDPTLNPQFQTAADLVAFGFDGPKFVSFVSQAATAYSSFKDGPNFPIATSYFKEHNTDFN

LNSAILSAQAQVTFYISLFTQQLNPLTVTPAVQTATASGISLAKDLFSELGNYDAVTVIP

W

>tr|Q6CCP3|Q6CCP3_YARLI YALI0C07744p OS=Yarrowia lipolytica (strain CLIB 122 / E 150) GN=YALI0C07744g PE=4 SV=1

MQQQINKCINEIASLANLSPESSLTTATSLLPSPGHKVAFVVLCTLQLSLCLYTAFFLSK

RRGTIKDLHYDVKDVTESVEEAYYLINELNSRVAQSTKEQPQSPTGVLDKHKEQLVANVL

SKMEDKLTHPIEAIKTRLTALENNLASLQRANSNFNPRAPPYRGGGKLTRDPRGNENYRH

GNGGGDFRSFDSRNDSRFNESNYSLDSRPWKPWNRTGGRSYANSTPTSAPIEFYGEKRSF

EKMEWADKHNVASPVVTSVVEPTAEVIQPVARPVSAVPVPTTITGAPSPATQSVPKLVVN

CTPQQSKKIIIKAGDREYDLASAEGREQWSSVAKRNIAKAITLPSHVQVQDIAPPPLAPL

RTANTRPHTIELSPLTSTYSSETDGTEASKASKPKEGGVTQEERRAGGLDNYIESVGSPQ

SPNASLSRGLQLKLEENVSKKESSGSIGDASNGGVGGVGGVGGVGGVSGDGEKKPYRRLF

IPGRGWVSARRIAMEKAQEQGGEITGCDRRDLGASASDTDVQPPVGENTAVAA

>tr|Q6CD11|Q6CD11_YARLI YALI0C04730p OS=Yarrowia lipolytica (strain CLIB 122 / E 150) GN=YALI0C04730g PE=3 SV=1

MGFRGQRLHHYVATVAGMGFLLFGYDQGVMGGLLTLPSFVKQFPKMDTSDYLPPDVKSFN

TTIQGTAIAIYEIGCMMGALFTMWGGDKVGRRYIIFYGSIIMTIGAVLQCASYSLGMFIT

GRVVSGVGNGFITATVPMLQSECAKPEKRGKLVMLEGALITAGIALSYWIDFGFYWVKAN

DADWRFPVAFQIVFCLFLFFTVLTIPESPRWLVKKGRFEEAAGVFAALEDVDIEDPYVVT

QITYVKESIMLEQLAQLGIDGPAAREKIAAGEFSMGEELPFLSQMKLMFTFGKKKNFHRT

MLAYWSQVMQQITGINLITYYAAYIYETSVGMTPTNSRILAACNGTEYFLASWIAFYTIE

RFGRRKLMIFGAAGQAATMAILTGCVYAASSPADGGLDNQSAGVAAAVFLFVFNTFFAIG

WLGMSWLYPAEISSLEIRAPANGLSTSGNWVFNFMVVMITPVAFDTIKWKTYIIFAVINA

AMVPVVYFFYPETAGRSLEEIDQIFADSNPKTPWDVVWIARRLPKSTAVDHNLVEPRILL

EEKTALETVESVSPTPSDQEETRHV

>tr|Q6CD19|Q6CD19_YARLI YALI0C04477p OS=Yarrowia lipolytica (strain CLIB 122 / E 150) GN=YALI0C04477g PE=4 SV=1

MRLLDNDTFWGQIVYLLSRRRLLRYPEEEPGFVPPTFGDFGVEAVEDRDLEQLENAKRQC

VDSDDYLPSSDTFESDKGMIYVSWYGPNDAANPKNWSLLKKIITGLQMGLLVGCIYMGSS

IYMPAILQLEAEFHIPEVVAILPLAVFVFGYGVGQTLFSPLSEHPRLGRMWIYNLTLLVF

WVLQAPTAKAKNIASLTVLRFICGVLAAPSLSMGAATMGDMFEMRYMTYAIASWAFFCCT

GPALGPLLGGVFYFIKDWRWTFWILGMVSCGGFIILSLTLPETYAPNILHRRAARLRKLT

GNPNYTTIYDEEYAQLSVSQLAREFMLRPFEIAFTEPIVLALDVYIAMLYATLYVWFEAF

PLVFHQIHHFNAIQSGLAYMGLMVGGLLGLVAYCFTTYWAFEKRDADIETFMKLCLIGAV

CYPVSVFIFGWTSHPDIHWIAPIIASGLNMFGAFFIFQTSFNYLGGSFPRFMASVFAGNG

LFRASFAGAFPLFSKALFVNLQTDPKWPVAWGCTLLGCIGVLMIIIPVLLIIYGGRLKGR

SKYTGQ

>tr|Q6CD26|Q6CD26_YARLI YALI0C04323p OS=Yarrowia lipolytica (strain CLIB 122 / E 150) GN=YALI0C04323g PE=4 SV=1

MDAEKLPTPLSGSENPSLKSQRDLDTTSQKSGTLIETNSFKKLRRNTTLAISGQLQMFYV

AHLVARDVIGPAQGGKLQEFNHLSDTQFEWTEAALYFPYATLAVPLAFLFLRVSPRVFIG

VEMFCWGLCIVLLGLCTNYAGLICDRAVMGACMSFALPALLHMIYEHHGRYEAHFVVGCV

WGGSWFMVPLFTMLAIAIRLINAGKPGWSWDFFIAGLCSVVFAPVVWYMLPENFYSPKWI

SKYGEQAFNVFKTLTEEDYGGGNPIEPTETPLEGLISAAKDPLVWFISILGFFTYSCLVS

CYAQVQSATFEELEYTAALGQLIIWTILVFGVIWSSFQGYLVAQKKVLSPFLLMNYLFCI

IGWALILCKPSEHNWWIKFGGGWFTIPNATAAFASLCGWLGSNVQGRNKRLMSFAIFSSV

SCWYGVLVLRVFTSTDGPLFKRGCWINMGFMIASVFGTLGIVFFLKRRRISGTNHATISE

TQKEFVYML

>tr|Q6CDW8|Q6CDW8_YARLI YALI0B20614p OS=Yarrowia lipolytica (strain CLIB 122 / E 150) GN=YALI0B20614g PE=4 SV=1

MLHATQNSSDCLFSELDSDTDTDKPLLHDTSHTVRFCEPQTKRRMGFRKWFSRTSNEKRP

LIDRITTPMDEILTDEPPTTTDALTSPPRGRKYRDHQREEYERRQHYERTRERDRDRDRE

GDKYSVPRRQLQDSARWHEHVRQQQPHIHQAHQIPYQIPYQMPPGLRQHMHPAYLQPRQI

SQTPDAISGYPNQSLLRPMSQVIEPRYIRTEGFPVPPVKYAQVPQGMKAEIMELKDDAGN

DKNEEKKYAKDKKDDKKKESESPQMQSNIEKMVKNALQNHRPAVYETLVRMNSFVLSLKL

ALCIACVAFYPLVGILLIVVLFGSEILLELAIKCGPDRS

>tr|Q6CH77|Q6CH77_YARLI YALI0A11605p OS=Yarrowia lipolytica (strain CLIB 122 / E 150) GN=YALI0A11605g PE=4 SV=1

MLDTTKIPGTSNWLESSEIVKLPVPSDDPDDPLNWSYRRKVLCMACMSAFTLCSGVASAS

IYSILVPISKDSGLPLATLNQGTGYMFLFLGLGCLVWQPLGQQYGKRPMYLASLLLTLAS

QVWSAHAATSNGHWIGSKIFQGFVQAPIESLCEVTISDVWFEHERGRWVGLYAFMLMFSN

FIAPVVAAPIAVGQSWPWVLYWEAIFCGVVSVILFFFFEETNYNRHTKAAVIVTEGNSDI

NSDCEVTEVVNTELAEKKGSFHEGSSVENGHGDLIHYSKKTYVQKLALFDKPRPMMLWTM

FKRPFKLLLFPAIVYSGFLYGSSLVWFNVLNATASLILSSPPYNFGTISVGLSYFSPSIV

AGIAGWGGGYMSDILKMYLARRNNGVSEPEHRLWILVVYLALVPGALVLWGVGAATHVHW

FGLIMAMGLIGGCGTLAATASVSYAIDSYREVASDSMATVIVIRNLMSFGIGYGITPWLA

NQGYGKTFGEGAGVCAGCGLVFLVFVVYGKKLRDLTKERYWKLVQESIDNGIAH

>tr|Q6C574|Q6C574_YARLI YALI0E20471p OS=Yarrowia lipolytica (strain CLIB 122 / E 150) GN=YALI0E20471g PE=4 SV=1

MEKEDIHHVEKTSIDVYEEKVRKPTTAEEMFPNIDKKKLLKKMDMNIIPILSLLYLLSFL

DRGNVGNANIEGLSVDLGLTGPQYNMCLTVFFFTYSAFEVPSNMILKKLRPSIWLPLIMV

AWGIVMTLMGLVKNYHGLLICRIFLGVTEAGLYPGVGYYLTLWYCRSEIQFRQAMFFSAA

SIAGAFSGLLAFGIGKMKGVAGLHGWQWIFILEGIATVVVAFIAFFFVHDFPETARFLTE

EEREFVVWRLKYDGNDNSAGGELVAQNDSRDWKYVWAAFTDIQVYVHLLLYFGVVVPLYG

ISLFLPTIVNNLGYTSSTAQLMTIPIYVIAAIASVTQAWFSDRYGKRSPFILVNLLFMLF

GYLLAFNLSHKKPTAVYVGCYFIALGLYPALPAIVSWMSNNVAGTYKRAVAMAIQIGIGN

LSGAIASNIYRAEDKPEFKLGHGMEIMFVCIGIVSLIVLNFGYHVVNKRKQAKIESGECE

HLTSAELSEMGDRSPYFRYRH

>tr|Q6C5A7|Q6C5A7_YARLI YALI0E19635p OS=Yarrowia lipolytica (strain CLIB 122 / E 150) GN=YALI0E19635g PE=4 SV=1

MARRSFLPESFLWIFPLQQAAELIIALALFNKAYGLYALLCAFITPLSFTQVVMALVSLA

LVFPYAMSIPVIRALPVTGSGPGALASTMASISPSTMSPKDIINKIPIVLLACVLYTFDL

VLTIFSGVIFAHDWAEDCEECDAAAAASTAASVSATLSDAVSATATATATATASVVSSAA

SAATSAVSSAVSSAASATSALVSAATSSALNVATSSVSPDDETRVRFGLRDATDRDLSLD

SASRGQETAATIIIAVFLLLVRIYFALLLLSYGIKLRALLKQLKTNQTQLQTHGHGSRLS

VPEANPLRPWAQSRPSIGGGGIMEPNPGAGDTDPIQGTDFGPYSPDRSVPYYLDSLWRSF

ALKSFDFISGQKTSRDGVY

>tr|Q6C5B1|Q6C5B1_YARLI YALI0E19558p OS=Yarrowia lipolytica (strain CLIB 122 / E 150) GN=YALI0E19558g PE=4 SV=1

MKVSILLAAATAASAIALPQLHRRHEGHDETEATGITNTPHSNDKTNINDINDNNDATSP

SAHSHSNTHSGNSTHDNEDMMEPNPASMNGGHHSHGLPILAHPEKLEPQQLAYWTAYDPT

NFFNTETPGQSLLTWHILMVATAWIGVYPIAVMLSSAKSGLYLPVQTAQVVMVALGAIFL

GMYGSSPEVEKMYPGAIYGKFTVLVLILSLVHYLAALLTSLAYRDTTPNDSDPRQLAEAF

QLQPYHRVSDSDSDHTLNGTPSATPSVPQPSKFRISEDAEDDTPMSPCQYYGDDECGGAE

AASSSSMRPQPFEQKMEDKWVARLMANSHVSSTVDRFGALANFVHTYLQGIMLLVAFAYV

ICGVVTGCRLGMGHNVFNILAHFIKGGVFTVLGLFTFARYVGVFTDKGWAWNLNQEQVNS

VKMLHSRSRANSRGSRRRARSSPPAPTKMTALSVARTAVSKVFGVLAFLKPQSFTFEFVE

SALILIYGVPNIFMEHLASKDGHWTPADLQHASIAFMFIGGGLSGVLLESALVRKLLSSK

TYPMSFNPFPALTIFWTGILMSQHAQALPLSTAIHTQWGYLLAIGAVFRGITYLQYFIVY

SQALARLDGGAEMNVDQLTLPSRPFTEIVASFCLISGGLVFMQSNRETVEAFVYRGIDSM

FTLNVTVGVTSFIMAWELVVLAIKGWASRRKRV

>tr|Q6C5P1|Q6C5P1_YARLI YALI0E16434p OS=Yarrowia lipolytica (strain CLIB 122 / E 150) GN=YALI0E16434g PE=4 SV=1

MLKDLFHRIKYRLLQDAPVGSSLAKLETSWDPRISWEKLRRKRWTWSDWPYVLMVLSLTI

CLSILQTVPAFVRVIVAFVLFALVSIPITSQFFLPGMPIITWLVLFASPTSLPLSLRPKI

YVRLLPALETILYGGNLSYSLAQMTHPVLDILAWLPYGVLHFALPFILAASLFVFGPPGT

LPQFGFCFGWMNYFGVIMELMFPNAPPWYKNIYGLQPANYTIHGSAGGLERIDKILGFQL

YGGTFGASGMVFGAFPSMHSGSACMLALFASHLCPRFTPLFFSYVLWIWWSTMYLTHHYF

IDLTGGACFAYITFFLIRRSVLPRIQRDKISRWSYDYVERGIQTQSKFRKSMEYDREYIE

MADEFGAQGPEHRLEDESDDADDGILPASDADIESIESGVPSTPILETPSSRMGSPMISR

IGSPAPNKQA

>tr|Q6C5Q8|Q6C5Q8_YARLI YALI0E16016p OS=Yarrowia lipolytica (strain CLIB 122 / E 150) GN=YALI0E16016g PE=4 SV=1

MKTILKITKSENQNALFKNPISPPHPPQTRTPSLKIKVQPQIPHFFHAGPYINRGCPFLS

PLLHYHLVEIPTTMTAGLVAAAAIGAAYLEAKTLISEDAYMIRGAMTNGLDFFYNAWKGR

VQYWYAFEDAVKKYPNNPAIVYPKPIEGKKPSGDSYDDLFDVETFTYQQLYDEVLKMSHL

LRNKYGVTANDTIALNAMNSPLFIIVWFAIWNLGATPAFINYNLADKSLLHCLKVGHASI

MFVDTEVEGNVRPSLAEIKSEAKCDTVFMDDDFLAAYAASPAYRAPDYERHPEQKDYDTA

VLIYTSGTTGLPKPAIMSWKKAKLMSSLYGHSIRLKNNGVVYSAMPLYHSTAAILGCLPC

LNRGAAYAPGRKFSTTTFWTQAKLTNATHIQYVGETCRYLINAPPSPDEKSHQIKVAFGN

GMRRDIWVKFKERFNIPAIGEFYAATEGPLGTNNFQQGEIGIGAMGRYGKLLAAILATRQ

TIVPVDPEDETELWRDPETGFCRVAQSDEPGEFIQKIPNPEKVHETFQGYLGNDKATNSK

IMRDVFKKGDAYYRTGDLVRLNDEQCYYFVDRLGDTFRWKSENVSTSEVEEHVGASDPNI

EQVVCVGVKVPEHEGRAGFAVVKLKDASVKPNLDQIAEYSLKQLPKYAVPLFIKFVDEIE

RTGNNKVQKVKYKNQKMPHEEGESPIYWLKGNKYVELDAGDWASLGSGKIKL

>tr|Q6CAT5|Q6CAT5_YARLI YALI0D00132p OS=Yarrowia lipolytica (strain CLIB 122 / E 150) GN=YALI0D00132g PE=3 SV=1

MVFGREKDDSEGIEHVPSPQDNPSDQTSDIIALNEKASNEHDDLPTIPKPEGDAPVNSEL

DPDNPLIRYSRAELLEIATQFAVDNDLADKAEAFRKGALVAQDPSGFENIDILDDDDRYW

LNREITNKWDHPMKVYYLVVCCSLAAAVQGMDETVINGANIIFPAQFGIKEDSGVVSRKS

WLLGLVNSAPYLCCACISCWMTDPINKVLGRKWTVFWTCFWAGATCFWSGFVNTWWHLFI

ARFFLGFGIGPKSATVPVYAAECAPPRIRGAMVMMWQMWTAFGIMMGYVMDLAFYYVKDR

GTIVGLNWRLMLGSALIPALLVCIFIVKCPESPRWHLARGEIRKSFECMREIRHTDIQAA

RDTFYAHVLLIEENEMKKGKNRFVELFTVPRNRRAAWASFIVMFMQQFCGINVIAYYSSN

IFMESGFGAIQALLASFGFGAINFVFALPAVYTIDTFGRRALLLATFPLMAIFLLFAGFC

FYIGQNDPTHSHARVGLIALGIYLFSAVYSCGEGPVPFTYSAEAFPLYVRDLGMSFATAV

CWLFNFVLAVTWPSLLAAFTPQGAFGWYAAWNVVGFFLVLCFLPETKNLTLEELDKVFSV

PTRVHMKYQFNAFKINIQRTILRKDVPKPPPLYAHEAGIGGTSHWSSKPQPNANTAEFV

>tr|Q6CAU0|Q6CAU0_YARLI YALI0C24354p OS=Yarrowia lipolytica (strain CLIB 122 / E 150) GN=YALI0C24354g PE=4 SV=2

MDSYNKFGFNSETGEPNTGPQDTSGSVPSEHRGDGSFSPDIGRKKSLIRPDRARLDPDSR

TYHYSQLAAQQQSRINVLPSSTGNDPILEEHYMRETGEIQPGQGYDQPYDNAVNQEVLEE

ENEESYREGEEDPYAYSGRQVYGLNDEVQTEAYVRKSDEISSSEEQAADTQPPIIDPPVN

EKVQLSRGGGTLKRHNSVKRRMGTQPKQISLWMTYCQIVTFWAPAPVLKMFGMPQKARQD

AWREKIGLITVILYIAAFVAYLTFGFTITVCSSSITRVRNGEVNSGNLIINGKQYDFTSM

NHPPTVEVPTGGNFMYPPVNAGSMDGSLLFQNVNNHCKGLITPKPDCKIPYNGDSVAWYM

PCQLLNQDGSSQPNTTEQFYPGYACHTSTTARNVYYHQLVDAGEVYFTWDDIKNSTRNLA

VFNGAVIDLNMVNFILKDDLNYPEIFDTLRDDDSIKGTDISRNMANGPDRQAARCLQEIA

KVGYIDTEQIGCLASQVVLYVSLVFIISIVAVKFLFAIYYKWAISAKQGAFPIDNKQMNQ

RENEIEDWSENIYSQAPIKRVDRDQPKSSATPFRGLIKHRFNQHDNKNKRMSTMVTQVGE

SGTAPSVYSRNSAAIDSNTDVGGVVYDYDSGSQPYEAPTVNVVPQPPIDYQPFNYPLAHT

MCLVTAYSESIDGLRTTLDSITTTDYPNSHKLIVIIADGIIKGSGNDMSTPDICLSMMCD

MPIPEDQVRPFSYVSTSSGTKRHNMAKVYSGFYAYDDATVPPEKQQRVPVVTIVKCGTPA

EANEAKPGNRGKRDSQIILMSFLQRVMFDERMTELEHEMFNGIWRVTGILPDFYEIILMV

DADTKVFPDSLTHMVAEMVKDPEIMGLCGETKIANKRDSWVTAIQVFEYYISHHQAKAFE

SVFGGVTCLPGCFSMYRIKAPKGGDGYWVPILANPDIVERYADNVIDTLHKKNLLLLGED

RYLTSLMLKTFPTRKQVFVSKAVCKTIVPDKFSVLLSQRRRWINSTVHNLMELVLVKDLC

GVFCISMQFVVFIELVGTLILPAAISFTIYVVIIAIVRKPTPVMSLVLLALILGLPGLLI

IITASRWSYVVWMFIYLLSLPIWNFVLPVYAYWKFDDFSWGDTRQVAGEKKGSGGHDEED

GEFDSSQIVMKRWRDFEMDKRRYVPLPPQNAWMSDPHMPSYNHQGFSANSSPALRPQGSS

HVYSDSVAGASDYNVAQGHEVL

>tr|Q6CB50|Q6CB50_YARLI YALI0C21912p OS=Yarrowia lipolytica (strain CLIB 122 / E 150) GN=YALI0C21912g PE=4 SV=1

MLISPAIAANWATMVLRQMQVATSVVYILSLDMKDAKTHFDKGIKVVEAVGILAVGIGPL

WRKPMVSAAKLQSWKISPYQFTAAILTLSTVLVSLAAVFLFVVKNYFLVVIVVFVNSVLV

NEIKWAEAYFSTNWLPKNKYLEMFEKSKNIILLFTIFPAMIATWIRTVWLFVPIPVALLL

ISYFLAFKLERLLDQQVKYKQMLEDNAEYTPLAEWNDPNSYDYYKIDIKQESWGQFYTYY

FFQFL

>tr|Q6CB67|Q6CB67_YARLI YALI0C21516p OS=Yarrowia lipolytica (strain CLIB 122 / E 150) GN=YALI0C21516g PE=4 SV=1

MFRLLLNSILLIAAVIIGVANYNGLPTSALDVRDSLLPMMWRYSGIEPVNNNCHVVEGVE

ACEDAKYDEESGLVFMACGNAEARKKWFPGTGFFMDPETARHQVDQIFVFDPKTEKIQNV

HVKDFEGHTFTSHGLDVFTLKDGTKIIAAVNHKADASVISFFKVNHPGEVAYIGEIKDDL

LKIPNSVALFYTPEGQLGFVATNDHVEREGPKRQVAEILGLKATYLVYCAVDIDNGVTGK

CHKVADHLVYPNGIAHIPGTHDFVQSDSRDAKIKHWSWNSTSQKLDLNSATMVGAPMDNV

RVIPGTKDVMVAAFPNVLQVMHKYKNMDDKNSRVKTVGLRLNHNEGYKIPHVIHKSNDDY

GVFVNTVYNYFPKENKILAGSCFAKGLVVCNGPDGAQAQKIVEEEVNKNVDIDEDEEEDE

D

>tr|Q6CB68|Q6CB68_YARLI YALI0C21494p OS=Yarrowia lipolytica (strain CLIB 122 / E 150) GN=YALI0C21494g PE=4 SV=1

MASQESHLDTIEFIETPAAKPQPPASTATGVRTTDYPCIKNAPLPADGPGARHFSNGLLF

SILLGVPFFINYKIGGSIWTYLFFFLITGLPLLVSFWAVVSAISPRINDDCKLPGRPVEY

YLEFHDPALAKKYSGNNKIPYETFHELYFDNKVSMRGDALDILEYRHDFMTFNFTWGLFK

FFLLSMIPEIIFHTQSQDEEQVREHYDRGDDFYAWFLGPRMIYTSGVISDINKAETLEQM

QDNKLRIVCEKAALKQGEEVLDIGCGWGTLARFASQEYGVNVTGITLGKNQTAYGNKNLA

KDGIPTTQSRILCMDYRDIPLAQGGKKKYDKIISLEMVEHVGVKNLGSFCSQVYDMLEDD

GTFVLQYSGLRKHWQYEDLNWGLFMNKYIFPGADASTPLSFFLDKMEGAGFETVSIDNIG

VHYSSTLWSWYRNWMGNKDKVINKYGVRWFRIWEFFLASSVITSRQGGAAAHQYVFRKNI

NKRHAVEDIPTHWGLKVPQPKHGNNWLPEGAY

>tr|Q6CBC6|Q6CBC6_YARLI YALI0C20053p OS=Yarrowia lipolytica (strain CLIB 122 / E 150) GN=YALI0C20053g PE=4 SV=1

MDSDHGGYHDPHDSHADSSDVTDSDNRPQNLRINKIKKPLFRRFQSEQTVTTSSASGWDA

NDTNDTNDSENDSATSPAKHKRRPSTRAQLKAPQTQSQPQSPVLKSKHHGPQHNSLRAVS

NGTKRLASRFHLGNFSLRSPKIQRKHSSYFTDNEDSDDERLVWGDRDDTQPLMSQSDYGG

QFSYGSVEFPNPPTLKLPPADHTNHGIVHKASQFMQSRVGIQSMMSDTGPERRFYDDFTT

VDWVRDTINDSSRVKYIQSIPGFRGRLIRSFDSLQDWILISVVAASFALIAYSIDRVEET

LFDFKFGYCSSSWLSPRGECEGEWMLWSDLTSFEIPYLSAHHFNFLVYLLIMLLLAFLAV

RLTLRTKTSSPISLKDNKPRVFYTAYGSGVAEVKTILSGFVIRRFLGTHTLVYKSVGLVL

AVSSGLCLGKEGPYVHLATCVGNIACRLFTKFSHNDLRRRQILAAAASAGVALAFGSPLG

GVLFSLEEVSYMFMPAQLFRVFFCAMTSALFLKLLDPYKTGKIVLFEVKYTQDWHSPEIF

VFVILGICGGIFGALFCKFSAWWPQKVRAPGKIFHGHHTIEVLVVTLITGLTSFSSPFTR

QSVAELLYQLASPCDPDNPALSKLCPTSISEIPGVAKALSWVLVLKIFLTCITFGIKVPA

GIYVPSMIIGALFGRVLGLGTQLLYHQMLNEKESGVLSIFATPFLGTLATCPGGALQCIT

PGTYAMIGAGAFMAGVTRMNVTLAVILFELTGSLDYVLPFSIAILVANWVANLIEPKSVY

ELQIKKNDYPFLDNRKTLAFDSSLADLVSHFPRKLCIEMDEGDEDGSAKVTVGQLKEMLA

QVQVRNGIDGSIPLVRNDNVVGLLPALELEVALDRISQWYEEVAGVVVAESEASGLRSPT

STETTGLKLSLPIPPTFPPTTIVPPSSLDSIVCTVSVKDVDITKFHHYYVGVKARETCET

DPEETSNSSSCEDGLLPFEATDLTQFVDRAPLALDVHSPLTLVQMMFSKLGVRQICVVED

GQFVGVLHKKKFIDFCNNQEK

>tr|Q6CBI9|Q6CBI9_YARLI YALI0C18359p OS=Yarrowia lipolytica (strain CLIB 122 / E 150) GN=YALI0C18359g PE=4 SV=1

MSQRPTGLKIDMPESFSDQMAKAKKYGNLGANSSGPSSAGPMRTPSWGILNVPELLNDFH

HHTLESKLDPVKEGYDEALPMRSRRRTLVNGEIRNSEDREEAAVELDTLKQECDHVIHNH

DCDLNEPRIPVYDPLGHIHDHTHDHTHDHIHDHIHDHIHDHTQDHIHDHSHAHAHTHDHK

HTHDGHDHSHDHNLTNTHTHTHDHGSCSHGHGHSHSHDPREIPDLQIHIPPLSSLLQSIP

SFISLPTSLLATSLLCLQLNVLSPIVSALLVAGCTTFWAGVFRLFGIKHTIGTRKGARNT

VVLTAGIFALVISAIFLGTTRVTAIVFVSLNVPLKKVPEWRSSWQYLVYLLISIAHDVVV

EGGPLLDFAIGYLALGVAVFVLQQNLETPSSQAIPLGLLITIPTALFVSLTIEQIILLVV

ACGSCGVFLIAKRDLQPNRINGVFSSLIGFTLDLSIKSRATPSEALVCGLSIFVPTLVSE

DGEDASDKSIKKRPGLIDSIISHSDTRNIFYFLLLNFSFMLIQLLYSILSHSLGLLSDSI

HMFFDCLALMVGLVASILSKLPPSSRFPYGLGKVETVSGFTNGCLLVAIAGGVCIEALGR

IYNPVELERTAELLVVSALGLLVNIVGIVVFNHGHAPGEECSHGHSHGGHDHSEHNSNTY

GIYLHIMADTLGSVGVIISTILTWYFGWSGFDPLASMLIAILIFLSALPLVSTAAKTLLL

SLTDSQEYTIRNILNDISVMPGVASYDEPRFWSDGGAVRGTIHVKLQQDASSKQVKQTIL

QRMQQDHVKDVFIQIDA

>tr|Q6CBR8|Q6CBR8_YARLI YALI0C16181p OS=Yarrowia lipolytica (strain CLIB 122 / E 150) GN=YALI0C16181g PE=4 SV=1

MKAFSTLTAVLLALVALLSFASAESFDIKYSVQDKALITPKSHPSLFSFTGRWYKRRDTF

IASNWPGTYVSALVYGKNVTLILKPLNTGKVTNHKFSYSIDKGRIQEAAIQEYDSGISST

LELTLSVPETSQPDKNVLVPRLLTIYSHAETPLRLHGLYMGNVVINEGKNWAETQHDIPR

IEFVSNYNTSLATPNIFKELAFTVPEQLGVHHSHVTADICLAPDCYPNKYAMMEQYFYFH

YFDGVRPKRGVDPKPAFFQFDREDVFLPLNEPQVIVVDIGDMDMKKNIDLVTFGEDFKKF

LASIMVQAYHNINHIFVLVKPGRYEQATYKAIEEIPSSRITPVYYARETPDWWKGFMCSN

VIPATGKPFPYDSLCGNKSFTFSTKGVLGHSNVWIMLAGIIAISGGGFYFIKKRGGIYQG

LPVSKQDK

>tr|Q6CC13|Q6CC13_YARLI YALI0C13574p OS=Yarrowia lipolytica (strain CLIB 122 / E 150) GN=YALI0C13574g PE=4 SV=1

MSKCNDSKSLEKTFPDPADEKLDVEVGEVNDITLDAATDKRLLRKIDMYLCPVMMLVYAV

QFMDKSTNATASVMGLRTDLHMEGNDYSWSGTAFYLGYLVFEFPAVYMLQRLPFIRTLSA

FIVLWGVVLCLHATPNYPGFIALRTLLGMLESAVTPAFVIITSQWYKREEQFLRTSFWVG

SNGLGIIVGCLMAYGLAINHHLPIHGWKLVFIITGVITIATGILILFHFPDDPSKAWFLT

EEEKKLVVIRVRTNQGSFANKQFKRNQFIEAFKDPRSWLYFFYSIASNIPNGGITSFGSI

LFSETLGLGPVRGLLLQTPQGAVEIVGCVLFGLLAQYTQWRLFWSIVAQLIATVAMCLLA

FLPHIQAAGLAGLYLLSVYPVGLVCMLSSVGSNTTGHTKKVTVNAIMLVGYCVGNIIGPQ

TFRADDAPEYVPAKVSMVVLFAVAVGLNTVLLFVNLAENKKRDREGHINPSLEELEEIQN

SDMTDQENRYFRYVI

>tr|Q6CC53|Q6CC53_YARLI YALI0C12408p OS=Yarrowia lipolytica (strain CLIB 122 / E 150) GN=YALI0C12408g PE=4 SV=2

MDTSDAPTTRVVYHEIPAPGEPPYSNIVALFFLVVFFLGSYLLNRAFHSAPKLWIVFLDW

AETTRPGSETQEATGKARQTGPANWMDIDSLSDAEMKEVMEKMQLIKSNAIRLKQEAKQR

MAEIQHELQYVAGMH

>tr|Q6CG94|Q6CG94_YARLI YALI0A21120p OS=Yarrowia lipolytica (strain CLIB 122 / E 150) GN=YALI0A21120g PE=4 SV=1

MDDNYLKFSLLSVITRDLVTKGQVQGTGTTVRFPPLKKRKISLGKWFKRLSGEERLFLSM

AVGGFTLANFQQLFSILWWSNKSVLKRYTHKRLIGTLVKLYRLGCFNLALLFIGYYWYRI

FFANDGKKERDILADVNLNNEKNRGPIFSDTPEKIEQGGSILHAVLKVEGEDLRVSYELK

GSCTVIISVPSRKIVSRVDCFDSKHFEKYSRLHAIDKDAVVTFYERDQDKCYFYIHGDQY

TETSGVEFAKCGDKYEGITSSAL

>tr|Q6CGA8|Q6CGA8_YARLI YALI0A20812p OS=Yarrowia lipolytica (strain CLIB 122 / E 150) GN=YALI0A20812g PE=4 SV=1

MSLHNKFPYNHPRDVGVPTPSPSRNNTALLAASAAFSTLAFMAAAYSLMDTNDIQANPTR

IVCCLGFFPTTIVMTRVVTLSWRSRAAFCLAVSCVNALIATIIMTLCDPLLGHVFLSLST

AGTVCYYKAHIVDIHDDQEGPCSTEDSYQAAALVFCYMACAVPLWTQIGCAHVMLIVLNC

GIMYFLTVRPTIISRILALLVIILSSLYLMCTSQGPAGGLHLVFMAIAAELTIDYSKYNT

LGEKKSTHDTLAFLCFICGLATLGVALNESQPREFLTVYLAVFLSLTVNAMSATRYSDKI

VHMVHTVLWSWLVSVYMMNQSYHHGIIHAMLSVTTICIYSEMDLPERDSKVRETPLRYIA

STVCIIAGLLTIDIWTKHVLDLVLEPDNVVMLNIMAGFFQMLFCFAPLCILDENHEGDIG

HQDALLLFLGVASIQIGTGVAQVYFISYFKGIVVLALSLFSVIICWAEYVKASSPENTDA

GGCEKSMV

>tr|Q6CGG5|Q6CGG5_YARLI YALI0A19558p OS=Yarrowia lipolytica (strain CLIB 122 / E 150) GN=YALI0A19558g PE=4 SV=1

MTVIERKSDDSLQEKGVPYATQDECILADIGYKEELKRHFSVVQVFGIAFSIMGLLPSIS

SVIGYSLTAGPVGMVWGWCIASACIMVVGLAMAELGSSLPTSGGLYWWTYHFAPENAKRP

LSFLCGYSNSLGLIGGLVSIDYGFSLMLLSVVSLATDGEFEPSKYTVYGVFAGAVLTHGL

AGILTTKLISKIQTACIVLNIGIIVLVVIALPVGARDHLNDGKFIFTQIENISEWPTGWN

FFLSWLAPIWTIGAFDSCVHMAEEASNASRAVPIGIISSIGMCWILGVIVNIICAAVINP

DVEAIINTPLGQPMAQIIYDCLGKKWTMAIMSIIFCLQWTMGLSILVAGSRQNWAFARDG

ALPFSDWLKVVHKETGVPRRTVIMGTFVGLAIGCICMIDDKAAYALFSLPPVSNDLAWLL

PIFLKLVFGASKFVPGPFYLGKVLSKIIGIAASGYLVFAIILLMFPTATPHVTTDTMNYV

VVLNVGVWIGALAYYFLYARRWYTGPRSNLEDDPRVLDAVRVVNQELAKEESEKANEKQV

EEHVERVVI

>tr|Q6CGJ2|Q6CGJ2_YARLI YALI0A18832p OS=Yarrowia lipolytica (strain CLIB 122 / E 150) GN=YALI0A18832g PE=4 SV=1

MSKHLALERVRASMDSTRTPTPSRPASILTKEKGEDIIVEVTDSCSVQEVYSEWGAYTDE

HNPEGLRTATDSDKDLRHIAGPVGKMTYLLSIVEFAERGSYYGLVNVISNYVQFPFPEGG

NGWGASPVGTQLTPGALGKGLQIATVVTLVLQFLSYVAPLWGAWLADARYGRFNTIWAGT

IVCGVGHVIIVIAGFPGIIEHTNASFGVFMVGLLVFAIGTGLFKPNILPLLLDQYREKAN

WVKKLPSGEEVVIDRDATLQRMTLVYYWGINIGVTLGIATAYSEKRVGFWLAFLIPTILY

FLLPPLLWYVRKDVYKVPPSRSSVLSGFYGVLYQRIMRKPPTYSEEFVFDVNSTLKACRF

FLFFPMYWINDEGIGALFNSQGASMLTNGVPNDLLNNFNPITIIVCIPIVDYIIYPTLRR

LKINFRASFRMFLGFMLAAASSGAGAIIQWRVYETSPCGYYATECPEGVSHITIWWQVVP

YFLAAVSEIFAVITSYELAYSLSPAGLESVVMSLLLSMSAVSTAIKTAITPALKDPDLIW

PFVGIACGGVGFAVVFLICFWRLGKDQVRPLT

>tr|Q6CDL2|Q6CDL2_YARLI YALI0B23078p OS=Yarrowia lipolytica (strain CLIB 122 / E 150) GN=YALI0B23078g PE=4 SV=1

MSAPPYDNPFEVGEYDNSDSLHRSGSTRSQGRRSSRYEYQYTKPLPGAPGQSQDQGFYSA

GGFSGAHHQIHPDKGDLPPLPGESQEYGDSNLRTSQDGNSLASYGDSFNGHYKASSQTSN

WYEDVETTRLMRTHTQTSRMSMNSNKPLPDPLDVANPRNSRIYGTNYSAYRGVTPAPPPK

DHFVMEQFEIEMPDDEERGREDGRRRRRKRRTPSEEEARQKRKQRRHRARRYESRQTQNH

PYFTWFMTTVHIAVFVAEIIKMGVLTGIPIQTQPSFNPMIGPSNYVLINMGARFTPCMMY

IKGVTDDPTLLFPCPNSTTLETDKCTLEELCGMGMKQTPGSDGGSITSGGQWWRFITPIF

MHAGIIHIGFNMLLQMTLGADIEKQIGIIRYFFIYFACGIGGFLFGGNYTPDGIASTGAS

GSLFGIIAIDLLDLLFNWSIFRNPVRILIIHIIEIVVSFVLGLLPGLDNFSHIGGFIVGV

LLGIAILRSPLKVVDEGTSLFNQGMSSEEQARLRRRQLIQQEEDDKNHLLAVFPKSRDQL

DRDIEQFKSRPRRWYIWFLVRLACLALVGVFFGLLSRDFQNGGGDCHWCKYLSCLPVNGW

CDLGNITTGDN

>tr|Q6CDS5|Q6CDS5_YARLI YALI0B21560p OS=Yarrowia lipolytica (strain CLIB 122 / E 150) GN=YALI0B21560g PE=4 SV=1

MYRFLWIVPLFACVVWWGMLIALLAAWAAQGKPIYDFMDPDDKVAYISDVGATSLKPVFI

ACAATQGGLFMFALIFERWMRHKGRLLRNHRQAEKWLSVGAIIFGILGQVGIILVSIFDT

KNHHSVHVGCLVLFIVGIGISAILNSAEFTLLDQNYPDVRRLKTSYILRWIWVAIAIVLA

IIFVSCNNRNEPNVAAGFEWALSFFYGFYLLILAFDLIPFKRYQERNAMATAPPMAEYGY

VQQPNDPGYSPTVGGATMVPPQQQDKPFDAASYATAADGFAAAPPNYNVAKPEPSAMYH

>tr|Q6CDT4|Q6CDT4_YARLI YALI0B21362p OS=Yarrowia lipolytica (strain CLIB 122 / E 150) GN=YALI0B21362g PE=4 SV=1

MSDKRRADLCVPYVHVVDKQNDAQSFITTSLPMAAMFLRNKLMSWTALFTAITAFMNEPL

IKPTQSDGASQPAWLSVLVSLVGLFTCYMDLAFPSQGRKLAAQHAASSTASAASSTASAA

SATVASVASKATGVAADALDKLFKKAA

>tr|Q6CDU0|Q6CDU0_YARLI YALI0B21230p OS=Yarrowia lipolytica (strain CLIB 122 / E 150) GN=YALI0B21230g PE=3 SV=1

MSSISSSQQPNFKSILQYNARQEMIYWMAFVACLGTLQFGYHVAELNAPLDVIICHDAVT

DCIDLHPRDVGYVTAAFSVGGLISALLAGMAASKYGPKKVSLFNTLSFIAGPLFMANATN

TNTLAFGRFVSGLGAGAAIVVTPLFLNEIAPHNLRGMFGALSQISVNVGIVAAQVAGLII

SQSWRYILIIGFFLGLINLASLAFIPESPKWLVSKNRATEATAILARLRDNRATAAREVE

EWQKELQSVMFESDQLMAPASAASANHIEPVSLSAFLTRKPYRKPLLAILIIMTAQQLCG

INSIIFYGVSILGQIIPKYSTLVNVFISVLGTVVTVGASRFIDVLGRKRLLLYSIFGMSV

SAALVATGIINSWPIVSSLSAALFVVSFAIGLGPIPFLMVSEMVEPNCVGVGQSVGMTSN

WIVTFAVGYFFPMVNARLGGATFYLFSVFGIAYLTLVVKFVPETKGKRNFTEVWTM

>tr|Q6CDZ2|Q6CDZ2_YARLI YALI0B20042p OS=Yarrowia lipolytica (strain CLIB 122 / E 150) GN=YALI0B20042g PE=4 SV=1

MRRKVKDRIPWMDIGGAKTGINTWMDCARIIRARTQSMKDEDKEATVVQYSGLLLHYCTN

TVSYWILLLFKIYVLNLLFPISRMSFKFVLDLVKGRERKELKRLKV

>tr|Q6CE14|Q6CE14_YARLI YALI0B19470p OS=Yarrowia lipolytica (strain CLIB 122 / E 150) GN=YALI0B19470g PE=4 SV=1

MPITVSQEVTETAPEVSWAATKTYLGSRVSTLKPPKLSAQEKRCLNPIYVLRQLGKKQWL

FFSVAILGWIWDAFDYFSVSQTATEIAKDLDMSVADITWGLSIVLMLRSIGAIIFGLASD

RFGRKWPFIVNIAIFSILELGTGFVQTYTQFLALRALFGIAMGGMFGNAAATALEDCPPE

ARGLISGFLQAGYDIGNLLCVIFTRAIVPNSKHGWRALFWFGAGPPILIMVFRAFLPETD

TYIASRINKEDNSTVEIDPETGLHMQPAQKVGTWASIVIFIKGVGHTLKVHWLMFTYLVV

MMAGFNFMAHGSQDLYPTLLKNQLKFSIDRSTVTNAVATLGALSGQVTIGHLSNVFGRRL

SVIISCVIGGALIYPWAFSGGGAGINASVFFLQFFVGCWGIVPIHLSELTPPALRTSLVG

VAYQLGNLASAASSTIEAKIGERFPILDEHGNHLPEEYDYGKVMAIFMGCVFAFTMIVMF

LGPEKRGSDLCAPQYEVTDAQTAAGDEKFEDKEKVEEVAIERIDTNLTR

>tr|Q6CE63|Q6CE63_YARLI YALI0B18260p OS=Yarrowia lipolytica (strain CLIB 122 / E 150) GN=YALI0B18260g PE=4 SV=1

MLPQYLINVATWSTFIAIFISTISILLQLYTYTRPADQRLVIRILFLVPLFALSSWLSLL

ETQDQISRPLARFNIVLSALKEIYEAFTLYTFFSLLTNLLGGERNIIFTTQGRAPLHTLF

GKVNISDPHEFLTVKRAVLQYVWIKPVISVAIFICKILGVYKQGEISLTSGYTWIGIVYN

VSVSLSLYALGIFWMCLHTDLQPYNPWPKFLCIKLIIFFSYWQGVVLALAQLMGIIQPES

SAPLQDWFMCLEMTPFALLHMWAFPHDEYCQRDCGFARLPVWLALRDVLGIGDLMYDFKS

TFWGKGYTYRNFDSVESVIVDHPDADSRVRKIMSGLRYTDGGRGKYWLSDESRDDHEGSG

DSSSSSRDATEHSGLLAGQQSHVAASDQLLQIASIAEIDDSFDQWRNEDDSSKARDYGAT

DLATHIVEPVSEQELYDDDDLYYDAREQRFGDFNYRVKTVKEQRTWRSEHNGGLRREL

>tr|Q6CED2|Q6CED2_YARLI YALI0B16588p OS=Yarrowia lipolytica (strain CLIB 122 / E 150) GN=YALI0B16588g PE=4 SV=1

MYSSIWTVLAILALSASLIFNPADQPLLSSVGFSLIAYLVTYNLIPALGPAFIKVGFSGR

DLSKKDRPEIPETMGAVCAIVYLFCMFLFIPFVFYQYLAKTIGGGVVVGYDSLSEGLVKG

RILSAFPHEKLAEYLSAILCLQSMFILGVADDLFDIRWRNKFFLPAIAAIPLLIVYYVDF

GITAVMVPVVLRPYFGDIVDLGALYYMYMAAVAIFCPNSINIYAGVNGLEVGQSVMIGVC

ILLNDFIYLVQPNHPAEESHLFSVYLLLPFLAVSIALLRHNWCPAKCFVGDTYCYFAGMV

FAVVGILGHFSKTLLLFFVPQIFNFLYSVPQLFKIVDCPRHRLPKFNPETGLLEPSRTYF

DKKKPGKLGQIMIYVLEKLHLLKVWRDEKGVVISISNMTLINLVLVYVGPLREDHLAAVL

LGIQFASGLAAIALRHSVAAMIFGRDNI

>tr|Q6CEQ1|Q6CEQ1_YARLI YALI0B13948p OS=Yarrowia lipolytica (strain CLIB 122 / E 150) GN=YALI0B13948g PE=4 SV=2

MTFLNVLYYVLLAAIMIGTGYFYYLWFTETNDQTEKIIRAALGVFDIAIWYILGISTSFK

ILTQMILACFLVVLAGLKIYINPRVGGALLAGSLLFVAAVWFGFRRDGREARDDLNDGQ

>tr|Q6CEQ4|Q6CEQ4_YARLI YALI0B13882p OS=Yarrowia lipolytica (strain CLIB 122 / E 150) GN=YALI0B13882g PE=3 SV=1

MKFVQLILCAIFAAVVSATAFQVKVKPQELQCFNAQVQRAGQKILFYYAVQKGGSFDIRA

RVKTPSGQISYDKTAKMDEYVMTSDQGGEYEFCFNNHMSTFDEKTVDFEIKLEHEELRAE

LPADINKERPEHASIENSLNKISPKVDSILSEMRYLKVREARNKATVESTESRIYWFSVL

EIVLMVGISVCQVTIVQVFFRGSRKQLV

>tr|Q6CET4|Q6CET4_YARLI YALI0B13134p OS=Yarrowia lipolytica (strain CLIB 122 / E 150) GN=YALI0B13134g PE=4 SV=1

MRLTSLLALATVALAGRRDKPGDKMLVDTSAVAGALSKSAKFGVDPGETNLEYYNNYWAC

PQYMATLMIWYRAVVPDEEQWKKVLQSDIVETYNEYGKMHLTLDEVVAVYENGTKYVETP

KNASAIVYAPVPFNQTGYDEYYPTVKEFYKQLDLGTYYGGGLLAYWLAIFLIGMFTNLAK

HAFFGIYQKMKGHRFRKHVSLPALWGYKHCQTSGGWLGGFISMCTPTRAQSLAVAGYLIM

AFIFCFTQFDLFTPNYYFEANGDQLARYLADRTGIMAMTQIPIVFLFGGRNNFFMWLTGW

SFDTFNIYHRYSSRVMVVYAIIHSICYSYIERGSYFAIASSQFYWIMGVVATICGSLILF

QGLHFFRSRWYETFLVIHLVLAVAFLVGMWYHCRTLGWMQYLYATIAVWSFDRFARIVRI

FWSGFVVSGDFELVDPDQLIVKAEMDYSKWWSIYPGVHVYIYILNGKFWESHPFTVYQSP

FAEETGKMTVLLKAKEGKTLSLANLLAKGDGKRRLKTLIEGPYGSKHPIGKYDSSIYVAG

GIGITATYSYAVDVIKKSTAKTLVFTWVVRNETCLTWFKTELDTLLADPRVEVNLYVTGN

HTDIPPLGGSESDSKNSEKSVSEMNPRLNIVYGRPDMTVEMPAYIERCAGSTAIVVCGPP

VLNDDIRLSLVQSADCSKVDYYEEAFSW

>tr|Q6CF22|Q6CF22_YARLI YALI0B10956p OS=Yarrowia lipolytica (strain CLIB 122 / E 150) GN=YALI0B10956g PE=4 SV=1

MGKLVDKLMKVINFLISQWFIIGMGVAVAIAYAAPNYARSGGMIRSDITIEYLAVAAIFL

ISGLSMPSRTLLKELGNWRAHLITQGLSFLVTPALMFGFVKAIYAADDPRIDKYVLVGMI

ICGCTPTTVSSNVVMTRNAGGNDSLSLLEVTIGNVMGAFVSPALLQLYLSDSTGFGFGNP

AKDSSMTELYRRVMKQLGLSLFVPLFVGQVVQNVFPTKTKWVVTKFKLAKLGTFCLLLLI

WATFSTSFYDNAFESVPKATMIMVPFFNIGIYLLFTVICFACARSPIPRLLAPHPPTEVS

SRPYKLLHRFISGFYFNKRNTVAIMLCGAAKTVALGVPLINAQYGSGSSLVGRVSIPLTL

FQGEQILTAQLLVPIFKRWIGDEGKEPENEFDDITVNSKSSNDLEKAMPAITAAPVSSPP

AGKDKSAVTSTAVDTNTTGTHTSDFIFESESNPDKIRQDEEVEAKM

>tr|Q6CF79|Q6CF79_YARLI YALI0B09537p OS=Yarrowia lipolytica (strain CLIB 122 / E 150) GN=YALI0B09537g PE=4 SV=1

MDKSTIEKTEYSGSIVDLPNESLSSTQSASFEDHEAQKEGGHTTKRALKSRHVQLIALGG

CIGTGLFVGSGAMLSNSGPASIFIAYIIMSFVIYVVMNALGEMTTYLPLAGAAPPMYVNR

FVDDSLAFACGWNYWYAYAILVASEVTAASIVVEYWTDKVPVAVWITIFLVSIVILNIIA

VSVFGECEFWFASIKILGIIGLIIVGIVIFFGGAPTHDRLGFRYWKHDAFKEYIVKGDSG

RFCGFWNALIRSGFSFILSPELVTISAGETEAPRRNIPKATKRFAYRLIAFYVLGSLVIG

VIVSGNDPRLLGAVNSGGEDAAASPFVIGIQNAGIPVLNHIINAVILTSAWSAGNSFLYA

GSRTMYSLACHGEAPAVFKRCTPNGVPYLCVAATAAVGCLAFLNVSSGSSEAFTWLSNIS

TISGFLAWLCVLTAMIRFRKAIWFNHGTFDVLPFKTPFQPWSTWFVLCLVALMCITNGFT

VFVGGSFTGSDFVAAYITLPLFAAIYVGHRYIYKRAGFKTPFARPIDEIDITTGLKEAQE

VEDMFPERLPRNWLEKVWFWIA

>tr|Q6CF97|Q6CF97_YARLI YALI0B09009p OS=Yarrowia lipolytica (strain CLIB 122 / E 150) GN=YALI0B09009g PE=4 SV=1

MALLTLLACVCSFVQADDNLHSTGAYPCSDQDSPLSVSNFSFIFDRNSGNVTYNVSGHSN

IEVSLEAHITVVAYGQQVLDQSVNLCEEGVTELCPIRPGPVQASAMYAVPEKYTRGIATI

AYKIPDLEGLITIQLYNADTGENLGCFRTEISNGNSTQTDSVKYVTMGIIAAALAAAAAS

SAGGGGGAGGAGSGGGGAGGGTGAAAGAHGAAPAAAQGPAGGFHAPGFVELFNSVQGIAT

AGMMSVNYPSVYSSFSQNVGWSVGIISWDGLQHQIDEFRNSTGGNLKKSSLTQLKQATLI

RQNQTNGAMYGGSLMNNETITERMVQLFTREDTNGTDTNGTATFSMGMGGGGHTPTSNGE

PQKTGVSLVQGLKAYVEELSIPSTNTFMTVLVWFCIIMAAAITLIMTVKLALEVYCRWYK

PLGKDLENFRRRYWIHLGSTIVRVVLVLYGLGVLYCCFQFKQNDSWAATVLAAITLALMT

LTLLALTARIFFLALYASREKGGLEYLFSHKPWIRKYGLFYDQFQVKYWWCFVVFIAASF

GRNAFLALGYGHGMVQVVGQLVIDCIVFIFVCWVKPFNTKMGNWINIAIAVIRIISMVLI

LTFTVELNLTKISTTGTGLALIIVQAILFCSLTILIFANFIYGFVITRKLKKKKKLEMAA

ALERERGANEIDLGLDHSLHGSGTDSLSGTRQVDEKHPYDAYDTSVSNEDERLYHRDIES

SYEDGTNFEIVQPGSRDSQQRESYNSNGNVERHSTTLSRLSRLSQLSSAHGETEFYKDRS

TTPSNALDHHQQMTGVAGTGAGVFGGDAIADSYNVHRGPLFADPRYSNSQTSFSSSGSQH

VSRFTEALNDSDDSDEGRGLGRYVAPLTTKNQFVSRGASTGAPLTPDTPENAQFGGYAGA

TPRAPSSGYSGVSDRPKSRLSTLSGFSGVSRVDGTPVGPPPGHSRGKSSMSDNFFDTHDD

FQDYSLPSNKRYSDLRFSTTTSDYRNSGRSSDVPSDLGSQAGSHHSRLLDDPAVFGRPLS

GEDSELDPAADITITPGSAGRFTTAPPAVPVPHAPHHGHHDTSLVDDFSFLEDRQDDEGF

SWEQDINRGSVTHERQFM

>tr|Q6CFA2|Q6CFA2_YARLI YALI0B08899p OS=Yarrowia lipolytica (strain CLIB 122 / E 150) GN=YALI0B08899g PE=4 SV=1

MVASILATVAPYHLWTYSLLFGATAYQSFYNGIMAYRALPHEHFSNLQAKLFPGYFAFQA

LASGILLLTPPFVAGPAFYWPLGICLVGGLLNTLILGPANRRVMAQRKAQLDKEGLSHHK

DPKASAELKAINKEFAKVHGASVLVNLIAFGAMTFYGATLTGGLLKTIPK

>tr|Q6CFB2|Q6CFB2_YARLI YALI0B08712p OS=Yarrowia lipolytica (strain CLIB 122 / E 150) GN=YALI0B08712g PE=4 SV=1

MTETLRTQCTWLGLYFFFNLALTLFNKAVLGSFPFPYTLTGIHTLCGTLGCALLHWRGVF

KLTRLSDQENTTLILFSILYTINIAISNVSLQMVTVPFHQVVRATTPFFAMLINVVFLRH

SYTVLTYLSLVLVCAGVGFATAGDYYFTAMGFILTILGAVLAAVKTVVTNRIQTGRFRLS

PLELLYRMSPLAFVQTLVYAYLAGELDVLGLRLSSPEDVVGATASATSGPLSFLGGIDYT

EIEFEYSQKLMLHLLLNGIIAFGLNIVSFTTNKKTGALTMTVAANVKQILTIVLAIFFFN

LTVTPLNMMGILVTLLGGAWYAKLELDRKSDNSGAESALPVKEANRSQA

>tr|Q6CFF7|Q6CFF7_YARLI YALI0B07425p OS=Yarrowia lipolytica (strain CLIB 122 / E 150) GN=YALI0B07425g PE=4 SV=1

MDEPSSSPLDIVSVRFRRALQVYQHYVDKCVPHKMNRWVAFGVLLTLFMVRIIMAQGWYV

VCYTLGIYLLNLFLAFLQPKFDPSLKSDLEMEDAEEGQLPTEEPEASSSSEEFKPFIRRL

PEFKFWHSATRATVISLVCSFIPAFDIPVFWPILLIYFFILFSLTMKKQIQHMIKYRYLP

FDIGKKTYK

>tr|Q6CFQ8|Q6CFQ8_YARLI YALI0B04642p OS=Yarrowia lipolytica (strain CLIB 122 / E 150) GN=YALI0B04642g PE=4 SV=1

MTILSATVEKAKKAKEIWLQDDPRDQDDDDSQVQAEVVEVDDSLIEWIAMKLGIPPSDFD

GSYPREVHFMAEKLAEMTLEKALNIVKVNFEYHDHDNNFRHEYRVEINGLKEAFGSGEDP

EKEDPENILLVRYWATIFHWWSPYPEVRSVTDPYDETETTVETWRVWVLGTIWVAIAAFV

NQFFSVRLPAISLGSGVCQLLLYPCGRFLQYTLPDWGFTFRGKRYTLNPGVWSQKEQLLA

TIMIGCAAGTPYVTSNIITQALPSFYNQDWARGFGYQFVLTLVTQMMGFGLAGLLKRVAV

YPVKAMWPSLLPTLAVNKALLAPNRKESINGWKISRYTFFMIIFAFSFLYFWVPNYLMNF

LQTFNWMTWIAPGNKDLAVVTGSVAGLGFNPIPTFDWNQAVAGIAPITLPLYTSVTGFVG

TFFGGLVILAVYYTNNSWTAHIPINSNRLFDNKGKSFNVTRILTDYKFDKEKFLNYSTPY

YSAGNLVLYSAFFSIYTFSFVYTILMDWRAMRDAIVETAKALRYIHRSNYHGRVDPFSRY

MRRHKEVPDWWFYLTLLIMFVLAIVLVEVWPVDTPVWSIVFVMGLVAVFIIPFTVFVSYT

ATSLSLNVLSELIIGYALPGRFMALNLIKALSVQIAEQAENYTSDQKLTHYAHLPPRSIF

WLQIWATLVNIFVCLGVIQFQLGLDKICDADNRLKFTCPSETTFFTASIAWGVIGPKKMF

DKYPVMKWMFLFGACAGLFFWFVQVMVPYYMAKKWPKYSEKIHYYRRKCLYFNPLIFVVG

MLAWAPQNLTYKVGGLYLAILFNGYIKSRYLSWWRKYAYVLEAAMDTGIALSGIIIFFSV

QYHPKDLEWWGNDVIYDGMDGGGIPLPPHVPIPEDPGYFGPPPSQW

>tr|Q6CFR8|Q6CFR8_YARLI YALI0B04422p OS=Yarrowia lipolytica (strain CLIB 122 / E 150) GN=YALI0B04422g PE=4 SV=1

MISLHLHLLRWPAYVTLLAALLLNLYIYNYPYLQNRTECIWRDDRGWNLDKSHEEVHLLA

FGDPQIRGASNTSNWRTRLDIFGNDHFLGHIYRVMAKRTKPNQVSVLGDLLSSQWITDDE

FERRADRYFGRIFDESLVKNNDGYVMWYNIAGNHDIGYGGEMTRERIDRFENRFGRVNYY

VPRKGYRVVVLNNLALDGPVYEQQFQNDCWMFVDMVRQAREQEPDVSTILMTHVPLYKPA

GICADGPMFKYYDNEYKWLRSQNHLSEDATNRLLDGIFGREDPRGVILAGHDHEGCVSSY

SFDPGHGNWTVEGGRGNAVSEYTVRSMMGEFGGNTGLLTGSWNDTEGKYDFDYSLCPFVV

QHLWWATKVVTIIATIVALPVAVSIHYS

>tr|Q6CFS8|Q6CFS8_YARLI YALI0B04202p OS=Yarrowia lipolytica (strain CLIB 122 / E 150) GN=YALI0B04202g PE=3 SV=1

MIEAPLSQGAGYGVVLGVGLGFSMIMIFITWAMRRYQREVMSSEEFSTASRSVKTGLIAA

SVVSSWTWAATLLQSTSQAYKNGVSGPLWYSTGACVQVLLFATLAIELKRRAPGAHTYLE

VIKMRFGPAGHIVFMCFGLATNILVTLMLLTGGSAVVKDLTGMNVVAACLLLPLGVILYT

LFGGIKATFITDYLHTIILLVIILVFAFTTYANHDVLGSPGKVYDLLVARALEFPVKGNE

HGSYLTMKSHDGAIFFVINIVGNFGTVFLDNGYWNKAIAASPAAALPGYVLGGLAWFAIP

WLCATTMGLACLALEGTPSFPGYPARMSAEDVSAGLALPNAAVALLGKAGAGCALTMVFM

AVTSAFSSELIAVSSIFTYDIYKGYINPKATGKTLIMTSHAAVVTFGFAIAGFAIGLYYI

GVSMGFLYLLMGVIISSAVIPAVLTLMWGGLNFWAGVLSPPLGFCFAIITWLVTTKCEKG

VINVDTAGSDLPMLAGNVVALLSPLMTIPVLTLIFGLDKFDFEKFKQITRADDSEFWKVD

SIVEELGGSDEIVSIREKRANSPNKTGGSTTPEDPMEAEYGLHEPLPAAVEPPVHLEELD

PAEKALLDKYAKIARIITAIMALCLLVLWPMPMYGVGYVFSKKFFTGWVVVGIIWIFFSA

FLVVLYPLWESRQGIFETFRGLYWDCTGQTWKLRRWQGQTEDKQMAAHGVTRTFSHLERL

DGQEVESDEGVQVTKIED

>tr|Q6C450|Q6C450_YARLI YALI0E29667p OS=Yarrowia lipolytica (strain CLIB 122 / E 150) GN=YALI0E29667g PE=4 SV=2

MIRTIAQQSFRVARPSLRQGPLAAYRCLQTQTTTPAEALDILNKQRALRPTSPHLDIYQP

QLTWYLSGLHRVTGVALGGALYALLCAYAAGPALGIHIDSTTLAHTFAAVPLVAKLPLKA

LVAFPFTFHAFNGVRHLVWDFTKELTVKGVYRTGYTVLGLSVLSAAVLAFI

>tr|Q6C4A6|Q6C4A6_YARLI YALI0E28424p OS=Yarrowia lipolytica (strain CLIB 122 / E 150) GN=YALI0E28424g PE=4 SV=2

MSSSTPLVKTSVNYSYGDYPLIDADPHFKRVVGYMRPSDYGVIGLATAALPAGICFAEWL

DPVKGKFARPSVKFLRVATMLGFAVGFGAAYARSSLRFFGVTENAREYKKDEAQMAARKA

AGLEPYGTSSLTPELQEIAAKNSAHSIAGLFIFPWFNFVNHPYHGREQK

>tr|Q6C4J5|Q6C4J5_YARLI YALI0E26257p OS=Yarrowia lipolytica (strain CLIB 122 / E 150) GN=YALI0E26257g PE=4 SV=1

MSQLWAKWKSRPPKINLLGVEFAWDDLTLKRLVKTFVNTTFCLIFCLIPACVHRIGKVGY

MLTLFSVIVPPAKRLGKEVESIVITLLGLGLGVGYANLTRYIAKLPLRHMELKSEKGLSG

NPDYRSALGILALGEFLMLMFHGYWRSKIPRIFAGVLTFLIVTHFSYLSDLEVSVQNMCI

NFGYPIMLACGTSIFFNVTIFPESGSTDLGKVTVALIKELKTSISMTVTYFVNSAEIMRV

HLEEEQRREQEASNGGAESPKSIHGNTDDSNETPETYVPESIHDNTKIDNNQSQASVSPE

LDITELGTLLKKKTALTAKYTSCKAALTECTFEITYAYVSPTVLKPTVKLLNGIVKSAGA

MLDACELEFALLGKVQAAQLQKTNSSSNQAAEEEQDIIKRLKPEREIVHGNKELLLAMVS

RVAEPIHVLDKAMTDAFDHAVHAVCYAFSVGEQLPNIRDFPDKREEFKDAIETFDVVSRS

ALASLDFGTVDHDHIDDYLLPRDELFLLASFILNLKSCALSIMSLLDEAQALHATRRKRE

DVRGWPILGKKRLWSAMFENRKSFSKYFFSRSDDARNDGDNIEGHDVLTFTQNLETREAA

ITRQMSNTDQPKDLKEAQPASEKKTKESHYVRFRNKWKMFDLWIGNHIANIIEVTPAEEK

HWKFALKVTIPMFLVSWPMFVPSMRLWYINMRGSWVGFVVALVTETTLGGSVFIFILRTI

GLTIGSAWAVLAHTAGRGHIHSVMGVVMAVIFIPALWFMLATKYFKGGMITVVSANVVLL

ATLYPTTTGGIVTNFAKRTLAMMIGGGAALLGQLFLFPVKAREELVVVTVQAIRECSRME

RIIAYGFDDPATAQKEFKKCAKVARGSLTAAATFRTFTKQEPRLKGSFEEISKIFGEIIF

VTRQIIDRLENIVFLRQQYGSVVLDEYGPEVLAYRRQTAAAITTTLRAVEAALLNKTPLP

QYMPSARLAHRRFVNRVREVMITRLSQNKHHHSVPNIGTPAVGTPRDTPRETPRETPRET

PLATPLGNPSHPELSSRASLPNVHTGSPRRCSEGDTTREKACVPDLVVNDTDTPHALHHS

SDDTSDSDDSSQEIEIVPRARTQQQQQQQPHDYLQHRAVVLQQKFMSWSATSSAVEEVIE

YVEELADLTALLVGVNKFKYGFLSRPIYAEWAASAARAYDEVLAPHDPLKADNDDEEPDE

TPHELRTAADHTPDILDRDHIPRGLRKRVFSVVEHNGQIYGGGEHQYNMVPGMPAVDRTT

SRQSQKSNKSHLSQPVARKTSSNASMPPLTRRQSIRSVVGAVNDSPEPESKYLTLRRRMG

RKINKKH

>tr|Q6C4K4|Q6C4K4_YARLI YALI0E26026p OS=Yarrowia lipolytica (strain CLIB 122 / E 150) GN=YALI0E26026g PE=4 SV=1

MTLYYTLVFAILVTEMATFLLLVAPLPEKIRRQFFLSLAKLEVLDKLRLGLKFTFVFILI

LFIDSVNRVYRVSVDRSTGEYKGNLVATERSELQARKFYSQRNMYLCGFTLFLSLILNRT

YSLVIELINARDLINELKGSSNAKVKAELSASDDANKEITLLKAELQQKDKDIAALKSQS

ANLSTEYNRISDELNASTGNTKLDKKTA

>tr|Q6C4R6|Q6C4R6_YARLI YALI0E24299p OS=Yarrowia lipolytica (strain CLIB 122 / E 150) GN=YALI0E24299g PE=4 SV=1

MSKYNPIDDDADTTAAWGDDLGDDILADEEPPRYDPQPETPAAAPASNINNVQGSMAGGN

TNTSSSTAANQRVPRANPGKFWNLSFYEQYFDVDAKEVGDKIVQAVNPLSGGGQSSTYFL

DQPADLYGPFWIASTLIFALFFANTLVGLIKNSSGEKFSYEFGLITANAALTYSYTFLVP

GALFLLLNYWDIGATSPHLSKLVNLIAVYGYSQCVWVLICAGNIILPVWLPVGFWINLVG

WVLTTVGFLSSGWFLLKALYFPVKQNSPHLHLRVLVLVLLLHVGFSLAVKFAFFKGVAPV

KD

>tr|Q6C501|Q6C501_YARLI YALI0E22264p OS=Yarrowia lipolytica (strain CLIB 122 / E 150) GN=YALI0E22264g PE=4 SV=1

MSFIAKHYGKKYVTSHLNGAIKTNLEPAHPYHYYEEIPPPPGANEKVKPKKKKIKRTLPQ

GLQKKDEKVLKSFLSWSYQLDWIFDMCGFGVGWATLIGLVPVVGDLFVFYLGYRLVQKAK

KQLTEGLPPALEAQMILNLAIGCGIGFIPLVGDVANAVFKCTTRNANLMETHLRQQVGAP

ATPMQDPPSNSTVVGSHGK

>tr|Q6C539|Q6C539_YARLI YALI0E21241p OS=Yarrowia lipolytica (strain CLIB 122 / E 150) GN=YALI0E21241g PE=4 SV=1

MSIHSSSSHDKDIVQIVEDSIGLSKTDTRSSHVSRASKASRAASDYQIDHIYGDMDRREI

TLARQITRETILSAYQEKVRSRASSIINGKIDPSTAPPPPLKNGGDGSQFLDVDPELVTW

DGDEDPENPQNWHTYKKVATTVIVSLYTFVSPLTSSIISPAVPAIAAEYNETRPVVQSLM

VSIMILAWAICPLFVAPLSEMYGRKIVMDVSILVLLAFTLGCGGAQNTAQMAVCRFFAGV

GGAAPLSVGAGVLADLYSPQKRGTALAWYAIGPTVGPVVAPIAAGWIVQETNTWRWVMWV

DGIFIGCVAACGFLFYSETYPPVLLQRKAKKLRKESGNDALHTIYDIASEPLSSKLYTSM

TRPLRILVTHPIVMGLGLFMAFTYGFMYIMIVTFPALWTERYGFSLGIMGLTFLGMGIGF

LAGTWFWCVYTQKVYIKLRDQNGGVPKPEFRLPCIFAAPFLECIGLIWYGWSAESHVHWI

MPIIGTGIFSFAVMDVFQTIQTYLIDMNPRFSASYVASASLFRSLFGFAMPLFGRQMYDA

MGYGWANTMCGILAVVLGLPFPIIVWFYGERIRNRFDKKLEASQAKKDEKNMEKMRKREL

ERELKEQEKLDKEKNEHVHFVAVETSPSIKNEDSL

>tr|Q6C559|Q6C559_YARLI YALI0E20801p OS=Yarrowia lipolytica (strain CLIB 122 / E 150) GN=YALI0E20801g PE=4 SV=1

MTASNTPQLLKHSGIVLSGLLAGGQFFTTLYVKPMLDRMSTSDAYNLFELIYHVGQVVFP

ALTLMSSASFATAAYLEKDINPTNSRQLAYSAALVFALLPYTRFAMWPTLTKLFAASADK

VKGPNVKALLKMWIVHHYIRNIFTLLGFIGGVAATV

>tr|Q6C563|Q6C563_YARLI YALI0E20713p OS=Yarrowia lipolytica (strain CLIB 122 / E 150) GN=YALI0E20713g PE=4 SV=1

MSYTPSENSVTKPEHDDLHNDVKGYTHDQEHATTDFSDGPAAEIADDGVKRGLKTRHLSM

MALAGIIGPGLLIGSGGALTSGGPASLIIGFGCIGIIAFSIMQSLGEMTTLYPTGGAFTK

LGDRFVDEAAGAAVGWNYFIIWACVLSNEYNALTSILYFWSEKVPIWGYFLLFWFFFLGF

QLLGVEIFGEAEFWLGLIKIVGLVAFFIFAIVYAAGGVGRLDNGDGTTRALGFHYWHDPG

AFPNGFRGVAQVFVFCSTFYAGVESVAVAATETRNPSVAVPRAIRQVFWRIIFVYLGSAF

FFGLVCSSKATQLVSADAKALQSPMTVAIQTAGWHGGVHLINAFILVTCLSAVNSSIYIG

SRTVLFMAKDKKAPKFLAYTNKRGVPVFAIIFTNAFAALSLMNISTGAGKAYGYIVNLSG

VSTFLVWGSISFIHLRFRAGWAAQGYSPEDLPYRAMFFPYNAWFGLVANVFLALVQGWTT

LSPFDAGNFVDAYILLPLFFVIFFLYKIIYKTRWVRANEMDVISGRRKDLDTKVDEEQMI

DGVMTPKKKTVWKKVCENF

>tr|Q6C5C2|Q6C5C2_YARLI YALI0E19294p OS=Yarrowia lipolytica (strain CLIB 122 / E 150) GN=YALI0E19294g PE=4 SV=1

MTDIKPVDLDTKEYDIDTADVREITLHTEEWIAQKLLIPPSDVDGTYPRSVTVISQAFEN

MSEAEAIAIVKKAWDQHSSDANLTAEHKDVLQHLIEMIGEGKEGEDESVPPEDLEELKYQ

ACLFHHWSPYQQVRSVTTPFDDQDEECETFRCYVIGIIWVGIAAFVNQFFSPRQPPIQLT

VAVIQLLLYPSGRLWQFIFPDWGFKVRGVRYSLNPGPWTQKEQLLATLMASVANVPPYID

YNIFVQYLPKFYNQPFALNFGYMVMMMLTTQFMGFGMVGFLRKLAVYPAKAMWPTLLPTL

AVNKALLAPNRKEKINGWTMTRYTFFLLVSAIAFIQFWVPNYLFQAISTFNWMTWIAPNN

VVLAAITGGASGLGFNPISSFDWNQFTAAVFPVMFMPISSLVLGVSGMFVAGFIILAVWY

TNTQYAGYLPINSADVFDNTGLPFNVSRILTNNEFDEAKYRAYSPPYYAAANLVVYGAFF

AVYPMSFFYTVIMEWEIMKVSLRDLYDGFKHWKRSNYYGFDDYFSRTMSKYAEVPQWWYM

VILLITFGMSIALIEHWNTKATVWMLVVVMLINFVCLFPFTIVMSYTGIQFTLNVLVELI

VGYALPGKGIAMMILKAFSVQIQIQCQNFLTDQKVGHYGKLPPRSMFRVQMLATIVAGLV

TLGVMQFQLNDVEHMCDVKYQTSHEKFTCPSETVFFSASVIWAVVGPKKIFDSQYPILRW

CFLIGFGIALLFVFLQKFVPRMLKKRYPDRASEITKWEARFKEVNPSIICYGFLNYAPYN

LTYVWSGVYLAFFFNVYIRKRFPGWWEKYTYVFCSAMNTGIALSAIIIFFALQYTNVNLT

WWGNTVSFAGVDGAGGAGIVPIPDKGFFGPDSAHYP

>tr|Q6C5H5|Q6C5H5_YARLI YALI0E18007p OS=Yarrowia lipolytica (strain CLIB 122 / E 150) GN=YALI0E18007g PE=4 SV=1

MADTNSRIRWWIIPMVVGGVVIGALYLLVFCKGQKKKRFTELQRQQLEQGYPNDPRPPNF

TYSYPETISGYNQTTVIYNHRVYSSNGEGGVNVTNDPVTHIPMTSQTIRDESNDVPPAYY

VAVSPPPKAYDGATSYGYDPVGGTGNGSHRNVYNPVNAGSGSQSAHISSNSRPQPQGFWG

RFKKRDKTESNRDSTSGAAGGSGDAYGGCHGGGGYGGGHGGHGDGGGGHGGGGGDGGGGG

GGGGGDGGGGGGGD

>tr|Q6C649|Q6C649_YARLI YALI0E12441p OS=Yarrowia lipolytica (strain CLIB 122 / E 150) GN=YALI0E12441g PE=4 SV=2

MSFPDKIVADFNVDYDQKSLWIVLGSIAFNPIFWNIVARAEYNTHFLTKLAGGAKTGCYL

LAITIFSLGIFRDYLYHQALEQQPTSILLSVTYVDIVKPLAIVLFAVGNVLVLTSMYALG

VTGTYLGDYFGILMNERVTGFPFNVSNNPMYHGSTLCFLASALWYAKPAGLLITAFVSLM

YSIALKFEEPFTGEIYAKRDRELAAKQK

>tr|Q6C655|Q6C655_YARLI YALI0E12309p OS=Yarrowia lipolytica (strain CLIB 122 / E 150) GN=YALI0E12309g PE=4 SV=1

MGTLIVVATAVVFVAEMVLQARQLVYLGASHTVPPELTHMLDCVGLRKQNQLAWAELRSQ

FYLALLPFVEKTAIFGLGLLPWIIWASEFIQGRLRLRLSQIHAPQTLITMALSDCVNAFL

ASAIFYILQRISLLLHAYERDANVLQLHITKMEYLSEYTSETLQKGVVQCLIVGFAELAV

SALPWKKPHMSLVFLVPLIYTATHMMVLKIHRTSPPPNAYPLMECQLRSKLENVARDLNF

PNPTQIYVYHKGKPRIHVAGWTKKTTHIYIHNHLLTMHTHPEILLFVAHVLAGRLFPINT

DTFPLGWMALSEYTRGVLTLLLCWNPSFHTYLGLKRWQPILLAVYIIPCIHYPIYKACQY

ANNYWKRSQTFQKDAGLVCLGNVHLSYYGYTLSSAYKRVSPLDGDPLWLTDLSGRSKLKE

RLEAIGWVPRGLAEKDTEKDTEKDKRRASW

>tr|Q6C6Y5|Q6C6Y5_YARLI YALI0E05269p OS=Yarrowia lipolytica (strain CLIB 122 / E 150) GN=YALI0E05269g PE=4 SV=1

MSAYQREEQNNTRLEELASKISSLRSVTNDIHAQASDYSLIDSTTDTVGNMMTSVRGSAS

KLGRSLKAGHPIWRTVGIALAIILLLWFLFKFF

>tr|Q6C7C7|Q6C7C7_YARLI YALI0E01892p OS=Yarrowia lipolytica (strain CLIB 122 / E 150) GN=YALI0E01892g PE=4 SV=1

MTPLIVRRLFLLLVTASLGLLVFLIYFSLQDDWTRTHADFLKEESIYKQPNDAFLQYTEP

KEDLTPKNNPLLEEVDEPVSAKPLVRATDTRRTKAALISLVRNKELDGIVDAMVQVEDTF

NHKFGYPWIFFNDEEFTDEFKEKVREQTDSEVKFELIEKKDWDAPRWIDKDKAKKAGKKL

EDAGVRYGAMDSYHKMCRWNSGMFYKHKAMDDYDWYWRVEPDTQYYCDIDYDVFAYMEDN

DKVYGFTIALFDNPKTVATLWPETKSFLMKNKEYLHKDNAMQFLLNPSRPDWNAQAGGYS

TCHFWSNFEIASLKFFRGDAYAQWFEHLDKQGGFFYERWGDAPVHSVGAGLFANQSQIHW

FKDIGYYHFPYFNCPKSKKCHGCKPGRFTTDDLKDSLMPENCLPQYLKYMGDR

>tr|Q6CCE1|Q6CCE1_YARLI YALI0C10142p OS=Yarrowia lipolytica (strain CLIB 122 / E 150) GN=YALI0C10142g PE=4 SV=1

MNSTTSAPIDSDQAPVPSDAPCERTPLLSGSSRPHRFSWWTHDSHPWFCEMPKYLLMVVA

VVVFLGTVRGSILGVHDLMDNTTMVVKDTKIVDLIGKDGIAVAVRGELQMDFEVVEHDLA

RFLIKQGAKLLDHVVVQPTTMTITRVTFPNDTLPIYEQVTLVDVPQLNLNTSPSATNDID

TVVTLHDYDAYTIARLGELMLAGTPFTLSGTAKISLGRGWLRLGSYWITSQQTINQDIDQ

EDFVDYKLKGMRVFGEPGRLSVDAQVALKTSLVDQLDFPELKWDLFHKRCEADLSSGNSR

KILVGQPQGDYVIVGEATSPAFHLHKGENEIQVRVDMKEGGEVLKECQEDEEKNKQAHRV

GVLGRLSRLLSGSVSDPKFAQSSLGELDSDDGLKQRLSTLDKIMTAFINGNSSLYSTHLS

PQQPRLPGPLTDTLSHFWALLDSPGLDRQESLLRNISFTHVSIASLSSGAVFIDADVETT

ILLPSNVQIQTEMLPFEVRGVNDLYYEGQLFARAHIQQWHDCESFQGLEGSQTVYNVKVQ

AKNVPVEVIDSSIFRKVASKLLWAGSVPILFNATVDALVETTVGNLTLEGMNILKETELN

K

>tr|Q6CCE4|Q6CCE4_YARLI YALI0C10076p OS=Yarrowia lipolytica (strain CLIB 122 / E 150) GN=YALI0C10076g PE=4 SV=1

MLPRFDFCPKIPHIVVLLLALSAMASSAKIASPFVDFAVQPLQGQMWDLYTAHFLLDTTG

GVSVGDFFVFSLESDNELFTQSYDTTIWSGDVQVGRLSSDEAWNFRFDFAEYVERHPDAK

ISFSIEFYLAELAPKSVNKCQLEGKNIFNKYTSPFVDKISQSQLNPNSVKLKLKDETKEF

QATLLVEKLQDTDMKMVRTARGVLYTQTIMFHKNQRHFELFLRTSPGQSWVDFSSPFDSL

YFVDNDGNVVDSAITARPRVSASRIHLKIVIPYMDGVAGIFFAATTRLGLEPGVVHICAT

VAAQGEAATRCQSLGSGPDGVPGAQGWVVGGESLDDAENPFQRRNRQFKRDIEIGDSSDS

GRNPSNGNNNGQGNAPGGYYGHNNGLGNGHHNGNNDGNNNGNNDNEAATGWRELASVTRP

GPWVIGPGDSVATTASYSPGSPTLGGSGSGWGLNPVPTQPAPKPIPPLPRTPLLKTTVPS

LASAPVWVTSPVLASSPVTDIPQANLAGSLRVAVITLSLLVFLGLILVF

>tr|Q6CCW6|Q6CCW6_YARLI YALI0C05951p OS=Yarrowia lipolytica (strain CLIB 122 / E 150) GN=YALI0C05951g PE=3 SV=1

MVKNVDQVDLSQVDTIASGRDVNYKVKYTSGVKMSQGAYDDKGRHISEQPFTWANWHQHI

NWLNFILVIALPLSSFAAAPFVSFNWKTAAFAVGYYMCTGLGITAGYHRMWAHRAYKAAL

PVRIILALFGGGAVEGSIRWWASSHRVHHRWTDSNKDPYDARKGFWFSHFGWMLLVPNPK

NKGRTDISDLNNDWVVRLQHKYYVYVLVFMAIVLPTLVCGFGWGDWKGGLVYAGIMRYTF

VQQVTFCVNSLAHWIGEQPFDDRRTPRDHALTALVTFGEGYHNFHHEFPSDYRNALIWYQ

YDPTKWLIWTLKQVGLAWDLQTFSQNAIEQGLVQQRQKKLDKWRNNLNWGIPIEQLPVIE

FEEFQEQAKTRDLVLISGIVHDVSAFVEHHPGGKALIMSAVGKDGTAVFNGGVYRHSNAG

HNLLATMRVSVIRGGMEVEVWKTAQNEKKDQNIVSDESGNRIHRAGLQATRVENPGMSGM

AA

>tr|Q6CCW8|Q6CCW8_YARLI YALI0C05907p OS=Yarrowia lipolytica (strain CLIB 122 / E 150) GN=YALI0C05907g PE=4 SV=1

MSYSQVPVNPPLETVELGEINRTQQDYHPGEHLRSQRHIWTRINALEHQIEFLGGQLREA

KEAMNPKGQKTTYSESTAYGILSVIALMCSIIPGFFVAMDLSDGPATLMCMGQICLICFI

VLFTTLPFAHKDRHIFRIFTAIVYLVLLGWETYVDLWAGLIHAATMVLTVVFYMLDAREA

RIREMTESEVDTVV

>tr|Q6CD90|Q6CD90_YARLI YALI0C02783p OS=Yarrowia lipolytica (strain CLIB 122 / E 150) GN=YALI0C02783g PE=4 SV=1

MKKDVEDSALELPTEEFISTDKYGISTDKNTPDVSSEEHITESKDVQSQEVIEKGVMETS

IVERANRTAEELFPNINKKKLLLKLDLNIIPILAILYLLSFLDRGNVGNANIEGLSVDLN

LKGNEYNMCLTVFFFTYSAFEVPSNMILKKLRPSIWLPAIMVCWGVVMTLMGLVKNYHGL

LVCRIFLGFTEAGLYPGVTYYLTLWYCRSEIQFRQAMFFSAASVAGAFSGLLAFAIGKMR

GVAGLYGWQWIFILEGIATVVVALVAFFFVHDFPETARFLTEEEREYVMWRLKYDGNEAN

SGELVEQNDARDWKYVRAAFTDIQVYVHILLYFGIVVPLYGISLFLPSIINGLGFTSAIA

QLLTIPIYAVAAIASVTQAWFSDRYGKRSPFVVVNLLLMLLGYLLAYNLSHSSPPGVYVG

CYLIALGLYPAFPAIVSWMANNVAGTYKRAVAMALQIGIGNLAGAIASNIYRSKDKPEYR

LGHGMEIMFVCIGLAALGVLNYGYHSANKKKAEKLAAGVYDNISGKELSEMGDRSPYFKY

RH

>tr|Q6CDJ2|Q6CDJ2_YARLI YALI0C00110p OS=Yarrowia lipolytica (strain CLIB 122 / E 150) GN=YALI0C00110g PE=4 SV=1

MALFINLLAVFVSLIIGGTIYLDRSFSELTNRVSWNDRHVYNKAAEYNAASCKVSKAIPE

ACEDSIVDDSTGLVYFSCGETENRTKWFPGCNWWDKEGMTTNSFYVYDPETRDFEKMNLD

YHGEFVSHGLSLLKDPNNPDTRYIFSVNHKSTGSIISIFSFQAGTNDVSHVGDIKDSRIY

TPNSVTAYVEDDGSIAIMYTNDHLFRKGFMRHVEDSYGPFMWGHVGNCNFKIEDGDIKGL

SCSRVAVENSYPNGITHIPDTKEFLVADTRLGTLSSFKWDVDTKSLNLQHSTPFGAALDN

VHVIAGTKDVIVAGFPDEKQVFYKFRNMHDKSIKVESLVFRANSRDNYTVPHVLQKTDGL

HGLQVVTNYNYLPKHDRLLGSSVCSEGILICDNIYGIIM

>tr|Q6CDK2|Q6CDK2_YARLI YALI0B23298p OS=Yarrowia lipolytica (strain CLIB 122 / E 150) GN=YALI0B23298g PE=4 SV=1

MTAKSKTTVIDGKTQRLNPKTTDLDFFGIPGATFITLTLPLFILFLQIGCTPESCTVAGV

LNIDWSAEMTKLRAMPLFDINVFGVYLTWFFSLAACDLILPGPVAEGVVLRDGKTRLKYN

LNGKTVMTTLAVILAARWVQTGGAMPELVFCYDHYVQIATSAIVWSFIVATYVYVASFTH

DCILSVGGNSGNPIFDWFIGRELNPRIGSFDIKYFCEMRPGLLLWAVLNLAMAHHEYITK

GFVSLPMALVVLMEIYYVVEGNWEEYGAIHMMDITTDGFGFMLAFGDLAWVPFVYTVQAR

YLSTRTIEYPLWYAGIIVTLFATGYYIFRASNNEKNAFKHGDAPHLRYIENDKGNKLIVS

GWWGAARHVNYLGDWLISWAWCLATGYESIFPYFFVAYFGVLLVHREMRDDHKCALKYKE

LWVQYKKAVPSAIIPGIY

>tr|Q6C7J0|Q6C7J0_YARLI YALI0E00462p OS=Yarrowia lipolytica (strain CLIB 122 / E 150) GN=YALI0E00462g PE=4 SV=1

MTEVYEYSPSGMRARRPSVDGASQHSDQDVCYPHDDNKEGFEIDFTALDDFNGEDDVPCG

VANADMGSRVVRVAPQDHFAFFSTECEETIRANEFSEIFKDGCKAKELFDAHKGTWWLDC

YDPTDAEMKLLAKAFGIHPLTVEDIQHREAREKVEMFRNYYFVAFHAYEQDVESDDFMEM

VPFYLCVFPEGVISFHYSPVPHCATVRKRIRQLKDHVTVTPDWICYAVIDDITDSFAPVI

REIEQEGEIVEQTVFDARDDGFNEMLRRIGKARAKTLSMMRLLSGKADVVGMFTKRVSEG

LSDHVPKGHISLYLGDIQDHIVTMYQSLIAHEKILSRSHLNYLSQLQVQSIDATHRVTDT

LGKITVIGTILIPMNFVTGLFAMNVRIPGESTDENPGNFGWFFGILGVLLCIIVTATLVA

KWYLAHVEKKIRGNFRNSASDNSIRSMRSKRSKHTINTIV

>tr|Q6C7K6|Q6C7K6_YARLI YALI0D27302p OS=Yarrowia lipolytica (strain CLIB 122 / E 150) GN=YALI0D27302g PE=4 SV=1

MSDKEKKKSSATHAAFPPSTASQPQSMSPLLSSTPPTVTKALAQAYPYILASDKVLGLLT

WTEDDQWQSFLLVAVYVTVVMYYEYLVIYCGHILAVGFIWAFVYIRQSVERRQTSEPSLD

AIVHTLTNVTTKANLLLLPITSLSLTPRDVTRLAFTTLFLSPLYMFGAYFFLGPRKFLLT

TGVFFLTYHSMAARVTRAVIWKSKAIRLVTFYLTGLDFSNTKRNLGAFGFTQSPLSVQSK

DGKPVRFTYVLYENQRRWLGIGWTANLLAYERTPWTDEFLNEVTPPSEFKLPDTEGTGMK

WQWVDPTWRLDCTNDGALVIIGNKALSTPDPSPSEGWIYYDNTWKRPTADDSFSKYTRRR

RWVRTAELITVTKPTDVVVTVEEDGVTDAAGDVEIITTETEEKVRRRKGIRFEEDS

>tr|Q6C7N1|Q6C7N1_YARLI YALI0D26752p OS=Yarrowia lipolytica (strain CLIB 122 / E 150) GN=YALI0D26752g PE=4 SV=1

MANLNGAGRTTSSPGFSRRFDRRDLLNTSRDGNTAPPINSVRDSTAYPDTRRSSGVAKPI

KTFHATQKPWRQEKITVPPKSEPLIPETYMSAANQRWAAVSLFLLFLASKITYLLTPVSP

GEQFGLLFKFFALDLVLLWTLPLFRIPWLTFGPVVTTLLSFAFLGFDMGIVYYREGLVFA

LGLGSAVWRTVNDKELSFSGLKIKVASLQDASSHLIGEHIVQILPKSTALINPMDQPFCL

TGDSAQTITIPVRLNATGLSSLELKYIDFDNLEEKSFNFTKNQLVKFRGRSDYVRSLDYK

LKDADKKKLFYVELPVDKTGLYRLGKVVDDSNNAVRTYMKDVIVGACPGAKLRQSNDSLH

LGSNRCIGGTEQPNLVVTGVPPLRIKYSKEVEGQSSVFSVQSVQPRHFVSPIASGDVNRY

VWKSGADLSWAVPHEVPIHLDARLSQTGKWVYTVDEVEDGLGNIINYTAIAKGKDEEWRT

RRQLTHEFFVHPRPQVSFRTCSAEKRARLAGDHVSLDLKVQAEEDLVLTIKHTDEYGKSE

VFNATVPRGQQSIPVSKAGIYELEAVQGKYCAGDIVESSECHVYVPPKPSLSVEFTDIQD

QCAGPVGATAELAFTGQPPFHAVYRTVRDGQVIDTQKITTHRLRHQLKFKPQSAGTYTYE

IVSFSDAIYKQPQDTSFTKKQTIRVLAGALFDKSIARQRFVCSGESVDLPVTLRGVPPFT

LEYEIVHGSSRRVPHTIKDIKDQKHVISTSGLSQGGQYTISLVAVTDSHGCRSLLDEQDI

RVEVRRQRPSAGFLPISGDYDVAVPQGSPVKLPVTLNGDGPWNLEYEYIEGEKRERHRVQ

VGDANGFFTVSRPGHYRLVSVRDAHCPGEITHDAIFSLTWIARPAVKIVKAGNTEAKDGV

LSRAPICEGDQDSVEIALEGSPPFTVLYRVTGPGGKTKDHEIQVATPFANVRVETNAPGE

YEYFFMGVLDSNYYDRKWLADVGAGDDVAFSDSRLRQLVKARPTAKFVDKGRVYRACQGI

ADSADGIPIKLTGEAPFSLTVSIRVDSSGHTEKVTYNNVGADFELKSQLYSRLGLGKHTV

QIYQVVDARGCRQSNFGADQRVTVHVADTPTITAVEERTDFCVGERISYSLTGIPPFDVT

YDFNGNVRESTSGTLFRRLAAEPGNLTIKAISDTASSCRIELEGAKQLPKRIHPIPTARV

EEGTSLIFDENGAEKAEIHFHLQGTPPFQLQYTRSEMISGKEVVVETHTVGSIKTHTYTI

WTGLQGIYRPIVVSDFYCTTAGERASH

>tr|Q6C7N2|Q6C7N2_YARLI YALI0D26730p OS=Yarrowia lipolytica (strain CLIB 122 / E 150) GN=YALI0D26730g PE=4 SV=1

MENHKNDPECNAAKAAAANIFPASPMANSMMFGSSYSQSYNKSFMKRHSGEYNRHSGEYR

HSGESNRFSGDFQRVNEDLESASETTHLNPRPARPSMAVSSSSEPSTNHASNNTIREPSD

TPVYGTTAASISTTSLDPEDNVWEDVPSQKRSQDVRELKILWQYSLPLAVTFLLQYSLTV

ASIFSVGHLGKEELGAVSLACMTSNITGFALFQGLSTCLDTLCCQAYGAGNMRKVGIYFQ

QCVCMSILVFLPIAAIWMCSEPLLALIIPEGQLAALAALYLQIVALGVPGYIVFECGKKY

LQAQGIFMAGTYVLAICAPLNAFLNWLLVWDKHVGIGYAGAPVAVVITEWAMAILIIAYI

AFVDGRQCWFGISPRAAFSNWGPMLRLAIPGVIMVEAEFLAFEILTLASSYFGSAALAAQ

SVLSTVMALAYQIPFSVAIAASTRVAHFIGAAQPQSAKRAARIALYSTVLISTFNCTTLF

LFRRPIAGLFSSDVDVVNLVAFVLPLCAIGQFFDCISSVVAGVLRGQGRQKIGGYVNLFY

YYAVATPLSLFFGFICKWELMGLWAGIIVGIVGIAATEAYFVLTSDWNAIIEKNAAMHRD

>tr|Q6C7R9|Q6C7R9_YARLI YALI0D25872p OS=Yarrowia lipolytica (strain CLIB 122 / E 150) GN=YALI0D25872g PE=4 SV=1

MEAVGENPYESRADISEASTKEVSVYGTNNDVADFFDEVDEIKKIVRRYDQNIDTIESLH

KQALSEISGEQETYTREQIGSLANETSALSQSLKDRIKSLQSRSTRDSTKKTQAENLKRQ

FMNAIQRYQTVEATFRQKYREQAERQFRIVRPEATDAEVKAAIEDVQGEQIFSQALRTSN

RRGEAQTALSEVQTRHREIQKIEQTMAELAQLFHDMELLVAEQEAPVQHVEKHTEQVQVD

VEQGMGHTSKAVVFARAARKKKWWCLLICLIIIILAVCIPVGIWASNNNKKD

>tr|Q6C821|Q6C821_YARLI YALI0D23441p OS=Yarrowia lipolytica (strain CLIB 122 / E 150) GN=YALI0D23441g PE=4 SV=1

MAGNSLKKQAANNKEILITLWTGTVSIDVLFLSSYYFLGNPASIIPWIIFSIPATVVMVH

LEKTCRPKFDERGGLIRPGEDLSQRGVMEYLKDIVYVTWICKFVSAVLASNWGFALYIMI

PLYAVVKAYTLYSENRPKHSGSSGSTANEGLIPKSKRQEKMERRGGQKVTYRS

>tr|Q6C8E5|Q6C8E5_YARLI YALI0D20306p OS=Yarrowia lipolytica (strain CLIB 122 / E 150) GN=YALI0D20306g PE=4 SV=1

MVLVALKKAIFGTEKQPLLRNNASYATMTDEEARLGLTDDDTLSQGSGSSGSGNSWVSPR

VVSDMIIGLSDGLTVPFALTAGLSSLGDTKLVITGGMAELVAGAISMGLGGYLAAKSEND

YYKSECTKERAVLKTESSEGESQIADILAQYNLSPETTASFTKDLQKNPTSMVDFIIRFG

KGLEEPAEGREFTSAMTIGLAYFFGGFIPLIPYFFTAHVDDGLMWSVIVMLITLFIFGCT

KTVISLGSDVGRGCITWNGIQMTLIGGLAAGAAWGLVKLIE

>tr|Q6C8R0|Q6C8R0_YARLI YALI0D17710p OS=Yarrowia lipolytica (strain CLIB 122 / E 150) GN=YALI0D17710g PE=4 SV=1

MNTLYSSHHVYLHLSNNDLLQIPYNQTLSSETPISSLTPPPGGSRLVISSPFSAQPVLYA

IDGHNLNYYDPIGDVWHQINSNALPWCSDATPFAPPVLQQGILFYGGSCSNNQALYSYNT

STKATQKQDTTVHPLGFADAAYTNIDYATTVFVGGNTSTSVWVGMNQVAYYQGGWNYRNV

DNSNMLDSRSGALLLPIYPASYSLDSTSTADRALILGGTVDGRTAEPYSAVLEFGDQGWG

YHANASFEVDGNVVGAALLFETLVTIGEGQGQDQSKEADSEGGLQVQEKVKKSALNPLRK

RSTYSIQLYNVNNLEPVQKYVPSQAATSTTPVTRASTTVSQASSTSVAATTDTSISSPPS

STTKSHSGMSTGGIAALSTILPLAAIFALLGIWWYRKKKTNEVQLPRERLDYFDDFMPRD

RDVMSDANSLNSWTEKRRIWESDHNRNSILSNKDLVNNRDSILDPDLPLPSPPRPARKAS

VKSAFSYDGNVNDVQVLVSSRRRTQLRVVNADEDLIDL

>tr|Q6C8S0|Q6C8S0_YARLI YALI0D17490p OS=Yarrowia lipolytica (strain CLIB 122 / E 150) GN=YALI0D17490g PE=4 SV=2

MAFGIRRAYPTPIVKPLWPYAVGGVITFFLFAKAANASMNTEEFINDPRNPRFKAGGVKE

EH

>tr|Q6C8T0|Q6C8T0_YARLI YALI0D17226p OS=Yarrowia lipolytica (strain CLIB 122 / E 150) GN=YALI0D17226g PE=4 SV=1

MKLLSLTLTLILLVVPALAYTEFCKCECFGEYEIIELRPQKEQLLTCNNCTRQFCLDQKL

TICKEAKGEVDVTTACFERESFKDQLIVYAFTGVTVGLLAWALFKPVLTRFRRG

>tr|Q6C8Y0|Q6C8Y0_YARLI YALI0D15972p OS=Yarrowia lipolytica (strain CLIB 122 / E 150) GN=YALI0D15972g PE=4 SV=1

MLNTCTNTTFTNNTMWAVQNPIRQAERDETDPPSPNPKWLSVAGDGQQTRAAAEMEQITS

LWNRTDFWLAWASMFLVSIALALEKMTFKVYSTFATSDFNEMSLLSALNVVQAVMYTATM

TIMAKFADVLGRFESFLLCVILFTMGEIMLAASSNISTYFAAHIFYVFGQVGIRFLQQVF

AADTSSLRNRLFFVVIPNFAYIFVPWCSAPITNAIIKHSTWKWGYGMWCIILPVVSVPVL

VILFRLKRRVKVVNGKKYDKEKYKGSRWNFIKERLLEFDMIGLVLFTGGLSLLFLAVTLV

KSQDSWKQAHVLSMIIIGPILLILFPFWEMYPKYPFLPFSAMKNKTLITGCSFCLVYSIA

QNLYNPYYFPWLLVCKGLSVTAASNTSATLMVASVASSVVAAFILRYTNRVKPVIVAGIC

IYMLGLGLTYHYRQPDQSLAKFIVTQAVEGVGQGFLQSPSLVIIQASVPKNHVVSATAIF

YAANSIGQVIGDAISGSMYRQTYPKKLADYAPFLNATEIDQMVNNVAAPLRYKWGTSERT

AVIEAFNHIYRKMLYGPMIVAAVCILIALALPNIDLSESEDGSECTIDDDKSVIVHKSSS

KTASAVIEKQL

>tr|Q6C983|Q6C983_YARLI YALI0D13156p OS=Yarrowia lipolytica (strain CLIB 122 / E 150) GN=YALI0D13156g PE=4 SV=1

MKYGSELSQRSVPEWKSHNLDYDEIKQLIKRATTDDAKDPTTETHVLEALKNEFEHISLF

VRSKTGEIDRRIIHCQKTIDHISEDDRVKEETYAAQGKSYAHHLNSKRRQTRLYKLRTEI

NRVTVEVQNLARFIGVQRTGFRKLLKKYAKWSGKQTLHQSFLPTLDGMYSFTQQDFTPVF

LELSFIFETLRQAKYQSINTYIQTALGDRDGSDPAQFDTEMVTLATNMPNAGAVAMWVHP

DHITELKVMLLKTMSFVDSPKTLRQKGEVLATSVDTNQGPSTPTLSRRPSLTLTRALSDQ

HVTEHSHAVYLDDPRKLISIQTNTEPGQIRWVDVLPKDSLNSESDSPHQTMHLSEAGPET

DPDYVLCAPVGGFRHFAASRVSRDLADAIYHSNLEDAKWEIDNLNGSGKLAVEWVAKRNA

VPLSQASMHRSRFLFEDSLSMSIGERRKSVSIFEQGTPSGSTLDNPNESSKVWATLDEGI

TVKRAGFHAHFSKDGPKSASFPYAVLEIKWKGLKPQWIEDIEKSHLVHVVEGFSIYSHSI

ALFHAQALSKPPHWMKTLNKVDLLKLPPKKVREEPTTASGNTAQLQLSRDSNTHPVSGSI

LLNSSPGSIPSLTDELSDTSQETTEPPKVRYWNEFDNPEDGEDDSGIFIYTNGDEQGLTH

DFLTEGHVNKLIRISDNLADKVHKTASKVRKLMGIKPKRSSLSDEESASDNEEESLLRGR

LYSPRLNPNYNDYDYGSAASSVSEQVTDIYGEDEFAGYGQQQQLVADRRDRVLSLFYSTC

FLLSTLIVGVLCGIIAGEDMNQVSGGAIAFSFLAFMFSTALGIVGLTLYLMRTSTPSLWH

QSFVFVVMMLNVCFGVGGFAWLLAEIV

>tr|Q6C9B5|Q6C9B5_YARLI YALI0D12496p OS=Yarrowia lipolytica (strain CLIB 122 / E 150) GN=YALI0D12496g PE=4 SV=1

MGASSSGEIPFPTYSSPPSKPRSRAISGGTTGVSSRRNTSSSSRSQQYETNYKHVASTIP

VWLIEDIGDQELSPQVNNSNSFQNDSDRITEIAEGDSDKAISDVDNGEGAAPTLNWRRRL

PTSSFNFASPQTTALRPLQGVLVAPLKLHTHIFRHHRLKYVQGESKNRYVETNPHYEWGT

FANKVIRRDVLGNEEIVDVSELEGQYDFESKYGGDDQGQLGSTSWFLKRKLKGFISLFRN

IEDDDKYKRGADLNFREFVSEKSRQSWKPKFYVILLNDPYVPLTFRTIMFSLSCVALSLS

ASVFVHSKNNTPISIPQQPSTIMAIVVQSIALVYIVYITYDEYSGKPLGLRDAGSKIRLI

MLDLLFIIFSSANTALAFNTMCDKRWVCRTDHDLSDPDPMPYNYAMCSRQKGLVAFLLMI

LFSWVSTFSISIFRLVERVAGDKQN

>tr|Q6C9E6|Q6C9E6_YARLI YALI0D11814p OS=Yarrowia lipolytica (strain CLIB 122 / E 150) GN=YALI0D11814g PE=4 SV=1

MIFRRQLSTLIPPKVASPATLHGAPNAKRMADVVSFYKALPQGAAPALPKTANPFKLYYR

KYFHPKSGKASGAPLLHLILGIFLFGYISDYQFHLKHHKNGAH

>tr|Q6C9N1|Q6C9N1_YARLI YALI0D09801p OS=Yarrowia lipolytica (strain CLIB 122 / E 150) GN=YALI0D09801g PE=4 SV=1

MSLHSSTSHDEKTSVVDPMSSEDVGKTRDIEIQELPDQILEAHIDKSKWYNKRLKIGKFN

CLPYSSSFVQMIMVSFIFFLCPGMFNALTGLGGGGQMDSTVQSNGNVALYACFATIGFFA

GTIANKLGVRATMTIGSFGYALYIASFLCYNITENGGFVIASGAILGFCAACLWCAQGMV

VMGYPAEHEKGRYIAVFWVIFNLGGVIGSLVPLIQTSVDNTELGKVGNGTYAAFLALSAV

GWLIAMFMLPAKYVVKSDNTRVIVRENPTWKSEIIALGKTIVTDKHIILLFPMFWASNWF

YTYQFNQFNQPRFNIRTRSLNNLLYWVTQIFGAGIIGLCLDSKRVGRKNKARIAHVTVFV

LTMAIWGGGYDFQKQYTREDIPTGPDADKDTFWKIDWSDSRYGGPCVLYMCYGLFDAIWQ

TYIFWVLGALSNSARKVAVYAGFYKGIQSAGAAVVWRLDATGAEYMSIFASCWALCCGSL

VLAAPVIWFYVKDHTEAEEDIQDLGPDVAVVNAAGEKDLDDKEHISVHERTESV

>tr|Q6C9R0|Q6C9R0_YARLI YALI0D09064p OS=Yarrowia lipolytica (strain CLIB 122 / E 150) GN=YALI0D09064g PE=4 SV=2

MKASSMCSNQINQLAGFILDLHLSIFVLLMLQYSSETFLNRVVEGWSGAKGPENPTCKVI

KRSTRSRRNSPESWTVSFSFFFASSFCSFSCFAMLLSSLVLSALSLVSVTAQNVVQATQD

TWDLINYAEHLSSLAICGEPYGVIYKPFQCAGRCSDFPDMELITQFTPQDPLDFSVSGFL

AVDHKRKVFWHVFRGTATLNNGLTDLRIKRQPLTSWNTAKMDCPDCQVHVGFLQAYNLAY

SEAKGAMDDTFAKYPDYQVIVTGHSLGGAATFLHGINLKTSGYDPLVITSGQPLTGNKAL

ADYNDKLFFGDNPDFTHQGPDRRFYRVTHKEDIVPRIPFWTPYHQSGGEVYIDFPGINPP

VNTLKVCDGQQNPLCSFSTSLASTATQGIVEAAHLIYFTFFFLCSTLLYPPLNSDLPVGV

WGKPLNGTI

>tr|Q6C9R6|Q6C9R6_YARLI YALI0D08932p OS=Yarrowia lipolytica (strain CLIB 122 / E 150) GN=YALI0D08932g PE=4 SV=1

MSSRRPRRIKKTPFLTKVKSFPFDLFLSINESLAVLELDNLPQTYHIYLGLAADITMILI

RLYFVRFETKKKRDAALFHSIGNNKARGGSGYYSLSAQAAAKRRSSNAIYSILMMTALAL

FAISVINMLVCFLQTKKYTLFGRDVSNVPGTPSAALEPLETPCDDLNQSGEAQFKTMIKK

RLSGFMSPAPAPPVVDPTTTDRPTRVWVLNVWEPSLFHLNIFALFSPIHVIALWFGPLSF

VQVFITLPAFSFALYAMSHMFLVYVKDKNIISGEVLGEFDKKVVHPLLGVIKQDAAVGAD

GSCEFYDAALDNKSVYREAGMAPSKPPTPVKLDTPTRRRTFVPTSGQLPGAGAQAGDGLR

HFSHRLMDDRRGSSGYTPSSFGNTGPSVPNHTTLSGSPVGRPWRRKQ

>tr|Q6C9U2|Q6C9U2_YARLI YALI0D08360p OS=Yarrowia lipolytica (strain CLIB 122 / E 150) GN=YALI0D08360g PE=4 SV=1

MLGRSYEQSIERFDTYETSYDAVSSTKDDLVLMERPLSLNEYYHSERLDPRYAPAAKEQS

RVEKLLVSRFAKIFFATILVQALIVLAFEGFIFAQFQKNLNKDIPSSSNSRSIAVYLALY

IFAEIFVCILSYLSLWKQNIMEIYGICVFMALLVVYGAIAYDQMYTAINDLTERSAFDST

SWALIKAFLVAVPCVIGLAFIIVCICAFKLKAELGWITYRNVGADLVMRRRWMQYEIFMS

ILKFDFFFFLGFTVQFIVVVLNVKDVEFGLTIAVVPLTIVFLFLLSLFVKKENKIGMLVM

QFFLLAGMAYFIFKLVRIYDPAQEYKYEAVRKSLTIFAAITLFLVTATIALSVVCILNFN

KGLKPIIEGRRKDPYGMNIFTAENDFVIN

>tr|Q6CA31|Q6CA31_YARLI YALI0D06347p OS=Yarrowia lipolytica (strain CLIB 122 / E 150) GN=YALI0D06347g PE=4 SV=1

MFGKHPKNPPGCKLLGPFALIVQASMGVLAVLTLVVKRQYEVPRRPWWVWFFDVSKQLCG

AAGLHFLNLFLSILTSSRTDVPDTSDNPCDYYFLNVLMDTTVGVPVLWGFLVLVTNIAER

QFKVTEIESGVYGHPPQWRAFWKQAGIYLLAMVLMKIVLATSFIIFPWLDYIGAFLLKWT

DNNPDLQVAFVMLIFPLFMNALQYYLIDNIIESNSFRQDYHSVPNIGEVDAPERIPQQLA

QSSYHTFK

>tr|Q6CER8|Q6CER8_YARLI YALI0B13530p OS=Yarrowia lipolytica (strain CLIB 122 / E 150) GN=YALI0B13530g PE=4 SV=1

MRPSTYIWALVLAVTTLLAPVHADLSFLDLDKPDTVDKALALIAEGLLDYYQGFTYGGTI

GMFVPPYYWWHAGAAWNAMIEYWHITGNSTFNNITFEAMKAQRGDKYNLMVRNYSSSEGN

DDQGVWAMTMMTAAERNFTNPDDDYGWLYVAQGAFNSMADRWDTQVCDGGLHWQIFQWNS

GYDYKNAIANGALFNLASRLYRYTGNTTYLEWAEKIWTWSEKVKIIDGGHVYDGIHLPNC

SDRSPYLWTYNSGIYMSGAASIYNATMLRNGTNATLEASVWMNRTYQLWNADIMKGIFFG

GNQSNIMVEIACQQVTITCNQDQRTFKGIYSSLLGQTAQMMPSLAGSILSYLRPSAYAAA

RTCSGGFDGHTCSINWLTGEYNVSYIGLGEQLSALEVILNTQILNSTGPLTDITGGTSKG

NGSAGTQGDNPFGTSQDDASPLDIHGGDRAGAGIITAAVGIMLILVFWFLIV

>tr|Q6C0B1|Q6C0B1_YARLI YALI0F26301p OS=Yarrowia lipolytica (strain CLIB 122 / E 150) GN=YALI0F26301g PE=4 SV=1

MTRHSMESNDSNDVLFDAPSSPRDALAAAFGSDSDSDSDDEHHLQSGGRMGRSYNQVDTS

TQDDDDDLNHDDDGGATLEELAQTEDPQYDRSNYADDRLGGSNNNGGHTRIPGGFPEAGN

TGSGSGGNAGSGSEGSNVFSTGFFNRLFNRGQLLPTTEPPASSSHDGVFSNTQANDDDED

KPTDETPPTYEEAAADATPPYWATTILHPGYAEEVFIDGLPVGSPISFLWNMTVSSVFQF

IGFVLTYLLHTSHSAKQGSRAGLGLTVFQIGMYMRNSGGNPTTDYSSVPAHQFVPSDPNN

FDVPGYGSNIPGNAVIPTMEHVDPTVQEQERAAHEKNVFWSGIIMAVGTLVILKALWDYW

RARRMELIVLQTPQHVVTEEDTPETAV

>tr|Q6C0C8|Q6C0C8_YARLI YALI0F25817p OS=Yarrowia lipolytica (strain CLIB 122 / E 150) GN=YALI0F25817g PE=4 SV=1

MLRRRSSASYPQQSLPKTQGTWQPSTMTQGVRTKARFYLSRMPLKILAVLAALLITAGFV

TFNLRHPAEQQPGGNKQYIKEIVFENPALHGPFVAGCRPIVANPEDKVNAAFVVLARNQD

QEGLLSSIASLERHFNQWYNYPYIFLNDEPFNATFKAAISKSTAAKTEFGVIPDSLWDFD

DSVDRKELKKALQNQADQNVMYGDSESYHKMCRFYSGKFYDHPLLKDLDWYWRLEPDVQF

FCDLTYDPFRYMQDNNKVYGYNIAIKELKETVPNLWRYTKSFKQKNKMQSKGMWEMFLQT

AEEREQRGGHPLKDARMSDKNDKVKGKAETNNPTSKRFADPDYEDMGEEAYNMCHFWSNF

EIGKLSFFRSKEYRDYFDYLEEKKGFWSERWGDAPVHSLAVGLFLAPSEVHYFRDIGYQH

SDIFHCPYNAKGAQLPPPERYWEGVSKRTGLKLTDEQIKEDEKWLVPDPAEEGGVGCRCI

CPLDKAAFIEPRDGSCIAHWVETRDKGL

>tr|Q6C0S9|Q6C0S9_YARLI YALI0F22033p OS=Yarrowia lipolytica (strain CLIB 122 / E 150) GN=YALI0F22033g PE=4 SV=1

MSWQSVLVKKPDDDGPDLWKHSSLLKTQLLIAGSLGMTAFLSFCVLRKNWPQIYAARTLR

RKGLPPLPNTYFGWIPALYKITEEEVLEHCGLDCYVFLQFFKMAIIIFALCTFFAVTVIG

PIRRNYDNGDEDGEAGIDVGVILLRLILTSFGVTTYRASGNATEEEDPLGDKKPKVPPPQ

EDYQPYLWTYVFFTYVFTGIVSFFLMRYTQKVIRVRQRYLGAQNSITDRTIRLSGIPTEL

RDENRLTEHIEKLDIGDVRSITICREWGELDQLFVVRKEIIKKLEGAWCVYLGPREVDRE

TVIGVVPTILPSNNGHILDEPSNTNNPSSSSSSVSTGQNTPLNTPRATLQLGSRTHSYDR

PTVRIGGWHGFTFWGLYGTKVDAIDYYTGQLDTLDQKIIKARHKEYTPTNTAFVTMDSVA

SAQMVAQAVLDPSPYHLIAKLAPAPHDIIWKNIYMSTMKREVRTYAVTIAIGFLTVALVP

PVLGVAKLMDTKTISKSWPALGQLLKDTPWLEKLVTGILPPYLFTILNFALPYFYVYLAS

LQGFISHGDEELSVISKNFFYIFVNLFLIFTVAGAAIDIFQYLKDTTSIAYKLASSLKKW

ALFYVDLIVLQGIGMFPFRLLEFGNLLRFGFEKPITHTPREFRSLYTPPVFNFGLNLPQP

ILILIITLLYSVFDVMSSKILAAGTAYFVLGYFTYKYLLMYAMVHPSHSTGQAWPMIVRR

VCVGLVLFHATMSGILALQQAYYLATLLAPLPVCAMLLLYNFENNYQPLTQFIALQAIRD

TGGSGVSFTDLEGQRSMRHMRSQTLDEARERFLTYNNPHLTARLDGPWIGNEGDQVVIAN

QEGTIRRKVRLDEFDS

>tr|Q6C3H0|Q6C3H0_YARLI YALI0E34903p OS=Yarrowia lipolytica (strain CLIB 122 / E 150) GN=YALI0E34903g PE=4 SV=1

MDEKNPGILQTRVFEEQSADSTNEGQTEICTIMSPSNLEKGEESDLPPDQVEPPDGGYGW

VCVACTMGVCGTGWGLNSAYGIFLAHYLSYDVFPGATPLDYAYIGGIGIGAGLLAAPVAN

LAIRNLGLKPSLLIGTFLQLAGCIVASFATHIWHLYVSQGLLMGLAIIFLFAPAVTVPPQ

WFKKKRSLANGLTVAGSGIGGVLIMLAAQEWINKWGLAWCFRATAIMTFVINMTAVILIR

EISKVKPTFAVWDFKFLKQVETNAYFLWVFFSLAGYVVMLYSMSPAAKAAGLTDNQATNI

SALLSAFMAFGRPFIGHFSDRYGRVNIVILSAVLVIVLEYALWLPANSYGMYIAYSILNG

FVLGTVWVGLGPIAAEIAGLSSFQTLSCMGMVGMSIPGFFSEVVAIKIRRDGDKPYLWPI

VYSSVAFFFSILSAFVMREYAIRRQMVAKREEDPSFVMPGYWCRMFKWGRA

>tr|Q6C3H2|Q6C3H2_YARLI YALI0E34852p OS=Yarrowia lipolytica (strain CLIB 122 / E 150) GN=YALI0E34852g PE=3 SV=1

MKFLVCLLVLALASVVSAHNVLLLPWKRQCFYEDVKKGDTLAVSFQVGNRDPQAGGQLAV

DFVINDPHGNQVVKQHEVPDGDVQVPIQVPGRYEYCFSNEFSGIGTKDVTFNVHGVIVID

LNQLTDDALDQEIAKLNQVVQEVRNEQSYIVVRERTHRNTAESTNARVKWWNILQLGVVA

VNSLFQIYYLKRFFEVKTLV

>tr|Q6C3M5|Q6C3M5_YARLI YALI0E33583p OS=Yarrowia lipolytica (strain CLIB 122 / E 150) GN=YALI0E33583g PE=3 SV=1

MPFSEAPPLPQGAGYGILVGVGALFAVFIVFTLRVSLNYLGEKANKSEMFMVANRAVGVG

LTASAVFSSWMWANETLYGAVVGYNFGMSGPFWFAAGLSFHIALMTVVGIQVKLKVPNGH

TLLEIIKFRYGVPGHIVYMVFCLINNILSCSTMILAAAGAISAITGMHSAAASMLIPVGV

ILYTAAGGLKATFLTDYVHTTVALVLLMYFCIGILTNEHIGGVYGLYEKVLAYSDNADHY

IKGNYQGSLLTFKSQGGALFSICHNIANLGLVIMDTAFWQKSMAADLKATVPGYMIGSVL

IFCVPWALGTISGLGARVLEQSGIIDPISAHALDVGNVFPIVTHALLGKGASVGIVLMLF

MSVTSTVSAEMIAVSSIISFDIFRTYIKPNASDRVLIFVSHAGVVFFGLLSGAIAVAFYF

GGIDLNWMSYFLGIAITPGVFPVILTILWKRQSRLAALVSPILGLATGLGVWLGTAQVYF

GEISVKSTGAPLPNVWGNIATLMSSLLFSVIITLIRPEDFDWLIFHNISKVNEDDEKPED

LEAIHENPTDPFFISDSDAPSTSKTNEVSEVIINHEIASSENDSENESDHGDPTKQTHDS

STHSLSSGSYHVAVVREAATSNGLFQPRLVIPQNISTWRKFLYYIGWDTAHTDYSNHPLG

ADSIPIMLYWYKVAKIFAVVICLITWIIWPYSLYRHYIFSKSFFSGWVIVAIIWVFLAFI

FVVIFPLYDGRKSLYRVYDGLRTDWKKWRHRKDGQAEEEKE

>tr|Q6C5R8|Q6C5R8_YARLI YALI0E15730p OS=Yarrowia lipolytica (strain CLIB 122 / E 150) GN=YALI0E15730g PE=4 SV=1

MVTQQSAAETSATQTNEYDVVIVGAGIAGPALAVALGNQGRKVLVVERDLSEPDRIVGEL

LQPGGVAALKTLGLGSCIEDIDAIPCQGYNVIYSGEECVLKYPKVPRDIQQDYNELYRSG

KSADISNEAPRGVSFHHGRFVMNLRRAARDTPNVTLLEATVTEVVKNPYTGHIIGVKTFS

KTGGAKIYKHFFAPLTVVCDGTFSKFRKDFSTNKTSVRSHFAGLILKDAVLPSPQHGHVI

LSPNSCPVLVYQVGARETRILCDIQGPVPSNATGALKEHMEKNVMPHLPKSIQPSFQAAL

KEQTIRVMPNSFLSASKNDHHGLILLGDALNMRHPLTGGGMTVALNDALLLSRLLTGVNL

EDTYAVSSVMSSQFHWQRKHLDSIVNILSMALYSLFAADSDYLRILQLGCFNYFKLGGIC

VDHPVMLLAGVLPRPMYLFTHFFVVAIYGGICNMQANGIAKLPASLLQFVASLVTACIVI

FPYIWSELT

>tr|Q6C6A3|Q6C6A3_YARLI YALI0E11121p OS=Yarrowia lipolytica (strain CLIB 122 / E 150) GN=YALI0E11121g PE=4 SV=1

MGRPPGSGMDGPVYTDYDPAIMEKLNLHVDIENGTLTNGPQCWLSFGDHIPTFVHIEYLD

TYAINGTTCDSPYYSMGNKGYLGVMLSVGIAAATLVTFFNLRKHGMNFMHEPKAEELMSQ

GGRPSKTKTAEPGWIRGRRLPWFLLLVLGLMLILTGFFAVDIDRNYIQGTAAGMYGVFLS

VCPVLGLMVSWEFIRRWGFFEERKLLEQDPYAIDQTDLKSKARIIPPIVFYVSCFFAMFM

MVLRNWSKHLDYNTFFDLDVRWVIGGICEVFAFIQLVNSLRVTITYYQVPQVPLVLPFMV

FMNLLTEIYQVTVMVVNDKTVSPFYPSSSVVLPVFLVYLPLWAITIAGNLSGLARDNEDQ

VILRLRALRWKIKQENAERARSQGEQEQEAAQIAERRETDATFNEQYPHFEDSDAEADEE

GSDPNASGGGRQTSRYQKLGNEGGNRAGLFKRLFGEYRPTNTWFGHNMAYVPLSRTKNQL

FDEEDFPAITLQKTRVRSEVNEDPDEMEGGFDNYHKPSLFSFLSNSFRPIPSKGRRDLND

LEMQDLGTPKSIKQQKLYGGRRGSRP

>tr|Q6C6F6|Q6C6F6_YARLI YALI0E09933p OS=Yarrowia lipolytica (strain CLIB 122 / E 150) GN=YALI0E09933g PE=4 SV=1

MEREAIFRSAEMSLVQLYVASEIGRDVVAALGELGVVMFRDLNTSVNVFQRSFIKEIRRV

DGVERQLRGLRAHIDKHGVAIDEQPEGVAAPTLDEVDNMCHQVGALEERVGHLDTTWNEL

VDKRALILERREMVQTAGIFFADARENRHEIRASLEGDRAGLLYDLDDPQPDVEAATVTW

NSVAGLSFVTGVIPSTKTAIFERILWRSLRGNLYFRHQAIEKPLAGVRKDVFIVFGHGES

LLAKIKRIALTLDATLYPVSEDFDTRREQVEELNIKLADVDNVLGSTNNALMTELALAAN

TLPHWEVLANKEKAIYHTLNMFNYDQTRRCLIAEGWIPKADFRAVQEVLRDVTLSSGVAI

NSILNEIKTSKTPPTFHRTNKFTAAFQLIVDAYGIASYQEINPGLATVVTFPFMFAIMFG

DLGHGVILALAGLVMVLKEKSILKMRNRDEIFDMAFSGRYIVLLMGIFSLYTGLMYNDIF

SKSMTLFRSGWAWPESWEEKERITAHQTGVYPFGLDPAWHGTDNNLLFTNSYKMKLSILM

GFTHMSYSFFFSFLNYKFFNSQIDIWGNFVPGLLFMQSIFGYLSLTIVYKWCVDWIAKDK

TPPGLLNMLINMFLSPGTIDAPLYPGQKFVQIILVLIALVCVPWLLLLKPLYLRRQHKQT

QYDAIRQPNAYHIGDTDDDADSFDMTIEEFEEEGEGHEQFEFGEVMIHQVIHTIEFCLNC

VSHTASYLRLWALSLAHAQLSTVLWDMTIQGAFGPTGPAGVAMVVIMFAMWFVLTVVILV

MMEGTSAMLHSLRLHWVEAMSKFFEGEGYAYAPFNFKDQQ

>tr|Q6C6J9|Q6C6J9_YARLI YALI0E08969p OS=Yarrowia lipolytica (strain CLIB 122 / E 150) GN=YALI0E08969g PE=3 SV=1

MSLCNDPEGWQLLTNYYDVSPCVSQNVFFILPGSVAALFGVPAVVHLYFQPGKRPAVWSY

TLKQILTLIQLSIAVSIPFTTTFQHDLRHWSPLVLAFGSIIIFFIQKLEFEKRKPSSTVA

LSFWGLQTLDSAIVVYGLGIRGFDEATYVVSLISIAFLGLFNLVLETTYSPSSEPAFYDD

SNLFVKLTYSYVSPILSKGINSTLKIGNVPKPPLELRSQYIFREFSEVWGPKIDLYTKAA

AKDPETAKFPSLIATFSYIHGYDYLKISLLQVFSIVVPFVQPLLLKQLILFVAKYNNGLA

PLSQGISIVFAAGSMMLLRTIIESKEELMTNNLMLRIETALSQTVYEKALRLSTAAVADT

SIGEIVNILSNSVKQLTSVVSYLHMIWSIPLQITICWLTMYSMIGNAMWVGMAALLLVVP

FTAFISKLKMGLFLAMQGICESRYTVTNNLLSNIKSVKLYGWEPTFYGKVEKIRNEEELA

IVRKMSYLSAIESILMRTCNNLAATASFAFIVLFQHIPLSAASAIPALNLFTRLLMPFMF

VPYIVQFGIQAWVALTKINNFLGLTEVEKFNGQEHIPDSSKSVPVNVNGSFFWDKQLEKA

ALENISYTADKGATVCIIGKVGAGKTATLMATLNELFVQNGSTSVTGSVAYSSQVPWILN

STVKDNILFGSREDPIFYNLVIEACALTHDLQLLADGDQTEVGEKGISLSGGQKARLSIA

RAVYSRADVQLYDDPLSAVDEHVQAHLIKNVFGPGGLLSSKTVVVATNTVNLLRHSSTIH

LIEDKTFVESGEFSELMTRDGKIKKLVDEFQTQAGESTPDASKVIDAIEVDDKRIESDAK

HPFSLRRASSISHFSVITVADDDARRTRVEDEVKETEALNFVELYKKYFVAVGYINMGVY

LVLSLVGSALTIASTYWVAEWGSDKIDLSDIQLVLGYFFIRFASALFEAFGGLAFSTFGA

VRASKLLHERMLKAVLRAPMSFFEATPLGRLTTRFSQDIAKLDWMMNNFITRLATAVITS

FSSLVLIVGSSPSTLVVVPPALYLYRIIQKYYLITSRQVRRLSAATMSPVVSHFQETLNG

LTTVRAFGKSRFFSTKSTAHIDVRTKMEFLSYSLQQWLGLRLTTIGVVIFLSSGLSLVAT

LHWKPLSAGLVGLVMSYASTISSCLSEVVKAAISVEQESVVLERIFEFCQIEPEAPPKAK

EPAAHWPNEGRITFSNYSTKYRANLDPVLNELSFNIKPREKVGVVGRTGAGKSSLTMALF

RIIEASGGSITIDGEEISNIGLQDLRSRLSIIPQDAQMFEGTIKTNLDPSGKFSDTELLQ

VLEHASLKKFADDNEGLETKLSDGGSNLSLGQKQLICLGRALLTPSSILVLDEATAAVDY

ETDKLIQKTIRREFKDRTILTIAHRLNTVMDSDRILVLDAGNVVEFDTPEELLKNKNSLF

YALVNANNGDKLDT

>tr|Q6C6L8|Q6C6L8_YARLI YALI0E08382p OS=Yarrowia lipolytica (strain CLIB 122 / E 150) GN=YALI0E08382g PE=4 SV=1

MSYSAIPSEPTDSAPGVVAVPQPSSPKAGLIQNFIFLQVKKSIAIAVVVLVSQGASLNAT

LEGVLILATSILPFWTPPMWTPNMAPHKFTAIVFYACWLLQLGVQFLCLDFNPSNQFSKV

LQISALNLVTGFLGNQIKYSQAYFVKRLYPADQQIKATKYAFLFAFLYPQVLESVLVALY

LNVTFLPAWTLEGFRAAAYLYFAADLYFRYPVESESGVTAWNDSSLYSFYKTELASVWKG

ILFYYYFFLQFL

>tr|Q6C6N5|Q6C6N5_YARLI YALI0E07920p OS=Yarrowia lipolytica (strain CLIB 122 / E 150) GN=YALI0E07920g PE=4 SV=1

MTILGEDDNKVKELVKGGIDDIKSIDVKKSVQKCVSGASSGLKNAYETVKFKTTTREGLI

GDYDYKFLLTPKIPFMKKSKKGDKNRSTFFAVDSSLPLLLAILLGFQHSLAMIAGIVTPP

LLMASAANFDAQMTQYLVSAAMIGSGLLSLIHIIRFRIPGTHLYVGTGLISVVGASFSTI

TVFTTGLPIMYKSGVCPVSETGAPLPCPEGYGMIIATASLCALIELLCSFLPSKFLMKLF

PPVVTGPVVLVIGVSLIQSGFKDWAGGAGPCFGRPTSGDFMLCPSNDAPMAAPWGSAQFI

GLGFLVYVAILTCEKYGAPIMKSCAVVIGLLVGCIVAAACGYFDATPINEAPVATFLWTT

TFPLKLYGPVVLPLLAVYLTIMMETIGDITATCDVSRVAVSGPEYESRIKGGIRGDGIIG

SIAGLLTLTPCSTFAQNNGVISLTKCANRQAGGWACFFILMMGIFSKFAAAFVSIPKPVI

GGMTTFLFTSVAVSGLAIISRNPITRRDRIVLTASLVLGLGATLVNNWFSFVFTYEGDNQ

SLQGFLDAIQLVMSSGFSVTGFVGVIANLIIDDDEEEDEDDKEHTLEICSSSSSITQVDL

EAGQKVE

>tr|Q6C6S2|Q6C6S2_YARLI YALI0E06743p OS=Yarrowia lipolytica (strain CLIB 122 / E 150) GN=YALI0E06743g PE=4 SV=1

MSSHPGESLTHLYEMAPTPLTKLGLLSFCVALIFVVFIFGRSLQRIFFGSLHPREVVHLQ

ERAVYTISEFAMQLFVFRAQLNRRFVGMFATLLFIKGLHWMCTVRTRYVYLEYPNSGIVM

RYPRLILALVILHVTDILWIRYCLRKLRVSQSMVVSVFLFEITILFCSLLGSTGIMLFDL

VEKALLYMCLSKGQLVKSKRYWLFLLGFAVTTAKLISYLIFSATLMGDYCIPLHIFREFY

KTLRITISGTRELIKSRKTPNFHGLYWNLQDASEKQINESNDICVVCRDSMKAGGLSGVQ

APDKNIPKVLTCGHIVHFGCIACWSEYSNRCPTCRRLVE

>tr|Q6C707|Q6C707_YARLI YALI0E04741p OS=Yarrowia lipolytica (strain CLIB 122 / E 150) GN=YALI0E04741g PE=4 SV=1

MLSVYADQHREAPFPKKAYKAELQKTTENAGLGLQVSEFLSEAEIIHSLYYRYRNAHHCA

EWFRPFSMLHRRVSQVANWIVDLHKATKKRAPRLITAIKISIQKIQTRYLQPAFWSFYSV

LALGQYVTVGFALLGTVARIASLLAQVHPIPRTVGKVLDAAVKKGAEVMSDDIGEVLVRV

TSEKPAKSDKIKKMTKSTVTKTTTGGKKLLKNMDIDDIFSTKKKTKKNIEKPRVVEENVE

ESIITNDDIEDNSMLTEPAPKAKKVKVAKVENFFEESKDKKKKNPASLDDSLEPPKKKKK

ILDSTDGMKKKKKKSKKNAIDDIFG

>tr|Q6C722|Q6C722_YARLI YALI0E04422p OS=Yarrowia lipolytica (strain CLIB 122 / E 150) GN=YALI0E04422g PE=4 SV=1

MTRFCDTVKTLIKEPSFYQFTGTVAGYTLFAAAFPVVAAIHTARGVALLKNKSLESCECE

NKLAKKFDISNITLNEWYSPITLLFPLFKRVVEPLGPLAVMWARYTGLVGEMTDKEVNCV

FNCWAKISLGYIYVTGAICFTGLSVWSYRRQTQRHIKKQSIVGVPEVTLALFTGLLFV

>tr|Q6C762|Q6C762_YARLI YALI0E03476p OS=Yarrowia lipolytica (strain CLIB 122 / E 150) GN=YALI0E03476g PE=4 SV=1

MQQEVYHFKVSWHHSQGARVCIIFRPCVISDGPSRITFIEPLRLISTRIAAHMIKMRLKF

HHLVFTVGAVLSVLLFLSLCPLTLKRIPSNYINTAVDGYYFQYHRLTHVRHRKNWFRPSV

RGIIDGSFFEVDSTAPWRVAYSSNKPGVAPAAVTQPNQRPTIVTLNAGDEDSDILAAWKR

AWYAAGFNPVVHIETDHSLQHKMEAVSQANALIFCDKHIIPLDFRPNLPGGRMKEEFSEN

REKNGHHGRNSHRWKPSEYMPDLYDDNDVIDKLKGTPSKTTIYSQIEDSLHMGLVHFESI

EFIRDLVQKLSSDRVSPGEASRDFRVSMENTRALAVYTPANLETITGYSSLHEDTVAGMI

SSHKRNMFSAVFSNGLQVVDPYEGMSQIFSLPSLRLAQRLGACPNVPLAKTCPPIAPVLE

YFKLHKLTEKSHDREGEAQLVAPKFNEEHIRELQKLGCMICQDQSQIRLLRAYNLRPRTF

KIGTLAHPLTRVAAEHVSTDVDIEMVYDSSTAYESWVHNVTHRVISSDRFGSVPKVLMMK

DVLYKSFFEQSNSIWLTWEDSFVPSSETNNDIEYHLGFVLPPEPDSRVHVKSFKSIGEGL

VSPHEFFNNGETIMKAINARLHNDDGNDSILNTVEGWNIPDTEIWRFLQELKSLHRRNQN

IN

>tr|Q6C7E1|Q6C7E1_YARLI YALI0E01562p OS=Yarrowia lipolytica (strain CLIB 122 / E 150) GN=YALI0E01562g PE=4 SV=1

MLHLLPPEVLENVLGNLETLNDLLALSHTCHFARSVVLHNDQRFKPHVQHLCQYMTPVSS

WHSSLLRLPRNANNLGTPVDDTWTMSPAVFATEPTKDLLRLPHERLQTHFNRSPQQITHL

FHDVRHRSLYGPIPDSQDTLFELRSGVGPVTRINMSTMSRSKAPPHSCLHHLDTKVAIIE

KFPQLNRLNHMNKRVVTGSRQHLRNVTTEVLVIDNDGEAVPLATFTDTISCRGEPHGSLC

LSVAVCGTYVACIVGYSLFLFTKRDDQLYLADSYKLDALYSPVSDSHNQECLLLQRSRSS

GPGHDILFNISSKTVSVVEGGIYAISIDTNVWRFDDAFLQQCSVSPQEEMHWTSAEVMCG

>tr|Q6C9V5|Q6C9V5_YARLI O-acyltransferase OS=Yarrowia lipolytica (strain CLIB 122 / E 150) GN=YALI0D07986g PE=3 SV=1

MEVRRRKIDVLKAQKNGYESGPPSRQSSQPSSRASSRTRNKHSSSTLSLSGLTMKVQKKP

AGPPANSKTPFLHIKPVHTCCSTSMLSRDYDGSNPSFKGFKNIGMIILIVGNLRLAFENY

LKYGISNPFFDPKITPSEWQLSGLLIVVAYAHILMAYAIESAAKLLFLSSKHHYMAVGLL

HTMNTLSSISLLSYVVYYYLPNPVAGTIVEFVAVILSLKLASYALTNSDLRKAAIHAQKL

DKTQDDNEKESTSSSSSSDDAETLADIDVIPAYYAQLPYPQNVTLSNLLYFWFAPTLVYQ

PVYPKTERIRPKHVIRNLFELVSLCMLIQFLIFQYAYPIMQSCLALFFQPKLDYANISER

LMKLASVSMMVWLIGFYAFFQNGLNLIAELTCFGNRTFYQQWWNSRSIGQYWTLWNKPVN

QYFRHHVYVPLLARGMSRFNASVVVFFFSAVIHELLVGIPTHNIIGAAFFGMMSQVPLIM

ATENLQHINSSLGPFLGNCAFWFTFFLGQPTCAFLYYLAYNYKQNQ

>tr|Q6C9V6|Q6C9V6_YARLI YALI0D07964p OS=Yarrowia lipolytica (strain CLIB 122 / E 150) GN=YALI0D07964g PE=4 SV=1

MSTALKKSEIRNRKDDAKAINVKDVPVSPEGAVQEEPRHPRREASGTFEEISVTSTSENI

VSCFKLVTDALAQRRNRANRRVIFHYYTLGALLSLYVLVRVFNDPVEDFGKFLVFMCAVT

IATLSLASRTTDKLIRDAESSVVADVLVPQSDTKAYLFNGTVVGVCLLAVSDEIDKENEN

NYYKFPIEDQAVYITAWTVMRKYRRTGLGMDLLFWAVNTARDVTSKKVKPNANRIIIETI

SAENEAENLLMAAGFELNNSRKIEGWMGKCGMEYRQWILHMDAIGAIMEEKVEEASAPTI

>tr|Q6C9Y3|Q6C9Y3_YARLI YALI0D07392p OS=Yarrowia lipolytica (strain CLIB 122 / E 150) GN=YALI0D07392g PE=4 SV=2

MSHYHDKNDISTFVESGSTRPVSASSLDSSIRPQFPESHLYDDEKPSPDVSVHSLPRDTW

FEKFIYYSSKVDSLGVELRGIQRIPLDERKKADTKAFIDISLFWICACGGLTSMQGMMLG

PLAFGLSLKDSLVSGLLGVFLGSGVAAYGATFGPRSGLRQLVGARIFAGWWPTKVFALLS

IIGGLGWSIVNCVVGGQVLGSVSDGKVPLEVGITIVTVVSFFTAVFGVRAVMWFEKVFAI

PLNVVFLLLYVCSHSYYDVHAKSQGTHLTIAGNWLSNFAACFGITNTWMPLAADYYAQYS

PSVKNWKIVTVTWLSIFIPSAFVGVLGTLLSTGVNTNTEWAAIHDKWGNGGLITAGFAKW

KGGGKFLVVLLFISIICNNILNTYSFALSIQTWGRAVMRVPRWFWSFICCCIYFGCSMGG

RYKLSEYLNNFLPMIGYWAHIFFLLILLEQILFRRKKLPVEPGHEDENDPYYRWYLWDSP

ERLPHGIAAFCSFLLGATGAVLGMSQVYFQGPIAKHVGAYGADLGLEAITFFVCISYPIF

RYLEAKRWPERFA

>tr|Q6C086|Q6C086_YARLI YALI0F26917p OS=Yarrowia lipolytica (strain CLIB 122 / E 150) GN=YALI0F26917g PE=4 SV=1

MPVTQAETPNSLLSKKLSTVVFFALATYANIQYGIATSHSDHDGPDWIHTNAFSASSIFI

FIFWILTFGFQLLYLSQVFSAEPAAISGSITYHFSVFNILHFLWAWLFARGHLVLALVVT

AANFFNVLALYISHKTYSLRPASNFIFIHVPVSALPLAWTFYNIFWTGAAAFHANGLLAR

IIANVLIWDFLIVPVLILFFYRDWAFGFAVQFLTWGIGVHQLFYKVIALQWGFAFAAAAI

TFVLTVLTAVGLIFKKPAVDQVDAENERAPLISNQN

>tr|Q6C0D4|Q6C0D4_YARLI YALI0F25685p OS=Yarrowia lipolytica (strain CLIB 122 / E 150) GN=YALI0F25685g PE=4 SV=1

MSTWESFRATGLASVESSVSVIAILGFGYYCRTKNIVTDSGEAFVSKLSTHIFLPCLLIT

EMGPHMTIESITQYWPLLLAPMLVLMLTYFIGNNIGHKLLGFPKYITAGVMFNNTTSLPL

LMLKALGTTGALDVLIPEGGKLEDVVKKASAYVLLVSIVHTIARFSLGPIIMGQKEEKFE

GETTPLLGGTAGRLEQNVETFYERHISKYINAAVIGGLIAIFIGIIPPLKWFIFDFTPMK

ASLTQAVTDLGELYPALQLFVLGAKLTAKPSVPVKPSYMVFIFCTRFILVPIIAISSVFY

LRQANENVWTRDPILDFILMMTPAGPPAITLAAVAELGGVGEDELASIAQMLLWSYAITP

FIAPTVAVAIQVVKGEYGL

>tr|Q6C0E2|Q6C0E2_YARLI YALI0F25509p OS=Yarrowia lipolytica (strain CLIB 122 / E 150) GN=YALI0F25509g PE=4 SV=1

MYKYMTRAGSKVACRFTRNMSTQAHKSSPGKFRKLVRLTAGGCLVGYTAAVFFASRDERF

RDIFIEKFPFGRPIGEYFEEQEIKKNRDSPKPKPIPTSPSGQPNARTSHLSALYPDKSNP

NAMTNNPDGDSHQERKFNLERALSSKGTKKEYFDPLTENQGNFFDPSSGVVAEEHKEYLP

LILLHDSADHAARHSAMALNDLISSINTSIVTNDSVKNVSDSLIQLSEFVAVNHPEIQTQ

LSERLGEKARRFHALGEHYHTIIQDFLQNEVEHGPHQGHAAEVELTIYETDKMRDLRKNV

IQEIHDTEDLLVKYCNRYSRPEGPVAEVITRRVPTVETVTLEPIDSTAKAAFDASKYKSQ

ISAAEQSLAVLVAAVQHSSVLGVDPFVEGVKQAINNVDIGDRSKILTEALKNVTVPSDVN

LTPYLEQITDKSS

>tr|Q6C0G6|Q6C0G6_YARLI YALI0F24915p OS=Yarrowia lipolytica (strain CLIB 122 / E 150) GN=YALI0F24915g PE=4 SV=1

MESVFRIILFIMLMGLLMSPGGNRYTPLSRKEKRQLEQLVANHKFQADRLRNHNAHDYTF

GNLTGFTFGYTKPRKGDKRQQLGCRDEGDKVSLFPQDVLNEASSYWKNEKGLYLRNISGV

VRGDWTGPANDTQKLLHIPMPVSQQNWTWHPHNHSVSITPLPIDPDDDGGDEGEQRINGN

ITAVDGKVELDFIDAIPSYVNQRILSSYFANHTVPKELEAFADVNVVTCTAQLMGQESDS

ELHTVVLRGLHFKHSGNVIMTTHSAKFWGYEALAGMLAEIMQTADRSRESTDDAFNTTKE

AMMPIIERHLSIIGDTDQAVPVSEMEQEAAASCEYIGVLHVDSVDANQYSGIDLRNIESE

LQYPVGRPHKRPPRLQMSGVLYSPDCGKKMTVGDAKGLLWNASLMKQNHVVVAVIFLGII

NTLLLAFQMQKASTPSLCSRVSIWSIGVMSMMDGFMCMFSLMAILYRTQPQLQFTALAFV

SFTYVSLFGLRFMLNITLSQIPEEFARPAAIQTPGGNAQDNTPGGDDTLPVTETQPAATP

FIPSDADVSTALYGRFYFCLLAFVIVTTVTYGMTAPRRYVMEYVIVLGMASLWLPQIYRT

AYRGTQQAPLAWYFMIGSSFTRLLPLYYVCLVKDDVFRHRIDRILPTLATLWIALQLFVI

YAQHKFGPRFILPQHILPPIYDYHPIISQDDIESGSYGALFENAANGSLEDAHTDCSICM

NPVELVVQTSDEQNNMDPAKLVARRQYMITPCRHVFHTDCMSNWMIRKLQCPVCRNPLPP

M

>tr|Q6C0I9|Q6C0I9_YARLI YALI0F24299p OS=Yarrowia lipolytica (strain CLIB 122 / E 150) GN=YALI0F24299g PE=4 SV=1

MVYDILATAKNQGQGSYNSDGSPRPGFLGRVYWMVIGAILAVITIFRVLSLLRQKYYLKR

QKPPRSDSVPAKIIGAIVSTAREASLLQIPGFDIPWVNYTVEFPPVGDILIILFYVMIVM

LLLFSKIDPFNPMNYETVAYRCGWVSISQISLIVILAARRNLVSILTGISAQRLNLYHRW

TARVLFLCVILHLAHFIKTWGVTHQIQNQVSTNTFALKGFGAFGILCWIFISSFAPFRNM

SYEFFVFQHIVTMIGFMVAVVIHAPNYAYHVVIPPLALFGLDRVLRLVWNLFINKAGLKP

HQTALRVEPGAVVATIPTGLSWYPGQHMYLRFPNVSPLEQHPFTISTLPGDDMQFVIRSK

AGFTKRLLKKASQLPEHEKMTLPVYLDGPYGETRDFKQFDTSVFVCAGIGASFCLPIAVD

IIRNQHKTIARRLKFIWITKRFQDMELYAAQLTELLECARTFYDTASGIDVVVEIFATCE

DDCCGAVTNCSSCCCDEVAVSRGECCCAAGIEPQPKNELTQAQTALAQSSTLSPVYYDFV

EAGYLRLKQGRPQPYQYNRRKKLGSDKSFYRLISPVISMAQGESGVCICGPRRFTRIVSN

TVVDITDDRAVLRGTGNQAVYTHVEHFGL

>tr|Q6C0K8|Q6C0K8_YARLI YALI0F23837p OS=Yarrowia lipolytica (strain CLIB 122 / E 150) GN=YALI0F23837g PE=3 SV=1

MIRTLRPHIIGLPRSRILQATLRPPLRAGIQHPVARWNSSKPTQTEVLKSDIKSPVLKKL

NLDPLKRPATLLPSEIHENLYTLPNILTFTRLLSAPLIGYFVIQHEQVLATGLFVYSCVT

DMLDGYIARKFDMKSVVGSVIDPLADKFLMITLTVCLAAAGEMPVYLTTLILGRDFGLGL

SAFYYRYISLPPPKTFTRFWDFSIPSAEVNPTTISKYNTAFQMLYIGLVMSKPIIGTFAD

MAAFDVGMTYYGYFVGATTFLSGMSYVFSKTAVKILPYPQQAKPMVFEGKKLNEK

>tr|Q6C0M2|Q6C0M2_YARLI YALI0F23485p OS=Yarrowia lipolytica (strain CLIB 122 / E 150) GN=YALI0F23485g PE=4 SV=1

MVRLQRRLATLGIAILGLTVISISIYSLYGDSNRVLEFAGAHTDWNSHSSAPVDSDPRPK

DDAPAPIELTAETKPDTEKQSAGQRPKSPTAPAGVAFEMHPDAFHAGADANRHMGHDVWR

PHGYVPSYPLGGVHRAPGIDPLQVGNAGEFMQQKLVADFAEGSDRLFLMLKTGATTFWKR

MPIHAYTTLTRVPHFAIYSDHPDTIAGYEVIDILANTSQEYMDSQDFRLYRQQQKLRELH

NQVDFGTMKLDGGWELDKYKNIPMLQHAWNTDSTKDWYVFIDDDTYVFFDTMLAWLKELD

PKEPLYMGSPTSVKGVTFAHGGSGVVLSHGAMKAMFEDAEIADGFDTLQHEYEEHTASVC

CGDYMVAKVLLDKAKVKLSMTSASKYPYIARKIQGEPITTLKFFQDNWCQPITTFHHLTN

RDIELMWQYERSKGPEFRKHITYQDLYHDFVKPYISDDVREDWTIEREDTDGDGQESSLA

KEKEDWKNKHTKKKSDDDEEDKEPEQEIPPFEPLKDSPRQSIDKCKAWCQSKDWCYQFVY

TGKDGDENSVCKIKKYLSLGYPVTKGKKKRVGWNIEHIRMHLRWSTDCDELAGLEEADAK

GLDPDEGWWKSRFQADREAERLRQQAEEQGAQAPEGENEDLKMKEAEKQEEVKDEKKEEE

KQKEEKKKEEKKDEKKGDEKKE

>tr|Q6C160|Q6C160_YARLI YALI0F18964p OS=Yarrowia lipolytica (strain CLIB 122 / E 150) GN=YALI0F18964g PE=4 SV=1

MENISDEVVYTTEPPQTTDEKKDQTDYDASIAEVSMTTVEWVAQQLLIPPSELDGSYPED

VHYMARKVEEMSNVTAASIIRANIDYHMNDKNLPAGMMDECRDLLEEYTHGPEGEKEDEM

VGDYAGDDFQPENSLMMRYYAALFHDWSPYPQVRSVTTPIPADPNEHVETIRCYIVGTIW

VAISAFVNQFFYPRQPAISLTTPVLQLFLYPSGVLLQYILPKWGFKLWGIDFALNPGPWS

AREQLMATLMCSVAAVPPYINSNIIVQYLPMFYDQSWALGFGYMFLFMLVTQYMGFGLAG

LLRRVAVYPTRAFWPTSLPTLAVNKALLDGHRKETVSGWKITEYQAFMMFGFGAFLYFWV

PNYLFTALSTFNWMSWIAPNNINLATVTGSVTGMGFNPIATFDWSVITALVQPVYMPLYT

IVTQYVGFVLSGLCILGVWYSNYYNTAYLPINSNDLFDNTGQPFEVAKVLTNSQLDEDKY

HNYSPPYYTAANLVLYGIFFAIYPMSFFYYVANEWSVCKQSVKDIWLSIRYLNRSNYEGR

DDPFSRQMAQYKEVPDWWYYAILIVMFGLTVAFVEHWEVQTPVWSIVLILCVTLVFLFPF

TIVMSATGAQLTLNVILELIMGYTIAGRPIGINVAKAFAVQIQAQAQNLSSDQKIGHYMA

LRPRSMLRVQLWATMVNGLVTIGVIQFQLTKIEHFCDIKYQKEHEKFTCPNERTFFTASV

IWGLFGPKRMFDNQYPVLRWAFLMGFGVFLLFWGVQTVLPWALVKKYPQKERKIRKWQDR

LMMVNPVLVAFGGLSWAPYNLSYMTGGLYIAVLFSYYLRKYYTAWWKKYTYVINAALTTG

TALSGIIIFFAVQYTNKDLSWWGNNVPFAGQDGAGPPLMDIPADPGYFGPPPSHYP

>tr|Q6C1A1|Q6C1A1_YARLI YALI0F18040p OS=Yarrowia lipolytica (strain CLIB 122 / E 150) GN=YALI0F18040g PE=4 SV=1

MAQIRGKLRKALVSLAALSVFVLCVLAIPRSHSISGANRLLNKRDVLPEDECSNVRHIPR

ADQCEFVKLHCSEDENTFIDYMQVYYCSPYGQRPFLLIMLLLWLLTLFMTLGIAASDFLC

PNLGTISSLLGMSESLAGVTFLAFGNGSPDLFSTYSSMKIGSGSLAIGELIGAASFISAV

VVGAMALARPFKVARKSFVRDIGFFTAAVLCTMAFISDGKLRRSECILMLIIYMIYVATV

VSWHYHYTKKRQAYLSEIRAREFFLDAGQEANVIEDEEVGESSNSVLQDGFDREGFLSLT

NSDDASRTSRSPVKSPQTPTANNLSPHPNPHAPHGRSLSPHGRLPDRGRSPLAVERLRTE

HAQSRSYSPLDNRSSPIITPVHSHPSSHSHRNVPRIVTHNLDLDEDEEEDRYTGLTSSMR

LPTPLHTTHRVHDSAPIRPSLYGALDFRDRINNLNDGEDHHAHNHSDLIHSPLTRRLDTE

LSPVPSSIEPMWSNVKEVTESAWVVALFPTLTDFWSKSFMGMVSSVITALPVFLLSITIP

VVESVVEEPQVPNASEPVSAYSDDASFVSNNTATTNPLLAAESENKVLYNMARRNKILVI

VQAMLAPPVINILMLDEVPGVWGTIFCIICSMTLMWLAATNYDRMVQSGQYRFLAFFGFL

TSIAWVSHIADVVVGVLKALGAILGISDPVLGLTVFAFGNSLGDLIANTTIAKMGFPMMA

LSACFGGPLLNVLVGVGVSGLIVTSSPGNAVKNGSYHIEISGTLIISAATLFLTLVITMV

MVPLARWHMTKTIGVTIISIWTISTIVNVIFI

>tr|Q6C1F0|Q6C1F0_YARLI YALI0F16896p OS=Yarrowia lipolytica (strain CLIB 122 / E 150) GN=YALI0F16896g PE=4 SV=1

MEMLEKRGGDVWQYTYDTGDVAYITVSMCMILVMIPGLGFLYSGLARRKSALSMIWVCFM

GAIVAQIQWYFWGYSLAFSPSSNRFIGNLDNFGLKNILKHEDGSRKYPELLFAGFQGMFC

SVTVAIVVGGVAERGRLLPAMIFAFCWATIVYCPIAFWIWGPNGWAGAHWDVLDFAGGGP

VEIASGIGGLAYSFALGRRKEQLLLNFRPHNVSLVTMGTALLWFGWLGFNGGSALGSNLK

AVYAVWNSNIAACFGALTWCLLDFRLEKKWSTVAICSGIISGLVAATPCSGIIPLWASVP

LGIVAAAICNFSTQIKYILKIDDSMDTFAEHGVAGIVGLIFNAFFGADYVIGLDGSTPHG

GGWVSNNWKQIYKQICFVLAASGYTFVMTVVLCYAINFIPGCKLRASEEAELRGMDEDQI

GEFAYDYVEVRRDWLAWTPPTKSHMHDEPEHSTDADAIEANADVLAGSSPNDQNSSMEQP

PTSSQATSADDEKNGIHP

>tr|Q6C1Z4|Q6C1Z4_YARLI YALI0F12133p OS=Yarrowia lipolytica (strain CLIB 122 / E 150) GN=YALI0F12133g PE=4 SV=2

MVNVLSIASRLAMMTLSYVVGAIQPPLQSIFSTHPDLMGILLVLVIFYVSLLVLKQSLRS

FWGLITTFVRMFVLLAVVGGAGWVYVRGVGGTIDDIQALRAGKMDQFMDGDYQTYKQGFD

YSRKLYEEAAKMKGSGGGQYDWKEMMDRVDTAEVAQLWERFTRGF

>tr|Q6C243|Q6C243_YARLI YALI0F11033p OS=Yarrowia lipolytica (strain CLIB 122 / E 150) GN=YALI0F11033g PE=4 SV=2

MEHLRKLDSPNVPWRQIIVGTAIGDYLLESYLNYRQYQVYKRTEVPASLQGIVSQEKLTE

SNDYSMAKMRFSFVHSTYSLVNFLATIHFNVIPKIFHVTKMGFTKRIAPKLAGATFFGAK

TLHKLALSTPVHTAFAFNVFGLVSSLLELPFSYYKNFVLEKKYGFNKMTPKTFVLDFFKE

QALSFTIQGLYIGIFEKILIKFGLSFVPYFTGFVVVLQIVLMYAVPTLIMPMFNKFEKLE

DGELKDRSEALAKKLDFPLSDLYVIDGSTRSAHSNAFFTGLPWKKQIVLYDTLIEQCSTD

EIEAILGHELGHWKMNHILQTLLAGNANILTLTLGFLAFAHNDSFYTSLGFFSNDRPAAY

LFNTLYLQVISPIQYGVTFLMNGMSRKNEFEADQFSKDLGYGDALAKSLITIHQENLSNY

DGDWLYNSYHRSHPLLLERLEAIGYKPKAQ

>tr|Q6C273|Q6C273_YARLI YALI0F10197p OS=Yarrowia lipolytica (strain CLIB 122 / E 150) GN=YALI0F10197g PE=4 SV=1

MVQKDEKIAILGAGITGLYIAYILTEKGYSNIHMTAQYLPGDTSIDYTSPWAGGNFCAIS

GSDPATLVYDKETYLGLAPIFDTWGAAKGFERLPITEFWDFEPPKQKIESLKTYLKDFQI

LPSSELPEGAKFGVRYLTYNFNCPVVLVSFKKYLESKGVTFERKTVQNLSDAFGDAKVLF

NATGLGARTLGEVEDKRCFPTRGQVVVVRVPSVKENRVRWGTDYATYIIPRPGSGGHVVC

GGFLQKDRYTASTFGEEAEDIIRRTTQLMPELKGAEIVRDAAGLRPSREGGVRIERQVDL

QGRTIIHDYGAGGAGYQSGYGMAKHAISLMEWDSKL

>tr|Q6C2A1|Q6C2A1_YARLI YALI0F09515p OS=Yarrowia lipolytica (strain CLIB 122 / E 150) GN=YALI0F09515g PE=4 SV=2

MIPRQPPPAGPLGTDFDYSVAGVAGVLVALGAPWPMGQIAEVLSDKQLGLAFFTMFLHVM

YVGGLVTEACFPANFVTTAIKIPKPIFLATKVVYIAFASWEFSGHGDMSLYLVGILLATL

VLTELGAALKANKHTAQDKKTQ

>tr|Q6C2A7|Q6C2A7_YARLI YALI0F09405p OS=Yarrowia lipolytica (strain CLIB 122 / E 150) GN=YALI0F09405g PE=3 SV=1

MATKILASTLFLFGLVVAGYLLFQGEGESFNVGEFLSTTSPLMWANLGIGMCITLSVIGA

AWGIFITGSTIIGAGVKAPRITTKNLISIIFCEVVAIYGLIMAIVFSAKLASVEDVYTKS

NMYTGFSLFWAGLTVGLGNLVCGICVGITGSTAAVADAADSALFVKILVIEIFGSVLGLF

GLIVGLLMAGKASEIS

>tr|Q6C2D8|Q6C2D8_YARLI YALI0F08723p OS=Yarrowia lipolytica (strain CLIB 122 / E 150) GN=YALI0F08723g PE=4 SV=1

MRPLIRLAMAVGAVTLFLVFISVGSHPSNPVPQSASTVNVVKSSINAYIPNRLKAKFAFK

VHDSDSVLQQLRSIAGAKKNRRFAITSSVQTASFTGLAMNLGYSIQKYNDLRALDADLVL

LVRAQVNGDDGVTAQNITNLEKVGWRVKEAEGIDFDGVDINKIRPWHKHNLNKLHLWSWT

QYEKVIFIDADVLCKGALKELLLMPGDTLAAAPDVWWDKLTDNKFNSGVISFKPNMEEFR

ALVKAVSDPKMHAPNDADQALLNNYYQFRYFGLPYKYNFNLVMYHYHRESWDQLWDEAVL

IHFTTRKPKPGKKNWCRTTCAETKVLEWYTQVYQEMMAYHGFTEKDIPVLG

>tr|Q6C2K7|Q6C2K7_YARLI YALI0F07062p OS=Yarrowia lipolytica (strain CLIB 122 / E 150) GN=YALI0F07062g PE=4 SV=1

MDLEKQHYDESSGQSEANDIMNDSGDEQKQAAEPEVLQRDEHDAVASLGPVDTRGSGMSQ

EEALSQILSHPRHRETNAPLPPMGNGKEYPPLLPSRDAYLVGFDGPNDPLHPFNWPLRQK

LMISCVLGFSTFVVTWGSAVFAPASPHIAKQYGVIQEVTTLGISLYVFGFATGPVVWAPL

SELFGRKLPICISAFMIMCFTFGVAAAKDLQTIMLCRFFASCTGSAPLVVVAAAFADIYN

NKQRGIAVLMFASVVFIGPIIAPVVGGFISESYLGWRWTMYITGIMAALGAILAVAFYKE

SYHPMLLTQRAKELRERTGNWGIYASQEVVELDLHAIISQNLVRPIKMLFTEPILLLITI

YTSFIYGILYLFLEAYPIVFIEGYKMSPTIGELPYLGVLVGFIFSCLFGCFIFEPRYIAA

VERNNGKPVPEARLIFMMSGAITFPIGIFWFTWSGNYHEHVHWAVPTVSGLFSGYGLMSI

FVAALNYIVDSYLIVAASALAANTFMRSIFAGVFPLFAGYMFHAMHVNWAGLLIGLVACA

LAPVPFLFYIYGQRIRRSSKYAFDLS

>tr|Q6C2U2|Q6C2U2_YARLI YALI0F05148p OS=Yarrowia lipolytica (strain CLIB 122 / E 150) GN=YALI0F05148g PE=4 SV=1

MSDSIDRVFVRAINTIRTLSSRPGYSSRPRPPIESRIQLYGMYKQATEGDVMGVMARPVG

DSEIDVAGQKKWDAWKQQQGLSKTEAKRRYVSFLISLMNTYASDTHEGRELLAELEYMWD

QIKDVASDSEGAEEELYEDAVEYPSRSALGRAEVGTSPSHVSHGSGGGAHGSGVGGSSRA

QQGSNLTHGSGNSRSHHTAIAPHPQSNLVQSQSQHQHQQHTPPIPSDRFKSDMAWALRTI

NEEMADMAKQYRPADPSEPRRNWRRRVLLLLRAIGVHVGIDMVLFAILAAILKARRGGKG

PAVSRGVVSPVHTFPTRVLSLASLGDHISYHIIQMADGVYGYAVAWVSMS

>tr|Q6C358|Q6C358_YARLI YALI0F02409p OS=Yarrowia lipolytica (strain CLIB 122 / E 150) GN=YALI0F02409g PE=4 SV=1

MVSVSRMMPVPSARSEPIPYHSLMTLVMKYRFGNMYRLLFTLCYLIGIVIEVLRRVLASS

DDSSWWWCLLPLRASWLLLGLLPVVIMRKADLHIRELPYLSLAAEICDRIFSTTFVVTFA

AYMLSASMFFGYYTKAMSTENSAFTTRATPLTQPMLNENALYLMVFPIALSLAQSVLHIA

KDIDKLIVQPVTEEPARPMERLKHQMTDIVKNAGTLSAGFSLVVYPIMFYPSFRTKIWNI

TMSLSGYWYGLNKAQTPEVYPGLRFSLVMDTFIASAILLLLWNLVNTTFSIYMSLGPLRK

GEGISESSPDRNGTLVSGLRDSKHVYPQSVAFQELLYVALFSPQGRVQMFEDLSRDQSMW

VEVYAECKKQIVAVTEAANSSIGKVKSKPKPNPFDYKGKQQADLVSTARPEAPQIVIRRD

NIFKNPKPDFPKVVQVFQDKEATTSNQFSESVSELFKTFTELLGGYQTQMIQLLRLPIGA

PFRQTIQRRCKRIVPEPLLTCNAIMALSFLVFHSIDEDRYGSVQRTIPEILDTLCQSMVA

LDELLRKPPVSWTDVKFNPDDIDEEMSDIVFIRSSAQTAFSRIVFRFEQWIEGMDLTPLV

QREIRKLYS

>tr|Q6C368|Q6C368_YARLI YALI0F02189p OS=Yarrowia lipolytica (strain CLIB 122 / E 150) GN=YALI0F02189g PE=4 SV=1

MSIRGTAKYHQIPVQSDAPQFAAPQGQQFSSYSQNRDFNLKSTITEGDFADKVKAVTQKI

EVFLDKYSGPVKPHLPTLGRFLIVVTFLEDALRILTQWSDQVYYITNFKHIPKFITVIFL

LLNVVAMIAGSFMVTAKKRIEVGCGLLVGVIVTQALAYGLIFDFGFILRNLSVIGGLFIA

LNDAFVKDKSKRGLPGLPSIDDKDRSKYVLLAGRILLVVMFTSFILNMTWTMSRVLVSIV

GIAACSMVVVGFKARVSAFLLCIILFIFNITANSYWAFPASSPVRDYLKYEHFQTLSIIG

GLLLVVNTGAGKISIDEKKKVY

>tr|Q6C220|Q6C220_YARLI YALI0F11561p OS=Yarrowia lipolytica (strain CLIB 122 / E 150) GN=YALI0F11561g PE=4 SV=1

MSSFWGNSQAGVPPETANNSSWWSNIQNGIRLPTTETETMAENAGVDTQIAEPEWFKLSW

WDRMIVFGVCLAGAVVCFAICFLIMPVLVLKPRKFAVLWTLGSLLFVISFGVLQGPVSYV

KHLVSKERLPFTVAFFGSIFATLYFSLGMKNTLLTIIACVVQLVAAIWYAVSYFPFGHQT

MRFASRVGARQVTGWINS

>tr|Q6C226|Q6C226_YARLI YALI0F11407p OS=Yarrowia lipolytica (strain CLIB 122 / E 150) GN=YALI0F11407g PE=4 SV=1

MRLVRQRGVLALAFVATVFVCWLLYSVRQQQAQDVFIQGSINTPVVYNARVMPYIIDNDG

YCGVPEWLKRGVYVAEKPCDDGVVPVPYGESSWWSVMDAPSGTGHAGVQDKWRTSTVDNN

TSLFMHALSECDKGSHMACLIMNVPKGPFHTVKDPLAALTGETISPYTNEDTIHVCGKTY

KDCVVVPGVIIEHLFFVDFNESYSDAQFETAVREAGYFWRQMEW

>tr|Q6C9P8|Q6C9P8_YARLI YALI0D09317p OS=Yarrowia lipolytica (strain CLIB 122 / E 150) GN=YALI0D09317g PE=4 SV=1

MPSTIALGCTVGIVSSLCQSVGLTLQRQSHILEDHKPGTDSYRPPHRRARWRVGLLLFLI

ANVLGSSVQITTLPLVVLSPLQAVGLVFNSICATVILNEPFTVFSLVGTALVSLGALLIA

AFGAIEEPNHSLNELLVLMKRKPFVLWMAFTAVLVFGIMGAIKAVSRSQKHLGSKRRASI

GSGYSTITEEDQNETRDSPTTVLIGLLYGGISGILSAHSLLFAKSGVELLLRAMTSGLGD

LQRWQSWAIVVCFLTLAVTQLMFLNKGLRLCSTSILYPLVFCVYNIITIVNGLVYFQQLE

RLSSVQIILVILGALLVFAGVVALSWRFQEKSSLSHVYEDEEIEPLMPAALIALENTMEE

AENESERDTSPSPLYSQNTNNNSGRRKRWSLSISTTPNNASSASIAGPPSPSPLRLHRRS

MSSLSYLPQSPSNRRKISVQQQTLMKELGL

>tr|F2Z624|F2Z624_YARLI YALI0A09449p OS=Yarrowia lipolytica (strain CLIB 122 / E 150) GN=YALI0A09449g PE=4 SV=1

MLNYSKVIFIGDSLTELSFDPTQFGLGSALSHHFRRRADVYNRGLSGYNSVWLEDQIDRI

CLEFEDSASQVVLIILWLGTNDSVIPGNPHHVLESEFLTNLKKYTAKLLSTFPEANLLLL

TPAPINMTQLQVSTLSDSGKARTPELALQYAQIVVRFFREQCQPDPHVKCIDLYNLLGSG

KDDDFYVDGVHLNSAGYREVWNAVHQELVGWVELPLIEPHWTVKAGEQKLGEDHTG

>tr|Q6C3R2|Q6C3R2_YARLI YALI0E32769p OS=Yarrowia lipolytica (strain CLIB 122 / E 150) GN=YALI0E32769g PE=4 SV=1

MTIDSQYYKSRDKNDTAPKIAGIRYAPLSTPLLNRCETFSLVWHIFSIPTFLTIFMLCCA

IPLLWPFVIAYVVYAVKDDSPSNGGVVKRYSPISRNFFIWKLFGRYFPITLHKTVDLEPT

HTYYPLDVQEYHLIAERYWPQNKYLRAIISTIEYFLPAFMKRSLSINEQEQPAERDPLLS

PVSPSSPGSQPDKWINHDSRYSRGESSGSNGHASGSELNGNGNNGTTNRRPLSSASAGST

ASDSTLLNGSLNSYANQIIGENDPQLSPTKLKPTGRKYIFGYHPHGIIGMGAFGGIATEG

AGWSKLFPGIPVSLMTLTNNFRVPLYREYLMSLGVASVSKKSCKALLKRNQSICIVVGGA

QESLLARPGVMDLVLLKRKGFVRLGMEVGNVALVPIMAFGENDLYDQVSNDKSSKLYRFQ

QFVKNFLGFTLPLMHARGVFNYDVGLVPYRRPVNIVVGSPIDLPYLPHPTDEEVSEYHDR

YIAELQRIYNEHKDEYFIDWTEEGKGAPEFRMIE

>tr|Q6C3Z4|Q6C3Z4_YARLI YALI0E31064p OS=Yarrowia lipolytica (strain CLIB 122 / E 150) GN=YALI0E31064g PE=4 SV=1

MKFNHSLQFNAVPEWTSEYIAYSQLKKLIYSLERTALGVPNYEDEDEENSTLIREADSNP

EDIFKRALDDELKKIDSFYMEREQDIYLDLDRLIEDEAVFQAEHAGHEQGYCPQDTDNEQ

PFRLARSRSSLSVDSTDGEDLHNQLSPVRSRARQSFGKAVAPSTIHSDDSDEDVVAIRRK

RTRSQSGARRRPSWFHHGGEEDVFSNLYDMRITLKKRATAVYVSLCELRSYSQLNKTGFS

KALKKFDKTLDRHIRESYLENMCSKTYIFSRATEQNLDHRVDEAVDVYARLATNGDVKIA

RQELRLHLREHIVWERNTVWRDMIGMERKAQAANLNIIGGNRNVRLQGEEPTSLLLEKVD

YVMMWGFRLPRAIVGRRAILCLIGLGILLTFIHVITPFDSDAQNNCLAIIVLASFFWATE

ALPLFVTSMMLPFLVVLLRVNLGTTVDKHGNTVTYRLTAPAAADVVFSQMWSSVIMLLLG

GFTLAAALSKYDIAKILATAILSKAGTSPRVVLLTLMFVAWFLSMWISNVASPVLCYSIS

QPLLRTLPPDSTFAKAVILGIALAANVGGMGSPIASPQNLIALENMDPIPTWMQWFAVAI

PVSIVSILMVWFFLMLTFPPGDLKLTPIRRMREPYTGKQYFVVFGSLVTIVLWCMSQKLE

PVLGNMGVIALIPIVLFFGTGLLTTEDFNNFLWTIIALAMGGVVMGKAVASSGLLATIAS

AISIKLSSLDLFGVIVVFGMLVLVVATFVSHTVAALVILPLVKTIGESLPDPQPRILVMV

SALLCSAAMGLPTSGFPNVTAICMTDEMGRRYLTVSNFITRGVPSSILVYIAIITIGYFF

MVVSGY

>tr|Q6CAF6|Q6CAF6_YARLI YALI0D03179p OS=Yarrowia lipolytica (strain CLIB 122 / E 150) GN=YALI0D03179g PE=4 SV=2

MCQDWNMNKMSYQRPNPNPFEGERLSDSDHEGYNDTGHPNLSNPHLNQDTSYNPSYNYTP

NTSAPYPVPTVNVSGVNRTPSGNHYEDPSEYGYHGNTSNLYQMDSLTPNHTINDFHEHEG

DITRASFQNDDDRVPLRDETAYHGYQGPFVSENEHHNAQQAPLPYPYPHQQHHEIHHAES

TTALPQGEQIGGEYIDLAEAQREFIGDVSNGTPEPSYNTIPEDGTLNEYHDGVYAGQPGY

PSSDFDPNFPNYPGSDGRTTSLLSTGSDGWQKRQQVNLKRFPTRKVKLAKGAVFSTEYPV

PSAIKNSILPEYIDAETGRTEFTHMRYTAATCDPDDFTLENGYKLRAHTYGRQTELLIAI

TYYNEDKVLTARTMYGIMQNVRDICRLKKSEFWKSGGTEPWQKIVVSLVFDGIDPCDKEV

LDMLATIGLYQDGVMKKDIDGKETVAHIFEYTTQLCVTPKQQLVKPTSNDPMSLPPIQMI

FCLKQKNSKKINSHRWLFNAFGKILQPEIFILLDAGTKPGPKSILYLWESFYNNKNVGGA

CGEIHAMLGKGHRKLLNPLVAAQNFEYKISNILDKPLESSFGYVSVLPGAFSAYRYSAVE

GRPLEQYFHGDHTLGSRLGKKGIDGMSIFRKNMFLAEDRILCFEVTFKAGCKWHLAYIKQ

SKAETDVPEAIDEFIGQRRRWLNGSFAATLYSVAHFPRIYRSRHNPLRMFFFHIQEIYNL

VNLVLSWFSLASYFLTTTVIMDLAANPRKYGGDENSHGFPFGDTASPIISLILKYLYCAC

LVISFILALGNRPKGSKTTYYIMVCIFGCIQIYVLVISFWLAANAFIDNKITSASDFFSD

FFDSITGLIVVALASTYGLYFISGILYLDPWHCIHSFPQYMFFMQSFVNILNVFAFCNWH

DVSWGTKGSDKADALPSVQSKKNKEDGEDEAVIEEADKPQKDIDMQFQQVVKRALTPYKP

PPPEKGLSRDDENRNFRTNLVIVWLITNGALAIGVTSQSIHAFGIDASQTTKTAYFFGAL

LWSTAVVALIRFIGCLYFVIKSTFKRLIAKR

>tr|Q6CC96|Q6CC96_YARLI YALI0C11297p OS=Yarrowia lipolytica (strain CLIB 122 / E 150) GN=YALI0C11297g PE=4 SV=1

MTYSTQSSSTLVAIRRDECFFPISSHSISLSSVLSYLIDWIFYISLTTLALVYAKIVSPL

FAEFYLYNTSLWYSHIPTDLTIVPTFLLIIYSILIPIGQFALTIGFTTSHRWHRRLWDLH

AILLTLMAAHALQTVIVSLLKNLVGAPRPDMLARCRPMSWMRPSFGTLSNVGICTQTDIG

HLEEGFRSFPSAHSATAFTSAMVQVLFWIARTRMLDCSGWSWKLLLSLVPLLSASAVAFS

RISDNRHHVFDVIIGMLIGLIAGYLAFIHYFPFPTFANVCTGGRAYSPRCGILGSVGCWS

LGDETGCLRTKLFKTPKCSVKATFTMGCGTELCVCKKPSCGACSVGGEGAKCSLRCSIRN

CTGNACTSRPVSRRSTRRTPSRRSRRSHQTYPGRSSETACLPSCSPTCTGNCHDTTCSSC

DSSATATEPSHTRSAHRCTNPVCTIAGCIGACLRRVTSRPRCTTVGCTVANCIGISCLVG

RVRTCRIPWCRNERCMRLAREHCFDEEASIIDVGGGRSRASRHRRVSRHAITP

>tr|Q6CC97|Q6CC97_YARLI YALI0C11275p OS=Yarrowia lipolytica (strain CLIB 122 / E 150) GN=YALI0C11275g PE=4 SV=2

MMYYAAFLLFFFFFVAGAVSGDGTFLLGSIDHAKYSGKISYDTAPYPSTGAVINLDTITV

NVTDVSFDESALLDSGLIVIAISDPQFKSRARFFLTAIQMSVSSSTLGTIQSFLTNLRSF

CPSLTSLEPPPTLSACLLFRTWQYLGQHRPAVSACKLVPEKKTPTQPSIWITRKLDWS

>tr|Q6CC98|Q6CC98_YARLI YALI0C11253p OS=Yarrowia lipolytica (strain CLIB 122 / E 150) GN=YALI0C11253g PE=4 SV=1

MKINVIEAAKHAKELWLKKDPRDEDELQSDVQVEVVEVEEHLLEWMGAKLGIPPSDMDGG

YPREVHFMAEKLAEMSLEKALELVKTNYDYHDHDNNFRHEYRMEIKGLLDIFKDSPVESE

KEDPENILLIRYWATIFDWWSPYPEVRSVTDPFDETDTTVETWRVWVLGTIWVAISAFVN

QFFSVRLPAITLDASVCQLLLYPCGRLLQYALPDWGFRFRGKRYTLNPGIWSQKEQLLAT

VMVTCANGTPYITSNVITQALPIFYNQKWASDYGYQFVFMLTTQMIGFGMSGLLKRVAVY

PVKAMWPSLLPTLAVNKALLAPNRKESINGWKISRYNFFMVIFAVSFLYFWVPNYLMNFM

QTFNWMTWISPRNADLAIVTGSIEGLGFNPIPTFDWNQATALLAPITLPLYTSVTGFCGA

LFGGLVILALYYSNNSWTGYISINSNRLFDNKGEVFNVTRILTDYRFDRDKYLAYSTPYY

SAGNLVAYSANFSIFTFSFVYTILTDWRAMKDAIVDTAKAVRYIHRSNYYGKVDPYSRYM

RKHKEVPDWWFYLVMIIMFVLSVVLVEVWPVDTPVWCIVFVIGLVAVFIIPFTVFLSYTA

TELTLNVLSELMIGYALPGRFMALNLVKALSVQIAQQAQAYTKDQKLTHYAHLPPRTIFW

LQLWATLVNGVVAMGVIQFQLGQEKLCEPDNKMKFTCPEQTTFFTASVAWGVIGPKKMFN

KYPVMKWMFLFGACAGLFFFFVQVTMPKLMVRWFPKHADRIDFYRRKFLYFNPLIFVMGI

VDGWSPYNLTYKVGGLYLALVFNGYIKGRYLAWWRKYAYVMEAAIVTGIALSAIIIFFSV

QYNPKDLDWWGNSVVNAGYDSEGIPPNVQVPEDPGYFGPPPSEW

>tr|Q6CGR0|Q6CGR0_YARLI YALI0A17171p OS=Yarrowia lipolytica (strain CLIB 122 / E 150) GN=YALI0A17171g PE=3 SV=1

MFFQFFFNFFNFFSIFSIFFFFNFFIFLLPFISQFFVIAALSQQLTQTGYLRHIEKNDLW

SVADNQQVAKLQARLEERLDFRYNQLLERKIAKLKKSKKPTTTKTSIYPGIHYDNDDPAD

PDHHDDHIRQQAIAALNKSSRPLVMALNDTFLVQFWFAALMTILRELCAVASALVSKELI

NYIERRYYDRNYPMGTGIGSSIGMFLLAVGVTFFFNHSFYAAQMVGAKSRAALIGTIFKK

STKLSISARKIHPPGKITNLAALDAHRVDFACQFVHQITAFPITTACSIAVLCVNLHESA

LVGVATMMLFIVSVAYITSYTMKMRRRIIKWTDKRVTLIRDALQNIRIIKYYGWEIPTLQ

KIQVARREEANGYIRMGSIRGISFGMFESLPTIAGALSFITYSSIGNETDPAKIFSSLTL

FNLLLPALIQLPMAMQYVGDARMSLRRIHEFLNAEEGVSNIAINTEQPESILVQDGCFEW

PQDEPMEFFSEEKQSHKPTEKSETRSIKSIKTLRSGKSVLSGKSSAKSSRKSSISSKFSF

FESNDIQSSDDEPEADVSLTLNNLNLALKPGSLTVVVGGIGSGKTSLLSALTNLMLMTDG

SLSFSSSNWIGCLEPWIQNASIRENIVFGREFDADKYRHVLKVCQLASDLDHMEHGDKTM

IGERGITVSGGQKARINLARAVYGKPDILIMDDVLSAVDARVGQAMIDQCIDGYLKDSTR

VLATHQVGIVERADHVIFMEKPDAQSYDNHAKIHQGTVAELSDRLPQFRALIEAGAVRGE

IEMEDDNEVKHELVELVEVPEKIEFAPATKRTSRVSMESSISANMMAEEERVRDSVGFGV

YKRYILFSNKNKALSIALIVLCAFLMLLVAFLTIFASVWLSFWTADRFLTTKGFYMGIYI

MLGMVLLLLLTCFIMASQVLGTRSIENMHNLAIETIIRAPVAFFDTTPMGRILNRFTRDT

DSLDNEVGMRYRMLFFSLTEFVGSFAMTIVFVPYTALVLAPIIVVFVVCLSYYRQTAREV

KRIDSLERSAMLSVFSESISGMSVIVMYGAQSKIVSRLHGTLDIMNSAYFIVAANQRWLS

LRLDAMGSLVVLICTILCAVGVFHLTPSNMGLIVSQAASIPENLSMMAQGFSELENCMNA

AERILHYHTITSEGSSKTVTASPSTPSVGKIEFDNVSLKYRPELSLVLKNLTVTFEPGHK

IGICGRTGAGKSTILQSLFRIVELETGKISIDGVDIASMSLEQLRKGLSIIPQESVLFGG

TVRSNLDPLGEHTDAEMWSCLQRSGLTVALDTKVDSDGTNFSLGERQLLTLARALLRNSQ

ILVLDEATSNVDYQTDALVQQTISQEFSHCTILCIAHRLRTIVNYDRILVLEDGEMLQYG

SPQELFRQDGGMFRHLCDASGIKM

>tr|Q6CGY6|Q6CGY6_YARLI YALI0A14883p OS=Yarrowia lipolytica (strain CLIB 122 / E 150) GN=YALI0A14883g PE=4 SV=1

MWAPIKHALYAPKPEPGSSPTVVENPSTFQKIFNPPVKKDENAIDENAAPNGSQEMAKIT

SLWNRHDLWMAWGSMLLIAVALALQGRTFKVYSTFATSEFEELSLLSSLAVTQAVINVAA

TTVFAKLADVLGRFESFLLSVVFFTIGFIMLAVSPNIGTYFAAHIFYVSGSQGITFLQQV

FAADTSSLRNRLFFVVLPNVCYIFVPWCAAPITNAIIKHSTWRWGYGMWCIIVPIVSIPV

LTILFRLKMKAKSLNMAGGKSLQVRDAKETLRQFDFIGLTLLTAGLALLFLAVTLVKTSK

SWTEPHVLVMVIIGPILLVLFPIWEKFPKYPFLPMKNMKNRTLITGCVFAGIHAMATSLY

NPYYMPWLLVCKGLSVTAATNASATLSVSYIVTTIIAAFIIRYTRRVKPCIVAGSCIYTL

GLGLTYHYRQPDVPLAKFIVTQAVEGVGNGLLQSPALVLTQSSMPKHLVISATAIYYTIN

SIGRVVGDAISGSLYRQQYPKRLAEFAPFMDEKTVQRMVNDVNSPLKFAWGSEERVAVIE

AFNHVYRKMLYGPMIIAAVGILIALTFPNINMSEVDNGFVVEETEEDMPATAVVNEKKQQ

QPSETSESETEKKLPEVSSSHTVSSGPRSADLSVEEHERRV

>tr|Q6CH05|Q6CH05_YARLI YALI0A14454p OS=Yarrowia lipolytica (strain CLIB 122 / E 150) GN=YALI0A14454g PE=4 SV=1

MQSNDHSKSFLNLTTSTLFGVFGSQTNLSELNGEDSETVELGRLDAPEFAALRRDAQGPP

KRRRSSFSYTKRAPSKRDSKPIKKPSKQTGFSFGNSPLFKTALLFVLGCGYGCVVHFLYE

KQVGTRRDTDKLTLPMWGVHCVLLGLGLPLLDKYKPSTSAPAKGLQRIRAKNGGSSPNKK

WGSLVRTVGAFLGVAYGVKNLNWHSTTEVAVIWALLNPFLWYLLDTTSNGFGLAAVSSLI

GAAVMSAIGVIPVGLGEWAGLVWLASNLFCGAICFGNLGRIIDRK

>tr|Q6CHB8|Q6CHB8_YARLI YALI0A10439p OS=Yarrowia lipolytica (strain CLIB 122 / E 150) GN=YALI0A10439g PE=4 SV=2

MRSNRYNTTMIILLLFLSVALSSPIMLDRFGDSMNSMIRGNNEAYESGETCGVSQEFEQR

LVRYMWFNNVAPCVPEKLQHPFKCIARGCKELGKHTELVDIFTHSDNLFDRTISGFVALD

HKHKEIILALRGTQDVNDWVTDLHLRLVELHPEHLGVSNFNCRNCQIDLGFLKGYLHSFP

AVDSIVQRLTEKYPNYQLVITGHSLGGTAATLFGLNYRLNGYSPLVFSTGAPALGNKQFA

NFADRVFWGSQNPNTLKVKERDIKFCRMTHLGDFVPRFPFWNGYQQMSGEVFINDVRGIY

PPRETLQRCNGQQNRQCSFGDQYRKLEMNFKPHSAYLVPGSKCSLSGGRELTFGEVHSAA

NDTNKDSDTDIEPDIEPILIPVVQID

>tr|Q6CI72|Q6CI72_YARLI YALI0A01023p OS=Yarrowia lipolytica (strain CLIB 122 / E 150) GN=YALI0A01023g PE=3 SV=1

MSEKNGVSSNSTTARTVTNSTALEVPPPYVQTIEQVTKQENANIHQGLSDSEVKDRLAKV

GPNQLDEGEGISLIKVIVGQVANAMILVLIISMCISFGIRDWIAGGVIAGVLGINVLVGV

IQQYNAEKTMDSLRSLSSPTARVIRNGEDTTVPSGDIVPGDLVVVKVGDTIPADFRIIEQ

VNFETDEALLTGESLPIAKDAEIVFDDINLPVGDRINMAFSSSIVSKGRAKGIVTSTGMN

TEIGVIAQSLKGQDRGFRPVKRDEFGKARKRDYMGAFFGTCGDFIQTFLGTNVGTPLQRR

LAKLALYLFGIAVIFAIVVMAAQKFDVNREVAVYAIATALAMIPASLVVVLTITMAVGTK

VMVRRNVIVRKLDSLEALGAVNDICSDKTGTLTQGKMIARKVWIPSVGTFTVERSEEPFN

PEQGVTDLLPWDPKTEYAKRRETDDPRAFDDVMVDVSRRNTDEQSPLFRNWIDCASLANI

AEVFTDNEGAWKATGDPTEIAIQVFVSRLGKSREELMKQQGLVHVAEHPFDSTVKRMSAI

YDAPDQSRVIFTKGAVERVLACVTTWTDPVSGDTATFDDTCVAEIEKQMETLAKQGLRVL

AFATRPMDASEDPKARADVEKDLHFLGLVGIYDPPRAESAPAVAKCHEAGINVHMLTGDH

PGTAKAIAQEVGIIPVNLYHYPQEVVDVMVMTAMQFDALSDEEIDALPVLPLVIARCAPA

TKVRMIDALHRRNRFTAMTGDGVNDSPSLKKADVGIAMGIAGSDVAKDASDIVLSDDNFA

SILNAVEEGRRMADNIQKFVLHLLAGNVGQAFFLLLGLVFQDDTGFSVFPLSPVEVLWII

MITSAFPAMGLGVEAAQPDIMTKPPRDPKEGLFNWELITDMVIYGLWLAAICISSFVIVV

YADGKGQLGVDCNKEFSESCHFVYRARALAFVEMTWMLLVLAWEVIDMRRSVFAMHPDSE

TPYTQVFKDLWSNQFLFWSVILGFFTVFPVVYIPVINDKVFLHKGISWEWGVAVLGLILF

VIGVEMYKWAKRVFFRHYEKKQKKKAVISDFDDSDPFAKFAQTVSRSNTMEKNPIAIV

>tr|Q6CI74|Q6CI74_YARLI YALI0A00979p OS=Yarrowia lipolytica (strain CLIB 122 / E 150) GN=YALI0A00979g PE=4 SV=1

MISLLPDLDHLTGDEQAWFLTFASSVACILGCLILYVDSVIYGFRRLKGWITGEPAGETE

DLWNSKGFLIISLSLSGGILIFTALYRLFPQALVYLEHSELLTPQQSNFLVLVMFTSGVI

VCAGINYLVHAATSKSIVHCVHGDERKDSAVTATSSSSHTHTELPPPRAHTQTRTHSHVH

GGHHHVHHQDSPLTHATHTHVIPAGEMSESTPLLHVAPRKRSILDLAHETIKGAAPVGEC

RGYTCGDCCAPEDQVICDPDPFEEPFAYPGEEFRFQDDHAHVHHDREDIDDIDPLDDHHH

DHDHHDHDHDTDLEHQVHHHHVSTRRSHLYSIALQTGLAITLHKLPEGFITFATSHADKE

LGLNVFLSLVVHNFTEGFTIAFPFYASLRKKWQAVLLASILGGCSQPLGAVVAWLMFRHQ

NLHTDATDILYGSIMGMTCGFLCIIGLQMYGTAIGFGGTQKVCLTSAFAGMFIVGSCYAL

TA

>tr|Q9B6E2|Q9B6E2_YARLI Cytochrome c oxidase subunit 1 OS=Yarrowia lipolytica (strain CLIB 122 / E 150) GN=cox1 PE=3 SV=1

MSLKLNIQRWLFSTNAKDIAVLYFIFALFSAMIGTGLSAIIRLELANTGSPFLHGNTQAF

NVVITAHAILMIFFFVNNRILFKSHTPNNNIIINPDSVIISDNNNNNNNNNNNNHFKYTK

VIIEDPLNNRDILLRVAKGQKGIYIWESIDGKNKYVGHSINLYNRISSYYMPSILKTKAR

KVLSYLNENGFKDIKLTIYILNSEILIDDLVKLEQHFFDTLKPNLNMTLIASATYNNGTM

SENAKSKLRTERGIPIFIYDVNTLNLLYIFESKQDMYNKLHIHHITLNKCLDLSEFYLDN

FLLSLDYIEEFINEDILSLDEIINLITKSREIYKITRQPKSKKILAEFKGNPDNNKVFTS

LNELAKYLKGDRATIRKYLKGEGYYRGVWRFKYWQGEIK

>tr|Q6CBF8|Q6CBF8_YARLI YALI0C19239p OS=Yarrowia lipolytica (strain CLIB 122 / E 150) GN=YALI0C19239g PE=4 SV=1

MHSEYLYYYLLFFPIVIAMILRLPHDILVVICELVSPRDLCALRETCQQLSRDIPGSFIL

GCLLSVWDCCNIEYSRSNDDTCQAPHKLNNSRVEFSDQVKCPVYIDQPLPRDFYCLCKEV

DEIFPWEYHDNGISFDEKLLDLTEHPEMGQGKPKKGLTMGRYYGSTYAFGGAPVIQTRHS

SKMLAGVSVINLIYSGIPQTRGIVNLDGLSLPFRLQVNGKSVLLHARACDYKTSEVICVS

PGGKVHTQRCRPRKAAPAGIVHYNDTFFNIDFQTRYRLRVCKSLHSLDSDPQLNEPYKVY

QDEEYSQFCLVYKPTGIIIGLIDLDNQQTEVFSAPGTGFVARNYSSISCEEYLVMVGMSK

GSLGIWKFSINHLRKRFRFQHGDGFANALSLAVSPLASKPR

>tr|Q6CBU1|Q6CBU1_YARLI YALI0C15488p OS=Yarrowia lipolytica (strain CLIB 122 / E 150) GN=YALI0C15488g PE=4 SV=1

MDLDNLPAPDLSWKSIKHYLATRVTTLKPPKLSAEEKKHINPIPALRTLNKKQWLFVLCG

LAGWTWDSFDFFSVSLVASDIAKDLNVSVTDITWGITLVLMLRSVGAIIFGVASDRYGRK

WPFIFNCVLFIVLELGTGFVQTYKQFLGVRALFGIAMGGIYGNAAATALEDCPPEARGVI

SGLLQEGYALGYLLCVIFTRAIADTSPHGWRALFWFGSGPPVLIIIFRFFLPETDTYIQS

KQNAEALGVEKHFWLGIKTTFKTYWLMFIYLVVLMAGFNFMSHGSQDLYPTMLKVQLGFS

PDRSTVTNCVANLGAIAGGVIIGHFSSVLGRRLSIMISCILGGAMIYPWAFVTNSGINAG

VFFLQFFVQGAWGVIPIHLTELCPPALRSSLVGLAYQLGNLASSASSTIEAQIGTQFPIK

DDNGVDRPGVYNYSLVMCIFIACVFTFVFVVTFLGPENRMAEMVAHEHVEYTKEMSDDEE

KGVQETVEVVERVDTNATK

>tr|Q6CC66|Q6CC66_YARLI YALI0C12056p OS=Yarrowia lipolytica (strain CLIB 122 / E 150) GN=YALI0C12056g PE=4 SV=2

MLQRAVHLSLFLSALLLFIFVATHSRPLIIVSDRPTKYKLPATKTTTSIQSKPTAMFNGN

PFVPIATSDPMSQFERREHPLKPPVARDGPQQTNKFYANMLLGDRDLPAYVYPYSVWWSK

TDNFQGLAISHTRASQLVYGPDPDQNPSQFYFNPVGIMSLVLGAHEFSQGMDMVVDKMDH

LSVDLQIGRGPAGLTAPLVRGMGMVTAKYNGVGPIIGSQVGFANIERVQNVRSDIQKYKL

TLNNQVVWIMYVNAPDGALQFNQSSPQHFAATSAAQNAVIQIAVLPETPGSEQALDAAAG

KYATGGSVSGTIDNDGLRGIYSLEFPTEGQSQCGFPLMYALEHQSHVMVDEIRQRETPCR

LDTCAKGVAIGYNTDRFTMAEQTLPRDIGFLPKPLKRDLQSQLQDQNALRLIFDAASKEM

DQDINGQANLDSMYFSGKGVDKFATILLVLNDVLGDKDRARTLLDRCKQIFSMFATNKQQ

NPLVYDTTWKGVISVAGFNDPNADFGNTYYNDHHFHFSYFIHAAAIIAKVDAEIGDGQWL

NQNREWVDTLLRDAANPSHDDRHFPVSRSFDWYMGHSWAKGLFLSADGKDEESSSEDYHF

AYGMKLWGQVSGNQAMEARANLMLAVMRRAMNIYWFMKDDNTNQPAKFIKNKVPGITFEN

KVDHATYFGINPEFIIGIHMLPTTPISSYMRDEEFVRQEWDQRVSTFVDGVDSGWKGILQ

LDRALFDPNASWKFFTQGMQPQWLDPGMSLAWSLTMIAGMGGK

>tr|Q6CHZ5|Q6CHZ5_YARLI YALI0A03091p OS=Yarrowia lipolytica (strain CLIB 122 / E 150) GN=YALI0A03091g PE=4 SV=1

MNESTFPKTSIEYLEDTKHSIEHLEDDKPSVEHLEETKTDDLYVQHSVTSGQVSDVSISK

KKEGHLKLHRSIAVGCQFQVFLASHIVSRDVIGVTVTGGIEPFLKLTPSEYEWSQAALYF

TYAVFVIPMALLFAKSNPRVFAGVQMFIWGLCCTLLGICVNYGGLTADRVIMGAVMSVAL

PVILQVTYESHGRFEGQFVIACSWAVSWFMVPLFALIAIALGLITDGKPGWAWMFFITGI

CGCLQAPLVWRLLPDYFYHPRWIRKHGEDGYQLFKKITEKHYAGGNPIEPIETVIQGLGM

ALKDPIVWLTGAMGLFTYNGIFSCYGELMTVTLHVLKYSPALGQCIVWPILVWSCFYCLV

QNWFSTKYKTNYPFLLANYLFAIIGWALVLCTPSHKNFWIKYGGAWFIIPNMVAAMPTLA

CWLGSNVQGRNRRLMSFAVFTFITNWYGVIVLRTWVATAAPLFVKAAWVNMAFMIAGVVF

TAALVVVLKLKTSHGAFVYLL

>tr|Q6CI02|Q6CI02_YARLI Cytochrome b-c1 complex subunit Rieske, mitochondrial OS=Yarrowia lipolytica (strain CLIB 122 / E 150) GN=YALI0A02915g PE=3 SV=1

MSLLRTAAQAVKAPKAYTPLVAAKAFAQTRSVSSQPIGGKSTYKIPDFTPYLKKDRNTDA

NRLFSYFMIGSFGMLSAAGAKATVQDFLSNMSASADVLAMAKVEVKLGAIPLGKNVIIKW

RGKPIFIRHRTSEEIEEANEVNVATLRDPQTDDERVQKPEWLVMIGVCTHLGCVPIGEAG

DFGGWFCPCHGSHYDISGRIRRGPAPLNLEIPEYDFADAETLVIG

>tr|Q6CI11|Q6CI11_YARLI YALI0A02629p OS=Yarrowia lipolytica (strain CLIB 122 / E 150) GN=YALI0A02629g PE=4 SV=2

MPNIRNLQVRKGIAKGVVLLFYIAIFHIFVNTSSGSIEQYLSASPPHLYLKHLAEHVSWL

PTDSNLWLTVVFVTVVINSLVLLTWVITQVIFLVLCMEILTKRRWSGVMNLLAELF

>tr|Q6CI27|Q6CI27_YARLI YALI0A02288p OS=Yarrowia lipolytica (strain CLIB 122 / E 150) GN=YALI0A02288g PE=4 SV=1

MKLAQFAVWLAPAAAIYFSDCFHQRNNPLGLSWTPDSVFADYDKDDGRLHFSVTGTVSGA

VLSDLNETENKYTTLNSQSRSMKGLFVDNYTRFCDEVSKDTKPETCPIQPGQEASFAYTV

EVPEVARRISDIATIFKIISPVDNGDGLVVGCVEIQTSPVIDKGTIYAVIFSTLGVSIVV

FVATFGVQSFSTWSRLNTGSDSDLDTSSPHATMKASILAQFTGLVAYWQFAFFVACLNLN

YAGAYQAVASGFGWAALTFGKSFTSDVIESADPANGLYNFHTPGMPAMATIVGLHDNRDI

WPGFIIWFLVIFGATIIAMCVSQCLAGRTSFASLLTGLAKTTITVVYSLLALPLLALSFY

QLQAGGTLTVAKVLSGLIVGFWALGGIYFCWSVSQGRNGDLGCFTGCMKPSTSSAFRASI

VFVELAHVYLLGVCIGVLQSSGVAQVAFLGTLEFLIFAITLIVAPYVFSRLSALVALFRM

IISLLSIVYIRSLDTSLSLKIIMGIVIMVLHLVVCLSFLFFAGLLIFRPRFRSQSSPVSS

SSSFNINSKSVSPVNGDTLLGQYNDDLNSQSEKRAAIRENIRFQPEMYERPMSGLQDHLK

GHVQDVQSPQDLGLADMDFDFEDERPMHQRPVSGHKRSMSGNERPLSGSLTGSRPTSDLY

ERRQYRRRRSSASGHRKSLTIDTSALVKSHTNPSPALPNPPALKTVADRVVASPHTYYRP

PRRRSEDRTSTATPQTPEVPVLPSPHVDYTQREADIYKRSSTMCEDYDLGGVGSHDALHK

STSHLISKTRSADEVINKHERKLFQPKTWIKPEEDPLEPKGFEVVGRGK

>tr|Q6CI42|Q6CI42_YARLI YALI0A01958p OS=Yarrowia lipolytica (strain CLIB 122 / E 150) GN=YALI0A01958g PE=3 SV=1

MFWKNMKNEPRQVLNSTLWLSVIVFGLLGSARGLDEGMIAGTTSQASFEHQFNLKDPTKT

ANQQANELSNITAMVQIGSVGGALIAMFVQDRIGRIRCLQEMIILWTVGVIIEVTSYSQG

QLLAGRFVAGLGIGQSVVVGPTYLAEVAPKNVRGLCTCIFSGSVYLGVMLEYFANYSTTL

HMSPNSRIQWVLPTAVQFIFAGLLFIGSFFIKESPRWLMKIGKDELAVETLSKIRHLPVD

DLYVQGEIVDVREQIEREKQALSGTSILSLLKELVSTKANRYRLFLGIMVQLLGQWSGAN

AVTVYSPKFFSMLGIPSKTDQMMYTAVLGVIKFTSAICCALFLIDTIGRRRSLYTGICLQ

FVSMLYLGIYLAIVPATVGVDRSPSQKKAGGAAIAAIYLSGCGWALGWNSIQYLINAEIY

TVRHRSLASGIIMVFHFANQYGNSKALPFMRSGITDHGSMFFFAGVLLLGLAWSWFFLPE

VSGRSLESIDEMFSLPWYQIGRRGHKLVPETGTVIQIQEEEEKKGGVIHVENC

>tr|Q6BZZ0|Q6BZZ0_YARLI YALI0F29711p OS=Yarrowia lipolytica (strain CLIB 122 / E 150) GN=YALI0F29711g PE=4 SV=1

MSKQPRDPPKVNIPPLSKRSPGCPLRPTQSSHETDVLGEQIQGSLVKASKEDYPGYLKTG

PQSTRKESFFDPDFKVISIETGVVGSKDDISPGSEPPGTVLLETTLNNTTRHPTPSSDPN

DPLNWKPWRKALNFFLVLFFTVVAFSCNCVATAFWGQLNTELGFSFDELNNGFALGIGGL

GIGCPLFVPIAVKFGKRPVYLVATILVIVATFWQARMKYLHDYYAASFLEGLATSLCEAM

IQMTIADLYFVHQRGTMNGIFMIIIAISNFLSLVPAGYIATNINWNTCFWILGGLGSGFL

IVSVFCFEETKFERAEDDPRDIHELHPPQGKMKWQLRPYTITTGSWGDFFKTFYTPFVVL

ISFPIVLFVTLHYTFMLTWLAMCATTLANSFTQPPYEFSPEAIGNMNIAPFIGLLIGSFY

GGWLNDKSMLYLTKRNHGVYEPEMRLYMLIPMCLIMTAGVFMFGLSIATGTHWAVPMVGL

GVFAAGFGANGSITLTYLIDCYRDMISDAFIGIVVVRNIMSMVVTFCLSPWIDLNGLRNV

FIIAGCLSLVPLVVTPFMIKYGKHLRRLSADKYNKNKGGI

>tr|Q6C2Q4|Q6C2Q4_YARLI YALI0F05984p OS=Yarrowia lipolytica (strain CLIB 122 / E 150) GN=YALI0F05984g PE=4 SV=1

MSDAEIQSQINGLAEKYGVNQRRLMMKVDLCVIPTICLLYILAFLDRVNISNANVYGMSA

DLGLKGNHFNVALAIFFVPYVVAEIPSNWLMKKTSPHVWLPGCMVLFGCTLLGQGFVKNY

GQILATRFLLGLFEAGMFPGCFYLISMWYRREESQKRYSFFFSSTCLAGAFGGLIAAGIH

NLDGHRGIEAWRWIFIIEGACTAFIAMLMYFVISDFPEDAKFLSENERQFMKEKLAVGTV

GGSEYDRGMSLKDLKFVFTDWKIWMSGMMYFGLVVPAYGYAYFGTAIVKTLGYSEIKTQF

YSVPPWVAAFGLSMGAAIFSDRFRHRFWFVIGSCIVCVAGFGLIMGEHHKIGTRYGALFM

ICAGAYTCMPMLVCWTQMNFSGHHRRAIASGWQIGFGNIAGFISTFVFKTEDAPFYMTGL

GCCIAFTALSGILSVVYYFGLRAANKKKQSPDYAVEFSQWPEDKQRMAGELHPSIFYTY

>tr|Q6C471|Q6C471_YARLI YALI0E29227p OS=Yarrowia lipolytica (strain CLIB 122 / E 150) GN=YALI0E29227g PE=4 SV=1

MAKRPRSASHHVPVAYHPGSLVSLNRTQRKLTEYSEIASWGWILLITTWSLIFFAVFTMF

DLDIYLFGSKTDRKNMHKAPDDDDDFPIRMYYPTSFFLSLVMAWVWCIVSWMGLKFFKHA

KVDPTSR

>tr|Q6C4K6|Q6C4K6_YARLI YALI0E25982p OS=Yarrowia lipolytica (strain CLIB 122 / E 150) GN=YALI0E25982g PE=3 SV=1

MSNALNLSLALGVFLLAYYGFSVIQYHIKTRKLEKKWKCGKPKDISRFPFSASFFIPFLV

ESKKNRLLEFVQWMFESQVYPGYTCKTTVFGVDMYHTVDPENLKAVLATQFKDFCLGERH

AQFLPVLGNGIFTLDGQGWQHSRAMLRPQFARDQVSDVEMIEEHIQYMTSRIPKDGSAFD

AQELFFNLTLDTATEFLFGQSVGSQTVETNPTAVPTDMPVHLRKSFQEDFNTAQEHLGQR

ARLQMFYWAWRPRELYSSGERVHAFVDHYVKKALEESEKHVDDGKYVFLRELAKETKDPI

VLRDQALNILLAGRDTTASLLSWCLYLMARRPEVYAKLREEVIENLGDGEDLSTITFESL

KRCDYLRYVLNEVLRLYPSVPANMRYATRDTTLPRGGGPDGMQPIVVRKGNLVSYHVFTT

HRLKEFWGEDAEEFRPERWYEDGASQAKGWEYLPFNGGPRICLGQQYALTEAGYALARIA

QLYDTIENADDKPEPPVKFHALTMCHHTGVLVKLYNSKTTKAQ

>tr|Q6C4Y1|Q6C4Y1_YARLI YALI0E22803p OS=Yarrowia lipolytica (strain CLIB 122 / E 150) GN=YALI0E22803g PE=4 SV=1

MGSQSRRQSHNGGSVLELRPTLSGSSGIIDEFDASAKKHNNKKSRALRRRRWRVGLILLG

LVVLLWVASGFLVNSIFSTGEYPKPYFLTYMNTAVFSVYLIPTMFRKVRGNKTATPEYSV

IDENSDESPKLTPFKSVEQLTGQDEDELLSTKQTAILSLQFCILWFFSNFLTNASLKYTS

VSSQTILSCTSSFFTLVIGSAFGVEAFTATKLLALVFSMCGVFLVSKADSVATQTRMGVQ

TSDIVFGDLLALAGAVVYGFYMTLLKVKVGDESRINTKMFLGFVGLFNILLLWPTIPLLD

YLGVEKFGLPQTEKVWLIVLANAAATLVSDFFWVLAMLMTSPLVVTVGLGATVPLAMAGD

LFIKRSLPSLTYVFGAIILCLSFVVINRQDEDELMDDDEE

>tr|Q6CA71|Q6CA71_YARLI YALI0D05357p OS=Yarrowia lipolytica (strain CLIB 122 / E 150) GN=YALI0D05357g PE=4 SV=2

METPNLSIPEGISDELRVPLEKLIHDFKEGDITAKGYQKRREQLIAVYIQRHQHSRSSDR

VDDSPASSRPASHHIYSLSKDLGSASSPSSFDVAHESVGEEEEALLMPLEPREIPDTMRD

PHNTAVAMAKFDNLPSILRHRAKTNAAHTAIITLDDAKGKDVNVITWEKLASKAERVAQM

IRDKSNLYRADRVALLYQDSEVVEFAVAILGCFLAGVVAVPINPFYHFKDTTYVMHTTQI

HLALTTEVTYKIVHKHMVQDRQQWPKGVEWWKTNEFGSYQKPSKHAEMPALQVPDLAYIE

FSRSPTGDLRGVVMSHRTIIHQMTCLTAMLKSRDKFVQPDNRYQRGDVILSSLDSRQSIG

LIMGVLLTVYTGSTLVWIPHSALAVPGLYANAISRHKVTILLSDYPALKQVAYNYQSFPQ

LTRAYSKKQQVNLASVKWCLIDAATVDTEFNEILADRWLRPLGNKHAYEAIAPLLTLTEH

GGMVISMRDWLGGQEKLGKGSTLSLDDDGEDDGPYELSKILLDKPSLTTNNIRLIPPVRG

GEDSLKHIRVGAFGYPLPDSTLAIVNPETRKLLPKMVVGEIWIDSPCLSGGFWGMGPETD

MVFHARCYGNSGMLDLEFLRTGLLGFIYGGKVYVLGLYEDRLRQRYDPADEDADATAGAG

SSGASTKQQSLVVAPPYRYHYTSHLVYSLVRKVPNVFDCSAFDIYLNEEHLPVAILESTL

AEQTPINPGGPARQLNYEALDDLARGCIAVLQESHKVRVFCVLITAPHTLPRTMKNGRSE

IGNMLCKRRFSQGLLPAVYCKFGIIQALKTIPCGQDPEGGIWSSAISHIRSDYLGMADKQ

YSGIELRDVVRDDRTSTPLSAFGSLVEILQWRVAHQADELAYSTIQQSGKEGKALSWKKF

DQRVSTVCHYLKNKVGLKQGDHALLLYTHSEDFVVAVYACMALGIVAVPLPPLDSGRLHE

DIPAYCGVISEYKIKAILVNSETETSMKAKLISQQSKQIAAQLKVVLPKKYNTSKPKLAH

SGTRDLRYIVKPEDTNRPAVVWLTWSAEHRRSGVMLTHRTLMGMCKVQKETCQMASTKPV

VGCVRSTSGIGFLHTCALGVYLGASTYLVSPIDYTVNPLTLFLAYSRYKVKDVYSTPQML

DYACATLKPKGFSLAETLNLMIAYDGRPRVDLAKNLRMLFLSTQLSNTAISSLYCHTLNP

MVASRSYMGLEPIDLWLDPIALRQGYISVVNPENYPNALHVHDSGMVPVGTRIAIVNPET

RQLCKVGEFGEIWVWSEGNVQQAYPARDEFDRARFQGKLDDDSNEAQAITRQGDYVRTGD

LGFLHTVSRSLASGGHATEMQTLFVLGSLGATFESLGLSHFPQDVENTIEGSHRHLCSNG

SVVFQAADHVIAVCEVTTDKFLASLVPVIASTVLDEHQLIINVVAFIPIKSMPKSRLGEK

QRGKVLSQFVSKKLKTLQIFGVCEGQSTVLKFMKKK

>tr|Q6CAY5|Q6CAY5_YARLI YALI0C23342p OS=Yarrowia lipolytica (strain CLIB 122 / E 150) GN=YALI0C23342g PE=4 SV=1

MNYTSAILLGVMLLWPWNAFLLATPYLRHRFLPIPTLSNNTASSIMTVSTVTSVVTNMWL

QTWKKDYRDRVVMGHVIIASVFAVLAVLCVLFLWLPTAFYFCVVMLLDCLSSVGVSVAQN

GSFALASERNTQGIMMGQGLAGIMPALVSLVATTAGDSVDYSSAASWSTAFSFFVATAIA

GLSLFVFSRSKPAKKDLEQEPFIGEEDLTSSTELRRPESPAYPVPGDEPASNVPIRVLAE

KLKAPAFSIIFTFMVTLSFPIFAELVEPNNSVSQAIIPIAFVVWNGGDLLGRSICAKEKF

VVKGSRNLVTYALLRFFFIPVFFLCNIKGRGAVIPSDIFYLLLQFCFGVTSGHLSSSSMM

SPGAYVSKSELSAAGGYMTLCLTIGLALGALASFILVALIG

>tr|Q6CET9|Q6CET9_YARLI YALI0B12980p OS=Yarrowia lipolytica (strain CLIB 122 / E 150) GN=YALI0B12980g PE=3 SV=1

MESTPPDYTGLDPTIDAEIRSIAESVHKDRVDDYDTEKGTVGNEKLLRSDTVQPNLDVNP

YIDNSDPQLDPLSDEFNARKWIKTVLGLKQRFGATKHITAGVSFKNLAAYGYGGGSQYQK

TFSNSVLAIGPMIMELFGGNKGTKVQILRHFDGLVRAGETCVVLGRPGSGCTTFLKSVAC

ETYGFHIEDKTEWNYQGVPRKVMTKNARGEIVYNAEVDVHFPHLTVGDTLLFAALARTPH

NRLEGVSREQFAKHTRDVTMATLGLTHTMDTKVGNDFVRGVSGGERKRVSIAESVVCGAP

LQCWDNSTRGLDAANATEFIRSLRLSAEMMGSSMFVSLYQASQEAYDLFDKVCVLYEGRQ

IYFGLAKEAKQFFLDLGFECADRQTTGDFLTSLTNPIERIIQPGWENKVPRTPDDFEKCW

MESQARQKLLQEIEEFNTEFQLGGESQDHFMELRKESQARHTRVKSPYTISWPMQTRLCL

WRGFLRIKGDMSTDISIVVGNFIMALVLSSMFYNMQQTTDTFFSRGALLFFAILLNAFSS

VLEILSLYEQRPIVEKQTRYALYHPFTEALASILTTFPTKILTLLAVNLTLYFMTYLRRE

PGPFFIFFLFSLLCTLAMSMIFRCMAAITKTLEQALAPASVIILALVIYTGFSLPITYMH

GWARWINWLNPVAYGFEAVMVNEFRNRDYGCASYVPTGGEYDQIDLQYKSCSVVGSVPAE

SSVNGDRFLGLAYQYYNAHLWRNMGILFGFIFFFGFCYLVAVEYIQGAKSKGEVLVFRKS

YLKKIKKHAANDEEAGAMMAGEKSEEPASEDSSINIQAQKGIFQWKDVCYDTKVKDGERR

LLDHVDGWVKPGTLTALMGASGAGKTTLLDVLADRKATGVITGDMLVNGEPRDESFQRKT

GYVQQQDIHTSTATVRESLEFSALLRQPSSISVKDKLAYVDEVIRILEMEAYAEAIVGVP

GEGLNVEQRKRLTIGVELAAKPELLLFLDEPTSGLDSQTAWSIIKLLKKLAANGQAILCT

IHQPSAILFQEFDRLLFLASGGRTVYYGDIGPESTILTSYFERNGADPCPKEGNPAEWML

EVIGAAPGSTAKRDWPVVWNESSERAAKRAELDEMAQTVERTQTNNTERDASGYSDSDQF

AVGQWTQFKIVTKRLYQRFWRTPSYLWSKIFLCTASAIFIGFSFFKAPSDIQGMQNKMFS

FFMLFLIFNTVVEQIIPQFDQMREQYEARERSSKTYSWQVFMASNMVVELSWQFLMGVIV

FFCFYYPVGFQWTAEHNNSVHERGALFFLYVLLLFLYNSTFAHMMIAGIGNKDTAANIGN

LLFSLMLLFCGVLATKEQMPGFWIFMYRVSPLTYFVGGMLATGIGRAPVTCSGNEIVRFQ

TFPGKSCGEYMQGFIQALGDNGGYLLNPAANELCEYCPMKSSDTYLSMVDVSYTQRWRNW

GIIWAYPVFNVAMAFLLYYVCRVPKKSKAQKA

>tr|Q6C1N3|Q6C1N3_YARLI YALI0F14795p OS=Yarrowia lipolytica (strain CLIB 122 / E 150) GN=YALI0F14795g PE=4 SV=1

MKLFGLSVVTLLATIGSVVADKQLENRQLFRTVDLSKSYVREFIAVNVYNDGTSDETDYI

VSIPPGFVDDLAKLDIKESSHEAPLKVIRLARDEHGEHWSAILPEPIKPGSQVQLQLFLS

YVNQLQPVPQTIKQSEQQHLVYDGLKTFYSLYPTKSQTNRVKIMAQNMQEINMDENSTPS

TEAEGGAQYGPFGPMGSFEVEPLVLRYDYPLPIPRVDSLKRDIWISHVSNSIAIEESYNL

TNAGAKLDGSFSRTDFFAHHGKPSSASASIIELPFTLPLGAHDVYYTDLVGNVSTSHFRG

GKEVSLLELRPRYPVFGGWNYNFTIGWEMPLTSVSRTTGADEYVVGLPLLNGPVDAQYRQ

IESNYILPEGAENVQVVTSEPALSKEESVTFSYLDKKGRPTIQVRHVNFVDQHRGGIVFL

KYTLPGSAATIKPIFIATVIGSIFLAIFSLSQLDFTISQRPKKTTKE

>tr|Q6C2J5|Q6C2J5_YARLI YALI0F07359p OS=Yarrowia lipolytica (strain CLIB 122 / E 150) GN=YALI0F07359g PE=4 SV=1

MTFADWLVAASFSHLITALYVGSLYLSKSTRPSKLVRRDDEVVVKKRMQIISLGTLVDVL

VITPLVLQWQLKPQIVTWSETIKFMDIELGPHFFTRTVPTSVLALIHVAILFAGPLYQRI

YLENGLPLGFHLAKRELKTVWGIRNYIVGPITEELVFRACIMSVHKAAGYSPLASVFVTP

LYFGVAHLHHGYELYVSMGSKGKNKVMVIALQCAVQLVFTTLFGWYAAWLYVRFQSVWPP

VCVHIFCNAMGVPDFSVDGVTKKQFKTYQTLLAAGFVGFLATMTLLIRW

>tr|Q6C3S6|Q6C3S6_YARLI YALI0E32461p OS=Yarrowia lipolytica (strain CLIB 122 / E 150) GN=YALI0E32461g PE=4 SV=1

MPSINILCSSALPFVPTKFQIDAHEHETVLVVKKRLCQKLVELQRQGSVETSTESYNREQ

EHLGNPGNLKLIFGGKILPDANELPNLAATNPTFHLHLLPVSLDKEKLSIIERFRRGSTS

KGDANGGAESSGSTPAESQTMYNVRLLSNQAKPTIHYNVQQQSLYNMDGEASLADLKHLL

INSLGNDTAVKSLDLAIQRKFVVFNSKGQDFSDDTMKVKDINNFGRPPHSQGTNVVIFYR

FVLGEFEEYQMEGENEVLEKPAEAQSTPVKSNNPFNKEVQQQQQQQQQVKSMVEPDGSLI

QFDRETAAVSVATSPSAALSGNPLAGAFTAQLNLDNEGKGKEVAEEVDEEGDVEMKEGKP

VQVETVETPVPTPTIKHLFIKDTSLGDFHLNPTDVKTGSCTESEWVAISPREMTRLKRMY

REFGNSETEPKRRKSGEFTRETGEAVASGRDMAGRGLPGRGMGQQIGTGASTPSSASAAS

NPSTSSESNSGPSSSSSSDSHANNHPHTSNHSHTHNHNHNHAHVNPTPNSHLHNDTANST

DTEAEVIQTPPRGAAHSPGNTTPASDQYRQFQQFLRWADTQHEDHTEPAEFDYRQFQQFL

QWGQVQPPTQQPLVDFSFRLRISPPPWNIRQRIREMIPPRDQLRQRAVVAVRQLGEVLVF

LLQLFVFYIMVLGRINNDYMFYASCGLTVIIFVGYRFRHHLERFRPASFNVGSEELSRVA

QIFPAVNYDQEEPAAGGNNEAVREALENPDNRHARGVRIGAAFFGSMIPAVHSRWLADNR

QRRVQLDALIRQREREGEETQTQEGEDNEGDEGNNRAEQNE

>tr|Q6C3Y7|Q6C3Y7_YARLI YALI0E31218p OS=Yarrowia lipolytica (strain CLIB 122 / E 150) GN=YALI0E31218g PE=4 SV=2

MAPPLNNPNYQPQQQNPNYQTRNNDEPSNACLYVLAVFFPFVAVGIKRGGCSGEMWLCLL

LSYFFYIPGLIYAIYIIQQDSDKRRAQDVEHMGQGYATQYQQPQPIQPHYNQQPLHQQQA

YPTEGTYPQVAPAKHEPAPMTQPPVNQHSGVAPQMHSGISPQAPPPTYAEGPSQPLHEKA

QYVPPN

>tr|Q6C5W7|Q6C5W7_YARLI YALI0E14509p OS=Yarrowia lipolytica (strain CLIB 122 / E 150) GN=YALI0E14509g PE=3 SV=1

MTLTDLVRAEPSGALLVSLATAYLVYLVVVAQLNPLKKCPGPWYTLYSKMPILVIFWRDS

RNRYVQALHEKYGPIVRLGPDEVSICDPKDVSKVYLGNFDKSRFYAQFGNYGAPNLFCIL

TKEPHIKLRKLMTGFYRKETINGTGIIQEKVDEMETVVAANNKQDVHTLFRYLAFDVVTR

LTISDSEMLAGRNQKLVFYHEMQTSMTFWTTLQPKWWDLAAALSERQTVLGEKTSISEAS

QRCNDWTFQQTGDPDTLLDMLKSKGVSHKIAMSEVQDHIAAGHETTAVTLAFLFHRLAHD

KSIQDKLYAELEPINVTLESVDSLPYLNGLINETLRLHAAIPGAEPRVCPAGVIFQNVAL

PKGTIISAQPYTLHRDKAWGDPEEFRIERWSEDPPLAYFMPFGAGIRMCIGMNVAMGELK

LATAQLVKKYEIKKSSPQQQFPTMKDSYTTLPEGDNTLLFEQRAT

>tr|Q6C6J4|Q6C6J4_YARLI YALI0E09042p OS=Yarrowia lipolytica (strain CLIB 122 / E 150) GN=YALI0E09042g PE=4 SV=1

MRHSIDTPADDYSDSELLSDSNMDMDADELDAEILDGLGNGKKLGMSKPKNQKKAKKSVK

IEVPAASDESPAYDESSPYGGQGLDYEGGDQSRDASGENLTSSQDQPKTVHWPEEDIDEN

YPDAIPLKEVKKPKKNTLKSIIKKGKSKFGGNSSEDSSSYSLLGGGRRSSLSGGRSGRYN

KVNFNRFATDSSDEDSDADSDFGQISIDVENRPRRKVRHQNTKTLNLLLFALLIIAVYFI

LTLTFGGNTGPKGRPSTYKSREKILLNNGTHDFHPTTLVISLDGFHPHYISPELTPHLDN

LMRKESGAPYMLPSFPTSTFPNHWTLITGLYPSSHGIVGNTFFDPDLNKQFVNTDPSRSR

DVAFWGGEPIWQTLSYQNVSTAVHMWPGSEANWLPEDAPLIVDPFNQTEALYKKLDRLTE

WLDRPIQTRPELMLTYVPTVDTLGHVHGISGSELEQGIHEVDLLVGAFREALDNRNLTDV

VNLVVLSDHGMAPTSDERLIFLDDLIDVGQIDHLDGWPLVGLRPGNNMTESELFNILKKQ

EPENESWKVYTRKNLPARWNFGGKTGGRYQNRLAPVWIIPKTGWSILTHDDYAKMDNSYQ

PHGTHGYDNRDILMRALFLGSGPYFPDFKFEPFSNVNVYSILCDTLHVYPSKNDAQPLTK

ALVRLNDDWQDDSDLYPGVDFSTSVVEGSTYDELYRTEGHANGSVPTNGQEPSSQKGGKK

QSWADYLKGQTNEAIDWLADKWAGIVGHD

>tr|Q6C7W7|Q6C7W7_YARLI YALI0D24772p OS=Yarrowia lipolytica (strain CLIB 122 / E 150) GN=YALI0D24772g PE=4 SV=1

MSVPDPTNALETTPLNPFHNPWREQSPWRIIRPLSVAFLATAIAGVIGPNFLLATLCKHY

YDGREGLDPDSLTISNPLCQISHLQVQAGTNQSRLNLCRNVVALIVFPRLSAWSDRRGRK

PILKIAAGFTLVSLTLQWCVQHFWRWFPVPTFILAGCLDLSAVVFQLGVLAIADSTCRRT

VNTSYLFVASYLFTALGSYVGSELAKRGLSTLAAVYVPLYLVFFIWTWFVSETLPYSERD

KATEVHNEKTDSRRKTLLYYGNPLQPLAALLSPAHRRIKIAMVIFAAACALEDTFYQTSQ

YVTLLYIEYRFKWTSIQLGKMQAASAAVSLVMVCIAPFILRELEKRRNPVTQDIHVLRTA

AFWTASLATIKALAGSVAPFVVAHVLLGLRAVADSPHKSVAMALAPSAAAGQVLGALEMA

VTLVKIPASTVLVRMYSWTVYTNPAAVLWIICFGEWIAFAFFLALWV

>tr|Q6CHH1|Q6CHH1_YARLI YALI0A09020p OS=Yarrowia lipolytica (strain CLIB 122 / E 150) GN=YALI0A09020g PE=4 SV=1

MSESNAALAAAQHSVANPIPVAPKDPHPHNPLHPHGSRHDAHDAHALGLGTPEHRRSLTS

NHEVNGHAATPASKTAPSIASVSSPKSHTGDRAGSSTIPHSEMNDQGVPTNQLKPNAAAN

MPITLIPVIFKEASLDSPSFRATVNHVISQLAVTSQWLEGFVQSVHDVTAEMHTLQAVIN

NLVGKFMPSFVTEGVVDHDYTMLVLRRFAEGSKLFWTNAMKNINGQNTHLGEPLVKLLKQ

RIAPFSETVKAFRAAQTKYDAAQAKYLALNKTREPSHLRDEAMAVYEARATYIKASFEFC

AGYLRLHHAIDRALILTITDTWRSNARNHDDSAHQHAGSAYFRLGIEMHRIRNWSNAIHQ

ANKVIVREIGKARKEIEDSTLAAAKPSKDIAEYQPLSPSTKLSVDSKVTNHPIEKHGWLL

MKTNSPAEKNPIWVRRWAFVKAGLFGWLNVSPPPECDFVEESDKIGVLLCSIKQLPTEER

RFCFEIKTQEVTLIAQAESLTEFKSWLVAFELAKQQSLDKESPYRSSLAFKKWTVPLPEF

ASSGGTSIDVAMAHTSSKTTDTTSPSYLGGPHAYASEHHVHQTPLHKLMASGKAFIDGNE

TNEHGEKSAFNFSGFGPFNTSLAPSPPIITPIYTRMTKEAIVSTAFVKPSYMPNAVTANV

WGSVNWALYAKNSTVAIASHENMVEFQRKMDENPTTETDPFPENYPIELKSQDLQMRAIF

QTAVDHDPNDRVVTVFRCLWSPNPRQELPGRVFVTLKNLYVYSYSLGFVCAVKKPLTEIV

SVQGSPDNAWENLYVLGTDGAVLRCRVFLDSGRLVQRRLQIILSNVLSDSPRNLEEMLPI

LQDGDSDHKILTENDGWYERESDLGYIDNVQGEHDEKFSQEELSEHLVKRYRDLRNKRKE

VKATKRPTSSSARSVASKTVDTPDYSLHSSTAQKVVDREFLCPPKALFHIMFGQKSRMFK

DIYSRFHKVISSRRTRWMLNPEDKTLSRLITYDLKLRSLMETGTLVSRNLQVIERMDENV

CYVVHNSRNPWEISHVSSFYQSMRFVITANDTGGSRLCIWDDLVYTEKKRSLFKSVVETV

AMRYMEHEAESHVRLIQQCLRRVGTFDSTNRSIIMYGKLGQIDTDPTPEQLAELERKDSQ

DVLKVSRRRLVTWVSESIFTFLLTALTAFMIIIVKLAKGFFKSLSPHIVLIVLLAVSGVS

NLWLSGRLTYFHIKQHQEARRAAPSDTLYRAVFINDLDPMMLPSDTNNTDSIALQKYLSL

EKSRVFGMDPDKILKGDDGIGLEDKFFENKYFSDVASRSASRKIQKARYMLGIKRRELLV

NLRVLNRSDRDLAIEEWQNWLRSEVRNCERFTARVAADPLYGKDKLDEGKFEQIENYCNS

CRAEIKVPALL

>tr|Q6CET5|Q6CET5_YARLI YALI0B13112p OS=Yarrowia lipolytica (strain CLIB 122 / E 150) GN=YALI0B13112g PE=4 SV=1

MRLQSLLLLCMALLVLAEPGLTPIDDRENWVGQACGYIDTVRWAGFKKVPRKDTTGAGAL

QKCKSAPYLTSVALCGEANFPGNWHRIYKVVDSVALQACRKYNLDVTSDQVRAMYQNYSK

LAIPIPRVNKTAMQTKPIGFNQTWFDEKYPSIRVYQHNLDLGQYYGNGMTAWWALILVIG

IAINVFRTVLFPQYLRWTSNSNILRRQLSLPATFGYKHSQPLQTMWGFVSWCCPTRVQSL

VILAYLAMCLILCCVSYEAMDSTTRWKKRSTQIQRYIGDRTGIMAFTQLPILFLFAGRNN

FLIYLTGWSYSTFNVYHRWIGRVMVTLAFVHSVAFTINSLKALTFYYSFPFMRWGVVATT

IGALMCFQGLHFFRSHWYEFFLVFHILLAVFFTIGVWRHCRTLGWMEYVYAAVAVWGFDR

LVRVIRIFASGLGTATMTMADPSLGIVKVDISYSKLWKVKPGHHVFLYVLNGIRPWESHP

FTVFQTCEAAANGNMSIMFRAKEGKTYNLFNKLKDSKTGQFKVLIEGPYGHHLPIAKYDS

QILIAGGIGITAMYSYASECVRRSSGSSGTLNFNWIVRSDSCLTWFHDELALLLSDSRVT

VNLYVTNTRGDFESDQSSIESKNEKDLSDSPGPTPFENTNLNVIQGRPNYVSDLRRMLEE

APGSTAVSVCGPPKMNDDVRLAVRKNLDVKTDRIDYFEEAFCW

>tr|Q6C1C8|Q6C1C8_YARLI YALI0F17402p OS=Yarrowia lipolytica (strain CLIB 122 / E 150) GN=YALI0F17402g PE=4 SV=1

MHQALPRPGGSENSFVPRNGRSGGLLGQIRATAQRIAGPSFNHRGSSNLRALVVGLAILL

SIYTFVSMLTTPAGYTYKTGIPVLGGGPDSIKVAYSKQPYIYPTIANEDLKLKDGADKDR

QTVTQYTQVIYRDGGKSKPVTQANIVNGHAPFTKSTPGKPRTVIVMSLDGTDAARADSEL

SLTILQNRLNYAKKHGFGLYARFYQDFLNPLSGHGITPENYENWAKFEIIRAAMQAFPDA

DRIWWLDSNALIANDKFNVETDLCDPVKLEKIMLRDVPVIPPNGIIHSYKRVPAKDIQLL

LTQDDAGLTSASMIISNDQYGQAITDYWRDPLQYKYEDFRKQQPGNPENAALTHMAQWHP

TVLSKTAIVPARILAARAFDGEVLKDVQYSPGDFVRLVPCHSAYQCPTQWLEAVHESQSQ

AASA

>tr|Q6C2A0|Q6C2A0_YARLI YALI0F09537p OS=Yarrowia lipolytica (strain CLIB 122 / E 150) GN=YALI0F09537g PE=4 SV=1

MSEVAELRMEMERELELLRSEVDELKSQLIQQDAERHEAQKNQGETNITKENVTAAGVLS

ILLLLGSMGPGWYMGFSVMFSEGDGTGIGLVMANMFLPVLILIVTWVLPLMDRDRLILEA

IMLLGYGSFMIYEMTKSVWSPLAQILMVSAAIFLRHLHQNEIHRRKQGLPVSVDLGDDV

>tr|Q6C7H9|Q6C7H9_YARLI YALI0E00704p OS=Yarrowia lipolytica (strain CLIB 122 / E 150) GN=YALI0E00704g PE=4 SV=1

MSHHFEQRYDTTDEKLVIERCEQMKAGLGVNRATKNMASPLGRFSLGAGWLMHWLYLHDT

SLTGNMRTIVPVAGFVALVGVAFFISKAVVPKPIVFSEPNSHVTINYYRFLFVVLSTRLY

INGKFGLSSDGAVDYTVLGLKIAILIASQFWATLITKRLRYLPGFGLPQVFSKKTLDRFG

SMIFTKYVDVGLGVAWAFPLSYVALYAIVQPKDDGFELLGWPLKALGLYVMAQVYGVHHK

MAIPYFAYFHPVESLRFMEYGIDVTTEQGKLALQRRLGL

>tr|Q6CFJ8|Q6CFJ8_YARLI YALI0B06347p OS=Yarrowia lipolytica (strain CLIB 122 / E 150) GN=YALI0B06347g PE=4 SV=1

MNGSPLANPPTTTTGRTTSPVVQPQTRTQITTQTQLPSQSTHHYPSLGYFKRPGVTILVT

SLFFFYLGYGVLQLTGPDAFLIAVCRRTLNDGSLTEYASACQTDAIHTKVATMGGRLRTA

GSLVGLLVKIHIYKLSDRYGRKPVIVMSMVAVLFAYLLQWGFVTYALSVPLFLWIIPNIV

FCTHFLMDFFLIYLADAASASKGKRSLQELATDVSFFYAFILFLGALIGPMLANTVVATN

AAGSSFAEMSKLGLMLVFAGVCCVVVILPESTTLESRDRATVKWDAEWSVLKWDERLIYV

FNFVAPISKVLRLAPSPQDKRKNSFLLAYLALFGLYGTSQYTGFLFYKRQWNWGMQEITI

LNVEVTIILIVSQIFLGPLFVNTAEKLLNNTTSSTPVAILGCKLQAVLTFVVGFFQLLFA

SQTIGTLAAILSGLSGTFISSTIALQMGLGPPENMSVIMGGIGVARSLVAVPSEMILAVL

FVTTISAPRIYQAALMAVDAVSLVLLGAASRENTGSIMLP

>tr|Q6C9M7|Q6C9M7_YARLI YALI0D09889p OS=Yarrowia lipolytica (strain CLIB 122 / E 150) GN=YALI0D09889g PE=4 SV=2

MSLHYFPPIKPSALAVGTIFSHFSSLIGFAPVIGDTIKRAKAADTPEEFARQKENNGVLA

LYGSSLLGSGLQSYAVSALIVLTGTTTTKGAAYLGGLIFAVNSIPTLVTGIFQENRPVEY

LVGKTLSALLETVGLTLTLNWWGTRNETLSLAK

>tr|Q6C5F8|Q6C5F8_YARLI YALI0E18458p OS=Yarrowia lipolytica (strain CLIB 122 / E 150) GN=YALI0E18458g PE=4 SV=1

MPASGEFTWQLTGNVAINTLFSAAFPVFTAIYAIRGLKQGAIETASKSEARLAKKLDIDA

ETLYENYSPLILIGYPIFAVNLQPLGTLALLWSRTTGLIDHLSDQQLENALSTWSKFSQV

YTWATGGICVAALGIWSRRRQQRRSKQVTKKMPLLGAPEISLLLFSAIFLPVVSQPIEVF

P

>tr|Q6C5T1|Q6C5T1_YARLI Aldehyde dehydrogenase OS=Yarrowia lipolytica (strain CLIB 122 / E 150) GN=YALI0E15400g PE=3 SV=1

MSEFDWESILPATPLGEIEKDIQTLRQGFRSGKTLDLNFRLDQIRKLFYALYDNVDAIKE

AIHKDLGRPVFETELCEISFQWGEFNNVVSNLKKWAADETVKGTTIQYTLTRPKIRKRPL

GTVLIISPWNYPFVLTISPLLAALAAGNTVALKFSEMCPHTSLLLGKLCTEALDKEIFKA

FQGGVPVVSEILKYKFDKIMYTGNHRVGKIILDAANKYLTPVILELGGKSPVFVTKNCQN

VSLAAKRALWGKLVNAGQTCVAPDYIIVEPEVEQEFIKACQYWVEKFYRGGVDSDHKDFT

HIATPGHWRRLTSMLAQTEGNIITGGNSDEKSRFLAPTVVAKVPDGDSLMNDEIFGPILP

ILTARSVDEGIRYVHENHDTPLAMYVFTDNASEGEYIQSQINSGGLIFNDSLVHVGCVQA

PFGGVGQSGYGSYHGEDSFLAFSHRQTFMKQPHFIERPMAIRYAPYTSRKQKAVQGSLAA

PSFPRTGKVDRSLLERIFGKLWFWVIVLGLGAASLKSGIFL

>tr|Q6CDF8|Q6CDF8_YARLI YALI0C00869p OS=Yarrowia lipolytica (strain CLIB 122 / E 150) GN=YALI0C00869g PE=4 SV=1

MVPKLVLFTCGILTVLTLCSYFFIKAPNYTQLIRASDVEYQFRLLRKDGDNPEHLFLPYR

NTRWDPDDTPELNNQLNHYMKHMTDPYTRSFPRQDELPPLVNGMFHAIAKEQMIAPAQFS

FEQARESYKTTTGRCPPPQFKEMYLGALAKGEWDELLVDQVTQQIKKLPKGQDLQAALKE

ALKGPGIEGIRISNGTVERSSEAKLSDVSQRTLDGFMTMVGSVSKYLPDMELALNVEPFP

RVLKFPKRLKELRKELTKGTLWAVPEGYDLDRYVGEMCAGIKAEALRGVFRDGLLGGGSP

LTYSEACYIRNDWGKTPVVSAITMLPVFSDFTSSMHSDIVYPSYDFYAPELSMNYQPERD

IYHMEKNSGVSRVKAGDWGYLKYQKALVRDTKEPAATVSVKGKRFSKKSLDNHAFLGALK

SRSTVLRASRWRTWLDLYTTNAWVSFIPVQLSGNDTEVVTKLLSERHTTAVGQAFASRGA

RDVRYRATRKKIELSLYYMLLEYHRRMYDPEACEDEIIARGGGQIQ

>tr|Q6CF55|Q6CF55_YARLI YALI0B10153p OS=Yarrowia lipolytica (strain CLIB 122 / E 150) GN=YALI0B10153g PE=4 SV=1

MDSTTQTNTGTGKVAVQPPTAFIKPIEKVSEPVYDTFGNEFTPPDYSIKDILDAIPQECY

KRSYVKSYSYVARDCFFIAVFAYMAYAYLPLIPSASGRAVAWAMYSIVQGLFGTGLWVLA

HECGHSAFSDSNTVNNVTGWVLHSSMLVPYYAWKLTHSMHHKSTGHLTRDMVFVPKDRKE

FMENRGAHDWSELAEDAPLMTLYGLITQQVFGWPLYLLSNVTGQKYPKLNKWAVNHFNPN

APLFEKKDWFNIWISNVGIGITMSVIAYSINRWGLASVTLYYLIPYLWVNHWLVAITYLQ

HTDPTLPHYHADQWNFTRGAAATIDREFGFIGSFCFHDIIETHVLHHYVSRIPFYNARIA

TEKIKKVMGKHYRHDDTNFIKSLYTVARTCQFVEGKEGIQMFRNVNGVGVAPDGLPSKK

>tr|Q6CH20|Q6CH20_YARLI YALI0A13585p OS=Yarrowia lipolytica (strain CLIB 122 / E 150) GN=YALI0A13585g PE=4 SV=1

MAIPRRVTRLIVVALVLGGLLHLLFAGSIGRKRYAFTNDPQTGPSQPTPDEGPQEPQYAV

DNVVAEPDTSHLQLTPPEREELEKQAHQEVTQEEKEEAINKHLDTAEHKLYARAFSAFYD

QRPQRLDIPKFDKYGDLGKLNEDKLKSQRLTEDRLRQFLQIEPKHVEQMAELHKNLVGEL

DKVLDDFGDLTKIYKGTGIAIVAGARFMPIVTNSLRMLRRTGCTLPVEIFLANQEEYEVQ

LCEEVFPSLGAECRVLPDVLGKAALRNFPLKGYQFKILSALASSFEHVLLLDADNVAIKD

PSVVFKSDVYKKYGYILWPDYWERSTHPEFFNIAGTTLGGVRDDMKKGGDEDNEPPKLAD

LMGSMPNPSTESGQVFVNKSAKFKALLLATYYNLNGPRYYYPLFSQGMMGEGDKETFLAA

AVVLKEPVYQVPRHVQAIGRWYRDSFHGAAMLQFDPIKTYELYDKAARGEIDDQTRNKLV

EKEGMFMHTNFPKMNPRELLEKKLLKDAEGNKFRAFGPRSGLNGFLPTRDLELEIWQEAE

WMTCRDIKFANWKGADREKLCSEVADQVDFMLSTTDK

>tr|Q6C0D8|Q6C0D8_YARLI YALI0F25597p OS=Yarrowia lipolytica (strain CLIB 122 / E 150) GN=YALI0F25597g PE=4 SV=1

MDTAFAQLLNYVSGGRWFPYPEQRAGFEVPAQFWNEPEPEQQEIGYEDAFNGIDLEENRD

IHNTTPLDASAASTIEKRGHTPDAESLSDDLVVGWYGPDDPDNPQNWSTLKKTTSAAQVL

ILTFSVYVGSSIYTPGLPEIMEDFNISETAALVPMTVFVFGYGLGPVVFSPLSEHPVVGR

LWIYVITLAIFVILQVPTALSKDIGSLIVLRFLGGIFSSPALAISGATFGDIYEIAYIPF

ALAFWAIAGICGPVFGPVIGSIFAQLLNWRWTFWVLTMISGLCLAVLFFFFPETSAGNIL

YRRARRLEKLTGKEYTTAARQEWAAKKMTTVAYDILLRPILITFTEPIVFALGLYIALLY

STLYSWFEAFPIVFSQTHGFNTIESGLAYLGLIVGGVIGVGLYLPIIYSKFTRPVLKGNF

PPVAMFMKLCLIGSSIFPASLFFFAWTSNKSIHWIVPIIASGLCIIGMVFIFQTVFNYLS

GSYPKYIASVFAGNSVFRAFVAGAFPLFTHQMFDAMGPESFPVGWGTTLIGFVGILMGAI

PLVLIKWGDKLKGSSRWA

>tr|Q6C174|Q6C174_YARLI YALI0F18656p OS=Yarrowia lipolytica (strain CLIB 122 / E 150) GN=YALI0F18656g PE=4 SV=1

MALNEVPPEPTNPSPEEERMTPNERTQLLTPEDPQVTPYNLLSVRIARSFITVFFAISVI

AFLLLTLNLFVHVPSLYTRGGGFLSWFFSLIAVTTTVNSALFFSTPSVMERQIQYATIFL

LFIDLIILVATPQLRYGEVIAGLIIPVWAILTCSLSAVSDLIVQWGKSTEERRLTGRVET

RRTLGEWLKVSVNLLFNLVNLLLLIAVTLSITLFAIDAARVPVPGKRVDVDHAAFKVHIN

CYDLPGNHLLQLQDTHPHVASRRPDRPDPSKKNVTIFAEAGSTSSEVYWSWITDLYAMNE

IPRVCTWDRPGVAFSDASRGLTSAGDISDLLSEALTSFFSSEEEGGQTELPNLLIVAHGI

GGLYSRVFASKHISIVKGMILIDTLHEDLISDTLTLWRGIKLFIEGAISPLGLQSLWSLL

KGHGSWDRLVGPDMVYTTKYLGYQLKENAVAWVTRSEVRASNAVLVNLDIPVEIVSSSQM

IKKSKKWSAKQRELTRLTKENVGWKILEGGHDLWKEGKTKKELQDILRDFVDYYI

>tr|Q6C7S1|Q6C7S1_YARLI YALI0D25828p OS=Yarrowia lipolytica (strain CLIB 122 / E 150) GN=YALI0D25828g PE=3 SV=1

MEKTVTAQVSRTSHNELSFQSVNPVSISVRGLSVTVKAEEQKGGFFSRRKKKTSATEKQT

TAESISIDDKSAKKEKNKKSQKKKEKESDDVEPQARDILSNISLDIPAGSIMAILGGSGS

GKTSLLNMMASRMSGGNLTVEGETLFDGKSIEHVTHAYVIQQDILSPHLTCRETLNFAAG

LRLDKSINKVQRSELVEEVIKELNLKECADTMVGNSIHRGLSGGEKRRLSIGIQMLSNPS

VLFLDEPTTGLDANSAFDLVKTMKNLSLSGRTLIMSIHQPRSDIFFLFDHVTILSRGLQV

YSGSTKESINWFASLGYDCPRDVNPADYLIDIAAVDTRSEEDEEQSFKRINAFVDKYNEL

KIETGISDVSGTASKSSSTANLQTRLFRNSFKTALFSSAPLGREIDVQVRRTWLIMYRDK

LGIVGLTVEAILMGLICGLVFLRMKPDLAGIRSMEGAAYIAISLQGYLMLLYETYRLCAT

DLAVFDREHNEGCASVFGFLIARRMAKLFTEDLIVPIIFSVLTYFLFGFRTDGAKYFFIY

FAQILLTHHISMNFSMVCAALSRDYTIASLVANLMFTLQSMASGFFANSEHMKVYIRWTK

WITYVFYGLSALLNNQFMNFFGDCPYGNKTADDPLCKDFIGENFLESVGFPRNFLVIPTI

ALLCWLIVFFLLSWLLLTIIRVDVGVGKNLKGDSNKAEKPEAAEKVTTIASVNRNPPLTV

AVSDLKLSVTKLRAKEKPILDGINAIFRPGSISAILGPSGSGKSSLLNLMANRLNSTLTQ

KYTASGDIFLNSTSIGIGNLGALCSFVTQEDDGLLSTLTVRETLYFSAYLRLPDNLTREM

KRRRADELILKMGLKDCQDTLIGDDNVKGISGGEKRRVSICVQLLSNPDILLLDEPTSGL

DSFTAGSILQVLQTLAQGGKTVICTIHQPRSDLFGQFGSVLLLSKGGHVAYDGQAKNMVQ

YFSDLGYPCPDLTNPADHVLDLVSVNLQMQWREDEDRERVDKLLSEWHKVEKQLFNAGLL

RENSASFDPKTLMPRKPAPFQIAYFILLQRGIIALSRSPQVYIARLTQPVGIGVVLVLFF

TPLRSSYIGIFNRFGLVQQLLSLYFVGVLNNMASYPFERDVFYREQDDGLYGVLPFFAVY

TTLEIPFEVVSAMVFCIIVVLPPGFPRTADFFFAAFYLSFVVINTGESIGIVFNTLFRHT

GFALNVVSVILSVGVFMAGLLSLQMPDFFKGLNYISPLKYACQSLLVMAFKDVPFHCTPD

TGGYDAMGNCIFTNGTQVLEAYGFKNHVRVYLGVAAVCLVLYRLVSLVILKLVRLRVGLR

SLKSRDNV

>tr|Q6CAF3|Q6CAF3_YARLI YALI0D03311p OS=Yarrowia lipolytica (strain CLIB 122 / E 150) GN=YALI0D03311g PE=4 SV=1

MSLFLSLFCHLVFSSVIWSFLLLDLLFSPCLLHPIMPTNYEEKLLEKLFLLHYLNKRGTD

NWKSSLKEMLRFCCQLWKLQTPRLGVIHDGKTVEYIHNLYLEKRWKSTKLDDGTITNIFT

YDLLKNILEYGFKSGAVYSLEYPTDLSALLKHTDSENCGFVDGVDVCAVKEIVKNVPQQF

LEFAYALVHERYPHRSVTEWREIHVNTNINESRYALKDYREYFRHFSSRFPHKYKKIDTK

NTQLHDEILVNFFLTNCGPHADIPGLLAATFLHKSVAQWKKHCEAALTKRLNSLAISWYD

PDTWKHVYNLFLPRYELVQAGQAKDNPIELDDKEEEEQVETSIETYSSTNILQSALNIFE

GFLDCTHHHSTPSEVFLACFNEAMKQETSFFNREKAYGKLKKLCGQLNTEQSTSDISPTN

LERFESLVSPPSSSQMSVPESVLGEFNGSGTTKNSLNTSEETSNTSGHTSTSKYNSTAED

TSGNSSASSNRVIPMEDSPPSSPLYIIQPQTNQTSTPVAPVPTQKDAKERDRKIRHELII

SKKQRLNGGKGLFKQPQIQSRTPQSCEIHPRYPQSRDTKVQSGVQSTAAEPTPTVLRPPG

TQTIPSRGSGTQEKRFYTQHEHFSQLLGTPQGQKSLVQPTIAARGQNSFQGLSEKISQAS

RDSEARKTQDGIYKRPKFKIPSHTGHVKAARISLEHLTATAHSAVPSGAPGRPAPVKQQI

TSVETVKELFRQLQNARNGPSIRNCNYK

>tr|Q6CC52|Q6CC52_YARLI YALI0C12430p OS=Yarrowia lipolytica (strain CLIB 122 / E 150) GN=YALI0C12430g PE=4 SV=1

MNLWKLVLTAGLVMCESQAGQPTPSHPAPTLRSFEEWRIHNLLQSGQNLGSTPGSGPGHS

NGGGGGSEPEMDPFTGEIDFLFAASDDPGKTYKDRFNYASFDCGATVVKSNKDVKGAGAI

LVENKDSYLLNKCVAGSKHVIIELCQDILVDQVVVGNYEFFSSMFKDIRISVADRYPVAS

GEWRVLGDFTADNIRDLQTFDITVPQIWARYVKIEFLSHWGHEYYCPISVVRVHGTTMME

EWKRSGGDGDIVAGEKLDESLQEETAQFEKAEETLHEEKMIQENVVEKSNPSLNHTVIEK

GECTPKLSLDKVKLGLNEYCRLDEFYRKIERNNSNNNTDTTTNNQSNNSTTTPPTQESIY

KTIMKRLSLLESNATLSLQYVEEQSKVLRDLLYKIEKKQNSKIDDFLFHFNATVLQQLVM

FSQQREQKLNRAILELDLQKSQTDKNMAAISQRFAMLADDLVFQKRMSMFQGLILLVVLI

FVALTRGTGPELSRRSRLMFNTDDSEDNSDFGSFRRHLRRNFKRNISSLSPRWSLNLTPQ

SPVSDDGVDEGLGLDSDSEDEHIESPPPPRTLGSPLATPGPKKKKSRSLYDEPVQWAGDT

PEPVDYMSPAEDDDDKKDHRRDRNKEE

>tr|Q6CE78|Q6CE78_YARLI YALI0B17864p OS=Yarrowia lipolytica (strain CLIB 122 / E 150) GN=YALI0B17864g PE=4 SV=1

MFIVDYAKRAYRKLRHFRITFIVVHYFIIIGSAIFGTFLFYPAKTTKYIDALFQCACAAT

QAGLNTVDLNSLKLWQQMVLYCISMWTNPIVIHSGVVFMRLHWFEKSFKDIKNQSKLQSK

LRRTATTLARQDTLENGLFQRPPLRHQNPGVAGLNSNINNDSSDETPHKEGHNNDNPNLN

KEITFGNLPQPKRTHSVEPEDMYRSINLLSHEHPEGQSDSDDDGPALVIRGPRDRPDRDD

DEEDSPEIERAPRAISFSTQEHPRAQKSALEQFKEEMRESQRQLDAEHAIDEGESSDATQ

PTSIKREDMYYRQHPDSDSSEGDTVKKVRSNVTVHRPSPEDDDFAGTQPMNRRAFTLDGD

NTEEKNHEGAFAHFKRSLTFDRFLSNRRRGSDAASSSGATSNADLNKTMSSNYLSWSPTV

GRNSAFVDLTEEQKEELGGIEYRALKVLSKVLVAYFVGFHLMCAIMLLPWAIYMPKYRDY

VYSCGVTPTWWAFFTAQSTFNDVGFTLTPDSMGSYQEAIYVLLTMSFFIVIGNTGFPVLL

RFIIWIMFKLSPRDSSLKECTGFLLDHPRRCFTMLFPSTATWWLFATLVILNSVDLVFFI

ILDFNAPAVTEMRPGYRVVSGLFQAFSTRTAGFTSVSIAELHPSIQVSYMIMMYIAILPI

AISIRRTNVYEEQSLGIYGGGDEESEYDQAPTADEEAENEENDPEHLYKGKKKKHPSYVT

AHLRAQLGYDLWYIFLGLFIICICEGTKIKDVTDLGFTGFTVLFEVVSAYGTVGMSMGYD

DVNTSLSGKFSTISKLIIIAMMIRGRHRGLPYSVDRAIILPSDEMHRKDDAVTRSHLRRG

RTMSANSNELTETMGLPYPMRSHTMMSSGPGAGGNESYPLRGSSHSRQTSNVSDIGPRDL

DPIVPSSPMPSVDEDYLKRMEKREQEQEDNKLRGELSDNKKKLHKDEQGIEMDSYFPELK

GPGGLDLADD

>tr|Q6CEV0|Q6CEV0_YARLI YALI0B12738p OS=Yarrowia lipolytica (strain CLIB 122 / E 150) GN=YALI0B12738g PE=4 SV=1

MDTKRSLSGAPPSPRKWTQKRVKTDSLLEDSRLFRPTSPLKWHRKKAGIRPKISAPLEAA

NTKFYTPAGSPRTDAGGFAYNVELPHSFTFNHSSIFHEHLSDSDKSREEKERVFNVSVDS

FSKSMEMLKQTGTASSSRLNLSMRPLAPLALSSVPPLDIPHELMQHNRESPSDDEFHDTR

TSFRHSMVSLHGLTDINNEINDINAQIQDINQQIRGIGEVDNIVTVSAIAPVEQMVTAKM

DTTPDSVNTENILDELSSLQHEDEEDAEEGEGEDEQVVEQSSESAAAAAEEEDDSLRPLE

YDLGNGPKASMPRFPTSSTLGTLVDDSDKQKTVTAAAAANIASPRSFATSASARTLFADR

FIDPESIRDSIRVVDISVRDDDEEEDPREEEGRHEGQPHQQEQEEPETSNDDSSSALNSS

SVIASTPIELSHANLPTNLFRTHQDRSKYTESGILDNRVSLISTGDKYPLDIDNVSPVRG

VDEPSFSLRDVPIETSHRDDYVFEQMKQEEDGLEGGLAPAVSSTSRPISSVSTHSSSLAG

TASSTDSLGIHFAPPDLLSQPLGFKMVSNDMGGCSSSDDSAVHHTNEATERAIPLGNFAN

SSFAELNSGDYQFPDIEVDEPQEPQMSQVAPETPMSVHSQQFSYPPSLSYSPASMEEPIM

HPPRSYVPFVFRSRELDEKDQGLYAQNNTVFVNNAFIPFYPSPENSPQFPNINSFPEVRG

VSNKEPPKEYLELKAERDKEIARLTREFEERDKQLLAKGVGKDTSPDVFNIPATPAPLRV

RKQRKDRPISDRPLSEVPQIPKRSASRRSSKRSSLIPSSETYHTDLDAIEPLPMIDASRI

EPEVEVEEKQAEVLSPSLSQQRQASAGSTGTVIRHPLQNQFPSLPDNQMLFMTRTGQKQN

FGKIQPLKSNPGGTHLYDLEAQKENNMCRDSISLLMLFLFFFMPPLWLAMGLGYLDSVAG

EVKQSHKKAALALGGTMMVAVVVGISVGLGVGLTR

>tr|Q6CGV6|Q6CGV6_YARLI YALI0A15774p OS=Yarrowia lipolytica (strain CLIB 122 / E 150) GN=YALI0A15774g PE=4 SV=1

MSRGPELPEPAFKRFTKEIGWSSLVHSSRDIKILIAQKVLRMIAYGQSTLILVQFFRAIH

VSDVSLGYFMTLTLLGDVIISYFLTLYADQIGRRHVLLLGSFLMMVSGIVFVFSDHFVVL

LIAAIVGVISPSGDETGPFKSIEESVVAHLTPTKELSDIYAWYGLFGTVGSAVGSVSAGL

MIDGLVDHYGWTKLKVYRFMFAQYAFFAFLKLLLNWGLSDKCELRKRSEEEERRDEAIAT

GDESDPLLRPTDHHDPTPSQSPKFSVWGTPLSRRSRQVVWKLCILFALDSMGYGFMPISW

VVTYFLDRWKASESTVGTLFFFTNVLNALSSLASSSMYKRLGPIIAIVAAHFPSAMAMSF

IPLAPNMPIAMALLLFRASTAVMDVVPRQAFLSHVVSAEERTKVMGIVNVVKTLARSVGP

IFTGMFAEKNMLGFAFLITGLLEAAHDMGMLTLFWKYNRIIKH

>tr|Q6C7M8|Q6C7M8_YARLI YALI0D26818p OS=Yarrowia lipolytica (strain CLIB 122 / E 150) GN=YALI0D26818g PE=3 SV=1

MNCPTRDPDPKGVPGFNQQPPQFDNELNNNKSFNKNNSGAESGKKLSAKGLANAMGDSDL

AGGQNTRSASRPSRRSRPGHQNRGLSESSLGIQLRRSTSSLNNMSDNSSNSDDSNESTPL

TASGSADPNKKPLSTWGNMMRILSKYGKFVGPGIMVSVAYMDPGNYSTAVSAGAMYQYKL

LFIIFVSNLFAVFLQVLCVKLGSVTGLDLAENCRQHLPPWLSVSIYVMAEIAIIATDLAE

VVGTAISLNILFGLPLFMGVLVTVVDVLIVLMAYRPNGPMRIVRYFEYGVAGLVAVVVVC

FAFELAALDNLDIPLVLEGFLPSKELLETNGLYLSCGILGATVMPHSLYLGSGLVQPRLR

EYDEDNGYAIPEDARFGDDDEDLKYKPSIHAIKYAMKYSIAELVISLFTVAIFINSAILI

VAGSTLNGSPDAYDADLFSIYDMLRTILSPAAGTVFALALLASGQSAGIVCTLSGQMVSE

GFLRWSFKPWIRRLITRSIAIVPCLFVTLFVGRKGLADVLNASQVVLSLLLPMVSAPLIY

FTGSKKIMKVAANAAVVHTRPRSNSNGTLTPADRSSNGYGMVEDEASTDDESICIPEYVD

MSNSATTQFIAYLVFGMVSALNLFLIISIAMGADVPL

>tr|Q6CCT6|Q6CCT6_YARLI YALI0C06644p OS=Yarrowia lipolytica (strain CLIB 122 / E 150) GN=YALI0C06644g PE=4 SV=1

MTLLAEAGIYLILDVNSPRIGESLNRYEPWTTYHEKYLEHIFKVVEQFSHYNNTLAFFAG

NEVVNDDQSAMVSPNYIKAVVRDLKYYLANQSPRKIPVGYSAADDLKYRTSLAQYLECGD

EMSSVDFYGVNSYQWCGEQSFVSSGYDRLVDDYRDYSLPLIFSEYGCNEVKPRTFQEVRA

VYSSSMTDVFSGGLIYEFSQEPNDYGLVQIYKNHSAQVLEDFEALKKAYHDAPKAKLSDF

RAVERPQRCELVYPNINTMNPLPDTFGLDMITRGVRAPRGKYVDLHKRGTSYTIYDLEGN

VIEDTEVQQVIDLKEPLAAPPPSPPTRKAPAVPEPKDPVEPVYDEYPKKVATVEEEDDHE

VVNPRRPFERKKTSTGSYRDTAVLGWIKYLVSVMLGVVVVEVVRLM

>tr|Q6C125|Q6C125_YARLI YALI0F19800p OS=Yarrowia lipolytica (strain CLIB 122 / E 150) GN=YALI0F19800g PE=4 SV=1

MSKPGWTTVALAAALSSLATACVLTFTKCNPNFYDDVRTQKPAESASKSTSASKPFLAPS

DDEQEELILEQLARSRVFFGDDGLEVIRKSSVVIVGAGGVGSWAATMLLRSGVGNVRLVD

FDQVTLSSLNRHATASRAHVGIPKVESVKQYFSGVVPWANIEAVQDLWVPAKLSKSEQEY

TEMAERLIYVNGDKPDWVLDCIDNIDAKVDLLHFCKSRDIKVISSMGAGCKSDFTQVRIA

DISQTAEDPLSKATRVKLRKLGVYEGIPVIFSTEKPGEAKLLPLEDSEFEKKNEENETTV

GELSVLPQYRVRILPVLGSLPAIFGLSMATHVLTSIGDYASIPEYIVTQSRHKPKAYDQI

QQTLVGQLARESKRRTGENGIRIAAPFDSADVGYLVEEVYKNKSVISGECSRLSLSRWRK

EGPINLQNVVVMTKDEQKKHEELVLRDENPMTVEEVYPEWVLNQVEHRLRQEKHYSMYR

>tr|Q6C2K9|Q6C2K9_YARLI YALI0F07018p OS=Yarrowia lipolytica (strain CLIB 122 / E 150) GN=YALI0F07018g PE=4 SV=1

MPSQRWTQINQGPDRDSLELASLASSHSDGNEVHSPSSSRNLSPWDSPIGSDFGDDREPL

HERTESGHTVESFQLQQSTDVGPQQALDKQKSLTYFNCLALVMGLQVGSGIFSSPGTVDH

NAGSIGSAIIVWAVAGVLAWTGACSYTELGSTIPLNGSSQAYLNYVFGSLAGFLFAWAAL

MVLKPGSAAIIALVFGEYVVKMCIGTDTPAPFWAVTLAALGGLAFVTGLNCFSTKSSTRA

GNGFLVLKLGLLLLIFIVGIVGVAKKETRSAQISENWFNGASTKLGNYAVALIAGVWAYD

GWDNVNYVTSEMKNFRRDLPRVIHTAMPVVIFCYLLVNLAYFMVLDKEEVRSTQTVAVLL

AGRVMGRAGEIIVSLLIALSCLGALNATTFTAGRLAYASAQEGHLPKLFSRLSARNTPIY

CILMECTLAAVFIFFGGFESLVMFYGVSSYLFYFVTVASVLVLRFREPELDRPYKTFLTT

PIIFCSVALFLISYGFFEKPRQALLSLLFIGSGIPVYFLRHYGYMGVSEPRA

>tr|Q6C6D7|Q6C6D7_YARLI YALI0E10329p OS=Yarrowia lipolytica (strain CLIB 122 / E 150) GN=YALI0E10329g PE=4 SV=1

MSLTRLPQDERKRLPTLFEVLNRKSLAPVDLWSFYVYTRDQHRGVDYLDFWLDVVQHLSL

CRHYIRGLRQSILASEHTNLSISNITGQGLAEQSRESSILLETLIHDHNLLEDGDSHRLS

EYLRGENLLSDHDSSAASRLSALLGAMHVRESQISDDHAPSNRSERFMMNLEEKRASHLS

NASSNLFQSAAASPIDEEDAGEEHGMVRPGSRASMTHSLQGSSRLSLNRKSSRASLGLAA

QAQPSLQQLYDEEQTNLSMSNVSQSQIGPTLQSQLQFPSPDNSGEQPQQQPQTPQPQSAL

PQQPQPTGPNLATRRNSQLSQILPQSPNANSPLLGTQPASPLQTQLNALPQTLPQPRGPI

GVDSRASSQPLTLEIIEKLFPKRNEHGVMSDVNSPNFITRERIRESSQQILVTYFMPGAE

RELALPAPLMESVRTAIEQQGRDDPEVFDECREYVFQILEREVFPGFLATKALGNLVPLG

ALVRMVAGLVAVFGAVWTGFCLIFLNYSRTERVWVLLPFAIGWYLVIAGLFSLDPIQAVL

GFSESSLTKRVRIKEPFVRRLLLIRSSWVMLWVVIVTACFTVIFACVPGHRL

>tr|Q6CB72|Q6CB72_YARLI YALI0C21406p OS=Yarrowia lipolytica (strain CLIB 122 / E 150) GN=YALI0C21406g PE=4 SV=1

MDLDNYPPPDLSWSNIKHYLGTRISTLKPPKLTEAEKKHMNPIPALRLLNKKQWLFVGVA

LAGWTWDAFDFFSVGVVATQIAEDLNVTVKDVTWGITLVLMLRAVGAVLFGVASDRYGRK

WPFIFNNFLFVVLELGTGFVQTYQQFLGVRALFGIAMGGLYGNAAATALEDCPPEARGFI

SGLFQKGYGVGYLLCVVFARAIADTSPYGWRALFWFGACPPVLIMIFRFFLPETDTYIQS

KKNAEAEGVEKQFWQGVKTTFKSYWLMFFYLVLFMTGFNFLSHGSQDLYPTMLRAQLGFD

KDQFTITNSVASLGSIFGGMVIGHASFILGRRLTILLACILGAACIYPWAFVTGPGINAG

VFFLQFFAQGAWGVVPIHLTELSPPALRSSMIGIAYQLGNLASSGSSTIQATIGTQFPLR

DENGKIRPGVYNYSLVMAIFIACVFVFLFVVTLLGPERRHAEMIAGGAGHRSESDEKIYD

EEKGAAVNVEQVESPASTPERSASPLPTTEVNHTSETLPGSQK

>tr|Q6CGB9|Q6CGB9_YARLI YALI0A20570p OS=Yarrowia lipolytica (strain CLIB 122 / E 150) GN=YALI0A20570g PE=3 SV=1

MNAALTYLNIAVFAMAGAIAREGIEHLTLFHGSFTESGLVWANFGGCIVMGWVNATNLFE

NVEKERGVTKKQLPLFLGIGTGFCGSLTSFSTLMLEGFLYGANQNDTKLGYPNAGYGVQS

VMSIGLINFGLSFAGLKVGHHLADLVPLPPLSSKVERLLSSFIAAASIALFCIFIIFAGL

WKSWRWWTYLGLFGIPGALLRWQLSKLNGKLPLGTFSANILACIVLAVCVLLQRGKTNTS

STQLVHSFLQCQILDGIMNGFCGCLSTISTFVNEMYGMNYKRAYIYGFATSFVGFSMMVI

IVGSFAWTQSLVSPAC

>tr|Q6C4E6|Q6C4E6_YARLI YALI0E27412p OS=Yarrowia lipolytica (strain CLIB 122 / E 150) GN=YALI0E27412g PE=4 SV=1

MLTLPATLSTQTINYLVASGLVGAGVILGRYVIPRATTPAPRKLAARRLSSSSISSSEAD

SSEDEFTEEDIKQLQAHEDKARGELKMVLVVRTDLKMTKGKIAAQCAHAALACYRQSSDL

KPKTTRSWVRWGQAKVALQVKSEAELFELKRQALERGIIACVIRDAGRTQIAAGSATVLG

VGPAPKSEIDRVTGHLKLL

>tr|Q6C6X1|Q6C6X1_YARLI YALI0E05577p OS=Yarrowia lipolytica (strain CLIB 122 / E 150) GN=YALI0E05577g PE=4 SV=1

MTKTAVPRKRFLWHPADIDTYYSDDYFSDEERDFKYGRPRPTPMASLLNLNLNEYELIRF

YNNESTPLLVAATGKNEDAQGLWMANVTKLASSNNALKEICMAFATMHMGHNRKKSIYIL

KDGREHPKTAGPLTDLTRVAGYTRLEEEVLEQMFIRLTNAVKAHQLQIVNMTLESYESVL

LSSVLIYLHAMSMGPFVPLFSFDGGVDLFSLGRTIHDLTFIYSDKIEPSNPFNYSFGEDS

ENDREKLPREEDMWAIIDFVDTDVELSVAEKRRIKRSLTAELNNLIMLFHMDREALSVSH

ISAWCTFWTPSFFRLLREEHNTYALLFVCYWCGYAHMWHILFWWGDRIQEDLLNLKDHLP

ERVHHFLEWPLQSCTRFDINYVDLLNGKMRQLYI

>tr|Q6CAS6|Q6CAS6_YARLI YALI0D00319p OS=Yarrowia lipolytica (strain CLIB 122 / E 150) GN=YALI0D00319g PE=4 SV=1

MASPNHFEDKLGSDTHFDHYDDPHRHSLEERDHRPSRKSSSRRGLPGFVQQLRNAVMDSD

DEAVEEEDEEAGGILLNQPALEKATITTTTSRRRSIIADDIDDTGSQVSMDHDDDVCLPV

EEGSGGIDFEEIDDYVQQQRRGSNATRTGTALNKEEDFSTDETDARFNPHADDVHLSNLE

PMRTRDSDRSRRRRSSVGGAGIPQMSRPSVFQDRFTFFTADSEATLRGQSMKSLLEQDPS

RSTYKELFEGNSDTWWLDCYNPTDGEVKMLSKAFGVHPLTSEDIRTQESREKVEIFKSYY

FVTFNTYEQDWESEDYLDPVPVYMLVFKEGIITVHFSPFQHFANVRRRIRQLREYVNVTP

DWICYAIIDDITDSFVPVSHEIEVETEVIEESVFDARDDDFSLMLRRIGKARSKTLTMMR

LLSGKADVVRMFAKRCTEGLGEGAPKGHISLYLGDIQDHIVTMYQNLIAYEKILSRSHTN

YLAQLQVQSVDANHRVTDTLGKVTVVGTILIPMNLVTGLFGMNVRVPGQDGTNLGWFFGI

LGFLLVVVLTGTLLARWWLKAMAKKISGNFRNVGAPSVRTMRRVETIRTTGFPQHREHNS

EFSMV

>tr|Q6C0F2|Q6C0F2_YARLI YALI0F25223p OS=Yarrowia lipolytica (strain CLIB 122 / E 150) GN=YALI0F25223g PE=4 SV=1

MTSRHQLFASSAQYGSPASSQASSRINTPQPPQPYAGSSSSFGEAKPYVPEQEEYDPKSH

KQYSTSMLSQLESQNDEQMEGLSAKVQLLKDVTSKIGVEIRDSSHLLNNLEDTFENASTK

LKGTFKRMMIMADNSGIGWRVWLGLFAFIFLCFFFVWVR

>tr|Q6C1D3|Q6C1D3_YARLI YALI0F17292p OS=Yarrowia lipolytica (strain CLIB 122 / E 150) GN=YALI0F17292g PE=4 SV=1

MWRLGPRHFSTSAARAADSSTHYSILGVSVSATAAEIKTAFYQLSKVYHPDRVRNKSEEE

QKMSRLKFEEVSMAYTVLSDPAQKRDYDQQNSYRFNGGSAGTQGNPNTGSGFHSTGMFRE

RPASGFNRRADKQRTSTFQGGYRTSKMKANTASSRFSGEHREASNYDVPHFDYDKHHHQQ

CSYEEHRVRHQRQQDEEFRQARRKKNEERQYHTQGGGINVGAVAGLGATGVVLIAMMCR

>tr|Q6CD18|Q6CD18_YARLI YALI0C04499p OS=Yarrowia lipolytica (strain CLIB 122 / E 150) GN=YALI0C04499g PE=4 SV=1

MEGSVFTQIVNVASFGKLFPYPEFRPDYVIPDFAAENDPVKSHSDISTNTAVISGSTVVV

TFTKDDPENPKNWGTAKKVFVSVQILLLTFAMYVGSSIYTPAISEISNDYGISTVAAILP

LSVFVFGYGVGPIVLSPLSEHPAVGRLWIYMVCQGISICLMVPLALSPNLGAMLALRFIM

GITVSPALATGGATLGDLFEIAYLPFALAFWSISAICGPVFGPVLGGVFAQVLNWHWTIW

IWMIIAAAVFVLLFFCFPETSEHHILHKRAKRLQKLNPNLNYTTEADEEWKAMSNKEVAY

QILLRPLVIIFSEPIVFFLDAYIGLSYAILYTWFEAFPMVFSELHGFNLIQTGLTYLGLI

VGGLLAAFVYVPIVYKTFTKPVIETGEFPPVALFMKIALIGAILFPISVFMFGWTAHKNI

HWIVPTIAGMLNVLGQYFIFQTVFNYLSGSYHRFIASVFAGNGLFRSGMAGAFPLFARAM

FVKLGPGNFPVGFGCTVLGCLSALMILIPIVLIKYGERLKGGSKWAN

>tr|Q6C0J0|Q6C0J0_YARLI YALI0F24277p OS=Yarrowia lipolytica (strain CLIB 122 / E 150) GN=YALI0F24277g PE=4 SV=1

MGGMDMGGSSSGGMSHSMAMTFHSNMVDALFSDQWTPSNRGQYAGTCIFIVFFAMIYRGL

FVVKFKLDEKLIKCGKLNKQVVALNDIGRDQPYKSLRDIEDEIDEEEEQERLQKLERPDQ

PSPAPTMTTRQGPRPWRLSVDVPRAAIQTVLSGVGYLLMLITMTYNVGYFVSVLGGIFLG

ELLFARYAS

>tr|Q6C2N3|Q6C2N3_YARLI YALI0F06424p OS=Yarrowia lipolytica (strain CLIB 122 / E 150) GN=YALI0F06424g PE=4 SV=1

MSQPSSPSQPTRRRSSMGTIALGDNSGPALATMPENVQQARASKARQRAISNTSLDDRKA

PAGTSSSAVVDQDDKPLGPLAYLWTSYREYSYQNTWLNPLLTMLVVVGAYYAQGNPGPQN

PLHRFIFPSYKLAPHTELNPTDDYMYGKGKRDFTFVGFYMIFFTFFREFVMQMFLKPFAT

FCGVTKKGKVNRFMEQTYSIIYYSLAGSFGLYIMYQTPIWFFNTTAFYENFPHKTHIAMF

KVYYLLQAAFWGQQSVILCMQLEKPRKDFKELVFHHIVTIALIWCSYRFHFTWMGLCVYV

TMDVSDVFLAVSKTLNYVDHAITGPFFLVFMGVWVYTRHYLNLKILYSILTEFATVGPFE

LNWVTQQYKCTLSQRITFCLLAALQLVNAYWCFLIFRIAYRFIFHDIQKDERSDDEDEDE

DEDESKKDK

>tr|Q6C8F6|Q6C8F6_YARLI YALI0D20064p OS=Yarrowia lipolytica (strain CLIB 122 / E 150) GN=YALI0D20064g PE=4 SV=1

MSRFDIDETKSKTDDSNGSQPLYWSTWRKQVNFAFVALFTLTSFATMCQASVVWGPQTRD

LGWQFEQLNRGYAFSIAGQGLGCLLMIPVAIKFGRRPVYLFGVALSLCTSLWQAQMTSLH

ELYTVSFLEGMAASLTEACVQMTIADLFPVQSRGTYNGMYIIAVSVGNFLILVPAGAIVN

QWGWRAAYWAIGLFQMVELVASLFLFEETKPGHVSGQKLSIYPRSSSKSDEEERETLVND

MERHNSYDTERSASRGCIKCTDSFPHIPLVTVTPGWSSFFKSFYTPFVLLASPIVAFAAI

TYGFLLLALSMSSVVTSLKFAEAPYSMSPWSIGLVFISPFVGMTMGSFFGGYLSDWDIVR

RTRLNSGVYSPQMRLKLIYPGLLAVTAGLLTFGISVSRGVHWVVPTLAFGAVSFGFGSVG

SIVLTYLIDYKESLAAQSLVAVVVVRNSICMVESFSLSPWVDSVGVDNVFITAGCFSLIP

IFLALAIELFHRKS

>tr|Q6CBH2|Q6CBH2_YARLI YALI0C18777p OS=Yarrowia lipolytica (strain CLIB 122 / E 150) GN=YALI0C18777g PE=4 SV=1

MNPATSMACVQIIMSSHFSYAGSMEAQDNYDMFDIDFDRPYRRWFYLGFLVPVLWVYLAL

KGGIELWKLSRTNISPHAPTAIELHAQKLSCVGSWVTNSVCGLVFYAVCGWTLYMMVMSR

W

>tr|Q6CCX1|Q6CCX1_YARLI YALI0C05841p OS=Yarrowia lipolytica (strain CLIB 122 / E 150) GN=YALI0C05841g PE=4 SV=1

MLPLLVISAISSFTLSGLVLLLPRLSSLFQLLVTWTVLSGAAGACWFSCFCVLKYSPTLY

TPSLAILVSSAVFHLCNTTYLCAITWYRMVGTKKSRKARQEKFIQHERILSEYRDLAKTE

WDKCYAGDSTCYEDTDGVDISSMDVDCENGRFSSSMESIMSTMGSVSITTDLTPEVSPTL

PFPSFCRTPEISIFKNRDSITWDEIRNVDFLCDHVERMV

>tr|Q6CI77|Q6CI77_YARLI YALI0A00913p OS=Yarrowia lipolytica (strain CLIB 122 / E 150) GN=YALI0A00913g PE=3 SV=1

MRVEQLWKRDSDDDSDDDDNTVVQGPSTDDNGVPYNYLTIIDAGSSGSRVHIFNWLSSRE

ASSGAEKRQDTNDKNRSKQKGAKGGAELHLLKMLEVKQDPSWYKKIDGGMDSYVKDPTKV

GKKHLKKLLKHAAKAIPDDQIARTPIYVHATGGMRNLGKQDQLAIIDNVCGYISDKSNFW

LPDCRTHVRVIEGGEEGLYGWIAANYLIGALDDPSNHDHGKDHSTYGILDMGGASMEVAF

EPVKGSGEGGAATDDTSYTLHLEGNYDIHTQSFQGYGVKMAHLAYLQALASQSAQEGDKD

VKIDDPCAPKGYRRDLKLADGTKIETTGSGLVEQCEKFLKVLAKDQCRTATARSRDLDGE

EPPRGCLLDNSPLDNLDIHHFIGVSKYHDIITSPALGLDDGSKYDHDKFKHAVNDFCEDD

WDDIVEEHHDVSEKHLSNLCFRGKWLDAVLEEGFGFVGSSEKNEDEDEDEGNGGQEDVKE

DAKEVEEAPKKPKRPSRPPSAIVGFVKGSGNKKPEDKEDDKAVDGKKEEEEDKDAEGKTK

LSSMSQASVSHNDKMVYNSLDKINNVMVRDTLLGRDTTYLDPFQSADLIQGRQYSWPLGR

AVLYASAENQEMNIVGYEYNHETVPGKVEINGAEQNVEWKRPQYKRGEVPTDNKTGDKEG

SSDWHEIIEDHGGDRVWGSVAFLLILVIAVYLLLGKTRRQLIFQSVSSRIWKRRGGGPAR

GGARQPYRPIDEDIELGDVETEFEVHSDDERL

>tr|F2Z5Z1|F2Z5Z1_YARLI YALI0D04026p OS=Yarrowia lipolytica (strain CLIB 122 / E 150) GN=YALI0D04026g PE=4 SV=1

MASPKGANPVEASKPPVAVAHKQKTTAWRGLMDYLSARQITLPLKVLTYIVVMHRFAALR

PYTRKFFHLQYPKHEVDGVAAYYSRGRDDIYFVLTGILALTFLRAACMDFVLVPMARALK

ITKRKPQLRFAEQGWALIYYTSSTWIGFYLYYHSPYWLNVEELWRGYPHFELDPFFKAYY

LIQFSFWVQQIFVLNMEEKRKDHYQMFTHHIVTCALMCGSYYYYYTRVGHLILVLMDGVD

TLLASAKMLKYLRYDTMCDAMFGLFVIAWVVLRHGLYNYVTWSAYFQAPVLVAENCLRDE

DGQETCFNPALHRVFVVLLIALQIITLIWLYMIVRVIVKILKGGGAEDSRSDDEDSDEEE

GEAEGEEEEEEEEEVMEEKVSDDN

>tr|Q6C122|Q6C122_YARLI YALI0F19866p OS=Yarrowia lipolytica (strain CLIB 122 / E 150) GN=YALI0F19866g PE=4 SV=1

MPWKTLLSLVYMSRACQVHGKQHVCTMTEEKTHSLSPNDSVSSGIGEIKTTSKWRNFVDS

FKEADLHDVDTEGMTELEISQLKAANSPLQRSLKDRHVKMIAIGGSIGTGLFVGSGGALA

QGGPAALLIAYTLVGSLLFCTINSLGELAVAFPVSGAFATFSSNFIDPAWGFAMGWNYTL

NWFITFPLELVAASITVDLWREFNNSNISPAAWVAIFYVAIVSINFFGAKGYGEAEFIFS

IIKVVAVVGFILFSLILVCGGVPGNGYIGGKHFHDPGAFANSFKGVAATFINAAFAFGGT

ELVGLAAAETENPRRAIPSATKQVFWRCFIFYFLSLLFIGLIVPYNSPELLAQGSVLGSA

SPFVIAIKNAGVKGLPSVMNVVIMVAVLSVGNSAIYGCSRTVAALAAQGMAPKIFGYIDR

TGRPLVGIIACSIFGLLAFVCAAPKEVENEVFFWLLSVAGLSGTFTWGSVNLCHIRWRLA

IKKQGRELKREVTFKSRVGIWGSIYSFVFCIVILAFQFWVALFPVGGADPTPRVFFQAYL

CVPVILVFYFVYKIWKRTKIWKLADINLDVGRREFDYDALQRQLEAEKIEMATRPWYVRW

GNYWC

>tr|Q6CAS8|Q6CAS8_YARLI YALI0D00275p OS=Yarrowia lipolytica (strain CLIB 122 / E 150) GN=YALI0D00275g PE=4 SV=2

MPEEPVQGIQSAAMFVDPMTGHLPATTDTPGFLARPKGDINPQKKADKMVPLHVLWFFSH

QAIVGTGACYFALFFMGLCNCKLARGLYRIFFLACISAYYTSIYRKYGGQRPSRYVLLQT

DTFQYLLMALLFLISRRSIFKLFPFFMYSVMHVAETLRRRVLKRDSPLAHQLNDNILKFE

APIQRIVADSEILILLRILLNAILFRQGAAVCLLIYVVIFRLRVAYSPPIQESLKRQEER

IDDVVAQSWVPEPVKKHWGRFRDLVDNYEHSRLTSLHDDPDDDLVEVHSDEEGYDQNEDP

DDIRNVPKKKTVKRRADLDENDKKE

>tr|Q6CF28|Q6CF28_YARLI YALI0B10780p OS=Yarrowia lipolytica (strain CLIB 122 / E 150) GN=YALI0B10780g PE=4 SV=1

MSNYNQYGDAPGNNPYSNPAGANSYEMDAVGSNPYGMDSKTSFNPTSTNEPPAYGRTNAP

QGDMMDFFEEVDEIKKSILQYDQNIDSIESLHKRSLNEISEEQEAYTREQIESLVDETSS

LSQSLKDRIKSLQSRSTRDSTKKTQAENLKRQFMNSIQRYQTVEATFRQRYRERAERQYR

IVRPEATDAEVRSAIDDAQGEQIFSQALMTSNRRGQAQTALSEVQNRHKEIQKIEQTMSE

LAQLFHDMELLVAEQEAPVQHIDNQTQGVQTDIEQGLGHTNKAVIKARAYRKKKWWCLLI

CFIIVALAVGLGVGIPVANAANKN

>tr|Q6CHB4|Q6CHB4_YARLI YALI0A10549p OS=Yarrowia lipolytica (strain CLIB 122 / E 150) GN=YALI0A10549g PE=4 SV=2

MSSFLGLFKRSDPSANYEKELQVLQTKLAETEKRLLSRRLRLRRTKGLFTLYSLVVYIVY

VGIVVAKNQLSRDQLLSGNRLTLGVLLGPPAIYIVRYVIQAVLGRFVSFDEDRIKYLRQQ

QEGKLEELKQKTGFYSTKAIVDRYEPKKKEVKKPAQQQANKQNAPTNRRQGVPPPTANVP

RNLPPTANVPAVPVVPAPGPGPNVPAAVPAIPKPVPVSEKEVLAAKQSLDNAGVSVAPVS

TGPYQRQWFDRVLDLLVGEDESAAQNRQALICSKCHNHNGLAPFGKTAAEIRYKCPNCGE

WNGNLVPTEAVELVEEVKEENENEKEEAEEEEGDAVVPSEVKELVSEMVSEVPEVSMAN

>tr|F2Z5Z3|F2Z5Z3_YARLI YALI0A10659p OS=Yarrowia lipolytica (strain CLIB 122 / E 150) GN=YALI0A10659g PE=3 SV=1

MSDFLVDFMMGGISAAVSKTAAAPIERVKLLIQNQEEMIKQGRLSRPYAGIIDCFKRTAA

EEGVVSFWRGNTANVIRYFPTQALNFAFKDKFKKMFGFKKSEGYWMWMMGNLASGGLAGA

TSLAFVYSLDFARTRLANDAKSVAKDGKAAGERQFNGLIDVYRKTIASDGIAGLYRGFGV

SVVGIIVYRGLYFGLYDSLKPVVLVGPLEGNFLAAFLLGWTVTTGASTASYPLDTIRRRM

MMTSGTGVKYSSAFDCGVQIVKAEGVASLFRGCGANILRGVAGAGVISMYDQMQMILFGK

KF

>tr|F2Z6H3|F2Z6H3_YARLI YALI0B13816p OS=Yarrowia lipolytica (strain CLIB 122 / E 150) GN=YALI0B13816g PE=3 SV=1

MLTNLTIVLITLLVTYTVLTRTALRIQRARKAKQMGATLPPRVNNGILGWYGLWLVIQNA

RSMKLPHTLGKRFANGPTWLTPVAGNEPINTIDPENVKAILATQFKDFCLGIRHRALSPS

IGDGIFTLDGEGWTHSRALLRPQFSRQQISRVHSLERLMQILFKLIRKENGEYFDLQNLF

FMFTLDSATEFLYGASVDTLADLLGEPVEGDHGGVGEEVRKAYQQSINNAQDISAIRTRL

QGLYWIAGNIYQRNLYQKSNKGVKDFSQFFVDKALNTSKEKLKEMEDSDNYVFLYELVKS

TRNPVVIRDQLINILVAGRDTTASLLSFTFYTLGRRPDVLKKLRAAILEDFGTSPDEITF

ESLKRCDYLRYVLNEVLRLYPSVPINARSATRDTTLPRGGGPDGKQPVFVYKGQMVAYCV

YWMHRDKKYWGEDALEFNPDRWDPKVQPQNKGWEYLPFNGGPRICLGQQFALTEAGYVVT

RMLQEFDTVHCKNQKEEEHPPYALDLTMRHGEGVWVSMK

>tr|Q6C0E0|Q6C0E0_YARLI YALI0F25553p OS=Yarrowia lipolytica (strain CLIB 122 / E 150) GN=YALI0F25553g PE=3 SV=1

MILFWLHRGVFALSEYRIYIRGQVPRSRSLFLTARLTMLFSKASGTKYFGLKGKTLQRAI

GGIAGLGFFLFGYDQGVMGGLLNLKTFREQFETINTVDDTSLHTATIQGTAIAVYELGCM

VGALSTIYLGDKLGRRKVIYFGTWIIIIGAIIQTASYHLGQLIVGRVVAGIGNGLITATV

PMWQSECARPEDRGLMVMIEGCLISGGIALSYWLDFAFFFVKVGSMDWRFPVAFQILIAL

IIVAFVLEFPESPRWLVKVGREEDAREVWAALENCGPNDDYINYEIHEVKKNLAAEEQMT

SWQTFKLIFTYGERKHFHRACLAFWNQAMQQLTGINLITYYAAKIYQDSLHMDDVVSRAL

AAANGTEYFMASFIPFWSIERFGRRKLMLFGAVGQACTMGILTGTAWAADPNNKDNRAAG

IAACVFLFVFNSFFAIGWLGMTWLYPAEITSLEVRAPVSGISTASNWLFNFTVVMICPVG

FNSIQSYTYTIFAAINACMVPVIYFLYPETAGRSLEEIDEIFKESNPKTPWDVVGIAARM

PKRDHYNYDIESRGNSMGEQYPEGEPKPEHEEVPEGYTSETAGENSNSSVEYPDTTTHQ

>tr|Q6C387|Q6C387_YARLI YALI0F01771p OS=Yarrowia lipolytica (strain CLIB 122 / E 150) GN=YALI0F01771g PE=4 SV=1

MGGNGHYMGWWGHMGSPPQKGIAGYTISPFAARPFAGVVHAAIFNTFRRTKNQALFVILP

VSFFYYVWTQASEKNEWLYTKAGRHELAKALAE

>tr|Q6CAQ3|Q6CAQ3_YARLI YALI0D00847p OS=Yarrowia lipolytica (strain CLIB 122 / E 150) GN=YALI0D00847g PE=4 SV=1

MFVATNPGFVGAPVTRALVTIVVCLPVFVSLLELQPYFGLQMVPFITAYHQWWRLLTWQE

VYVNQSEIIFASLALYNLRIVERLLGSRQFLSYVLVVLMYTSIFVPLISAFIGILPFVSI

PYIPSGPTALIFSLLVQYRELVPVAYKFKFLLSSTSDSVTEVTLTDKIFLSLCVLQLTWT

KYWGSIIIAFTGWTVGVLLDLEILPGKGWRVPLFFRFDSRREAERIEAVSEEEETDTAFQ

RPLTRQIIDTFR

>tr|Q6CD41|Q6CD41_YARLI YALI0C04004p OS=Yarrowia lipolytica (strain CLIB 122 / E 150) GN=YALI0C04004g PE=4 SV=1

MFDRNQGGLAPSTRKTLGLVSVFGTALYLCLSILSWVFSGELALVPFTSSSLPPMADLGI

DSVHHYDLADYTGSARGQDRNERVLFLVPLRDAAAHLPMLFAHMDKMTYPHVLIDLAFLV

SDSKDDTMGVLLKELNTLQSNRDVSKRFGSIEIFEKDFGQAVGQGFSDRHGFEAQGPRRK

LMARARNWLLSVALRPDHSWVYWRDADVEKVPTTIMEDLMSHDKDVIVPNVWRPLPDWLG

NEQPYDLNSWKESDAGLRLADTLDEDAVIVEGYAEYATWRPHLAYLRDPFGDIHVEMELD

GIGGVSILAKAAVFRHGAHFPAFSFQKHAETEGFGKMCKRMGMSVVGLPHYTIWHIYEPS

DDDLRHMEWMAQQDELREKERVLKEAHDKVWQSGFDNVKQEWERERELILKNVELDFDVH

QKVRAKKAEEQSKAEAKVHGPEEEKVVPGVGKAALQPDAVKKVVQG

>tr|Q6CGC0|Q6CGC0_YARLI YALI0A20548p OS=Yarrowia lipolytica (strain CLIB 122 / E 150) GN=YALI0A20548g PE=4 SV=1

MHAPSRLTIVQKQHVFRNSPPSPMVICTPYYNRQTIKVGLRAVQLTLIMTVVITTGVLTM

YSDLPRDWVLDNYIDPYTSEFQLEWPNDYHNAEIWDQTLEFDTPIYLETHSHTNHSDGSL

TPSQLVDWAQAYGLTAICVTDHNNIEGGWAARNYSETLRSEGKPAPLVIPGVEFTCCRIH

MNFLGIEETIKPTENWPSDDELKSVIKKVHALGGVVIVNHLPWSESTEWGRKVPTLMHHP

TPDQLLNWGVDSFECVSDGVIDLQAIRYADRNNLMCVTATDIHNPSDTVRAVTVLDGPLT

QKNIIDKIRRKSVRSKSTFYYDPIGPTGAQIVPPHNNKRNKWAPLAMLNFGYMWDESKGM

YSFVDGFCHERKFTFRYGAAFWTLFWIVLGWVIYELIRAIVVGIAGSNSCRRRRPLQLE

>tr|B5FVE5|B5FVE5_YARLI YALI0D10274p OS=Yarrowia lipolytica (strain CLIB 122 / E 150) GN=YALI0D10274g PE=4 SV=1

MAPQLKDPWARREAWRYQTNFTRANRFKGAAPGFGIAVVAFGAYLAAEKFLFEKKDDHHH

>tr|B5RSK7|B5RSK7_YARLI YALI0F04114p OS=Yarrowia lipolytica (strain CLIB 122 / E 150) GN=YALI0F04114g PE=4 SV=1

MAAPIAPIVGSLKRRIFFDITTGLTLGFSAGALWWFGYHKPTVAAKDAWYAAREQEKNNQ

>tr|B5FVF3|B5FVF3_YARLI YALI0E23749p OS=Yarrowia lipolytica (strain CLIB 122 / E 150) GN=YALI0E23749g PE=4 SV=1

MINANPGFWNGPFRYLRWSAHNRPHLFFAFAIGIAGPVAALTLTPLRRKYLYPDHSPLPQ

SYPLPQRAREQLTGFDDE

>tr|B5FVH1|B5FVH1_YARLI YALI0E04169p OS=Yarrowia lipolytica (strain CLIB 122 / E 150) GN=YALI0E04169g PE=4 SV=1

MALPRKSMTIEPTQTQSSEAEPRLSRALSTRSLWKASAAPMKDQENPTTLDLLNYLQTNP

NSEYSIRAKFFLLNMALCHMCLPEGNLEEGIEYTSASPDEVALVAAARDLGYIMSNRYNS

TVTVRTFPDGLDDDPVDETYEILDVIEFTSGRKRMSVVVRMPNNELFIFCKGADNVIVDR

LRNSAIAHEKIDRISNQAADRKRAEAEVVLESRKSMGAATSPRKSGVDTDARTSFGIPRP

SINLGRSRDPLDSIDDWLYKKTREEEDVHEVASTARKSVQLARKRMYNEPQPRRSMDVRQ

TAHRVSMGRVSTGRVSFAYGEERLPRNMDDILTRTTPKKNSGGGFDHEDAAGDLSVEFGI

DDELVKNDAYIMEKTLNHIEEFSTEGLRTLMYAHKKLGQEEYKQWKKLYDDARTSLENRA

MKCEKVGEMIERDFELSGATAIEDKLQKGVPETIEKLRRANIKLWMLTGDKRETAINIGY

SCRLIKDYSTVVILKHDEDIVGALAQSLLKLDTGNVAHCVVVVDGQTLSIIEEDLTLMSL

FIDLGIKADSVICCRASPSQKALMVTNVRSKVKSAITLAIGDGANDIAMIQSADVGIGIT

GKEGLQAARSSDFAIAQFSYLLKLLLVHGHWNYDRTCKYVLATFYKEMLFYTTQAIFQYN

SMFTGTSMYEQWSLAMFNTLFTSLPVIVLGIFEQDLSAATLIAVPELYAKGQKNEAFNYL

VYLVWMTIAISQSLIISFMAFYIWGFSSLPDIDNTLYPLGMITFTAVVLLITLKLQMMDI

QSRTVIALGVTIISIGGWFAWNLFLSAVYHNGRVKIYYIPDGMVSRWGKDLSWWAALLVV

TIAAALVDILWKVFRVWFFPTDTDLFQELEQDDSIKKRLEEEAYEELQQGWRHGNWVADL

EAQGADAPGTPRSSRTRSTRYSNSVRSPASPRYYDEYTPYTSSSGRESSSNDHQSRTDKL

RQKLRFPRKKTSGEYEREVEEILKRRAEDERSQALRDIEMRRMA

>tr|B5FVH6|B5FVH6_YARLI YALI0E12276p OS=Yarrowia lipolytica (strain CLIB 122 / E 150) GN=YALI0E12276g PE=4 SV=1

MVLLISSTYSVPIGYKVPPFPSLYWPAGSSERLYEVSSLYYTYDIWRFTVLWTIILFCCF

HFIAGMWASLVKRDFKNGLIIMFLYIGIAGIEAIVSGTIVGLIITGVYRAGLCSVSTWIP

LVWAVVQILWMVFTSYSMMNVFL

>tr|B5RSK9|B5RSK9_YARLI YALI0F06061p OS=Yarrowia lipolytica (strain CLIB 122 / E 150) GN=YALI0F06061g PE=4 SV=1

MVELKPSSAIQRGPLNKGGWDAPHALHNDGAIDRYAHWRTFYQERFKYTRATGKSTLIFL

VAFPALIGYVAYQSEGLFEFAGKRRGESVTTRG

>tr|Q6C1F6|Q6C1F6_YARLI Alkaline phosphatase OS=Yarrowia lipolytica (strain CLIB 122 / E 150) GN=YALI0F16709g PE=3 SV=1

MDQKTDSREYLDGFKAGQKVAYERLYWRRILVLFGGLAMVLGFFGYKGYAVNDVLDLDLS

MSRADSVNDNPLVSPLSKPKKNVIFMVTDGMGPASVSLARTYRQYVQGLAYNNTLTIDKH

FIGSSRTRSSDSPVTDSAAGATAFSCGAKSYNGAISVTPDYKACGSVLEAAKRQGLKTGL

VVTTRITDATPACFGAHVAHRSQEDEIADQLLGYATPLGRSVDLLMGGGRIHFTTKGRQD

GRDLIAEAQIDGFQYIANRKEFDELDTSNATLPLLALFTDYDMPYEIDRVPEQFPSLKET

AISALEILHQETKDSDEGFFIMIEGSRIDHAGHQNDPAAQVREVMAFDEMFAAVVDFADS

LDTETIIVSTSDHETGGLAIARQVQAEYPEYVWYPEALAAAQHSGEYIARKLQAFARPDH

PSEASGSKLEKFVKREILENDLGVKDYTKQEVKALIRNRADPIDTIVDIVSRRSQTGWST

HGHSAVDVNIYAHSNKQSGLDKLDALRGNHENIEIGQFLAKYLEVDVARVTEILEDLPVR

VQHVEEIDDCDQYHHGLY

>tr|Q6C3C6|Q6C3C6_YARLI YALI0F00814p OS=Yarrowia lipolytica (strain CLIB 122 / E 150) GN=YALI0F00814g PE=4 SV=1

MPTLRRFYAIDPTPFPELNKDEKLDVRAMQRTFEGAYYRSAMGQLGFALLVLKVFSVKFM

PVGTTFTACGILILVVALRRKRITDHMMYEETLGNRIFITSGNAVIWFAAFSLASYIVML

VLLMRLD

>tr|Q6C5Z3|Q6C5Z3_YARLI YALI0E13882p OS=Yarrowia lipolytica (strain CLIB 122 / E 150) GN=YALI0E13882g PE=4 SV=1

MPSSPSQRLLGSTAASSLAVSPVGWATRAGITLPLFRKASMILPNTGQRVRRQVTFSNEE

GRLVPQTIKITPEELEALADANANANANANNFQTTYQNYNSITEFGEPNSSSSPNSPEPD

PERSFNYAVSTVDSPTMCHRHKPKPTGVFGKLKYYATGAWIDPEAKVVLKCSVGYLLGTV

FVYNDRLSRLLGNGDSKHMVATSVIYFHPGRTMGSMVEALIFASIALLYSFCVCSLSMLV

SAFLVDLHMRLLAYIIDLVIFCAVGLGTLAFVKQKMDLPTFNTATSLACVCLVSVLTKEG

NVQQGRISLEQLNQIFLLAITGCTVSALVCVTLWRTSAVTELKQSISNTMDIYGDMLLYL

TRQFMRAVDVDSVEYIELKAKLRAQLSCFRNIRHAKLELYLTGREDEYHQLEKLVHHMNS

IHQHLNALTSGVETEHSTLTRKKLSQLFKDSPIRKRHSLKRTLSTPGSSLPSPNFASTKP

NEPTITPENLFDVFLYHLGPPMKSFSLTMKRILHGSELDKCDAEADVAQYQRSLGLAEEL

FCEARKKALAEVYGLGLFKRRHNDEFITDWEQVAASCGNFSLLLEEMASEVQKLLGSIEG

YLTIRENDERSYRWLWGGKHREPTAAEEAETSQRSAVNKIISGRKTMAFMTRQSDLLEEG

FAGRMHDDLNSALTKKAKHGELEKPSMSFRLWKRLRVFRRRDVQFGIRVGIGAAMFATPA

YMPSLQPIFYTWRGEWGLISYVVIMSKSVGGTTWTAWKRIIGTFLGAMCAYVSWVLFPEN

EYMLALIGAAISYPCFRIIVTWKDNNAFGRFVLLTFNITAVYSYSLYLGDNDNDDDEGGL

RPLVGTIAFHRFVSVCAGVMWAFLVTVLLLPNKARVNLKHGLCSLWLRMGLTWKDPLASQ

FDPATGGYRLVGLAEQPQLQNTLIQLDGLLKHAPNEFRLKGRFPKNTYDNILKSTQKILD

AFQNLRLVIEKDPTTTPHEAVLLKSTLAERTELSHRIFLRFYMLASAMKLGFPFPDNLPS

TKHSRDRMMVKLNIYRGSQPEEYEQNNVMAAGSEITDNDFALLYIYILVCVTITSELEIL

AKLMQDLFGVIDEEMFDISYS

>tr|Q6CAW7|Q6CAW7_YARLI YALI0C23771p OS=Yarrowia lipolytica (strain CLIB 122 / E 150) GN=YALI0C23771g PE=4 SV=1

MRLRPLTASLDTRFATRSLHMTGVLRQFNNFDPSDELQPRIKKVIDPDNQTAGPFNNKLF

KIADEPLYSAPATFQVALAKKSAFTLGLIGTYASYLFFHYPLLPDLGGYLALGLWIPFPL

ATHYLSPYVAKISRIYRRGEPETLENMTKDETVLIETISLIGRKPMCWIIHLKDLKVVNK

RGGWANWEYTNPKTGDQHVFYVSESNGGFKMDRIWGITERNAGIDSGR

>tr|Q6CF21|Q6CF21_YARLI YALI0B10978p OS=Yarrowia lipolytica (strain CLIB 122 / E 150) GN=YALI0B10978g PE=3 SV=1

MGFQDWFAQKSRKWAALRQKPAMRLFIWSTWIPVAICFLDHAYFLGHISGNSMTPALNPD

SNLGKRDIVLLQKFLIKQPGYLKVGDVVLLRNPMDPDKFLCKRILGVGGDEIVTRHPYPQ

KTCFVPFNHVWVEGDNIHSFDSNNFGPVSLGLMHGKCPKVLWPFNRFGAIPDGGREARKE

KLGYVDDVVEG

>tr|Q6CH81|Q6CH81_YARLI YALI0A11473p OS=Yarrowia lipolytica (strain CLIB 122 / E 150) GN=YALI0A11473g PE=3 SV=1

MSTKDPYEGLSEESIAILKAQTTSEPVNVNYRTLLLRYATPLDKAEILVSYVFSACAGAC

LPLFTLIFGSMTNEFVRYFVEGATPAEFGHQINYLARYFIYLFAGIFAFSFLETYMHVQM

GEKLTGRIRAHYLEAIMRQNIGFFDKVGAGEITNRITTDTNLIQEGISEKAGLIVSSIAA

IISAFIIGFIKSWKLTLIMMSSFFALLFAMTTAVYFVVKFAKLAIVSDAKASSVAEEVLG

AIRNVVAFGTQDRLTQKYDDRLVVSMKYHIFRGRGSAAAIASVWTIAYLNYALSFWEGSR

LVSWGQVNVGNIMTVLFAVMIGAVMVGNVAPNLQAMGSAIASGQKIFETIDRVPPIDSFS

DKGQKLDQVHGHIQLEHVNFRYPSRPDVSVLHDFSLEIKPGQTVALVGASGSGKSTIIGI

LERFYEILGGKVTIDGVDISSLNVRWLRQQLALVSQEPTLFGVSIYENIAYGLIGTPHEN

ADPEKKRQLVEDAARQANAYDFIQDLTDGFETNVGDRGFLLSGGQKQRIAIARAIVREPK

ILLLDEATSALDTKSEGIVQDALDKAAADRTTIVIAHRLSTVKNADLIVVMNKGSIVEQG

THHELIEQKGMYFSLVNSQTIMKQNDDGSDTAADDKLEEDVVAIQSLTMSSFSEDEEEYN

TKEQGIIEMIRFVYSYNKEETTLLLIGGACAFVGGIGYPGMAVIFAKCIEAFMTPPSGYP

HMRSLINTYTGLFFMIAMIEMVAFYVEISILTLAGERLVRKLRLAVFKQFLRMDIGFFDR

EENTTGSLTSNLGKDAHNVRGLSGTTFGQILVSIVTVVAGFVVSVVFNWRMGLICGACIP

ILIGCGFCRYYVLTWLNNRAKLAYEQSGSYACENTNAIRTVTTLTREYQVYKTYKESVEG

QVQGSKRPIFFSSILFGLSQSLSPLIMGLAFWYGGILLKHHTISPFRFFVAFIAIVFGSQ

SAGSIFTFAPDMSKAAGSTRNIMNVLAVEPEIDWWSDQGTKIDPKDVKGNIEFQNVHFRY

PTRMQVPVLRGLNLSIKQGQYVALVGSSGCGKSTTVGLLECFYRPTSGKILLDGLDLADL

NINSYREAVALVQQEPILFSGTIKENILLGTQDPDVTDEVVYEAARKSNIHDFIMSLPEG

YDTVCGSKGSLLSGGQKQRIAIARALIRNPKILLLDEATSALDSESEKVVQAALDAAAKG

RTTIAIAHRLSTIQNADVIFVFENGVVLESGTHQQLLANRSKYYELVKLQALEG

>tr|Q6C123|Q6C123_YARLI YALI0F19844p OS=Yarrowia lipolytica (strain CLIB 122 / E 150) GN=YALI0F19844g PE=4 SV=1

MVAFVALWWLIALSLFSQSFWRVPEDTSLSGCAENWHVWKPDCGVNATGCLPPFEPLPDG

TRREMRLKCPVGCVRGSWKWGETLVGMQSVQYRPYVIGGGLEKETDVQEDEQEDYEESDL

HVGGFFKRQTKEEEATNRYYRADSSPCAAALHAGLVSPYTGGTFRLRFEGPRDSFMGSKG

AYTIDSIDFDAPFPASFSVHEDHVSGGKDNRISIVAFCVIMSFIFGYFCVNVAGFYWVQF

LVSFWVLVMVADPIVDFSRGAGPTSLAVREFWLSMAVERLLPAVLGGYIIYRAAVKNMIG

TDENPARTPGGDDGYANVESVSSGHVDGGDEDEVDENDDSDLENGDSLFKTFEKVPRSLG

TPSLSRLLLWNSGLWCGVLENYTFANLPVDRLMVSDLNARPEGWIVVIVFVIIIIAAIAS

QTYLIWRAGLFQRYIVVYISLVLLLVGLGLVGALNNLELRIHHYIIGLVLVTGASTYTHL

GLLYQGLLVGLYVNGVGRWGFDSTLESPSALARGSVVNQDIELMLVDSELTWGPPPGPGI

YTPSLMVDDVVVLSASGMDEGVDVDYIQSVVGRRPTFIRAGYVDGGYSLPLVL

>tr|Q6C276|Q6C276_YARLI YALI0F10131p OS=Yarrowia lipolytica (strain CLIB 122 / E 150) GN=YALI0F10131g PE=4 SV=1

MPPTATTAALVSRTLLARKETPSCGDREKCPMPTGSQGLPIGLGVGIPILAAILICAFLH

WRHLQKLKKEAEDDRDFDAASVYDEENKGFAIESSRQLPDKTGSKKGDQPPRTNYHQDFN

QNDPFFDSPYILKGEGYSENYSRSLTNLSAPYSGMPPPGSSSGLLPNHSGFLASLGGDRY

PSSPASSVFRAPHINSSAASLASAGADTLRKPAPTYQRQEQLPKKDVQEQDMVKQPLPPP

VPRMNISRPSLSNPNSPIEPLYSHNKQFSIGSVSDSEVDSPVTEDPFDRESNIVHTPVAS

SHGERDTDSQHQQQSSSFQSSSHTSHDSQDSFSRVMSEQSTATTPDDSEKHDKANGYFED

KDHLDHAPTVPVPAIPDQEENDMTSPPTQFDRVRSVYKVYFDENKEKAIRETALTQEGVV

ETPEGNLTMASDKELPELPGDVTADSFPADLTIDTEYSTSQMRDSHHSQSSSIYDHPVAA

PPVSAGYTAALARVPPGATAPLSTNRGYAPVNALMIDFAPTTPDLSAVPHQHASPVGSSS

PHSNWGTPPQDGYAFNHYGHGHQPPHPPHGGGYHGGAPAEAYLPQHSTSLPRRKPPPLQL

KPLQTVPTPHKLDQSDLGVTSFVPSQKRIQTPTGSAPGSPVLSQSPSVGYNPLHNPLHSM

TDTEMMPSPHALRKSVSLASLDFAPPTRFGRDSSRNNSLTSQFTSGGTPIPQQRPTQVRS

KMDTAMMLKPSWEMRN

>tr|Q6C7R3|Q6C7R3_YARLI YALI0D26081p OS=Yarrowia lipolytica (strain CLIB 122 / E 150) GN=YALI0D26081g PE=4 SV=1

MKFTLLLAVAFLVTLVTAYRDHPGDHVLLVTSTVQGATGKFNFDFKPVKGFGAAYYQSRW

DNKPFIQTRAIWYRAVLPDDEQWKRALDKDIIQIYNKRGMLNLTMDDMMEVYENGTKYAV

SNQNTSAVLHAPVAFNQTAYDEYFPTVHEFLKQLDLGTYYGAGLLSWWMLIFLVGMASNL

GKHAFFGLYQKIKGHRFRKHVSMPALWGYKHCQPAGGYLGGFISMCTPTRMQALVVLGYL

IMAFIFLFTQYNLFTPNFYWKAHGDQLARYIADRTGIMSFTQVPIVFLFGGRNNFFMWLT

GWSFDTFNIYHRWTSRVMILYAVIHSICYSYLQRDFFSVAASQFYWIMGTVATICGSLIL

FQGLHFFRSRWYETFLVIHLILAVAFIVGLWYHCVTLGWMQWVYATIAVWAFDRAARIAR

IIWSGGVVKGDFELVDSDQLIVKAEMEYSNWWKIYPGVHVYIYILNGKFWESHPFTVYQS

PYALKTGKMTVLLKAKEGKTLSLANLLAKGGGSRRLKTFIEGPYGAKHPIGKYDSSIYVA

GGIGITATYSYAVDVIQKSTAKNLIFTWVVRNDTCLTWFKTELDTLLADTRVEVNLYITG

GGGANALASTGSESDSKHSEKSTSLTNSSRLNIIYGRPDMLTEMPAYVERCPGTTAVVVC

GPPVLNDDIRLSLVHSTDCTKVDYYEEAFSW

>tr|Q6C8V6|Q6C8V6_YARLI YALI0D16665p OS=Yarrowia lipolytica (strain CLIB 122 / E 150) GN=YALI0D16665g PE=4 SV=2

MAFTSSDILKIIVAIFLPPVGVFMERGCTADLLINVLLCCLGYIPGIIHALYIIFKY

>tr|Q6CAR3|Q6CAR3_YARLI YALI0D00627p OS=Yarrowia lipolytica (strain CLIB 122 / E 150) GN=YALI0D00627g PE=4 SV=1

MKSAKLLAVVASLMASADAFPFQSKEQEPKHFNPRLARRQWFFGSNTSSVVTEPSSISET

ELYTQQAGASASVSPEDTPAPPPASSPEVPPTPEAPSAPASVVTESFHVSEVVSSYTSIE

TTVIVIPSVASSVAPVPPVASEPSVASEPPVASSPVVAVPESPSAPSLPVEPSSSEPTVS

EVGSSTETTTQLTSRQLSSSRFDGLFSSWFDDVTSGQSWLTSFNSSQTATPRPTTPGAGI

PSSNRRPSETTHDTTHQAPSRESSSPVVFVPFSSSTVAAPAPASSSSHVAGTPSSAFEPS

SAVAASSSETSTEAATTTVVVASSEISVGSTVTVGEGTSSEVVSSPPPVVVASSTDSVSD

TKTLITETTPIGEPAPTPSSSSSSSPVVVPVPPVSSGGPAVSSGGAGILSSVLPFTDTEE

SSAVPETSETVPATPTASTSIESSTGTPTAATESAQTVVEPTPVAPTSFAETTTSRPSVV

VPPVSHQSGMSQFTSVLPFTDTESETSSETSSTEVVLPVVPTSTTEETTSTLEPVEPTTT

SNPSEIPISSGAGFTSVIGWSDTETTESPTKTPTETPIKTPIETPIETPTETPTETPTAT

PTPTETPAATSELPLETSSTEVLTSAAVPSTPLEAPTQPTISSVFSGMSSVAPWSPSETE

TSSESSSSEVTAVPVIPTSIVETTSASPTEEAASASAIPSHVSSDMGFSSVEPYIPPTTS

EPTETLAPTSEVVNSAATTVDLLSSSTAEAPAGVSSPVSSPVSSGDAFSSVSPYIPEAST

ESLAVVSQESSLEVVSQVVPTTTAEEAAAASSTAAAGMLPVSSYSADEPFTSVLGSSVDA

SSRVSEITAVVQSSAASGDAAQATSEANSEANSEATSEAPAGVESSAVASSSGPVVVGTA

SPAVTSATEAAPAPSGSTSETSNWFPAGIVTQSASSTLEAGADYSSGDAVATATASATAS

GIPSSLPKIIAPSDGFPDSPKDSTLIQIGFTYALNYPFIVNNPVSVAQIFHYLPEGVGYG

LGVDAQNVSMRNIQPYDSASAGYIVSLALAYIPSDQVSTLTALLHTPTSKLYRNPDSSIQ

TLMNLIDPSIPLIPGSETISSPGSGLDAVNGNANGAKSGQAGSSGNGGDGSDKNNGNKKA

DAGGGAGGTNPYGSIDTSGATSSTPVDRKTVGIAVGAIVGAFAYGGAIFLVSKRYRKKKT

MALDSSDSGSSVRSRYFDDTESSNFDVGSVPSHSRTHHSPVNNQRSFVPRAQISQPVMSE

NSLGWI

>tr|B5FVF2|B5FVF2_YARLI YALI0E23192p OS=Yarrowia lipolytica (strain CLIB 122 / E 150) GN=YALI0E23192g PE=4 SV=1

MSNPYDLISKQDIVPMSQDKPNRWWYSYSVHSSHALMTDVEKRLFNVVVLAFAIMMALAV

VAYIPKNTAFVVRRLAYLLTGNETQHISNLWKDMLKM

>tr|Q6C0K5|Q6C0K5_YARLI YALI0F23903p OS=Yarrowia lipolytica (strain CLIB 122 / E 150) GN=YALI0F23903g PE=3 SV=1

MSLDKNRQITTSESSSGSSADEGTHIMRGLTSTSTQDETTGSISEGNLEAEKISPFVFVL

VALASISGFLFGYDTGYVSGALVVIKEDLGRALSNGDKELITASTSLGALLGGVIAGAMC

DFFGRKWVITFANILFLVGAAIQCGAHAVWTMIGGRFVMGWGVGIASLCAPLYISELAPT

RIRGRLVVLNVLAITGGQLVAYGIGAGMAHVHQGWRILVGLSMVPAFVQMVIFVFMPETP

RYLVRKNKIAEAKKVLAKTYATDDDNLLDRKLHELMLHNAYKESGLSTMARARNTMKELY

CVPSNLRALIIACGLQGIQQFCGFNSLMYFSATIFEVVGFDNATAVSIIVAGTNFVFTIV

AFMVIDRIGRRRILLGTIWGMSLGLVVNAIAFHFLDKQKEKNPNHELDKEHISGWAYVVL

VAQLVYVAFYATGIGNVPWQQSELFPISVRGVGTGMATATNWAGSLIVSSTFLTMLENIT

PTGTFSFYAGLCALGEVFVFFLYPETSGMDLEQIQQLLTGGFNIKESMRLSDEAKRGYKK

>tr|Q6C139|Q6C139_YARLI YALI0F19492p OS=Yarrowia lipolytica (strain CLIB 122 / E 150) GN=YALI0F19492g PE=4 SV=1

MPDDHRESGHDHPVEVEDLQRHYQIKLIKLHPIENAIFIAKFFIVGLYFYYTFKIDYQYY

VASYGWELAAPIGALALEWITYPVAFVLQYTQETGRQKYQYIRLAASVVAWIIRFTFYGR

YEVRREVFIVVAVAISSIILWFAKWRVALHQRAIQRSFNRAVDVDPEQQ

>tr|Q6C142|Q6C142_YARLI YALI0F19426p OS=Yarrowia lipolytica (strain CLIB 122 / E 150) GN=YALI0F19426g PE=4 SV=1

MKYDATTIAITLLFLILFQSTHVLATDVTDSSDPLWDSKYTTSQNRSLRIVSICFSSLSL

LAGTYGIFWFFYHQYKKRHLTKNAGFRSRVIKFRRKLVLALILFDFLKALMLTIYPAKKL

CGTPDSPQFAAVIGWFTNVAIEGGDMSAFLLAVQTAMMVFAPQNEHSKDNLWNRKFAKYF

QNGLYPYRKYIYVFCFTIFPTLLSSLAFVGTSYPAAQDLGFRGYVPLPTFVFLPASPTWY

RAVLSWGPRYFIMFAIFAIYIAVFIHVRREFKRLDVSMAELAGAEEGGALEDKVESSKGW

KRVRIWLSHFPGLGMLYHHHHHYPGVEEDIYGEGENEVWPGVGRQESSSDLEAQDDPLST

PPGSPTRTPDSPTEDEGPVAATPGRRAANLADLQSHINKQNLKRFRQRRADIERQMNYIF

IYPLAYLLLWTGPFVQQAMQYSKHKNEYSKFSIMIWAVVSQASNCFIDTVVFILRESNYR

KWRRERKLLKQAEERKQECTNCNEHRNKPHDDDNQSRSEENESSAGTIISLPTFGNYEMR

FGGSGGGDGPDVREAPDSRESDPLSAPPSAPPSAPPSGYQTPTSVTFPPRSTAKSPEKDN

DTVPEMMFVRSPGGVTFGVPFGRRSGDLDREQPHSQGLCQRDSHRDHHRGSISWGFGGRR

KSEFSTGDEEVDLVDFLRQ

>tr|Q6C1K1|Q6C1K1_YARLI YALI0F15609p OS=Yarrowia lipolytica (strain CLIB 122 / E 150) GN=YALI0F15609g PE=3 SV=1

MSLKMPQGQLAAPPTLYNFPDTFAFLNKLKKNDTIHKYRTSVGATIASLISTTSTFPLDS

IKTRQQTYNYRGTLHVIYDTYRHEGIKGFYRGIWAPLISTSVVRTIGASVYAATVPFSRD

FWSVHFQLPPVLHDPEHVMVGDHLDHFLLQSFKMTLLYLVPGAIAGASSTLISAPFEFTK

LSSQIEGLVARQLQAAAENKGGHINQLDGFKPHSVMETGKEILQRAGVRGLYSGYKYHLL

RDSISTGFYFTTYEIAKIVVAQKMFGKQSETMQNVAVAFAGACAGIVSWTIIFPLDTLKS

VYQKNVVAAAMAHKYGVKLDLNKDAVKINRIRDLFQRRLYKGLGISCIRTAINGAIFFSV

LEYIRKRL

>tr|Q6C1S5|Q6C1S5_YARLI YALI0F13761p OS=Yarrowia lipolytica (strain CLIB 122 / E 150) GN=YALI0F13761g PE=4 SV=1

MNKITVRRKRFLWHPDDIDAYYSDDYFSDDNERRHKLFKPRPTPMASLMNLELGEYELLR

FYKNDSSPLLVAATGKNKEAQGFWMENVTKLATSNEALKEACMVFAMMHLGHNHKHATYL

LKDGEEVPKEKLPPTDLSHVIGYTRLDEAMLEQMFIRFTNTIKAHKQQIMTMTLETSESV

LLSSVLIYLIAMSMGPYVPLFNFDGGADLFSLGRTISDLTFLFSNAPLPTPFYLNNDFDL

NDEREKLPREEDLWEIIDFVNTDEELSSTEKKRIQRILTTELKNLVGLFHMDAAGMSVSH

IAGWCTFWTPDFYKLLREEMNTYALLFVCYWCGYAHMWHVLFWWGDRIQEDLLHLMDHLP

SRVHHFLKWPLESCSRFDINYFDLLSGKMRDLYL

>tr|Q6C7A3|Q6C7A3_YARLI YALI0E02420p OS=Yarrowia lipolytica (strain CLIB 122 / E 150) GN=YALI0E02420g PE=4 SV=1

MRITRLLLLAAAAVAAVIPTPKPEGEEEAPAPCPEISGKEWETTVAEGTYWVKFYSPQCG

HCQMLAPKWERMYQEIGNDVASRHDFHIAAVNCLADGDLCNQENINVYPTLNLYKNGKKV

ETYDLRKGTQPSRLAKFVEEKIKEASGISKLEGDEEKIASTKKANVNVEGLSVDLNPTNF

KALVSDDPTGWYIKYYLPSCPHCVAMDDAWNEVAAKFKNQLNVGEINCAKYADFCRGQGI

EYYPAVTFQIGELSVTYNGERTTDALTLFGLQAVEARDMKSVNQLEFEELRQKHPETVSY

IYLHDEALFPEDLQALQKFAINVVAHANVYKSDDKKLVDKFSVTRLPALVAVNNDYTDEM

SFEVYPEYDVGHLRNSERLLQFARRTWLPLMPELTPINQDSVFADGRIVVLALVDREDKF

ATKVAIDELKQSATKYFKAKMHARQKELDLLRIQRHSERAKLLKEKDPKLQKKLEKDDTS

VPTRPPVQFAWIDADKYSKWIESRFRFNWDDRKANAPIIVIDDAKNVQFFDTDDKNARLE

PSDAEQLARVLDLIEKNNSRQLFPRSTRGFFASWFIFVWGLKTYILLVFVLLAVAKMGKR

YLRHRRSAATPTLGKWE

>tr|Q6CA52|Q6CA52_YARLI YALI0D05819p OS=Yarrowia lipolytica (strain CLIB 122 / E 150) GN=YALI0D05819g PE=4 SV=1

MSLSPSTSDVEKNSVRKPSLDSDAEVEKTPGVMLDTQIIDEATNTRILRKIDLCLMPLMM

FIYVVQFMDKSTNSLASVMGMRQDLGMKGDDYSWSGTAFYIGYLAFEFPAVFMLQKFPMA

KTLSVFVVLWGMVLCCHAVPRFGGFIALRTILGVLESAVTPAFVILTAQWYKREEQFLRT

SIWFASNGLGLILGSLVAYGLTENHHLPMHGWKLLFIITGVLTMALGIVIFFHIPDDPSK

AWFLNEEERKLVLARIASNQQGFENKTFKKEQVWEALTDIRTWLYFLFSVSNNIPNGGLT

NFSTILLSETIGLGPTRALLMQTPQGGVEFVGCIFLGWVVQRTERRCLIATIAQAIALVA

SCLLAFLPHSQGAGLCGLYLVMLYPIGLICVLSLVASNTAGHTKKISVNAILLIGYCLGN

LLGPQTFRAEDAPAYTAAKICIVVFFGLAIGITMLIWFVNVRENRRRDQLYGGDITEAEL

HELQTADLTDRQNKHFRYAF

>tr|Q6CCS5|Q6CCS5_YARLI YALI0C06930p OS=Yarrowia lipolytica (strain CLIB 122 / E 150) GN=YALI0C06930g PE=4 SV=1

MSKYRKLEDPSTSSIERDLGDVGSSHVTPGHPSVTFDMDEDDGYDELGDLGDPMNLPQVD

QIPIPKLGARGSGSRRKLQSRRNKIVGLATAGVTVLLIVLYLLLPTISGEPRKSEQGAAA

LSEAQHTGAVYKNKFHMQENWGSLSPFFSDGVSFHGVTAREKGRLDHHGFPLVPEMGVCT

LRQAHVLHRHGERYPTDGAADNMVAFADKLKSSINNQTDEMFAWVDHWNYLLERELLVPR

GYSTEFAAGAQFWSSHGRHLFNEGSLLGKWSHDPIVIRATEQERIRDSAQAWSEGFFAKN

NDLFTLSLQPEKDGENATLASYFSCKNAYNNPKSVSGKQKEAEWIDNYLNKASLRFQELI

PGFENFTAHDAFQMQQLCAFETAAFGHSKFCEFFTETEWRGYEYASDLKFYYNDMFGSKT

GVAQGAGWLSELVARLEGVLITTPAYGVDVGETSSNKTFPLDQPLYMDMSHDSVLLSVLT

ALNLDFLQESLTPDKIKVPRNVIISRLTPFGARITVEVFDCDAETEGVSSTYIRIVLNGR

ILPLSSLKQCPENADGVCEVGLFIDSVKSALAAIDYDKLCEGEL

>tr|Q6CG99|Q6CG99_YARLI YALI0A21010p OS=Yarrowia lipolytica (strain CLIB 122 / E 150) GN=YALI0A21010g PE=4 SV=1

MNAPPQQADTSTDDWAANPLAGQSIAENTKSIQYVRSVSSLAIGVGAGILHLESYYGFLF

YAIASTIVSILLYTVSSTGNPGRYFVYPVKQLFVDDIFAGLSSFLLMWTLFYELVDA

>tr|Q6CGG6|Q6CGG6_YARLI YALI0A19536p OS=Yarrowia lipolytica (strain CLIB 122 / E 150) GN=YALI0A19536g PE=3 SV=1

MSEFAHEEKFLKAKSSLNIDNIVKLADKILLNPLFAGAALALSYQNGDLYENLPRFAGIA

GAGILTTILYFFRVISRRYLKIKGWKLTSRDVAVITGGSNGLGHYIAYELSKRGVRCAIL

DREKPARTLPGSTYYYCDLTDKTVIDKAFAEVQRDMGPISILVNNAGMMCEQRVQDLNEK

LIRNLFEVNIVSHFWTLQAVIPDFLKYKRGWVVSVASTVGLIGPGHMSAYTSSKHAVVGL

HDSITHERGLAGKVGTTLVCPGQMNTRLFADLSTPTKFFAPVVESQALAKIIVDNIANGQ

RGEVIEPLYARLTPLARIVPHWLADFCRWATNLDGCMEEVEHKKVGRGEL

>tr|Q6CFX9|Q6CFX9_YARLI YALI0B02684p OS=Yarrowia lipolytica (strain CLIB 122 / E 150) GN=YALI0B02684g PE=3 SV=1

MDSYVFEVSNLHCSSCETSVKEALAPVIPPSWQVFVSVAEGKVTLVPDEGKLDSKGIRRV

VDRLLASGYDVEDMNVDEIVAKPGWFRQFKSALFDGGQKEKRHKKHCESCRDEKHKESED

SDEAPTIIGENSPATATEFKTTFAIGGMSCVSCSNQITETVKAQFPSLDTFAVDLMNKSA

VVIINNKTTATKIQECIRGMGYMCDLVEMAPVKSTKAYKVTASIGGMTCAACVNQIVAAV

QDIEGVDCVTVSLMSNSADIIIADKKWLPKVVEAIEDSGYDCDIHAVEDTESKRVEEMTR

TVNLAIDGMFCDHCPDNINNALSSYGQAMVVDDPVALDHPFVKFTYKPELPNVTIRHIIR

KVQDLSPQFTLSIVHPMTLEERSAKLQKHEQVRLFFRLALSVAVAIPTFILGIVGMSLLK

KDHPFRMYIDESAWAGSASRVTWALLILATPIYFFAADVFHIKAVKEIRNLWRPGVSWKR

RFFKFGSMDMLMSLGVNVSYWTSLALLILAAKSPNREEHGKGDEEMGYHTTFFDSVVFLT

MFLLIGRCLEAYSKARAASAISLLSDLRPKEAILVFAEDGALAKEKVDFEEGKMESDEEE

EEHTTYSGDDKVSVDYLEIGDYIRVLPGMTPPTDCIVVQGGSTFDESALTGESRPVRHGQ

GDQIFAGTVNNGSGSLIAKVMALEGGSLLDQIVNVVREGQLHRAPIERVADRLTSVFVPI

VTSLAIITWIIWLALGTTGSLPPHYLDIDLGGWVVWSLQFAISVFVVACPCGIGLAAPTA

LFVGTGLAAKYGILARGGGEAFQEGAKVDVVCFDKTGTLTEGGEPKITDEKVISYDTKEI

FFLAKLIEQQSGHPLAVAVVNHVTDMNIELPGYSVTVEEVPGKGLKGSLSVPKTSSLGQA

GVTEFLFGNERLIEENNAAISNEDAALLHRWKTEGKSVMLMALRPDGGNFALALIISAED

KIRPETPSVLKALQEQNIQTWMISGDNQITANAVAARIGIPADNVIGGVLPQEKAAKVQW

LQKTAVSARSGKSTGRAIVAMVGDGVNDAPSLSTADVGIAIGSGADIAMSSAKFVLMRSE

LTSVLTLFDLSRAIFRRIKLNFAWALVYNCIGIPVAAGVIYPYNNSRLDPVWASLAMALS

SVSVICSSLLLRLYRPPKVQFDAEVGDEGESIKSVHTNRKWKRRLAKIKNVKKLKNAGKR

LETIEVVEE

>tr|Q6BZU0|Q6BZU0_YARLI YALI0F30921p OS=Yarrowia lipolytica (strain CLIB 122 / E 150) GN=YALI0F30921g PE=4 SV=1

MQRGRQRREESEDYGAEAISNNPGLRHLTVGHAAGHSHPSSHSSSQSPTTPSFELSPPPP

EESSSADKQVRIADEEPNLELVNRRLAERRGRTRNESRSRSRSRRGSRSGERVPRQRSSL

HLGDLNVSMPISTGSYGFRPHSPFLTPQGLSGRSRPVSRSGSPTRTPAFTPGHSRHGSRV

NIKTVNSTPNMSRSASSARIQRIGSDYFQTLSTRMKRMDHTTAHTEDSELLETYGLEELR

EGYFDAIFEPARKAEENARMVVLQMREKAQAEANQVKSPIQLFLGMFVSTYHAIRTTLLT

KNGITVLKAFLAYFVGFLLCIIHPTARWLGPHCYFLPIATILHHPGRRTGSQIEITVQCI

VGQALGMGIGALALYVSDSTTTARKGHGGLLAVAVIPTIAVVSWMRACYIRLYHGFLSMG

IALLAMTDIDVSSDFHWRSAWDFGIPYLFGMLLPVVINLLIIPSIGQGDMVTCMADALSC

CRDCVHKMMATEPGVGKEAQDALNAQSIALSDIHRNMCNEIMLTTFSKDNVRIVRNLIQT

VIGHIRIVDLPCEIFSVNVAAAKEDAEEDYFNQHPHHAIPDMAPKIFELFQEGARDLVLS

IMEALTLAEEFIKNIRHPPEEISLKFQKAMTNLQHKMNHLDAQYTKLIVDQQELNISRRA

AINALFVQRLSHCGHAVFNLLRHLHGMSLETPHWRLSLPRYPWKRALTWSNTQSLHDRGG

KTAGHYFRVKAEVEEIFHRTFNMNTSRHIVREGEKNQKQQMCVNAVDHSDFDAKHGDISS

ARYKLWKILHRLQRFESKFAVKTTIAVTLLSLPGWLEGHDWYQHYDAFWAPILVLISLHP

RAGGNAHDLIVRTVVAIIGAVFGALAAGIWQGHKASPYIMGVFCAVFMIPCLYRFTVSNH

PRSGLMGCLSFTIVALGIYTDKLQGPKRWRDANPVTGTWTRALGILIGLWSAILISWILW

PFVARTESRVFTADMLGHVSQCYQIVADRYLYHDLDDDPTTLAIELSQIREARMRQGFQA

YRELILSTRHEPSLRGDFPIQTYLALFQSCEHVFNRIVEARISSVYFRIYEFDETKETTA

HLLSYRRDAIAATIMVLFIIADCYRARRPVPRYLPSSKMARKRLFQTIASLETIMLDESD

NETHVQDHEKDAQKMASILAGSRKVGEQPVFQNPFGKTDESGEKEKTEDDVSSPLAGPLH

GDSVTERKRKFWTIIHESAFATAFTDISEELEKIVGFSKSALGEET

>tr|Q6C114|Q6C114_YARLI YALI0F20108p OS=Yarrowia lipolytica (strain CLIB 122 / E 150) GN=YALI0F20108g PE=4 SV=1

MFTRLNQMLYPSSANPKFMPSDPWSFICFHVLHTVLLVFHLAYSLVHFVQYVAHRIKIRG

LAISYHHNRTPQLISHDVADLTKLPNHLAVIVDLQDGSEEGGGVEGLVAQISEIAAWCCG

TGEIKQLSVYERTGCLKSYNIKDVYKLVEEGMRSYYGSEMPSIKIDVPHSGAAHPTGESN

GKVVNRKTDNKNDLTIHLLSEEDGRECLVDLTKTLSELAIAKKLKPRDITVDVIDEQMNM

LVVTEPELLIVFGPQLDLQGFPPWQIRLTEIYHQPDNDAVTYGVFLKALQSFASCKQNVG

K

>tr|Q6C165|Q6C165_YARLI YALI0F18854p OS=Yarrowia lipolytica (strain CLIB 122 / E 150) GN=YALI0F18854g PE=3 SV=1

MTADDTDSLSSHSPLAYVVLSGGIGGATGDSVMHSLDTVKTRQQGAPHALKYRSMLRAYS

TLYLEEGFFRGLYAGFTPALLGSFPATCMFFGTYETTKRIGAYYKAPDTFVHLLGGLLGD

LVSSVWYVPSEVLKTRLQLQGRHNNPHFYSGYNYRGFNDALKTIYRKEGLGALFFGYKAT

LARDLPFSGLQFAFYEKFHQWAQDYVGHGKDMGVGLELLTGAAGGGLAGIITTPLDVVKT

RLQTQITKPTSVGGPTDTRVILSDSVLRSLATIWRTERFAGLFSGVWPRFVWTSTQSSIM

LLLYQTALKAFDVYDPFNLEDHGRQKLQ

>tr|Q6C426|Q6C426_YARLI YALI0E30327p OS=Yarrowia lipolytica (strain CLIB 122 / E 150) GN=YALI0E30327g PE=4 SV=1

MPEQAHIESDPECPHCLYPDVALKISGIFFSGLAAGGTLMTTLYLRPVFQKLPTNEAYNL

FDLIYGVGKVAFPVFSILGTASFGGAAYLERHPKGSYNQPRKDRAWYNPSSSRLLAYSAA

TLFAILPYTRIIMWPTLTKLFAVRLETVKGQNVKELLSLWSAHHLVRVFLALLSFAGGII

STVVL

>tr|Q6C8K4|Q6C8K4_YARLI YALI0D18920p OS=Yarrowia lipolytica (strain CLIB 122 / E 150) GN=YALI0D18920g PE=4 SV=1

MMSARTARVLMQSGRSARLAAPLRPLHRSLHSNPKYEYTKASFTGFLKWSAIFAGATAFA

FYTVDSRSAIHQYIALPILRLCTDAETSHRLGIEILANGMSPKQRQEDNEIDPNHLLEVT

IFANSKRPLTLRTPIGVAAGLDKHGQAIDGLFGLGFAYVEVGSVTPEPQDGNPKPRFFRL

PKDDAVINRYGFNSEGHFAVMGRLKQRLLHALGHEQPQHEDTRHSLDKNKVLAVNLGKNK

TGDEVSDYTQGVTRFANLSDALVVNVSSPNTPGLRALQGEERLAKLLTAVVAERNKVQFA

NTYTPILVKIAPDLSESEIVSIAAAAKQSKIDGIIVSNTTIQRPEHLRSQPYLSAETGGL

SGAPLKPIALKAIKTLRKNIGDEITIVGCGGISNGKDAIEFARAGATFVQVYTALAYQGP

GLPVHMKQEIVKELNGKKWVDIIGADVDVKKE

>tr|Q6C1T2|Q6C1T2_YARLI YALI0F13607p OS=Yarrowia lipolytica (strain CLIB 122 / E 150) GN=YALI0F13607g PE=4 SV=1

MVWKFLPHLNTPSETDYNNVYQSHPKYAPPVVTAAVSDTDEHSSSEKITAEDSDIVSVAL

GVTEEKELEYRDEAGRPWWKFFDEYEYRLNKQDRSGQKWFKWFDENDTPAERKLIWKLDI

LLSCYAFLIYWVKFLDQTNLNNAYVAGLSDDVHFKGNDLVNTQAIFSVGAIVFQLPFMFL

LPKVPLHIVLPLLDFCWGIMTLCQYKMNNVGGIQAMRFLVGAFEASLYPALHYLLGSWFK

PKEINRRAGFMYLAQFFGVLTSSVIAAGAVKHLDGVNGLEGWRWLFIIDGIITMPIGIIG

LWMIPGTPEKCYSIFLSKEEVVLARERLKDHKERTSGEPPKPKYSFGDRGMWKKIFSSWQ

IYVLCLEGIFCWNNNNGTSGAYLLWLKSLKNSEGEQRYTNSKVNDLGAITPGLGMVWIVL

TVGIADLFGSRWGAIVFTQLFNFTGNTILAVWDVPEGAKWFAWMLQYFGWAMAATMYSWL

NDICRHDPQVRAIILMIMNMMAQTSTAWISVLVWKTSEAPRYLKGFTFTACSAFCLMIWT

MVVLYFYKRDERQHAKENGIVLYNSSTGEGLEHVPETIIEVKPHGEKAAIKSQAVSIHSA

>tr|Q6C256|Q6C256_YARLI YALI0F10703p OS=Yarrowia lipolytica (strain CLIB 122 / E 150) GN=YALI0F10703g PE=4 SV=1

MLSELLLLASVAVAAEFKEFDATKEKVDAVIGETKAAFSEGTGTMTEKVLAFINKIFVHQ

TEYTPEAWAALSIKERLLHYDWSKEAFIVAFIVFYVGLFYLGSSLNKKKINGWVDANKAV

LASQFYQVGANPVGVKDRKILVADTPSNFTTYATGRINIDSLTAKAKLKTRQNLLTLMFE

YVFSFFMDAVPVPTDEVTITLKINKNASVPDGIWAIVHKDAMRKAREDQYFLSLTKTSDS

DKLPVSCVFMSENSELTEKLYNPALKELLTETAPYFRYLAITDLPSERPNKASEYEKSLV

GATKTVVLKLNIPSGATDLAKTAQLLETAINLVDTATKLNLRPEVAKKIRATRETEIKKI

QKVADEERAEEEAEKRAAAKKEADTKAISSLSPEAQRKAEQKLREKEARKAQKKQTRKG

>tr|Q6C852|Q6C852_YARLI YALI0D22638p OS=Yarrowia lipolytica (strain CLIB 122 / E 150) GN=YALI0D22638g PE=4 SV=1

MTALSYSQIIRLTLEAIFQVFVVCVFGYIAARCRILTPQAQKHIANLNVFLFTPCLIFSK

LASSLSLQKMIEVAIIPLLFVLMTVVSLSCANLMGWMLKLNKNQANFVKAMAVFGNSNSL

PVSLTMALSYTLPNLSWDQIPNDNPDQVASRGILYLLIFQQLGQIVRWSWGYNTLLRYAD

EEEDETNVVAVVEEDEEIVIESHDTSEQSPLLIKDTREETGILLNSDEEVSGGTASTYGS

HVTHSNDGSDSDHVSGDNTCSDNHHVTGSGHTTACHSADTSTNNSTIDLHSYQSSPAVEE

IARKPRRKNHKKKHGHKHRHRRRPHVVIRVAKAVLNFMNPPLWAMLVAIIVASVPILKYE

FFESNDIIQATITKAIQQLGSVAIPLILVVLGSNLSPDSGAPPACKNYKKMVFGAIMARM

ILPAFVLLPLIAWGVKYSEVSILDDPIFLLVSFILTIAPPAIQLSQICQLNGFYEKEMAG

VLFWGYVVLTLPTTLFIVVSSLEVLDWAGKLVS

>tr|Q6C893|Q6C893_YARLI YALI0D21604p OS=Yarrowia lipolytica (strain CLIB 122 / E 150) GN=YALI0D21604g PE=4 SV=1

MARNSGFATQTTPWNRPSSYFNSPLTTSSVRYHSSQATQNRYQVTPLLSQLPRPKRRLLV

NPVPSLKRGLCYINSSKRRTASGKLLTGARKVGTGFRVTRRYASSGTRGSAKALMSMSQR

KYFTWWFVPAISIVLWGIYQSRRNSVKDEYDEEGNLVKRAPVRPSGPWHVAVYSTLPLKA

LSRWWGSFNDITLPVWMRDPGYRFYSFVFGANLDEVAEDDLRVYQNLGEFFYRELKEGAR

PIDPDADIVCPADGKVLHLGAINARGEVEQVKGVTYSLEALLGPPTPSKDGEKSHAVSLA

APTSEIEFPDNKEENKDREFANVNGISYTLDDFMGGNASSDTTFKQEGDATTTAEPSDNA

TVAQVGKDLLQAKFNKSDDKELFFAVIYLAPGDYHRFHSPVNWVAEIRRHFVGELYSVAP

YFQKKLGNLFVLNERVALLGKWKYGFFSMTPVGATNVGSIKIHFDKDLRTNTVYEPKTES

EAAEQEKIKKKRMQKNTCYEATYGKASKLLGGYPLGKGDQMGGFNLGSTVVLVFEAPTNF

KFTIQPGQVVRVGQRIGEIGGK

>tr|Q6CGZ8|Q6CGZ8_YARLI YALI0A14608p OS=Yarrowia lipolytica (strain CLIB 122 / E 150) GN=YALI0A14608g PE=4 SV=1

MTDDRGPGHIPVDFTEPRGADNFTTPPDSPSDTSSTANRDPQQVDPQPFYTDIVEDDEYN

RYNHEEQELHRIQTNATTTSMKSIRRTGTLLGNNIPYNRAFPNRPPGRKRQKLLQYFFRP

AWLFDHMDIISFKTVIRSWIGVSLGFMLMMIPAFVRWCGSAPYLLPVVAVIVPAGSQPIT

VCFIINAIMCLFTCMGYAVMVIAMRINNTWFHNSINAEGMARQMIQEGLCAAPDSNDPNS

RATLTACVIDQVYAGKFMTARGSVVYCVLLGAFMFFMLWMKNKSRILIPGYLCGVIAVGA

LMPLAHMFPYFSPNTIGLTLVKPMGLQFAINVALSILVFPFSSGFQFTQTALSELKILEG

LVKFHENFMTSTLPSNEADWLNFSQTEADVQKARQLYPKLEAEAALLPIELTYSRFAFPD

YTALKTSVGRVISGMSGLVYFYDNVENIRRSILLVETAGRPIDMSRKLADSTGNDSRKVD

SMLKRRKKDTGRFDIDEKKKPAPVGVYEIKTGIKGAKAQYPDSLTMEELDALMIQLREMS

LPFVEQVRLALETMIKWLAAANVYRVNSLFFFWQKKGHRQRQTELAAELAERRQAFKEAA

HDFLNSKRFELFQSLEDSDEKTSFVTFYFQGALYCTYMKGVAETIDLLFTTLSNMDETES

KPSWTIGKGSSKNDKAKSTEAAPAAGENAGRGSSEEESESSETSGIWTGVERELKSYAHG

HEEHLCNNDDLEELYAQSIHGAGIDGDRKRNPDAKPPTTRVGKFGVKLNRVIKHLMSPDI

IVPLKGAIFTILCALPFYFKSTAPWWRENRLVWAVIMTGLSISENMADNLYGFGSRILYT

FYAGVIGMVAWYISTGNGDGNRAGFMVTTCILYFVLMFYREFSVHSTPMPQIVMVVTTVI

ILGMSWTDGTGVTTPSIGVGFAVAWKRFVTVVIGLSYGFICTLLPRPVTGKKIIRTTLAS

IIKDTGTLYCRISDFAVGRLAHPHKDVDLNNDPIFKSIVGAQNRIAGSEFLASMVMYEPS

LQGQWPSKVYREITTIVSELVELHHHLYIVLRRIEDPRYWFPHLLDLVGWGDQPLMSHYF

AVLYMAQGALYDGSPLPQVTPAMLMVEHLDHLEGLFKTSFDAEARHSMGESARSDFEPQP

ETNQISLERLKSNDGLIFSAAIVLSNCIFDRMDRLMFQVKTLVGEQFVNSEYYRDRTRFK

NDRLV

>tr|Q6CHN9|Q6CHN9_YARLI YALI0A06831p OS=Yarrowia lipolytica (strain CLIB 122 / E 150) GN=YALI0A06831g PE=4 SV=1

MNNPILVQYSEGCNRSSSTYCTVSVHTRFGDIPSRHNLYQSFQTPLQQSPATMSRPYRGR

GRGRGNERFGYGRERRHAYEQEYARDREISDRAGFRRDEVRDYRRDGSRGPREGPRDWPR

EPPREPRPWESRDRPPPRDPRDRDPRDRDPRDHDPRERDPRERDSRERDARDSPREFRDR

PPSGPRERHDSHSSNHSGRPPPREPRGEFHRPPERDKDSPSRPHGPKPSSGNGSRHNSSH

HSSTPTTPVVGTPKTHTNQAYQEAHFYLGSETTVSRASEPYHIKTAQTEGIQKRLTELEA

FNQALEILEAKKRKLEGEYASIETDVVREGVRAEVTGHALEVLNSQA

>tr|Q6CIA8|Q6CIA8_YARLI YALI0A00110p OS=Yarrowia lipolytica (strain CLIB 122 / E 150) GN=YALI0A00110g PE=4 SV=1

MSKHTEVFSSEKVSSMDKDDLVDTQVAEVTTSLLDFVALQLEIPPSENDGTYPLEVHFMA

EKLEETTLEEAIKIVKTNFAYHDHDNNFRDEYRIEVRELLKVFDGKDDYEKADPDDVLMM

RYWGTVFNWWSPYPEVRAVTDPYNDEDCTTETWRVWVLGTVWVAIAAFVNQFFSVRMPAI

SLGAGVIQLLLYPCGKALQYALPDWGFHFRGKRYSLNPGKWSQKEQLLTTIMVSCASGTP

YITSNIIVQYLPQFYNQTWASSFGYQFVFMLVTQMLGFGLAGILKRVAVYPVKAMWPSLL

PTLAVNKALLAPNRKENINGWLISRYTFFMIVFIGSFVYFWIPNYLMNFLQTFNWMTWIA

PENADLAIVTGSVGGLGFNPIPTFDWNQATASLAPITLPLYVSSIGFAGTFFSGLVILAL

YYTNNSWTGYIPINSNRLFDNMGKKYNVSKILTNYRFDDKKYHDYSTPYYSAGNLMLYSA

FFAVYPLSFVYTCLMDWRAMWDALKDTGKALRYVHRSSYHGREDPFSRYMKNYPEVPDWW

FYVVMVIMFALSIVLVKAWPVDTPVWTLVFVLGLVFVFIIPFTVFAAYTASSLSLNVISE

LIIGYALPGRFMALNLIKALSVTIASQAQNYTTDQKLTHYAHLPPRSIFWLQLWATLVNG

LVCLGVIQFQLNLDGICDADNKQKFTCPGETTFFTASVAWGVIGPKKMFDKYPVMKWMFL

FGALAGVFFWFVQVILPQILVKYFPNKAQTIDYYRRKILYFNPIIFVVGCLGWAPYNLTY

QVGGMYLAVLFNGYIKSRYLAWWRKYAYVMEAAIVTGIALAGIIIFFAVQYHPKDIDWWG

NTVIYDGVDGSGIPPRLEIPEKGFFGPPSSEW

>tr|Q6C1Z2|Q6C1Z2_YARLI YALI0F12177p OS=Yarrowia lipolytica (strain CLIB 122 / E 150) GN=YALI0F12177g PE=4 SV=1

MTSFFVSTFNCGKLTQDTQYYQHLSAELAGIDAPHVLLFGYQELCSIPDGCLGYAVDPLN

DLIQQVLKAIRDQPWLERYVVVGQHTFGAMGLVAIASADVLVTDVKLCHVACGMFWSSLK

GAVGLRLNVDGNPVVCVAAHLAANEGFLEQRNDDFATIAKEMKFLDPRTGIYDDATVFFL

GDLNYRASENEQGLADYKVVDELFYAMRSLDAFWDFQEAEIGFKPTYKFHTGTSEYNTKR

IPSWCDRVLYRGAVVPRKYDSIPAVMSSDHKPVYLWASVKLTQSTAVEQGDVPPHILSAA

HVSELLHIRTSIMDYIIALGLLGFTTRMGWISIAGFLLFAWALYR

>tr|Q6C2L7|Q6C2L7_YARLI YALI0F06776p OS=Yarrowia lipolytica (strain CLIB 122 / E 150) GN=YALI0F06776g PE=3 SV=1

MFSLTGKPLLYFTSVFVSLGVFLFGYDQGVMSGIITGFYFKEYFHEPTRAEIGTMVSILE

VGAFVSSLMVGRIGDIIGRRKTIMYGAFIFIIGGAFQTFAVSMSEMILGRVVAGFGVGML

STIVPVYQSEISPPHNRGKLACIEFTGNIVGYASSVWVDYFCSFINSNMSWRIPLFLQCA

MGALLFGGSFLIAETPRWLLDNDHDEEGLVVLANLHGGGDIDSPLAKQEYREIKQSVLIH

RLEGERSYTDMWKKYKKRVLIAMSSQMFAQLNGINVISYYAPLVFEEAGWVGRSAILMTG

INGIVYVCSTIPPWYLVDKWGRRPILLSGAVIMAISLASVAFWMRLDFAHTPALVVISVV

IFNAAFGYSWGPIPWLYPPEIMPLTIRAKGASLSTATNWAFNWLVGYMTPILQETIKWRL

YLMHAAFCSLSFVLVYFTYPETSGINLEDMDSLFGDKSVVNTPDSRSLLGDRDTPEPDVP

HSYTDAATDRLPAGMQGYGSAPSSRGGSVVGSPRRGNSVVGSPKRDFPQPPV

>tr|Q6C2X1|Q6C2X1_YARLI YALI0F04400p OS=Yarrowia lipolytica (strain CLIB 122 / E 150) GN=YALI0F04400g PE=4 SV=1

MSLLPQIPGLLPWWQLIVSITATLNSAQAYTGTHATARVYSNDTNKEVTRLQARTFGTWT

FLSAIIRFYGAYYLTDKHVYDLTLASYLVAFGHFMSEFLIFKTVKLSGPSIAPMIVSTTS

IVWMVLQRDWYLS

>tr|Q6CDG9|Q6CDG9_YARLI YALI0C00627p OS=Yarrowia lipolytica (strain CLIB 122 / E 150) GN=YALI0C00627g PE=4 SV=1

MRFIPTLRHVEDDLKVENTSDQTELQLEYRDETNRPWWKFFDEYEYRQNKYDRASHKWFK

WFDERDTASDKKLICKLDVLLTFYALVVYWVKYLDQTNVNNAYVSGMREDLGFEGNDLVQ

VQAMYIVGAVIFQLPFMYILPKVPLHIVLPVMDLCWGLMTLFCCKIHSVAALKVLRFFVG

VFESGFVPTAYFLMGSWYKPSEFARRAGFFYMGQFLGLLSSGLLASAALDLHGLGGFEGW

RWVFIIDAIATLPLAVIGYFMIPGTPDKCVSLFLSDDEIRRCRERMRPYVTEKKAKPSKK

LTDWTLWKPLLTSWQLPTMILITSVFYNNNSGNSGSFLLWLKSLTDSDGNQRYTNQKINQ

LSSVAPGLGFVYVYSASVFADMFKCRWGAIVITQVFNFIGCVILAIWDVTESAKWFAFCL

QYMSWSMSPVLFGWVSDIMRHDPQEYAIVVICMNMIGQSSSAWVSVLVFPTVESPRFTKG

FGTCAACAACCAIGAMVILYFYKRDEKRICYKNGIVLEGQIMEVLDADKIESEATSQADK

VLEKVSDAEMSDGKAS

>tr|Q6CDU1|Q6CDU1_YARLI YALI0B21208p OS=Yarrowia lipolytica (strain CLIB 122 / E 150) GN=YALI0B21208g PE=4 SV=1

MWWNKKEDKPADVETEQTARVAVNQQAPNTQKQTQQPQTREQEERAEREERAEKSQQAVR

DMLSYKQQDSTQRFNTKPEARILSVVIATTSFGFLSGFYTGYKRNALRFLAENSHRMPKT

VQGWYYYHKNKNYHVLSGGMALGFKYAATMTTCGIAFFGLEAYLDHARGTIDFFNTLAAT

IAAGSVYSLWYRLSKQQTFNTLRRGAAAGLALGLAQDGLRYVRGNDLWYLPSSLNHEKKH

KEEVMHA

>tr|Q6CDX4|Q6CDX4_YARLI YALI0B20482p OS=Yarrowia lipolytica (strain CLIB 122 / E 150) GN=YALI0B20482g PE=4 SV=1

MNNPNWKKFSNQLNQLQRQAQKGAGGAGGPKFVGVGGLVVLAIAAATINSSLFNVDGGSR

AIMYNRIGGISPRIYPEGTHIAIPWFQSPIIYDVRAKPRNVASLTGTKDLQMVNITCRVL

SRPSISALPTIYQTLGKDYDERVLPSLVNEVLKSVVAQFNASQLITQRERVSRLVKEQLI

KRASKFNILLDDVSLTYMTFSPEFTAAVEAKQIAQQEAQRAAFIVDRARQEKQGAIVKAQ

GEARSAELIGDAIKKSKDYVELKRLDTAREIAHVLAKSGNKIMLDNDSLLLNVANDFRSK

K

>tr|Q6CFD5|Q6CFD5_YARLI YALI0B08096p OS=Yarrowia lipolytica (strain CLIB 122 / E 150) GN=YALI0B08096g PE=4 SV=1

MLLISSPLSMLLPLLSLLTLALGQIPPESRTIVDVLAETGQFSILLRHLQRHQLIPFLNT

QRNITLVAPDNGAFAQFGGQVTRELLLYHILNGSATFENSTDGSHVYLTALDHVPVDVVV

SHKKETMAVNGVAVVETDLLAGRQRGIVQVLDSVLELPSTWQKEIGKIPRLSHFAKLANK

ARLGDLSNMTLLAPSNAAMRMFTPLMDAYLTCTDGRKDLEHIVNHFIVDELVYGSRDGKP

IPLPSREYTARDGTVYSFSDLLTVNSTWAPLQSNIPCQNGLIHVYDSLLSRDELLVFTPR

MALQGLGLDRFVALLRLAGLGELIEGKGGEQTVFAPMEKPSAMGESREFPRDSDSRSRSR

DLSYLLSHHQASLSRDEQTALVPSLLASLESDLRPSSSGLYSEGGAYHFTQSELILSRDL

FKHKAHLLVDTKMKSKKIGYRSQRIKLSYDEVADEYVINDRRIVSSPKYDTNLFVVGNSS

IYLIDEEMEPPSGLASSVAPIFHTSRSLFYLNELGRLKLPLNYAGWTVILPTTTAWDDLG

ITTEYLSSNKTALTLLFDSLIFHNPFYSDSPQTKLPTYDKSQNVSLSWVHDGKNDFVSVG

GDLYRVNTLEYDVLFNSGVVHTVENVFIPSALDVTVSNLIETTNSDFLELLQVSNLSYTL

DPALGYSFLVPSEDSMAAENISVHSPDLSDYMKLHVIQNNTLSSGGSASTLKNGVSLVAK

KFSEKLLMVSIFQGIDREARILNRGIASNNATVFAIDRPLSPSWIIPDNPWFPSHDHLRT

PVAIFIGIIIGLVAFFAILSCLLWVFLGKSREPDEERAASTPVVVTKPKPKSRTDVLRQE

AGVNTPLLAGGSGKTYATAPMSVNQPRKNGRGLKIGPQH

>tr|Q6C5L3|Q6C5L3_YARLI YALI0E17083p OS=Yarrowia lipolytica (strain CLIB 122 / E 150) GN=YALI0E17083g PE=4 SV=2

MVAIKNIAILAAATSVVVAAPAANGGFQRMEIRQLDVADDLIHIAETFLSAITGTLGVLT

NTSKGTQLQNLETALDSLNKQIPALIKDINGLNLPVISGIAGTLGSIVVNPIFKTLALIV

ETIGATLASLPVDAFNTIQNPAKIFQTLLANIGLLTDAFKKIFHNLGDALGSLDSATSSL

GSEVSSKLPPSSAQ

>tr|Q6C7Y9|Q6C7Y9_YARLI YALI0D24233p OS=Yarrowia lipolytica (strain CLIB 122 / E 150) GN=YALI0D24233g PE=4 SV=1

MPESDSTKAKGDAQHSREPSRESLQAPQHDIYSSSIDTIRPGEFRSVSPRTAPNPNSTAT

PNPGPNPGPSGSSNPGPAAVKPSPNLTALSQPSPSLNPKQPSLVLPQPRSSLSRGTSTSH

ERRYQYSVDDDEYEFEADLEREFLEGYAKAIKDQERKRRRKKSHVPGGGSISGGSRERDA

SRGHSLSHVRSRDSVPRESPSPIIKQDFNPAYSDHDADQCDVDGDHDLHDPAGSHVTHAD

GDHADSTEIADSAGLDDDDDADADDSADADDSADDNASVLSNESFTLKERQAAINTTHPF

GIRIWKPAIYKKNRSVQAQAEGDIHMTPGKTVGWRIFLGNALWTLSFGLLLYMVCGLGFI

TCSLFFWSNSARQYGRVLHKTGFYLLYPFGQFVQLAADENYLHEDEGEGRTLGEYERWQA

GDLEHGRLFFGPDSNHGHPTQGTNPTSHPNHPNHHDPSSGAHVDPVRTPLLGRNRSSLSS

ASEDSLGEDSETGVSRKRRFFGRGEWNLGRILFYTWFYVIITPVIYAVSLICWFLVFLIP

MGKVTNVLCYHLRRHPLALKFKSDSQYYELNGSGDGSGAILLCTYRAFGWKYYKYTIDGT

NIFFINLIGVVFFGIFSNYFLRLHLGLDLLITQPLFIFLVSLVSVIPLAYFIGQAVASIS

AQTSMGMGAAINAFFSTIVEVFLYCVALQQGKSALVEGSIIGSILAGVLLLPGLSMCTGA

IKRKTQRYNPKSAGVSSTMLLFAILGTFAPTFFYQIHGSYELRCDGECEGPNLMMGRNLI

SHAGSALANGQQVLAESATAAASFCSRCQFFQVPLVYDDLYQKALKPLSGICAILLFVSY

IIGLWFTLRTHAAMIWQTPIHEKKEVVQVNPNPGAGGRSSSVATPVHRATPATILDDPAG

LKHRPGTNPGHPNSGNPNPGNPNPNPGNPNPGSGPTVTLATPLVVSAPVEEAPSGHSPDA

NWSRSKSTFILLSATLLYAVVAEMLVDTVDVVLENAAIGEKLLGITIFALVPNTTEFLNA

ISFAMGGNIALSMEIGSAYALQVCLLQIPALVLYSMWNNNVDMDTAHMFTLIFPRWDLFV

VMICVFLFSYIYAEGKSNYFKGSILVLAWCVVMVGFYFDGVVSEDGFGDSPGMLSALLLP

QRMVR

>tr|Q6C0B0|Q6C0B0_YARLI YALI0F26323p OS=Yarrowia lipolytica (strain CLIB 122 / E 150) GN=YALI0F26323g PE=3 SV=1

MVSSDTKKAEPWKSLVAGSTAGAVEGLVTYPFEWSKTRLQLVDKSSTASRNPLVLIYNTA

KTQGLGAVYTGCPAFIVGNTVKAGVRFLGFDAIKGLLADKDGKVSGPRGVLAGLGAGVLE

SVVAVTPFETIKTAMIDDRQSKNPKYQGLFKGTAQLIKDKGLSGIYRGLVPVTMRQAANQ

AVRLGSYNWMKVFIQSRQKDPKAPLSSLSTFIVGAFAGIVTVYTTMPLDTVKTRMQSLEA

KKEYRGTFHCFARIFKEEGLLTFWKGATPRLGRLILSGGIVFTIYEKIMEIL

>tr|Q6C0C2|Q6C0C2_YARLI YALI0F26015p OS=Yarrowia lipolytica (strain CLIB 122 / E 150) GN=YALI0F26015g PE=4 SV=1

MFRSSLKMAPRLTRVTRMPVRNTSTSSTTYNVSGGAKGAKGPKRSWWKYLAAAFMTGGVV

GMTVSPTKVMEIALNSLPDINNESYKKMAVRDTEEQMQKLPIVKALMKDSRFRLVRGWEG

MDKTMQEHSLTGSTETMNRVGYITIPPYIFINDTDMEAVTVMHLGHHVAGYPYTVHGGVI

ATILDEALARAAFLAFPSRTGVTANLKITYKAPVRTDQFVTVCTRVKEASDKKALVTGTI

ETAEFQKPPLVEASAVFVVPKSIKLKPLSHHQK

>tr|Q6C0P2|Q6C0P2_YARLI YALI0F22957p OS=Yarrowia lipolytica (strain CLIB 122 / E 150) GN=YALI0F22957g PE=4 SV=1

MSEYRRQAHSKYKMPGGLLPPVNLGEGSGPTHANSGLRTDSKMASFVAVPDRKATSSPFR

HSGQFSNSFTSLASLKDLSDRDLPVKASPRNNNSHGTNNSNNSNNSSSNNSAKDVSIPTT

PRSSSPATAALSPVDEMNSPKSPTEAAYSSGQTPPYLRNMTMKRRNNSKSATPPPSTPGL

LGRAGDNGKKSKDEIQASPRFLDVNNSQWREAATSHNSTANLGPNSNSNSTTGMPPRSAS

SWMMSNTPIAPIALMGHAGLRNGQGGNKGGRAFDLGDVLSRDDDSDSSSSRSVSRSRSVS

HDPDERSGRASSPSCMDDWDGDNRLNMLMAITGDIPQTPMSAGGGFSDDFTNDSPGSPPM

SAGGAGWMGHQGEDVDALGILEKLDPAPTLVDYTALIDDYGDLGSQDVRKQKIKEFKRKL

QERAQQDSEPVAKSISVVDATQHMKSNLISNVGAQIGSVETRLSKKLELFQANLEALIDL

KARAKETVLAFKKETLTSYETFNSQLDHIRAIQESSEAIELLEERIGNCRAKVTEYKERL

KTVNQWIDEQERFDRLWSRRKRAAFKITVSVLVVILLISSLVLFVATLRGGFGKSLKLGF

KKAKGSTVTLSKQSQFDDVLQCLGDFDHCRFKNDMKEL

>tr|Q6C111|Q6C111_YARLI YALI0F20174p OS=Yarrowia lipolytica (strain CLIB 122 / E 150) GN=YALI0F20174g PE=3 SV=1

MTWSREEPAHTPQPTQSRRYTPTYGPAKDDYRGDSGGNRHSGHGGNRRIEIPITTIIQRP

FEHYRSHNKSPIEFSTSPSGPSDSDEDFGDLTEAEIEVLRRQISTDKVKVNFLTLFRYAT

KLDIFILFIGMVTAAAAGVCMPLFTVIFGQMTNEFLAFIVLGSSADRFQHQINHYALYFV

YIAVATFCLTSIKTYITVERGERLSARIRENYLKAIMRQNIGYFDKLGAGEVTNRITTDT

NLIQEGISEKLGLIVSAVSSFITSLVIGFIKSARLTGIMISTVVALVLAMGICSTFLVRY

VRWAIEDDSECSSIAEECFASITNIVAFGMQVKMDKRYEKPLNSSLKNYLLKARVLGAMV

GILWCITYCMYALALWEGSRLVNKGETSIGHVITVLMALMIGAFQLGGVAPNMESLGSAV

GAGKKIFETIDRVPDIDSLSGGETLSNLRGAISFKNVHFRYPSRPTVPILREFNLDIPSG

ATVALVGASGSGKSTIVALLERFYQPLGGSITVDGVSILSLDVKWLRQQMSLVSQEPTLF

NCTIFENISHGLIGTEYENAERSVKMKLVEDACEQANCSEFIKTLTDGLDTQVGEKGYLL

SGGQKQRVAIARAIISNPPILLLDEATSALDTRSEKLVQQALDKAAKNRTTIVIAHRLST

IKNADKIVVMSKGEILEQGSHDELIAARGTYYGLVGAQRIEDGGPETASTTEKGYYWESG

SGSDFDVGSNVSVEKTTPLNTWGMIKLLARFNRNERLPLLLGSGFAVICGAGYPSLALLY

GSVMQAFMVDPLAYKHMLHEIDKFSGFFFMVGMVQLGSYFMQVYYLGVASETLVRNLKRT

IFSHLLNQDLRFFDTTTTGKLTSSLSKDTQNVQGLGGATFGQILSSIVTVIISVILSCCY

TWKLGLVCSACIPLILSSGFFRFYILTQLNQRGRKVYESSAGYACEATNNIQTVMALTRE

DDVLNFYSSKVNNVVYHSAKSNAISSMLFGASQTLIILINALGFWYGSTLIRKREIDINQ

FFVAFVTVVFGVQSAGSIFSFTPDMGKAKVATQSIHEILKVKPEIGGDKESGLSLDPEKV

VGNISFDNVRFRYPERPKIPVLQGLSLSIPAGSYVALVGSSGCGKSTTISLIERFYDVLQ

GSITIDGIDIRDLNLGSYRSLISLVQQEPILFSGTIRENILLGAEGDVDDATLHSAAIQA

NIHNFVMSLPDGYDTFCGNKGTLLSGGQKQRVAIARALIRDPKILLLDEATSALDSESEK

VVQQALDTAAQGRTTIAVAHRLSTIQNADSIYVLEDGKVLEQGTHSHLMAKKGRYYELVK

LQALEG

>tr|Q6C4X1|Q6C4X1_YARLI YALI0E23045p OS=Yarrowia lipolytica (strain CLIB 122 / E 150) GN=YALI0E23045g PE=4 SV=1

MLRTLTRLRLQPLQSAAIARPLTQPLLQKRWNSTQPPKDNEQQAAPASSEGDKPKRKPLS

RISVSKGASPGEMHRRQYKGIFDFKVFALFVASGVGIYWFFQSEKAKVTQRREAEANRGY

GKPLVGGPFVLQDHKGGIFSSEDLKGKFSLLYFGFSMCPDICPDELDKMAIMIDEVNKSN

PGQLQPLFITCDPARDSPEVLEEYLSEFHPQILGLTGTYDEIKQTCKAYRVYFSTPPNVK

PGQDYLVDHSIFFYLMDPEGQFLDVLGRNLTAEEAVAKIREDIKTWEPKAVRDRKSKGWF

GSFFY

>tr|Q6C826|Q6C826_YARLI YALI0D23331p OS=Yarrowia lipolytica (strain CLIB 122 / E 150) GN=YALI0D23331g PE=4 SV=1

MSIQHTLYRPLKEGASLAILGGGIGGLLSAAFLARARPDIKITLYESKAQCGGWIKSKLL

ECHDGGSEMIEKGPRTLRMHPGTLILLQAISHVDPKFSIYGLPTDSPANTKWILNKDQLM

SLSVFKSPANLMRFLSSSVFRAMAMGAFNFLVYPWTRKRDPNVQDESVADFIGRRASPAL

GDRMGSAILHGIYAGDHKTLSARMTLRPFYGPDRLLKPAKLFTNEEFEKLYKKLFPEAST

SLFDLKKFPAMFAFPQGLSGMIDVLHKHLEKSPNVEIKCNTEITRVETTIGGMLVSDSTG

VSTTHNAVYSLLSYQAVSKLLPDTADRALIKDMSSVDVMVLNFSFEKPKDPKYTGFGYLI

PQAEENPEQVLGVIFDSDVGRGSELISGEPYKSPSGRENVTVMMGGHYWKDGNIPSSEEC

VERAKKVLKRHIGLEVTDTTEYDVELHKSCIPQYAVGHLERVDQFKHIVSKETHHRVALA

GMSFGRGVGISDAAVDAFLFVASQSHDPETLKLTRKIVDHYETTVRL

>tr|Q6CDG3|Q6CDG3_YARLI YALI0C00759p OS=Yarrowia lipolytica (strain CLIB 122 / E 150) GN=YALI0C00759g PE=4 SV=1

MGWDQLGIDDAHLAYAIIGTFTMIFSVVSLFVKEKLYIGEATVATLCGLIVGPHCLKWFT

PDTWGNTDYITLELSRVCLVIQIFAVAVELPKKYIWKHALSVFYLLFPIMAFGWLISSLF

IWALIKDLRWKEGLVMAACITATDPVLASAVVGKGRFSQRLPTHLRNLLSAESGCNDGMA

FPFTFISLNLILHYGNAGEICKEFFVITVLYECMTGICLGIVIGWGGRILIKFAESRNLI

DRESFLAFYLVLALMCAGFGTIIGVDDLLAAFAAGTAFSWDGWFAKETEESHVSNVIDLL

LNTSFFVYFGSVVPWADFNNKEIGLDVWRLVVIAVLILLFRRIPAMLILSPLVPDIRNWR

EALFCGHFGPIGVGAVYMSLIARAELESGTPSPRRKEDWPKEGQPNWLAVQTIWPVCTFL

IISSIVVHGSSLFVFFLGKHVSNLSITINSTTTAGDESHMWISRLPGIGDNGRTISISRV

DTREPGMITFGEKKRLAKQREREAQTEKHGDEANISSESTAKQRKPRRRMSKDPPVNQPL

SLGQGRGREHIDTFVEGDHVIQENDDGDIVAEYDQEKAGSSSGTDGDDQSPHITDRRHSH

PHNVAAGGHHHLHHERVKAVAYKIDDELIVENEDGDILKRYKIRQKGHGPDSPNEEFVTP

KHEGGVTKFFDRIFHKNFQDIKDIHKDKAPATDFVDLEKNEPLDVALARDGILSGVEDHP

SEGAVQGTTTILHPTHTPCNDEVCLDDPLQRATIVEGSLERQDTSRSRRSSRGSLTSFRL

GRRDSKDGTPTPGPSESDIPEETPAEKRRRMSALGLGNSAKDDDDEEEQTVVHPIREDEE

DAPRIMWGDTVRG

>tr|Q6CED5|Q6CED5_YARLI YALI0B16522p OS=Yarrowia lipolytica (strain CLIB 122 / E 150) GN=YALI0B16522g PE=4 SV=1

MEKNEVVSTNSPHEEGSVTEANNGRWANFKDSFRRADLSEYDTEGLSDLEKAALATANSP

LSRSLKGRHLQMIAIGGSIGTGLFVGSGTTLATGGPAAILIAYSIIGTMLFCTMHSLGEL

CACFPVSGAFASYSTRFIDPAWGFAMGWNYTLNWFIVFPLELVAASLTVNYWNEANGTNY

NAGCWVAIFWVLIVSINFFGVKGYGEAEFYFSLIKVIAVVGFIILAIVLTCGGGPNHDYI

GGRYWHNPGAFSAGAKGVCAVFVSAAFAFNGTELAGLAAAETANPRKSIPTATKQVFWRI

TLFYIVSLTLVGFLVPWDHPQLLNKSSSADASASPFVIAIKAAGIKGLPSVMNVVIMISV

LSVGNSSIFGFSRTIAALAQQGQAPSFFAYIDRKGRPLFGIITVSLFGLFAFICAGPIET

QNTVFYWLMAICGLSSIFTWGSINLCHFRFRMAMKVQGRSLREMPFLSHVGTIGSMYGFL

LNVTVLGLQFWIALFPVGGAKPKADVFFQAYLAVPVVLAFYIFFKLWKRPSFVALKDMDL

DTGRRETDFELLMHEMDEEAAYIASKPFYYRVYKFWC

>tr|Q6CAY7|Q6CAY7_YARLI YALI0C23298p OS=Yarrowia lipolytica (strain CLIB 122 / E 150) GN=YALI0C23298g PE=4 SV=1

MTHHDLETGVDSLSSAGMDKLMRVVTSGDNDEYIHLGGHKYHRNELAKAFGGQFNPGSAP

IPSRKFANTAPIGVFAFSIAIILLGLYLTETRGIMTPNLVVGNALFGAGLVLFVAGLWEI

VAENTFAAIVFMCFSCFWMSYAAINIPWFGIIEAYTDPGEFANAMGVYLMVWFAFAVVLT

LCTVRATIPFFLLFFFLDLALMFLAIGYFTDNYAFINAGGGFCIACGVMGFWNAWAGMAT

ADNTFTWLLPGTFMMPWAKKTD

>tr|Q6CCP4|Q6CCP4_YARLI YALI0C07722p OS=Yarrowia lipolytica (strain CLIB 122 / E 150) GN=YALI0C07722g PE=4 SV=1

MDTAFTQLLNYASRGRLFPYPEERAGFQVPTEFWYGPPTHKHDDVERGLEDAFDGIDVDG

NRGNHDERPLETSDGSSILNGGNMEKGSGSESEELYVGWYGPDDPDNPKNWSSVKKSISS

SQVLILTFSVYIGSSIYTPALPEIMADFHISQVAGLIPLSVFVFGYGIGPVVFSPLSEHP

AVGRMWIYIVTLAIFVILQVPTALCHNIGSLIILRFLGGIFSSPALATSGATFGDMYEIA

YIPFALALWAISSICGPVFGPVLGGVFAQVKGWKWTFWVLAMISGLCLVVLFFFFPETSE

GNILHRRARRLHKLTGKKYTTAARQEWASKKLSTVAYDILLRPILITFTEPVVFALGLYI

ALLYSTLYSWFEAFPIVFTETHRFNTIESGLAYLGLIVGGFVGVAMYLPIIYNKFTRPVL

KGNFPPVSMFMKLCLIGSTIFPASLFFFAWTAHKSIHWIVPIIASGLCIIGMLFIFQTVF

NYLSGSYPKYIASVFAGNGLFRAFVAGGFPLFAHQMFTTLGPSKFPVGWGTTLVGCIGVL

MGLIPLVLIKWGDKLQSSSRWA

>tr|Q6CFP0|Q6CFP0_YARLI YALI0B05258p OS=Yarrowia lipolytica (strain CLIB 122 / E 150) GN=YALI0B05258g PE=4 SV=1

MTPRTRHSTFARFVALSTFGFVFFGLFTLTFAPRTSVRRDLRRFFQGTQTEFDVKRSILL

NTKAENIRENSLKYTAEPHLAGTNKELAEWTRDQLQSFGWDAGIEEYEVYLNYPLDHALN

LVDDNGKVIYEASLEEDVLKEDPTTGLRDRVPTFHGYSANGNVTAQFVYANYGEKGDYDL

ILEKTGDPDFFKGKIVITRYGGIFRGLKVKFAQDLGAKGVVIYSDPGDDSGITPENGYKT

YPKGPARNPSSVQRGSVQFLSLGPGDPTTPGYPSKGAVERQDPSKYIPSIPSLPVSYRDI

LPILKELNGRGETFDGFEGGLEGVDYSIGPSKTSLNLYNSQDYKITPIYNVIASLTGSTY

QNEAIVVGNHRDAWIAGGAADPNSGSAVLLELARVLGVLSKAGWTPTRTLTLASWDAEEY

GLVGSTEWGEDHSKYISDNVLAYVNLDVAVSGSKFHSAGSPVLSALQRAVAKEIPHPSDN

NTSLYDVWKKDSRAYIGNLGSGSDYTVFLDHLGVPSIDVGFSGGKGDPVYQYHSNYDSFH

WMDNFVENAWEYHAAISKYVTLFAVSLSEREVVAFNVKEYALTLSKFLKAIQRKYVGASG

HLSGLETESNEQKVLEKDNSGFDTSTAPWAESFDDVLKTAKEFVETAHKYDEYTSELQKD

IIKDWPWYKYYKKLALLARIRVANRKLFHLEKLFLSSNGLQGREWFKHIVYAPGRYTGYA

GEVFPGIVEALEDGDYENAIKWLSITKASLKSAKGLLK

>tr|Q6CGR1|Q6CGR1_YARLI YALI0A17149p OS=Yarrowia lipolytica (strain CLIB 122 / E 150) GN=YALI0A17149g PE=4 SV=1

MPTTTMNIEELKHKNKDTLDKLDKHAKYVFDFKDRQAEKFFGAMSPKTRSIMTFFFSPEY

DYNSATETFDPNDEVEDLQLTHSEIVRKVWSRNQLIMAWCFLIFMSLASTIMSSSFSSYI

VYASSEFSQMGALASVQIVQSCIFLIARALCGKLADNFGRFEAMTISVVCLAVGSGLYMG

IHNIVTYFAALAFYSVGDIGIQMMYEIFAADTVTLRDRTFFQTITLIANLFTPWVSGPMV

AAVLKNSTWQWGMGMWCIIVPGCSLPFLFTLLYFRIKAYRMGLRIRRGDPNRTWFKNTVT

GIIRMDLPGLVFLCAGLILFFVPISFCTGTDNWKLGENIAMLTVGTFLLYVVFPLWEFFF

TKHPFLKYKLMYNRFVTVPGCIIGLYNMAFAIYHPYFQTWLLLAKGLPQARATNIHMAMF

VALTGTTLVFSPLMRWRAKLKSVLTWGTILYFIGMGLTYEFRRPEKPLSTMILAQVFEGV

GAGLLVIPVMVYIQVVCKQSDSAAAITIYYFFTSSGTILGNVISAAVYRNHFPKILRETG

LFSEKEIQTIFSSIYAGLGYKIGTPQRAAIGASLNTVMRSLLVGPFVLCAIMFCLALTNA

DINLNVAQRIKRASEQRKMDENEGSTEENGDAGLVVTNSFEEKSAGSSDDKKQVNEKVVQ

AV

>tr|Q6C388|Q6C388_YARLI YALI0F01738p OS=Yarrowia lipolytica (strain CLIB 122 / E 150) GN=YALI0F01738g PE=4 SV=1

MPEPDKRAAATAASQSPSDHVTRISSTATPSRPRIHTTPTPQSEVLLPQQPLSASPTKTE

SAEQADKYKTLKGRPEEAGPPPTPTIAPAAVITHLVPKHKKSKTRLKSTPKSSPRTTPTR

TTPYRSYSTTDEDPESSDEDIGPGSLLAAQQRLPIGVPDKNRHPKLKSHASAPAVPMARD

PSGGSVTNGSSVGPTITTITPGGYNSTPGASTGESLTNASSSLPASSLPGIASVSSPSLP

TTLPPSSTSNSGTSTPAVLSDPVVKPRKASAPAPASNSHITVETETVPAVPTVSVAPPPP

VQPQTDSNSNTNYQGGSVNSTVALSGGPQLGSGSNSLRLKKSQDVKSRKKKGRSQLSQQG

NTKAEIFAAKVASAVDEVHSSDSDEDFVYESNPMEKPTVNPRALGVALGPTARMARPSVS

SLSSIVHDEEDNPAVSVPDLNYDSSCSTTSDHPEFVNMTNNPRFQSRHPSLSSVGKPMGS

SERRGMSQDYSDIEKDGIPLFPHDNGLDSKGQANRTFSGPPRMPPRLNNAHSGSHLGNDA

RKRAPVSNYGSLRNAASLARLGGGSLRRLGSYTKEDPPLEPTKTRGTGGSRRGPLGGSSS

AGFTNDEEYYDFDVDADLDDEEFDDASESTPLRRGDSSRRVRKNGQFSPHNFHKSSHHAR

WRGIRRAIWIMSSLVAVLLTGFVFGFVLATNRPLQNVSVVDITELLISEQELVFDIVVKA

FNPGILSVVVPFLDIDVFAKPIVSKDDKDEKDKNILLLGNIDEFDVPLRFEGGFFTRHAQ

SAMGEVRLNDPGVPRNATLDSGVPLMLRVPDKDKGDKDKEPLTPEERWKKICSEPFELIV

RGVLKYDLPLFHKGQSTTVSKSVKVTPHKN

>tr|Q6C581|Q6C581_YARLI YALI0E20273p OS=Yarrowia lipolytica (strain CLIB 122 / E 150) GN=YALI0E20273g PE=4 SV=1

MSKARDSELGATPPSPDSPSQAPSVQEVELKEDHDTFIESLPPIVDEKKVIRKIDMRIIP

ILSLLYLMSFLDRGSIGNANIEGLSVDLGLTGAQYNMCLTVFFFTYSVFEVPSNMMLKKL

KPSIWLPTIMVAWGIVMTLMGIVQNYGGLLAARVFLGLTEAGLFPGVSYYLTLWYCRRDI

QFRTAMFFSAASVAGAFSGLLAFAIAKMDGVGGLEGWRWIFILEGIATVVIAILAFKLVV

DFPDTASFLNEQEREWVIWKLKYDTNKSKNAVQPPVLVEENHDTTWAEVRKAFKDPQLYP

QTLLYAAVLVPLYGVSLFLPTIVKNLGYTSSKAQLMTIPIYTVAAIASVLQAWVSDKFGL

RSPLLVVNFCCMFIGYMLAMNVSAVDNPSATYAGCYLVALGLYPGIPGIISWLSNNTSGA

YKRGVSMGVQIGFGNLCGAIASQIYRAEDAPQFKLGHGIEVMFICLGFICIFVLNIGYYI

VNKRKRRMIAEGQCGHYTAAELSQMGDKSPYFIYAH

>tr|Q6C6V9|Q6C6V9_YARLI YALI0E05841p OS=Yarrowia lipolytica (strain CLIB 122 / E 150) GN=YALI0E05841g PE=4 SV=1

MFHALGSIASVVFPIFASYRAIKSHDMTYATPWLKYWVVMGIEQALENTFGVVLSVLPLY

SLARLVFFAWLVLPQSQGAVRLYDEKVEPFLDRYNTQIEDFFANGHTYVRDYGLQYLSVL

VKWLTGKGFDTEAAKSTSANPAPATPVVPQSYMDSVMGYIKSNSPATGGKITRLFDMYRA

AGAVGGAVGSREVTETVEIPSSLPHSEQLGLIDKERSRLLAALSSLDARGSLLKSDTKSK

SNASLASGVNITASDYSPLLASSSDTEFDVVKSEDVLGLDGNVKDERPNSRGWFWQRHYS

KVGEANKAGYEKLVDPPSEPPVSGASMARESEEEEPLIPSNL

>tr|Q6C548|Q6C548_YARLI YALI0E21043p OS=Yarrowia lipolytica (strain CLIB 122 / E 150) GN=YALI0E21043g PE=4 SV=1

MRIAAILLCWLATLVLAKERQLSADTLVTCMQNSSFTADFFDVKFFPDNRTVDYNIRAKT

DISGRVYATLDVWAYGFMAYSVKVDPCTNDKLQQLCPMYPGNIDLESTATLSKHDVDQIP

GVGFTFPDIDLMIVGSVYMLDTDKRVACIVIQMTNGKTVEHTAVKWVTACISGLGLLVAA

FAATFGNSITASHIAANSVSLFNYFQATVILTMQAVPRVPPMAAAWAQNVAWSVGLIRIT

FMQKIFRWYIQSTGGNPTLYLTHKTISILVQRRNLFVERVTRLNPVLGKVVNHMFMATYD

AASNVYRNTDRYTRNLVSYHPSGVRARDLAPDHVMNLLSKRAQLEDISPESSSTLLVLRG

ILRVIYDAHIEQTSGTITSFTFFVLFGILLGICFGVFKLFQLCLQRKRGGEAAPNMYYGD

NSQQKEHDQDGYRHDYYASSERLLLKGALSRTLLLFCPQLLVFSLWEFYTRDSAAVIVLA

CFFLVLTLGVLCFVCFRVVYFARKSIREHNTPAYLLFGNPRILNRYGFMYLHFNANHYWY

CVLQLAYTFVKAIFIAYGQQSGKTQAMAIFIIELFYFALLCWRKPYMDKRTNALNIAISL

VTLINSFLFTFFSRIYGQPASVSSVMGVIFFILNAAFSLVLLIIVIVTCTIAILSKNPDK

RYHPAKDDRAYFMPNQDGLHGDNEFNALGNAAQEGHDLPFEEEIRDNDDSNSMLGVETKS

SDDLSMQQQLHHNNHSRHNMRDSFRDSLPSGASFDSRDAPQQETRII

>tr|Q6C4H7|Q6C4H7_YARLI YALI0E26719p OS=Yarrowia lipolytica (strain CLIB 122 / E 150) GN=YALI0E26719g PE=3 SV=1

MKLATAFTILTAVLAAPLAAPAEAFLFLLAGFAAKISASMTNETSDRPLVHFTPNKGWMN

DPNGLWYDEKDAKWHLYFQYNPNDTVWGTPLFWGHATSDDLTNWEDQPIAIAPKRNDSGA

FSGSMVVDYNNTSGFFNDTIDPRQRCVAIWTYNTPESEEQYISYSLDGGYTFTEYQKNPV

LAANSTQFRDPKVFWYEPSQKWIMTAAKSQDYKIEIYSSDDLKSWKLESAFANEGFLGYQ

YECPGLIEVPTEQDPSKSYWVMFISINPGAPAGGSFNQYFVGSFNGTHFEAFDNQSRVVD

FGKDYYALQTFFNTDPTYGSALGIAWASNWEYSAFVPTNPWRSSMSLVRKFSLNTEYQAN

PETELINLKAEPILNISNAGPWSRFATNTTLTKANSYNVDLSNSTGTLEFELVYAVNTTQ

TISKSVFADLSLWFKGLEDPEEYLRMGFEVSASSFFLDRGNSKVKFVKENPYFTNRMSVN

NQPFKSENDLSYYKVYGLLDQNILELYFNDGDVVSTNTYFMTTGNALGSVNMTTGVDNLF

YIDKFQVREVK

>tr|Q6C5M4|Q6C5M4_YARLI YALI0E16797p OS=Yarrowia lipolytica (strain CLIB 122 / E 150) GN=YALI0E16797g PE=4 SV=1

MTQPVNRKATVERVEPAVEVADSESEAKTDVHVHHHHHHHKRKSVKGKILNFFTRSRRIT

FVLGAVVGVIAAGYYAAPPELSIDIDALLGDLPSFDFDALSLDNLSMDSVSDFVQDMKSR

FPTKILQEAAKIEKHQKSEQKAAPFAVGKAMKSEGLNAKYPVVLVPGVISTGLESWSLEG

TEECPTESHFRKRMWGSWYMIRVMLLDKYCWLQNLMLDTETGLDPPHFKLRAAQGFASAD

FFMAGYWLWNKLLENLAVIGYDTDTMSAAAYDWRLSYPDLEHRDGYFSKLKASIEETKRM

TGEKTVLTGHSMGSQVIFYFMKWAEAEGYGGGGPNWVNDHIESFVDISGSMLGTPKTLVA

LLSGEMKDTVQLNAMAVYGLEQFFSRRERADLLRTWGGIASMIPKGGKAIWGDHSGAPDD

EPGQNVTFGNFIKFKESLTEYSAKNLTMDETVDFLYSQSPEWFVNRTEGAYSFGIAKTRK

QVEQNEKRPSTWSNPLEAALPNAPDLKIYCFYGVGKDTERAYYYQDEPNPEQTNLNVSIA

GNDPDGVLMGQGDGTVSLVTHTMCHRWKDENSKFNPGNAQVKVVEMLHQPDRLDIRGGAQ

TAEHVDILGRSELNEMVLKVASGKGNEIEERVISNIDEWVWKIDLGSN

>tr|Q6C404|Q6C404_YARLI YALI0E30855p OS=Yarrowia lipolytica (strain CLIB 122 / E 150) GN=YALI0E30855g PE=4 SV=1

MTLVKYLLYTYIASFVLFALIRLTTGISIKRLGYLCVRGISFSPKAGVQINIGKVGFLLH

RPSFARPGWINFYVSGLDISIDPVELEESKSHKSKKSKSATSCTYENKEWTLTPPDNAAT

RVARSFLTITRFIDFAIYSTTVDVKDVSTFILAQMVFQLDLRSHIASRRHTVTGTLYENL

KLDGELPAFFTIIFRELLVKPSGDDYDDEQTEEILDSVLLEVRGFLKKKDLSLKRLAVNF

KLGRINVLVDKLMEIKDAIKKVKKHEPEPEVVEEEKPHVAEADAKCAEEAAQVKANFATL

LIRTIREVEVHVAQFGVYHIPASLFNDSPTDKLSFAIMAKDFNLDLRRLNPKSPAFRMYF

ESDDTAHQTIFSVISVVVGLSVNKDVPEELVYVPMVTLNSKTNMFSRTLSILSDSGSATT

SEAETSDTESDAAAPIYSGQLITGALAISSPTVDLQTHHVPLLLTALNRSSKSKPPKVSD

EHFFMKGLKPATNLKVTIEEPAARVLVTRASKDHKARKMVISSCEAIVCNLHSSHENLHD

NDLETKYKLQTSLKVVGYSTWYRSPEGSRHDILDSQILQISINADMAPKLLMTVDGQLLK

ARFDMTEAEVVRGLREVVQNVRRNDVADQELHAEAEAKAKEEIACGHDLEDDEEDPCTYD

PTLPKKPNALRKLPCWLQNVNFESSESAIAVGLLFHDDTYKDVSTLMVIGVEKCVLDYKN

GVTQDGVSNEAARQYALEHSDQVVDETDDRRFVVSVKNVLAKESISSSAYEDEDDKKFDN

REILNLPSLDMAVFTTSNKKGPYTQTTVMVPKVLMQYSAHVHWFVTVHLGLLLSIFHEEL

GTGKHSIEEDEDVNLVEPDTNMSEALEALQLRKKTNKFKKLIRERRMRKKVVKGPKEEVT

VNLAIAMMRIKWHLPHDFWAMLELDTLTLDGRDLKKPIILAKYLRLYGTSPTVEDYWNRI

LIFRGLRVRADVPLIKETLASTERVHEDPLPCDERDDAVTAFIDGMQLCIPNQFICYTIL

DNILSGVKSIAHISQRLRLNTSDVVVWPKVMKPRAIPKVRIHTPRLLVIVEDDPFESQLN

LIFQVGYKEQQSRLEKEKAFEEYSDRIMASRLPKKRSTRVATEDEIGSHKEGGHHHNLAS

IGTGLKDVFHKKHKNDSSDPQNSSSGGYKDTTTVSIEQAEEELRRNFATSWIINFNAASD

ERKLAVSDLYGTVWGDEQIAEDTAKNENILEYSDNPPLMSMYHKDVDWVIDRVQFPIAQI

PDFLYDVGKGQPKDTVYTIMLPFFWNVQMGEWRINLRDYPLPLVHFPPLHPSQDASGPAV

SFHGTFIVGEMLSEGIEYVRRIKVPLAPFVHPDEIAPSTFMMEVFRTVTSVKVFTNLTFE

GHSISPTRINWGSSMSPAISAGFGAIDTFSKPPADHSPKPGFWDKARLIAHGRFKLKWDK

SDIHFVMKSSKNPYAVLGDDAGLVWVWRNHVVWDINGEDNPKEFMRVDSEEFMLAVPNLG

LSEREYLLRAFNAKDYGSQGQFLKVIMKVVGKVRWLAGVAFERDDPEHPGERTDKFKPHY

EVITKNSAEINDPHWDAFRGFRSEYIHLALSVRNPQGRDWINATEPEQQAYNAIHLSPET

FSHFKKWWHMFDSSTSMPARHGRLWMSPEERRELKKAPKKVSFGKSMHTVKYMIQLSQLF

ICHLYRHPENEDEASKHTRSAATGIKLRTDAYMMELHQRREPIKGSDVWKMKLNAGEIDF

QSCDIRVISAVFKEESAHGVLARHMGLKDDVSSLGSSTNNTNSHQSGTTNTGSSRVGTFQ

ISDGDFSWIDMDDFVEINESTPDGKSPEVAVIPMLYTPRWTYFRETDHEQKEYMTDSRGN

KYRRFGDETNVLDGFLGKIAPQLTQIRLFEEREKDLAEQIKTHEAMLDSLKHGGNQSGQA

VKDRIASVEETLFNLKTRYHVISHTCLGIRAPGESSCGPNSISERIKNELNDVNETKFNN

RFIVHNVHLKWNNIVRNAMFRYLHRVEERKKLTYFMSRGAVKRIEDLLKELDKTMADDHY

TSEQKQERAKKVTTNLLQEAAHMIDNRATCQERVDTMEEGLYNTYSDPNITIDNTYLVKL

VSPQVQLMSEQNPDKVVMLTAPNISLRITSAIDESDIDADFKGVVEKRHGVQLEHANFFV

LDREAVLNRSAFLMNNNNYGCHRNSLWPPWLAIEMCYDATALRDDVVVSDATVGLRYDKP

NTLRLKSLQESAREARCTEDEEKEAFNRFNRIEVDFPKVTAYCTSEQYFAIYTIAMDLLL

YSEPIKESNKKAMDRMLMTFDPKRLHSAPETIECLQNEMRRLRKIRDLMQTQKDGKKDVR

QIDVELASGYLELFTMMAATTKILVTSKETEDFTDTMKWAIGSDEIIWHLLEDDHTPLVD

FGMASPSFVRFQEPNGANTNCMEIQMMQGFNLKPNAPYPEMLGPFLDGDVNKNRRNAKLK

KLDEPALVYAAWKMLAPIGGIPVMELLEVQLQPLKLALDQDTAEKMFSYVFPKNNDSPFN

VHDPTKHKTMADAVAIYADSDNGSDLTGGNESLSSSLASRLDASPNNRYQVVGVKMPGDP

DSPSASSTRKSMESDSSDESSSGRFNIFRNRRRQDESDEGTQMMERASEYTSIVDIRLHS

AVICISLKGHGTKALADVHNFVFTIPDLVYQNKTWTNLDLANQVKKDVIRALLQHTGSLL

RNKIRPHKRETNSAPLRQISEYTKFTAVDDLRADKDSNDHQNGRAGLESRAGTFTQPSSA

SVSRKPSTDSSLRKVTTSKSKESNGKEHRKSGFFHRLRESL

>tr|Q6C7B3|Q6C7B3_YARLI YALI0E02200p OS=Yarrowia lipolytica (strain CLIB 122 / E 150) GN=YALI0E02200g PE=4 SV=1

MASYKPYPGSDSSNPTSSPGEANNNHYSTPRLSSPAAAESNRHTSVSTLPATAEWDQLPY

PSFNNSIHHKSQRSTSPRSPRSPMLPPQTISNRRFSPRESPAISRQPSQRTRARSSTVTS

DDGRSVFMSEFRKHSGSGARPDMKGLYADQVGSEQAIASDDDSLVIPGTMPRSNPIPRSE

IESEPMERTSSSKRSSKIFTRGRVSPDGSIAGDDSESGFMAKVSKLLKTKESKQDLINDI

LADEAANGNSQMYEEHYQQMQAGTLPVETQPSAVAFSPQYPLPYHYPETVAHIQIPPAGT

QLDKSAEYDLMMQRQQEEFAADLEPGDSKVPKRKSNSKKSRSASPSLHGIGIDGIDGMMM

VDGSIDPLTAGHFTHSKHHRSKSKTSKKSPTMSPAMLPTNPQSKTVPYPSHLSRNSSPPI

DILELSALDKPSSSRRKMKRSSKKSQTDYGKIMRHLYNSIPSLDVIVKVVMEPVETARKS

EYPNLAIVIAVVELLVFIWLLYQAIIIVEFACAVIRFICYPAIYILKAIASPFASIKRV

>tr|Q6CB62|Q6CB62_YARLI YALI0C21626p OS=Yarrowia lipolytica (strain CLIB 122 / E 150) GN=YALI0C21626g PE=4 SV=1

MNTILSNLGQTASELARKAAVDTEEIYTALDDDSGYQINELSTLYDDGDAFVLGLQRLKA

DKNLERARVLSHSSATPVEKQDLRKIGTDLLFVRQYELEVDGREQQLTSLIKRRETEEIE

LERERQEREALLEGDQDLTPQSHGLRKRGAHGSMDNEISQDATLQTKLRVDENEQDDIAN

DVLSLVRRMKQNAVDMNQKLAKDDAMVKDTAAALESSSTKMGSVGQKMNEYQRTAAIGYF

FYIKAVVFMVVAVAGGMIIIRLFPKW

>tr|Q6CCD8|Q6CCD8_YARLI YALI0C10252p OS=Yarrowia lipolytica (strain CLIB 122 / E 150) GN=YALI0C10252g PE=4 SV=1

MLFGVRLQNEIYEPWKDHYINYDSLKRLLRENVVPNDKEEWSESDETRFVQALDSDLEKV

YSFQTNMYNKLSDAIGEIEKQIVEVKEIDLVNLEHTIEDILESAQQLEHFSRINFTGFTK

IVKKHDRLHSKYSVKPLLQVRLKALPFHSEDYSPLINRLSAVYSFLREQAPQSQSASINA

KLSSYTNDSDLNYTSYKFWVHSDNLMEVKTRILRHLPILVYNNQNDSQDSVDPTLTSLYF

DNSDFELYEGKLQKNDTSPSLRLKWAGKTSDEDSQIYIEKRTVDHSIAGGDERLPIKEKY

IAGFINGTYHMEKTVQKMKSSHVPDYQVEGYEKSVENIQDFIRENSLQPVCRAQYTRTAF

QIPGDNRVRVIMDNDILFIKEDAFDKDRPIRDPNNWHRTDIDAVSDPKKVLRKGEYSKFP

YAVLEFRVQNRPGVDTADTGNSIITSSSVYRKHGKWIDELTSSHLVKEVPKFSKFIQGIA

SLYGEDDKLNFLPLWLSVLEEDDIRIDPKQAFEQETKKLKERAKTAAVRARAASVSSPRK

GATTPAGPSSSTKKVTIMEDDSDSDDDEGRPKNRKSLKHKYSIPFLGTFGPKLDDALSED

EEVVLPPGVVKPDVLIRNTGPVKVETKVWLANERTFNKWLHITTLLSALTFTLYSSVQAA

VDKSVATYVAYTLFALTLFSGIWGWSTYMSRLNYIKQRSEKHLDNPIGPLIIAVALLLIL

IVNFIATYKAVGEPVPLWN

>tr|Q6CEQ8|Q6CEQ8_YARLI YALI0B13794p OS=Yarrowia lipolytica (strain CLIB 122 / E 150) GN=YALI0B13794g PE=4 SV=1

MDSEAPVETLTAAQIKSYVDTAYILVCAVGVSLITPGIGMLYAGMIRRKNALTIVAQSVL

TTCVICLQWWIWGYSLGNVPNGKMLGNLSLAFMMGSPTSHDELSLDQLEEEGIPTSVHFI

FSAFFVVCTVQIFAGAIAERGRLISSQIVGFIFATVVYCPLSYWFWAEDGWLNAMGVLDF

AGGGPVHIASGTGALAYALYLGKRIDDNGEASMLARAKPHNPTLVLLGTLLIWYGWLFFN

SGTLLAVSSRTGYIMLNTQLSACTGGIIFAAVDKWRYGRVSLVGLCEGVICGLVAITPSC

GYISPWFSVVGAIITAGVCCSLSNINQWIGIDDTIRSFNIHAIGGIMGSICTAFFADPAW

AQEDIPGGWIKHNWVQLGHELAAVTTCVAWSFVLTYIICFVVDHIPGLKLRVTAEEEMLG

TDVKDLSEKVENMEDSLWGNSPSMSREPTVAKRDAEPMV

>tr|Q6C1K6|Q6C1K6_YARLI YALI0F15411p OS=Yarrowia lipolytica (strain CLIB 122 / E 150) GN=YALI0F15411g PE=4 SV=1

MLASAAVAFSPINVHSIEPLINSSESLQVREILKVDRTRLLCAVDGAGNKRACLTEFNNK

NIDVLAASLNNVLLGKQLEARSEASSSFSGAGRNSDEELDCSFEPRDLKKPIRIGALFAV

LATSALGVFPPVLATSVFKINLQSLPMTFVKQFGTGVVLSTAYVHLAAESQEDFTNECLG

DLSYDPTAMSLALAGTFIAFVLEYGSARWLRARHERKKPNHSSESDDCDKDQVKGAVDVI

ETQIDMSGAANMGCAAHNATLIDPNDKISVIIMEGGIIFHSVLVGVAVTIADDDGFISLF

IAILFHQAFEGIGLGSRIAGLRDSSLFFKMSMCTYFTIITPIGMAIGLGVMDSMNSNDPA

TIWAIGTISALSAGVLIWAGVVEMLAFDWLFGDLSFAPKKRVFFAMSGLVGGMICMSLIG

KWA

>tr|Q6C1Y6|Q6C1Y6_YARLI YALI0F12331p OS=Yarrowia lipolytica (strain CLIB 122 / E 150) GN=YALI0F12331g PE=4 SV=2

MVLRPLIGAFTILLIAGATLLSFFVLLAGARDSAPLNNFYWLEAETSNIPGAHDFTRWTT

YNFCGVSTDNKNFECSKNQADFPFDPVRNFGTEEGIPQEFIGTHKWYYLTRFAFPFYLIS

LFFTVITLFVSLFSLCSRLGSALAISANAISVFFWTIAASLSTACYSQAKGKFDQAHLGT

KMFAFIWTTEFILLLTLFFFGFACCSRKQSSKVESYSEKQPRANKFFRTSRKSKSTGVTS

AA

>tr|F2Z6D5|F2Z6D5_YARLI YALI0E23474p OS=Yarrowia lipolytica (strain CLIB 122 / E 150) GN=YALI0E23474g PE=3 SV=1

MIIIETLIGAVVFVAVYVAFVKLDYYRRKAKFETSDMPVAYNGLLGWKGLRHMLTVFNND

IGPVGWREVFATYGKTLKYYAFPSNTILTYDPDNIKAMLATQFKDFSLGLRKEALAPSLG

YGIFTLDGSSWSHSRALLRPQFSREQISRLESVETHVQEMMSCIDRNQGAYFDIQRLFFS

LAMDTATDFLLGEAVGNLQEILHPEMPRTGTTFQVAFDRAQRLGSLRIICQEAFWVVGSL

FWRRDFNNTNQHIHDYVDRYVDKALLARKEKSEIYTNPDKYIFLYELARETTNKITLRDQ

VLNILIAGRDTTASTLSWIFMELAKKPDIFHKLREAILNDFGTSCESISFESLKKCDYLR

QVLNEGLRLHPVVPVNLRVAVRDTTLPRGGGPQGDKPIFVAKGQKINYAIFWTHRDKEYW

GEDAEEFRPERWETTSGGALGKGWEFLPFNGGPRICLGQQFALTEMGYVITRLLQEYSDI

SIQPSDAAVKVRHSLTMCSAQGINISLTRAKEE

>tr|Q6BZQ1|Q6BZQ1_YARLI YALI0F31867p OS=Yarrowia lipolytica (strain CLIB 122 / E 150) GN=YALI0F31867g PE=4 SV=1

MIFSWSTTTTSTVDVKISPELPWHKDDISNVIKSAFAKLAISNHQEQEIVHDLRATGALT

ASWVAIDNNTQQVVGTIFLSEVKVDGQVQSPKWYGIGPIAVLPDFQGNGIGAKLMHTAIE

HCQGQLDAGGVVLLGNPEFYKQFEFVPRRDLSLMGVPSQYFQVRKFRGEWPSGEVTYDDA

FMRHSW

>tr|Q6C3A3|Q6C3A3_YARLI YALI0F01408p OS=Yarrowia lipolytica (strain CLIB 122 / E 150) GN=YALI0F01408g PE=4 SV=1

MLHMATDNYLFTNGRILGASSILFGGLQQLITDPAQAIASSNSLHVLFGMFLSGLSAFLF

TPIFMPNYEAHLPWAPFGSWTNLIAGVLVGFGTKLGSGCTSGHMLCGIARGSKRSFVATA

TFSIFAMLTARVVGSLPTCFDAAGQEVACHHIAENYSAEIPYLAGLLGVIIVLRNVIPKL

NLPANTRATLSSIFSGFAFGTGLLVSGLASPQKTLGFLAGVPPKFDPSLAMVMLFGVLPN

AISVWKSGFFKNVTPLLTTEFSLPQIFDVTPRLIAGAAIFGIGWGIAGVCPGPGILGSFL

DGLKGFSWLAGFIPAYYLAGKV

>tr|Q6C4G5|Q6C4G5_YARLI YALI0E26983p OS=Yarrowia lipolytica (strain CLIB 122 / E 150) GN=YALI0E26983g PE=4 SV=1

MDKNIEMMHRKRAKDVDQGLPVSASNWAEVATDEIGEVAAQNYTSTTHDRKTLSFSQLTP

KDRQRFLVLVLLYLLQGIPLGLAMGSVPYLLKEHLSYGEIGIFSLASYPYSLKLLWSPIV

DACYNKSFGRRKSWIVPIQAVSGLVLIWLGTFIEKLIMGGINEPGSQPVEKNLFRITLFF

LLLVFLCATQDIAVDGWALTLLSKEALSYASTAQTIGLNTGYFTSFTVFLAFNGPDLANK

YWRSVPSADPPISLSGYLTLWGLIYLAVTVCVALFQKEDREKHDDDSLVTIYRTMMKVLK

LPNIQIFILVHFLSKIGFQANEAVTNLKLLEKGFSKGELALTVLIDFPFQIIFGYYGGKW

STGKEPLKPWMMAFVGRLGCGVLAMLVVYMFPTERAKSGEGIGFGYFMVVIIMHVLNSFL

STIQFISITAFHTQIADPVIGGTYMTTLNTISNLGGLWPKIIVLKAVDWFTSATCTPKAG

KTFAAFSCVAKEAKNMCEQSGGICTIQTDGYYITSMLCITIGATLFFGWIYKKMRFLESL

PISSWRV

>tr|Q6CCE2|Q6CCE2_YARLI YALI0C10120p OS=Yarrowia lipolytica (strain CLIB 122 / E 150) GN=YALI0C10120g PE=4 SV=1

MLVTIVTVTSLPLHNDTDIDTDTDIDSDTSQNRHKHTIAAHMDFLLKDNSFQDYGIELAR

KAGSEIKTFATEELLRQWTDNVDDSVAPPAPKPNSPLVMPFILLIKATFLVRDMAVFITN

TIYVALVLPLDMSLVFLHFMASGMAGNTPQFSNILPLHLSRDVLWSGVPVSTGRGCRELT

LPPSLVTVDSQGGQVIRDNSRSRVRFPSSGLMGALTRFFRRVPFFSRAFPQVQRVESTPS

VDFHTAMGSLNDALNSQLIHRAAPVVVQKSNYSSL

>tr|Q6CD55|Q6CD55_YARLI YALI0C03630p OS=Yarrowia lipolytica (strain CLIB 122 / E 150) GN=YALI0C03630g PE=4 SV=1

MISKRKLLPFIVFLVIFGTWLSLPPKKRHFVDNWRLTNYITRLGASHNLYDDTIDLGHKN

GAHYFTAPRDQGPVAGHANYTEALCHVSQKVIVPDVQISASNEEGVKEAVEDYVARLPES

PLTSELLSYSEGDISGHQCMATKFSQIMLVLELGYFASWGLVEESRDYENDPKEKFVMSR

IQSGDLHVAEKTSVPDVIIEEDIGDDSVEEEEEEKEDDSASYDEPVERDLQVNPRAAHLS

AKYPSLLRHVEFNGMDYAAFVESRDKFTSNEPFTTDHVYKNGEVEKIRHIQVPKMGPGHA

AFMLRKTFNFEKIEISLSFGDGELWEDYVFEPVLRGGQRAGRVTAKYPGYVEDHKSDDSV

HSFPHNDARFFGDQCAPKPDDVMLMGKVIYGADISMKQYPFDDPIWLTHQAATAWQKVKW

YDLRPEFLNKGLLDSMCHFAHTNQGEAFNVEFGVGFKCSEGRHVPNLNYYFCQQLH

>tr|Q6CDD2|Q6CDD2_YARLI YALI0C01419p OS=Yarrowia lipolytica (strain CLIB 122 / E 150) GN=YALI0C01419g PE=4 SV=1

MIPQLFPSSDQGCAYVAQLVIDRIVAFKPTEERPFVLGLPTGSSPEGVYRRLVEAHKNGL

SFRNVVTFNMDEYCGLAPTNDQSYHYFMYHHFFSHVDIPEKNIHILNGQSDNFELECANY

EATIASFGGIDLFLAGVGVEGHIAFNEKGSTRDSRTRQVFLDESTIRVNSRFFEDPSQVP

RSALSVGVSTVLAAKEVIILAFGFAKAEAVKKTLLDEVSSDCPSTFAREHTNSQLIIDTG

SASGLGALAQSLHMHDGSPVFEEMPAVQKVEAK

>tr|Q6CE68|Q6CE68_YARLI YALI0B18150p OS=Yarrowia lipolytica (strain CLIB 122 / E 150) GN=YALI0B18150g PE=4 SV=1

MSLNPDPHTRRRSSSILRHLEPETITEQSDQNALPNLNATWVNNKGAWGVHFFIIGILKI

CFDLIPGVTRELSWTLTNITYVIGSYIMFHYVTGIPFEFNAGAFDNLTMWEQIDDGDQYT

PAKKFLLGVPIGLFLISTHYTHYDLHMFILNLLACLVVVIPKLPSSHKLRVGLPDLSPDT

PSTY

>tr|Q6CET8|Q6CET8_YARLI YALI0B13046p OS=Yarrowia lipolytica (strain CLIB 122 / E 150) GN=YALI0B13046g PE=4 SV=1

MKISTLFTLVAGASASVSQTKPRDHDAYKATACFTVAQNYNFGIEANYYESQYAFFHEFC

NTKPFLQSVLLCMDKAYNGNMKKLQKQFKERVLVMCKFVGEPRPDKQQVLELLTSAKDSQ

PVQIPEPASQDPKAPPSPLYTKPVIVDEETYEGTLATTNISELASDKGLWLASGLLGYWA

LILLFGTISHMFRKLFFPVLLKMTGLHINTLRRNVTMSALFGHKHAQGWLAGCLPTRVQS

LTVVGYWCLIFIFPCVGYKFETPTEGNSHTAQLRSYLAVRLGIMAVSQTPLIFLFAGRNN

FLMWLTGWSFDTFNVYHRWIGRGMVALAISHGVTFTVAVGDSLPAAYKRHWFYCGVMAVI

SASFMILFAISAIRHRFYEVFLIIHLCLAIVFVYAIYNHTEFVGYHEFIYAVIAVWSFDR

AARIARIFYSGINSRAKITVVDTNERIMRLEIRHSGRWTPKPGQYIFLYVLSNRKFWESH

PFTVFQSPQAEKDGSITLMVKAQKGMTETLYKRTLEAGTLDTRVLVEGPYGSKMPVDKYD

SSILIAGGIGVTAIYLCASSLLKSSAKSITVCWVLRSDQALEYFSPELDYLLNDPRVRVE

IYFSTQKQQLETSPSSSISDSESMVDNGVVTEKDGVVERAKTRSRLSSAATATNRLHISY

GYRPDMLTHVPNSVASQGGSTAIVVCGPPAFSDDVRLAVRDNLNSTTERVDYFEEAFSW

>tr|Q6CBB6|Q6CBB6_YARLI YALI0C20295p OS=Yarrowia lipolytica (strain CLIB 122 / E 150) GN=YALI0C20295g PE=4 SV=1

MHISLIAITIAMDHGTMDHATMNHATMDHSGHNMASMATSILTTAVSTAMNAMATGMDHS

TMDHSTMDHSSMDHSSMGGMDHDMGGMDHGGHMGMNMWLTTTWRDYPVLFKTMTASTGGK

CFGIFCALFFTGMFHRGLALLRQTMEAKWAQADREKIKETGVPAKTGLSGFFANINPIRD

LIRVTVAFTYYMIGYALMLSAMSYVLVYFFGICLGLAFGEILFNNVARTCQVAQIEDPKG

EDCCC

>tr|Q6CAM3|Q6CAM3_YARLI YALI0D01507p OS=Yarrowia lipolytica (strain CLIB 122 / E 150) GN=YALI0D01507g PE=4 SV=1

MRGETGLGESNEAIVFVRVYPEMFIRAHSTPDGQVVVKHKNRYSNGTRTIPCDLTNYGKL

VDPLLASKVIYTCTLCTLFASYYVVATVC

>tr|Q6CDZ1|Q6CDZ1_YARLI YALI0B20064p OS=Yarrowia lipolytica (strain CLIB 122 / E 150) GN=YALI0B20064g PE=4 SV=1

MKNETEVVAEEFELSSVYAEPPPAFDSKDESKVVVDTDINEITAFQSHESGESDISKTEE

KPTGLYGKIFGQKENKTVNGLSSRHVTFIALGGTIGTGVFLSLGQGITIVGPMGCFTCFI

IVGIFVYSVVICLGEMASYIPSSGAFAHYGSRFVDDSFGFALGINYYLQWAFSIPSELTA

AAIIIQFWAPHIGSWVWAIVIIVPMFFLQLISVKTYGETEYWLAIIKVFFVVAFIIIGLF

YDWGAMNGLKNAVPSPGLSNLKNGQAWVGGFSGFFQVVVLCFYSYGGTELVALTSGETAK

PWKSIPSAVRATVWRIMIFLVMTVFVIGLCINYKDDRLLKAAYDSDVAQSPFTIVFEDAG

FGAAKHVVNAILLTAVLSAVNACFFASSRMLMNMAHDKRMFSVFGLVNKRGVPIGALLLT

FAISCLVFLTTIWGNAVVFTWFMNITGASAILTWMSIGFVSIRFRQALKVQGIPLTDLPL

KQPLYPLLPILILVLGGFLFAGMGYASVKQDPFSWKNPFGTYLGVAVFAICYFGWKGWNY

KTDKFVRSADADLISGRVWSPGQGPDYMAADKEAIEARIRANKDVNIWGKIAYYNFIAGR

AVGAVYERVTTLPKLPKWSKKEE

>tr|Q6CEL7|Q6CEL7_YARLI YALI0B14685p OS=Yarrowia lipolytica (strain CLIB 122 / E 150) GN=YALI0B14685g PE=4 SV=1

MSLQNVVSQISALVPGLSPALSAPEVSQMTNVASGMLAELTANSNGLTPTEQLLSLLPKL

VTGPGALSGIVSHLPNGLVETLLNSPYGPAVIDILTSQVGNRPLEYDDLLMSIFPIPVWA

KTGTGVMKIVSMAHDIVGFNLPQSVINLVLTTQYNIYGGFPTPADVAPSAVFVAVNVILM

AAHFYIFFRGFLRRHYFWPSFGLGWQCILNCLGFGMRIGWGKNLLSLRLGIASTVFIILS

IILINLMNLLLAHRIMTFRHPETGDATWFGMLMILVYLAIGGVLLLAVVTEVTIFSYFLD

YTHWRQATGGMQAASVLIAVISVGGVFIIAIAYALPRGSLALSNQDRSRLPASNIESYGI

FYFPPKFSQVLQYKGDPTAKISSGKLAARVINGRDLNFSASLIVITSVILCATSGMRAAT

TFIGDRWSHHNKPIFSPTLFYVGFGVFECICNVLYLVARVDLRFYIPDMPRKGYGPILVD

PETMQYTDPYTDGRGVVMDEKKGFTDHVEDVINVPAPTYNPAYALSPQVAMAARAMNASQ

MPPLPKSGPPPPVNKHTDFQEAQTLPPNSSFVEPPPIVPPPMPHPTLIGEPKMLYTPQMV

PPPRMTPSNTSPRGSLKGSPKLISPEGSPKLPPQAPTLPKVSMGSPTLPNINMDSPNFGS

RSAPKTEMVSPTIPNISHSPAAPLSMPRHSPLPSHAPQLPEILPTPFPQTPYQIRTATPP

EFATPTNILPPVSPQHFPEPSFTPVEPGSSGHYARPYEEPEDEFRFSRDSENM

>tr|Q6C293|Q6C293_YARLI YALI0F09691p OS=Yarrowia lipolytica (strain CLIB 122 / E 150) GN=YALI0F09691g PE=4 SV=1

MSFEKDEKETNLGEVHDIEEVEPSLLDWVAQKLGVPPNDIDGTYPAEVHFMAEKFAAMTE

DEALVICKENLAYHQVDPNFREEYKIELEELLNSYHGSHDPTQQSVYVDEKEDHDVDPTE

RAMLLRYWATIFYWWSPYPEVRSVTDPFDDVNQTTLTWRVYVVGTIWVAVAAFVNTFFHP

RQPAISLSGSVCQLLLYPSGRLLQYALPDWGFTIRGKRFTLNPGVWSQKEQLLCTIMVTC

ALGTPYITSNVLTQYMPIYYNQSWAGGFGYSFLMMLVTQYMGFGLAGLLRRVCVYPVKSM

WPTLLPTLAVNKALLAPNRKEKINGWSISRYYFFFLVFCGSFLYFWIPNYLWEALSTWNW

MTWIAPNNDNLAIITGSVGGLGYNPIPTFDWNIINFGIQPILLPLYTSINMYVGVFISGF

FIMGIWYSNNNYTKWIPINTNTLYDNTGKKFKVTKILTDYVFDEEKYKNYSPPYYSAANL

VTYGAFFAIYPLSFVYTVLCNYEMMWVAIRDTALAMKNFRRSNYEGLEDPFSRHMKKHKE

VPDWWFYVILLIMFGLSIALVEHWPTNTPVWVIVLCLGLVFVFIIPFTLFLSFTGVPLSL

NVLAELIVGYALPGKFQALNLAKALSVQIASQAQNYASDQKLVHYSHLAPRDIFATQLWA

TLVNGLVALGVMQFQMHDVKNICEPDNAMKFTCPSETTFFSASIIWGVIGPKRIFDHQYP

TLKWMFLLGAGVGLLFWFIQVMLPKILLKYKPEKTQAILRYQRIVTKFHPVIFCQSCLNW

APYNLSYFTGGLYAAIFFNGYVKTRFLAWWRKYAYVFSAGMDTGIALSGIIIFFAVQYND

YSISWWGNNVPYAGVDTLGVTHPPLPDVGYFGPGPENYP

>tr|Q6C3H3|Q6C3H3_YARLI YALI0E34837p OS=Yarrowia lipolytica (strain CLIB 122 / E 150) GN=YALI0E34837g PE=4 SV=1

MGYGEKLAFRNDLGGNHFTLAQPRGGKGLDPSFVPRQKPGFITNTAKRAIAYIFVFSLIA

TVMLMQMRDNSDPNEPELALVKKKPAPLRKDPREQEVVLGKPKPKPTVAAKKEDAKIKVN

ARKGAPAADPAQVPEKAEKPAANSKDTKPLKSEAGGKAVKADEADEVNKAKTQKKLQDLE

EELEEQEEEEEDDLEELEDYMDADDAAGAAEARAGAATGAEDSNASPTDLAEKLGKKRPA

SDAQAKAMEAKKVEDAKAAKFKDERAKQDKVKAQQKQVRDEDSEYEEGEEGDEGEGDEEE

EEEEEIEIGDAKIAKALL

>tr|Q6CCU2|Q6CCU2_YARLI YALI0C06512p OS=Yarrowia lipolytica (strain CLIB 122 / E 150) GN=YALI0C06512g PE=4 SV=1

MSSHTNHDVISLSDWSDTKLDKRDHIIAVKEANDLEVVEVIEVGEVGDVSSADYSVITSR

SKPNKTWKTLLWDSANKSPGEKHLLIKLDWFLLSSVMLGYFIKTLNQNNINTAYMNGMKE

DYGMSGSQLNYLQTVWIVGYIVGQVPSNQILQRTSARYYLGCLELVWMALTFLTLTCTNI

KSLYALRFFVGLTESGFFPGVEYLLGSWYNKDELTKRSALFAVSGIAASMVTGYLQAAVI

HGLEHTAITPWKWLFVFDGIISFPVALYTMFVNPNTPETTTSWYFTKEDIAIAKERRAQI

GDTGNKEEPFLQVLKRSLKTWHIYFFSLIFLCYNNTCIASTQPSMISWLKSQGYTPTQYN

VYPSAVGRVGIGVTLIMAVVSDAFGGLNYPFVAAYFVVQIIGSAMLSYWNISDGAKWFAY

FAIGVPTAWGQPMIFSWLNRSLYRDYKKRNLVVSITSDMAYVTLSWVPILTWNAQDMPRY

FIGFTYNACLSSLGLVLTVIATYLWKRDLSSKSVADV

>tr|Q6CBT1|Q6CBT1_YARLI YALI0C15807p OS=Yarrowia lipolytica (strain CLIB 122 / E 150) GN=YALI0C15807g PE=3 SV=1

MIDVPLSQGTGYGVVLGAGLGFSVVMVFITWALRRYQREVMSSEEFSTASRSVKAGLIAA

SVVSSWTWAATLLQSSSQAYKNGVSGPLWYATGATTQVVLFATLAIELKRRAPGAHTYLE

VVKMRFGPAGHAVFMVFALMTNTLVTLMLLTGGSAVVNDLTGMHVAAACILLPLGVVLFT

LFGGIKATFITDYINNIVLLIMIILFAFTTYSNHEVLGSPGKVYDLLVERALQLPVKGNE

QGSYLTIKSYDGAIFFVMNIIGGFGTVFLDNGYWNKAIAASPAAALPGYVLGGLAWFAIP

WLCATTMGLACLALEGTPSFPGYPGRLSAEDVNAGLTLPAAAVALLGKAGAGCALVMVFM

AVTSAYSSELISVSTIFTYDIYKGYINPKATGKKLIMASHAAVVVFGVATACFAIGLYYI

GVSMGFLYLLMGSIISAAVIPAVLTLLWSGLNFWAAVLSPPLGFCFAITAWLVTTKSLEG

EINVETAGTNLPMLAGNVVALLTPAITIPLFTLIFGMDNYDFEKFKQITRADDSEFWKVD

AVEELGGSDEVASIREKKQANAPNKSTGESSAPENGMVAEYEVDDSLSDEAPIHIEELDP

AEKALLDKYAKISRIITIVMAVCLLVLWPMTMYGTGYVFSKKFFTGWVAVGFVWIFFSTI

MVIIYPLWESRQGIFETLRGLYWDCTGQTWKLRRWQSQTENKQMAAHGVTRTISHIERLQ

GEEVESEEGVQVTKVDN

>tr|B5FVD3|B5FVD3_YARLI YALI0C21789p OS=Yarrowia lipolytica (strain CLIB 122 / E 150) GN=YALI0C21789g PE=4 SV=1

MAIPFEALLPYGIIFGLLTAGGGAMQVLHVYRNGGVRDRFAIDQWDSQMMERDLRLNGGQ

GRKQVDQATAPEAFKHNHVWKSERPLI

>tr|Q6C1V3|Q6C1V3_YARLI YALI0F13145p OS=Yarrowia lipolytica (strain CLIB 122 / E 150) GN=YALI0F13145g PE=4 SV=1

MSDDLKPHTSHPQDIETPSSHQDEPVVLGRVRTSGERDEYIHIGGVKYHRDDFARAFGGT

LNPGSAPVPTRQFGNAAPSGLFAFSMTMFILGMCLVNARNVHAPNVMVGCAFFGGGLIEV

IAGIWEIVAENTFAATVFFCFGAFWFSWSMLNFPIGIEKYYSTPDEFAQSVGIFLMGWFI

FAFLMTLCTLKATVAFFLLFISLDLALIFLAAGYFQSNSKLTNAGGGFCISTGLLGCWNG

YAGVASPQSTYKWLIPKAVMMPGAHQ

>tr|Q6C704|Q6C704_YARLI YALI0E04807p OS=Yarrowia lipolytica (strain CLIB 122 / E 150) GN=YALI0E04807g PE=3 SV=1

MLQAAIGKIVGFAVNRPIHTVVLTSIVASTAYLAILDIAIPGFEGTQPISYYHPAAKSYD

NPADWTHIAEADIPSDAYRLAFAQIRVSDVQGGEAPTIPGAVAVSDLDHRIVMDYKQWAP

WTASNEQIASENHIWKHSFKDHVAFSWIKWFRWAYLRLSTLIQGADNFDIAVVALGYLAM

HYTFFSLFRSMRKVGSHFWLASMALVSSTFAFLLAVVASSSLGYRPSMITMSEGLPFLVV

AIGFDRKVNLASEVLTSKSSQLAPMVQVITKIASKALFEYSLEVAALFAGAYTGVPRLSQ

FCFLSAWILIFDYMFLLTFYSAVLAIKFEINHIKRNRMIQDALKEDGVSAAVAEKVADSS

PDAKLDRKSDVSLFGASGAIAVFKIFMVLGFLGLNLINLTAIPHLGKAAAAAQSVTPITL

SPELLHAIPASVPVVVTFVPSVVYEHSQLILQLEDALTTFLAACSKTIGDPVISKYIFLC

LMVSTALNVYLFGATREVVRTQSVKVVEKHVPIVIEKPSEKEEDTSSEDSIELTVGKQPK

PVTETRSLDDLEAIMKAGKTKLLEDHEVVKLSLEGKLPLYALEKQLGDNTRAVGIRRSII

SQQSNTKTLETSKLPYLHYDYDRVFGACCENVIGYMPLPVGVAGPMNIDGKNYHIPMATT

EGCLVASTMRGCKAINAGGGVTTVLTQDGMTRGPCVSFPSLKRAGAAKIWLDSEEGLKSM

RKAFNSTSRFARLQSLHSTLAGNLLFIRFRTTTGDAMGMNMISKGVEHSLAVMVKEYGFP

DMDIVSVSGNYCTDKKPAAINWIEGRGKSVVAEATIPAHIVKSVLKSEVDALVELNISKN

LIGSAMAGSVGGFNAHAANLVTAIYLATGQDPAQNVESSNCITLMSNVDGNLLISVSMPS

IEVGTIGGGTILEPQGAMLEMLGVRGPHIETPGANAQQLARIIASGVLAAELSLCSALAA

GHLVQSHMTHNRSQAPTPAKQSQADLQRLQNGSNICIRS

>tr|Q6C8F4|Q6C8F4_YARLI YALI0D20108p OS=Yarrowia lipolytica (strain CLIB 122 / E 150) GN=YALI0D20108g PE=4 SV=1

MNFDNFPAPDLSPKNIKRYLVTRVTSLKPPKLTVEEKKHINPIPALRLLNRKQWLFVAVG

FCGWAWDSFDFFSVSLVASEIAESLHVSVTSITWGITLVLMLRSVGAVIFGMLSDRFGRK

WPFITNCVLFIVLELGTGFVQTYKQFLAVRALFGIAMGGIYGMAAATALEDCPVQARGVI

SGLLQEGYAFGYLLCVVFTRAIADTSPFGWRALFWFGSGPPVLIIIFRLCLPETDTYLLS

KKNAAEAEGIEKNFWRGIKMTFKTYWLMFAYLVVLMGGFNFMSHGSQDLYPTMLKKQLGF

SEDRSTVTNCVANFGAIAGGLVVGHASSFFGRRLCIMISCVIGGALIYPWAFVTNSGINA

GVFFLQFFVQGAWGVIPIHLTELAPPVLRSSMVGVAYQLGNLASSASSTIEATIGETFPL

YDKFGNEKPGVYNYSLVMAILMGCVFFFVLCVTFLGPENRQAEMISGGSEQDNAYERKLE

EMEDEEKGVTENIERRSSSDTR

>tr|Q6C8Q0|Q6C8Q0_YARLI YALI0D17952p OS=Yarrowia lipolytica (strain CLIB 122 / E 150) GN=YALI0D17952g PE=4 SV=1

MSAESIVESTIEVTAANVSPVPSAAEIILTPSFSQRFPLVIPLISTLVLIAIGLFVYVSF

AKKQALRRPPSVVICGPSGAGKTALWSALTGESVPATVTSFQQNIKSGYEGKNYALVDYP

GHNKLRQGLWTEVNGGAVQGLVFVVDLASLQRNITETGSFLLDLLLLLEGSNLPLCSKRL

LIVGNKSDVFNAAGLAKMRLVLQDELRQQKESRSKSVSESNVDILGKVFDFNSLETEVEF

CETSVKRDSFAKVKGWLEEL

>tr|Q6CA45|Q6CA45_YARLI YALI0D05995p OS=Yarrowia lipolytica (strain CLIB 122 / E 150) GN=YALI0D05995g PE=4 SV=1

MKYAEDHNGYYFQLPGEKTSLTIAKGSGAIAANEGVVAPGGKEIAAIVGTIRLLASQYII

VASKTETVGAIFGQQVHRVTAFDILPINGGSADPQEQQYLKILQFHLDSSRLYFCRTWDL

TTSLQAQSHAQRAPGVSFETADERFFWNKYVCTDLIDAARTQPGVALFVTPMSFGFVELS

QSTINGRSITFGVITRRSRHRAGTRYFRRGIDAHGNVANFNETEQLLIVEGTAEPPRVFS

YLQTRGSVPVYWGEVINLKYKPNLQIGQPATDAAKLHFDDQIKRYGRNYLVNLVNQKGYE

LPVKRAYEQLVDQLGYPEDQVSYVYFDFHHECSKMRWHRVLLLIERLQELGLDQQGYFEA

TLMPNRQLQPQANQTSVVRTNCMDCLDRTNVVQSTLGRWVLQKQFEAAGILAPGQKWETT

EPKFELIFRNVWADNANAVSVTYSGTGALKTDFTRLGKRTKQGALNDGLNSATRYIKNNF

QDGIRQDSFDLFLGNYVPYSGRPARFYDVRPILFQAVPYMVLSAVVLILCATFFPRQDLS

ALVSRSFVGFWVLVLVWSVRYMTKNGLQFVNWPKLIKPDFVSENEIVNNGKVSGIVYKVG

DSDRVNKLD

>tr|Q6CBC4|Q6CBC4_YARLI YALI0C20097p OS=Yarrowia lipolytica (strain CLIB 122 / E 150) GN=YALI0C20097g PE=4 SV=1

MTDKVIQYYEHYYDRSRDLEALATYVANHYTHNMPIIIGTLCCMLFVCSYVLHDIITESK

EVESDEKELPLPVAKPRQSLFREFSFDSHKEYDNDEVDPLTTLDELKEMEEERKKQKERK

REELRLEEEKRAEQERKKTDNTYPPSVLDPMLDPVHSFKQPFWTESKEPYWETTDSMQWP

EPQITLLEALA

>tr|Q6CBW6|Q6CBW6_YARLI YALI0C14872p OS=Yarrowia lipolytica (strain CLIB 122 / E 150) GN=YALI0C14872g PE=4 SV=1

MPYIQSYRAYPNRPPPPPSKPYWYRFVHPDWFLNNLNLKSAKIILRNWAASFSMLFLLTV

KPAWAWTGSSAHLALIMTVVQTSGQVSVIGAFIANCVLCAFIVWGWVCGYIIPGYIANRY

FYDSVTQADILKDVISRGVCSASLPKKELIACMTDYCVHGHYMKAGPGVVYAIWVAVACT

VCFWCKFHDPMTFAAGASTSFIAAAVAADSAMHFPWYAPKTIGAGMLKPMGIGLAANVVV

AIIFFPFTSGYQFTTKLLRFQGAVVQTLEWQVGFLAQAKPSVPDTWLGYWKLEQCVSGLR

SLGPVLRMSSGPIPLEFSFTRFTIAGYQSARITARRLTSQLTGLIMIFDSIEHLRRVIIE

DKEASIEPVISEIHKKYAPVGVYETQHSVASVLRQSEKPVTLSDLDETIQTLSDICGPMV

LEQAGMMKVAIKWITVANDFRGWSWLPWLHKKHVLDQQTMALEISSAYEHFSKATESFRE

KRFAVTENTMSPYLILQKTLYCGYVEEVNRTITFMVEKLISFDRDHPTPHWITGIFKMAS

LKFASISVFTSSADALVGNDAEAEKITPLKAERDPDALPARHWGHKIGKKVRLFYARSRA

SSWLVPIKGALFCVAALTPALFTNTHYWYYKNRCYWAVVMTGMSVAEYAADNMFSAVNRI

FHTFYAVVIGMVTWYISTGNGHGNAYGFMVAFGIVLLLMHFYREFWPPGVSPVPRLMLCI

AMCLVVSISWLDGQNPGAVYVGYGFTAAWHRYVTVCVGIVIGMICCNFPHPKSGKKAVRL

IVASVVGNTGTLFCQVMTFAAEKLKHPGLVVDATDDPFSDNIEMCVLKLMGAKRLSGVIK

YEPDLSGPWPRRSYMELLALCSELVELYAHLYFVIKRLEDVEKWLPEVIRCVGWDHPVLM

SNFFSVVFMASTGLREGIGLPQMTPGHLYHEHAAVARKRLMDANLQGPVNTPDSATFLSA

VSIANKIYDRMDRILVLIKAIVGEQYSHHDYYMQKMLENMA

>tr|Q6CET3|Q6CET3_YARLI YALI0B13156p OS=Yarrowia lipolytica (strain CLIB 122 / E 150) GN=YALI0B13156g PE=3 SV=1

MRLSKLAVSSVLAAVACAQDADAAADAAPSSQVEHPEFTPYTGAVTGFFEQFLDGHKWQK

SSAMKDDEFSYVGEWAVEEPYVFPGFKGDKGLVVKSPAAHHAITTAFDTPINNKGKTLVV

QYEVKLQKGLECGGAYVKLLSAEVNADDKGVEEFSSETPYQIMFGPDKCGSTNKVHFIVK

RPLPDGTYEEKHLVSPAHARLNKLTNLYTLVIRPKNEFEIRINGNVVKTGNLLEEGLFKP

SFNPPAEIDDPEDTKPADWVEEPYMPDPEQAEKPADWDEKAPFYIADPEAVMPADWQEDT

PDYIVDPEAFKPEDWDDEEDGEWVAPEIPNPVCEEIGCGPWVAPKIQNPDYKGVWSQPMI

ENPDYKGTWAPKKIPNPNFKADEHASDLEPIGGLGFELWTMQEDILFDNIYVGHSVDEAE

AIGNATFVPKLALEAEEEKLSGPQKETAPWDTDEGVLSSVDMFLADPVSFVLERVLGFFE

VFSQDPVSAIREDPVVAAASFGLLLITSATAFGLLNVIIFLLFGKKKQSAAPAKKTKKTG

DGPSKLTKADVVEAEAEAEQAAETVVASGVDDGSAELKKRKA

>tr|Q6C189|Q6C189_YARLI YALI0F18326p OS=Yarrowia lipolytica (strain CLIB 122 / E 150) GN=YALI0F18326g PE=4 SV=1

MPSSSSEQHPSAPNGSLDKDLFSEHSRQLEIDKESLSSQLPSYRRRKRLDIVSDEEKEDV

GPPVHRHKLRPTSMAKVLGLNMTELELLRFYFHESSPILCAASGAKDSIWMGNVPALSNR

SPALKRAAMTFATLHLGKNRQTATYLLNDGNEHPTSSGPPDMKQVIGYVPLQDRVTERLL

IEFTETLRAHKKEIMETEAENCEALLVTSVVIYLVAMSLGPLIPLANFDGGSDLFSIVRN

LRLITTTITGQEPVIHPPFEAPAEGSIRLPREEALWEIVELVELNMDYSPITKRRIKGIL

TKEINSLIQLLNMDILNHSVAHFSAWCTYWQPEFSDLKNNANPYALMIICYYAAYTHMWH

ILFWWADRSSEDVYYIMDHIPIEFHDYLQWPLEIVQTYEYNYEDMLSGKLREMTLA

>tr|B5FVE3|B5FVE3_YARLI YALI0D08635p OS=Yarrowia lipolytica (strain CLIB 122 / E 150) GN=YALI0D08635g PE=4 SV=1

MPAEDKVSKNVPLEFIQEGTAFLNKCTKPDRKEYTKIVRAVGVGFLVMGAIGYIVKLVHI

PIRHVIAA

>tr|Q6C4K9|Q6C4K9_YARLI YALI0E25894p OS=Yarrowia lipolytica (strain CLIB 122 / E 150) GN=YALI0E25894g PE=4 SV=1

MSQNWRRKSSVSAWDKPEGGWSRRQRGGIIATVGGFIEGLATALIRRKIFVVIFLLLTLL

SFFTAVPFVPHFNWGSGKYVIILAANEGGGVMHWKGAKEWAIERSSIENKREYAERHGYH

LAVKDVSLKKRYSHEWRESWQKVDIIKETMRQFPNAEWFWWLDLHTLIMEPQISLDQHIF

NNLYNETYRDLTGQFNPLNLPVQIPYVDYNQPVDLIVTQDCGGFNLGSFFIRRSDWTDKL

LDLWWDPVFYEQRHMEWEHKEQDALEYLFTNQPWIRGRVGFLPLRKINGLPPGACSEMEN

DKQLFYDEKSRDFLVNMAGCEWGRDCWKEMESYKAKAVELHRKKWWLF

>tr|Q6C4L1|Q6C4L1_YARLI YALI0E25850p OS=Yarrowia lipolytica (strain CLIB 122 / E 150) GN=YALI0E25850g PE=4 SV=1

MYYSNNNVKHLLDEEDVVPYSSSFIGQWYYTYKVHFTVGMITPLEAGFCNVVIVLVAALL

MVALLYLPQNSMLLANRAYYYWSGHETWSTPNPPNFTQILGTAINKVYHTV

>tr|Q6C541|Q6C541_YARLI YALI0E21197p OS=Yarrowia lipolytica (strain CLIB 122 / E 150) GN=YALI0E21197g PE=4 SV=1

MRNNLAEKNDLLEVHPNGNGTTNGPGNEFSASSRTSSIQSFLGNYEMPQVEPHKAAGNKS

TDHYKSRMSPWRYRMRSAMLPLIRWETPYLAQIQKSSRNIWLDVYFAMTANLGTHTFYVI

MLPVLFWFGQADMARGLVFVLAYGVYVSGVIKDLLCLPRPLSPPLHRITMSGSAALEYGF

PSTHTTNAVSVTLLFLQKLYECKDNLSSFSFESLRALCVLYGASIICGRIYCGMHGFLDV

ISGFLLGALLWWIRFAFGDLMDATTTAEAPYALLAIPLALLLVRVHPEPVDSCPCFDDGV

AFMGVIMGQDVGIWLFGKTQYATSVPLRGAIAYNFQDIGIIKSFLRVVVGIMLVLVWRAV

AKPMMHTYLPPFFRIIERVGLSMPRRFFLSASEYKDVPGQIPDSTLIEPHEIPSLIHKLG

HARKDSVGPQSTADVIESIAYAEYQRQRQDKKLGVDDDAPYPDAEGVSYRGDKTSKMDEK

CGHSHGHDQDAEDELFASIQPPRVRYDVEVVTKLIVYSGIGCLVMFCGVVFQVVGLGVTA

EHLTHTAAKVVGEATEKIL

>tr|Q6CB29|Q6CB29_YARLI YALI0C22363p OS=Yarrowia lipolytica (strain CLIB 122 / E 150) GN=YALI0C22363g PE=4 SV=1

MDLQVDPQIIPQMDEFPPPPSQEEINDRIKQLAIKHNVNHRKLMLKIDICVVPLTCLLYV

LAFLDRVNVSNANVYGMSEDLNMTGNKFNTALAIFFVPYVIAEIPSNWLMKKFKPHVWLT

GCMTLFGVCLLGQGFVRTYGQLLATRFLLGLFEAGMFPGCFYLLSMWYRREEAQKRYSFF

FSSTTLAGAFGGLIAAGIHNLDHDRGIRSWQWIFIIEGACTATIGILMIFCLADFPEEAR

FLKQNERDFIKEKLEIGNAGVSEYERSMTRKDLAFVFSDWKVWISGLLYFGYIVPAYGYA

YFGTAIVKGLGYSPVMTQLYSVPPWVAGFGFSMVSAAFSDHFRHRFAFAFFAGIVAVAGF

GLLMGQTKHIGIRYGSLFMICAGTYTAMPILVCWTQMNFSGHHRKAIASGWQIGFGNTAG

FISTFIFEANEAPTYPTALDVCLAFTAFSTVLLAVYFAAIWADNRYKRTEKYRQKFRSWP

KEKQQLAGELHPSIFYSY

>tr|Q6C2G8|Q6C2G8_YARLI YALI0F08019p OS=Yarrowia lipolytica (strain CLIB 122 / E 150) GN=YALI0F08019g PE=4 SV=1

METSTPAKQDKGKSRDSSDGGSSSGSSPGSPSPSNIPIENYAAKLVIRFSNGIPDLVIDV

NDVRHINVSWVKQQIRLRVGGLVTSKRLRLINQGRLLSSSTSFARDVVKVMDPENGVDGM

PSIYLHCSVGDTLSEQELAEEVDEQPQRSTLPELRGFDRLRTAGFSEEEISDLRRQFRNI

YGGITSDNNLEQMQNLEEEWIDNGVNAGGVPNVDLNPGGGTFAGDLIGMLMGLFLGILSI

YFLQEQSLFSKRQQRAITAGLAVNFAFNVLRYLYS

>tr|Q6C6Q0|Q6C6Q0_YARLI YALI0E07381p OS=Yarrowia lipolytica (strain CLIB 122 / E 150) GN=YALI0E07381g PE=4 SV=2

MIRKIVVFLVCGALLILKSYIYAYHRFPKHDTLSVKEHGGTNSYSPVMELHKSLVDIPSV

SYNETHVSRFLQLYLSTRGYTIDLIGDHTRQNVYAYKGAREDAKVLLTSNVDHVTSGTPY

NVIDGAIYGSGALDAKSCIAAQVTALEELLRDRKVAYNEVALLFVVGGEVFGSGGMTEVN

KMVKPWKTIIFGKPSDLNLVSRHCGVVIIGLEGENQVLIDMMQAFKTSIISGTVNVTFTN

NQKATVEVRFLGDEENVASEVAGVLTRFRDVIFTYQSYPPQDFSCEVPGFESTVSSLSSD

SPHLHGDFVRYLYGPGNMSLANAPDEHITVTDLLAAVEGYKKLVLFAIA

>tr|Q6CAK9|Q6CAK9_YARLI YALI0D01914p OS=Yarrowia lipolytica (strain CLIB 122 / E 150) GN=YALI0D01914g PE=4 SV=1

MSNQTQMSKRSPSILVTDVRTNPVRPAPFAGGRRQSFSKRPKSKGNKFSNISVSGLLDGA

PAKRLQHDYQSTVQKARGIPQRTSKQSQKLVLIPDESPSYDDDDEDAYGVPIPGKLKGHE

LFDLNTDADAYDEANAKVYVTDVPRRSIAERMSKDARDAKLPRVTAYCTAEGYRLKATAE

FLQENHEIRPRIYDEALFAPYYLPLLPGEDGCRVRSSPPLRNAVGESLMETLIETSENTD

HHFEYYGADGEEPTDFSPKDAPIPLKEESTSPESLSEPQPGSSNDTHKSDAPPPTTKRLP

TLPDLKRHAELFIFSYGVAVFWNFTEAQEKEILADLVFASEERKEEGSGSVDEDYEEEFS

HNTTGQDWGKLGSRSYRHAERHEIETEEFHFVYSPQAERPRIFNDMITLRSGDHMVKLAM

SHAIAQSTKLCRFEARMDTNMHNVKHVPKVLALTGKLGMQREDVLKMSGKLFQLRVDVNL

SSNVLDTPDFFWEAEPSLHPLYSAVREYLEIDQRILVINERCKVFLDFIDIIADSIAEFN

MSRITWIIIILIVLSLIVSGFEIAFRYSMLRRRH

>tr|Q6C2T0|Q6C2T0_YARLI YALI0F05434p OS=Yarrowia lipolytica (strain CLIB 122 / E 150) GN=YALI0F05434g PE=4 SV=1

MALFRVLTLLSLALLGAAQYSPEELQGYYGLVEPTNRTQNNYNSFSLTVEGDYVSNIQAL

TVETDKLAGLNSAAAVSGNLRMISGNYTGPALDWSNAALIDCDNTTDVIDALNEVLINNP

ACMILYSLNSDACHFTRPYRVFSADIGFVFTTVSAKGAQSLLEKVQQSPVLLHSVAWLNT

TSLPDDNTPVVDDKGPSRDTKIAMAVLYCICGVLALAVVVLISLGIWRVHRHPERYGLAP

PDQTDEEREISDDFNNGGGDVYTPPPTRVEKAKGLARAVLDAIPLMRVMIGSQEDEKDKG

TAPGESSSSPAMSTHKRDVSNVSVKSSCDMSRMETLPVYIQEDDNCAICFDNFEDNQIIR

QLPCTHRFHADCVDHWLLNSSSQCPMCRMNLRKEENETTENEATTPSTHTQTQTAQPQTT

SQQQPSVITVTQRDLNISLATRIIDWWNMLCLPEDARAEARERLNHESELRRKIRQLQRS

DGTLMPNDGIVATDDGALRVLLDNRPLQAPPIDVPLSERMLVPGPDLDLNNVEAPVPRYS

VLPDMSAASESVYNRDSVSLPQACLLPQLDLSEFSVDVDGMNTHYDTSTGTGSDIDTDIG

DVTRVDTRDATTAHVDGDCASYVEDAKTKEANETASDALYDPASDTLELETLRDASAACE

DGPAAHGTHQDPDFEFDFENTTHSKPI

>tr|Q6CAU1|Q6CAU1_YARLI YALI0C24332p OS=Yarrowia lipolytica (strain CLIB 122 / E 150) GN=YALI0C24332g PE=4 SV=1

MDFTSVTESWVDGQIGASVYDYGDSEPLTDDLRSSKQKAVEAAYSLGDLSLLKLLAQSKG

GLLNSKLRKQTWPLLLSEASVTGSDPPEDPHPDETQVQLDIDRSFVYYPKFEDDEKCAEA

RQNLSKVIVTVLRRNPQLRYYQGYHDIAQVVYLVMEDVHQSIQVLEKISLIYLRDFMLPS

MGYTLDHLEFIPSLVTELDSHTGDQIKEVRPVYGISSVLTWFAHDIPEFDTVCLLFDFIL

ASGTMATPLFIYASLTSLRSDELSNLDPEDIDIINCVLNTFPKTEQETVSRCIQKAQKLA

SKTMLENYPAWHKLSNYSVLKTTAAQSPQAESPSESILSSNSMSSSATAPSLISSYLSLQ

IKESEVREENERLARQKLIDKRNQQREQNKLSKKNSSSSISIRRIFFFSLCIGLTSVILA

IFISPKDSRSYLTRHFEAGIQGSLQLLHRIATGQHSF

>tr|Q6CBQ5|Q6CBQ5_YARLI YALI0C16522p OS=Yarrowia lipolytica (strain CLIB 122 / E 150) GN=YALI0C16522g PE=3 SV=1

MKLQVPAFARSSSDVKTSFMGARGQKLHNLVAAIAGLGFLLFGYDQGVMGGLLTLDTFIQ

QFPKMDTSDYLPPKVKTFNTTIQGTAVGIYEIGCMIGALFTMWAGDKLGRRYMIFFGSII

MTIGAILQCASYSLGQFIAGRVISGIGNGFITATVPMLQSECAKPERRGKLVMLEGALIT

AGIALSYWIDFGFYWVRTNDADWRFPIAFQIVFSLVLTFTIMSLPESPRWLVKKQRFEEA

AGVFAALEDVPLDDPYVINQITSVKESIMMEQLAQLGVDGVDARRKIQSGEFQMGQELSF

IGQMKLMFTFGKKKNFHRTMLAYWNQVMQQVTGINLITYYAAYIYQTSVGMNATDSRILA

ACNGTEYFMASWVAFYTIERFGRRKLMLFGAVGQACTMAILTGCVYAASKPEDGGLDMQG

AGIAAAVFLFVFNTFFAIGWLGMTWLYPAEISSLEIRAPANGLSTSGNWAFNFMVVMITP

VAFNSIKWKTYIIFACINAFMVPMVYFFYPETAGRSLEEIDMIFAESNPRTPWDVVGIAN

RLPKNSVATYDDYEAGEQEEKAIVETAESVSHDSAEFQ

>tr|Q6CCU1|Q6CCU1_YARLI YALI0C06534p OS=Yarrowia lipolytica (strain CLIB 122 / E 150) GN=YALI0C06534g PE=4 SV=2

MSEPQPITQPTQTTPLLASSYAQEVNPFQREIRRRSSVSSANRYRPPRGAIGWWQKSVIV

VIAAVLFILLMISLLTLATDHGRDWRWKDYPGHEHEPGHNHTTV

>tr|Q6CET6|Q6CET6_YARLI YALI0B13090p OS=Yarrowia lipolytica (strain CLIB 122 / E 150) GN=YALI0B13090g PE=4 SV=1

MNLLLFLTLLGFVCAANTPKLADHDKWKCQPCVATAQMFKFGITATRYTPELYYKQQCSN

KPYLQTLLKCMQDHTDEVRGRKMLEKLVGKRCEENGFELNIEDLEPIPELVTVAPENINK

TQTAPVVVPEEMYAPSYKSYSTYFHQTDMGTYLGVGLLSYWTLVLVVGAFVNLTRTYLFP

IMCKLTGLHINTIRKHISLPAAFGYKHSQPFRVLQGICSMCMPTRVQSVIVTLYVMFAFI

ACFPGYKIFDENTVFESHATQLRRYLANRTGILAFAQLPLLFLFAGRNNIMMWVTGWSFD

TFNVYHRWIARTMVALAITHGVTYTILTGKFLSSSYPELYFFMGVIAVISGSFMCIQGLH

FFRSRWYEVFLIIHIILAVLFTMGVWFHADDVGFQEWVYAAVAVWAFDHLARYLRILFSG

VICNGQFSVVDHEHQIVRVEIAYSKLWKVYPGAHVFIYVLTPWKFWESHPFTIYQSPKAM

ENGNISIMLKAKSGMTLRIFKRLQEQNNSANIKLLVEGPYGHQLPVRNYDSAVVIAGGIG

VTATYSYAADLLKCATTKAITFAWVVRNDAAIDWFKDELNSLLQDERVHVSIFVSTQEPD

DPERDVHQRFGSYSASASSDDEEKTDKGATTTATAMDSTSDGGSKDTSVRRLRKKLSITY

GTRPKMCTALPQFLSFCEGPTAIVVCGPPVFNDDIRLAVSESLHTKNERVDYFEEAFSW

>tr|Q6C025|Q6C025_YARLI YALI0F28369p OS=Yarrowia lipolytica (strain CLIB 122 / E 150) GN=YALI0F28369g PE=4 SV=1

MDKESLSHDEYTESGGLEHVEYPQGQVPQEVIDAEIAQLAEKYGINQRKLMFKVDICVIP

VICILYILAFLDRVNISNANVYGMSKDLNLYGDRFNTALAIFFVPYVVAEIPSNWLMKKF

TPHVWLTGSMLLFGVTLLGQGFANNYSALLATRFLLGLFETGMFPGSFYLISMWYRREEA

QKRYSFFFSSTTLAGAFGGLIAAGIHNLDGDKGLASWRWIFIIEGACTAFISIIMFFLMA

DFPEDAKFLTENERQFMKEKLEVGNAGASEAERPMTWKDVKSVFSDWKVWVMGWMYFGTI

VPAYGYAYFGTAIVKTLGYSDVKTQLYSVPPWVAAFGFSMLAAIFSDYLRHRFYFAVFAG

IVAIAGLAVVIGVHDKIGTRYGSLFMICAGTYTAMPLFVCWTQMNFLGHHRRAIASGWQI

GFGNCAGFISTFVFQANDAPFYTPGCAVCLAFACLSLVMQFVYAAGITWENKRKRTPEYI

EKFNALSEDDRRIAGELNPSFFYYY

>tr|Q6CE52|Q6CE52_YARLI YALI0B18502p OS=Yarrowia lipolytica (strain CLIB 122 / E 150) GN=YALI0B18502g PE=4 SV=1

MPEKSTTTSEIRSEISSASKDISSNAPIALKASGVFFSGLAAGGTLITTLYLRPIFSQMT

TNAAYTVFNFVYGVGKVAFPLFAGLGAASFGGAAYIESQRTRKDVTPRKWYNPSSSTLLA

YSAASLVAIVPYTLIVMKPCLTMLFDRRGEVVKGEDIMDLLNLWATHHLARVVLTSAGFL

GGIVSLLK

>tr|Q6CFC1|Q6CFC1_YARLI YALI0B08426p OS=Yarrowia lipolytica (strain CLIB 122 / E 150) GN=YALI0B08426g PE=4 SV=1

MTEPLVINISELRKYKDIDAKSMLMRVGRWYFYQLGELQKTQHDLELFQQLVYRFKFISA

AQEKALRKVNLESYPNKHLFGISVHLDGTETLRFYESEGTPGGIIHAKDQRLDNEPIISP

DSRNYPRVGDTDFEVPSQQFPYLSYGAMLVIAVFLVAFITKKLRHVDKRRD

>tr|Q6CH13|Q6CH13_YARLI YALI0A14069p OS=Yarrowia lipolytica (strain CLIB 122 / E 150) GN=YALI0A14069g PE=4 SV=1

MFCAGVISKLPRFFILVFLVINIFLATFIFMGCINKSASFSNVYLVEYSYNQDSVFFDAI

KSSFKQSNATDLAYLKARSGFFGACATVSNATECVSRGNLTALEDMFPSLSITQSGTNSS

ALGLIQLATQFTGDIVHPSLPVVTLVLDVLVFVFVLWSIFAFIPGATQAGIMMMWTSICA

FLTWSVCTIWFHVSTLVGTIFIESSSAGAVTSAVGGRIKGMIWTTFTFQLLIGLLAVWTT

HKSSIQKAKKEADYEARAYAVKA

>tr|Q6C1P2|Q6C1P2_YARLI YALI0F14597p OS=Yarrowia lipolytica (strain CLIB 122 / E 150) GN=YALI0F14597g PE=4 SV=1

MKFTELLAEYQVPEWKSQYVDYKWGKKLLKKIPDDLKDRLKRHNTFNDEIDAPSEPQNIA

AQQQAVLVDQTAGEASISPKTVAPAAAAATSPNTGITPGALLGEPSETEPFNSGSENDNY

GATESREGSVEDHERHDPQSGGVASHDPNLSPSYMSRERLSRGQSGHPTPILLSKISTSG

GDSVKLPPAALEGGTTPEMAPHRGIITRGKSSSGRADSVKAKFHIGTPKITPEVNTNYSV

MDNSFSVIAPYDSEKDFIAWLDSQLDKVNEFYDQKMNETHERYRILVGQLVRLQRQKLHL

RQRPNKPAREGLSVVERQIELPSLPSFAWLKKSDSNDVCDLDAENTRASSESALRGQNPH

FHDATTDHEGISYASARRQLKTAMQEYYRSIELLRSYCTLNRTAFRKILKKYDKISGRHM

SAYYMDLVDHTDFCNVENSRLDIVAAKVEDLYTNNFERGNRKHAISKLRSTGVNKTYYFA

TFRGGIFFGLAIPFFIEGLYRGCLNLVEHKSPDTQYLLQIWAGFFLILLFLLLFPLCCLV

WNKYKINYTFIFEFSQDHLDYRQFFEMPAFYFFFMSIFAWLTFYSFWESSFRAVYYPCIF

LVFAVVTFFMPLNIFYWSARQWLIRALSRILLSGLYPVEFRDFFLGDIICSMTYSMSNIA

LFFCLYSHEWSEGFHGIYNPSHCGSSHNRLMGFFNALPGIFRWLQCLRRFADTGDAFPHL

ANMTKYSLTIMYYVAQSVWRIDTTNGNRAFFIFFATVNSTYCFIWDIMMDWSLLEFGSKN

FLLRNQLTYKVKWPYYTAMVVDLVLRFNWIWYAIFEQQIQQKQLLSFFVALSEIFRRVMW

MFFRMENEHVSNVKRFRASRDVPLPYHVHVCAEPGETDETAVEEGHQADDEDEDEARDRM

GSSTGVEWARESPGVSRDSPRKSRVTDSPLRKRGSHVEEGNTPSLRATATPVMEAISNMV

QKAHTADFQRRKKPQDDQGKDSDGEDDDDDEDDDE

>tr|Q6C3D0|Q6C3D0_YARLI YALI0F00726p OS=Yarrowia lipolytica (strain CLIB 122 / E 150) GN=YALI0F00726g PE=4 SV=1

MLDYEAIPGTISFVDSSQSDIVLHPTPSCHPDHPLNRSYRRKLRMFSMVTYTVAVTVPSA

SIYSVLTSISHSTGLPLATLNQGTSYMFLLFDLGCSISQPLSHQFGKRPVHLVAVLGTAL

IQL

>tr|Q6C8C4|Q6C8C4_YARLI YALI0D20834p OS=Yarrowia lipolytica (strain CLIB 122 / E 150) GN=YALI0D20834g PE=4 SV=1

MLDYEAVPGSIILVDQDQEIVVSPIPSSDPDDPLNWSRNRKLLSMFCMVVYMVAIVVPSN

SLYSIFSVLTEESGQTLDELNQGTGYMFLFFGLGCLICQPLGQQYGKRPIYLLSVLGTLA

VQLWSPYAKSQGAWIGSRILQGALGAPMETMCEITISDVFFEHERGRWVGVYAFALMFSS

YIAPLVAGFIAEGMNWKWVFYWGSIFNGVCLVFLFFFFEETNYVREHVAQPVPKEGSVLE

GSSGSGDPEKIDDYSEGRSESSLEHQQKPMKTYRQKLALWDKPRKNMLWEMFKRPFIIFF

KFPPVVYAGFLYGSGIIFFNILNASASMILSAPPYNFKPSMVGLCYVSPIIVTFITSWYS

GYLSDLLRIKLAKRNGGLSEPEHRLWIILIHIILNPAMLVLWGVGAYNGIHWIGPVIGMG

IIGGLATIPAVSSVNYALDCYREIGSDSLVTLIVIRNCMSFGIGYGITPWITREGLKNAF

GEAAGVSAACMGTFFIVLVVGKRMRKWTKKDYWNFVQKSIDNGMVH

>tr|Q6C9U1|Q6C9U1_YARLI YALI0D08382p OS=Yarrowia lipolytica (strain CLIB 122 / E 150) GN=YALI0D08382g PE=4 SV=1

MAASTSSSIPDNVEKSASVEKSASAEDYEMVHAPVEENPEFVVDDVKRNRRQRIADGFTV

LAAGFALISDGYQNSMMTMMNVTFQERYGKEVYNSTVSTRVSNALLVGEVIGMIVIGLTC

DYMGRKTAIVVTTILIVLGGIMATASHGVTTDGMFWMLVVSRGVIGFGSGGEYPAASTSA

SEAANEKHLRKRRGAIFVLCTNLPLSFGNPFCVIVFLIVYAACGFKGQHLSTVWRTCMGI

GCIWPLTVFYFRLKMAQPRLYTKSAIKKSVPYWLILKFYWRTIIGTAGTWFIYDFVVFPN

GVFSGSIIATIIKPDEPDKLLKTAEWQLLIGAIGLPGVFIGAWLCENRVLGRKYTMLIGF

ALWIVVGLIIGCAYTPLKRSTGAFVFMYGMLSSSGNLGPGNMLGLVSSEAYATAVRGTLY

GFSAAVGKAGAAVGTQVFKPIQGNLGDRWTFIIAAILGLCGVIVTLFFVPRLNGEDLEEE

DVRFKQFLRAHGWEGEFGTVADKVDDIEETTSDEAHDTDGIETQQIITETPNKMSN

>tr|Q6C5I8|Q6C5I8_YARLI YALI0E17721p OS=Yarrowia lipolytica (strain CLIB 122 / E 150) GN=YALI0E17721g PE=4 SV=1

MDTLAKNSDVTVGAFAVPPRTEYKGMALYIGANIAALVFFLWSLSPTWLLHYLHIYYFPS

RWWALAIPSWLIAAFIFTYVFLTLYNIEVMTYPLDRLEVIVDEHARVDGGKNGEYFWEAT

DGVWDLPLVDVSKVLYGDDSE

>tr|Q6C9P9|Q6C9P9_YARLI YALI0D09295p OS=Yarrowia lipolytica (strain CLIB 122 / E 150) GN=YALI0D09295g PE=4 SV=1

MSIFPSYYQDSSVSFETTGVELELFDDAASEGCIDEQDNPHLERLAERYLDNYEDGELEN

EGFRTDLEELNFQTARRILNQQQLEIHKRMVDEGDNDTINIDDIHQGDDDMPYTTQRRLL

PRHLHMLALGGPLGAGLLLSAGRSITTAGPLGAILGFMLTGLITFACIKSIGDMVTLYPS

QGLPLLADRFLNTSLGITAGVLYWLLFAVAIPSELSAAAALLEVYPSMSQPSSALVAWLT

LFILVIVFANLLSVDYYGELLYWCNLGNLLIVIGMIITMFVVCPGTRFWKTSEGYGAFRP

YYDSYAGFQLPLKGEHHDNQIHGASGRFLQIYASMTLSVFAFVGSESVFVAAPEVKNPRK

AVPSAMRRIAVRIGLCYMLGVISLGLAVPPFNTLGIEREGYGTGNLSHGVVPVDNTGVYK

RSLVDLNIREIGTGSKGSDIAPLLKIHHGHEKATPANEQAPDNIPDRPSPKPHRNSTSTP

NQILNPTFNSPWVVALSLAGYETAALAVNGIFVICAVVASASHLYSASRTLYAVSTRWER

LRWFSTCTALGIPWVAVLFSALISLLSYLVVGYSTRHVFDWMLHWICTVGLILWIIMTAA

FLRFHKCLKVRRVPFKYSFPQPYTAWFGMIGCCLLLCFTIFSVYISWDRRLFASNYSGIF

LTIAIYLVARICTRDDRFIRVSEMDIYSGKRQMDLQVWDDDRVYNSWQNKMKRRIMEWF

>tr|Q6C0T9|Q6C0T9_YARLI YALI0F21813p OS=Yarrowia lipolytica (strain CLIB 122 / E 150) GN=YALI0F21813g PE=4 SV=1

MDIAQIRQAALNSLYAHKDHLDTLRKNTANSTHPPAGDEIRVVDVKQYTQPTTQGVSAHP

VPQGVSLPTPPALPIVTTQPALSPMPPLTRQKPSPYPRQHAPPPPATSKPTFLGLLGGHP

VYQSVFDLRRLGLRFDEILQQSGCSEEFLRHIFKQLGYSIESVVQTPMPPIPTPVAAVQQ

TQIQSQSSRTQPVMTFEQFRKTRSELNTPTPQTPPIGAAAFNPAYVVPVRSIPRFGEKSR

RQKRLCIEISDSESDSASEGVVSKKHKDMSLEETRLEIARIRALIAQQNASSRNGTPSHE

DSEASETNETAAIVEKSPLVEEAAGKLEYVVQIAAAAPKSGSPASPPLPVTPAISPEVQA

YLNSKHIALESLSAVQLDKIVQIVKLKTLLQGEEDAFTTSLKSATQPHSIPQPDIPPPAT

RQPNQSDTEGIDASDRAPSSHSHQQQSQAAHVVSMANTEEQIGVTNELSGVGKRAWRGHG

KNFTGGSGMAKSCIRFIFFLWCMLFFAWCTAIFRFSILASFFSSTSTRSMLLFYFSQH

>tr|Q6C0U6|Q6C0U6_YARLI YALI0F21659p OS=Yarrowia lipolytica (strain CLIB 122 / E 150) GN=YALI0F21659g PE=4 SV=1

MSPEITDQLVRLLTRDDDADAAPVVCDSGNEYDGRMGARISSIFVILICGTFGALFPVLC

SKYSQIKVPPVFFFCAKYFGSGVIIATSLLHLLQPANEALSQECLGHWNDYPYAFGICLF

MVFFMFAVELVCFNMFGHQGHSHGPVGLASSKDVEISGVHEHTHEGHSHDDHSSDDIVKE

NEKPREHSSVPVSMPNPIANHDPLTPKDHYGHCEEHTDPNDVDLENDMGLETYSAQLVSI

FVLEFGIIFHSVFIGLTLAVSGDEFKDLYIVLVFHQMFEGFGLGTRLATAPWPKKKVWTP

WILGLAFGLTTPIAIAIGLGVRKTYPPGGKTASITNGIFDSVSSGILLYTGLVELMAHEF

LFSSEFKHANNWRIFWAFAWMCAGAGLMALLAKWA

>tr|Q6C1I7|Q6C1I7_YARLI YALI0F15917p OS=Yarrowia lipolytica (strain CLIB 122 / E 150) GN=YALI0F15917g PE=4 SV=1

MGLYGFFVSAPLSHYLVGALQAAFKGKKGIQWKLAQILTSLLTVTPITSTVFLVFMSIFA

GARDVKSVIASLKVSLLPVLRSSWVASPIVLAIAQAFIPEHAWVPFFSLFSFLLGTYNNY

IVKKKRRLLREGKKDDKLQ

>tr|Q6C3U6|Q6C3U6_YARLI YALI0E32035p OS=Yarrowia lipolytica (strain CLIB 122 / E 150) GN=YALI0E32035g PE=4 SV=1

MHVPVLGRLELSHWPMLFVSLFLVCLEFLVSFITALLPNFFIEWCHDKVEILQVYFPSTG

EVALRKSPEQLETVNALLKARDFVEICDFHGYQAEEHVVQTKDGFLLGVHRILPKNPAAL

LTDDPEVLSTPAPPPAFLAGNLEVYSSPPKRGSKRSVSSKLITRPVVYLHHGLLMNSEVW

VVNTDAKKSIAFALADLGFDVWLGNNRGNKYSRKHMKYNPESREFWDFCLDDFALFDIPD

SIDYILSVTKQKSLSYIGFSQGSAQAFASLAIRPPLNDKVNLFIAVAPAMSPPGLRSKIV

NSLMRASPQLLFLCFGRRAILSSSPFWESVLDAKLYARIIDAACRMLFDWYGENITWPQK

IAAYHHLYSYTSVKSVVHWFQIIRAASFHMFEDVINSPLDPHLCKYSTVTRYPTENIRTP

IALIYGKTDSLVDIDQMLSTLPSSTIAFGVPKHEHLDLLWGDEADTLVIPKIVALLDYYT

PVPRSNKSSSDHISERLFEPANSKVSGLGLSQDLKSPLSLKKRSLSNSSSIQDSHKVGGD

TDSVLSLRNLTPKGYALGSAKPVSGTLNP

>tr|Q6C4K8|Q6C4K8_YARLI YALI0E25916p OS=Yarrowia lipolytica (strain CLIB 122 / E 150) GN=YALI0E25916g PE=4 SV=1

MFVSAKTFIVARSTFMWSLAYFLWTDPTVIIDNMYVNIVGQAMQLPPLKLENAEPLLGLV

AYLLTMVGVSDVAPLNGPYLEYFYSIMPLRVITLFAIIAYCAYGNSIYVSNSMVFSIVFL

DLLFSFFTYLALREERNEYLKKKILAVQATQKDQEENNLVS

>tr|Q6C9N4|Q6C9N4_YARLI YALI0D09735p OS=Yarrowia lipolytica (strain CLIB 122 / E 150) GN=YALI0D09735g PE=4 SV=1

MFNLTDHKFRERVEDNVRARSALLRNSCSTCSYQSVDRRAYGWRSTSSKTRLFSRDNLRN

ISYTRLIGGMALFVSLSFAINYLLLMSFGASPVPGGLTGGFRLTPMVPGTAPPGGAPILT

NTALIISPNSDFVVPARMAAFGRGLMMDEFETTVDETEKQARDAQETDKLAHDIAEHFPT

GISDGTPILIPPNRRKEPTVNAPLRGEIEVVGGDACGSGKHRNLTDKIALVMRGGCSFYD

KVLTIQGWNAKAVIVGDNQYNRGLVTMYSTNDTDMCQVPAMFVSRASFELLSTEDEVSII

PGPSATPALDTILFLLISPICSLSIIYLMVSVHRYFTQLTRRAPKRAVKQLPVRVWMGQG

VHSPTVRGKTQGNNSSSGVLESVEPTSTAAAALEDSPEPPSTHSDKVWVSSDECIICLEE

FTVGESRVMQLPCGHDFHEECIQRWLTTQQRTCPICKHDITQPMSSPGETTPLV

>tr|Q6CBA0|Q6CBA0_YARLI YALI0C20779p OS=Yarrowia lipolytica (strain CLIB 122 / E 150) GN=YALI0C20779g PE=4 SV=1

MLTHYFIAALFLATFVWAGSALNNPITGIKAQLSNNRTGPGYYWDTWEVVVDFAFNKYSL

EDIRGGDFFGFTLAGKQLNPRGPKDKVPVDYDFYIVNYDGRRLFHVTTTSDSYNFRATAT

SVFDNPSMEVLELSGQLTVEFLVKPNSPVGLSDVSIGPWSSSINLEDPDMADADAGACWW

NIDSYQPANNGNSQKICAQQGLPYPPSGYGRAYLKTIIHPMYTKVVTGLTAGSETLVDDR

NIPYERVTTTPASTITVAGYETGLEFFESVGSIFERTTETPAYTTTVEGLKAGSEFLTNS

EHVLYEQVTAIPAFTSTIAGYEAGSQLLVDPLNGVVFKQITITPVSTSTVVGHVVGTELL

NDSEGILYEQITVTPAFTTTIPGHQAGSQFLSNPVDGVIFEQVTETPAFTTTVPGHRAGS

QFTVDSNGTIFEQVTVTPVSTSTVPGHKMGSEFVWDVMDGVFAERVTVTPAFTSTIAGHE

AGSEFVTNGTHVLYERVTATPVFTSTVSGHRAGSEFTADALGNIYERVTIAPAYTSVVPG

HEPGSEFLDGSDGNFYERVTTTPARTVTVPGHEEGFQFLSDFNDGEFIEQLTITPAFTVT

VTAHKAGAEFVTNADNVLYEQVTVSPASTTTVVGHEAGSEYLQDPEGVIFVQVTVTPAFT

STVTGHTAGSEFRTRGDGVFYELFTATPVHVTTVTGHMGGSEFLENLQGDIFEQVTVTPV

FTATVIGHKNGSEFLKDSNGTIFERVTVTPAFTSTVTDREGGSEFSTRADGVLYEVTTAT

PVYAATVTGLAGGSEFLEDSQGVIFEQITVTIERGTVAPAYTTTISGHDADSQFFTNDSN

VENGKDTATSALTTAVTEHEAGAQLLEEFYGKFYENVTATPVVTTSEIESRNGSESLRES

HGIIIAQVTVSPVTSLVVSSAEGSASAEEKEILTSLSNPNVSISISDGLSPSTSSKADDS

PSVTSTSSVSSFSTSVSSPGRSAVDTTQSNNSSISADDITTSGITSSFRTSFSTINVSSA

SGDISSSSVSSYTTSVSSSSENATSAAKTLTSCGSSVSLPVSSIISVITSSSPSILSAGN

STDGIVTSQTTPLDNVPFQNASFASNEGPDSSTDGSSISIDNVTATLSTAAVTSRISTAL

STVAVTSQASTAVSSSVSSAQVSSSGVIYNGGGVSDAQSDHIDRSSRFGESHGVHGDKLE

GWKDFSDVSNHVTGPFGYADGITELSDGSGTAGYGGTSGSAGGNSDSHSNDGSDQPSSGA

TYNFDPNSASSSSVGALLALTFLVMLV

>tr|Q6CFN5|Q6CFN5_YARLI YALI0B05390p OS=Yarrowia lipolytica (strain CLIB 122 / E 150) GN=YALI0B05390g PE=4 SV=1

MSENQPLLGAPTRGPLKTHVIGPSASSNASSSATARKRPPVDQSTPPQLNHRIGPQRTSR

TTQKLKLLPEEPDRDDDDESGREVYSQVTRIKDPPARKDAERLGKAHRDLLPRVTAYCTA

SSYRMRDITKYLQSRKHRRAHPKLFDECLYTPYSYRDQEISAQEDFIRIDDEGGDIDVVI

TRTDVFLFEYGVVVLWGFTEAEEKRFLKELARFETEKLADEDVQVEEFNYYITQSYQPRI

YNDFITLRDGRNYMMKLSISHAIAQSAKISLFEELVENTIEDTQDFPQEIAVTGKIDMDR

SDIIKSIGELFILRININLHGSILDSPELMWAEPHLEPIYQATRGYLEINQRVSLLNNRL

EVISDLLSMLKEQLGHSHGEYLEFIVIVLIAVEVVVAILNVIVDLTAENL

>tr|Q6C6K3|Q6C6K3_YARLI YALI0E08888p OS=Yarrowia lipolytica (strain CLIB 122 / E 150) GN=YALI0E08888g PE=4 SV=2

MSQACPPQDCTKCASNEVCVLGVASQDNPCPSITCSAAMANTNTDSGKSSSGTSAGAIAG

GVVGGVAAAAIFGFFVWFFCFSKRAQRVREEKEWIPVVGQDHVTDNHTAGQMPPNHRNSS

STFASLASSALSRASNVIPIAYVPGVTSRGQMSRDSSTGGTLGSNPPIPNVPWEYQFTPE

ELLRQSRQSTVYGIRDSVNTEAYRESQAYVSSAMMTAIQAKPMLVNVKDGHVTNNDGSAR

DSALRQSFMSGTSDGSVYEVASAERVNARSIRIGKAQRVGLQSTYISEESEEEIGGGASR

TRDSTHSAAVPIGLDRPTLGRSLTGNSGSGVSGVSRKSEVPPLPAALEVSSSEAPQLDED

IFAGLDIPVQLITTDDGQESPFDDKFRL

>tr|Q6C6R9|Q6C6R9_YARLI YALI0E06809p OS=Yarrowia lipolytica (strain CLIB 122 / E 150) GN=YALI0E06809g PE=4 SV=1

MAPMIQLCPQAMAQPLISFSKRGLAESAKSTGEAFKSWDTCMDNTFCKIVAIVGIVLGGM

LVLWVLTAFFRCCFFGVSAFSACCALCSCCACCCNDKINREANQGNYAAANNPAMYPASN

PYQAYNYQPQPVYGNQSNNNFAPDYYVNRSVDDTDYEMEKYPKINTYEVPAEQERTRF

>tr|Q6C8E6|Q6C8E6_YARLI YALI0D20262p OS=Yarrowia lipolytica (strain CLIB 122 / E 150) GN=YALI0D20262g PE=4 SV=1

MLPGGIPYPPPVDNGYWGPTTSTIDWCEENYVVSKYVAEIMNTTTNAVFMIMALYTIINV

YREKHHPTIIFAAIGFFIVGFGSWMFHMTLWYEFQLLDELPMIYATCVPLYIVFSNKKSN

HFKTLLGVGIAAGALLLTAIYLHNKNPTFHQAAYGILNFIVIGKSVALTKAYISDQKTKN

LFWRLLALGLFSFLFGYFLWNLDIHLCNQWIKIRREVGLPYGLVIEGHAWWHIFTGLGVY

IYIVYLCYLQVFLAKRQHLYHFLWWCGFFPHVDLLPEAKSLWNKLGRAPTETEMAEVNSL

SGVKRVSSIHAKSSSVNHHN

>tr|Q6C2Z8|Q6C2Z8_YARLI YALI0F03795p OS=Yarrowia lipolytica (strain CLIB 122 / E 150) GN=YALI0F03795g PE=4 SV=1

MFGSLGILQPRDYGGEVTGTVVMMGHRDKESKLMTRNDGDGVGEDADAANATQGDSSDTT

PSGVVTDPATVARAKASSESDSSDLKKTPGGIVLFPQPRNDPNDPLNWPIWRRDIALLVI

GFHSFISGGQTPILASGFNIMAKEFDVTLNTLSYLVGAFMLAMAVGSAILAPTAVIYGKR

MIYLISCLIFFGGAIWGGAAKSFGSLIGARIIMGIGASPTESLPSSSIAEIYFMHERAYR

LGIYTLLMLGGKNIVPLVSAFIISAKGWNWVFWVLAIIVGMDFVLIFFFVPDTWFIRAPT

PNKRSLEESMMAQEARANSLMSWNSRQSMRRDFLDEINVQEANEELREMEETAEKDAEKE

AADKEAREDATNPTAPALTREVSFADESTAPPADEPEVDLPIPEDGVLGSSTPPRPPMVK

KHSILRDHDTPHDHHGPKHTSFAVEDQEEGDGDFPGFGPSTSAPGRPSYISRNLSYASHF

SVASQDVPKKSYIQTLKPYIGRQSQDKLWMISLRPYVLYLYPPVMFSTLVYSMSVVWLIV

VSETISHIFSSQPYNFPLTSVGLLYVSTFIGGCLGSAVAGKISDMFVRIMCRHNNGVYEP

EFRLVMIVPVMITTSMGLMGYGWATHDGDHWAVVCIFLGLLGFGCSLGSTTAITYCVDSY

KMFASEALVSLNVSKNVLGFVFSLFNTMAVESRGQKTVFLAYGGAQIFLCLFGIPLYIYG

KRFRRWTDEMNLMKYLYVRTEDDADDADE

>tr|Q6C4W2|Q6C4W2_YARLI YALI0E23243p OS=Yarrowia lipolytica (strain CLIB 122 / E 150) GN=YALI0E23243g PE=4 SV=1

MVAAAGGGSSSARKPYKGGNDMGAFFEEVDDIKKSLLQYDENIDTIETLHKRSLNEISEE

QGSYTQDQIQSLSNESMSLSQSLKDRIKNLQKYSSGDSTKKTQAENLKRQFMSSIQRYQT

VEATYRQKYREQAERQYRIVQPEATDAQVKAAIDDAQGEQIFSQALMTSNRRGEAQTALS

EVQNRHREIQKIEQTMAELAQLFHDMEILVAEQEAPVQHIDNQTQAVQTDIEQGLGHTNK

AVIKARALRKKKWWCLLIVICILGISLGVGLGVGLNNN

>tr|Q6C6E3|Q6C6E3_YARLI YALI0E10197p OS=Yarrowia lipolytica (strain CLIB 122 / E 150) GN=YALI0E10197g PE=4 SV=1

MLQYAIPAFLVLGLALVFVNSSAKTLAIFAWNCFIKPFTGHKDVAAGQQGALESFYKGQA

NIYDRTRATLLKGREQALATTAAELRKKHDLVWVDIGGGTGWNIEHMTTNYLPISCFKAI

YLVDLSPSLCEVARKRFAEKNWKNVHVLCIDAADFTPPAGSSVDLFTMSYSLSMIPTYYA

VIDRLSTLLGKDGLVTVIDFYVQSHATLTAKSTTMGGELLRHVNWFSRTFWRLWFEFDRV

YLDSARRDYLEYRFGTIKSVNCRNTRLGGIPYYYWIGCDKDRSTNILQRANALATESPYL

SPQNELVVGDEDAANNTLAIRSKGYDAALVNMQRNFPAPSFFYQTEIWRIFYNQELPKYH

QFANQYIYAFTWEDPREDKNILQFKPSDTVLAITSAGDNILSYASMDAPPKRIHCVDLNP

CQNHLLELKLACLRVLPFEDMWKLFGEGKHPKFQELLTTKLAPHLSSHAFQYWHKRGHAS

FTGKGLFDTGSSRWAIRLAHWVFAISGVSPHISALCEAKTLDEQWSVWEKSLRPCLSNPI

VAKVLVGNPIFLWKALGVPVEQTSMIEGGMLKFVIDTFEPIIKRSLISDDNYFYYLCLKG

CYAANNCPDFLTKHGHANLAKRKGALDGIRIHTDEINEVVKRLNPGSVNHAIVMDHMDWF

PKDGNAAREEIKSLHHALTEGGNVMLRSASQKPWYLSVYEEEGFTTRPAAIRESGTSIDR

VNMYASTWVCTKKGAMESLKI

>tr|Q6C9D4|Q6C9D4_YARLI YALI0D12100p OS=Yarrowia lipolytica (strain CLIB 122 / E 150) GN=YALI0D12100g PE=4 SV=1

MSTHSVRDSVTHSLKEDAEIHIQSVHPTINEKKVTRKIDLRIVPILSILYLMSFLDRGNI

GNANIEGLSTDLGLTGPQYNMCLTVFFFTYSVFEVPSNMMLKRLKPSIWLPAIMVAWGIV

MTLMGIVQNYGGMLATRIFLGLTEAGLFPGVTYYLTLWYPRRDIQFRTAMFFSAASVAGA

FSGLLAFGIGKMDGVGNLEGWRWIFILEGIATTLIAALSFFLVVDFPDTAKFLTDEEREW

VLWRLKYDTNSRKTNSASQQAIMVEENDSHSWDEVKKAIMDPQLYPQCLLYSAVLVPLYG

VSLFLPTIVKNLGYSSEGAQLMTIPIYAVGAIASIVQAWVSDRYGWRSPLLVFNFCCMFI

GYMLAMNVSAVRNPSATYAGCYLVALGLYPGIPAVISWLSNNTAGTYKRGVSMGMQIGFG

NLCGAIASQIYRSKDAPQFKLGHGIEVMFICIGFICIFVLNLYYYLVNRNRRRMVAEGKA

DDLTVTQLSEMGDKSPHFIYSH

>tr|Q6CD37|Q6CD37_YARLI YALI0C04092p OS=Yarrowia lipolytica (strain CLIB 122 / E 150) GN=YALI0C04092g PE=4 SV=1

MSVCLAQNPTVTRVVKLLETHVGRDKILRSIQYFSRFLTYYLFRKGYTKDTIDIFRKIQN

QFSMARKLFRVGKPIGHLKTAAVSFENKTLDPCLRYTTIGRNLGYAIYLVFDSIIYINGS

GIKKIDNIKTIKKVGSYFWAFGIFCNILNSIHKINICKKKRAALAAEKEKDTTSAKKNDK

DAAAAQKQLVWDLLDFSIPLTSLGYLHLDDGLVGLAGFATGIMGVQKAWAATA

>tr|Q6C1T0|Q6C1T0_YARLI YALI0F13651p OS=Yarrowia lipolytica (strain CLIB 122 / E 150) GN=YALI0F13651g PE=4 SV=1

MNYALIGITTAICGNIIISVALNLQRYAHIRLEADVSSPHYTSSKVWWCGLALMTIGEAG

NFLAYAFAPASVVSPLGVFAIVANCLIAPIVFKERVKWSNMMGVAVTVVGILFVVLSATS

AQSDTRPVEPRDPHAMIMAALQQKSFLVYIVFVFVSATLLLHFSRQQLRQQTALFVYLGL

VALFGALTALSTKAVSSLLSFAFLRALYDPLTYACAFVLAATAVFQINFLNRALQTFPST

VVIPTHFVLFTLSVIVGSAMTYHDFDGMTLGQITCFVGGCIITFGGVTVIARTAPGRPRL

QQNPSYSSFSTRSPTPTESLAIPSEVSGLLEVPPDPTHQLASSAPAPHHHHHALRRTRSA

HLAFDPIALGGISFFVDSAREWRSQSLGRGEREEP

>tr|Q6C6U0|Q6C6U0_YARLI YALI0E06347p OS=Yarrowia lipolytica (strain CLIB 122 / E 150) GN=YALI0E06347g PE=4 SV=1

MKLPSLATIACLVLPVLADYGLSEMYELSLPNSMASDDRFKTHFYASGSAVLTSSGPGGR

SQIALTELGRPNQRGAVSGNQPIPSKDFITEVSLAVYGPVLPGGGFGIFFTPQPYQSGPV

YGMKDKWNGLALILDSVIGNDGEGHLHVHDNTGGDYAAMDASQVEKDALALCKLKYRNTG

APVKITTYYINGKLKVEINGHKCFDERPVALPDSPYFSISAASTEGPDTFEVYSLKTYRV

DPNGVGGAPTENTPVEQKQEAPQAQQQQQQQQQQQQQQQTQQQPPQQQLQDVATSEHITE

LAKKLISVEEKLESFTDLKKLSTLSNDLAVLTNRIGVMDQKLREMEVEGKEHVRERTQDT

KNYITNHLQEIKSQLHSLTQTFDSQVKNELRSQFTDFSSDKKNQIPSLWIPLVVIVGAQS

ALFFVYLKSRGKYEKLL

>tr|Q6C9Y7|Q6C9Y7_YARLI YALI0D07304p OS=Yarrowia lipolytica (strain CLIB 122 / E 150) GN=YALI0D07304g PE=4 SV=1

MTADVFSVEVFFICFRECLEAAIVISVLLSFVKQGLGGGEDKTLYKKLLKQIWIGAAGGI

VICLCIGGGFIGAYYSFEDDIWSKSEDLWEGIFSVIATILITVMGLAMLRINKMKEKWRM

KIAKALLEDHQQVKGFKKFLNFRFYSKKYAMAILPFITTLREGVEAIVFIGGVAVGRPAT

AFPLAVFCGLLAGCACGYLIYRGGNMMSIQIFLIASTCFLYLIAAGLISRAVWYFQMYRF

SLKTGGDVAEQGSGPGSYNVHEVVWHVNCCNPETDNGWEIFNALFGWQNSPTYGSVIIYN

LYWLCIIGAIFAMLFQEKHGYYPGFKWAKRFQKKEYSKEEIDEMFHRAAATQINEAAQYH

AGSSSEDLDKGVNDVESKGVDDISPGSSNPEVSRVQIEMTDKSQEK

>tr|Q6CA22|Q6CA22_YARLI YALI0D06545p OS=Yarrowia lipolytica (strain CLIB 122 / E 150) GN=YALI0D06545g PE=4 SV=1

MNIFRLAGDMAHVASIFILIHTIRTRKSTSGLSLKTQALYLAVFVARYMDLFVFSDVRAG

RYYNVLMKLLYLGTSIYTIYLMTQKYSNEQTNRHIDTFKVEYLVGPAVVMSLIFNDGYTF

LDILWSFSVWLECTAILPQLFMLQRTGQAENLTIHYIAALGVYRALYILNWIYRFWTEDK

TNLVAFIGGIIQTVIYSDFFYVYYVKVLNGEKFELPV

>tr|Q6CD20|Q6CD20_YARLI YALI0C04455p OS=Yarrowia lipolytica (strain CLIB 122 / E 150) GN=YALI0C04455g PE=4 SV=1

MQLTSETIPRYTVLTRYVQTGLSLLCWCLAISAVALIDGGYWSTDAYGIAVGILTFIWLA

YVMFVPARYPKYANGLAAVVGEVLINIFWFTSFIASAAQRGPESCNFRGWGNRYLKDWGS

SCKVAKAYIAFAAITWLFFIFSTFFVIKTSIPHFKIGTNAVINAPVLVGGTLGAAVASDP

EDPEAAERDLEGGLQPEETVHVSDEAEKEITVDPQVSQVDPHVSQVTTHTQDQETAQDLG

TVKMPDETYQPYTSTLDTHNK

>tr|Q6CEW6|Q6CEW6_YARLI YALI0B12342p OS=Yarrowia lipolytica (strain CLIB 122 / E 150) GN=YALI0B12342g PE=4 SV=1

MAKDKEIDFDYTGELVMDDFEFPIDDMLHNDGDDFVKKETWDEGFGFGTNGAVGAQMDVQ

TSPFSDPVFGGVGAGPDMMGLMDTNMNHINGSHNMNSVVKQEDYYTPSMGTPMNPQQQQS

MTPQQQHHMNHNQPSQLQSLHQQSQKAQPQQQQQQPHQSTGVDSIITKAYTRAAGDLPYG

RKYSRQLNKYPEDVEYSSFDPSLWSNLLTNSETPYQYQIHVHSMPGKSRVETQIKCALSI

YPPPPQQSVRLPTDTISRPKFQLKQGHIPDSCLSLEVYIVGEQNPSKPVNLCSRCIKREQ

KRACRKKLFDESEELSWVETRQRRLAVFNCSEVLEFKDVERRVYIPESGTTVTAKQLVLP

LRLACYCRHHGEKKGFRILFCLRDEGGQIVGVGQSGTTVMITDDHKVVGDAVAMPTTATA

PATAGSSQPPTQVPTPAASSSTSYRPRNSLPLSPTSMEDSSSEFTSDHSHYSNYGSKRRR

DGSSISDWSGMMNVRGMDRQASITSIPEMVGGMSNMTVASASGSATNLAAHNMNNPADEN

LPVIKRIIPSQGSIRGGIEVTLLGSGFKSNLVAVFGDNKAVGTHCWSDSTIVTHLPPSTI

VGPVVVSFEGFVLDKPQIFTYFDDTDGQLIELALQVVGLKMNGRLEDARNIAMRIVGNNG

GVAGAQGAMAGGNMSNGDVGMESAAADSSVQPVSPPTDHEDVVLRCLALTDIPGGRIANW

QLTNAEGQTMVHLASILGYSRVLVALVARGARVDVSDNGGFTPLHFAALFGRRKIAKKLL

RCNADPYKRNRIGETVFDVACPHILDLLVGPQGMPMAVQTSYTPDYHRQRRSSSSSTLAS

IASIQDSREYGFYDHGMISNLSHIPSTCSIRSSTSQFDAEDEWDERDEEDGDFDDDSDED

SDDDSDALFMSVRKHAKAKSVESPLSEEEERLVRHIEAEDQAVEARVAAGIVSSNVPDVV

SSNDSDHVRSDTSTENKSFSRYFDRTLSMASWDDVLAYIYRPKRATVPNKRSSGAPPSVR

STRSPLSDHPITSSGDESDRTISAHAPSGGAGRGRSHSSISRMWRYLKNSSADEATRSRS

RDANGAGAPPAYEEIFPGHGVVHDKKVVQMAAASAAENSSGPVGASSSAVASTSAAAAVV

PSPLAPIVEDEEQLVEAWRRQRRSMANDRMLFAFWLPVLLMAIGYMVIKAFGLFPDQVSA

VESVAETVGVHCRGAVAKLWFKQYPVHRGQPLKDTCSFEPNSLVESALRQMNGWSDREVP

IHQAQAQAA

>tr|Q6CG69|Q6CG69_YARLI YALI0B00396p OS=Yarrowia lipolytica (strain CLIB 122 / E 150) GN=YALI0B00396g PE=3 SV=1

MSHRPWDCLPLWNTVVVFRRCVLWSLVMCFVIVMDGYDGFLIPSFYAVPSFQRRYGVQLP

DGSWTVEAKWQTAYMVGAPIGRIAGALGVGVLADRFGRKRVTLVGLFCLSGITFIVFFAV

GKAMLVVGWFLSGLIWGIFNTMTPTYVSEICPVSLRSTFAAAINLSWVVGQFISTAVITA

SESRQDEWAYRVPLAVQWVFPVVLIPLVMVMPESPWWILKHGDKKGARQVLTRLMDEKDV

DIDMYLEYMRQTLEDEVDGGSFVDCFKGSDVRRTEICCLTYFFQPLSGLYILSYAAYFLQ

LTGIPQNVVFKLTLGLTGLAVMASLVAPIVILTFTRRSIYMGSLALMAATLFAIGITACY

DTQAAKWAAPVLIYVWVGTYDATIGPLTYVIVSETSSVKLRSKTIALASITNSIMVLVLH

ISVPYMMNEEEANMDGFVGFIYAPFCLLAIAWAWWRLPELKGMSFMEIDMLFGNETGTQR

APLVFDGQKRERQPLESLDGLGWRFGGQQDLSETNDNNEGSREERLSNPYCSSNEDNRSD

RFQPSERGCILDSPKRKHAIEKNGVVITETLEMGENADR

>tr|Q6CHB2|Q6CHB2_YARLI YALI0A10593p OS=Yarrowia lipolytica (strain CLIB 122 / E 150) GN=YALI0A10593g PE=4 SV=1

MAILHKPSFHFQKATFENPFHRSPEEKNETWIKYIFDPHVTVVTTKTHDHSNNKADGAKE

IETIKQFMDKKMLFFAWISLMLVSTVVALERGTTSTYAAVATSHFNELGLLSAVGVVRSV

LYAVTRPTIAKLADVLGRVEALVLAIIFYIIGFAMLAGGSNIATYFAAHIFYVSGEVGIQ

FMEAVFATDTSNLRNRTFFLALPNFAYVWASWVSAPIASGVLKNSTYRWGYGMWCIIMPV

VSIPVCIILLRLKFKARAMGLEYGKAVRNFRGTFSQFDMVGLILFTAGLCLLFLAVTLAT

SPTSWGQAHILVMIIIGPILLFIFPFWEIFGPKYPFMPSYVITNRTMFTTCLLIFCYALA

YFLYGTYYLPWLMVCKNLSVKAATNTVNVFVVGSTVSSFFAAAYAKYFKRMKPVIIAGAA

IYMLGIGLTYNYRKPTHRLSQFIVSQCVEAVGAGFLQTPMISMIHAVVPHHNVVTATAIY

QSIMTVGSIVAEAIAGTLYRQSYPKQLAKHAPFLSPREAHQVVNNLMAPIKLYPWGTPER

EAIVTAFNDIYRKLLYPPIIIAGFIFVAAFTLPNVYLGDTDEPTQSDSEVDSSSIDEKIH

PHDEEAQYEQHHDMEASTSPENIPQISEGMEEEKKM

>tr|Q6C460|Q6C460_YARLI YALI0E29447p OS=Yarrowia lipolytica (strain CLIB 122 / E 150) GN=YALI0E29447g PE=4 SV=1

MNHRLPLNQSDVRAGRSGGGPMQMLRRIFRPRTLDFETASWEIFYLIFRPKRVYKNLYYH

KQTKNKWARDDPSFFILLNVLLLISALGWGLAYQPGIVRIIRLMFYMVLVDFLLLGLIIA

AVFYFTIRKFLTKKGDQFSQGALEYAYCFDVHCNGFLIVWLLLYVLQFVLLPVLTKDNWL

ALFVGNTLYAFSTCYYFLVTFYGYSSLPFLEHTEFILLPIPIIIVFYIASLFGFNVVQHM

VEFYFGK

>tr|Q6C5T4|Q6C5T4_YARLI YALI0E15334p OS=Yarrowia lipolytica (strain CLIB 122 / E 150) GN=YALI0E15334g PE=4 SV=1

MAEDIPLEEVEGSGRRWSSNNPFLDPGSGTRRAASRSNSSLSSVESGSTTDNPSIASIRQ

GLTTALGEGGSGGEWLTSEQIHNDEKLSPKKRRTSVFEESPKSTKGGLKRPEVGWNPSSS

DYLTPNRTVDFELESLDNIAYTPKRYTSFDSADPMMHNDLRNTDLERAVSRNSPKSKDNT

GDKWNNLTSAVQRVSYRIISGMEQIDPDEIVTDEEDRESSDGTIDGDEEDPFTQYDHLRP

SSPLRPTSPVRPSPHRFNSNTSSRRPTTPKNDYDDPSQYLFGRSLRIFPPQHPWRWKMWK

LCSSPWFEPFVLLLIILHTVFLFCESSWNIFKDKDTEHIFASWGHKWTDWGFLAIFILYT

FEISAKIVAFGLWDDSQMFYSTGQMPDSSWNRFFPNKLTKSSRWNQRKGTSSAPISHALT

LLVHNEQPTHRVQRAFLRSSWNRVDFISVVCYWISLGVAAHDYDIIHQLFLFRFLACLRI

LRLLNLTHGTTAVLRALKTSAPLLINVSLFIGFFWVTFSIIGTQSFKASLRRQCVWVDST

GQQDNYTYEQYCGGYLHPETLEHMPYVFTDGTVGPQSKGYMCPAGSICVSDKNPYGGTVS

FDNIFNSLEMVFVIISLNTFTDIMYYTMDSDYMTACLFFMAGILILALWLANLLVAVIAT

SYSQIRVEMILSRKSKTETLFRKRLTSAEARNFLKKTVKGRWYLRMEWLWSLIIFMSLVV

SSTRTADTTDSQETALYILEIIITSMLTADVILRFYVYLPNWRIFFLNIANVVDCALGVM

CIVILIPPIHNKPVLYGWLTIFQILRIYRVVMFFKFTRLLWRRVVGNYRTMFYMSCFFFL

LTFLCSVMSTRLLRGVIPQEDDSGESIEFSFYNLQNTFLGMYALSTTENWTDMLYNGQQF

ATNTFSALCIAAFFIGWFIFSNNVVMNLFIALITENLDVSEETKRIEQIKGFVRKYTDQI

SNNGGSLPTMQLLMQKVKHLGKNREINQTDGSHVFDMLMQRHVVQDFLEDEVNEAEEMPA

EQRSRPSRIKRMLKQAHVKSLMFWRKKEPTENPFLDPPQGGDFFSRKKSLNPINLAKDFL

DDYQQRLKTQEEFLAEHPDFNVVLYCLPPHNPIRRFCQRIVMPSHGTRHDGVIPKQWVWY

TFTFIMFLATVALVVLACVATPLYQKENGGGGVWSWITWSDLGFLILFSIEAIIKILADG

FYFTPNAYLRSSWGVIDFIVLISLWINMIAIFAGNGYVSRAVRAFKALRALRLLNISNAA

KETFENIVIVGIRKIFGAAMISMCLLYPFSVWGLQIFKGRFYSCNDENTEFLANCINEFA

SSPSNWNVLAPRSVVKPYYDFDNFGHSFLILFEIISLEGWTDVLKTTMSIQGPGMNLEWA

ASVNNSAFLVLYNFISTVFILTLFIAVIIKNYSRTTGTAFLTQEQRAWAEAEKILKLVKP

SIVPVYVREGTWREYCYRVVTNRKGKWNKFLAGLLFIHLIILCLEWYPQYLYAEDIRNGL

FLPILIILDLNEIMRFYAFGYKGFMSRKWDLYTVLVVTGALVTTIFSFASFVSNTYYNFQ

KLCQVGILLLFIPRSERLDQLFKTTSASFASILNLMATWIVLFVVYAIAFNQIFGLTRVG

PNGTGNINFRTVPKALILLFRMSCGEGWNQIMSDYLLEPPYCINGAGFNETDCGSRPYAY

LLFISWNILSMYIFVNMFVSLIYENFSYVYQQTGPLSRAIKREELRKYKKAWREFDPTGT

GYIRPDELHRFLRRIEGTFSMCIYEGDWSIKELISDHNIRVNDHDPYDVDLPALNKAVRT

IPVGDMINRRRAFDTLCEQVLLERNPDKGISFSQVLLAIPIYKMVDENKCFMLDAYLKRR

IMLQQVEENLLKAKLRGFMDMVQLRREFHERRDDMGPMSASRDPFESTGVTRIPSGSRKN

IVPQIFITTDGDSKEDFTSEEPSLLPRKTADFDVEEVPTTPDGVPVPADYFLSTDPMTPN

PNAGSSGRFSWASERSNIDRNSMWDRRVSGDSDNFKDQDSSDLNLIHRQEDQLIDSFENS

VWGGALSKRASRYQRAEQDDEKDETLDDDSHDYI

>tr|Q6CAV0|Q6CAV0_YARLI YALI0C24145p OS=Yarrowia lipolytica (strain CLIB 122 / E 150) GN=YALI0C24145g PE=4 SV=1

MSKPQIVKQETITSPDGLTSTTTTVDSDGVTTTTSTCGTVSTTTVVGPDGTHTASEGDAA

FESSFPNLVKIPVGQPQEANGTTPINRRGANPSSSVRNNYKHPDLHSELTAKSFGNGMFS

LLDLSLIGLVFSVLILGALVGKIVDHTYFADKRNFLNILFVKNGWLWTTIAFGYIVYETF

SGSIGLGVSFGTNTSTGTRTGTTHDGVSETNSDSKANAALKLLVGISAPTPIGQLSRYII

HSLWWLLFTQWFLGIPIMDRFFVATGGKCEYQKENASPITGKTISSASCRRSGGTWIGGY

DPSGHCFLLVLSTMFLVYETIPHIKRRPYKLSAKIALGTAALWTWMYFMTSLYFHTFWEK

LMGVIFGLLTVQLVYVVIPYLTRPKPSVGSNN

>tr|Q6CBR6|Q6CBR6_YARLI YALI0C16225p OS=Yarrowia lipolytica (strain CLIB 122 / E 150) GN=YALI0C16225g PE=4 SV=1

MEKIGHKQKIWGERLEKLICFHPWVRAIFFSKAQIKHYPKRGSSTNAKERGWTRPIIIVG

WIFVYLYAASKELAEKVHPTMAPLATSHFAGLSLLSSKAVVQSSIFIVTRPAFAKVCDHI

GRLEGWVIVVLCHLVGSVLFASAPNTGCYFAGIVFWEVATVGGHMMTELYASDTSTINTR

IIFSSFIQSPNIWGPYAAAPIVGAILDVATWRWGYGMFAIVIPVCSLGVLFLFSVMRVKE

FRLGARGDFYTPGGFKRFLLSFDIVGLVMLCAGTILLFFPITLAEGTDKWSRPGFIAMLT

IGSFLLAAFPLWEIYGAQSPLISWKLFRNRAMICSCTILFFSSMSNKLSVPYYTSWLLVV

RGYSPKATTNINSAVTVANNAFGILFAAPYLLWSGKPKHMMVLGTCLYLVGTGLTYRFRR

QHSGLGILIISQVIEGIGRGTLYIPCGTIAQAMFDKHKVAKVTAVYFTMGGIGQLVGDAI

LGAIYKEVYPQYIHKYAPDIPQKDLNLIINKIKKATKFPIGSPVRNEINRAFDETMKHML

YGTFACAAMMFLAVLCYPSVDFKEQGSVSVDEGEDIEPDDYRIEVRSQSDTELSESPSEK

LEDPHFGSCKGQEEKLKK

>tr|Q6CBZ2|Q6CBZ2_YARLI YALI0C14190p OS=Yarrowia lipolytica (strain CLIB 122 / E 150) GN=YALI0C14190g PE=4 SV=1

MRNLTGEPENPFDQQSYQSSDERSHGSGDYLYPENDSYHDVTSQTDTPNPSGTEHLMPQV

PSRATKPYFDSNHDGASSRQHLVDRSSSSGSLAGMGGSMTPNGFDRYPDRISSVNIRSAT

ASEVGSISENDDNPFVVNADFSPFGGYPASSFPLHIDEKEQDDYLHNPDPVLDQEMDKRC

QCQKMDRRGISSLIAFILLIGGLIVVFIVLPILTYSGVTERVAHPVVHREVEVLSAYQFP

QLSFRKELVDPDTPSSAMTHTADDGSKWDLVFSDEFNMEGRTFYPNDDPFWTAVDIHYAA

TKDLEWYDPDAAITENGTLVLQLDAFKSHDLYYRSGMVQSWNKVCFTQGYIEISASLPGQ

GRTPGLWPGLWTMGNLGRPGYLSTTDGVWPYGYNNCDAGITPNQSSTDGISYLPGQRLNK

CTCKGEEHPNPGTGRGAPEIDALEGSVGPKVGVASQSLQVAPFDIWYIPDYNFIEIYNAS

VTSMNTYAGGMFQQAISGITMLNNDWYDVVSVEGQDESELHYQTFGFEYLNDDDKGYIKW

FVGQDPTFNLHGYALGPNGNVGRRDISKEPMSIIMNLGLSNSWTYIDWPALNFPSKLRID

YVRIYQPSDQHSITCDPDDYPTYDYIEKHKNAYENANLTLWTDAGYTFPQNTLMHSCT

>tr|Q6CG76|Q6CG76_YARLI YALI0B00154p OS=Yarrowia lipolytica (strain CLIB 122 / E 150) GN=YALI0B00154g PE=4 SV=1

MHSSDEDEVPCVDFDDDGDYFSGQPQSPTTPKSPPKTLKTRRRKRDRSVSSSKGPTPGPP

TPEKTQTSSTSVPPIFQLLGSITGRASDLYNSWTRPAPAAQSQPPRRGSAATNSSYVTAF

NSNNSPLMRDIMMSPELQPLPSLLSVPVNAPPTPPGEDYLKPKAQTQTQAQPHTTQKASP

TPATKRLSLPNVTNIRQQALQQATHYSNTFLSSPLLLAGAAVVVGLAITVGRPLLEIYIH

KGIHYFFNFIFYATVTAAVVTCAAIAWMLISGKLKKTNVDIDPTQTKTSTGSSHETNPTT

TPFTDTTPTADLSRRSSKRRSRRASAATTSASAPPPPMMYPPYGYPPQQQWDEYNHPGHP

PMNMYMPPSPMYQPQLFHPVPMRPFEPPQSTPAAPPVTPQTSAFSLPPTPGHARTESGNV

APIPEILQQPKKKPVYRVVQRKIERDPVREARPPLRTRELPPVPREPPQVPKHQGLPAVF

TRFEDENSMVNYASPISEVDLKVGDHSFKPYGSPPRRYHGMNV

>tr|Q6C8C0|Q6C8C0_YARLI YALI0D20922p OS=Yarrowia lipolytica (strain CLIB 122 / E 150) GN=YALI0D20922g PE=4 SV=1

MTGDILDANSISEEFVDSGITKRDTKGKEKGDVEVEEVEDTSKSRRGGFVEFWKTFTSKS

FRHRHVWVFHLFWSMFFTAWWISILAQPKHRHMWLIPTILWMCIIARFITLHVPARYLIV

WASYIWDRTVIKVYERVPSHLRIPGAALGTLAVILIGTFVTKEYPDSLRSDRAVSFFGYL

VGLFCLFVSSNNRHKIRWETVIAGVLIQYIVALFVLRTKAGYDIFNFISTLARELLEFAK

AGSAFLTSPEESQLPWVVFTVLPAVIFFVAFIHIFMYWRWVQWATVKLAYLFFWAMKVSG

AEAVVAAASPFLGQGESSILIKPFIPHMTKAEIHQIMTSGFSTIAGSMLVAYIGLGINPQ

AIVSSCIMSIPASLACAKLRYPETEETVTGGKVVIPEDESVESADNVLHAFANGAWLGLI

VAGAIMTTQMCIVALVALIDALLTWFGNFWNIHELTLEMMLGYILFPVGFLLGVPRHEIY

KISKLIGTKFIKNEFVAYLALSSPEYHSLSKRGAMLVTYALCGFANLGSLGIQVGVLGRL

APSRAGDISRVAISALITGGIATLLSACMAGMVLADMTKFATPSEAVATGTVG

>tr|Q6C5J0|Q6C5J0_YARLI YALI0E17677p OS=Yarrowia lipolytica (strain CLIB 122 / E 150) GN=YALI0E17677g PE=4 SV=1

MSSTRTTTAAGQIRFRADPCAQESFIEHAQHTVTDTIEEVKESLTHLWDDIPAWQRDNCY

IRRGYVKETNCYKKTFKALGFVHNETVNIYSHLIPALTTFVGGVAAYGLGTYLDVGLPIY

PTTTWKDHGVFILFMFGIVSCLGMSATFHCIKCHSQQVARTGNQLDYLGIVSLVVSSMFG

IIFYGYDHGDYERWLYWGLTFSLGTICACVSLMKKFHTSEWRPFRALMFVLFGLSGGFPV

IHACFRFGYEGTVLRIQLPWILLEAAAYIGGAGIYAARVPEKWSPGTFDIIGSSHQIFHM

CVVLGVILHWIALLGTYHAYHGWALSQSPIFFR

>tr|Q6CBM9|Q6CBM9_YARLI YALI0C17105p OS=Yarrowia lipolytica (strain CLIB 122 / E 150) GN=YALI0C17105g PE=4 SV=1

MSHEPPGTTILENHASDAIILQPTPTADPNEPLNWSKWRKHTNLFIVCFFTLMAFTANCV

STVFWTPQNQELGWTLDQQNNGYALSVAGLGLGCPLMIPFAEKFGKRPVYLVSSAIALAC

AAWMAKMTTVGELYGSSLLQGMATSVTETIIQMTVADLYFVHQRGTCNGIYMVVVDVGNF

LIMVPAGYITMSLGWRWVYGIIAVITGAQFLSTLLFFEETKYTAGTETLVGVGEDEEDRP

EENLSNKDAKDIVRNLESPSTESVIMDFSDSASSRNAYQYQKNPLSKRLALVTYTPGSWR

EFGRKMYTPFITLFAYPIVTFVAIQYGFMLTWLAMAATTVASAFAEPPYSFSSAALGNIN

IAPFIGMALGSVYGGWFNDKTIIWLSRRNGGQYEPEMRLYGLIPANLTLTIGLFLFGLPI

AHGVHWMVPTLGFAIIMFGFGSSGAIVLTYLLDSYKNIVADAFIGVIVVRNVFGMIMVFA

QTPWIEKVGLQNVYITVGCISLVPLALTVPLIYHGKHLRRKSEARYLRESTRR

>tr|Q6CFG5|Q6CFG5_YARLI YALI0B07205p OS=Yarrowia lipolytica (strain CLIB 122 / E 150) GN=YALI0B07205g PE=4 SV=2

MFSDMQKYGAAFTAGGIFFIFLGIMTFFDSALMAFGNILFLIGITLLIGPQKTLAFFARR

QKLRGSIAFGLGIFLILIKRPFIGFAIELVGILALFGDFFAVIVAFLRSMPVIGPILRHP

AVAPVIDRLAGVRILPV

>tr|Q6CHI3|Q6CHI3_YARLI YALI0A08536p OS=Yarrowia lipolytica (strain CLIB 122 / E 150) GN=YALI0A08536g PE=4 SV=1

MSRLGLEIYAKWRTGWVYGTRSIGWLPLLSLLVSVIGALWLAVLPLDGQYRYTYVSENAL

LPGQAHTHFRESEWNHVRAYKQEIQAIVNSEAGGHINEQERISHVRGYLADIGLKTSLHE

WSVDHFSESYNGTNVYGVMHAPRGDNAEAMVLVAPWINQDGEHNVGGISVLIALARYLKR

WSVWSKNIVFVIPSDSGFALRSWVTAYHTSLALTAGAIEGAIVLDYPEASSEHFDFMEVF

YEGLNGQLPNLDLINTAVIAASTENLATVIQGVTEDDESFVKKYMPESLKEFFGNQQMDV

LNRYANRLKIMLSGMTRQLTTGISNSPGSEAFSGWRIDAITLRARGTTGPFDITTFGRMA

ESTLRSINNLLEHFHQSFFFYLLLSPTKFVSIGTYLPGAVLVCASFPLTAIYLYATKPNR

STSLVKSFFIIIAVFGACTTIGGVLTVVPTSAVGPVLTSLFVISACLPILSVVAPSPPPH

PASAPAEPNVTLAISLLLHGLVTTALATINFSLAMVLGLLTVAFNFIRPGSAHTKSNLLI

LIITCPWTWLVAVGAVLHTSPFDILQMLLWSLRGNQVWTWQVLFGLWLPQWVLAVIVSRS

STPAPAQSTVGYFRKQ

>tr|Q6BZX1|Q6BZX1_YARLI YALI0F30239p OS=Yarrowia lipolytica (strain CLIB 122 / E 150) GN=YALI0F30239g PE=4 SV=1

MGIKDWLLSTERGETVDRAREMLSEEEAMRRYEELVKAGFLDSKLAPSVSTFARVRSLLL

IFLAVGIVAGVIYVSWVVYLGFKATVKEGLQRHHIKVSRDGAQVEVKNVTQERMIEKATK

KGLRAWERSDAWMASSVAVQHANEREAARKRNA

>tr|Q6C1N8|Q6C1N8_YARLI YALI0F14685p OS=Yarrowia lipolytica (strain CLIB 122 / E 150) GN=YALI0F14685g PE=4 SV=1

MTAKSRVRVDHDEQQRTQVQSHLQDEGSATEPAQPADPPLDHPDSNKVHKWISSAYHLGS

LRLENKGAVARDHLANERTFLAWMRTSMTFATLGVGLSQLLRLSDSATNVNDPQDQLDKI

EDNKRLARIAGALFVSAGIVVLLLGTTRYFHSQHMIVNNHFPVSRMSVLFVMCLTLALVG

VIIWMVLRV

>tr|Q6C311|Q6C311_YARLI YALI0F03520p OS=Yarrowia lipolytica (strain CLIB 122 / E 150) GN=YALI0F03520g PE=4 SV=1

MEYPDLDRLRVTQRPSRLQSLTLQSILAQTKRPTAFFITISLIEVGREVLKEHLDHMTAD

LKEHPELKQHRGMATSYVALERLVWLIQKVNQSIQQKPQLASESEEELMQNMSWFSETLE

QVARMYAACTDELMDAVVVCSITIYIHAASLPNVVPILSFEQEESGETASADLVSIGKSV

TDLAESMDGVYTLPICWDREQTFFLPREEDMWKIWHFADSESKAVIGYELLRFSKTYIAV

VEDGSFDNMATWALKAPELMRLCRKRNPYALLLYCYYLAIMYFGLPLFDKLKRVEMDIVD

ICEQVPANFRPHLDWPLEICCSERDSSGEYESFMEGRLRQKFLRPGEEEGLVRGRPDEEG

VLREGVGVGLVAD

>tr|Q6C3C0|Q6C3C0_YARLI YALI0F00946p OS=Yarrowia lipolytica (strain CLIB 122 / E 150) GN=YALI0F00946g PE=4 SV=1

MYPSPIQGFVVASDFDDAPALGLKNVLEPRSLPKISTLKQTSPSALHALHTHTVETHLGT

ASDSEPSPISPISPKRSFSPPQQSHVPQVWNRRYSESSASSDRPLRRFSFDTDAYQRQRR

QSVQSQGPVLKWLRKATVFLVEIFDEADPLEPEEEFPAPSLTHQNYRLSSANNSAIESDS

DTELEKIKSRDSSNKNTSTSTSTNSTNNMEIISTGKRVYNFFSERPHWLGLLAIFTALPL

SSHVVYEWLQWAVIGSAVLLGPTYTAGFYPVLGFVSLGFMVNFPAQVCAFGLLLWLAREW

HTVEVMRKSKLTE

>tr|Q6C777|Q6C777_YARLI YALI0E03124p OS=Yarrowia lipolytica (strain CLIB 122 / E 150) GN=YALI0E03124g PE=4 SV=1

MNVIFENTPMADYLQQADNPGMADWGLDDEYDFDSSDEDLLQSYEGRRSYEKDPNVEENL

RRIDEEDEYDTDTNTENVSSPEQQPSQHPQSHPQNTQRKHRRFASTSAIQPLHSANENLM

KRERFQLLLAGALRQSLPLVENNRFLEKFRYIVVTSQLLSEQPPVSSKTPNFPDDKDLLP

ETTWATSGGCVVVVVGLFSWAVRKKAIFEGKARAMASFVATLCLVIFMYAHSRRRLLRII

RIKAVQSARQFVLSSQGFDAAVHKSLGVIHEVEFLSKGFSFPQKTVEGTDLDVRLCKQLR

AALSASLYLSISSLTRSIRKMLNHCHKLDLEKYLDIYEVDVMDLSLFVEDDGPIPEHDFT

ASSPVEALRNMVDVPEADFYGSKGANHSHKNLKFYHYKFHLLRRVMLCCLLSMTASGTDG

SEYTQWKLVSGELSRSEKLLRQLDESLVDTIYGLDHDAKLLSHSRSDSLFGKVIDSRKGR

RLSSFNLLSEPSSAASEPSKEVQQHLKQLSRLSSTLRQIEGRMQIAREGSMSHIESASAD

ELLTNYRTLGDDIQSLASIWQGGMSSLEAKLKNPSTSRAEKTLSFIEKQRHLSDATVDTL

ASPLLQQLASLGSPVHNNDYSTVSQATTLAEFEDDVHGPAAKQSALSNTYRALCGDSTPV

SSPPMSPTMLEDENGIPIQYSQFGKRRSFHQRDMSLTTILEGIANKDKGKGLGIDTSGTV

HEQREKRIARMKEEREREVDNRAAANRRGSVMLELKTVLENR

>tr|B5FVD7|B5FVD7_YARLI YALI0D03949p OS=Yarrowia lipolytica (strain CLIB 122 / E 150) GN=YALI0D03949g PE=4 SV=1

MSALFNFQSLLLVILLFICTSSYVHATFPAIMDRNKTGPLGIFWKAARIGERLSPYVSLC

CIAMAVSKLIE

>tr|Q6C334|Q6C334_YARLI YALI0F02981p OS=Yarrowia lipolytica (strain CLIB 122 / E 150) GN=YALI0F02981g PE=4 SV=1

MSSWRLILALVISTLVTCLMFAEALVVRPNFYSACISLTQSNSKKSVLLATILTHIFCAR

KLLMRIFYGELRAVEEQHTGEKLWYSGTEILFAIAVFHKELSQVHYASIFTFVWLVKSLH

WVAEDRVDLLFTTGESDWAHVRYLSTLTVLCATDVYLLNSFYPHIDFDFSKFSTGVEQGI

WVILALEIGLVLNSIALCHNKYLISMRERYFLRQNPNDETWTHKKDWLFAAEAASDLIQI

AMFCIFFVLISSSHGMPIFKFRDAVVSVLNLVSRVKGYYNYRVLTRQVDSFTTTPSEDDL

ARNQTCIICFEDMELVEEPKQLVPKKLSCGHVLHNGCLKHWLERSKLCPTCRRNVFTAPE

VVATTTVAQVTQVTVPVAPQVADHPAWVEQAVQSQSSSTSESSSNDAHAHHKLKLRQSAI

EAGVHDSWEVFPTRRNNKNQVELCLGEGRWAVLETVY

>tr|Q6CH92|Q6CH92_YARLI YALI0A11121p OS=Yarrowia lipolytica (strain CLIB 122 / E 150) GN=YALI0A11121g PE=4 SV=1

MSRLRAATKRLLDRPDDVDDGVTAPLDEQEQLELLKSMEMDMANSNEQFRFAFAALACLE

TPIFIVLPYCHNHGNKPLAYMSLTVLLVCAYITYKVKVDDKAPKKYLLPMASVLAVLILV

QAYLKHAPWVGYDYLWVIPAFTCFTTMVIRHWISESSSDLHKLKGMTYKLKGA

>tr|Q6CEE3|Q6CEE3_YARLI YALI0B16324p OS=Yarrowia lipolytica (strain CLIB 122 / E 150) GN=YALI0B16324g PE=4 SV=1

MSNKPRDLPEPPSPAFVSQRQSLHIDANPFSNPGSPTRSPERQGAYGSPYGTPRAESPTR

FGSPTRYGSAHGARGTPRAEWTRRETPVSEFDPIVIDDDDDDAVLAPPPVFPTVDSLRQS

QMSVTSDLRQSVMSTSSGGHSSHGYGHQYALARSITSQSTSSSITATSSRDSMIDHDEDD

DDEMEFRKGDEKNGYRMKYLDDRVHDEEEYDEEDDSDAHSVATVASHLYEAPLQAQPRRH

KSMARKKVKLVRGNLVLDCPVPTKLYSFLPRRDNDEFVYMRYTACTATPDEFNDEGFTLR

PAVYERETQLCICITMYNEDEVAFTRTMHAVMKNIAHLCSRNKSRVWGKEGWKKVVVTIV

SDGRTKIHPRVLDCLAAMGVYQDGIAKQTVNGKEVQAHLYEYTTQVSLDSDLKFQGAERG

IMPVQLLFCLKEKNAKKINSHRWLFNAFCPQLQPNVCILLDVGTKPGKDSLYHLWKAFDA

DSNIGGACGEITAMKGKQWRALLNPLVASQNFEYKMSNILDKPLESVFGYISVLPGALSA

YRYRALCNNPDGTGPLNSYFKGESLHGRDADVFTSNMYLAEDRILCWELVAKLNEKWMLK

YVPSATGETDVPDSVPEFVSQRRRWLNGALFAAIYSLTHFTQIWKTDHSVWRKCLLHVEF

AYQFLQLLFTFFSLGNFFLAFYYVAGSISGPDSAVISNNGGLVLFVIFKYLCITTIVAQF

VISLGNRPQGSHYLFLGSMVIFALITAYSTGVGLYFVIKTLTLEDTVSLGNNVFTNIFVS

LASTYGLYAVMSFLYLDPWHILTCSVQYILLLPSSICTLQVYSFCNTHDVTWGTKGDNEL

TPDLGSAVAQKDFGQDVVEIEMPSEQLDIDSGYEEALGNLRENKVIESAPPSMSTVTDDY

YRDIRSRVVLVWMALNMVLVVVVTEVYAPSETASNDYLKFILWSVAALALFRAIGSLAFL

FIRILKYLNEAKARTLKRHQGLTGQQ

>tr|Q6C3A4|Q6C3A4_YARLI YALI0F01386p OS=Yarrowia lipolytica (strain CLIB 122 / E 150) GN=YALI0F01386g PE=4 SV=1

MRASLAFVLTLLLATSVSAYKSVRGGIPAPVGLACWYAAREYDNTCSVGNLTACRCGSPI

FMGSVTNCIDRWTSIAPRYPGLHFQENNPFSLITGSRREEFEVAYGYSVEALKAYKFIQD

LCYQLGPRFQYSIGHLEAMAVEAKEHVIGAAEAEAQLHFLGEVRAPVAVRETRFIQYLYS

MDVVIRQYSLGIIQGVVLNIYWALVIFVGFATNVFYSYFPDVVRRMKGPRITWFRRHFTL

PATIGTEHNAPHVILKYIQIHLPTRIHSIVIAIYVLLTVVLTIFPIHFSANDPYLGTSLI

KFARYTGNRTGLVALTQIPLLVLFGGRNNILLRYTKWPFNTFLTYHRAIAWVTFSHMVFH

SIAFAFLAVYKSTYPHNWGFINWNMGHLATVAAAVMVTVAIRPIRSRVYELFYQTHISGF

VIFLVGLGLHCHNFGWMGWVYTSGTIFLYNELSSIAKIFFCGWNSIATLTLFEEGMFKIV

VTTSGRWNFFPGCYCYIIARPNLLQGHPFSIYVAPDNTSTPGNAQLTMVVKPRKGKTARL

TRMLQDHYESTGSRSMQTRLYVDGPYGNQQPLSEYANILLVSGGVGVTAAYGYASYITGL

QQSPLRSTRALQIIFVWIVHDARSLKWFQTELLHLIKSPNVKVEIYITRGFLDKDDSFVP

STRVYQAFQLERWVLCGSPTAPMISRSVPIETIEAVKHAPIKITNPTENTNYFDTTDTPP

RPLHSAGSTLTSHASPMLSAFPPPPDNDTSMTGQSLDPGGTARTLDPNVLSAKLAQASAS

ISQERKDKEAAGDTPHDSDSDEKPHDLVVTPPTPELNHPAQDPEGNRESYHVPEKGKFKI

EMSKPEVAKLLSRHFSDITNDDSVGVFVCGPEKFNDEVRAAFVTETEIHGNIRMDYFEES

FC

>tr|Q6C8F0|Q6C8F0_YARLI YALI0D20196p OS=Yarrowia lipolytica (strain CLIB 122 / E 150) GN=YALI0D20196g PE=4 SV=1

MFNLNTKSSKEPEVTEVAVDSTPSSPVTRESSPESSPDNSAVDLEKGKKSKFEMHDQTNL

LPRSQLFLVFGAMAFCMLVSFMDQNGISVALPDIAKDLNATDTISWAGTSGLIANTVFQV

LYGRLSDIFGRKLVFMIVVCTLVCADIGCACAQTSTQLYIFRAFSGIANGGMSCLTMIVV

SDIVTLKERGKYQGILGACVGLGNTIGPFLGAAFTENVSWRAIFYLLAPMGGLCAVVIFF

ILPSKKPQGSAIQKAKAIDYPGLFCSSVALVFLLVPIAGGGSYYEWDSPMVISMLCIGGV

FFIAFFVIEGFFAKLPMMPLRIFGTKALFALMMHSVLLGIAYYGDLYYLPMYMRNIRGWS

SMKAAGMSCALVTTQAVTTVISGQYLSRMGRYLEVIYFGFGIWTVGAIMKCFWKRDSNMA

LLIFSLLFEGAGVGCCFQPTLVAAQALSRKEDRSVVISSRNFLRSFGGAVGLAVCSAILA

NSLKADLKTKNLPSELYELIVKAPFSLPDLSHYPEYRDQVLDAYMNGSHTVFVFLCPIVG

ACLLVTVFVKDHGLQTHEKKAAEAKTVEDKDKDESGTDCEDMTKGEVLVSEKEGKLSRNS

SSQSMHFGDHTAVNTPAPTGYNTPVVGTLCHNSPNFPPMDHNDVITPLEDFDESPLPPHS

PNSSGKRVTIQEE

>tr|Q6C9X5|Q6C9X5_YARLI YALI0D07568p OS=Yarrowia lipolytica (strain CLIB 122 / E 150) GN=YALI0D07568g PE=4 SV=1

MSAPPVAKEDTNPVQELARTPYPAWIMSGLLLATLPYAKNTVKPTKLSCAGFGLVFAAGG

YMMLDDITNGAGFTSAWSMLYLLANGTRSVTVFRKPWPLLLSCLAVSNAGLYGATFFFPK

KNEVPPKKF

>tr|Q6CGW8|Q6CGW8_YARLI YALI0A15422p OS=Yarrowia lipolytica (strain CLIB 122 / E 150) GN=YALI0A15422g PE=4 SV=1

MSAFPEPSSFEIEFAKQMNRPRTVQFKQLVAVLYIFGGTSALIYIISKTILNPLFEELTF

ARSEYAIHARRLMEQLNAKLSSMASYIPPVRALQGQRFVDAQTQTEDEEGEDIPNPSLGK

SSHVSFGESPMQLKLAEKEKQQKLIDDSVDNLERLADSLKHAGEVSDLSALSGFKYQVEE

LTNYSDQLAMSGYSMMKSGLPGHETAMSETKKEIRSLKGSVLSVR

>tr|Q6CG07|Q6CG07_YARLI YALI0B01914p OS=Yarrowia lipolytica (strain CLIB 122 / E 150) GN=YALI0B01914g PE=4 SV=1

MMKLGQLSKFLPTERRVEDNVDNLETERERDEGDFPLEYRDETNRPWWKFFDEYEYRMNK

YERASHKWFKWFDERDTRSDKKLICKLDILLTVYSLMVYWVKYLDQTNVNNAYVSGMKED

LGFKGNDLVHVQSMYLAGAVIFQLPFMFLLPRVPLNIVIPAVDCMWGLMTLLTCKVHSVG

ALKALRFLVGAFESGFIPTIYYLMGSWYKPSEVARRAGFFYMGQFLGLLSSGLLASAALN

LDGVGGFEGWRWVFIVDSICTLPLAVVGYFIIPGTPDKCTSLFLTDEEIIRCRERKKPFV

TEKKGQQLKKLTDWSLWKPLLTSWHLPTAILISCFFYNNTSGNAGTFILWLKSLTDSNGN

QRYTDQKVNQLSTIAPGLGFAYVYLASLFGDLARCRWGGIVITQMFNSIGAVILAVWDVP

EGAKWFAFCLQYISWAMSPIMYTWWADIMRRDPQHYAIVLVMMNMLGQSSSAWVSVLVFP

TSEAPRFRKGYGTCAACACCCAIMAVVILYFYKRDEKRDCLRNGILLVNDNEDNGDKTGK

GVVIEGLSESFDEDTGVAKKLEVEKIREASVSDENVSNLSLVVI

>tr|F2Z6G8|F2Z6G8_YARLI YALI0A09471p OS=Yarrowia lipolytica (strain CLIB 122 / E 150) GN=YALI0A09471g PE=4 SV=1

MNVVTLLLYVSTVSAVVVTVTLIETAQAVDSSRAATTKSMTSTSSSSRAQILATGTPPMF

ASAKNAIISNVRSLKVPSASGYLAKDVEPTQSVTPFTLSSSVSPATTASANPTSAVSVAQ

TNSVSRTGVSVMFLSLSFVLVTCSIA

>tr|Q6C464|Q6C464_YARLI YALI0E29359p OS=Yarrowia lipolytica (strain CLIB 122 / E 150) GN=YALI0E29359g PE=4 SV=1

MSPITQIAVFFPGVATSAYSISGSAYVSLMYSMMRGIEKPRQPHDKVATAKTRPMVVKKQ

HVASSKHRTASARNLTKMRGSSTHLSGLQKEGSGDSLTKRKARSSDSLQRLARPNLSMTR

TNSGNSNKQDTSASSDKLNHNETSPKPASSKEANDKENEEQQKSKEQDKPVHLRQGGKVV

IGLPVSDDEGSEEEDEEEVDEDNGDNGVAANGLTRDISRLAIGTEINEQEQADANQQNGV

YANDIPGEVADTESKTMAEGEEAVVAMQEKMAGEPEVRRTSNNIHKEKHVLIDENGGHVE

HHEHAQPGSADSPVSPKSAPGITNKRLGKSVEKDGPRDPESSQENICEVYDESEFPDSPE

LKNVSDSCNPSRANSRNPSRNPSRNPSFEGLGLLRSLGPLSNNKMTPASRNSSYDSLRLS

QQATEKAPTLELSSHHVLSNPDGSSGHTLGSNTVVTSESPQKTPNSTPRKEQRSRTQQKL

WLQRENAIDEHGSPRLPATEMPDQLANGKAEYQVIDKQYKSVRTFYNPILEGLQRHEICR

WYENGKVVNPKRREVQQRQLQKDLQAPTGDVNEILATLFKGVKSESQGLANQAQRATLAM

KNGE

>tr|Q6C7Z3|Q6C7Z3_YARLI YALI0D24145p OS=Yarrowia lipolytica (strain CLIB 122 / E 150) GN=YALI0D24145g PE=4 SV=1

MSTHSDTDVSHEKRSFKKPTMPNLDSEWLTQVLLYLNLSVFAMAGAIARQGITLLTVFTG

SYSPSGLLWCNFGGCIIMGWLEESDIFAVIEEQKGVEKRKIPLYVGIGPGFCGSITSFSS

IMLEAFLFGANQNIPKADYPDAGYGVQSVMAIGLIHYSLSFAGLLIGHHIADFLPIPCMS

VPFERAVSFVIGCCSIALFCLFIIFAAVWKSWRTWMYLGLWALAGANFRFQFSRMNRLGS

GKNPWGTFTSNMIGTIILSVGVILQMGFVGDSQLVTNVVQCQIIGGIANGFCASLSTVSD

LVNECYNMSYKRAYVYGWFTNFFGFSLMIVILGSFTWTNSLTAPHCA

>tr|Q6CG86|Q6CG86_YARLI YALI0A21307p OS=Yarrowia lipolytica (strain CLIB 122 / E 150) GN=YALI0A21307g PE=4 SV=1

MDAQNKTAGGNAAFHNFVNDFAHIEDPNERRRLALEKIDSASFGWYHIRAIMVAGIGFMT

DSYDIFAINLGITMMSYVYWGQGTGHDGHIPDSTNTLLKVSTSVGTVIGQLGFGILADVV

GRKKIYGVELILMIGATIAQCMTGHSQAISFVAVLTFWRIVMGIGIGGDYPLSSIITSEF

ATTKWRGAMMGAVFANQGWGQLMGSIVAICCVAGFKNSLEPYHGANDCGFDCQQALDKSW

RILVGWGCVPAMCALYFRLTIPETPRYTFDVSRDIEKAQADIDKYTSGEHGNASPEEIEA

LKAQAEELQTQQNAYNPPVASAGDFFRHFSQWKHGKILLGTAGSWFLLDVAFYGLGLNNS

TILHTIGFDKGDNLYSTLMKNSVGNLILICAGAIPGYWLSVVTVDFIGRKPIQIGGFTIL

TALFCIIGFGYDKIGEGGLLACYILCQLFENFGPNVTTFIVPGECYPTRYRSSAHGISAA

SGKVGAIIAQVVIGTLVNHNCDRDGKPKGCWLNHVMEIFALFMLLGIGTSFLIPETKRRT

LEDLAEELHGEVQWRPPVEDQAEDVSDFNDNEKSV

>tr|Q6C034|Q6C034_YARLI YALI0F28127p OS=Yarrowia lipolytica (strain CLIB 122 / E 150) GN=YALI0F28127g PE=4 SV=1

MVSTRRTRVLILTAILFTVGLLIFAVHGPRYDLAVDPRSSSQKTVKTTDNNIDKEVLEVN

EDEGVAHKSEKEKTEKIVKGEEKADLTTSQGEEFNPEKEYQSILKLAPVIVFSKSYCPHS

QFVKDLLQKEYAITPDLQIVELDKHPSGPELQAYVKQKTGRGTVPNVIVAGKSLGGGDEM

RALHKDGELAETFHEAGPKALTVVRRSPL

>tr|Q6C282|Q6C282_YARLI YALI0F10010p OS=Yarrowia lipolytica (strain CLIB 122 / E 150) GN=YALI0F10010g PE=4 SV=1

MFTSRVSEASTTNFIRPTARSHIHFFFAFIAATVHQLLLMLYQLLGDGYLKSFVDTGITL

AQQSGLSGIVNALTSEAKLRIDKRSIIKKLLEDQENAESYFDWLKASSELDYLLGNQEWK

ERDECPAYDYEYVRLRLDELRHARTNNDTTRLLYLVRTTWSRNLGNLGDVKLYHNSFTGT

KRLIEDYILECELALNALLAAGNDKIPDQELLTELLNTRKAFGRTALLLSGGGCLGLLHT

GVLQALSDTSLLPHVISGSSAGSIMAAGLCIHKDEEHEAFITELMERDFDIFEESGNEDT

VLERVSRMLKHGSLLDNRYMQDTMRELFGDMTFLEAYNRTRRILNVTVSSAGIYEMPRLL

NYLTAPNVLIWSAVCASCSVPLIFNAYTLLEKEPKTGAIQTWNASSLRFIDGSVYADVPI

ARLSEMFNVNHFIVSQVNPHVAPFLKLTEDKANPDSVDEIYTLKLWHNFKTLVTDEVMHQ

LQVLYEFGIFKNLCSKMGGVLSQRYKGDITILPQVHLSELPGILTNPTAAYMKDTNRRGA

QATYRKISLIRNHCAIELALDRAIHELKARMLPSKLGSGRTSPQGTFKHSQSSNQISALK

PPSRHMSASSATTAHTRLRNRKSFSHARIKSDAAAVFDKEPIHETPKSSPQSSYVNLHRS

ASERSRRPKSAFNLGSLPTSPLYHPHLTHSMSMGGANQAPLYNPGRGSVSQNTSPGTKIP

GNADPSYFDGPNNVRFHWDSDDDDVRETEFLNNMSSSSSRRVSPVQSRRASVDGLRNSVV

STATSVTDGSVSSRPSRAWESISQLFEGDENCSDSC

>tr|Q6C2Y5|Q6C2Y5_YARLI YALI0F04081p OS=Yarrowia lipolytica (strain CLIB 122 / E 150) GN=YALI0F04081g PE=4 SV=1

MTLDDSRTTVKRLAKHLLTQFEALTKGYTGMLLALMSSFFSSGMAVTARLLQNGNNPDDD

FNAFQILFLRMSLTIGVVLWGQHKVYARKVKQQEENLPPRLQFITGIPEVRHLLVIRALC

GFFGVFGLYYSLNYLELSDATVLTFLTPVATSLLAWMFLGEKFTRSMALGGLVAFCGVIL

IARPVFLFQLITGSRDTSGVRPIDRLRSIGFSMLGVLGGGSAFVAIRSIGDRAHPTVNVQ

YFSTWCWLISLIALVYTGKGIRLPHTWSQAAFIVILALCGFMTQLCMTGALTREKAAKVA

NITYTQIVWALLWDKVLWNNWPDVWSVLGGGLIIGSAIWVAMKKDVTVATVNDIPEVVPT

TPQVDEYHVDTSYYSPDEEAAVGMPLTPISKREAKVTIKSTGNDDDHLRLDAEDEEKDED

DDDDGEEVGLMHKLK

>tr|Q6C9C8|Q6C9C8_YARLI YALI0D12232p OS=Yarrowia lipolytica (strain CLIB 122 / E 150) GN=YALI0D12232g PE=4 SV=1

MIISLTVTVKTTLSCVFVLLPIPVAVLAFVAAAPLTSPWRAALYLQGAASVAQAASYAAF

LVHKKAIPACVFSAFLAWLAGMVFYAKVVRDVAFDDNSGSVLVTHHAGVTTAHFALWSIV

AALAGAYTLFVLISEHSSIIAAIEGPNSTKPSPSTISVDQFKVDPASTGMDLNMNTNSTV

ARTNSGGYAGHAPHHSYTTSNHMTNNNINNYQAGLTQEHHATPLLPSMEPEHEEGIDNPT

VVVNSLAGLEAIPASAGSTAEPTSRHTQSTSSFGSINFAGLNIRPPPSSKFIHPSLQIRH

NGRRMDRLRHSIDELSDGHVRCDSAGKFDSSELAGPSRESGEINTSHHRYSTELHTGTQS

VFSTATTATELTVKSPAEPLHPHTNIAPSSTPPNPSSPTKSTHGSIYNSAHNSLSSSPAK

SCSPRKPSRLSRLSRLSLSLEGLNDISPKKLRKSRSDFILPGSRDKNDTPGNVTTPQPDE

FNQWEIQDVAWNLRNVSGSSADTDGTRQDGQRSASTASSGVNIISSRGFVREEVV

>tr|Q6CDT7|Q6CDT7_YARLI YALI0B21296p OS=Yarrowia lipolytica (strain CLIB 122 / E 150) GN=YALI0B21296g PE=4 SV=1

MPKVEIKEVEGDTIELDGMKIPLRMPTRGPSGPTLMDQIDSLKQKQTEKEAKAQGRPIPV

YKPQPPRTTKEGKPLRYRKVAVRTQDDVYDDDAEDEDEEDTGIPTDSKWFEFLDFCMYLI

PLMSVHLVLDVLVQKQYGGGDLDPALYGQPFPKRGDLDSHSIQIRRQELSTIIQAAKRSL

TSIPVLAGLHAFCAPYVNAISKLQRGQVEDDRQLPRGIRVFRVVTFAASIAIGCYILYAA

NEQGYMAVMKRAPPLGTLWVWLIVEMEWNWGALSLVVVCMWSYMRGYLS

>tr|Q6CHA7|Q6CHA7_YARLI YALI0A10703p OS=Yarrowia lipolytica (strain CLIB 122 / E 150) GN=YALI0A10703g PE=4 SV=1

MAAPDSPSTPDRSFVNIPVLPPPKRSPHKSPKIGPQSPTLPLPPVQSPIPTTPKSALRFP

QLEPRSRSNSNTSNRSRSPSPLRKVVFSPKEELIDGGKKSKDNLPNLADLKELNQRDLKE

LKEQQAKDFGSFLSSPKTSTSPFHSSTPPPPPLHLDTETRSPKGRSKSVGAASSPVDITP

TQTPIMHTPTSTLPSYINPVSTAFCLGLAVGLSISAIRPTLEKAVNHGSSWLAWLFKTVI

LWTVIGAAAYMAFTLGKAVLITIQQNQQKQQQTAHKLPPTPRSDPFEGLKPNKPSSSSGS

TVSASSSKSASSTKSSGSAASIYEFNKYVDEQKVKRRGQKDEEHSFRPYPARPQLSSTAP

GKIYGNMS

>tr|Q6BZT1|Q6BZT1_YARLI YALI0F31119p OS=Yarrowia lipolytica (strain CLIB 122 / E 150) GN=YALI0F31119g PE=4 SV=1

MPESNQSGEAIFRSAEMSLVQLYIASEIGRETVMSLGELGLVQFRDLNKKVNVFQRNFIQ

EVRRLDNVDRQLRLFERECEKEGLTLEDGDPHSAASASDIDALVALGDTLEKRITELRDA

EERVTESQTESQELRAVLTETAKFFDQRAGGGSRDIESQSLRNVQFVAGVIPRDRVEVLE

RILWRVLRGNLFLETAEFGGDDGDKSVFIVFSHGAEIISKVERIAKTLDAHLYWIADDVR

ERENQLQEVNQKLSDIDIVSQRTRHTLNTELRLIAQKLPNWRVIVIKEKSVYSTLNLFQY

DTNRKVLIGEGWVPKDDISKVKTTLKSITDEADVEIPSVLNVLETSRTPPTYHRTNKFTS

AFQLIVDAYGISSYREVNPGLPTIVTFPFMFAIMFGDIGHGFILFLAAFALVYYEAKIGK

MKRDEIFDMAYQGRYILLLMGAFSMYTGFMYNDIFSKSMTLFKPGWAWPESWKEGQTIQA

HQTGVYAFGLDPTWHGTDNNLLFTNSYKMKLSVLMGHVHMTYSFFLSLVNYIFFGSVVDF

WGNFVPGLLFMQGIFGYLALTIVYKWTVDWVAIGQQPPSLLDTLINMFLAPGKVPVPLYP

GQAYVQVILVVIALICVPWLLLVKPLWLRRDMQKHEYERVSGNGGPLDLLDAPDQLEETV

GDTPGDATGGDDFDDEEEEEHGFGDIVIHQVIHTIEFCLNCVSHTASYLRLWALSLAHAQ

LSTVLWDMTLQAAFGFSGVVGVIMTVILFGMWFVLTVVILVCMEGTSAMLHSLRLHWVES

MSKFFEGEGTPYEPFSFKDELATN

>tr|Q6C2N5|Q6C2N5_YARLI YALI0F06380p OS=Yarrowia lipolytica (strain CLIB 122 / E 150) GN=YALI0F06380g PE=4 SV=1

MLPRELVELILDELDLESMCVLYDTNRFWRVNLTETDFQHKLQESCPWFEPQFSHRKTWR

ECSVEYVRRMRPKSRFTPKLRLLSDEHMFKGDFYNPQDDLHSPHDLVHIQCNHLRLLDDA

YYTSEHGIEVDLSEMAAEYSDYVHDPEFDPQMECQVFSHPHMVIIIYMALSDRHFDHCGV

VVKFKDGDCRKPDIKQHITVQGSPSVYTLGAHTFLFYTHSHCFYSSFTQPAAMYLFKDRY

FVPIDIGLNDLRHAVVYDGLFAFFGKKKYYCLQANLNSSPARHSSWIKHLFRSTGSHRDP

WWGTRYVMVMGSAKKYVFDVRKGILQKVHEQTFEAPKRKFTWRDAIVIGTIFGSAVINWT

VIYFAYRWFL

>tr|Q6C3X8|Q6C3X8_YARLI YALI0E31405p OS=Yarrowia lipolytica (strain CLIB 122 / E 150) GN=YALI0E31405g PE=4 SV=1

MRTASLTTAKLLLTLIARLPLLHALPYGGLSRSGSYSPYEHDDDTLDPESSDFWLYMFVS

FVLVVAGGVFAGLTIALMGQDEVYLHVISQSGESHERKAAEKVLRLLKRGKHWVLVTLLL

ANVITNETLPIVLDRCLGGGWRAVVIATVAIVIFGEVIPQSISVRYGLSVGAYFAPFVLG

LMYILYPLAYPTALLLDHLLGEDHGTVYKKAGLKTLVTLHQTMGVERLNEDEVTIISAVL

DLKEKPVGTIMTPLDDVYTMSSDTVLDEKVVDQILQAGFSRIPIHAPGEPTNFIGMLLVR

ILISYDPEDALPVSSFPLATLPETRPDTSCLNILNYFQEGKSHMVIVSESPGDAYGALGV

LTLEDVIEELIGEEIIDESDVYIDVHRAIRRTNPGPLSKRNLVSYVQNSPRNSIGNYESV

SHFLSRSLSRDEAGKVQHQSHSNLGPHGSSQLAANPTITVSSSPKTGLKLGNNTATPHDA

SAPGPFQKKHHRIEPLNLAANPKETSNTKVTIKKSGSGHRPTGSSDLVHHPDHPEGGTST

QYGTIFETEQGAVVDALNDPEINTRLSPRTGMSPRTAAVNGVNGARPSAIPSRPDLGTEI

HSFRSGGIIESVVNVQGVHKTIIEAAVSDNDFEEEDSPKSSNGSSRRGSRRALLDSSP

>tr|Q6C498|Q6C498_YARLI YALI0E28578p OS=Yarrowia lipolytica (strain CLIB 122 / E 150) GN=YALI0E28578g PE=4 SV=1

MATLARTAKIFGLSLVAGATAMTGYHFGALGRHELTYPVATEKDHFHSKESTFLHNSQFA

KELREKGFRETRLSEKIPLEYSRGFLESFLKKPGYLTVDPLLFYKRCDDGKNGVMYAFLH

VGDHLNGHTGIVHGGFLGTLLDEFVCLGAFPSLPSQQYGVTGTLEINYRQPVRENQYLMV

RVETKDIQGRKVIADGSIEKFEGGDLWEKKKQNVLAEAELVVIEPKWIKEMTQYFKKKDE

>tr|Q6C8U0|Q6C8U0_YARLI YALI0D17028p OS=Yarrowia lipolytica (strain CLIB 122 / E 150) GN=YALI0D17028g PE=4 SV=1

MPPRKRDKKSASPSPQPPSPKGKKRLKKKLPSSSDSPTRTSPLWDSPLGFFLSPFRPTTT

QWGARYMYVSFALIIRAAVALGPYSGFQQPPMHGDFEAQRHWMEITTALPISKWYFYDLQ

WWGLDYPPLTAYHSWLCGVIGKYVNPEWFELDASRGCDAYGLKTFMRLTVLLSELLIYIP

PVISFAKWTGKQYGYFPTDLSISAAAIIFQPALILVDHGHFQYNSVMLGLALLAFVNLNH

QKYVVASFFFVASLCFKQMALYYSPVIFAFLLGLCVFPKINLRRFISIGVTVIVSFGVFF

LPLILGGGMDQVKQCLIRVFPFGRGLWEDKVANFWCAGNTFFKFKLRYTSEQLQMYSLTL

TLAAILPVMLIVFFNPKRKLIPWAFAACSWAFYLFSFQVHEKSVLVPLLPSTLLLATLDG

NVISLVTWINNVAVFSMWPLLRRENLQLQYFVVLFLWNWLLGNFEPSRLWTATLPKSIFW

RLVVIGFYVGMVALQVAEYLYPKVDRFPDLWVVGNVLLCAPCFALFWLYTLWNLWDKRGE

RA

>tr|Q6CB32|Q6CB32_YARLI YALI0C22297p OS=Yarrowia lipolytica (strain CLIB 122 / E 150) GN=YALI0C22297g PE=4 SV=1

MLIIIILFFPISPISPLFHILYFIYFIFSFSYFFFFFFFFFYIFIFIFIFIFIFIFIFIF

IFIFIFIFIFIFIFIFIFIFIFIFIFYIFYILYFIFYILYFIFYILYFIFYILYFIFYIL

YFIFYTLYFILYIFISYSTLSAPCSISRFVFKLGRKTLFSLFSLFFPFFLTLIIPQSCTK

SALWVYSKGYSWHYHGLTTKDRTCTKYKTGGQLQKSRETRHPATTPPTAQSPPLAWVRSS

HSRKIVYFVPTHVVPCTAEVSLEGCLRVRCGVRGGQEGSER

>tr|Q6CCZ4|Q6CCZ4_YARLI YALI0C05302p OS=Yarrowia lipolytica (strain CLIB 122 / E 150) GN=YALI0C05302g PE=4 SV=1

MSNLDPLIETCLSLISRLQRFGTHSLASRDVRQELADLIQTKLRDLDTEFARYKATCPQD

TTSQIALESYTEQIANLRVLYRKAQVTSAKNVEKSLLKERQMLFEGRKEVKEEASEKAVL

NKSRDITQSLKRVHQLASQNVLRGEMHLESLDESSRDMQMLQQKYTTFDVLLAGSKKLVR

HLEQADKQDRILMMASMGFLALVVAWILYRRVLKLPLMIITWPLLKLFGIVRGSGPKIPK

NPGNPVQGLVDTLVDTVVEATPSVGMETLVDTLVETLETALSSTIAADIDLELPVVSEPL

DDTLPDSGVPEQKEVPIRDEL

>tr|Q6CE82|Q6CE82_YARLI YALI0B17776p OS=Yarrowia lipolytica (strain CLIB 122 / E 150) GN=YALI0B17776g PE=4 SV=1

MIDHDKIPGTINLVDTTDSEVVLHPVPSAHPDDPLNWSYRRKLLSMFCMFIFTLGVSIPS

ASIYSVLIPISENTGLSLDTLNQGTGYMYLTFGLGCIIFQPLSQQFGKRPIFLISMLGTC

LIQLWGPHAKNSGQWIGSKLLQGFFGAPVESLCEVLVSDVWFEHERGRWIGLYGFGLMFA

SNIAPVLAGLISQGQDWTWVLYWGTIFNAVCFVFLFLFFEETNFVRHPALKKVDSITSDD

GAVTLNEKNTAKSVSVTTDVELATTDVMKKKTFIQKLALFDQPRPIMIWEMMKRPFIIFF

RFPVIVFSGFLCGLGLVWFNLVNGTVSAVLSADPYNFTPDKVGLCYISSCVIVFISSIYG

GYASDKLRIVLAKRNGGISEPEHRLWVLIAYLILCPSMLILWGMGAADKIHWFPIILGMG

FVQGMSALTSICSVNYAVDSYREIASDSMVTVILIRNCMSFGIGYGVTPWFQNEGLKRCF

GEAAAVSFVCISTFFIFIKWGKSMRKNGKHVYWNFVQQSIDNGMSI

>tr|Q6CFX2|Q6CFX2_YARLI YALI0B02838p OS=Yarrowia lipolytica (strain CLIB 122 / E 150) GN=YALI0B02838g PE=4 SV=1

MDHSNGGNASDDSPRSSTSSLFEKLDRINTAKQYEDSAASSLLRRDPASERDLESGAHIN

PRVSQKSKKFKKWFMLAGMTAITVWGLFVLALFVQSLTQPEASKDVQTVVQGSKPKTIIP

GDGGDNANYAGSNFKLVVTPQSVRTGTFRPESKSVQWIQAADLEGAYLVRDRKYVVKTYG

EDDKEIEITDTSVHYGEHNLRIDELWLRPDLKKALFVTERKKNYRHSSFGNYWILDVESK

EVKPLLKGDDKARIAVASWAPNGKAIALVYENNVYVTHVDKYETVQVTDDGSEQIFNGRP

DWVYEEEVLSGDNALWWSPDGQHLAFLRSDDTKVQEFTIPYYVQDPKPQAYPEYRSIKYP

KPGSPNPDVQMAFYNVEEDATTFINEPLAQDDIISDVTWSQTGVALIRASDREADVLKIV

HVDSSKGYAAKILRENNVKELDGGWVETVRSSKIIPQNESLGLLEEGYIDTVISEGRNHL

AYFVFGEPKPRTMLTSGDWEVVNAPSSYDASTGTVYFLSTQKDPTERHLYSVKIDGSDLK

PVTNTTEDAYYGVSFSEDARFALLSYNGPEVPYQKIIDLHSASPLDAPKVVKNEFLQTTL

DSYALPTSHFAQINVGTEEKPILANSVEIRPPNFDEKKVYPVLFFVYGGPGSQMVTKTFN

IDFQKSVAASLDYVVVTVDGRGTGYMGREFLSVVRDDIGRREAADQIAAAKLWAQKDYVD

ANHMAIWGWSYGGYCTLKTLEADAGETFRFGMAVAPVTDWRFYDSVYTERYMHTPQHNKK

GYEQTAVHNMTALGANERFLVMHGSGDDNVHMQNTLSLIDNLDLAGIENYDMHIYPDSDH

SIYFHNAQAMVYDRLYTWIQRAFNDEFS

>tr|Q6C3K4|Q6C3K4_YARLI YALI0E34089p OS=Yarrowia lipolytica (strain CLIB 122 / E 150) GN=YALI0E34089g PE=4 SV=1

MQSCDQKLTNESYHLFLFLFLSLSVRHVIILLSQVTASRLVAIELGCACISVQYIDSLVV

NNLDWFCHNLHQEDQPRSHRSNTPTMPITFDKEKFKSGVSSVRSKSGSAKGKMDGFRAKW

NGNSSSSAGSYEPPAEITSRPLTSLPDPKTFAPPPKHREWYGDEVSQQSLAVAKDAQYVQ

QPARPQPAAPAAAQPPAPAAYQPAAPAAAQPGAYQAAPAPPPRAVPAPPGAAAGGPPPPP

RPVPTPPAAGYQQPAAYQPAAAPAAAPPRDQPPSYDNLAPPQTPSRPPPMPARPGAPTQP

YGNTPPASDPNVYGAPAAPVAATPAAAASTPAGINFAAQIAGRRSSAASSVSDITSGMAQ

TAIGKKKPPPPLPKKKPGLAGKPVPGAKPTAFGAPAAAAVASVPASQAASAVEAALGAQS

YSKAPAFVPPPSGKPVDTQMHTQWFVNDQPPPDLQGLGWTSQFSSSGGTTTKIYAFRIPE

DLSIIKLRIVYPTNNPAAATCERKDVPPPRELTPGELYEYNLQYGDGVARWAESMVGTQV

GDGECWTLAKNAIEQSSQTALVSQAYTHGALIYQQNGSTNNPANIVKNVEAIRRGDILQF

YEVKFESRSAGGFSSASYGVPNHTAVVVGVSEGQRTVYILHQNTGGSKIVQQGQLELADM

TSGELKAYRVVGAEWAGQLDCSM

>tr|Q6C774|Q6C774_YARLI YALI0E03190p OS=Yarrowia lipolytica (strain CLIB 122 / E 150) GN=YALI0E03190g PE=4 SV=1

MILIKNKVALKLLQLANDRKYSMTVCGVLLALEAVFLVAIIHKVPYTEIDWKAYMEQAAL

ILGGEYDYSKIVGGTGPMVYPGGHARIFTALYDVTSGGENIRLAQYIYAAVYLGTQFFAF

TVYLLADAPPYLFPLLCLSKRLHSIYVLRLFNDVFGTFAMTFCVFTLQNQMWLLSGLWMS

LAIGIKMNFMLYLPAFLLITLQGMSRNGLLVVFALSIVGIQVFLALPFCNPSPDHIWSYL

AGAFDLSRAFMWKWTVNWRFVGQKVFENRKFHGILLWTHLTALIGFACTIWNAPSGYQNP

FEMFFTNPPPAPRGLTAAQRAEWDRIRQEASGNEVQFDVQQKRSKREQKRMQEQEEEIKK

QQLAQQEGREKPPLFYSRTQFSTLVTQEYVFVTMATCNLIGVLFARSLHYQFYSWFYWTM

PFLLYRATYAISPLSPMQSTLRYEGRLSKTEIRIFFTDIIGFVFIFGTWALQEYAWNVYP

STFWSSIIAVSCIAITIIGVYVSECRYPSANNTEAPTSNELQKYLAKVEKENEARAKN

>tr|B5FVB3|B5FVB3_YARLI YALI0B11913p OS=Yarrowia lipolytica (strain CLIB 122 / E 150) GN=YALI0B11913g PE=4 SV=1

MGAAYHILGKTVYPHQLAIGTIVSVVGGIVIASSGKKAEKPAAPAIQAGSSDEEKFIANF

LKEQEAAEKK

>tr|Q6C375|Q6C375_YARLI YALI0F02035p OS=Yarrowia lipolytica (strain CLIB 122 / E 150) GN=YALI0F02035g PE=4 SV=1

MEDAPPFIYLYHEMSDSIEPRPQQKGQFLWILGGLVLVVALRGILFSSLALGVLLGFLSA

VVLVLALLYVHNGNSTNGAYSTGIEHPSGSFKPIGFTLPEVWERHVTELTTEPAELPPIE

PNSFIISDSLDTLIEYIVRDFVMAWFGNVCKTDHMFGYEIDTALRSITKELSLRLKRIDL

AVFLILKLLPIVKTHFTDFVTADKTVRERAGTKLTESHEFNAAVATEFLQNVSEHPHPAV

AVKDMDNEARGKEWLRGVSQRLLLRTLPVEERNVKLVVLLLREILACTVLFPVVSGTLSD

PDTWNQLLVKFVEPSLEDKRNVQQVRDALDKHSAAQTTSPAPSEGSATPKKRPIKLSPSA

SQQDFERFIRKIKAIDTAQEAKQLRYNISVQLQRLDKEASGSYESGLYSGRLREAQSILD

AKLQDFGIASSASAPSSAPSSASISEKPHNLNAFEKFLRNTTATASSTISSSSSMSEEHI

VAALRHDKLKYGLSDVLNDPSCLQWFMEFADQRSRMVLLQFWLTVNSIKDPLEEFAGKTQ

EGLDLDNEDQSDDEDIPVGVTASQLKQSRDDVETIYEMYFQGHMEGPQLVVDPRSFNDVR

TFVESKHPTMDQYRQARKGILRTQHAAFQKLEKCDLEDFKKSDLFSKFLAIQPTEQQIAA

YHKKKKKWGVTDMFKKLDTPKRKPVSSSASSSHHDTKSSPEKRDRSSHNDHQDKRNSHHQ

FDGANTSLLDGVDVEANDDFNSHYSSLDQSQKPAADKSLIDLNDTQSQSEKVAPQSAPLI

SLDDEATSRPVTPPPQQPTSAGESQLSQSEAVQAALKSIMDSPSGTRDPLFDDDDDFGIF

GSKRSGSKLLEEGSRPGSKLFEGSRPASKLFDDSDYDESDVEKVQSSVVASLSQSLDEDQ

IKALDTADAIPPEEESFSLHFASPGDLGLTEAIKTLTIDIEKLKGQVKVLEPLLRKAELT

NNTNEIRILSKSVSSLQREINNKEMQRQQYIVQETDNSLYGRSAIAIQNAVSSHDKMGNE

FVVYLVEVTRHGDDSDKEGASSATWIVARRYSHFLQLHQHLIRSFPYISRIPFPKKKVVI

KFNQKSFVDSRKVQLEAYLRELLKMPEICNSVAYRAFLSSHNFVTKYKSVAEEGLRADAD

NRVSLRDELRVATNFFANDGLLSDSLNSNMIGANGSNNSSASLASESTQTATASAVSGTS

NSTATTAAPKKESKISNATFVKPISDLFVKLFRLDRGDGLILRGRAIVIVIQQLLGGTIE

RRVRDSLNSHVANEQSVSNYLQLLLNNVWPDGKFRSAGEVRSVHEKTSTRHHAKTLLEAA

IVDFSSKVVGTSNSKYAAHNLFMMLQNPILNRQLVLLILDELVNELLSEH

>tr|Q6C5I1|Q6C5I1_YARLI YALI0E17875p OS=Yarrowia lipolytica (strain CLIB 122 / E 150) GN=YALI0E17875g PE=4 SV=1

MTNLVLLVDQTQVAIDERERLTLLGIKAAESDDRDIERALENIKQGLLDNRQAATHGKDV

GTLTRVREDYHNLVQAYEKISDTDLPDLYLNESLFVSKKKSVKFRDNNLEETSGAHSLND

QHRDFEPYRDDPEALIPERYTDEEPSPAARFEGLSTQQVVYSQREDMGEQDERLSTLAQS

VSRQHQLSLQIGAEVDSHNVMLDDIEAQVDNSDGRLNLARRRLDDFSRRAKESGSLLTII

ILVFILIILIVLLS

>tr|Q6C5Y1|Q6C5Y1_YARLI YALI0E14146p OS=Yarrowia lipolytica (strain CLIB 122 / E 150) GN=YALI0E14146g PE=4 SV=1

MTQQEREATRAIRTTKAPYKPWISSPNVGLSFAYHLIGFISFASSFHFLEQYPNPLNASY

GGKYQYLTILGLCGSYLAFLLALLADAASLYSKDFSNLLYQLKNSVSVAVTPLECVITVL

YWSLKLYDPKLLKHPAVKEQIPLWLDMSIHLVPAVALFLDVVLMGQPWNLTTVQAVVANF

GLSAAYWFWVHKTASVNNFFPYPFLGLVSTEVRVGVFIGAGVIGYISYIIIRGLQSTFAR

SPLDQLKGKKSI

>tr|Q6C6V5|Q6C6V5_YARLI YALI0E05929p OS=Yarrowia lipolytica (strain CLIB 122 / E 150) GN=YALI0E05929g PE=4 SV=1

MAELRNRKGGVAAEPAAADSTPKVMVKPKKKSNAKYFQANLVLTLLAFLTRFIMLSHPDE

VVFDEVHFGKFASYYLQRTYFFDLHPPFAKLLIAAVGWLIGYDGSFKFENIGENYITNNV

PYVAYRALSASLGSLTVPIIFKTLQYSGYSVAACTIGAAIVCLDNAHINDSRLILLDATL

NISVALSLFCYVRFSNQRKSPFSLAWWTWLFSTGIALSCTISTKYVGVFTFMAVGGAVVY

DLWLLLDYEKSGLSMKDFLWHFGARAYALILVPFVIYLAWFQVHFVVLNRSGPGDAFMSP

DFQETLGDNFLAQQARDVEFYDIVRFKHKSTGALLHSHDARYPLRYEDGRISSQGQQVTG

YSTADPNNAWQILPEKDFPEDQRVGHPVYGSNKVRFRHLATNTYLLTHDVASPYFPTNQE

FTTVSPEVAAERYNETIFEVRLNTGKSNNVRTKAAEFKLIHDKTKVAMWSHSKLLPEWGF

GQFEINGNKNTQDPTNTWTFDEIVGLSPERAAFTPKKTRQIPFLQKYLELQGTMFRENNA

LTASHPYASEPITWPFLIRGVSFWTNAPNRAQIYFHGNFVGWYIEDIVIALFVGAIAADQ

FMRKRQITTFGRRASSKLYHSMGFFFLAWSTHFFPFFLMGRQKFLHHYLPAHLCAALLAG

AGFDFIFGEFDRLDEEANASKKDLEYTQRKNKPLHLATITVLILLTACFLFFCPLTYGLA

NQSVQAIQWRQWMKFELHFSK

>tr|Q6C708|Q6C708_YARLI YALI0E04719p OS=Yarrowia lipolytica (strain CLIB 122 / E 150) GN=YALI0E04719g PE=4 SV=1

MSNRLLGVLTTIAIPVGVGITLMQSAMYDVRGGYRAVIFDRLAGVKQNVIGEGTHFLVPW

LQKDIIFDVRTKPRNIATTTGSKDLQMVSLTLRVLHRPVISQLPHIYQSLGLDYDERVLP

SIGNEVLKSIVAQFDAAELITQREVVSARIREDLVKRAGEFNIALEDVSITHMTFGKEFT

KAVEQKQIAQQDAERARFIVEKAEQERQAAVIRAEGEAESAEAISKALEKAGDGLLLIRR

IEASKEIATTLAQSNNVTYLPKGGNGSNGASSGNSLLLNVGR

>tr|Q6CA20|Q6CA20_YARLI YALI0D06589p OS=Yarrowia lipolytica (strain CLIB 122 / E 150) GN=YALI0D06589g PE=4 SV=1

MANNERRKKNAPMGKRIGHVIRRGPFHQIIRPFRQNRNLQVSPFSRVWTRFEFHLSSGHD

TNSTALHNTSPTMLFVRTLVLAMAGLVAAKPPVEARTAVIVDKNTDLAQYSQFFDDAEHL

LDTVTVIDVKDKSFDLFTYGYQAYDMVILFPPQVKSLGPALTSRKLLEFFNKGGHILSIT

SPEHTPESIRDFAQEMGISMAPKFHKLVDHFGETGPNHDSLTLDTVNSVISSAENVVYSG

SAAYLSNNPQLVPLLTAPSTSYVYDYKEDEEDEENAGTMGTPWITGSQGFPAVGFQGKNN

ARFAWVGGDIMTNEQYTAHPGNRQFMKDVVEWVSVAKNVISPEYIRHNLAATPDVLNEKI

YKVNEDITYSVALTEWDNVKEDWVPFIADDVQIEFTMLNPYYRLTLEQTGTTNFSAIYST

TFKIPDQHGVFTFNLDYKRPGYTFIEEKTRATIRHTANDEWPRSWEITNSWVYLTSAVMV

VIAWFLFVVFYLFVGKADKEAVHKQ

>tr|Q6CB12|Q6CB12_YARLI YALI0C22726p OS=Yarrowia lipolytica (strain CLIB 122 / E 150) GN=YALI0C22726g PE=4 SV=1

MNTATTSTSSSRDDDQNREPTYLPHLKKSIDVLTYTYIVAGYFCDSSSALLLMRVIPQFL

FLGPRSLVAALRPPSYFGMLSYILFANIYAIIVLHGLLHQGAGSQLFVPSWTVSAPVGFP

TLWRWLRGSAEMVPAPVVPSLDETSKLMNHIYPGGVPGVTEGTMVEYINPATRNHGGLLS

FEFTGNVLESTGRLILSDLIILFLQLCLFSLIVMPHYKNSQSSDSTADDPLGQRLGTFSD

ESDESNSSQDPLLSGEETPLTPREQMIEDRISGRSVAGEIAIWPTISNVIRHRNVNRYHE

EGEQENASESASTHSLQEVGDSPLRFMRFQDLEQQQPAPPQNNNDPNNPPQNFGDTQFRS

NFMNRIGNWARSVGSNQQTGGQRLGTQPNEQ

>tr|Q6CAJ0|Q6CAJ0_YARLI YALI0D02343p OS=Yarrowia lipolytica (strain CLIB 122 / E 150) GN=YALI0D02343g PE=4 SV=1

MQQKGFDEIPADLPFAEMVFTVERALTKQISCSYSYEQLQDASHNVVGVFVESLRKETQT

NPGIVAALLVARMRLSGGSRDVLSGRVCKTRALVAELIAERYIERLNTEKERLMFVVYEA

VEEEGADQHRHAASRNFESESLNQDGYTSFHSDIEDVEYFNDGNERFDTGNEDYGADYGS

SDTSFDPDNPGPNPKYFHDEYMYHTTALELAVYSSAKHFLANPTIQQILNDIWTGKIVFW

KDINSQSHKEALIYPTCYVLATRRIPDAYTRLRVPRYRQFFMTINYIILLGLYFSLLFIQ

KKADDYFEMNFTFVEVVLDLFFLGFVVEKIAQEGDRYWSTFDLLVNLDFVVFLVLRIYAY

VINDIWLTKLSFDVLSLEAILLFPRVFALLSIYPYYGILLPCLKTLTKDFVKFTTIIAIC

YFGFLTTFAFLGRGDFTTNEMFWLLVRVFFGSSFAGFDEAPKIHPMFGSLLMLVFVMLTN

VLLLTVLISILSTRFSKMMNNAREEYTLLFAESVVEATSDRVTYLYPPLNLLPLLLRPLR

LFLTKTQQRRVKIVTLKLTHFPLVFLIWSFENFVVFYHRQMNRRAKKLAEKRWVKARRRV

YVEEEGEGLGDGIAVGVRW

>tr|Q6CGY7|Q6CGY7_YARLI YALI0A14861p OS=Yarrowia lipolytica (strain CLIB 122 / E 150) GN=YALI0A14861g PE=4 SV=1

MASIDTSTSSGGDEKVREHPYKRTLTGHALPYNRAYPNRPEGRAKQPFLRHYFRPSWVVD

HLDFASFKVASRTWVGIWFGLMLMMIPGYVKWLGRAPYLTAIIGVIVPSGDMSVTVCFIV

NAGMMLFTVFGWIVMIVAMYINNKYFHDSMTPKQMTQILIERGICKAPTDPTDPKAMAAL

QTCAQGAVFDAQFMTAKGSIVYAVLMGCFAFFLLWVKNKSRIIIPGYVCGIIAMSVLLTV

GPMVPYYQPITLGVVLLKPVGLQCAINFSLSVLIFPFTTGFQFTKTVIGEISVLSGLVDF

HQQFLGSSLPSNESEWLKFSEIEADVTKARQLYPKMQSESAMLHLELSFARFCHHHYEGL

KVAVGRLTSSMAGLVYFYDNIENIRRAIIISAEADEAIRAVPSQEESETVPPVGVYEMKS

GLRNRTALQQKATLRELTMQDLDLLILQLRQMSTPFIQEVCKSLDVIKRWLEAANVYRGN

SLFFFWMSKSFKQQQKQLSEELRERYADFQRESKDLLESKRFALFGEHEENTKFVTFFFQ

GALYCGYMKNLSDSLDFLYKILLDIDEKHATPSWRFGKGIKKAKDNKAALYIMNHYARAD

ENHLKTTHNEDLDDLFNQTIHYATDRRRNPDAMPPSTPLHKFGARFRKEIIKLGSPSLFV

PLKGAVFTVLSASPCFFTASAPWFYKNKIMWMVIMTGLSISENMADNLYGFIARILYSFY

AGVIAMVAWYISTGNGNGNRAGFMVVICILYFALSYYREFAQHTTPMPTIVLVVTTVIIL

GLSWTDGMGITTPTIGVGYAVAWKRFVTVVMGLTVGFVCSCVPRPVTGKRLIRKTLASII

KDTGTLFCRISDFGATRLANPDIEVNSSSINDPIFGAISNGQSRIAGTRFLASMIQFEPS

LQGRWPSKTYREVTNIVSELVELHHHLYVVLQQIGEPRYWLPHLLKLVGWKNQPLMSHYF

AVLYMAQGALYDGSPLPQVTPAMLMVEHLEILERQFKNTYDTTAREAMGDTSDMASDDGT

PGLEQISLQRLKTTDGLYFSASIVLSNCIFDRMDRLMFQVKTLVGEQFVNADYYRDDEVV

>tr|Q6CH11|Q6CH11_YARLI YALI0A14212p OS=Yarrowia lipolytica (strain CLIB 122 / E 150) GN=YALI0A14212g PE=3 SV=2

MSIKSLSKGSSLDDTCVGISRVATPTNLEKDLSLWETMKIYKKATFWSFVMCCTIIMDGY

DGNMVPSFYALPVFQKKFGIQLPNGDWTVEAKWQTAFLVGVPIGRITGAVGVGLLADRFG

RKKVTITSLAFMVGVTFVVFFATGKEMLCAGWIISGIIWGVFNTMAPTYVSEVCPIKMRS

LLAAAINLSWVAGQFISTGVVTGTSTRTDDWAYRVPLAVQWVFPAILIPTLCFAPESPWW

LLRRGEIDKARNALARLADETKIDLDAHLEHMIQTDKEEDKSGTIAECFKGPDLHRTEIC

SMVYSIQPLSGANIINFFAFFFQLTGVPQDIIFKLTLGLTGLGFLATLLSSVPIARVGRR

KIMLTGLFVLTVALFAMGILGCFTSRGTNWATAILLFVWVGTYDLSIGPGAFVIYSESSS

VRLRSKTIAIASVISSTVTLVFNVAVPYMLNEAEANMRGLVGFVYGPLCILSLIWVYFRL

PELKGLSYMEIDRLFEGKKVAKDGV

>tr|Q6C2X9|Q6C2X9_YARLI YALI0F04191p OS=Yarrowia lipolytica (strain CLIB 122 / E 150) GN=YALI0F04191g PE=4 SV=1

MRWTNMLLLASAAVAESTFWVSPSAEQNCVGIYAGSEESPASISVYTPKLDNSSVINFMG

HEGWMALAIYKYEDLPFIGDMGRKEWGYKYHCNSMNVQYKLCTHDEIGQYIVKQNYTGNT

IFTTKVDLLEDTTFNYTVNETGLYCVTAHPNNEMNKFMSTVTVKDGNGQLSGYDYNKLAY

HGVMSGLWAATLIGYLVLCFLRKNLLPVHKRLLALIAVNLAVSLLWTIYNAIYNAQGPTS

FVKTLVVISAVISALRWALSMWLALYVAHGMHYFSTPLIQSAKKKAVAVTATFWVFESIV

ASMFYLIGMVQGTYVRMAIGACLCFVLYSTLYIFYNIYSWMNHTQKSYIDENMSVQIDQI

SKLRAVYLAFLLLFAFMVLFNIMVLLRGSIVGSTLNLIQTTNIAWVNEWKYRWFFVHDWI

ELANLLVVLAALWVLRPSRYFNQMMDGSGQISQKDYDEFELDEYDEGV

>tr|Q6C5D6|Q6C5D6_YARLI YALI0E18942p OS=Yarrowia lipolytica (strain CLIB 122 / E 150) GN=YALI0E18942g PE=4 SV=1

MDSSTVTQSEVSELSFAGDQVSSGVESGLEADHSEGHESEKNGLNEIRHRNTKPWPQLVN

PNTKQFKHGRTRSRGSLGTLDRPELTPVETSQQKTRNVIEVFLVVLPAAIAIYHERDIDL

TDPKYTTLVLSLQVWVAGLAISLSYCYNSGIFVANTLYLALIPPLVAYTTNKASTTLLSV

NLCLAACTLPYPTMVWDKFLDFNIVGPTLALEYVTPPFRTLKSTYDYVEQAVSSLTQESL

TEVEKRLLSSLVVNVVCMHLFRDPSPESIVLQCLFLGSVFCIFPTYPWVERSIAIMRTPR

HRRPLHWEKTLTKLSVAILGTFFVLMIILTVPVCTHLLGVTPFEFMWLYLNPNEETGVDH

TRLYMMAYWALFVAAIPVIDKIGNKFKFSTDIRRKIWHFCIVGMFLPCGVNTDSTFTMLA

MGCTFVLFIWVEFIRATALYPIGSQIHEALLAYVDERDTVGPLVVSHVYLMLGIFLPILF

HKSPIGIVCLGLGDSSASIVGRRIGTIKWFDTKKSVQGTLAFIFMASAGIYFCQQLIPGY

THSVISDMPLSKIVATTTATALLEANSDINDNVVVPVYMYLMAEVLTK

>tr|Q6C6Z9|Q6C6Z9_YARLI YALI0E04961p OS=Yarrowia lipolytica (strain CLIB 122 / E 150) GN=YALI0E04961g PE=4 SV=1

MSLEESWIDVDQKVTVKPPARYSARMPNVEALETESSALFYHAPSVYIIESDAEEDDEYN

CDSRDETTDDRTLLFSRDSRNSPSSRDSTTTTTSIAALQDSLSTIVSRDMKQARACGSSR

SKPPSSVCLDSLRIVPESQLPKMRARGSGTVAPAMSSTDSDSDTKMAVKKRRVKRVRTGV

SSPSMEVSGAGSPGGTGGFSPAALAAGITIIGLSFSAGYAIGRQSLRTASVN

>tr|Q6CDJ3|Q6CDJ3_YARLI YALI0B23496p OS=Yarrowia lipolytica (strain CLIB 122 / E 150) GN=YALI0B23496g PE=4 SV=1

MAHTQASVALAIALTLWYLAYQLWLMSLWLWLIALALACSFDHVWRLSKQIFALQLAALA

FQLGSSDSRRLLIQEFHLWERSLERSFTLENLCRWLCRCFTVWLFALAAAHVVLKNSQDT

E

>tr|Q6CGP4|Q6CGP4_YARLI YALI0A17534p OS=Yarrowia lipolytica (strain CLIB 122 / E 150) GN=YALI0A17534g PE=4 SV=1

MKIPGTVPLVESTEIILSPTPSSDPDDPLNWSPRRKLLSMFCMVLYCVAFCVPSASIFSV

FIPISQNTGLPLATLNQGTGYMFLLFGLGCIIVHPLSVKYGKRPVYLISVLGTALIQLWG

PKINSSGTWIGSKVIQGLLGASVESLCEVTVSDLWFEHERGRWIGVYGFALMFASNIAPV

FAGFITQSMGWRWVLYWGSIFDAVCFLFLFLFFEETNFRRQGEQSLQADSELENETEAGE

KKKQVQFITDVEVGQLFEKKSFLSKLSPFSVSKETILLPMLVRPFTLVKYPVVLFSGFMC

GTGLICFNICNATTSFVLSNAPYNFPASRVGLCYLSPCVMVLVFSFYGGYISDKLRVLLA

RRNGGLSEPEHRLWILSAYLLLCPPALVLWGVGASNGIHWFPIVFGMGLVKGLGTLTSIS

AINYVVDSYRDMTSDSMVLVMLIRNTMSFCISYGITPWFMNEGLTRCFAEAAGLAFACCA

TMFIFVRWGKKMREVGAERYWRMVEEK

>tr|Q6CHI2|Q6CHI2_YARLI YALI0A08558p OS=Yarrowia lipolytica (strain CLIB 122 / E 150) GN=YALI0A08558g PE=4 SV=1

MKCNNRKQTNNVHLLIRQDIKRLIDFDQRKPFIHTVHTQLFLQQRSDKRNNIVKSVNMCT

CTVTVFFKTPPLSIPTVLLLNTPSGILTHPQVLPTHTSPTYPIRPIRTQSTRPVSCQCKQ

ITHKQPLSPLKMISVYELAATVLILALYTAYLGNLPTFDYHTAREHKLSKVRILKECIYR

LDKEIIQPCEEVERSLRDLIQEYKHESGARKPTVTIPIFKRVRRENLRLFRYAPKAKAFV

RVQSPKEKPHPQQRLPKDTNEFDVTNLRQVNKFGVNSDKDPYWHPQPRHPLSSLSCNPST

RRYPPKLHA

>tr|Q6C8K6|Q6C8K6_YARLI YALI0D18876p OS=Yarrowia lipolytica (strain CLIB 122 / E 150) GN=YALI0D18876g PE=3 SV=1

MFWKNMKNEPPQVLNWTLWLAVIVFGSLGSVRGLDEGLISGITSQASFESQFKLKDPLKS

KSQQDDELSNITAMVQIGSVGGAMIAMLIQDKIGRIRSLQEMLILWTVGVIIEVTSYSQG

QMLAGRLIAGLGIGQSVVIGPTYLAEVSPKNVRGLCTCIFSGSVYLGVMLEYFANYSTSM

HMPATSRNQWVVPVSIQFIFAGLLFIGSFFVHESPRWLMKIGKDEEAIETLSKIRNLPAD

DLYVQGEIMDVREQIEREKQALSGTNIFSLMKELVSTKANRYRLFLGIMVQLLGQWSGAN

AVTVYSPKFFSMLGIPSKIDQMMYTAILGVIKLVSALSCALFLIDTIGRRRSLYTGICLQ

FVSMLYLGIYLAIVPAKTGVERTPSQRHAGAAAIAAIYLSGCGWALGWNSIQYLINAEIY

TVRHRSLASGIIMTFHFANQYGNSKALPYMRAGITDHGTMFFFAGVLLLGFAWSWFFLPE

VSGRSLESIDEMFSLPWYVIGRRGHKMIPETSATVHLRQEEDSESKKGGVVMVESC

>tr|Q6CA80|Q6CA80_YARLI YALI0D05137p OS=Yarrowia lipolytica (strain CLIB 122 / E 150) GN=YALI0D05137g PE=4 SV=1

MPAGKAVPAHHEAGKVKGAVDEIITGSLIGGVQGAVFGAASHFLAKMYWPHYGKLTKQFK

LFLQLSCVITGGVYAADKRLIRYEHKLRIDQRNLIAERAAAAEARGEYIDHDGHVRDSSG

KVIRK

>tr|Q6CDV0|Q6CDV0_YARLI YALI0B21010p OS=Yarrowia lipolytica (strain CLIB 122 / E 150) GN=YALI0B21010g PE=4 SV=1

MQFKNPFVKEKAVSAASVDYETAEVSSTEGLSATEDEKFDGVLDGSGDHKNPFTDPNVAE

KFRQLYEDSQYESRHVFDPDFEWTKDEEKRVKRKMEWRVAGWACIMFWGLQIDRNNLAQA

VSDNLLEDLKLTTNEYNYGNMIFYLSFMSAEIPSQLVSKRLGPDRWIPIQICLWSAVAMC

QGALQGKSSFYATRSLIGLLEGGFIPDLVLWLSYFYTSKELSIRLSFFWTGLYLVNVLTS

LLAFGILRIHSGGLAGWRWLFIIEGLITLCIGLASFFRMPASAVSTKKWFRPNGWFTDRE

EKIVVNRVLRDDPYKGDFHNRQAITPFTLYKCLSDFYMWPIYAIGLLAYIGDSPEGTYLT

LTLRHLGFSPFNTNLLTIPVSVLKMILLLSFTWLSEKINERSLVSIIQPLWVMVLVIALR

FYPESGINVWCTYTILVLLLGGPYIHAVVVAWTSRNSNTVSQRTVSAAMYNMCVQAGGVA

ATQIYRKDDAPFYHRGNTQLIIISGATIALLIVTKIFYVTINKRRDRKWSAMSKEEREEY

AQNSTDKGSRRLDFRFAH

>tr|Q6CFS0|Q6CFS0_YARLI YALI0B04378p OS=Yarrowia lipolytica (strain CLIB 122 / E 150) GN=YALI0B04378g PE=4 SV=1

MPSLTWDIQKPVREETAPIRGGPSNTPDHNYGALQRPCTVQQEEDRLIGDFYKRHNSVAG

DKPADTGDEKLGTFSGVFMPTTLNVLSILMFLRFGFILGQVGILGMFALLVLSYAIDLLT

TLSISAIATNGTVRGGGAYYMISRSLGPEFGGAIGVVFFFGQVLNAGLNVAGFCQPILSS

FGQNAGGFFPEGYWYEFFYATGVLLFCTSICMFGSGLFSQAGKVLFVILIVATVSVPLSV

FFVKPFLVTKLDIWYMGPSWDVFSDNLLPRFTTGAVGSDLPPGQMETFTSLFGVFFPATA

GIFAGASMSGDLKRPSYSIPKGTLSGLGLTFILYAATILGMGVAIPRALLYKDISVIETV

NLSKWLILFGEMSTSLFSSMVGVIGAAYVLQAIAKDALVPYTSFLASEINGLPIPAVFTT

YILTQLTLFFPLNRLATFITMAYLMTFVVTNLACFLLKIASAPNFRPSFKYFSSTTAFLG

AVSCIASMFIADGWASIGAIVILAFLFILIHYVSPPKPWGDVSQALLYHQVRKYLLRLRQ

DHVKFWRPQILLLVDDPRSAWGLIKFCNYLKKGGLYILGHVVITKDFQETFKEVKKQQQS

WTKLRDMTGAKAFVQIACSPDVVWGARNVFLGSGLGGMKPNITILGSLRDKSQPLDLHRA

QTIDMEALPTDNCRKESNIRVTQWVNIIEDIIAMQGNVAIARGFMGMELPGKNIKTTQKY

IDLYPIQMSAQVVDENGTSTMTTNFDTYTLILQMGAILRTVPLWKERYTLRVIVFVEFED

AVEEERERVSTLLDTLRIKAKILVLCLNSGNYAAYECIIKGTSNPAVESKLSEQDWWSEL

VEARESNKPYAFTRKMEEHKVIAIDHKRRHTFSSLSHLGASFSLRANSISSAGTFDSEGF

SDYDENSSVIDDEEDEDEDDTSELEQNTSALHRHNSLFSTSRPLDRVKDKSRDDESSSGR

SSPSSSTHHERHNSNKPAFTSQAIPKTKVNDNDDDEGNTVMFEHGDDPAHPNVLSFNDVP

ARAQHIILNDMMATLSSKEDTVVIFSTLPAPSMGTHRSERESLDFVDSLELWCQDLPPVL

LLHCQTMTVTTAL

>tr|Q6C6V3|Q6C6V3_YARLI YALI0E05973p OS=Yarrowia lipolytica (strain CLIB 122 / E 150) GN=YALI0E05973g PE=3 SV=1

MKNDYKNSIEMESIAGSVSEESIPESSLPPYTEFDKPAEPCSVFKFTIKSDLPYIFVGIV

CCALEAGVGPLQTVLMGKIFDTLAKHMKGTLENKFMAEIGKYCGMSLGLVFLGFCTDFFS

NIAFIYYGDRQLLRLSRKIKDTFVKYKSMAWYDHNQGVQGSLNVAFRNIEDIQGACCSAI

AFAIKDVLTIAISMGVAMYHSWSLTLVIMAGLPIIVLVAMGVAPRLQRHFRDYKGVITDA

SVMIDWSMSGLQHVKLSNGEKKQMSILQYQMHLATICYMKFTTWSAAQQAFMQVLALIMF

VQGFWFGAKQVQIGNLTAGAVMTCFFSAMAVTSHIASITGQMMSIMKAMVSAGLVNQLIN

SAKPSKVDVRQYPEKCDGNIIFNGVTFAYPTRPGVPVLKNVKLEFKQGQTTFVVGQSGSG

KSTISNLLLQVYDSYDGEIRVDGFETRGVSTRWLYESINVVRQSTALFETSIRENIQLGR

GAEWKSATEHDIDKACQFALLSSTICDLPLGLDTKTTNLSGGQRQRVALARAYVRNSPVL

IMDESLSALDIVFRELMVEAIRKWRKNKTTIVVTHELSQIQSGDYVYLMRDGEVVQSGLR

KDIENVGYFKELREQGQTKEADKEAPPVNEIGDFDQNMIRNTYIPMKTTRGVRKWVDVKP

PPQRDVLDRKRKPVGYFEFSKLVLKTIPNKPLFYSGLLLSFLLGAANPAFGWTISQVISQ

IMPNGESDSSLNKTLVKWSLIVIGIALLQGALAFLHTYTLERAANGWAVFLRKSSFKTVV

TREMSWFSRAVDSDHDTPDIGPFHFDRNSSGITELIINETEELRSAVTTFFSAVISIFTI

CVVGFIWSLVQGWKLTLVSFSIFPGFLLTSQLYAYVSSVWEMGLRERIVRVQSLIHECVT

GVSEVRILNLENYFEHKYIKLEDAVLKYAKMRAFLVSITYASHQMFTPLLQVIILWVGMK

LINDGEYTMEKLMGVMVILIQAMTTAAGALETIPQMSKGMNVTKTLFALMDEGRAETSED

GGTECPDLHGEIVFRGVDFSYQSRKEAPVLNNFSMKVASQEAVVIVGPSGSGKSTITNLL

TKLYPYRRGIVSIGRHDIRDIDTAYLRQRLAVVTQSAAFYNGSITSNLTYGCAADPEEIE

RVCKLVGIHDFIMSLSDGYNTVIGNETGSGTALLSGGQSQRLSIARALLRKPQILILDEC

TSALDNASAKVVHELILKLKKMRKVTMIVITHSHELMKLGDRVLVLGAHGEGVVEQGPYK

ELISHRGPLYTLVNGGICH

>tr|Q6CBA8|Q6CBA8_YARLI YALI0C20471p OS=Yarrowia lipolytica (strain CLIB 122 / E 150) GN=YALI0C20471g PE=4 SV=1

MDQLLSLLPRLFEGNGNLAGLISLVPFGILSTVLESPYGPALVEVLNSQDTLPLDYSDRL

LSLFPIPPFAYTGTGILKIVVMAHNVVGNNIPQYVLDMVMTTQYNIFGNFPKKDVDTIPC

AIFLVINCVLALANFYVFMRGIMRNHNFYLMFGLGWQCLFNCLGFGMRLGWAQDILQLRL

GIGSTVFIILSIVTINFMNFLLAHRILTFRHPETGDATWFGMLMILIYLAFCGVLLMAIV

TQIVIFSYFLDYMHWRQATSGMQASSILIVLFSAGGVLIIIAAYIIPRGAIPLHHKSRLR

LSASNIESYGLFYFPPKHSQVVQYKSDPAMKLDSGQLAARTINGRDLHTSSIIVIFSSLI

LAGSAAMRCATIMIGDRWEMDAKFIYSQTAFYIGFGVFEVIVNVIFLVFRIDLRFYIPDW

PKKGRGGIVVHRDTKEIVKEMDAEARGDKRDFVFINVPTPAPTQDWIPPDDSNTYGDLKG

ETRSFDFDDKEKLAIVEIMPVPGDDTDVPQTPMVPDKTYVSMRSKSPQSLVPSYASPRTY

ANGQQLKNGETIDGFVMTSKDPEPANETIPGNPPTLAAATRPTVISPPPSASTPATPILP

VLVETAENGSSHRSFSPQSREVLSMPSPSYSDYNYYARPYEERRSELYDV

>tr|Q6CD98|Q6CD98_YARLI YALI0C02541p OS=Yarrowia lipolytica (strain CLIB 122 / E 150) GN=YALI0C02541g PE=4 SV=1

MKWLTSLRNSVVGGPHLYNNDKDSCSECDSDTESVSWTRKMFDPPVKQQREDYTDSEDEA

GAPSGSREMEQLTTIWTKTDLYMAWASMLLLSVAIGMMIQTVSVYSTYATSDFNQMSLLS

ALNVVKSVMYIATRPILAKFADVLGRFETVLICIVIFTVGFIMLSASPNIGTYFAAHIFY

IFGQVGIQFMEQVFAADTSDLKNRMFFVVLPNLCYLFTPWCAAPITNAILKHSTWHWGYG

MWCITVPVVSIPALTIMFRHRMKARKMGLECARSGLSNVKTAMRQFDIIGLILFTGGISL

LFLAVTLVKTSKSWSQPHVLSMIIIGPILLILFPIWEKLYPKYPFLPFSALKDRTLITGC

IIVALYSLAYYIYAPYYTAWLLVCKNLSVTAATNTRVTWTVASTFGSLLVAPLIRYTYRL

KPTIILGGCLYMLGLGLTYRYRQPDNSLAQFIIAQVIEGLGTGMIQYPVLVLIQAVVSHK

QVVAATAIFYSSISVGGVIGDAISGSMYRQQYPKRLADYAPFLSEKDIDTMVNNVNAPLK

YAWGSEERTAIIAAFNNVYRHLLYGPVIVGGALILFAVTLPNVDLSQMEERVKGVVFGNT

NKKETERNDDENQDLGSEKSCQKDNVVETKV

>tr|Q6C8G9|Q6C8G9_YARLI YALI0D19756p OS=Yarrowia lipolytica (strain CLIB 122 / E 150) GN=YALI0D19756g PE=4 SV=1

MFLEKFALEVPQGAAYENTVWTNRDLVPIPAERRTWNAWGYIGYWTVGGSCVTAWTLGST

MLAHGSNAPHAIGASVIGAFLTGLLAVGNGLVGDRHHIGYTVSCRATFGMRGSYIPIVLR

CCITSAWFGLQAFWGGQAVKALIGSMIPGFITGDLDNLFSESSHLAKNDFIGFWIWMVFF

VFILFFLPPEKMQVPFLCSFLLLMMSCFSLLGWTVGLAGGAGPLFSKDWKPEVHKNRAVG

WTMIFGVSSVLSTWGTGCLSQSDWTRYARRPSAPRWSQMLAAPLTIILTASMGIIVTSAS

MVIFNGKIYWNPIQMLPLLQKHLDHSAKSRAGVFFISLGFAVSQLALALVLNSVSTGMDL

AGLFPKYISITRGAALMVIIGIAIQPWQLVANSAKFLTVLAAFGVFIAPITGVMIADLLV

VRRNKFVIYDLFHTMQSEEEESNTADGKSIYWFFHGFNVWNLISIILGMWPMIPGLANAG

IMSSDERHYKHPEIPEPLPLSWRRVYNIGFFVGFAISFTMTCVGAFIWKPPGVGKHSTWC

SGDETDSDKEMSYEEPMMEAVETK

>tr|Q6CDW9|Q6CDW9_YARLI YALI0B20592p OS=Yarrowia lipolytica (strain CLIB 122 / E 150) GN=YALI0B20592g PE=4 SV=1

MSFAEMSIDEAPDDEPLVTGFSGFTLSDQDVRNRQLQYHRPLQQQQPFDDLTSEFSNLRT

SETQDPDEMDIDDPTDLLDLQSVNLSRMNPEFLPPVSTPIKAGKRRTSSVSVAAAAPNSP

WKISSVFNPTELGAQFAVKEIEKNEDVDMSMVLYSPVNEGKPLDQADDIDPDLRPDAKHG

DFSPQSAQAAVHTPAPTLHTSEAQSPGQDQSAEYHLHLPNPTLGWMIQNPNLPYIITSYL

QLAFNGIMLAVMAYVVYLFISSVRQDVGFKFSAHSGEILMAKSKCAKLYLLNECAANSRR

PALETLCDQWELCMNRDEHAVNRARLGAETLAEIITGFIDRFSYKALGLISLLFLGSMFV

SNFAFGFFRAKAYYNGEVEKQNGLWDKARYTWGGRKEDEPKRLVFGQQNVLQN

>tr|Q6CEP3|Q6CEP3_YARLI YALI0B14124p OS=Yarrowia lipolytica (strain CLIB 122 / E 150) GN=YALI0B14124g PE=4 SV=1

MHLFVLIHGLWGSATHMAAVKEVLDTTYGVKAGGDMVAYATQSNHGTLTYDGVQVCARRC

YLEIKEVIRRYADDEGVTFDRISILGYSLGGLIARYLCGIFLDEGFFDKVKPVLFSTIAT

PHLGSKFHRTDKRWFSWMNTLGSTYLGNTGRDLFLKDPTLADMSNPSSSAYKALEMFDNR

VLLANCRNDRTVHFPTAFITAANPFANLRWLDLKFHEVKLQEPVVDSYGWSHSPRIIDFK

RTTKRSLPFPVYHEHIRQKLIFWSFAATVGPLAITAVLIASMFFTNNSNHRVSKFLREGD

KALMGEILESIKMETFDEEWLNEGDERKVGGKGEKTENTDKQSSPVSKTDASKVEEEADV

MAAGITEAAVEDVILGDDLNNTDTPALSETKVVGLGEAMEAARHTGDRGLLANWANNAPF

SEDVLSMIENMDKLGWEKYAVYITVIHSHAAIVARRGLTQQGQGAATLRLFSEIIRSKI

>tr|Q6CFY4|Q6CFY4_YARLI YALI0B02544p OS=Yarrowia lipolytica (strain CLIB 122 / E 150) GN=YALI0B02544g PE=3 SV=1

MTDPVPITQDPTIYSSQQDAEIRSLAESIHSQHSNNSNNSTELTNPYVDTSDPELDPWSG

QFNSRKWSRTILGLKRRYGTSKEITAGVSFKNLGAYGYGGGADYQKTVANAVLGLEGVVR

TLFHLEKKEDKVQILSDFNGVLWPGETCVVLGRPGSGCTTLLKSIACETYGFQLDKETEW

NYQGIPRKIMQKTCRGEIVYNAEVDVHFPHLTVGDTLMFASLARTPQNRFDGVTREQYAK

HTRDVTMASLGLSHTLDTKVGNDFVRGVSGGERKRVSIAESIVCGSPLQCWDNSTRGLDA

ATATEFLRWLRHSAELTGASMFVSLYQASQEAYELFDKVTVLYEGQQIYFGPGEQAKQYF

EEMGFECPHRQTTGDFLTSITSPAERIVAPGFEGKTPRTASEFAERWRQSQAYANLQEEI

ERFNTEFPVGGNRVADIMELKQEKQSDHIKVSSPYTISIPMQVKLCLTRGFQRLRGDLSM

ALTTVLGNFVVALILSSMFYNMPEDTSSFFSRGALLFFAMLMNAMSSVLEIIVLYELRPI

VEKHQRYAMYHPFCEALASIICDFPTKFLTMLCVNVTLYFMSNLRREAGPFFIFFLFTLL

CVLAMSMIFRTIAAVTKTLQQALAPAAVIILALIIYTGFTLPISYMRGWARWINYIDPIA

YGFEAVMVNEFRNREFPCALFIPQQSTYDQLGSPYQGCMAVGAKPGERFVNGDRYLEMAF

DYSQAHLWRNLGIMFGFILFFAFTYLTAVEFIQSAKSKGEVLVFLRSSLKQRKKRAHLMD

VEANAEKVGAAQDREILVQQEEGQQEETSSCTPSDSTPKDIFQWKDVCYDIKVKGGEKRL

LDNVDGWVKPGTLTALMGCSGAGKTTLLDVLADRKATGVITGDMRVNGQKRDASFQRKTG

YVQQQDLHTATSTVREALEFSALLRQPSNVPKAEKIAYVDEVIDILEMQAYADAVVGVPG

EGLNVEQRKRLTIGVELAAKPELLLFLDEPTSGLDSQTAWSIICLLKKLANRGQAILCTI

HQPSAILFQEFDRLLFMTLGGKTVYYGDIGANSSALINYFESKGADPCPEEANPAEWMLA

AIGAAPGSIAKHDWAVVWNESEERARERDLLDKMAEELAAQSTHDEKNELVTSKSVGSSQ

TSSSSYSAKSQYATSQATQLYYLTKRLWTYYWRSPRYIWSKLLMSIASALFIGFSYYKAS

QDIQGLQNQMFAFFMLFLIFVIIMVQILPHFVAQRELYEARERSSMAYSWQAFMGSNILV

ELPWQTLVAVLVFFCFYYPIGLQNNATGHLGERGALFFLLLWSFYVYNSTFAHMMGAAFE

NKENAATIGYLLFALCLIFCGVLATKEDMPHFWIFMYRVSPLTYLISGLLSAGVGETRVE

CTDNELVLFKPMNGTNCGKYMHPFMEGLGHTDMPMGYLVDPSATDMCGYCPISNTNGYLD

QIDVKYSQRWRNYGILFAYPAFNVFMAFAFYYIFRVPKKSRKQKA

>tr|Q6CGC7|Q6CGC7_YARLI YALI0A20416p OS=Yarrowia lipolytica (strain CLIB 122 / E 150) GN=YALI0A20416g PE=4 SV=1

MAQVTPSSPPPVYDAEDDESRQNLLHESRDSSFEIEQFELDDSPSGPLPGGSHPNASTST

LARAQQVAQNVATYSSKLFTPVKQLLDPVARFWHKCSVAMDVELARWGNPLILKRLVYLV

FVSTFVILAILFGLVPNNRNSFGLPDAMATREDLLTYMREVLESSEMKSRLDYMSSMTHA

AGTSGDLTLAKYVANEFKRYNLDNVEVKEIKGYVVMADNGQVGWEVVGEQGNQKREDQFG

QTPEENSVPAFMANSHPGEAEGHAIFANYGTDADFKALEAAGIKLEGAIVFVAYNNVPTG

LKVQTAQYHGAAAVVVFSNDPENADAVRRDTTTNVLMSPGFVNFPGGPRSYRPSKGVDPG

DATAGSKIPVVPASLKDVAPVLQKLKSGARYDNDGVRYSGDDKSNVRIKVRNNVKTDEQH

PLWNVFGRIEGNQQPDLNVIVGSARDAACFGAMDPMSGQIVMLEAARIISGMTQHLQWYP

ERTITFASWDGSKQGFAGSNERPIVFGHDLCDETTAYINVADAVTGSEFQAHGHPYFNNI

VRKALVEVKSPQNESFVDHWPEKRVKQFRQAGDYMGLLSTCGIPSIDVGFKGSSFPHDTC

KDTVGWRDEFLPDSIDYLKAMVEIVVRLALSSADDPVLPIDTAYYASEMRSYVDDLIKYS

EEADPNFVHDFRGLDAAVARVETFSQKFIDATSQYSEITRAMEFPNNAAIRHSNNIALRK

FESRLLEPRSPEEPRSWAQHCIFSPQAVPQEDFEWYTFGNIRDQLRLKRHAKAQELMQNA

AHRIYKIYGNI

>tr|Q6C5L2|Q6C5L2_YARLI YALI0E17105p OS=Yarrowia lipolytica (strain CLIB 122 / E 150) GN=YALI0E17105g PE=4 SV=2

MRASTDAELAEQGDNIAPLQEVGSNIDTTQSKRHRFGTQRRTHSVNRRRSLFQKLVHPER

VEVDNTHEITDEAHGPSRTVYFNEPLPEDQKDPKTGNPLAHYVRNKIRTTKYTPITFVPK

NLWYQFHNVANIYFLLIAILSAFSIFGVQSAGLAAVPIIVIVVLTAIKDAIEDYRRQILD

MEVNNNVTRVLDGIENPNVEEDDVGLWRRFKKANSRFWGPKGRAFVRLFMPKQRRIESKE

KEQELHRVLSNTTVITARMSSDLGGRPSGVSRGRRSDVRRSDIGRDVRPSMDLGRPSMDR

DQRRSSVRFSGDFGEGPAGDSRTLRASTDNRDSKNPGEFSDDPTVIDYNQRPEGVAKFRK

KYWKQVNVGDIVRVLSDDEVPADIVVLSTSDDDGACYIETRNLDGETNLKVRQALSATKG

IRHASDFERSHFEVMSEPPHANMYSYNGVLKWRNTDGGAQSEPINSNNLLLRGCSVRNTR

WVMGLVVFTGDDTKIVLNTGETPAKRSRMTRELNINVWSNVVLLAVLSIVAAAVQSQHFR

RHDTSDHFFEFGIVGGTYAVGGLVTFFTFLIVLQSLVPISLYISIEIVKTCHAFFIYNDI

DMYYAPLDYPCTPKSWSISDDLGQIEYIFSDKTGTLTQNVMEFKQCTIGGKSYGKVFTEA

MLGLRKRQGANIDTLKVEMEQDIADDRQLMAREMAKVYHNPYLTAEPTFVSSDIIRDLEG

ASGPDQQKHVHYFLLALALCHSVLPEVDEEGVLVFKAQSPDEAALVSTARDLGFTVVERT

RKSVVVDVMGKRIEYDILAMLEFNSTRKRMSTVVRLPDTGKIVLLCKGADSVILSRLNRQ

INESSLVEETARDLDRYANEGLRTLCLAHREISEREYEQWYSLHSEAARAIENREDKMDE

VAEQIERDLRLLGGTAIEDRLQEGVPNSIALLAMAGIKLWVLTGDKVETAVNIGYSCNLL

DNSMELITIQVKNPTVESVGAVLDEFAAKYNIDTSKEALKAAKKDHSPPKNNAAVVIDGD

ALTVALSDPLRIKFLLLCKNCKSVLCCRVSPAQKASVVSLVKKSLDVMTLAIGDGANDVS

MIQEADVGVGIAGVEGRQAVMSSDYGIGQFRFLNKLLLVHGRWGYRRIAELTANLFYKNI

VFAMTIFWFQVHTAMDGVMLFDYTYITLFNLAFTSLPVILLGIFDQDVSWQISIAVPQLY

RRGILRLEWTQWKFWGYMLDGLFQSVICYFFTYLTFYKGHVTTNVGREINYREAYGAYAG

TASMIACNIYVQLNMYQWSKPFLIICWVSSALVFAWTGIYTQFTASQLFYKTAQHLYGAL

NFWTCLLLMIIVCILPRLLGKCVHRSWFPMDIDIVREMWWAGEFNYLEGQDIDTIVSETT

QNYGTSAREKPGYDQPPTDEYSDPNTDRVYKSERNSFMESATFPGSDYPVKGYSTSPVEM

DNLEKRPQTDDSHLSPFADPNPNTSSPMQPAGRDVLHSDDDEHLVPK

>tr|Q6CF89|Q6CF89_YARLI YALI0B09251p OS=Yarrowia lipolytica (strain CLIB 122 / E 150) GN=YALI0B09251g PE=4 SV=1

MVIPDISYQDGILAALPIPKGAATGTGLRPLLKMVTELLGNIRIGQYLIDMVIGSQMAIF

GGVPNPDQDRAPCILFIVLHCVFCVCNGLIFLYNLYHRHLFLLTGCNACYNLVCAIGFGL

RLKWCDYTIQYLVAVFSVCLPIGTSFYLNFCNTVLGHRVFTKRHPETGNSSWFHVVMTVF

YCLIIGVVVGAILGQALPYLYFMGPKHYQMCRNVAKAMGIICILWALGGLAMVLAAYIIP

VGAVGHEFPGFKAKRAKNLRQTTSPFWITSFGIFYYPEKPKYKTFTKRSLEEDERHGASN

NNTLFARFVDSERRNPVRVISDLNNNPTVCALLICFTSCVLTTITVCRTVSLFTDEFFKV

RQVGNSPVFQNYTLYATFGALECIVNALFLLFRIDLRFYIPDREDFWDWLDKYPLFRSGK

LGHIYDKSKEIDAVGLAHNVHQPQDLSSSAEKTRQSIDSLSSHNSSGGRRSTS

>tr|Q6C1L1|Q6C1L1_YARLI YALI0F15279p OS=Yarrowia lipolytica (strain CLIB 122 / E 150) GN=YALI0F15279g PE=4 SV=1

MSYQQANNDLLDVDDDFIIEEDTPAPSNSQNTTSVGSEQLVTRRFMGGDTLDEPVLRTLG

RDVRGVGNRLKEVVWPGSLSGFINPIDNSSQLIKEWDMWGPLVFCLILALSLGWGNSEAF

SGVFAITWMGQILVTLNIKLLGGNISFFRGLCIVGYSLFPVVLGSVVEIFIPWIWLSVPV

IVLNVAWALWSANNSLKHSGVLPGRTVLAAYPVGLFFVSLGWLCILR

>tr|Q6C1W2|Q6C1W2_YARLI YALI0F12925p OS=Yarrowia lipolytica (strain CLIB 122 / E 150) GN=YALI0F12925g PE=4 SV=1

MSEYELPDLVWVTTSGALVWLMVPGIAFLYSGLSRKNHALGALWTGMMTLGVSFFQWWLW

GYSLSFSAKDNGQHFIGGMEHVVAQHVWTSRSASTPTVPDVVCYFYQGMFACVTAALMIG

GGNERVRLGPLAVFLFIWQTIVYCPIAYWTWNPNGWAARLGSLDFAGGGPVHMCSGSAAM

AFALVVGRRKSPACDFVPAFRPSSVAYIIMGTIMLWFGWLGFNGGSAGGASVRGIVAMVN

TNLAASCGGLTWMMVDYFRHNGKWTAIGICSGAIVGLVGVTPAAGFVPAWSAVVIGIVSG

VAANLGIGIKGVVYIDDGLDVFALHGIGGFTGSVMTAFFASDWVTKTDGVTVIQGGWVNG

HYVQLGYQLASACATIGWSFAITYLILITMDLIPFLTLRMDAVDEQLGTDVTQLSNECPE

QWEELTLYLRPWLENVEKEKQERMAQEAMHQVV

>tr|Q6C677|Q6C677_YARLI YALI0E11825p OS=Yarrowia lipolytica (strain CLIB 122 / E 150) GN=YALI0E11825g PE=4 SV=1

MDLENQKVPFQQVTTELSDSLQTISADVAKLDRFVSWIGTRKDGDTNRGRVTDLAEKITA

DIKTMHANVRRLNMFPEAELSNTEQFAQKRLSNEFGLLLSRFQNLQHQSTDAYKRQDTAA

RAALEEERSEQDRLLAAKPMGMNNTNYGGLQDQLLDVVDQSEVDLQQVLIAEREEDIRGI

EQGINDINGIYRDLGALIAHQGEQMDSVENNISTVADQTSAAAGELVKANDYQKKRRTCS

LIVLGIMILALIIILAVAA

>tr|Q6C8M0|Q6C8M0_YARLI YALI0D18568p OS=Yarrowia lipolytica (strain CLIB 122 / E 150) GN=YALI0D18568g PE=3 SV=1

MKPSPSTRSVEKKTSGIDPSIDKDEGLVEYSEVVIGGYSETWSGYSDEYNRAGLRIATDE

DCHTLRKVAAPITGMTYMLSLVEFAERGSYYGLTNVISNFVQFPLPKGGNGWGATPRGSQ

LTAGALDQGLQVATALTLVLNFLSYLTPLLGAYLADSKYGRFRTIWAGTVICGIGHTVIV

IAAIPGVLEHQKASLGVFIVGLIIFAFGTGLFKPNLLPLLLEQYREDDNWVKRLPSGEEV

VIDKESTLQRMTLVYYWSINVGAFLGLGTSYAEKRIGFWLAFLVPTILYFFLPFFLFLIQ

NRVYKIPPTEDSIIAGFFGVMYQTILRKPSTYPDKFVADVWSTLKASRFFLFFPIYFLND

TGIGALSNSQGSSMITNGVPNDLLNNFNPITIIVAIPMVNYGLYPLLRRYKINFRASLRI

FLGFILGACSPMIGAILQWRIYETSPCGYHATGCNKGVAPITIWWQVFPYVCTALSGIFA

TITSYELAYSLAPTHMRSIVMSLLLFMSAFSTAIATGITPALRDPYLIWPFVGLSAGGGA

FAVAFLCCYWRLGEGQKLSV

>tr|Q6C9K2|Q6C9K2_YARLI YALI0D10549p OS=Yarrowia lipolytica (strain CLIB 122 / E 150) GN=YALI0D10549g PE=4 SV=1

MTDTKEQSSLPEKDPAMDPDTTPTTPTSDLTSRNVSTGTGVLLGHYWGRSKEGSVIPGPA

TATVSSDRPKVIANDIDKADVRISILLLLASVLVRFYDLPNPPATIHEETAVGAYINKYI

GRAFFPTSEPPLAFLMYYLVSLFFNYDGNFPFIKQDHIYVGSRVPYIELRSFAALSGVIL

VLSAYYTVRLGGGHKLGALIASFFVLFDTSLATTSRYIFAEPLYLMFVSLSLLFWKLFER

QQPFSIKYYFWGLTLAVNLGVVVSTRWIGILTVLYVLVASCYNVFWILADLNISWWAYIK

HITFRAVAFTLIPLAVYATVLQAHLLIASGPHDGDYLLAPAHQDAINKHRRPPVHADIVS

GSVLTVRHLDTHSYLHSHDEFYPVGSRQQQISLYQHTDLNNVWVMENATKPNFEENDFLN

NFKHGDSVKLRHLQSTRRLHSHEVKAPVSDNDYQFEVSAYGADGFPGDLNDMWSIEIVAQ

YSTPGLPSRQMRTLQTVFKLRHLIQKCYLYGHRTQLPAWGFAQQEVTCNRYPAGTGALWY

VETNYHPNFPKEVNVTRDMIHYGNMTRVQKLKDMHNVIKVSREMTPDQSAFNTPPVSWPL

GMAGLPFYRSHHRQILVIANLACWWFALASLAIYVLFKAVVFLRWQRGLQPVALSADLIR

FDHEMGHALIAWACHFVPLIPETQAYTFITYLPAFYISCLAAGQMFSIFFKTFGRSKVIQ

VLVLAIVVVLAGGFYQEYRPIVAAKPWTREECKSHKYGLIDFDCDHYFATQEEYNVYDSQ

NIVNYVRFSEEMYQKLEGEATGPARPLQTLFKAREDKPNHDLGRPEVINAIRLHELERQN

KHKEKAALEKSISAEIKKKAYAEAGIGEASEEEDWTDAEAAAAAPIDKKPTASYDDELQK

KIQAQWARITDPPSLDLPDELKSQFNMSKFAKPKFEEVDLNDEDEYDDIMAAGDEEIAQE

IKGEDIGHESIYEPPVYEEIIDVNTD

>tr|Q6CA69|Q6CA69_YARLI YALI0D05401p OS=Yarrowia lipolytica (strain CLIB 122 / E 150) GN=YALI0D05401g PE=4 SV=1

MNLEELDKKAKYVFDTKDRVAEKFFESLPPKTRGLMKFLFSPDHDYDSAIEHFDDTSSTD

DIQLTHAEIVRRVWSKKQLIFGWICMLIMSLVSTIQASAFPSYIVYASSEFSQMGALASV

QIVQACIFLVSRALSGKLADNFGRFEAMIISVICLTIGNGLYMGSHNIVTYFAALAFYSI

GDIGTQMLYEIFAADTCDLKDRTFFQTITLFANLFTPWVAGPLVASVLKTSTWQWGMGMW

AIITPCCAIPFLSTLLYFRIKAYRMGLRIRRGDPNRTWFQNTITGIIRMDLPGLVFLCAG

LILFFVPISFCVGTDDWKMPQNIAMITCGTFFLYVAFPLWEFFGTKHPFLKFSLMKKRLV

TVPGLIILLYYMAYATYHPYFQVWLLLAKGLPQARATNLNMAMFVALTGTTLVFSPLMRW

RAKLKSVIVWGTILYFIGMGLTYEFRKPEKPLNTMILAQIFEGVGAGLLVIPIMVYIQVV

CKQSDTAAAITIYYFFNSCGTILGNVISAAVYRNHFPNVLRKTGLFAEPEVKAIFGSIYV

GLSYKVGTPQRAAIGATLNSVIRSLLIGPLIFCAIMFCLAITNADINLNTAQRMKEESEH

VKGDDSQSSDQGVVIETAYEEKSPIYDEKKVTEVVNKA

>tr|Q6CAG0|Q6CAG0_YARLI YALI0D03091p OS=Yarrowia lipolytica (strain CLIB 122 / E 150) GN=YALI0D03091g PE=4 SV=1

MESSPDFLPESDGDTEVELLPNLKLAGVTEPRRREKPVLKPPKTADAENIPLPPSPPPLS

SPSEIYSNLLILEESLRQQYLNQRQSQRKYSTFYFVLLSLTAYLVYSQIYPSIYSYVFFF

HRFCLIAVLSTLGLFHLSGMYTKKLVYPRKFIYNSNRGLRGFNVKLVKTGGYFSSTVHLV

LNTRVFSTENIEQWEEYRLGYFERERKKKIKEKKERKERKEPVRS

>tr|Q6CCC1|Q6CCC1_YARLI YALI0C10736p OS=Yarrowia lipolytica (strain CLIB 122 / E 150) GN=YALI0C10736g PE=4 SV=1

MLRPTFRTLPKVNGGLLTPLSRPALTPLSRPTLTPLSNPASMAGSRTFLFRKREPHPDDN

DPILKHIPRFIRPLARRFKNAPVSYVTSFLIVHEITAIIPLFGLWWYFNRYDFVPPGLPD

WLIVKGMSVIDKLLETQGWSFLNMENSARVVMQGAAAYALVKATLPVRIPISLLLTPPFA

RWIFIPTTRGVGRMFKNVFSRKKSTKVDTKPVEQPKIESLKTEPPKSVFNPKPGVSKPAE

PPITNVHGGPKQ

>tr|Q6CFK2|Q6CFK2_YARLI YALI0B06248p OS=Yarrowia lipolytica (strain CLIB 122 / E 150) GN=YALI0B06248g PE=3 SV=1

MLGRTLLVSDHCVDITASIFPGSSHPFTTTNPAMNTFHLLIALATFVVVYILSSSFVYQR

RTNALEKQWKCGKPLYYASFPRSLWHFYVFLRESRKHKLLEGFQRSFEAKKPFLTNKVNI

FGKDIFNTCDPENLKAMLATQFKDFCLGERHAQLFPVLGDGIFTLDGNGWQHSRAMLRPQ

FARDQVSDVHMVETHLKFLTSRITGSKPVDMQELFFNLTLDTATEFLFGQSVGCQIAESD

PGAYSSDMPLDLRKSFQKDFNKAQEHLGERVRLQMLYWLWNPKELQTAGARVHAFVDHYV

SKALVEAEEKVDDDKYVFLRELARETKDPKVLRDQALNILLAGRDTTASLLSWCFYLMAR

DDRVWQKLRSEVIEHFGDGENLENITFESLKRCDYLRYVLNEVLRLYPSVPANMRFATKD

TTLPRGGGPVGQDPIVIRKGNVISYHVFTTHRLTQYWGEDAEEFVPERWAEGKARGWEYL

PFNGGPRICLGQQYALTEAGYVLVRLAQMYDTLENADDKPEPPVKLHALTMSHLTGVHVK

LYKKN

>tr|Q6CHU1|Q6CHU1_YARLI YALI0A04983p OS=Yarrowia lipolytica (strain CLIB 122 / E 150) GN=YALI0A04983g PE=4 SV=1

MVNLSLRPRPAKAKFKGLPANLEVSPEDTVASVVAKLSAATKLSKSRIRLTVADEENGGA

PGAKKKHIVLKPEHAVGDYLFSDSPVVFVKDLGPQIPWRTVFILEYLGPLLAHPIIFFGQ

KFFYRQSFEYTFAQKLVFTLCMLHFLKREIETIYIHKFSSATMPLFNLFKNSGYYWFIAG

FNLAFFVYAPASFSSPQAPLWKRFLFSTGFFERTPLFLNLMAALWLWGETSNFWTHFNLA

SLRNDGSKDHKIPFGYGFNLVSCPNYFFEVVSWIAIALMCGNWSAYVFTAIGFGQMYVWA

VQKHRRYKREFGDRYPRNRKVMVPFLL

>tr|Q6C7L8|Q6C7L8_YARLI YALI0D27038p OS=Yarrowia lipolytica (strain CLIB 122 / E 150) GN=YALI0D27038g PE=3 SV=1

MKEAGDSSTTPPKPDTTSAYQVGGMTCGSCVSAIINGLEACPGVTEAAVSLVTERASVHH

NKSIISAEELQERIEDCGFDASLIDSSPIAAPVSTPMERLKVKIFGMTCSSCTNAVRDTI

QDIRGVANVVVALATEEATISFNPQECGARDIINAIEDCGFEGVLSAQQDNATQLASLSR

IKEIQKWRSDGIQCFILGLPVMLLTHILPMVGLQPLHDLTIFKGLYVDDLVCFVLATYIQ

FWLGHKFYVSSRRALSHGTATMDVLVAISTSSAYFFSVFSMLYAIATVADTHPHTLFETS

AMLIAFTTLGKYLENRAKGQTSGALSKLISLTPTTATILKDSSKYDPSIVYDESAEMDIA

AELLQRGDIVILKPGAKVPADGVVVSGETYIDESLLTGESTPVVRKVGDQVVGGSINGSG

RIDFRVERAGKDTALANIVRLVEEAQTSQAEIQRYADKISGVFVPCVVALALLTFIFWII

MSNVMKHPPNVFSLPEGKFLICLRLCISVVVVACPCALGLATPTAVMVGTGVGATHGILV

KGGAVLETASKIKTVVFDKTGTLTTGRMTIQKHVFEKDTLKNLNMTETEMWLILAGVEAS

SEHPIAQSLVRQAKEAAQVEDVPGVADFVAIVGQGVTGVVDGHSVAVGSSELVNSSCKSL

DKPPATPHSPDNPATVIHACVDGQYVGYMAFADSVKSDARAAVSVLKKMGINVAMMTGDN

HFVAHAVADEVGIPRSNVWASTSPAQKLAIIEQLQEPQDPNEAADSTDLSLNASVVAMVG

DGINDSPALAKAAIGIAMSSGTDIAMDAADIVLLNKESLMDVPASINLSQVTFRRIKINL

VWASVYNLIMIPFAMGCFLPFNFMLHPMEASAAMALSSVSVIVSSLALKKWQPPKDTEYG

SDEWRDMESQTVKPKRRWWQKLWRSEGVRYERL

>tr|Q6CDF9|Q6CDF9_YARLI YALI0C00847p OS=Yarrowia lipolytica (strain CLIB 122 / E 150) GN=YALI0C00847g PE=4 SV=1

MTEEKLASTTINPTDTVSNTAVSNTAVLTCPDTPASSADLESLVSLSDKDPYSRFPFRQK

CIFTLVVACVFFQPFLVAYGLMPALDKIAEEFNVSDTVVIIGNALFYIVQGLSVFFVGPV

CETYGRKAGLLTCCFFFCVSGIGLALSPNLACYYVFRAMSAVGGNSTFSVGVAVISDIWK

PEQRSKAVGCCLAGTQIGSSLGPVLGGLIVMKSNWRNIFWMQCGIGGLAMTIVAVVMRET

KPNTPFSQRKPGQWFFPLHCTHILKGFAKKHIMMVTVSLIFALYINETLIVPIAAIMKPR

FNLENELILGLFYVPQGIGYFFGCLFGGWYADYQVKKWVKIKGRRVPEDRLRSVIIFTLI

TGPLFMMLYGWSLKKEFGGMALPIVAMLLTGFSLSIYYPSVNAYYADSTPDLGGAAVMIN

YSARNVGSCVTAASTLTAINNIGIGWAATIAAIVCIFAALPLLFLIYRGEGIREAEYGRT

KVLLEEKEKAEEDPCNKNVCVGEYGPMRAEA

>tr|Q6CER1|Q6CER1_YARLI YALI0B13728p OS=Yarrowia lipolytica (strain CLIB 122 / E 150) GN=YALI0B13728g PE=4 SV=1

MRAWVTTFVLSTIIQGVAAGTPSAQELGLTKDQWYDWLNAHYGLYSFMPDYDSNLAGLVL

FAFILLTHLALGAFWRQWWFGVCVSCGCVLEFLGFLGRFLSREISVEDESYYIMQIVCLT

LAPAFVMAGVYCILAKLVVVYGESYSRLGPIVYTIIFVVGDWVSIIIQAVGGGLAATQGN

SDSGTWIMVAGIAFQVLVMSIFFLFYFEFLFRVYTGRRNPEVLDSQAHRPDIEELKKGKK

LPIFIVGQTIAIILIYTRSIYRIIELAGGWHGRLVINEVYMLVLDGLMMVLATYLLAIFH

PGFMFGRVPMKANLHKKKLSNMKEEDLNSSHNDEERVEW

>tr|Q6CC67|Q6CC67_YARLI YALI0C12034p OS=Yarrowia lipolytica (strain CLIB 122 / E 150) GN=YALI0C12034g PE=4 SV=1

MVKVADLILRGLIFMFAAIIMGLAGSLASTHKKGHYNPQVSYAVFCGAWSALFGVFYPVL

ANFIEAIAFPIVILIIDFISWVLTLAGGAALATAIRCHSCGNMNYVNSNKVTQGSKGRCR

KAQATVAFLFFANFSFLATMILSAISVKQLGAFTLPGRSRRSAPRTGIPTMSQV

>tr|Q6C280|Q6C280_YARLI YALI0F10054p OS=Yarrowia lipolytica (strain CLIB 122 / E 150) GN=YALI0F10054g PE=4 SV=1

MLRLLRPQRALPSLLRPLSVARPLSTTSRLWLHQQSTDAKKDDEKLLPEDRNLRRYEGHT

FEPFTPEKVPNAPVTVDTELPNITSHKMRRWAGFGLFVVIMTVSLAFSFNYEKMSSSQVS

SCMYTLRKSPLANEELGSGIRFASAYTWVSGTIRPLVGKVDFSFPVKGNKAEAIMHFNSR

RASGREKFRVLEWSLTFPDGRVVNLADSLIDPLIIDEEEHLVN

>tr|Q6C345|Q6C345_YARLI YALI0F02739p OS=Yarrowia lipolytica (strain CLIB 122 / E 150) GN=YALI0F02739g PE=4 SV=1

MNSLAKVALAAYLGVNAKNLLFAWHVRFFYYFFRYLFLPENLLGYPEAELPKTPFEECRY

DSRCNLLELDINIHKSNATYFEDLDSARTKLIVWVLNRFLKDSKKNDGSWAFIPIGSVYC

NFKNEIAPFQKYTMVSRVIGWDHKWFFVESQFEMNDPKNPTIAATAITKYVIKDGRRTVP

PGEAFRRAGYSEEDLVKGISEFKRLGLQRFIDIEEIGATPRAKL

>tr|Q6C4H8|Q6C4H8_YARLI YALI0E26653p OS=Yarrowia lipolytica (strain CLIB 122 / E 150) GN=YALI0E26653g PE=4 SV=1

MADFGDADLDGDLSLGFSSRREEPRIEKKKLDEEPDGDLASMRFDLDEEETDGEPEDEQA

TESHDIYSQVNASTATITGPSATTTATASPTTGYVVPQQVTSPRRGIRPILPISTDYVEE

QPRRQSRDDLMTPTEANRPSFGSSRSARDTTPKKQSTEQATRQRTPSHSPSHTTHSATPG

DHSMTFEEEFIQREGQMPFTQLSEKEIHQKQPHEKKVSHHVRGPSDATTDSSRSETSEEE

DDDDEGEWQDMLTVASYDVYDDRGNVVVKNKDNLEVEEVDPTARSGYTRVTRDEDAKSIN

SMDENTSYLFDDDEMARNPLSQMQATKGLLSDSQRIGYVGLCKLMLIEMARELASLKGSK

KIGRGLSDAQGSMAMWSQKMMFRLYSHMELSPEEQIMIEQLYSHGVEPRDITPSLIGVKR

VKNPLNEENQAEMEAAEEEKKEEKKRAKEESNEKGQGDTQAKGEWGEENDPGSTEIQVDD

ATAEADVDVSGDDGQLSKFSFDGKTSFDGSVPDINEDDATKVKIGGAEVVEEMENGDLAR

VETRVGVEEKNLDGDSSSTRSSSRPSSTYSDEYDDRVYAPEELEDKKMLTIDIRWTLLCD

LFLVLVADSVYDARSRTLMQKVGEEMGVTWLEMAQFEQRVTDALEMEENTQQTWDEKEIM

KTRKKKNLRKKYMYVGLATLGGGLVVGLSGGLLAPVIGAGLAAGFTTIGITGTGAFLGGI

GGAAVVTTAATALGARVGSASMMRRMGSVKTFEFKPLHNQKRLNLIITISGWMLGKEDDV

RLPFSTVDPIMGDLLSVLWEPEMLQSMGQTINILATEVLANSIQQVLGATVLTALMSSIQ

LPMILTKLGYLVDNPWNVSLDRAWATGYVLADTLIQRNLGVRPATLVGFSLGARVIYSCL

VELARRGAYGLVQDVYIFGAPVIVKTDQLCLCRSMVSGRFVNGYSRKDWVLGYLFRATSG

GLGRVAGLTKLESVEGIENYDATDRVDGHMAYRKAMPQMLKDLGWEVLSEDFAEIEDPDP

EKHRERQRELIQEFDIARKQMEAELKLEEEKESKEKKKKGFFGSWKKKQPKKKSWWDMTS

KVAEDDINEGNITRKDGEEGDGGEEGKECKEGEEGTTKDNTNPEGADNTLFDLDAIQREV

LRLENDPKLAAARRGSVPDVPITGRPRSGTNGSVSSQATTFVEYNPFTDGNTEQIEMSFE

PFQDDHDDRVGSIGQKISAGVASVASSASAGASAVANSFSGDTVSPADSTTAVTNGNSSG

AATNGRVPAAAAAPPAAAPPETPFKKYIDPESNRPKSKSRSPTPKEGEIVMTFD

>tr|Q6CA44|Q6CA44_YARLI YALI0D06017p OS=Yarrowia lipolytica (strain CLIB 122 / E 150) GN=YALI0D06017g PE=4 SV=1

MFYVVAALVALILAVYAAVFAFSHKPRPVHKHEQFFETTVADGSVSPKQPLVALEKQAHG

RKDTDADDDDDYVFISVVVPCYNETKRLGVMLEDAVPVLDALKQPYEVIIVDDGSRDKTP

EFALEWASQHMKPGSLRVTRLAKNRGKGGAVAHGMRFSRGKYVLFADADGASDFKDMPRL

LEAVKVNDGVAIGSRAHMVGTDAVVKRSFIRNFLMRGLHLLVWTFGVRTIRDTQCGFKLF

SRRATENIFPYMHTEGWIFDVEVLMLAQRKGLAIAEIPISWHEVEGSKIDLAADSIKMAI

DLVVTRVAYIIGVYGDREVNFKVE

>tr|Q6CDD3|Q6CDD3_YARLI YALI0C01411p OS=Yarrowia lipolytica (strain CLIB 122 / E 150) GN=YALI0C01411g PE=4 SV=1

MPRRATMRSVYSESTHSMASYDNRSSDFSEDYTTEGHITENAATDGHYTTTDDDSYDSYY

DGGDNASTMRMAPDTSSLAAVNSSVDWIREKDEAIVNPGVAPDLQGDGNLHSIPPDHQKY

EHGAPAFTTGKMSAPYNQKSAPYDGQEMSGFVSPQVRYRMALFPEEIGSGNVPLEYTDEH

QKHLFETYFDWVPHPQIVIPINLYEVVEVFDLLQSKFGFQVQSMRNMRDHFMCLLDSRSS

RMSYNDALLTLHADYIGGEHSNYRKWYFASQMDITDKIGGINVDYSGKLTKAGRRMVATD

TVWNEENANFSYEHSNRNWKNHMATISPKDQLKDIALYLLIWGEANQVRFMPECLCFLYN

CARDFCYSTAFATAPDVEDGVFLDTIITPLYSFYRNQRYENFEGKFIDRERDHKDVIGYD

DINQLFWYRQGLLRIKLKGGTNRILDLPASERYNALSTVDWTTCFYKTYHESRSWMHLAV

NFHRIWIIHFCVFWFYTAFNTPSLYTENYSQELDNLPPAHVRISVVGLGGVMAPLICLVA

VMGEAVFVPMRWPGRERVAYRLFCLLLVTSLNAAPAVFVLLWYSRTEENGQALMISIIQL

VIAFVTVLYFAFTPLKSLFTFFPKDKFNRRQLPTKFFASSFPPLKGNDRWMSYGLWVCVF

VAKYIESYFFMILSLKDPTRELGLVEYDKCVGAEYVGKILCKYQPLFVLACMFVTELVLF

FLDTYLWYIIFNTTFSVIRSVYLGGTLWTPWRNTFSRLPKRIYSKILSTSHLPSNRYKKS

YLVSQVWNSIITSLYREHIISQEHAHRLAYQQEIDGQGMCVLSEPKYFASQEDQSFHNSV

FDSQTEGERRLSFFAQSLATPIPDNYVIDEMPTFTVLVPHYNEKILLSLKEIIKEDGENS

RVTLLEYLKQLHANEWDNFVCDSKLMHDFMHNNGGEEVQGSYQEKKDGGEDGLLNVPEVI

HKRDQKSGKYDNLPYYCVGFKFSSPENQMRTRIWASLRCQTLYRTVCGFMNYSRAIKLLY

NVENPELLHHCQNDTRVFNQHLDMISRRKFRLLVSMQRLSKFDVQETENLEYLLKMHPEL

QVAYLDEDPSQGGREPIVYASLIDGDSDILDNGRRKPRYRIRLSGNPILGDGKSDNQNVA

LIFHRGEYIQLVDANQDSYIEECLKIRSILAEFEEFPAGNVPASPYASPKANEKNPDTLA

NPVAFIGSREYIFSENIGVLGDIAAGKEQTFGTLFARTLSKIGGKLHYGHPDYLNATFMV

TRGGVSKAQKGLHLNEDIYAGMNALMRGGRIKHSEYVQCGKGRDLGFGSILNFSTKIGAG

MGEQMLSREYYYLGTHLPLDRFLSFYYAHPGFHINNMFIIMSVEFFLIVGINIAALYSSS

VICEYDRSAPITAARVPEGCTNVIPIIEWLERCILSIFVVFFMSFVPLFIQEFSERGFLR

AATRLAKHLACLSPLFEVFCCQIYAKALLQDLTIGGARYISTGRGFATSRIPFVTLYSRF

ATASIYFGAISLLIMIVISTTMWRVALLWFWVTAVALCISPFLFNPHQFAWVDYFVDYRN

FIRWLNRGNTKWHKSSWIGYTRLIRTRITGYKKKTLNEISETDSRGVMKPSLVNVFLSEV

VGTLLSACCITLPYLFMNYQNEQIDGTPSNPLMRLAICTLFPIVMNIVMELVLFGVSCLV

GPIFSVCCKSAPGTFAAVVHTFAVLNHLVAFELMWFFQRWNVPVTLLGFISCTLIQDFIF

KTIITLFLSRELKHDHTNRAWWSGKWFTAGLGWRILTQPWREMLCKKIESSMYALDYILG

HALFFVQFPLILIPFVDRWHSMMLFWLRPSRQIRSPVFSTRQKRVRRRIVTRYAILFALN

LLAMTALFVLPIVFKDTLDIELDNHVPWFLRGLQQPLPNARLPLGKLPEKKIDV

>tr|Q6CFF1|Q6CFF1_YARLI YALI0B07601p OS=Yarrowia lipolytica (strain CLIB 122 / E 150) GN=YALI0B07601g PE=4 SV=1

MAIPMLGSLAALVLAIFVMVGSTSNSAPINDLYFLRIDTTNLSVSSVVPDINGVDVSSTV

NSIAHEVGISDFYTSGLWNYCTAKKPDPNNENLMNYTFCDKPKAMYWFNPEEILSESLSQ

GPSLTSELSAVMPEDVKKYLDILAGASKAMFVCYLVGTIFAFLTFVLGWFTFYSRGTTCC

VATLALLTFLLLLVSSGISTGTYMIVRDAFNNNLSEFGIRGSLNTKMYGITWGAAVAAAW

ACFGWFFAICCGNTDRVKVVTEEKQPFIGYVPDPYQPHGHQEGHRI

>tr|Q6C7T8|Q6C7T8_YARLI YALI0D25454p OS=Yarrowia lipolytica (strain CLIB 122 / E 150) GN=YALI0D25454g PE=4 SV=1

MRFYVILVLLTTLVLASVGDRSPDFRNCVTNCIRHTCQTQKYVPPLMHRLLLWDCPQECD

YRCQQIITFARLNQGQEIVQFHGKWPFFRFFGIQELASVVFSLANFVPHYRGWLMLKHLN

QRKPNPLIPYYIGFALVGMNSWIWSAVFHTRDFPVTEKLDYFSAGLSVLYGFFFATVRIF

RLDRDSRETTRLVLASVCVTLFLAHVSYLSFIKFDYGYNMTANVVVGALQLIMWSVYSFT

QFAKTHQWWSLMPFGLCVTISAAMGLELFDFPPWKFFIDAHSLWHAATVIPCFLWYTWMK

KDLQYEERAEKQE

>tr|Q6CGY4|Q6CGY4_YARLI YALI0A14927p OS=Yarrowia lipolytica (strain CLIB 122 / E 150) GN=YALI0A14927g PE=4 SV=1

MVLKFCSCSSQFLLCAVVILDVFQALQHLLLWWLVEFHLRAAPGGFSALFQCCVVGRWLS

GSLRLGLVLCSGGLAGWAQTLCSMAVFVDSNAKQAGSGLVRVPAVLLTVIAGVLILSQQQ

MEGTYDCSNDSPSLQSIQELLSMQGVAPKGATHTVGELLDNVSCPSLLHIHGDRIHEGLA

KPPTRHHPASS

>tr|Q6CIA4|Q6CIA4_YARLI YALI0A00198p OS=Yarrowia lipolytica (strain CLIB 122 / E 150) GN=YALI0A00198g PE=4 SV=1

MLNNEEDTQQQLTHTLLPALMANWFSMILRHGVMLSGLTVTIYRLQNQNLSPGEQGRRVM

ELVACLAAGILPLWTPPKISAAQLHLFQVSPYIFTAAILLASTLLLGVCQCWLLSVHPAA

FVLVLLLNSFLVNEIKWSEAFFASNWLPESQYYETIEKSKQLTILSTLISSGLVALFKTA

WFYLVLFAAVLYVSYMMAFVVDARLENQTKYKQMLEESEDFAPLQEWNDDHSSGVYAVIR

ERNWAAWGVYYFFQYL

>tr|Q6C3W8|Q6C3W8_YARLI YALI0E31581p OS=Yarrowia lipolytica (strain CLIB 122 / E 150) GN=YALI0E31581g PE=4 SV=1

MKPVCVLTLLLVALAAASWMADTWALMDVQRTLTPANRRQKHIHGCLKKVKTIAHKHKDD

SCTQCKKLVEYGHRVAKQQPHLGHDVIKKLCKTYADSKLCDKYNLGEEENDNVFNMTSTI

VRVLTLIDTSKGSLDAQYICANHFDGACDAPKTPKHDLEKLKWFKPRSEKEIEKYHRKLA

ENTEHLPPTFNVVHVSDFHLDLRYTVGSEATCSQDMCCNIENFHEEAPLNDTALSGHVVL

SPAREQGEYQCDAPRPLVKSSLEHINKTINEVGDFVFSLFTGDMVAHNAPEHKTLNYTLE

SELAVYKYMKNNIGNLPVFSSLGNHDSYPFGQLSQASTRHKTEDWNAQLASQLWEEYDWV

NTTQDELSNYAGYSVSPYKGLKVISLNSNYWYKTNLYNYWDIRNPDTSGMLRFLSDELKS

AEKHGQRAWIIAHIPSGGNSKNAVPWAGEVFATITERFDYVVAGVFFGHTHQDKFSVQYR

KKHPRRHKHSYKEKHAINVAWLGPSVTPIDDLNPSWRYYTINSSNFEVQDAHTYYTDLKS

LDYKWEHLYSSRETYNTFGWPEEAPLNATFWHRVAHQIKDDPEVRQLYTDLEARNSPFER

KCDSHKCITKQYCYITSWTTDRYDKCRKILK

>tr|Q6C4F3|Q6C4F3_YARLI YALI0E27247p OS=Yarrowia lipolytica (strain CLIB 122 / E 150) GN=YALI0E27247g PE=4 SV=1

METKDSNTHIESVEKLNDAVTSVEYLPSLEEKFPHVDGAKLLRKMDLKIVPMVTLLYLMS

FLDRGNIGNANIEGLSEDLGLTGQQFNLCLTVFFFTYCTFEVPSNMILKRLRPSVYLPII

MVCWGTCMTLMGIVTNYHGLLICRLFLGMTEAGLVPGVAYYLTMWYARKEMQLRQALFFS

AAGMAGAFSGLLAFAIAKMRGTAGLAGWKWIFIIEGIATVVVAVIAFFTIQDFPEDAKFL

SEEEREYLQYKLKYDGLDRDSAAAATGVHQSMGVNNSNDKKFVWQAFLDPQSWAIAFLII

FAATPIYSISFFLPSIIKNLGHVSSKAQLMSIPVSFTGAITTVVQAYFSDKYGIRYPLMA

LDFVSAIIGYIMALTTAESHPAVTYAGCFFIVGGTHPAFIAMLSWLSVNSAGTYKRAIAL

AIALMLGNMSGAVGANIYRAQDRPGYKLGHAINLAFVSAGLTLATITYLCYRAANRRRRE

GLAAGKYDHLTNEEIHDMGDRSPYFTYQL

>tr|Q6C711|Q6C711_YARLI YALI0E04664p OS=Yarrowia lipolytica (strain CLIB 122 / E 150) GN=YALI0E04664g PE=4 SV=1

MSCNHLNIYIIGITMYVFYSSGHPYAMAIHHSFKISKKKALPPAKSKATLRPKSTEPVPV

AANPLITAFEVDFSNDGPVADFQWDIETIEKSRVVFPLAPEAWETTFAAVHESDTTQTTQ

PPSEDDTTLEITEEKVATKIVPQKFIPGWMMVGDTGDSTDVDQSVDPPQCFDWVDLFFEP

KSATEYDPRNFSSHFQETLKVSQELYTPGSVVNPITEGYVISGKSTHAFFNPITGAMTPW

IHQPAPRASKDKSTNTTLRTIPNTDCLNVGDPSYWWNVRICSNYFHLGEASSNRVVAALF

STSYKASLSGPTEICIIRCFQPYVKEASYASQFYTFLPQKNEKYALALAGSPTSNLFLVE

SYKEDVHYIYLLTSSLNSPLPHYKLVLSVSHISRTQKNGNLRGKQWWHHRILTFGDNLVI

FKEGRLASWRIIYDCSTDSVKAYLIPEKYGFVADVHPVQFGKHLVFSRGVLDMSTFQFCT

VPVGQNDRKKIVWHKRNGVLVGSTVNSLSFQCFYLGDPSEV

>tr|Q6CG17|Q6CG17_YARLI YALI0B01672p OS=Yarrowia lipolytica (strain CLIB 122 / E 150) GN=YALI0B01672g PE=4 SV=1

MVEKNPASPKRTSKKSVPSPRSLLFPILLLAGILILLLSQTVGVLSIVESLASLKPAATN

PYPYENRKWIYDFTHQPWSGADTKDRRQIGRENATLVMLVRNRELKEAVLAMRSIEDRFN

KKYKYPWVFMNDREFTEDFKEYTTGMASGHTEYYKVPKEAWQMTPGIDRDLVFESVENFT

KEGVIYGDSLSYRRMCRFNSGYFFRMAPLLKYKYYWRVEPDVELYCDQQYDPFTFMRENN

KLYGFVMAMYEYPQTVATLAEHVQGFFDVHPQYVHPNSSVGFLKDKRVNRFGDLMPEWHG

EWNLCHFWSNFEIGNMDFFRSKKYLEFFEWLDRAGGFFYERWGDAPVHSLAVGYMMDKTQ

VHQFADIGYYHPPFFRCPADEQSHKSGRCACPEDLNGYKNFDYEPMSCLPRWWGNAGKAW

MKDGVHINV

>tr|Q6C3A8|Q6C3A8_YARLI YALI0F01210p OS=Yarrowia lipolytica (strain CLIB 122 / E 150) GN=YALI0F01210g PE=3 SV=1

MTKESVHNLPTVSTSQPAEPVDAIPPTAYVPPDEAYTRSRPMGLGENMRNLFIACIGEFV

GTFMFLLFAYLIATVANYDKTVAGPNAAKIIMISFGFGFSLLVNVFIFFRVSGGQFNPCV

TLALTLVGAVPPVRALCLAITQLLAGMAAAGVADALTPGPVTFINTLGDGVSRTRGMWIE

MFCTAQLCLTVLFLAVEKHRATFMAPFPIGMSLFIGHLVAVFPTGAGINPARSLGPCIVG

KSFPHYHWIYWVGPILGSIFSVGLYHTLKFLDYETSNPGQDNQD

>tr|Q6C3R0|Q6C3R0_YARLI YALI0E32813p OS=Yarrowia lipolytica (strain CLIB 122 / E 150) GN=YALI0E32813g PE=4 SV=1

MFRCCLRTVPLRAMLRPRAIHSMRPSFTNARQANFRVAGMATGLAAGFAVGCSMVLNDAP

QEKDDKKQDLTREEKFYIESQQQTESRKLPDYEDSSNKLYVFFRRLLTRLEVWIWDPIAT

TFRFFYLSILFAPVLLTLPVILIGPRDKSKDNERYTAIWWYGFLTKMMERAGASFIKLGQ

WAASRTDIFPPQLCAEMSDLHSNNKAHSLRVTKKTLEGAFGMPFEEIFDEFVDKPLGVGA

IAQVYKGKLSQKALNSTKQKKGERSPSGWVAIKVLHPRVSQIVERDLSIMRFFANIINAV

PTMEWLSLPGEVEQFAGMMRLQMDLQIEGKNLEVFNKNFQDKADIHFPHAFLEFTTRDVL

VEEYIDAIPVHLFLERAKDGGGFDKEIANKGLDAFLQMLLIDNFIHADLHPGNIFVRLYK

PESMMHHLGFGDHNNANEDYIEMQKVNDHLKSLKHDPAAWDKEIERLKADGYHAQLCMID

AGLVTELNELNRRNFIDLFKALAEFDGYKAGDLMVQRSRTPETVIDPETYALKVGKLVAQ

MKSRTFKLGNFKIGDLLTQVLAMVRQHHVRMEPDFITVILSILLLEGIGRQLNPDLDLFK

NAIPVLRELGTHSGDGRALFNNEDMMSMLKVWLALETRQFISASVEEVNQQVKYDGLCPN

V

>tr|Q6C3U4|Q6C3U4_YARLI YALI0E32065p OS=Yarrowia lipolytica (strain CLIB 122 / E 150) GN=YALI0E32065g PE=4 SV=1

MKFVGLISLLLVGLYMVTNWLFINVLPHRYIFDKDELMTMVNQTLERNPDGNSTAILTDL

GASLHDRYGKFINELNFDEWVFNNAGGAMGSMFVLHASVTEYLIFFGTAVGTEGHTGVHY

ADDHFMMLTKYQMAHTAGELEPEVYYPGSSHHLKRGVAKQYHMPGGAWALELAQGWIPAM

LPFGFLDTLSSTMDIETLWKTVKISAVNMVRNALWGKI

>tr|Q6CAP1|Q6CAP1_YARLI YALI0D01111p OS=Yarrowia lipolytica (strain CLIB 122 / E 150) GN=YALI0D01111g PE=3 SV=1

MGRNWLAFSQASTEVKTEFLGLRGQKLHRAVAFIAGMGFLLFGYDQGVMGGLLTLPRFIH

QFPKMDTSDYVPEETRKFNTTIQGVSVGIYEIGCMIGALFTMWAGDKLGRRRMIFWGSII

MTIGAILQCASYSLGQFITGRVVSGVGNGFITATVPMFQSECAKPERRGALVMMEGALIT

GGIALSYWIDFGFYWVNNDADWRFPIAFQIIFSMFLTFTVMSLPESPRWLVKKQRFDEAA

GVFSALEDVPIDDPYVIDQIAEVKESIMMEQLAQLGVDGSEAREKIASGEFQMGAELPFL

EQMKLLFTFGKKKNFHRTMLAYWNQVMQQITGINLITYYAAYIYETSVGMNATNSRILAA

CNGTEYFLASWVAFYTIERFGRRKLMLFGAIGQACTMAILTGTVYAASPPEDGGLDNSGA

GIAAAVFLFVFNTFFAIGWLGMTWLYPAEITSLEIRAPANGLSTSGNWVFNFMVVMITPV

AFDTIKWKTYIIFACINAAMVPVVYFFYPETAGRSLEEIDKIFAESNPRTPWDVVGIARR

MPRESALTRRKQGVNQTFDNSNEKAVVEENTESAVGSTTGSASPSFENVSQA

>tr|Q6CBC9|Q6CBC9_YARLI YALI0C19943p OS=Yarrowia lipolytica (strain CLIB 122 / E 150) GN=YALI0C19943g PE=4 SV=1

MTEKYIKILIIFKKSKITQHHFSVFIMSFSVALKSTLIHPHCTCLCLSHSKYLTYIFLPH

LMIRTNLSKTWFASSRLIRIAASPILIPARMIISGTFMYPFMGIYYFFTHPILWAYLFTI

FLPQIVLTMVVFFFMYLFFYPISAAFAFLFNGPTGLFTAWIALLQQSTVIAGILGDMFLL

PTPMKMLFDSIMSREGLDDIVVAGKQRRPTPVPPPQTRVKVLLKHLPLTLVFPTWLVRLT

VTVLLHFIPIIGPFVAILVNAPRRGRNAHARYFELKMMPKADVEQFTRVRRGQYLGFGMV

AGALESIPFLGLLFAFTNCTGGALWAVAIERRMRDTARPSLNNARARAQQMMIG

>tr|Q6CCK8|Q6CCK8_YARLI YALI0C08569p OS=Yarrowia lipolytica (strain CLIB 122 / E 150) GN=YALI0C08569g PE=4 SV=1

MSLKTELSHHEGEITPLESPDRFLTAEEKFPHINRSKLLRKCDLHIIPMLAVLYLMSFLD

RGNIGNARIEGIIEDLNLTGTQFNWSITVFFFTYCVFEVPSNMLLKHIRPSIYLPSIMVL

WGIVMTLMGIVNNYAGLIACRVLLGVFEAGLFPGVTYYLTMWYCRSDMQYRQAMFFTAAG

LAGAFSGLLAFGIGKMRGTAGLNGWQWIFILEGIATVVVAVFAYWALYDFPENAKFLTQD

EREFLQYSLEYDGYDRDLAAKGASSNQPVGRDNSNDKEFVWQAFKDVQSWLGALAMILIV

VPLYSLYFFTPIIVNSMGHSVAKSQLLSAPPALVGAIANVFQAYFSDKTGKRFPFLMANF

SLGLVGYSLCIAYGVKRIWITYAGCCIVNLGMQPCFICFISWISVNVSGPYKRAIAMAIV

IGLGNMGGAIASNVYRAPDAPAFKQGHTIAMSMVAAGMAIVIGMYLGYGFVNRRKTKNML

SGKYDHLTGAELARMGDRSPFFIYRT

>tr|Q6CDG0|Q6CDG0_YARLI YALI0C00825p OS=Yarrowia lipolytica (strain CLIB 122 / E 150) GN=YALI0C00825g PE=4 SV=1

MSMTSDEQIIDHSVQHDAEKTPISPNATPAVSDNENRGSDIEKSQPAVTTEPNGATADAP

SADTVAPIPPPDTGRAWLAMIGASFGLYSSFGYINVVGLYEAYYLHHQLSHYSASTISWI

TSLQIFILFVGGIFFGRIAEMYGPQKLAAFGTVFTITGIMTTSVCKEYWHFLLAQGICTS

IGNSAIYYASLLAATTWFQKKRALALGVVVAGSSLGGTTMPFIFTKLQPRIGFPQTVRAI

GYLMIGVCVICTLLVSSRFPPNKKLRKEFFNLREEVLVPYTKPSIILVTLAMFFSFWGLF

TAIGYMSTHAIAHGMSEETAYYLVSIYNGSSLIGRILPGFVADKFGSYNMHSICTVICGV

FLLAMWIPAKTNPVIIAFSSCFGVISGATVSLFPALVASITPPTEVGRRMGVVSFFYSFS

SLTAMPIAGQILHSDNDNFTGLQVWAGVTMLVGGFCVFASKMCIEGKTWRSKF

>tr|Q6CHT8|Q6CHT8_YARLI YALI0A05115p OS=Yarrowia lipolytica (strain CLIB 122 / E 150) GN=YALI0A05115g PE=4 SV=1

MTLASRIRRSRRVIILALAVVSLLIMAFIISKPDLAMKLGAAATESTRSRPSAFFDRTLD

FPGVESDTDHLVMVPCHGVWKQPRKSSTKLPGLAFSDWVAGPFLEGKTDILLKHITEGVR

RASEDPSALLLFSGGQTKKAAGPISEGTSYYQLAEALGLDMSQTAVEEYARDSYENLAFS

IARFRELTGRYPVRITVVGYEFKRARFEQLHRPAVGYDSDKFEYVGIDPVWGEDELPDAG

ELEHAFLPFQRDPHGCVEQVLLKKKIERNPWRRQHPYAHTAPEMKQLLLACNQR

>tr|F2Z612|F2Z612_YARLI YALI0E02222p OS=Yarrowia lipolytica (strain CLIB 122 / E 150) GN=YALI0E02222g PE=4 SV=1

MISRDKKPNELPKYEKTGGVDYRTDFKPRKPSPLSRPNLQVPQITKLRLAAVFVAVTLIV

FLFGSHHEVSYSDVLQSVQGYKQYVSDSIKQGSISSQSPAEAHRKPLGEVSFVDLRDAAG

DWSKNSPLNQRVLVCMPLRNVEKVVPIMASHLRNLTYDHNLIDLAFLISDTDDNTVNVLE

DEINSIQSDADPKMPFNKITLLFRDFGSAVGTDFSDRHGVAVQGVRRKLMGRARNWLLSS

LLEPTHSWVYWRDADIETSPPTIIEDLMKHNVDVIVPNVWRPLPDWLGSEQPYDLNSWQE

SQPALDLAATLDEDEVIVEGYAEYPTYRPHLAYVRNADGNPDEQVDLDGIGGVSILARSR

VFLSGAHFTGFTFENHAETEAFGKMCKKMGFTVRGLPHYTVWHMYEPSEDDLKEMMRREK

EEKEKEKQEKGTTENKDLEVNEAPEVKELEA

>tr|Q6C0W8|Q6C0W8_YARLI YALI0F21109p OS=Yarrowia lipolytica (strain CLIB 122 / E 150) GN=YALI0F21109g PE=4 SV=1

MDETKELSIEHKELAEIASITSPKNIGLPTAEEKFPNINRKKLLRKMDWHLMPMLTMLYL

MSFLDRGNIGNARIEGIVEDLGLSGTQFNWSITVFFFTYCVFEVPSNMLLKRFRPSIYLP

TIMVAWGIVMTLMGIVKNYGGLLACRLILGATEAGLFPGVAYYLTMWYCRADMQYRQAMF

FSAAGSAGAFSGILAFGIGKMRGKADLNGWQWIFILEGIATVVVAIIAYFCLYDFPETAK

FLTEEEREYIQYALEYDGYDRDLALKGGAQNSDAVGRDNSNNSKFLKAAFMDPQSWIGAL

LMILIVSPLYSMSFFSPIIVRSMGHSASISQILSAPFGAIGAITTVIQAYFSDKYKRRFP

FLMVNFSLGIIGYALCIKYGVNKQWITYAGCMIVNMGLQPSFICHISWMSVNIAGPYKRA

IAMAIVIGFGNMGGAIASNMYRASDAPHFKQGHTIAISFVAAAMAVTICLYLAYNFINKR

KAKRLLEGHYDQYTSEELSAMGDRSPYFIYRM

>tr|Q6C170|Q6C170_YARLI YALI0F18744p OS=Yarrowia lipolytica (strain CLIB 122 / E 150) GN=YALI0F18744g PE=4 SV=1

MTMVEDIVGTKPGAVYKTGHTVKFLVLFAIKNWFIIGMGVVILLAYLFPNVARSHGVLRA

DIAFSYCAVAIIFLISGLSMPTKVMAKQAKHWRAHLITQGLSFVITPAVMFGFVVAIHKS

GNKNMDPWVLVGMIIAGTTPTTVASNVLMTRMCNGNESLALLEVTVGNLLGSFISPGLIQ

LYLSKHTGFAYGNPANHMSIRELYAKVMKQVGLSLFVPLFVGQVVQNVFPKQTKWAVTHL

YLAKVGTLMLLLLIWSTFSTAFYDKAFEEVHYTTMVMVCFFNVGIYLLFTLLCLGAARLP

VNHLPKPTENSGKFYKWFYRMARPFHFSRKDTCAIMLCGAAKTVALGAPMISAQYGSQSP

VIGKVSIPLTLYQGEQILVAQFLVPVLKRWVAGEDDIPKPEDIEAPAITSSSSGDDVGHE

HTKAERRPTREPSVQPEAVAEK

>tr|Q6C3F5|Q6C3F5_YARLI YALI0F00176p OS=Yarrowia lipolytica (strain CLIB 122 / E 150) GN=YALI0F00176g PE=4 SV=1

MSLTSREIRMIALLIIDTCFFLLEAIVGYAVHSLALVADSFHMLNDVFSLIIALWAVRVA

KSRGADSKYTYGWQRAEILGALANAVFLLALCLTILLEAIQRLFEPQIITNPKLIAVVGT

AGLCSNIVGLLLFHEHGHAGHSHGHDHDHDHDHAHDEEEAVDTVLASQFTAPTEQTSLLQ

HPTSHRRSISNIDSSEHATHFHAKKKNEQKKKVSLNMQGVFLHVMGDALGNIGVIATAFF

IWKTDYSWKYYADPVISLVITVIIFSSALPLCRSTSSILLQAVPQNINAEDVKNEIVALD

GVEELHDLHIWILKEDTFVATLHVGVASDPSEFMTLSNDIKKIFHEHGINSVTIQPEFNV

ATGSTTPDKHQYHVSVGGLRSANSNGCLAPQ

>tr|Q6C6M7|Q6C6M7_YARLI YALI0E08096p OS=Yarrowia lipolytica (strain CLIB 122 / E 150) GN=YALI0E08096g PE=4 SV=1

MKVSTVLVTFLSASSASAKTYRRQDSLNNKPYWAALGCDHLIGGHYKFNFPKFDSSTVKS

RKQILEEYCKVMPFIQSKLLCYQGAIEPEHYHTQVRKQIIQGCPNYLTLEMTDEWLANAT

KIAVEPEDPTEILYLPVPFDREHWEEEYEEVKPHYVALDYASYYGVALLAYWALVFVIGT

IINFSQHALFSVYKKVGFNKFRKYVSLPALFGYKHSQPMGWDPLKMASPTRIQSLVVLGY

LIMAFVLCFPSYPFDDDYEEAGPWGAQLEAFVADRTGIMSFTQAPIVFLFAGRNNLLMWL

TGWSFDTFNVYHRWTSRVMMIYAIIHSCIWTWMCRHSLARDAAELYWVLGTVATIAGSLM

LLQAMHVFRSRWYEIFLVLHIVFGILFVVGLWYHCWTIGWMQWVWATIAVWGFDRIIRVC

RIAYCGGIVTGDFTLVDREQLIVKAEIPCSRLWSIYPGAHVYIYFLSGNKFWESHPFTIY

QSPEACKNGNMTLLLKAKDGITFDIVTRLVSAGGARPMKMLIEGPYGAKHPVGKYDSSIY

IAGGIGITATYSYAQKVVANSVSKSIIFTWVVRGDSCLEWFGDELEYLLADPRVRVNLYI

TKIDKKEGLAEELAEEACELESKSPEKLSITSSNRDNLNIQHFRPHMAELLPEYLKESPG

STAIVVCGPPAMNDDVRQALCANIESKSERVDYFEESFSW

>tr|Q6C8X5|Q6C8X5_YARLI YALI0D16137p OS=Yarrowia lipolytica (strain CLIB 122 / E 150) GN=YALI0D16137g PE=4 SV=1

MFPFDVEKNKGTSSESIHSIHVSEIHSDVRSESIIGAPEEEESPLGQHVGKFTVVALNFS

QMIGTGIFVTPGSILKGVGSIGASLMLWLAGIIISFSGFAVYTEFASMYPKRAGADVAYL

EKAFPKPKYLMPVVFAVISVLLSYSASNAIVFSEYVLVAANQEVTEWTQRGIAIAAIAGV
[truncated: 295,964 more chars]
